# Supplementary material for: Facile generation of bridged medium-sized polycyclic systems by rhodium-catalysed intramolecular (3+2) dipolar cycloadditions
Source: Nat Commun. 2021 Sep 2;12:5239. doi: 10.1038/s41467-021-25513-7 (PMC8413281; doi:10.1038/s41467-021-25513-7)
Supplement: Supplementary file 1 — Supplementary Information [file 41467_2021_25513_MOESM1_ESM.pdf]

Supplementary Information for

Facile Generation of Bridged Medium-sized Polycyclic Systems by Rhodium-Catalysed Intramolecular (3+2) Dipolar Cycloadditions

Bao-Long Hou[1,‡], Jonathan J. Wong[2,‡], Na Lv[1,‡], Yong-Qiang Wang[1,‡], K. N. Houk[2,*] & Chuang-Chuang Li[1,*]

[1]Shenzhen Grubbs Institute, Department of Chemistry, Southern University of Science and Technology, Shenzhen 518055, China.

[2]Department of Chemistry and Biochemistry, University of California, Los Angeles, Los Angeles, CA, USA.

[‡]These authors contributed equally to this work.

[*]Corresponding author. E-mail: ccli@sustech.edu.cn (C.-C.L.); houk@chem.ucla.edu (K.N.H.)

Table of Contents

1. General Information S3

2. General Procedure of Optimization and Substrate Scope S3

2.1. Optimization of Rhodium-mediated Type II (3+2) Cycloaddition of **1a** S3

2.2. General Procedure and Characteristic Data for Substrates S5

2.2.1 Procedure A: General Procedure and Characteristic Data for Substrates **1a-1n**, **1p-1r** and **1ac-1ah**. S5

2.2.2 Procedure and Characteristic Data for Substrate **1o** S18

2.2.3 Procedure B: General Procedure and Characteristic Data for Substrates **1s-1z**. S19

2.2.4 Procedure and Characteristic Data for Substrate **1aa**. S26

2.2.5 Procedure and Characteristic Data for Substrate **1ab** S27

2.2.6 Procedure and Characteristic Data for Substrate **1ai** S28

2.2.7 Procedure and Characteristic Data for Substrate **1aj** S29

3. General Procedure for Synthesis of **3a-3aj** S30

3.1 Procedure for Synthesis of **3a-3i**, **3m-3q** and **3s-3aj**. S30

3.2 Procedure for Synthesis of **3j-3l** and **3r** S30

4. Asymmetric Total Synthesis of Nakafuran-8 .... S56

4.1 Comparison of the NMR Data of Natural and Synthetic Nakafuran-8. .... S56

4.2 Experimental Details S58

5. X-ray Crystallographic Data S73

6. Computational Section S89

6.1 Computational Methods S89

6.2 Computed Energies S89

6.3. Cartesian Coordinates S89

8. ^{1}H, ^{13}C NMR and 2D-NMR Spectra S95

7. References S192

1. General Information

All air and water sensitive reactions were carried out under argon atmosphere with dry solvents under anhydrous conditions, unless otherwise noted. All the chemicals were purchased commercially and used without further purification. Dry dichloromethane (DCM) and acetonitrile (CH_3CN) were distilled from calcium hydride. Dry diethyl ether (Et_2O), tetrahydrofuran (THF), and toluene (PhMe) were distilled from sodium-benzophenone. Other solvents purification was conducted according to *Purification of Laboratory Chemicals* (Perrin, D. D.; Armarego, W. L. and Perrins, D. R., Pergamon Press: Oxford, 1980). Yields refered to chromatographically, unless otherwise stated. Lower temperatures were maintained using acetone/CO_2(s) (-78 ºC), salt/ice baths (-20 ºC) and water/ice baths (0 ºC). Reactions were monitored by thin-layer chromatography (TLC) carried out on 0.25 mm Tsingdao silica gel plates (60F-254) that were analyzed by fluorescence upon 254 nm irradiation or staining with basic aqueous potassium permanganate ($KMnO_4$) or an ethanolic solution of phosphomolybdic acid, and heat as developing agents. If not specially mentioned, flash column chromatography used silica gel (200-300 mesh) supplied by Tsingtao Haiyang Chemicals (China). Preparative thin layer chromatography (PTLC) separations were carried out 0.50 mm Yantai (China) silica gel plates.

IR spectra were recorded on a Shimadzu IR Prestige 21 using thin films of the sample on KBr plates and only major peaks are reported in cm^{-1}. All products were further characterized by high resolution mass spectra (HRMS). Optical rotations were recorded on a Perkin-Elmer 351 polarimeter at 589 nm, 100 mm cell at 25 ºC. Data were reported as follow: optical rotation (*c* (g/100 mL), solvent). NMR spectra were recorded on either a Brüker Avance 400 (^{1}H: 400 MHz, ^{13}C: 100 MHz), or Brüker Avance 500 (^{1}H: 500 MHz, ^{13}C: 125 MHz), and calibrated using residual undeuterated solvent as an internal reference ($CDCl_3$, δ 7.26 ppm ^{1}H NMR, δ 77.16 ppm ^{13}C NMR), (CD_2Cl_2, δ 5.32 ppm ^{1}H NMR, δ 54.00 ppm ^{13}C NMR), (DMSO-d_6, δ 2.50 ppm ^{1}H NMR, δ 39.52 ppm ^{13}C NMR), (CD_3OD, δ 4.87 ppm ^{1}H NMR, δ 49.00 ppm ^{13}C NMR). The following abbreviations were used to explain the multiplicities: s = singlet, d = doublet, t = triplet, q = quartet, m = multiplet.

2. General Procedure of Optimization and Substrate Scope

2.1. Optimization of Rhodium-mediated Type II (3+2) Cycloaddition of 1a

Supplementary Table 1. Optimization of Rhodium-mediated Type II (3+2) Cycloaddition of 1a

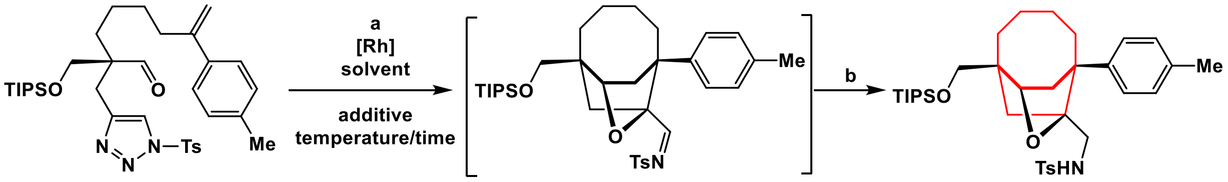

Entry	Catalyst	Additive	Solvent	Temp./Time	Yield(%)[c]
1	$Rh_2(OAc)_4$	—	toluene	90 ºC / 8 h	50
2	$Rh_2(OAc)_4$	—	toluene	110 ºC / 8 h	49
3	$Rh_2(OAc)_4$	—	1,2-DCE	85 ºC / 8 h	65
4	$Rh_2(OAc)_4$	—	$CHCl_3$	62 ºC / 8 h	20
5	$RhCl(PPh_3)_3$	—	1,2-DCE	85 ºC / 8 h	0
6	$Rh_2(esp)_2$	—	1,2-DCE	85 ºC / 8 h	0
7	$Rh_2(OCt)_4$	—	1,2-DCE	85 ºC / 8 h	71
8	$Rh_2(OCOCF_3)_4$	—	1,2-DCE	85 ºC / 8 h	0
9	$Rh_2(S\text{-}DOSP)_4$	—	1,2-DCE	85 ºC / 8 h	49
10	$Rh_2(pfb)_4$	—	1,2-DCE	85 ºC / 8 h	0
11	$Rh_2(OCt)_4$	5Å MS	1,2-DCE	85 ºC / 8 h	78
12	$Rh_2(OCt)_4$	4Å MS	1,2-DCE	85 ºC / 8 h	77
13	$Rh_2(OCt)_4$	3Å MS	1,2-DCE	85 ºC / 8 h	80
14	$Rh_2(OCt)_4$	3Å MS	1,2-DCE	120 ºC / 3 h[d]	80
15	$Rh_2(OCt)_4$	3Å MS	1,2-DCE	60 ºC / 12 h	53
**16**	**$Rh_2(OCt)_4$**	**3Å MS**	**1,2-DCE**	**85 ºC / 3 h**	**81**
17	$Rh_2(OCt)_4$	3Å MS	DCM	85 ºC / 12 h	<10
18	$Rh_2(OCt)_4$	3Å MS	DCM	40 ºC / 24 h	0
19	$Rh_2(OCt)_4$	3Å MS	hexane	90 ºC / 12 h	0
20	$Rh_2(OCt)_4$	3Å MS	$CHCl_3$	90 ºC / 8 h	<10
21	$Rh_2(OCt)_4$	3Å MS	THF	90 ºC / 12 h	0
22	$Rh_2(OCt)_4$	3Å MS	EtOAc	90 ºC / 8 h	0

[a] Reaction conditions: **1a** (0.1 mmol), catalyst (5 mol%), 3Å MS (30 mg), solvent (2.0 mL). [b] $LiAlH_4$ (2.0 equiv.), THF (4.0 mL) at 0 ºC for 1.5 h, under argon. [c] Isolated yields. [d] Heated in a sealed tube at 120 ºC.

2.2. General Procedure and Characteristic Data for Substrates

2.2.1 Procedure A: General Procedure and Characteristic Data for Substrates 1a-1n, 1p-1r and 1ac-1ah.

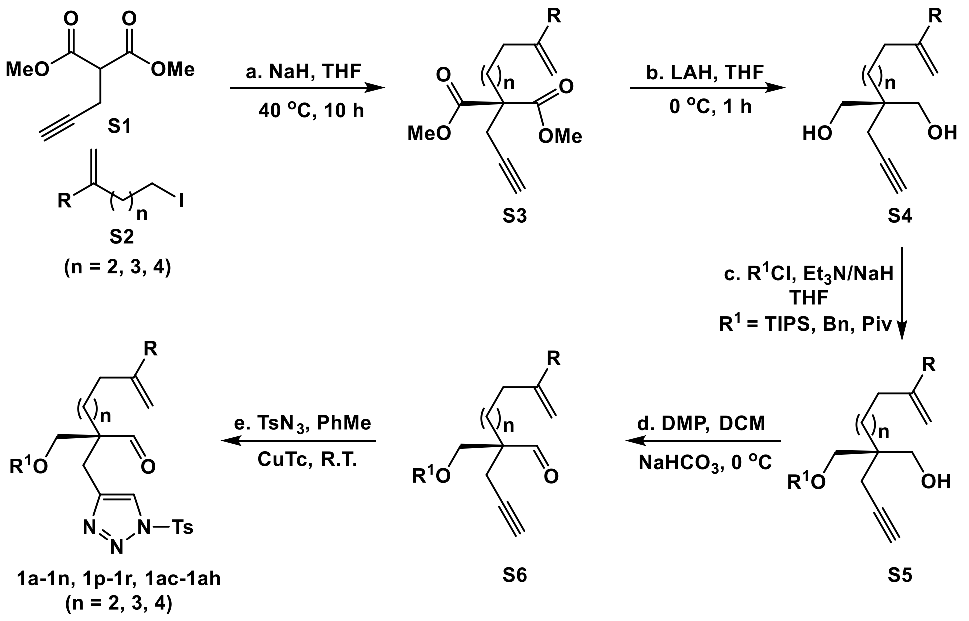

Step a: The compound **S1** (1.0 mmol, 1.0 equiv.) was dissolved in THF (5 mL, 0.2 M/L) at 0 ºC. NaH (60% dispersion in mineral oil, 48 mg, 1.2 mmol, 1.2 equiv.) was added slowly in portion-wise into the reaction mixture and stirred for 30 minutes at 25 ºC. Then **S2** (1.2 mmol, 1.2 equiv.) in THF (5 mL) was added to the reaction mixture and stirred at 40 ºC for 10 hours. The mixture was quenched by addition of saturated aqueous NH_4Cl (10 mL). The layers were separated, and the aqueous phase was extracted with EtOAc (10 mL × 3). The combined organic extracts were washed with saturated brine (10 mL), dried over Na_2SO_4, concentrated under reduced pressure and the residue was purified by flash-column chromatography on silica gel to provide **S3**. (Note: The iodide **S2a-S2h, S2j, S2l, S2n, S2o** were prepared by the method according to described in Ref.1).

Step b: To a stirred solution of compound **S3** (0.8 mmol, 1.0 equiv.) in anhydrous THF (8 mL, 0.1 M/L) at 0 ºC was added $LiAlH_4$ (1.0 M in THF, 2.4 mL, 2.4 mmol, 3.0 equiv.). After stirring at 0 ºC for 1 hour, TLC showed the consumption of the starting material. The reaction was quenched by addition of saturated aqueous potassium sodium tartrate (8 mL), and the mixture was stirred at 25 ºC until a clear solution was obtained. The product was extracted with EtOAc (10 mL × 3), the combined organic extracts were washed with saturated brine (10 mL), dried with anhydrous Na_2SO_4, filtered and concentrated under reduced pressure. The residue was purified by flash-column

chromatography on silica gel to afford **S4**.

Step c: The above product **S4** (0.56 mmol, 1.0 equiv.) was dissolved in anhydrous THF (5.6 mL, 0.1 M/L) stirred at 0 ºC, then the NaH (60% dispersion in mineral oil, 27 mg, 0.67 mmol, 1.2 equiv.) was added portion-wise to the solution. (Note: Et_3N (2.0 equiv.) was added to the solution and stirring for 20 minutes, followed by the addition of PivCl (1.1 equiv.)). After 20 minutes, the TIPSCl (120 mg, 0.62 mmol, 1.1 equiv.) or BnCl (1.1 equiv.) was added by the syringe. The reaction mixture was stirred for 2 hours, and TLC showed the consumption of the starting material. The reaction mixture was quenched by saturated aqueous NH_4Cl (5 mL). The reaction mixture was extracted by EtOAc (15 mL × 3) and the combined organic extracts were washed with saturated brine (10 mL), dried over Na_2SO_4, filtered and concentrated under reduced pressure. The residue was purified by flash-column chromatography on silica gel to give the product **S5**.

Step d: The above product **S5** (0.4 mmol, 1.0 equiv.) was dissolved in anhydrous DCM (4 mL, 0.1 M/L) stirred at 0 ºC. Then $NaHCO_3$ (168 mg, 2.0 mmol, 5.0 equiv.) and Dess-Martin periodinane (DMP, 254 mg, 0.6 mmol, 1.5 equiv.) were added to the solution. After being stirred for 1 hour at 25 ºC, saturated aqueous $NaHCO_3$ (5 mL) was added slowly, followed by saturated aqueous $Na_2S_2O_3$ (5 mL). The reaction mixture was extracted by DCM (10 mL × 3) and combined organic extracts were washed with saturated brine (10 mL), dried over Na_2SO_4, filtered and concentrated under reduced pressure. The residue was purified by flash-column chromatography on silica gel to afford the product **S6**.

Step e: The **S6** (0.35 mmol, 1.0 equiv.) was dissolved in dry toluene (3.5 mL, 0.1 M/L) stirred at 25 ºC, then copper(I)-thiophene-2-carboxylate (CuTc, 7 mg, 0.035 mmol, 0.1 equiv.) was added in one portion, followed by addition of tosyl azide (TsN_3, 83 mg, 0.42 mmol, 1.2 equiv.) via syringe. The solution was allowed to stir at 25 ºC for 3 hours. Then the organics were evaporated, diluted with a minimal amount of DCM, loaded directly onto a silica gel column and purified with eluent to give the desired the triazole compound.

Synthesis of compound 1a

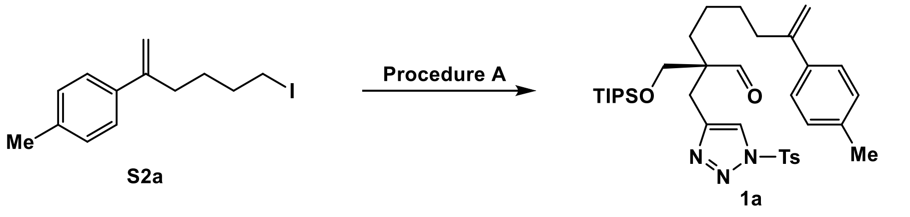

1a (190 mg): colorless oil, 30% overall yield from **S2a** (300 mg);

R*f* = 0.5 (silica gel, hexanes: EtOAc = 5: 1);

IR (film) λ_{max} 2941, 2864, 1730, 1595, 1514, 1462, 1395, 1296, 1194, 1179, 1107, 1009, 970, 883, 812, 671, 588, 542 cm^{-1};

^{1}H NMR (400 MHz, $CDCl_3$) δ 9.60 (s, 1H), 7.95 (d, *J* = 8.5 Hz, 2H), 7.88 (s, 1H), 7.35 (d, *J* = 8.0 Hz, 2H), 7.28 – 7.26 (m, 1H), 7.26 – 7.24 (m, 1H), 7.12 (d, *J* = 7.9 Hz, 2H), 5.21 (d, *J* = 1.5 Hz, 1H), 4.95 (d, *J* = 1.3 Hz, 1H), 3.80 (d, *J* = 10.2 Hz, 1H), 3.63 (d, *J* = 10.2 Hz, 1H), 3.06 – 2.95 (m, 2H), 2.43 (s, 3H), 2.34 (s, 3H), 1.65 – 1.61 (m, 1H), 1.45 – 1.32 (m, 5H), 1.26 – 1.14 (m, 1H), 1.10 – 1.05 (m, 1H), 1.05 – 0.97 (m, 21H) ppm;

^{13}C NMR (100 MHz, $CDCl_3$) δ 205.0, 148.0, 147.3, 143.8, 138.3, 137.2, 133.3, 130.5, 129.1, 128.7, 126.1, 122.5, 111.8, 65.0, 55.3, 35.0, 29.6, 28.8, 24.7, 23.1, 21.9, 21.2, 18.1, 12.0 ppm;

HRMS (ESI) calcd. for $C_{35}H_{52}N_3O_4SSi$ $[M+H]^+$: 638.3442, found: 638.3441.

Synthesis of compound 1b

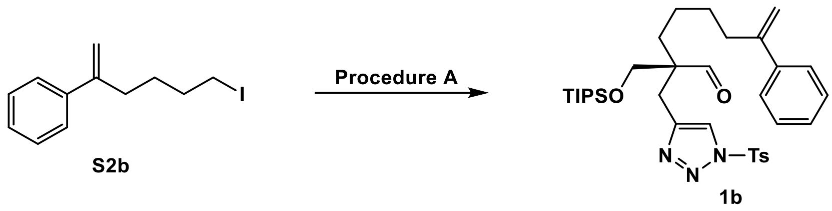

1b (250 mg): colorless oil, 32% overall yield from **S2b**[1] (360 mg);

R*f* = 0.5 (silica gel, hexanes: EtOAc = 5: 1);

IR (film) λ_{max} 2943, 2866, 2361, 1717, 1682, 1599, 1456, 1396, 1169, 1123, 1034, 1011, 883, 816, 683, 569 cm^{-1};

^{1}H NMR (400 MHz, $CDCl_3$) δ 9.52 (s, 1H), 7.87 (d, *J* = 8.4 Hz, 2H), 7.81 (s, 1H), 7.30 – 7.20 (m, 6H), 7.19 – 7.16 (m, 1H), 5.16 (d, *J* = 1.3 Hz, 1H), 4.92 (d, *J* = 1.3 Hz, 1H), 3.72 (d, *J* = 10.2 Hz, 1H), 3.56 (d, *J* = 10.2 Hz, 1H), 2.98 – 2.86 (m, 2H), 2.37 (d, *J* = 6.9 Hz, 2H), 2.34 (s, 3H), 1.37 – 1.24 (m, 5H), 1.19 – 1.09 (m, 1H), 0.99 – 0.90 (m, 21H) ppm;

^{13}C NMR (100 MHz, $CDCl_3$) δ 205.0, 148.2, 147.3, 143.7, 141.2, 133.3, 130.5, 128.7, 128.4, 127.4, 126.1, 122.5, 112.5, 65.0, 55.3, 35.0, 29.5, 28.7, 24.6, 23.1, 21.9, 18.0, 11.9 ppm;

HRMS (ESI) calcd. for $C_{34}H_{50}N_3O_4SSi$ $[M+H]^+$: 624.3286, found: 624.3288.

Synthesis of compound 1c

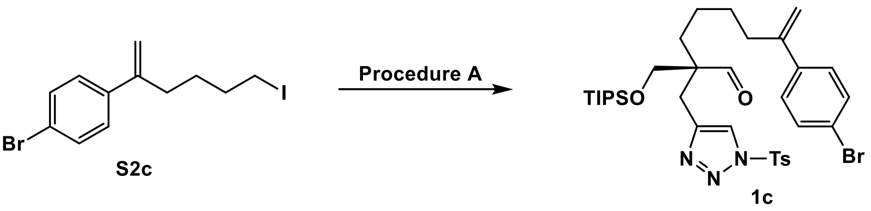

1c (280 mg): colorless oil, 15% overall yield from **S2c** (970 mg);

R*f* = 0.5 (silica gel, hexanes: EtOAc = 5: 1);

IR (film) λ_{max} 3662, 3095, 2888, 2827, 1778, 1575, 1488, 1393, 1297, 1254, 1178, 1104, 1010, 970, 882, 808, 673 cm^{-1};

^{1}H NMR (500 MHz, $CDCl_3$) δ 9.60 (s, 1H), 7.96 (d, *J* = 8.1 Hz, 2H), 7.88 (s, 1H), 7.45 – 7.34 (m, 4H), 7.22 (d, *J* = 8.2 Hz, 2H), 5.22 (s, 1H), 5.01 (s, 1H), 3.80 (d, *J* = 10.2 Hz, 1H), 3.61 (d, *J* = 10.2 Hz, 1H), 3.04 – 2.96 (m, 2H), 2.44 (s, 3H), 2.40 (d, *J* = 6.6 Hz, 1H), 1.44 – 1.33 (m, 5H), 1.27 – 1.21 (m, 2H), 1.06 – 0.96 (m, 21H) ppm;

^{13}C NMR (125 MHz, $CDCl_3$) δ 205.2, 147.3, 147.2, 143.7, 140.1, 133.3, 131.5, 130.5, 128.8, 127.9, 122.5, 121.4, 113.2, 65.0, 55.3, 34.9, 29.5, 28.6, 24.6, 23.1, 22.0, 18.1, 12.0 ppm;

HRMS (ESI) calcd. for $C_{34}H_{49}BrN_3O_4SSi$ $[M+H]^+$: 702.2391, found: 702.2396.

Synthesis of compound 1d

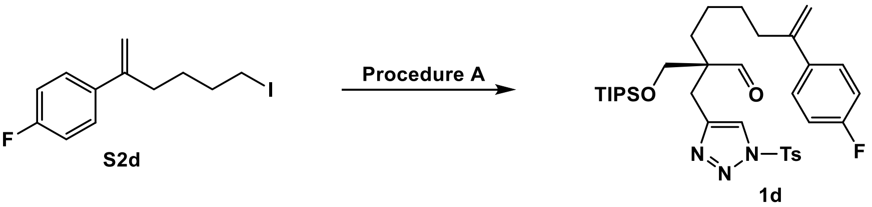

1d (256 mg): colorless oil, 18% overall yield from **S2d** (676 mg);

R*f* = 0.5 (silica gel, hexanes: EtOAc = 5: 1);

IR (film) λ_{max} 2920, 2860, 2325, 1725, 1600, 1574, 1410, 1356, 1125, 1065, 1045, 886, 542 cm^{-1};

^{1}H NMR (400 MHz, $CDCl_3$) δ 9.60 (s, 1H), 7.96 (d, *J* = 8.4 Hz, 2H), 7.88 (s, 1H), 7.38 – 7.28 (m, 4H), 7.03 – 6.95 (m, 2H), 5.18 (d, *J* = 1.1 Hz, 1H), 4.98 (d, *J* = 1.1 Hz, 1H), 3.80 (d, *J* = 10.2 Hz, 1H), 3.62 (d, *J* = 10.3 Hz, 1H), 3.06 – 2.94 (m, 2H), 2.45 – 2.38 (m, 5H), 1.45 – 1.31 (m, 5H), 1.25 – 1.15 (m, 1H), 1.11 – 0.95 (m, 21H) ppm;

^{13}C NMR (100 MHz, $CDCl_3$) δ 205.0, 163.6 (d, *J* = 245.8 Hz), 147.3 (d, *J* = 8.1 Hz), 143.7, 137.1 (d, *J* = 3.3 Hz), 133.3, 130.5, 128.7, 127.8 (d, *J* = 7.9 Hz), 122.5, 115.3, 115.1, 112.5, 65.0, 55.3, 35.2, 29.5, 28.6, 24.6, 23.1, 22.0, 18.1, 12.0 ppm;

HRMS (ESI) calcd. for $C_{34}H_{49}FN_3O_4SSi$ $[M+H]^+$: 642.3192, found: 642.3193.

Synthesis of compound 1e

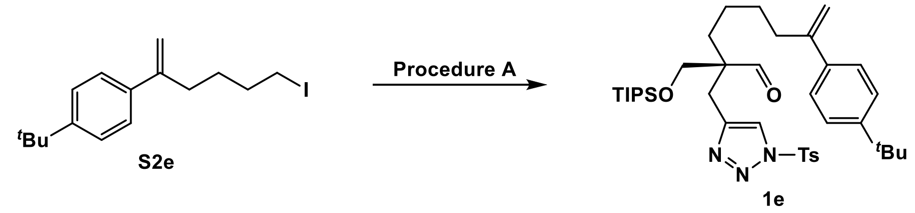

1e (249 mg): colorless oil, 18% overall yield from **S2e** (697 mg);

R*f* = 0.5 (silica gel, hexanes: EtOAc = 4: 1);

IR (film) λ_{max} 3418, 2943, 2866, 2357, 1730, 1634, 1462, 1395, 1296, 1194, 1105, 1009, 970, 883, 799, 733, 638, 586, 542 cm^{-1};

^{1}H NMR (500 MHz, $CDCl_3$) δ 9.61 (s, 1H), 7.95 (d, *J* = 8.5 Hz, 2H), 7.89 (s, 1H), 7.39 – 7.29 (m, 6H), 5.24 (d, *J* = 1.5 Hz, 1H), 4.96 (d, *J* = 1.4 Hz, 1H), 3.80 (d, *J* = 10.2 Hz, 1H), 3.64 (d, *J* = 10.3 Hz, 1H), 3.06 – 2.96 (m, 2H), 2.46 – 2.40 (m, 5H), 1.46 – 1.35 (m, 5H), 1.32 (s, 9H), 1.25 – 1.18 (m, 1H), 1.08 – 1.03 (m, 3H), 1.04 – 0.97 (m, 18H) ppm;

^{13}C NMR (125 MHz, $CDCl_3$) δ 205.0, 150.4, 147.9, 147.3, 143.8, 138.1, 133.3, 130.5, 128.7, 125.8, 125.3, 122.5, 111.8, 65.0, 55.3, 35.0, 34.6, 31.5, 29.6, 28.9, 24.7, 23.2, 22.0, 18.1, 12.0 ppm;

HRMS (ESI) calcd. for $C_{38}H_{58}N_3O_4SSi$ $[M+H]^+$: 680.3912, found: 680.3919.

Synthesis of compound 1f

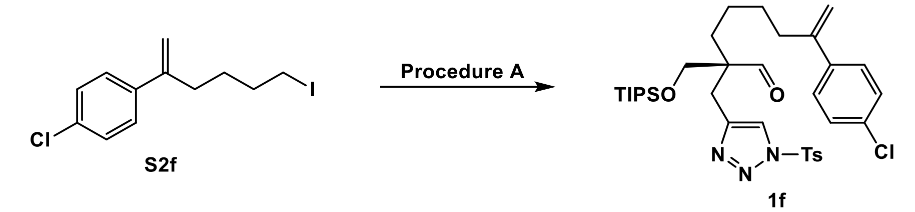

1f (241 mg): colorless oil, 25% overall yield from **S2f** (470 mg);

R*f* = 0.5 (silica gel, hexanes: EtOAc = 4: 1);

IR (film) λ_{max} 3445, 2926, 2864, 2342, 1651, 1458, 1335, 1165, 1125, 1098, 1069, 1011, 881, 814, 667, 554 cm^{-1};

^{1}H NMR (500 MHz, $CDCl_3$) δ 9.60 (s, 1H), 7.96 (d, *J* = 8.5 Hz, 2H), 7.88 (s, 1H), 7.36 (d, *J* = 8.2 Hz, 2H), 7.28 (s, 4H), 5.22 (d, *J* = 1.2 Hz, 1H), 5.01 (d, *J* = 1.4 Hz, 1H), 3.80 (d, *J* = 10.3 Hz, 1H), 3.61 (d, *J* = 10.2 Hz, 1H), 3.08 – 2.94 (m, 2H), 2.45 – 2.39 (m, 5H), 1.45 – 1.31 (m, 5H), 1.25 – 1.16 (m, 1H), 1.05 – 0.98 (m, 21H) ppm;

^{13}C NMR (125 MHz, $CDCl_3$) δ 205.0, 147.3, 147.1, 143.7, 139.6, 133.3, 133.2, 130.5, 128.7, 128.5, 127.5, 122.5, 113.1, 65.0, 55.3, 35.0, 29.5, 28.6, 24.6, 23.1, 22.0, 18.1, 12.0 ppm;

HRMS (ESI) calcd. for $C_{34}H_{49}ClN_3O_4SSi$ $[M+H]^+$: 658.2896, found: 658.2898.

Synthesis of compound 1g

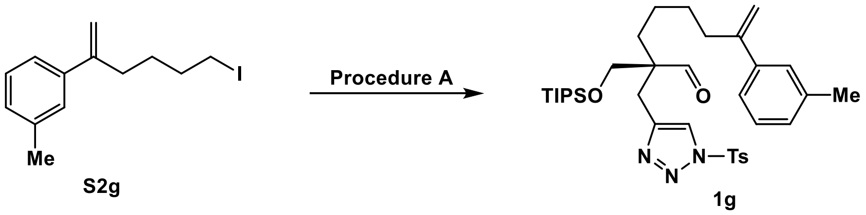

1g (249 mg): colorless oil, 18% overall yield from **S2g** (650 mg);

$\mathbf{R}_f$ = 0.5 (silica gel, hexanes: EtOAc = 4: 1);

IR (film) λ_{max} 2955, 2880, 1738, 1600, 1502, 1395, 1296, 1184, 1126, 1010, 890, 812, 671, 590, 560 cm^{-1};

^{1}H NMR (500 MHz, $CDCl_3$) δ 9.61 (s, 1H), 7.99 – 7.93 (m, 2H), 7.89 (s, 1H), 7.35 (d, *J* = 8.1 Hz, 2H), 7.23 – 7.13 (m, 3H), 7.08 (d, *J* = 7.3 Hz, 1H), 5.22 (d, *J* = 1.5 Hz, 1H), 4.98 (d, *J* = 1.4 Hz, 1H), 3.80 (d, *J* = 10.2 Hz, 1H), 3.63 (d, *J* = 10.2 Hz, 1H), 3.08 – 2.95 (m, 2H), 2.47 – 2.40 (m, 5H), 2.35 (s, 3H), 1.46 – 1.33 (m, 5H), 1.26 – 1.18 (m, 1H), 1.06 – 0.98 (m, 21H) ppm;

^{13}C NMR (125 MHz, $CDCl_3$) δ 205.0, 148.4, 147.3, 143.8, 141.2, 137.9, 133.3, 130.5, 128.7, 128.3, 128.2, 126.9, 123.3, 122.5, 112.3, 65.0, 55.3, 35.1, 29.6, 28.7, 24.7, 23.1, 21.9, 21.6, 18.1, 11.9 ppm;

HRMS (ESI) calcd. for $C_{35}H_{52}N_3O_4SSi$ $[M+H]^+$: 638.3442, found: 638.3441.

Synthesis of compound 1h

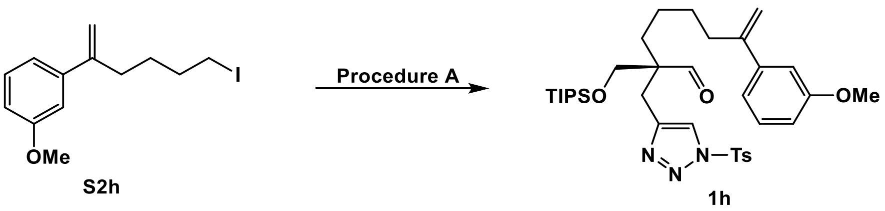

1h (196 mg): colorless oil, 31% overall yield from **S2h** (306 mg);

$\mathbf{R}_f$ = 0.4 (silica gel, hexanes: EtOAc = 5: 1);

IR (film) λ_{max} 2941, 2864, 2357, 1730, 1595, 1462, 1395, 1287, 1193, 1179, 1094, 1109, 881, 812, 790, 671, 586, 542 cm^{-1};

^{1}H NMR (400 MHz, $CDCl_3$) δ 9.60 (s, 1H), 7.95 (d, *J* = 8.4 Hz, 2H), 7.88 (s, 1H), 7.35 (d, *J* = 8.1 Hz, 2H), 7.23 (t, *J* = 7.9 Hz, 1H), 6.98 – 6.92 (m, 1H), 6.91 – 6.88 (m, 1H), 6.84 – 6.77 (m, 1H), 5.24 (d, *J* = 1.3 Hz, 1H), 4.99 (d, *J* = 1.2 Hz, 1H), 3.83 – 3.77 (m, 4H), 3.62 (d, *J* = 10.2 Hz, 1H), 3.06 – 2.95 (m, 2H), 2.47 – 2.37 (m, 5H), 1.45 – 1.32 (m, 5H), 1.27 – 1.13 (m, 1H), 1.09 – 0.97 (m, 21H)

ppm;

^{13}C NMR (100 MHz, $CDCl_3$) δ 205.0, 159.7, 148.2, 147.3, 143.8, 142.9, 133.3, 130.5, 129.4, 128.7, 122.5, 118.8, 112.7, 112.1, 65.0, 55.4, 55.3, 35.1, 29.6, 28.7, 24.7, 23.1, 22.0, 18.1, 12.0 ppm;

HRMS (ESI) calcd. for $C_{35}H_{52}N_3O_5SSi$ $[M+H]^+$: 654.3391, found: 654.3390.

Synthesis of compound 1i

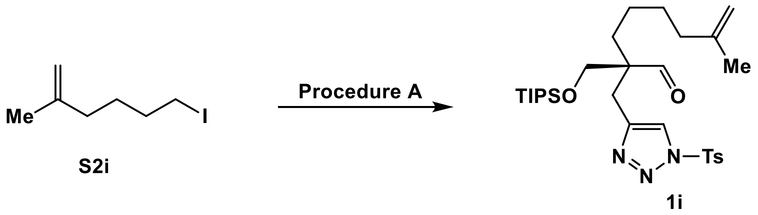

1i (201 mg): colorless oil, 26% overall yield from **S2i**[2] (310 mg);

R$_f$ = 0.5 (silica gel, hexanes: EtOAc = 5: 1);

IR (film) λ_{max} 2941, 2866, 1730, 1649, 1595, 1462, 1395, 1296, 1196, 1179, 1107, 1094, 1009, 970, 883, 812, 671, 588, 542 cm^{-1};

^{1}H NMR (500 MHz, $CDCl_3$) δ 9.62 (s, 1H), 7.96 (d, *J* = 8.2 Hz, 2H), 7.89 (s, 1H), 7.36 (d, *J* = 8.2 Hz, 2H), 4.66 (s, 1H), 4.60 (s, 1H), 3.81 (d, *J* = 10.2 Hz, 1H), 3.64 (d, *J* = 10.2 Hz, 1H), 3.08 – 2.94 (m, 2H), 2.44 (s, 3H), 1.94 (t, *J* = 7.1 Hz, 2H), 1.66 (s, 3H), 1.47 – 1.40 (m, 2H), 1.38 – 1.30 (m, 3H), 1.22 – 1.11 (m, 1H), 1.08 – 0.96 (m, 21H) ppm;

^{13}C NMR (125 MHz, $CDCl_3$) δ 205.0, 147.3, 145.7, 143.8, 133.3, 130.5, 128.7, 122.5, 110.1, 65.0, 55.3, 37.5, 29.7, 28.1, 24.6, 23.1, 22.4, 21.9, 18.1, 11.9 ppm;

HRMS (ESI) calcd. for $C_{29}H_{48}N_3O_4SSi$ $[M+H]^+$: 562.3129, found: 562.3128.

Synthesis of compound 1j

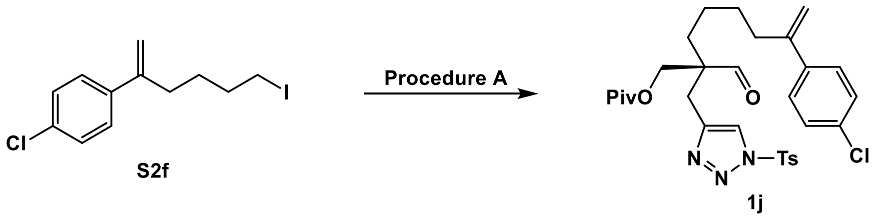

1j (144 mg): colorless oil, 23% overall yield from **S2f** (342 mg);

R$_f$ = 0.4 (silica gel, hexanes: EtOAc = 5: 1);

IR (film) λ_{max} 2926, 2854, 1739, 1396, 1259, 1178, 1093, 1010, 813, 671, 586, 542 cm^{-1};

^{1}H NMR (500 MHz, $CDCl_3$) δ 9.54 (s, 1H), 8.01 – 7.93 (m, 2H), 7.88 (s, 1H), 7.37 (d, *J* = 8.2 Hz, 2H), 7.28 (s, 4H), 5.22 (d, *J* = 1.3 Hz, 1H), 5.01 (d, *J* = 1.4 Hz, 1H), 4.21 (d, *J* = 11.7 Hz, 1H), 3.96

(d, J = 11.7 Hz, 1H), 3.04 – 2.92 (m, 2H), 2.48 – 2.31 (m, 5H), 1.49 – 1.43 (m, 2H), 1.41 – 1.33 (m, 3H), 1.27 – 1.20 (m, 1H), 1.15 (s, 9H) ppm;

^{13}C NMR (125 MHz, $CDCl_3$) δ 202.6, 177.9, 147.5, 147.0, 142.7, 139.5, 133.3, 133.0, 130.6, 128.8, 128.6, 127.5, 122.5, 113.2, 64.2, 53.4, 39.0, 34.9, 30.1, 28.4, 27.2, 25.3, 22.9, 22.0 ppm;

HRMS (ESI) calcd. for $C_{30}H_{37}ClN_3O_5S$ $[M+H]^+$: 586.2137, found: 586.2142.

Synthesis of compound 1k

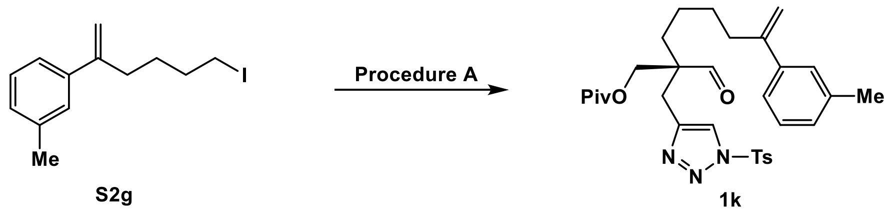

1k (155 mg): colorless oil, 29% overall yield from **S2g** (284 mg);

R*f* = 0.4 (silica gel, hexanes: EtOAc = 5: 1);

IR (film) λ_{max} 2934, 2868, 2359, 1732, 1595, 1464, 1395, 1281, 1196, 1011, 972, 816, 669, 586 cm^{-1};

^{1}H NMR (500 MHz, $CDCl_3$) δ 9.54 (s, 1H), 7.99 – 7.92 (m, 2H), 7.87 (s, 1H), 7.37 (d, J = 8.1 Hz, 2H), 7.21 (t, J = 7.5 Hz, 1H), 7.19 – 7.12 (m, 2H), 7.08 (d, J = 7.4 Hz, 1H), 5.22 (d, J = 1.5 Hz, 1H), 4.98 (d, J = 1.5 Hz, 1H), 4.21 (d, J = 11.7 Hz, 1H), 3.97 (d, J = 11.7 Hz, 1H), 2.99 (s, 2H), 2.48 – 2.41 (m, 5H), 2.35 (s, 3H), 1.51 – 1.43 (m, 2H), 1.42 – 1.34 (m, 3H), 1.27 – 1.20 (m, 1H), 1.15 (s, 9H) ppm;

^{13}C NMR (125 MHz, $CDCl_3$) δ 202.7, 177.9, 148.3, 147.5, 142.8, 141.1, 138.0, 133.1, 130.6, 128.8, 128.33, 128.29, 127.0, 123.3, 122.5, 112.5, 64.2, 53.4, 39.0, 35.1, 30.2, 28.5, 27.2, 25.4, 22.9, 22.0, 21.6 ppm;

HRMS (ESI) calcd. for $C_{31}H_{40}N_3O_5S$ $[M+H]^+$: 566.2683, found: 566.2689.

Synthesis of compound 1l

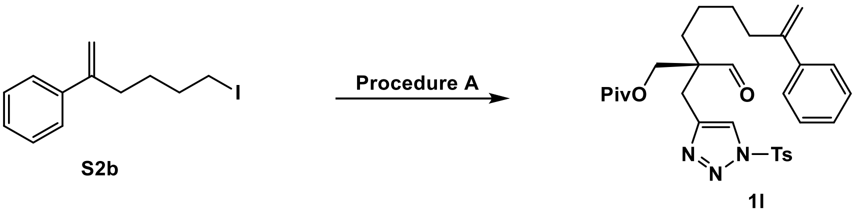

1l (200 mg): colorless oil, 20% overall yield from **S2b** (520 mg);

R*f* = 0.5 (silica gel, hexanes: EtOAc = 5: 1);

IR (film) λ_{max} 3460, 2359, 2081, 1645, 1395, 1281, 1194, 1150, 1009, 968, 702, 669, 586, 542 cm^{-1};

^{1}**H NMR** (500 MHz, $CDCl_3$) δ 9.54 (s, 1H), 7.96 (d, *J* = 8.5 Hz, 2H), 7.87 (s, 1H), 7.38 – 7.29 (m, 6H), 7.27 (d, *J* = 1.6 Hz, 1H), 5.24 (d, *J* = 1.5 Hz, 1H), 5.00 (d, *J* = 1.4 Hz, 1H), 4.20 (d, *J* = 11.7 Hz, 1H), 3.97 (d, *J* = 11.7 Hz, 1H), 2.98 (s, 2H), 2.51 – 2.39 (m, 5H), 1.48 – 1.42 (m, 2H), 1.40 – 1.36 (m, 2H), 1.27 – 1.23 (m, 2H), 1.15 (s, 9H) ppm;

^{13}**C NMR** (125 MHz, $CDCl_3$) δ 202.7, 178.0, 148.1, 147.5, 142.8, 141.1, 133.1, 130.6, 128.8, 128.5, 127.5, 126.2, 122.5, 112.7, 64.2, 53.4, 39.0, 35.0, 30.2, 28.5, 27.2, 25.4, 22.9, 22.0 ppm;

HRMS (ESI) calcd. for $C_{30}H_{38}N_3O_5S$ $[M+H]^+$: 552.2527, found: 552.2535.

Synthesis of compound 1m

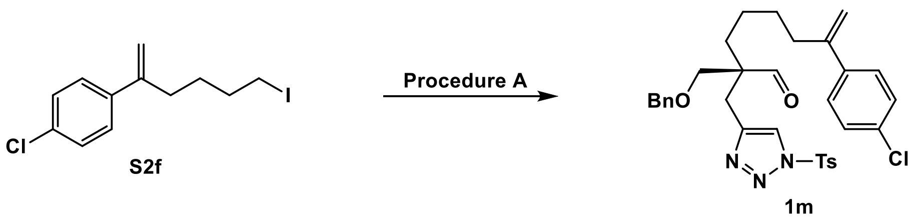

1m (224 mg): colorless oil, 21% overall yield from **S2f** (578 mg);

$\mathbf{R}_f$ = 0.6 (silica gel, hexanes: EtOAc = 4: 1);

IR (film) λ_{max} 2940, 2880, 2377, 1720, 1390, 1100, 1011, 814, 741, 665, 570, 532 cm^{-1};

^{1}**H NMR** (500 MHz, $CDCl_3$) δ 9.54 (s, 1H), 7.96 – 7.89 (m, 2H), 7.72 (s, 1H), 7.37 – 7.29 (m, 5H), 7.26 (s, 5H), 7.25 – 7.24 (m, 1H), 5.20 (d, *J* = 1.2 Hz, 1H), 4.99 (d, *J* = 1.4 Hz, 1H), 4.41 (s, 2H), 3.46 (d, *J* = 9.6 Hz, 1H), 3.29 (d, *J* = 9.6 Hz, 1H), 3.07 – 2.92 (m, 2H), 2.42 (s, 3H), 2.39 (t, *J* = 7.0 Hz, 2H), 1.43 – 1.28 (m, 5H), 1.19 – 1.09 (m, 1H) ppm;

^{13}**C NMR** (125 MHz, $CDCl_3$) δ 204.2, 147.3, 147.1, 143.5, 139.6, 137.7, 133.20, 133.18, 130.5, 128.7, 128.6, 128.5, 128.1, 127.9, 127.5, 122.5, 113.1, 73.5, 70.6, 54.0, 34.9, 30.1, 28.4, 25.1, 22.9, 22.0 ppm;

HRMS (ESI) calcd. for $C_{32}H_{35}ClN_3O_4S$ $[M+H]^+$: 592.2031, found: 592.2029.

Synthesis of compound 1n

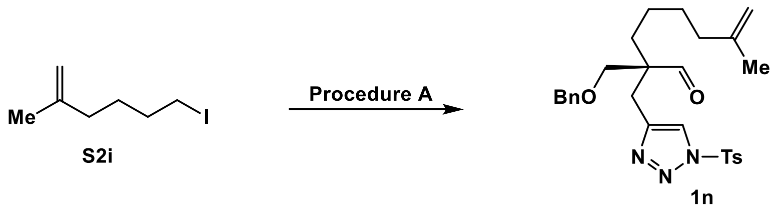

1n (149 mg): colorless oil, 26% overall yield from **S2i** (260 mg);

$\mathbf{R}_f$ = 0.5 (silica gel, hexanes: EtOAc = 5: 1);

IR (film) λ_{max} 2934, 2860, 2344, 1730, 1647, 1595, 1454, 1395, 1196, 1179, 1094, 1009, 887, 816, 739, 671, 586, 542 cm^{-1};

^{1}H NMR (400 MHz, $CDCl_3$) δ 9.57 (s, 1H), 7.94 (d, *J* = 8.4 Hz, 2H), 7.75 (s, 1H), 7.39 – 7.29 (m, 5H), 7.29 – 7.25 (m, 2H), 4.67 (s, 1H), 4.60 (s, 1H), 4.43 (s, 2H), 3.50 (d, *J* = 9.6 Hz, 1H), 3.33 (d, *J* = 9.6 Hz, 1H), 3.11 – 2.91 (m, 2H), 2.44 (s, 3H), 1.93 (t, *J* = 7.1 Hz, 2H), 1.66 (s, 3H), 1.48 – 1.39 (m, 2H), 1.39 – 1.30 (m, 3H), 1.19 – 1.07 (m, 1H) ppm;

^{13}C NMR (100 MHz, $CDCl_3$) δ 204.3, 147.3, 145.7, 143.6, 137.8, 133.3, 130.5, 128.7, 128.6, 128.1, 127.9, 122.6, 110.1, 73.5, 70.7, 54.0, 37.4, 30.3, 28.0, 25.2, 23.0, 22.4, 22.0 ppm;

HRMS (ESI) calcd. for $C_{27}H_{34}N_3O_4S$ $[M+H]^+$: 496.2265, found: 496.2265.

Synthesis of compound 1p

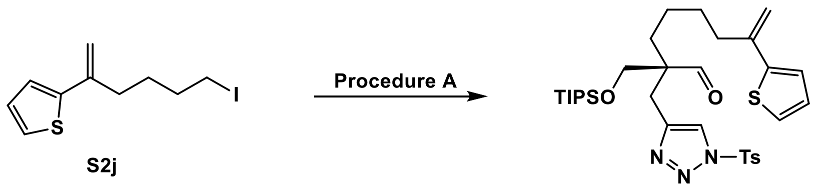

S2j

1p

1p (307 mg): colorless oil, 32% overall yield from **S2j** (438 mg);

R*f* = 0.4 (silica gel, hexanes: EtOAc = 5: 1);

IR (film) λ_{max} 3391, 2941, 2866, 2359, 1717, 1599, 1464, 1396, 1175, 1123, 1034, 1011, 883, 816, 683, 569 cm^{-1};

^{1}H NMR (500 MHz, $CDCl_3$) δ 9.62 (s, 1H), 7.96 (d, *J* = 8.4 Hz, 2H), 7.89 (s, 1H), 7.36 (d, *J* = 8.1 Hz, 2H), 7.15 (dd, *J* = 5.0, 1.0 Hz, 1H), 7.02 – 6.93 (m, 2H), 5.35 (s, 1H), 4.89 (s, 1H), 3.81 (d, *J* = 10.2 Hz, 1H), 3.65 (d, *J* = 10.2 Hz, 1H), 3.09 – 2.95 (m, 2H), 2.43 (s, 3H), 2.42 – 2.38 (m, 2H), 1.51 – 1.37 (m, 5H), 1.31 – 1.23 (m, 1H), 1.07 – 0.97 (m, 21H) ppm;

^{13}C NMR (125 MHz, $CDCl_3$) δ 205.0, 147.3, 145.3, 143.8, 141.5, 133.3, 130.5, 128.7, 127.5, 124.2, 123.4, 122.5, 111.1, 65.0, 55.3, 35.3, 29.6, 29.0, 24.7, 23.2, 22.0, 18.1, 12.0 ppm;

HRMS (ESI) calcd. for $C_{32}H_{48}N_3O_4S_2Si$ $[M+H]^+$: 630.2850, found: 630.2849.

Synthesis of compound 1q

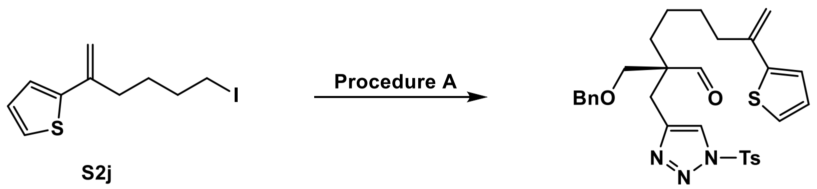

S2j

1q

1q (226 mg): colorless oil, 30% overall yield from **S2j** (390 mg);

R*f* = 0.3 (silica gel, hexanes: EtOAc = 5: 1);

IR (film) λ_{max} 2928, 2860, 2361, 1719, 1389, 1193, 1179, 1092, 1011, 814, 700, 671, 586, 542 cm^{-1};

^{1}H NMR (500 MHz, $CDCl_3$) δ 9.57 (s, 1H), 7.93 (d, *J* = 8.4 Hz, 2H), 7.74 (s, 1H), 7.40 – 7.29 (m, 5H), 7.27 (d, *J* = 1.5 Hz, 2H), 7.15 (dd, *J* = 5.0, 1.1 Hz, 1H), 7.05 – 6.91 (m, 2H), 5.35 (s, 1H), 4.88 (s, 1H), 4.43 (s, 2H), 3.49 (d, *J* = 9.6 Hz, 1H), 3.33 (d, *J* = 9.6 Hz, 1H), 3.08 – 2.97 (m, 2H), 2.43 (s, 3H), 2.41 – 2.36 (m, 2H), 1.53 – 1.31 (m, 5H), 1.24 – 1.16 (m, 1H) ppm;

^{13}C NMR (125 MHz, $CDCl_3$) δ 204.3, 147.3, 145.3, 143.5, 141.5, 137.8, 133.3, 130.5, 128.7, 128.6, 128.1, 127.9, 127.5, 124.2, 123.4, 122.6, 111.1, 73.6, 70.6, 54.0, 35.3, 30.2, 28.9, 25.2, 23.1, 22.0 ppm;

HRMS (ESI) calcd. for $C_{30}H_{34}N_3O_4S_2$ $[M+H]^+$: 564.1985, found: 564.1992.

Synthesis of compound 1r

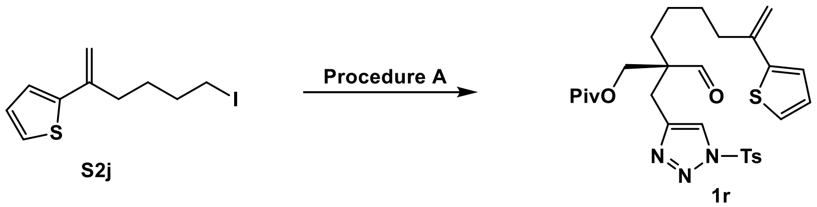

1r (166 mg): colorless oil, 29% overall yield from **S2j** (300 mg);

R*f* = 0.3 (silica gel, hexanes: EtOAc = 5: 1);

IR (film) λ_{max} 2934, 2359, 1730, 1159, 1123, 1034, 1009, 816, 681, 669, 567 cm^{-1};

^{1}H NMR (500 MHz, $CDCl_3$) δ 9.56 (s, 1H), 7.96 (d, *J* = 8.4 Hz, 2H), 7.88 (s, 1H), 7.37 (d, *J* = 8.2 Hz, 2H), 7.16 (dd, *J* = 5.0, 0.9 Hz, 1H), 7.03 – 6.94 (m, 2H), 5.36 (s, 1H), 4.89 (s, 1H), 4.22 (d, *J* = 11.7 Hz, 1H), 3.99 (d, *J* = 11.7 Hz, 1H), 3.05 – 2.94 (m, 2H), 2.45 – 2.39 (m, 5H), 1.54 – 1.38 (m, 5H), 1.31 – 1.23 (m, 1H), 1.16 (s, 9H) ppm;

^{13}C NMR (125 MHz, $CDCl_3$) δ 202.7, 178.0, 147.5, 145.2, 142.8, 141.3, 133.1, 130.6, 128.8, 127.5, 124.3, 123.5, 122.5, 111.2, 64.2, 53.4, 39.0, 35.3, 30.2, 28.8, 27.2, 25.4, 23.1, 22.0 ppm;

HRMS (ESI) calcd. for $C_{28}H_{36}N_3O_5S_2$ $[M+H]^+$: 558.2091, found: 558.2097.

Synthesis of compound 1ac

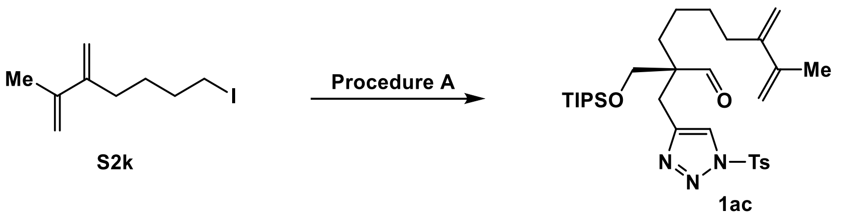

1ac (338 mg): colorless oil, 6% overall yield from **S2k**[3] (2.40 g);

R*f* = 0.5 (silica gel, hexanes: EtOAc = 5: 1);

IR (film) λ_{max} 2928, 2357, 1722, 1595, 1456, 1395, 1339, 1194, 1179, 1159, 1070, 1011, 972, 814, 741, 671, 586, 561, 542 cm^{-1};

^{1}H NMR (500 MHz, $CDCl_3$) δ 9.62 (s, 1H), 7.96 (d, *J* = 8.5 Hz, 2H), 7.89 (s, 1H), 7.36 (d, *J* = 8.2 Hz, 2H), 5.03 (d, *J* = 19.7 Hz, 2H), 4.91 (d, *J* = 30.0 Hz, 2H), 3.81 (d, *J* = 10.2 Hz, 1H), 3.64 (d, *J* = 10.3 Hz, 1H), 3.07 – 2.96 (m, 2H), 2.44 (s, 3H), 2.20 (t, *J* = 7.2 Hz, 2H), 1.88 (s, 3H), 1.48 – 1.33 (m, 5H), 1.25 – 1.16 (m, 1H), 1.08 – 0.98 (m, 21H) ppm;

^{13}C NMR (125 MHz, $CDCl_3$) δ 205.1, 147.7, 147.3, 143.8, 142.6, 133.3, 130.5, 128.7, 122.5, 112.7, 112.3, 65.0, 55.3, 33.4, 29.7, 29.4, 24.7, 23.4, 22.0, 21.3, 18.1, 12.0 ppm;

HRMS (ESI) calcd. for $C_{31}H_{50}N_3O_4SSi$ $[M+H]^+$: 588.3286, found: 588.3282.

Note: Compound **1ac** was a common synthetic precursor for the synthesis of **3ac** and **3ad**.

Synthesis of compound 1ae

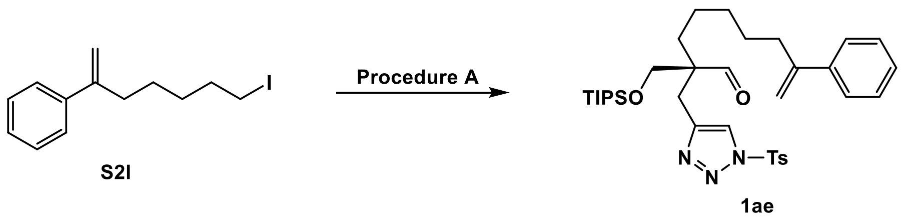

1ae (128 mg): colorless oil, 25% overall yield from **S2l** (240 mg);

R*f* = 0.5 (silica gel, hexanes: EtOAc = 5: 1);

IR (film) λ_{max} 2940, 2864, 2359, 1732, 1456, 1395, 1260, 1194, 1179, 1105, 1011, 970, 881, 812, 669, 586, 542 cm^{-1};

^{1}H NMR (500 MHz, $CDCl_3$) δ 9.62 (s, 1H), 7.99 (d, *J* = 8.4 Hz, 2H), 7.91 (s, 1H), 7.43 – 7.37 (m, 4H), 7.37 – 7.31 (m, 2H), 7.29 – 7.26 (m, 1H), 5.27 (d, *J* = 1.3 Hz, 1H), 5.04 (d, *J* = 1.4 Hz, 1H), 3.82 (d, *J* = 10.3 Hz, 1H), 3.63 (d, *J* = 10.3 Hz, 1H), 3.09 – 2.95 (m, 2H), 2.52 – 2.43 (m, 5H), 1.47 – 1.34 (m, 5H), 1.30 – 1.18 (m, 3H), 1.11 – 0.99 (m, 21H) ppm;

^{13}C NMR (125 MHz, $CDCl_3$) δ 205.0, 148.5, 147.3, 143.8, 141.4, 133.3, 130.5, 128.7, 128.4, 127.4,

126.2, 122.5, 112.4, 64.9, 55.4, 35.3, 29.83, 29.78, 28.0, 24.6, 23.3, 22.0, 18.1, 12.0 ppm;
HRMS (ESI) calcd. for $C_{35}H_{52}N_3O_4SSi$ $[M+H]^+$: 638.3442, found: 638.3447.

Synthesis of compound 1af

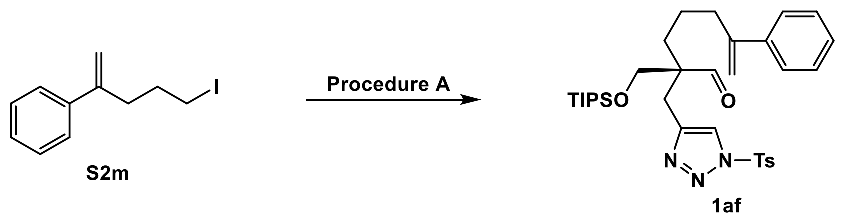

1af (134 mg): colorless oil, 14% overall yield from **S2m**[4] (430 mg);
R*f* = 0.4 (silica gel, hexanes: EtOAc = 5: 1);
IR (film) λ_{max} 2943, 2866, 1730, 1595, 1462, 1395, 1194, 1179, 1107, 1009, 970, 883, 812, 779, 671, 586, 542 cm^{-1};
^{1}H NMR (500 MHz, $CDCl_3$) δ 9.56 (s, 1H), 7.96 (d, *J* = 8.4 Hz, 2H), 7.85 (s, 1H), 7.38 – 7.29 (m, 6H), 7.27 – 7.24 (m, 1H), 5.24 (d, *J* = 1.1 Hz, 1H), 5.00 (d, *J* = 1.5 Hz, 1H), 3.76 (d, *J* = 10.2 Hz, 1H), 3.61 (d, *J* = 10.2 Hz, 1H), 3.04 – 2.90 (m, 2H), 2.47 – 2.38 (m, 5H), 1.56 – 1.44 (m, 3H), 1.40 – 1.32 (m, 1H), 1.05 – 0.96 (m, 21H) ppm;
^{13}C NMR (125 MHz, $CDCl_3$) δ 204.9, 147.7, 147.3, 143.7, 140.9, 133.3, 130.5 128.7, 128.5, 127.6, 126.1, 122.5, 112.9, 65.0, 55.1, 35.7, 29.4, 24.7, 22.1, 21.9, 18.1, 11.9 ppm;
HRMS (ESI) calcd. for $C_{33}H_{48}N_3O_4SSi$ $[M+H]^+$: 610.3129, found: 610.3135.

Synthesis of compound 1ag

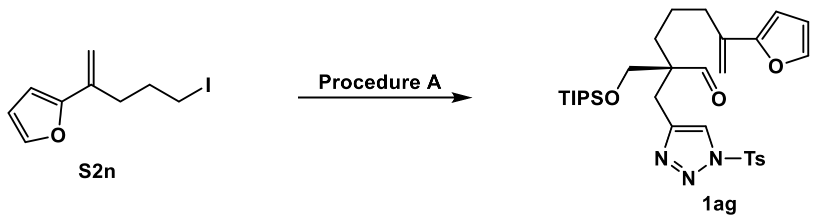

1ag (172 mg): colorless oil, 29% overall yield from **S2n** (260 mg);
R*f* = 0.5 (silica gel, hexanes: EtOAc = 5: 1);
IR (film) λ_{max} 2940, 2864, 2359, 2330, 1732, 1717, 1519, 1456, 1396, 1196, 1180, 1093, 1011, 881, 816, 669, 586, 419 cm^{-1};
^{1}H NMR (500 MHz, $CDCl_3$) δ 9.61 (s, 1H), 7.97 (d, *J* = 8.4 Hz, 2H), 7.86 (s, 1H), 7.39 – 7.35 (m, 3H), 6.37 (dd, *J* = 3.3, 1.8 Hz, 1H), 6.28 (d, *J* = 3.3 Hz, 1H), 5.47 (s, 1H), 4.88 (s, 1H), 3.80 (d, *J* =

10.2 Hz, 1H), 3.64 (d, J = 10.2 Hz, 1H), 3.01 (d, J = 4.0 Hz, 2H), 2.45 (s, 3H), 2.32 – 2.25 (m, 2H), 1.51 – 1.46 (m, 2H), 1.25 (s, 2H), 1.01 – 0.95 (m, 21H) ppm;

^{13}C NMR (125 MHz, $CDCl_3$) δ 205.0, 154.4, 147.3, 143.7, 142.0, 136.8, 133.3, 130.6, 128.7, 122.6, 111.3, 109.8, 106.3, 65.1, 55.2, 33.7, 29.3, 24.6, 22.7, 22.0, 18.1, 12.0 ppm;

HRMS (ESI) calcd. for $C_{31}H_{46}N_3O_5SSi$ $[M+H]^+$: 600.2922, found: 600.2930.

Synthesis of compound 1ah

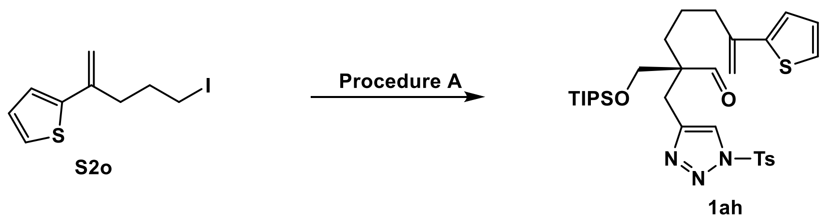

1ah (147 mg): colorless oil, 26% overall yield from **S2o** (256 mg);

R$_f$ = 0.4 (silica gel, hexanes: EtOAc = 5: 1);

IR (film) λ_{max} 2943, 2866, 1728, 1462, 1395, 1194, 1179, 1094, 1009, 970, 881, 812, 586, 542 cm^{-1};

^{1}H NMR (500 MHz, $CDCl_3$) δ 9.60 (s, 1H), 7.96 (d, J = 8.5 Hz, 2H), 7.87 (s, 1H), 7.36 (d, J = 8.1 Hz, 2H), 7.15 (dd, J = 5.0, 1.2 Hz, 1H), 7.00 – 6.93 (m, 2H), 5.35 (s, 1H), 4.88 (s, 1H), 3.80 (d, J = 10.2 Hz, 1H), 3.64 (d, J = 10.2 Hz, 1H), 3.06 – 2.93 (m, 2H), 2.43 (s, 3H), 2.42 – 2.33 (m, 2H), 1.68 – 1.59 (m, 1H), 1.56 – 1.44 (m, 3H), 1.05 – 0.97 (m, 21H) ppm;

^{13}C NMR (125 MHz, $CDCl_3$) δ 204.8, 147.3, 144.9, 143.6, 140.9, 133.3, 130.5, 128.7, 127.5, 124.3, 123.5, 122.5, 111.3, 65.0, 55.2, 35.9, 29.3, 24.6, 22.4, 21.9, 18.1, 11.9 ppm;

HRMS (ESI) calcd. for $C_{31}H_{46}N_3O_4S_2Si$ $[M+H]^+$: 616.2694, found: 616.2702.

2.2.2 Procedure and Characteristic Data for Substrate 1o

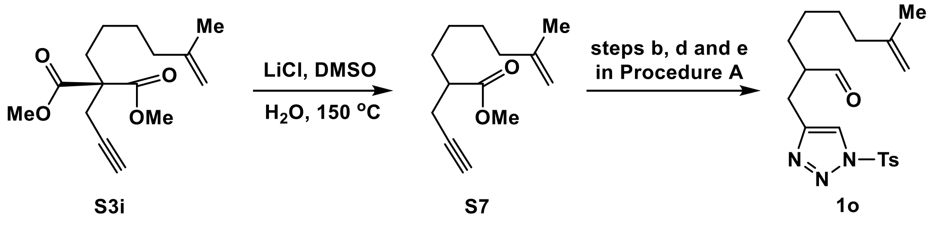

The compound **S3i** (2.58 g, 9.7 mmol, 1.0 equiv.), anhydrous LiCl (2.06 g, 48.5 mmol, 5.0 equiv.) and H_2O (0.9 mL, 48.5 mmol, 5.0 equiv.) were added in DMSO (48.5 mL, 0.2 M/L). The reaction mixture was heated at 150 ºC for 8 hours under argon. The reaction mixture was cooled to 25 ºC and H_2O (100 mL) was added. The mixture was extracted with ether (50 mL × 3), and the combined organic extracts were washed with saturated brine (50 mL) and dried over Na_2SO_4. Solvent was

removed by rotary evaporation and the residue was purified by flash-column chromatography on silica gel (hexanes: EtOAc = 20: 1) to afford the corresponding product **S7** (1.35 g, 67% yield).

Compound **S7** underwent steps b, d and e in Procedure A to give **1o**.

1o (1.05 g): colorless oil, 29% overall yield from **S3i** (2.58 g);
R*f* = 0.5 (silica gel, hexanes: EtOAc = 5: 1);
IR (film) λ_{max} 3129, 2931, 2858, 2341, 1607, 1454, 1395, 1167, 1123, 1034, 1009, 816, 683, 671, 567 cm^{-1};
^{1}H NMR (400 MHz, $CDCl_3$) δ 9.66 (s, 1H), 7.96 (d, *J* = 8.4 Hz, 2H), 7.89 (s, 1H), 7.36 (d, *J* = 8.1 Hz, 2H), 4.67 (s, 1H), 4.62 (s, 1H), 3.04 (dd, *J* = 14.9, 7.8 Hz, 1H), 2.86 – 2.74 (m, 2H), 2.43 (s, 3H), 1.97 (t, *J* = 7.2 Hz, 2H), 1.73 – 1.69 (m, 1H), 1.67 (s, 3H), 1.55 – 1.29 (m, 5H) ppm;
^{13}C NMR (100 MHz, $CDCl_3$) δ 203.5, 147.3, 145.5, 145.2, 133.1, 130.5, 128.6, 121.7, 110.1, 51.1, 37.4, 28.5, 27.4, 26.3, 24.0, 22.3, 21.9 ppm;
HRMS (ESI) calcd. for $C_{19}H_{26}N_3O_3S$ $[M+H]^+$: 376.1689, found: 376.1690.

2.2.3 Procedure B: General Procedure and Characteristic Data for Substrates 1s-1z.

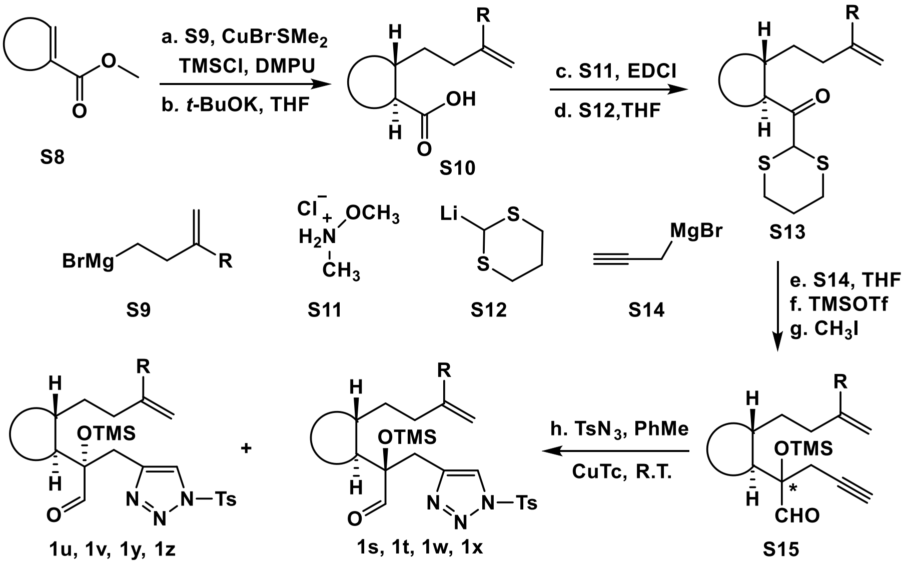

Step a: To solid Mg^0 ribbons (415 mg, 17.2 mmol, 1.6 equiv.) and a crystal of I_2 (1 mg) in THF (26 mL, 0.5 M/L) were added alkyl bromide (13.0 mmol, 1.2 equiv.). The reaction mixture was stirred at 25 ºC, and the magnesium was clearly being consumed. After stirring for 2 hours at 25 ºC, the reaction was stopped and the solution of putative Grignard reagent (**S9**) was used directly in the next step[5].

A suspension of $CuBr•SMe_2$ (222 mg, 1.08 mmol, 0.1 equiv.) in THF (22 mL, 0.5 M/L) was cooled to -78 ºC and treated sequentially with DMPU (2.6 mL, 21.6 mmol, 2.0 equiv.), TMSCl (1.0 M in DCM, 25.9 mL, 25.9 mmol, 2.4 equiv.) and **S8** (10.8 mmol, 1.0 equiv.). The reaction mixture was stirred at -78 ºC for 10 minutes, then treated with the prepared Grignard reagent (**S9**, 1.2 equiv.) dropwise over 10 minutes. The reaction mixture was allowed to stir at -78 ºC for 1 hour and then slowly warm to -20 ºC over the course of 1 hour. The reaction mixture was then poured into aqueous HCl (1 M, 50 mL). The reaction mixture was extracted with pentane (30 mL × 3), and the combined organic layers were washed with saturated brine (10 mL), dried over Na_2SO_4, filtered and concentrated under reduced pressure to afford the crude product as yellow oil as a ca. 4:1 mixture of diastereomers which the major isomer was tentatively assigned the *trans* configuration, which was used for the next step without further purification.

Step b: The above crude product (10.8 mmol, 1.0 equiv.) was dissolved in THF (43 mL, 0.25 M/L) and potassium *tert*-butoxide (*t*-BuOK, 2.42 g, 21.6 mmol, 2.0 equiv.) was added. The mixture was stirred for 3 hours at 25 ºC. The solution was slowly acidified using dilute citric acid until pH 7 was achieved. The mixture was then poured into a separatory funnel and extracted with EtOAc (50 mL × 3). The combined organics were washed with saturated brine (50 mL), dried over anhydrous Na_2SO_4, filtered and concentrated. The crude residue was purified by flash-chromatography on silica gel provide *trans*-acid **S10**.

Step c: To a stirred solution of **S10** (7.3 mmol, 1.0 equiv.) in DCM (50 mL, 0.15 M/L) at 25 ºC under argon was added Et_3N (1.62 mL, 11.68 mmol, 1.6 equiv.). After 5 minutes, 1-ethyl-3-(3-dimethyllaminopropyl) carbodiimide hydrochloride (EDCI, 2.52 g, 13.14 mmol, 1.8 equiv.) and $CH_3NHOCH_3•HCl$ (**S11**, 1.14 g, 11.68 mmol, 1.6 equiv.) were added to the reaction mixture. After stirring for 12 hours, the reaction was acidified with aqueous HCl (1 M, 20 mL) and extracted with DCM (50 mL × 3). The combined organic extracts were washed with saturated brine (20 mL), dried over anhydrous Na_2SO_4 and concentrated under reduced pressure. The residue was purified by flash-column chromatography on silica gel to afford the Weinreb amide.

Step d: To a stirred solution 1,3-dithiane (180 mg, 1.5 mmol, 1.5 equiv.) in dry THF (7.5 mL, 0.2 M/L) was added *t*-BuLi (1.3 M in hexane, 1.0 mL, 1.3 mmol, 1.3 equiv.) dropwise at -78 ºC. The solution was allowed to warm to 0 ºC for 1.5 hours to give **S12**. Then the mixture was cooled to -78 ºC and the above prepared Weinreb amide (1.0 mmol, 1.0 equiv.) in THF (2.0 mL, 0.5 M/L) was added dropwise. The reaction mixture was kept at this temperature for additional 2.5 hours. After

quenching the reaction with ice water, the reaction mixture was extracted with EtOAc (10 mL × 3). The organic extracts were washed by saturated brine (10 mL), dried over Na_2SO_4 and concentrated under reduced pressure, to give the crude product **S13**, which was used for the next step without further purification.

Step e: To a stirred suspension of magnesium turnings (634 mg, 26.4 mmol, 2.0 equiv.) in dry Et_2O (13.2 mL, 1.0 M/L) with mercury(II) chloride (35 mg, 0.13 mmol, 0.01 equiv.) and a crystal of I_2 (2 mg) cooled at 0 ºC was slowly added 80 wt% solution of propargyl bromide in toluene (1.5 mL, 13.2 mmol, 1.0 equiv.). The mixture was stirred at 0 ºC for 1 hour, then warmed to 25 ºC and stirred for another 1 hour. The solution of putative Grignard reagent **S14** was used directly in the next step[6].

To a stirred solution of **S13** (0.5 mmol, 1.0 equiv.) in THF (5 mL, 0.1 M/L) was added dropwise with the prepared Grignard reagent **S14** (1.0 mmol, 2.0 equiv.) at 0 ºC and the reaction mixture was stirred at 25 ºC for 3 hours. After quenching with saturated aqueous NH_4Cl (10 mL), the reaction mixture was extracted by EtOAc (20 mL × 3), washed with saturated brine (20 mL), dried over Na_2SO_4, and evaporated under reduced pressure. The residue was purified by flash-column chromatography on silica gel to afford the alkyne as a 1: 1 mixture of diastereomers.

Step f: TMSOTf (0.14 mL, 0.76 mmol, 2.0 equiv.) was added to a solution of above prepared alkyne (0.38 mmol, 1.0 equiv.) and pyridine (0.092 mL, 1.14 mmol, 3.0 equiv.) in DCM (4 mL, 0.1 M/L) at 0 ºC. After stirring for 1 hour at 25 ºC, the reaction was quenched with saturated aqueous NH_4Cl (10 mL) and the resulting mixture was extracted with DCM (20 mL × 3). The combined organic extracts were washed with saturated brine (10 mL), and dried over anhydrous Na_2SO_4, and concentrated under reduced pressure, to give the crude silyl ether product, which was used for the next step without further purification.

Step g: To a solution of above prepared silyl ether (0.38 mmol, 1.0 equiv.) in acetonitrile (1.2 mL) and water (0.3 mL) were added $CaCO_3$ (114 mg, 1.14 mmol, 3.0 equiv.) and MeI (0.71 mL, 11.4 mmol, 30.0 equiv.), and the solution was stirred at 60 ºC for 12 hours. Then water (10 mL) was added, followed by filtered on diatomite with Et_2O. The mixture was extracted with EtOAc (10 mL × 3) and the combined organic extracts were washed with saturated brine (10 mL), dried over anhydrous Na_2SO_4, concentrated under reduced pressure. The crude product was purified by flash-chromatography on silica gel to give **S15**.

Step h: To a solution of compound **S15** (0.2 mmol, 1.0 equiv.) in dry toluene (2 mL, 0.1 M/L) at 25

°C was added copper(I)-thiophene-2-carboxylate (4 mg, 0.02 mmol, 0.1 equiv.), followed by addition of TsN_3 (42 mg, 0.22 mmol, 1.1 equiv.) via syringe. The reaction mixture was then stirred at the same temperature for 3 hours. Then the organics were evaporated, diluted with a minimal amount of DCM, loaded directly onto a silica gel column and purified with eluent to give the triazole.

Synthesis of compounds 1s and 1u

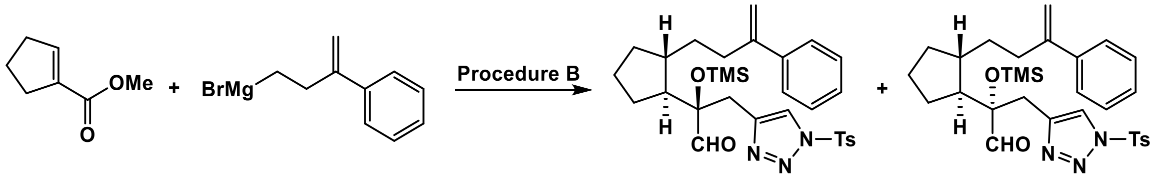

S8a S9a 1s 1u

Data for **1s**

1s (204 mg): colorless oil, 9% overall yield from **S8a** (504 mg);

R*f* = 0.4 (silica gel, hexanes: EtOAc = 5: 1);

IR (film) λ_{max} 2922, 2851, 2357, 1732, 1643, 1485, 1395, 1196, 1011, 843, 812, 671, 584 cm^{-1};

^{1}H NMR (500 MHz, $CDCl_3$) δ 9.56 (s, 1H), 7.96 (d, *J* = 8.4 Hz, 2H), 7.82 (s, 1H), 7.37 – 7.34 (m, 4H), 7.31 (t, *J* = 7.8 Hz, 2H), 7.24 (d, *J* = 7.1 Hz, 1H), 5.24 (s, 1H), 5.01 (s, 1H), 3.11 (d, *J* = 14.7 Hz, 1H), 2.91 (d, *J* = 14.7 Hz, 1H), 2.57 – 2.51 (m, 1H), 2.43 (s, 3H), 2.36 – 2.30 (m, 1H), 1.82 – 1.77 (m, 2H), 1.75 – 1.69 (m, 2H), 1.56 – 1.47 (m, 3H), 1.32 – 1.22 (m, 3H), -0.16 (s, 9H) ppm;

^{13}C NMR (125 MHz, $CDCl_3$) δ 202.9, 148.3, 147.4, 142.9, 141.2, 133.2, 130.5, 128.8, 128.4, 127.5, 126.2, 122.9, 112.5, 85.8, 51.7, 39.3, 35.2, 34.2, 32.5, 32.1, 27.6, 25.3, 22.0, 2.4 ppm;

HRMS (ESI) calcd. for $C_{30}H_{40}N_3O_4SSi$ $[M+H]^+$: 566.2503, found: 566.2510.

Data for **1u**

1u (218 mg): colorless oil, 9% overall yield from **S8a** (540 mg);

R*f* = 0.4 (silica gel, hexanes: EtOAc = 5: 1);

IR (film) λ_{max} 2928, 2860, 2353, 1730, 1599, 1454, 1395, 1198, 1008, 851, 816, 683, 586 cm^{-1};

^{1}H NMR (500 MHz, CD_2Cl_2) δ 9.59 (s, 1H), 7.94 (d, *J* = 8.4 Hz, 2H), 7.77 (s, 1H), 7.40 (dd, *J* = 17.6, 7.7 Hz, 4H), 7.33 (t, *J* = 7.6 Hz, 2H), 7.25 (t, *J* = 7.3 Hz, 1H), 5.31 – 5.25 (m, 1H), 5.10 – 5.04 (m, 1H), 3.01 (d, *J* = 14.8 Hz, 1H), 2.86 (d, *J* = 14.8 Hz, 1H), 2.69 – 2.59 (m, 1H), 2.46 (dd, *J* = 9.2, 6.8 Hz, 1H), 2.43 (s, 3H), 2.06 – 2.00 (m, 1H), 1.86 – 1.81 (m, 1H), 1.77 – 1.69 (m, 1H), 1.64 – 1.58 (m, 2H), 1.52 – 1.47 (m, 2H), 1.37 – 1.33 (m, 2H), 1.28 – 1.26 (m, 1H), -0.19 (s, 9H) ppm;

^{13}C NMR (125 MHz, CD_2Cl_2) δ 202.7, 149.1, 148.1, 143.4, 141.7, 133.5, 131.0, 129.1, 128.9, 127.9, 126.7, 123.3, 112.8, 86.6, 51.2, 39.8, 36.5, 34.6, 33.0, 32.2, 28.5, 26.2, 22.1, 2.5 ppm;

HRMS (ESI) calcd. for $C_{30}H_{40}N_3O_4SSi$ $[M+H]^+$: 566.2503, found: 566.2510.

Synthesis of compounds 1t and 1v

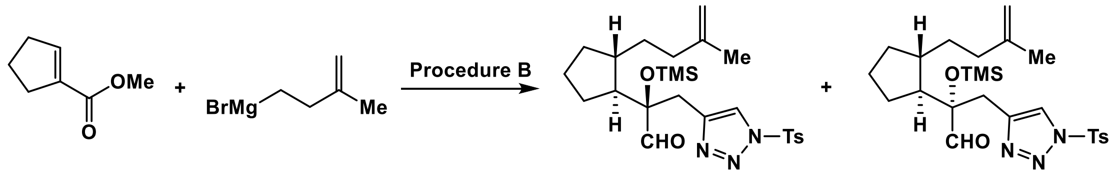

S8a S9b 1t 1v

Data for **1t**

1t (500 mg): colorless oil, 10% overall yield from **S8a** (1.25 g);

R*f* = 0.5 (silica gel, hexanes: EtOAc = 5: 1);

IR (film) λ_{max} 2924, 2853, 2359, 1732, 1635, 1502, 1396, 1260, 1196, 1180, 1011, 843, 812, 669, 584, 542 cm^{-1};

^{1}H NMR (500 MHz, $CDCl_3$) δ 9.69 (s, 1H), 7.97 (d, *J* = 8.4 Hz, 2H), 7.87 (s, 1H), 7.36 (d, *J* = 8.1 Hz, 2H), 4.64 (d, *J* = 25.4 Hz, 2H), 3.18 (d, *J* = 14.7 Hz, 1H), 3.00 (d, *J* = 14.7 Hz, 1H), 2.44 (s, 3H), 2.02 – 1.94 (m, 1H), 1.92 – 1.83 (m, 2H), 1.80 – 1.72 (m, 2H), 1.71 – 1.63 (m, 5H), 1.57 – 1.49 (m, 2H), 1.48 – 1.40 (m, 1H), 1.30 – 1.24 (m, 1H), 1.23 – 1.14 (m, 1H), -0.15 (s, 9H) ppm;

^{13}C NMR (125 MHz, $CDCl_3$) δ 202.9, 147.4, 145.8, 142.9, 133.2, 130.5, 128.9, 122.8, 110.0, 86.0, 51.5, 39.3, 36.6, 34.7, 32.5, 32.3, 27.6, 25.3, 22.6, 22.0, 2.5 ppm;

HRMS (ESI) calcd. for $C_{25}H_{38}N_3O_4SSi$ $[M+H]^+$: 504.2347, found: 504. 2351.

Data for **1v**

1v (200 mg): colorless oil, 9% overall yield from **S8a** (556 mg);

R*f* = 0.5 (silica gel, hexanes: EtOAc = 5: 1);

IR (film) λ_{max} 2924, 2855, 2357, 1730, 1622, 1454, 1386, 1196, 1179, 1009, 843, 816, 671, 584, 569 cm^{-1};

^{1}H NMR (500 MHz, $CDCl_3$) δ 9.69 (s, 1H), 7.96 (d, *J* = 8.4 Hz, 2H), 7.88 (s, 1H), 7.36 (d, *J* = 8.2 Hz, 2H), 4.67 (d, *J* = 21.8 Hz, 2H), 3.19 – 3.06 (m, 2H), 2.43 (s, 3H), 2.06 – 1.90 (m, 3H), 1.89 – 1.83 (m, 1H), 1.70 (s, 3H), 1.69 – 1.64 (m, 2H), 1.60 – 1.47 (m, 3H), 1.45 – 1.38 (m, 1H), 1.33 – 1.24 (m, 2H), -0.12 (s, 9H) ppm;

^{13}C NMR (125 MHz, $CDCl_3$) δ 202.6, 147.4, 146.0, 142.8, 133.2, 130.5, 128.8, 122.8, 109.8, 86.0, 51.3, 39.9, 36.7, 35.7, 32.7, 31.8, 28.1, 25.6, 22.8, 21.9, 2.4 ppm;

HRMS (ESI) calcd. for $C_{25}H_{38}N_3O_4SSi$ $[M+H]^+$: 504.2347, found: 504.2351.

Synthesis of compounds 1w and 1y

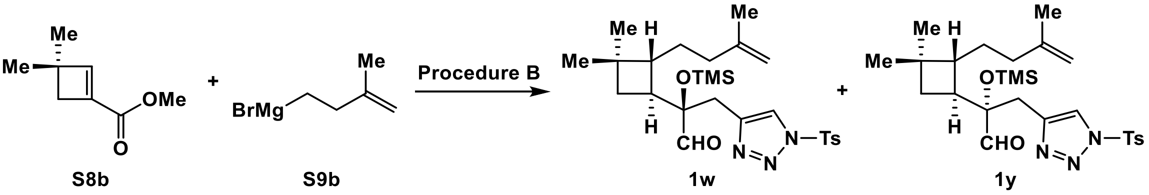

Data for **1w**

1w (144 mg): colorless oil, 12% overall yield from **S8b** (326 mg);

R*f* = 0.5 (silica gel, hexanes: EtOAc = 5: 1);

IR (film) λ_{max} 3273, 2954, 2929, 2456, 1730, 1456, 1330, 1249, 1163, 1066, 977, 840, 551 cm^{-1};

^{1}H NMR (500 MHz, $CDCl_3$) δ 9.58 (s, 1H), 8.11 – 7.92 (m, 2H), 7.87 (s, 1H), 7.36 (d, *J* = 8.1 Hz, 2H), 4.70 (s, 1H), 4.66 (s, 1H), 3.10 (d, *J* = 14.8 Hz, 1H), 2.98 (d, *J* = 14.8 Hz, 1H), 2.44 (s, 3H), 2.14 (q, *J* = 9.3 Hz, 1H), 2.03 (td, *J* = 10.1, 3.8 Hz, 1H), 1.98 – 1.85 (m, 2H), 1.74 – 1.69 (m, 3H), 1.56 – 1.44 (m, 3H), 1.38 – 1.31 (m, 1H), 1.04 (s, 3H), 1.01 (s, 3H), -0.11 (s, 9H) ppm;

^{13}C NMR (125 MHz, $CDCl_3$) δ 202.1, 147.4, 145.9, 142.8, 133.2, 130.5, 128.8, 122.8, 110.0, 84.5, 44.7, 40.9, 36.4, 34.3, 33.2, 31.7, 31.4, 30.1, 22.7, 22.5, 22.0, 2.5 ppm;

HRMS (ESI) calcd. for $C_{26}H_{40}O_4N_3SSi$ [M+H]$^+$: 518.2503, found: 518.2506.

Data for **1y**

1y (155 mg): colorless oil, 12% overall yield from **S8b** (350 mg);

R*f* = 0.5 (silica gel, hexanes: EtOAc = 5: 1);

IR (film) λ_{max} 3072, 2953, 2127, 1734, 1595, 1450, 1396, 1251, 1191, 1091, 1010, 970, 842, 671, 586 cm^{-1};

^{1}H NMR (500 MHz, $CDCl_3$) δ 9.59 (s, 1H), 7.97 (d, *J* = 7.9 Hz, 2H), 7.86 (s, 1H), 7.36 (d, *J* = 8.0 Hz, 2H), 4.63 (d, *J* = 30.6 Hz, 2H), 3.01 (d, *J* = 14.8 Hz, 1H), 2.87 (d, *J* = 14.6 Hz, 1H), 2.44 (s, 3H), 2.11 (q, *J* = 9.1 Hz, 1H), 1.91 – 1.83 (m, 2H), 1.78 – 1.73 (m, 1H), 1.65 (s, 3H), 1.55 (t, *J* = 10.0 Hz, 1H), 1.49 – 1.41 (m, 1H), 1.37 (t, *J* = 9.7 Hz, 1H), 1.25 – 1.15 (m, 1H), 1.01 (s, 3H), 0.99 (s, 3H), -0.08 (s, 9H) ppm;

^{13}C NMR (125 MHz, $CDCl_3$) δ 202.2, 147.3, 145.7, 142.7, 133.3, 130.5, 128.8, 122.8, 110.1, 84.4, 43.3, 41.2, 36.3, 34.1, 33.3, 31.7, 31.3, 29.2, 22.5, 22.0, 2.5 ppm;

HRMS (ESI) calcd. for $C_{26}H_{40}O_4N_3SSi$ [M+H]$^+$: 518.2503, found: 518.2506.

Synthesis of compounds 1x and 1z

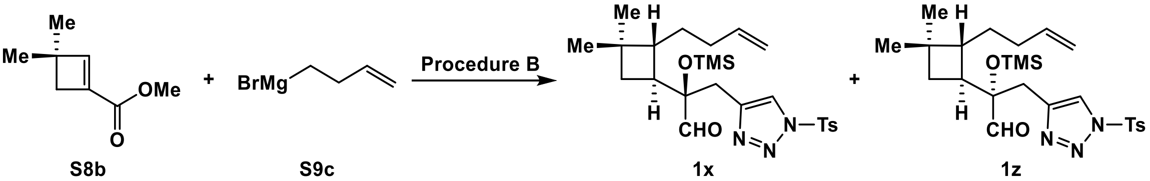

S8b S9c 1x 1z

Data for **1x**

1x (150 mg): colorless oil, 7% overall yield from **S8b** (596 mg);

R*f* = 0.5 (silica gel, hexanes: EtOAc = 5: 1);

IR (film) λ_{max} 3281, 2947, 2876, 2456, 1728, 1458, 1327, 1256, 1179, 1052, 993, 817, 534 cm^{-1};

^{1}H NMR (500 MHz, $CDCl_3$) δ 9.59 (s, 1H), 7.96 (d, *J* = 8.4 Hz, 2H), 7.85 (s, 1H), 7.41 – 7.32 (m, 2H), 5.74 – 5.65 (m, 1H), 5.02 – 4.85 (m, 2H), 3.01 (d, *J* = 14.6 Hz, 1H), 2.87 (d, *J* = 14.6 Hz, 1H), 2.44 (s, 3H), 2.10 (q, *J* = 10.0 Hz, 1H), 1.97 – 1.85 (m, 2H), 1.83 – 1.75 (m, 1H), 1.55 (t, *J* = 10.1 Hz, 1H), 1.45 – 1.34 (m, 2H), 1.17 – 1.09 (m, 1H), 1.01 (s, 3H), 0.98 (s, 3H), -0.08 (s, 9H) ppm;

^{13}C NMR (125 MHz, $CDCl_3$) δ 202.2, 147.4, 142.7, 138.6, 133.3, 130.5, 128.8, 122.8, 114.7, 84.5, 43.1, 41.2, 34.1, 33.3, 32.3, 31.7, 31.2, 30.4, 22.5, 22.0, 2.5 ppm;

HRMS (ESI) calcd. for $C_{25}H_{38}O_4N_3SSi$ [M+H]$^+$: 504.2347, found: 504.2346.

Data for **1z**

1z (153 mg): colorless oil, 7% overall yield from **S8b** (608 mg);

R*f* = 0.5 (silica gel, hexanes: EtOAc = 5: 1);

IR (film) λ_{max} 3254, 2961, 2834, 2454, 1724, 1465, 1337, 1258, 1173, 1012, 982, 840, 541 cm^{-1};

^{1}H NMR (500 MHz, $CDCl_3$) δ 9.57 (s, 1H), 7.96 (d, *J* = 8.4 Hz, 2H), 7.87 (s, 1H), 7.36 (d, *J* = 8.2 Hz, 2H), 5.84 – 5.71 (m, 1H), 5.06 – 4.89 (m, 2H), 3.09 (d, *J* = 14.7 Hz, 1H), 2.97 (d, *J* = 14.7 Hz, 1H), 2.44 (s, 3H), 2.13 (q, *J* = 9.2 Hz, 1H), 2.09 – 1.97 (m, 2H), 1.97 – 1.85 (m, 1H), 1.59 – 1.51 (m, 1H), 1.51 – 1.46 (m, 1H), 1.45 – 1.38 (m, 1H), 1.38 – 1.31 (m, 1H), 1.03 (s, 3H), 1.00 (s, 3H), -0.11 (s, 9H) ppm;

^{13}C NMR (125 MHz, $CDCl_3$) δ 202.1, 147.4, 142.8, 138.6, 133.2, 130.5, 128.8, 122.8, 114.7, 84.5, 44.5, 40.9, 34.3, 33.1, 32.5, 31.7, 31.25, 31.23, 22.6, 22.0, 2.5 ppm;

HRMS (ESI) calcd. for $C_{25}H_{38}O_4N_3SSi$ [M+H]$^+$: 504.2347, found: 504.2346.

2.2.4 Procedure and Characteristic Data for Substrate 1aa.

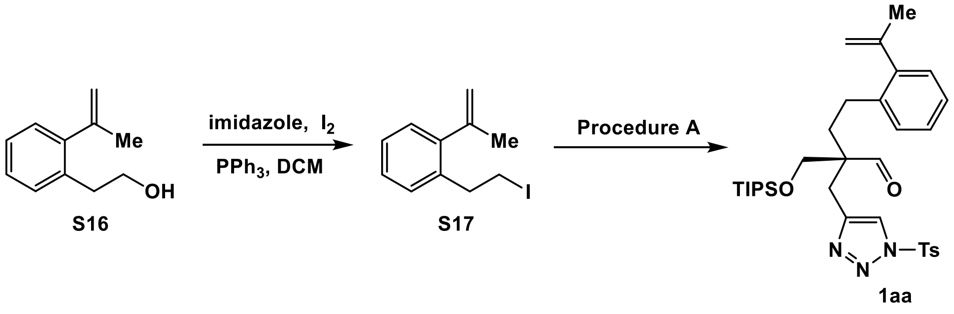

To a solution of **S16**[7] (108 mg, 0.7 mmol, 1.0 equiv.) in DCM (7 mL, 0.1M/L) was added imidazole (71 mg, 1.1 mmol, 1.5 equiv.) and PPh_3 (288 mg, 1.1 mmol, 1.5 equiv.). The resulting solution was cooled to 0 ºC and iodine (279 mg, 1.1 mmol, 1.5 equiv.) was added. The resulting mixture was stirred at 0 ºC for 1 hour, and then diluted with water. The phases were separated, and the aqueous phase was extracted with DCM (15 mL × 3). The combined organic phases were washed with saturated brine (10 mL), dried with Na_2SO_4 and concentrated. And the crude product was purified by flash-column chromatography on silica gel to afford compound **S17** (171 mg, 90% yield).

Compound **S17** underwent steps a-e in Procedure A to give **1aa**.

1aa (78 mg): colorless oil, 19% overall yield from **S16** (108 mg);
R_f = 0.5 (silica gel, hexanes: EtOAc = 5: 1);
IR (film) λ_{max} 2941, 2866, 2359, 1732, 1716, 1259, 1194, 1180, 1094, 1011, 881, 814, 669, 586, 542, 419 cm^{-1};
^{1}H NMR (500 MHz, $CDCl_3$) δ 9.65 (s, 1H), 7.97 (d, *J* = 8.4 Hz, 2H), 7.92 (s, 1H), 7.37 (d, *J* = 8.1 Hz, 2H), 7.17 – 7.09 (m, 3H), 7.08 – 7.01 (m, 1H), 5.11 (d, *J* = 1.8 Hz, 1H), 4.74 (d, *J* = 1.8 Hz, 1H), 3.86 (d, *J* = 10.3 Hz, 1H), 3.70 (d, *J* = 10.3 Hz, 1H), 3.14 – 3.05 (m, 2H), 2.74 – 2.64 (m, 1H), 2.55 – 2.46 (m, 1H), 2.45 (s, 3H), 1.97 (s, 3H), 1.73 – 1.66 (m, 2H), 1.07 – 0.98 (m, 21H) ppm;
^{13}C NMR (125 MHz, $CDCl_3$) δ 204.7, 147.3, 145.5, 143.7, 143.6, 138.0, 133.3, 130.6, 129.3, 128.7, 128.3, 127.2, 126.1, 122.6, 115.1, 65.2, 55.5, 32.2, 27.1, 25.4, 24.5, 22.0, 18.1, 12.0 ppm;
HRMS (ESI) calcd. for $C_{33}H_{48}N_3O_4SSi$ $[M+H]^+$: 610.3129, found: 610.3135.

2.2.5 Procedure and Characteristic Data for Substrate 1ab

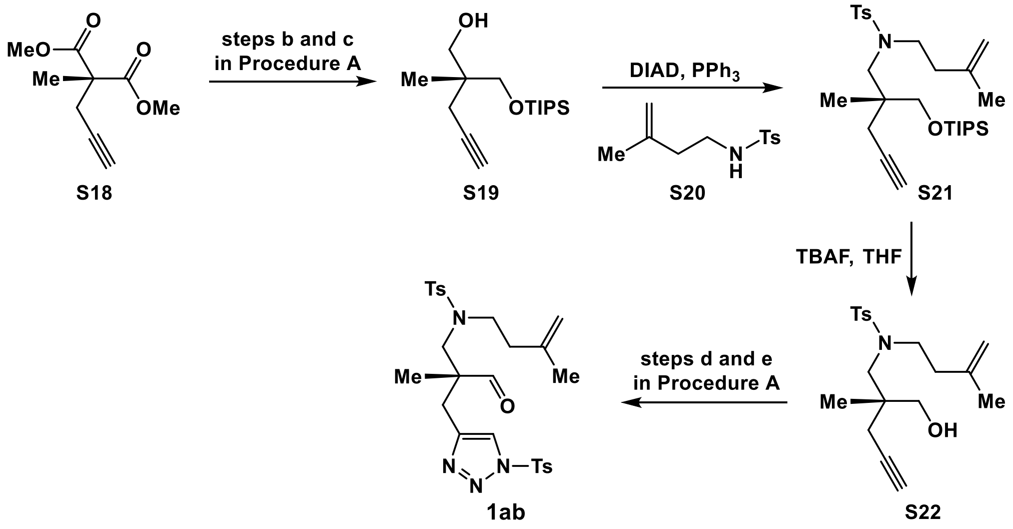

To a stirred solution of **S19** (synthesized from **S18**[8] by steps b and c in Procedure A, 138 mg, 0.5 mmol, 1.0 equiv.), **S20**[9] (143 mg, 0.6 mmol, 1.2 equiv.) and PPh_3 (157 mg, 0.6 mmol, 1.2 equiv.) in THF (5 mL, 0.1 M/L) was added diisopropyl azodicarboxylate (DIAD, 121 mg, 0.6 mmol, 1.2 equiv.) at 0 ºC. The mixture was then stirred overnight at 25 ºC. The mixture was concentrated and filtered through a pad of silica gel. The filtrate was concentrated and the crude product was purified by flash-column chromatography on silica gel to afford compound **S21** as oil (210 mg, 84% yield).

To the solution of **S21** (201 mg, 0.4 mmol, 1.0 equiv.) in THF (4 mL, 0.1 M/L) was added tetrabutylammonium fluoride (TBAF, 1.0 M in THF, 0.5 mL, 0.5 mmol, 1.2 equiv.). The resulting solution was stirred at 25 ºC for 3 hours. Saturated aqueous NH_4Cl (5 mL) was added to quench the reaction, and the reaction mixture was extracted by EtOAc (10 mL × 3). The combined organic layers were washed with saturated brine (10 mL), dried over Na_2SO_4, and concentrated. And the crude product was purified by flash-column chromatography on silica gel to afford compound **S22** as oil (131 mg, 95% yield).

Compound **S22** underwent steps d and e in Procedure A to give **1ab**.

1ab (65 mg): colorless oil, 12% overall yield from **S18** (184 mg);
R*f* = 0.3 (silica gel, hexanes: EtOAc = 3: 1);
IR (film) λ_{max} 2930, 2357, 1730, 1581, 1456, 1355, 1339, 1190, 1180, 1159, 972, 814 cm^{-1};
^{1}H NMR (500 MHz, $CDCl_3$) δ 9.71 (s, 1H), 8.02 (s, 1H), 7.98 (d, *J* = 8.4 Hz, 2H), 7.63 (d, *J* = 8.2 Hz, 2H), 7.37 (d, *J* = 8.2 Hz, 2H), 7.30 (d, *J* = 8.1 Hz, 2H), 4.69 (s, 1H), 4.54 (s, 1H), 3.47 – 3.33 (m,

2H), 3.18 – 3.01 (m, 3H), 2.95 (d, J = 15.0 Hz, 1H), 2.43 (s, 6H), 2.04 (t, J = 8.3 Hz, 2H), 1.60 (s, 3H), 1.12 (s, 3H) ppm;

^{13}C NMR (125 MHz, $CDCl_3$) δ 204.0, 147.4, 143.9, 143.0, 142.1, 136.1, 133.1, 130.6, 130.0, 128.8, 127.5, 123.0, 112.2, 52.9, 50.5, 49.3, 35.6, 29.7, 22.6, 22.0, 21.7, 18.7 ppm;

HRMS (ESI) calcd. for $C_{26}H_{33}N_4O_5S_2$ $[M+H]^+$: 545.1887, found: 545.1889.

2.2.6 Procedure and Characteristic Data for Substrate 1ai

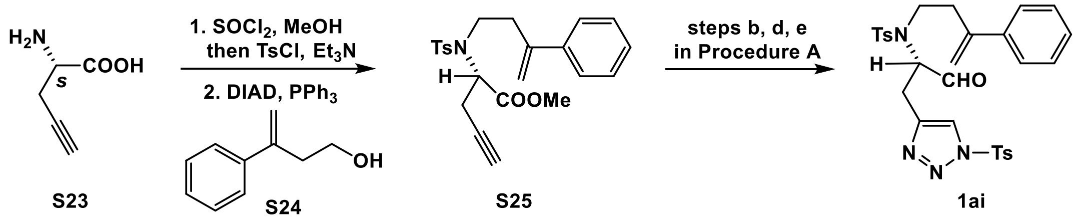

To solution of methanol (21 mL, 0.2 M/L) was added thionyl chloride ($SOCl_2$, 0.61 mL, 8.4 mmol, 2.0 equiv.) drop-wise in 10 minutes at 0 ºC. Then the chiral amino acid **S23** (475 mg, 4.2 mmol, 1.0 equiv.) was added. The reaction mixture was stirred at 25 ºC for 12 hours, and was evaporated under vacuum to afford the crude product. The crude product (1.0 equiv.) was dissolved in anhydrous DCM (21 mL, 0.2 M/L) and then the solution was cooled to 0 ºC followed by addition of *p*-toluene sulfonyl chloride (TsCl, 963 mg, 5.04 mmol, 1.2 equiv.) and Et_3N (1.7 mL, 12.4 mmol, 3.0 equiv.). The reaction mixture was continuously stirred for 2 hours at 25 ºC and TLC showed the consumption of the starting material. The reaction mixture was diluted with water (30 mL). The aqueous layer was extracted with DCM (30 mL × 3), washed with brine (20 mL) and dried over Na_2SO_4. The reaction mixture was evaporated under vacuum to afford the crude product, which was used for the next step without further purification.

To a stirred solution of above product (1.0 equiv.), **S24** (740 mg, 5.0 mmol, 1.2 equiv.) and PPh_3 (1.32 g, 5.0 mmol, 1.2 equiv.) in THF (42 mL, 0.1 M/L) was added diisopropyl azodicarboxylate (DIAD, 1.01 g, 5.0 mmol, 1.2 equiv.) at 0 ºC. The mixture was then stirred overnight at 25 ºC. The mixture was filtered through a pad of silica gel. The filtrate was concentrated and the crude product was purified by flash-column chromatography on silica gel to afford compound **S25** (1.31 g, 76% yield for 2 steps).

Compound **S25** underwent steps b, d and e in procedure A to give compound **1ai**.

1ai (607 mg): colorless oil, 25% overall yield from **S23** (475 mg);

R_f = 0.2 (silica gel, hexanes: EtOAc = 2: 1);

$[\alpha]_D^{25}$ = -10.00 (*c* = 0.5, CH_2Cl_2);

IR (film) λ_{max} 3475, 2881, 2830, 1736, 1642, 1598, 1495, 1447, 1395, 1334, 1157, 1092, 1012, 970, 814, 672, 585 cm^{-1};

^{1}H NMR (500 MHz, $CDCl_3$) δ 9.65 (s, 1H), 7.92 (d, *J* = 8.4 Hz, 2H), 7.60 (d, *J* = 3.7 Hz, 2H), 7.58 (s, 1H), 7.35 (d, *J* = 8.2 Hz, 3H), 7.33 – 3.30 (m, 4H), 7.27 (s, 1H), 7.25 (s, 1H), 5.33 (s, 1H), 5.04 (s, 1H), 4.44 (dd, *J* = 8.4, 5.9 Hz, 1H), 3.39 – 3.26 (m, 2H), 3.10 – 3.03 (m, 1H), 2.84 (dd, *J* = 15.5, 8.4 Hz, 1H), 2.71 (t, *J* = 7.6 Hz, 2H), 2.44 (s, 3H), 2.42 (s, 3H) ppm;

^{13}C NMR (125 MHz, $CDCl_3$) δ 198.1, 147.5, 144.5, 144.2, 143.2, 139.5, 136.9, 133.1, 130.6, 130.1, 128.8, 128.7, 128.1, 127.3, 126.0, 122.2, 115.1, 66.4, 47.3, 36.2, 23.8, 22.0, 21.7 ppm;

HRMS (ESI) calcd. for $C_{29}H_{31}N_4O_5S_2$ $[M+H]^+$: 579.1730, found: 579.1744.

2.2.7 Procedure and Characteristic Data for Substrate 1aj

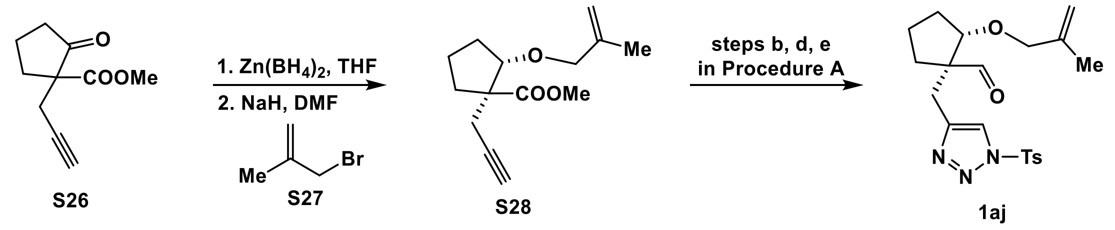

To a stirred solution of **S26**[10] (200 mg, 1.1 mmol, 1.0 equiv.) in THF (11 mL, 0.1 M/L) at 0 ºC was added dropwise $Zn(BH_4)_2$ (1.0 M in THF, 1.7 mL, 1.7 mmol, 1.5 equiv.), the resulted mixture was allowed to warm to 25 ºC and stirred for 2 hours. The mixture was quenched with saturated aqueous NH_4Cl (10 mL) at 0 ºC, extracted with EtOAc (20 mL × 3), washed with water (10 mL) and then saturated brine (10 mL). The organic layer was dried over Na_2SO_4, filtered and concentrated. The residue was purified by flash-column chromatography on silica gel to give the alcohol (121 mg, 60% yield) as colorless oil (*dr* = 8: 1)[11].

The above alcohol (121 mg, 0.66 mmol, 1.0 equiv.) was dissolved in DMF (3.3 mL, 0.2 M/L) at 0 ºC. NaH (60% dispersion in mineral oil, 32 mg, 0.8 mmol, 1.2 equiv.) was added slowly in portion and the reaction mixture was stirred for 30 minutes at 25 ºC. Then 3-bromo-2-methylprop-1-ene **S27** (107 mg, 0.8 mmol, 1.2 equiv.) in DMF (1 mL) was added. The reaction mixture was stirred at 25 ºC for 4 hours. The mixture was quenched with saturated aqueous NH_4Cl (5 mL) at 0 ºC and extracted with EtOAc (10 mL × 3). The combined organic extracts were washed with saturated brine (10 mL) and dried over Na_2SO_4. Solvent was removed by rotary evaporation and the residue was purified by flash-column chromatography on silica gel to provide **S28** (117 mg, 75% yield) as colorless oil.

Compound **S28** underwent steps b, d and e in procedure A to give compound **1aj**.

1aj (84 mg): colorless oil, 15% overall yield from **S26** (250 mg);
R*f* = 0.4 (silica gel, hexanes: EtOAc = 5: 1);
IR (film) λ_{max} 3428, 1962, 1989, 1715, 1601, 1454, 1169, 1123, 1024, 1011, 816, 685, 569 cm^{-1};
^{1}H NMR (500 MHz, $CDCl_3$) δ 9.53 (s, 1H), 7.94 (d, *J* = 8.4 Hz, 2H), 7.90 (s, 1H), 7.36 (d, *J* = 8.1 Hz, 2H), 4.92 (s, 1H), 4.86 (s, 1H), 3.96 (t, *J* = 6.3 Hz, 1H), 3.85 (d, *J* = 12.4 Hz, 1H), 3.71 (d, *J* = 12.4 Hz, 1H), 3.24 (d, *J* = 15.2 Hz, 1H), 2.99 (d, *J* = 15.1 Hz, 1H), 2.43 (s, 3H), 1.98 – 1.91 (m, 1H), 1.88 – 1.78 (m, 2H), 1.77 – 1.71 (m, 1H), 1.68 (s, 3H), 1.66 – 1.61 (m, 1H), 1.59 – 1.53 (m, 1H) ppm;
^{13}C NMR (125 MHz, $CDCl_3$) δ 203.7, 147.3, 144.8, 142.0, 133.3, 130.5, 128.7, 122.7, 112.4, 81.5, 73.7, 61.2, 30.3, 30.0, 25.0, 21.9, 20.8, 19.6 ppm;
HRMS (ESI) calcd. for $C_{20}H_{26}N_3O_4S$ $[M+H]^+$: 404.1639, found: 404.1642.

3. General Procedure for Synthesis of 3a-3aj

3.1 Procedure for Synthesis of 3a-3i, 3m-3q and 3s-3aj.

An oven-dried tube was charged with triazole compound **1** (0.1 mmol, 1.0 equiv.), 3Å MS (30 mg), and $Rh_2(OCt)_4$ (4 mg, 0.005 mmol, 0.05 equiv.). The tube was evacuated and backfilled with argon (repeated three times). Then DCE (2 mL, 0.05 M/L) was added into the reaction via syringe. The reaction mixture was stirred at 85 ºC for 3 hours. The solution was then cooled to 25 ºC. THF (4 mL) was added into the reaction via syringe. The solution was cooled to 0 ºC. $LiAlH_4$ (1.0 M in THF, 0.2 mL, 0.2 mmol, 2.0 equiv.) was added dropwise and the solution was stirred at 0 ºC for 1.5 hours. Following quenching with saturated aqueous potassium sodium tartrate (6 mL), the reaction mixture was extracted by EtOAc (30 mL × 3), the combined organic layers were washed with saturated brine (10 mL), dried over Na_2SO_4, concentrated in vacuum and purified by column chromatography on silica gel to give the products **3a-3i**, **3m-3q** and **3s-3aj**.

3.2 Procedure for Synthesis of 3j-3l and 3r

An oven-dried tube was charged with triazole compound **1** (0.1 mmol, 1.0 equiv), 3Å MS (30 mg), and $Rh_2(OCt)_4$ (4 mg, 0.005 mmol, 0.05 equiv.). The tube was evacuated and backfilled with argon (repeated three times). Then, DCE (2 mL, 0.05 M/L) was added into the reaction via syringe. The reaction mixture was stirring at 85 ºC for 3 h. The solution was then allowed to cool to 25 ºC and wet

basic alumina (500 mg) was added into the reaction. The solution was stirred for 1.5 hours. The residue was concentrated in vacuum, purified by a flash-column chromatography on silica gel to afford **3j**-**3l** and **3r**.

Synthesis of compound 3a

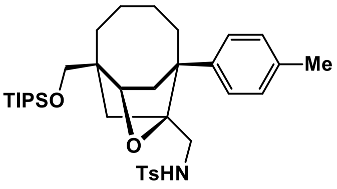

3a

3a (139 mg): white solid, 81% yield from **1a** (179 mg), purified by flash-column chromatography on silica gel (hexanes: EtOAc = 20: 1 ~ 5: 1);

R*f* = 0.3 (silica gel, hexanes: EtOAc = 9: 1);

m.p. = 182 - 183 ºC;

IR (film) λ_{max} 3273, 2926, 2864, 2359, 1601, 1506, 1456, 1337, 1264, 1165, 1098, 1069, 881, 814, 773, 660, 554 cm^{-1};

^{1}**H NMR** (500 MHz, $CDCl_3$) δ 7.42 (d, *J* = 8.2 Hz, 2H), 7.16 (t, *J* = 8.6 Hz, 4H), 6.98 (d, *J* = 8.1 Hz, 2H), 4.41 (d, *J* = 5.3 Hz, 1H), 4.15 (dd, *J* = 9.5, 3.1 Hz, 1H), 3.48 (d, *J* = 8.9 Hz, 1H), 3.27 (d, *J* = 8.9 Hz, 1H), 2.60 – 2.49 (m, 2H), 2.39 (s, 3H), 2.28 (s, 3H), 2.19 (dd, *J* = 13.5, 5.4 Hz, 1H), 2.05 – 1.91 (m, 4H), 1.86 – 1.78 (m, 1H), 1.76 – 1.57 (m, 3H), 1.56 – 1.44 (m, 1H), 1.18 – 1.10 (m, 2H), 1.10 – 1.02 (m, 21H) ppm;

^{13}**C NMR** (125 MHz, $CDCl_3$) δ 145.2, 143.1, 136.0, 135.5, 129.5, 128.6, 127.2, 127.1, 91.3, 82.6, 72.0, 51.0, 48.2, 45.8, 36.6, 33.1, 33.0, 29.1, 26.8, 25.8, 21.6, 20.8, 18.3, 18.2, 12.1 ppm;

HRMS (ESI) calcd. for $C_{35}H_{54}NO_4SSi$ $[M+H]^+$: 612.3537, found: 612.3539.

Note: The structure of **3a** was further confirmed by X-ray crystallographic analysis.

Synthesis of compound 3b

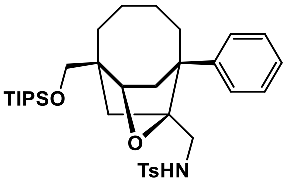

3b

3b (172 mg): white solid, 80% yield from **1b** (224 mg), purified by flash-column chromatography on silica gel (hexanes: EtOAc = 20: 1 ~ 5: 1);

R$_f$ = 0.3 (silica gel, hexanes: EtOAc = 9: 1);

m.p. = 164 - 166 ºC;

IR (film) λ_{max} 3200, 2850, 1661, 1500, 1477, 1435, 1200, 1189, 1011, 814, 777 cm^{-1};

^{1}H NMR (500 MHz, $CDCl_3$) δ 7.40 (d, *J* = 8.2 Hz, 2H), 7.30 (d, *J* = 7.7 Hz, 2H), 7.19 (t, *J* = 7.7 Hz, 2H), 7.16 – 7.09 (m, 3H), 4.43 (d, *J* = 5.3 Hz, 1H), 4.13 (dd, *J* = 9.6, 3.1 Hz, 1H), 3.49 (d, *J* = 8.9 Hz, 1H), 3.27 (d, *J* = 8.9 Hz, 1H), 2.61 – 2.50 (m, 2H), 2.38 (s, 3H), 2.32 (dt, *J* = 15.2, 4.0 Hz, 1H), 2.23 (dd, *J* = 13.5, 5.4 Hz, 1H), 2.09 – 2.00 (m, 2H), 1.98 – 1.90 (m, 2H), 1.87 – 1.81 (m, 1H), 1.77 – 1.60 (m, 3H), 1.57 – 1.49 (m, 1H), 1.17 (d, *J* = 13.3 Hz, 1H), 1.01 – 0.93 (m, 21H) ppm;

^{13}C NMR (125 MHz, $CDCl_3$) δ 148.2, 143.2, 136.0, 129.6, 128.0, 127.3, 127.0, 126.0, 91.3, 82.6, 72.0, 51.4, 48.2, 45.8, 36.5, 33.2, 33.0, 29.2, 26.8, 25.8, 21.6, 18.3, 12.1 ppm;

HRMS (ESI) calcd. for $C_{34}H_{52}NO_4SSi$ $[M+H]^+$: 598.3381, found: 598.3379.

Note: The structure of **3b** was further confirmed by X-ray crystallographic analysis.

Synthesis of compound 3c

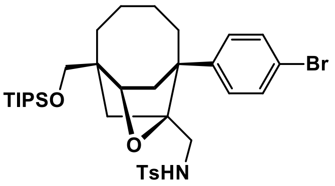

3c

3c (166 mg): white solid, 69% yield from **1c** (250 mg), purified by flash-column chromatography on silica gel (hexanes: EtOAc = 20: 1 ~ 5: 1);

R$_f$ = 0.4 (silica gel, hexanes: EtOAc = 9: 1);

m.p. = 169 - 170 ºC;

IR (film) λ_{max} 3671, 3262, 2940, 2864, 1723, 1597, 1461, 1334, 1168, 1069, 994, 883, 814, 692, 553 cm^{-1};

^{1}H NMR (500 MHz, $CDCl_3$) δ 7.40 (d, *J* = 8.2 Hz, 2H), 7.27 (d, *J* = 5.5 Hz, 2H), 7.22 (d, *J* = 8.0 Hz, 2H), 7.13 (d, *J* = 8.7 Hz, 2H), 4.41 (d, *J* = 5.3 Hz, 1H), 4.14 – 4.11 (m, 1H), 3.48 (d, *J* = 8.9 Hz, 1H), 3.27 (d, *J* = 8.9 Hz, 1H), 2.55 (dd, *J* = 12.9, 9.8 Hz, 2H), 2.43 (s, 3H), 2.24 (dt, *J* = 15.6, 4.0 Hz, 1H), 2.11 (dd, *J* = 13.6, 5.4 Hz, 1H), 2.03 – 1.92 (m, 3H), 1.88 – 1.81 (m, 1H), 1.78 (dd, *J* = 12.7, 2.7 Hz, 1H), 1.76 – 1.62 (m, 3H), 1.55 – 1.48 (m, 1H), 1.22 (d, *J* = 13.3 Hz, 1H), 1.10 – 1.03 (m, 21H) ppm;

^{13}C NMR (125 MHz, $CDCl_3$) δ 159.4, 150.1, 143.2, 136.0, 129.6, 128.8, 127.0, 119.8, 113.9, 111.0, 91.2, 82.6, 72.0, 55.3, 51.5, 48.2, 45.7, 36.7, 33.0, 29.1, 16.8, 25.8, 21.6, 18.26, 18.25, 12.1 ppm;

HRMS (ESI) calcd. for $C_{34}H_{51}BrNO_4SSi$ $[M+H]^+$: 676.2486, found: 676.2503.

Synthesis of compound 3d

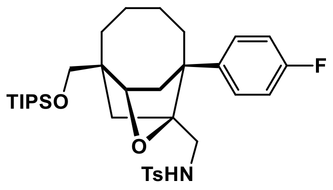

3d

3d (148 mg): white solid, 67% yield from **1d** (230 mg), purified by flash-column chromatography on silica gel (hexanes: EtOAc = 20: 1 ~ 5: 1);

R*f* = 0.4 (silica gel, hexanes: EtOAc = 5: 1);

m.p. = 163 - 164 ºC;

IR (film) λ_{max} 3256, 2941, 2864, 2357, 1599, 1510, 1464, 1335, 1236, 1167, 1096, 993, 883, 814, 681, 554, 519 cm^{-1};

^{1}H NMR (500 MHz, $CDCl_3$) δ 7.41 (d, *J* = 8.3 Hz, 2H), 7.22 (dd, *J* = 8.9, 5.3 Hz, 2H), 7.18 (d, *J* = 8.0 Hz, 2H), 6.83 (t, *J* = 8.7 Hz, 2H), 4.41 (d, *J* = 5.3 Hz, 1H), 4.14 (dd, *J* = 9.8, 2.4 Hz, 1H), 3.48 (d, *J* = 8.9 Hz, 1H), 3.27 (d, *J* = 8.9 Hz, 1H), 2.57 – 2.50 (m, 2H), 2.40 (s, 3H), 2.30 – 2.22 (m, 1H), 2.18 – 2.11 (m, 1H), 2.04 – 1.92 (m, 3H), 1.88 – 1.80 (m, 1H), 1.80 – 1.62 (m, 4H), 1.55 – 1.43 (m, 1H), 1.20 (d, *J* = 13.3 Hz, 1H), 1.12 – 1.04 (m, 21H) ppm;

^{13}C NMR (125 MHz, $CDCl_3$) δ 161.3 (d, *J* = 245.7 Hz), 143.9 (d, *J* = 3.3 Hz), 143.6, 135. 5, 129.6, 128.8 (d, *J* = 7.5 Hz), 127.0, 114.5 (d, *J* = 20.8 Hz), 91.2, 82.6, 72.0, 51.1, 48.2, 45.7, 36.9, 33.1, 32.8, 29.1, 26.8, 25.7, 21.6, 18.2, 12.1 ppm;

HRMS (ESI) calcd. for $C_{34}H_{51}FNO_4SSi$ $[M+H]^+$: 616.3287, found: 616.3290.

Note: The structure of **3d** was further confirmed by X-ray crystallographic analysis.

Synthesis of compound 3e

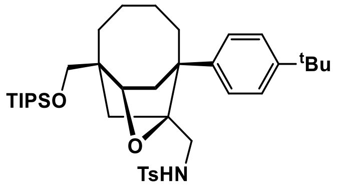

3e

3e (138 mg): white solid, 70% yield from **1e** (204 mg), purified by flash-column chromatography on silica gel (hexanes: EtOAc = 20: 1 ~ 5: 1);

R*f* = 0.6 (silica gel, hexanes: EtOAc = 4: 1);

m.p. = 172 - 173 ºC;

IR (film) λ_{max} 3283, 2936, 2864, 2359, 1508, 1454, 1327, 1167, 1067, 1008, 816, 669, 554, 419 cm^{-1};
^{1}H NMR (500 MHz, $CDCl_3$) δ 7.43 (d, *J* = 8.2 Hz, 2H), 7.25 – 7.19 (m, 4H), 7.14 (d, *J* = 8.0 Hz, 2H), 4.42 (d, *J* = 5.3 Hz, 1H), 4.17 (dd, *J* = 9.7, 3.1 Hz, 1H), 3.49 (d, *J* = 8.9 Hz, 1H), 3.27 (d, *J* = 8.9 Hz, 1H), 2.60 (dd, *J* = 13.0, 9.8 Hz, 1H), 2.51 (d, *J* = 13.5 Hz, 1H), 2.34 (s, 3H), 2.33 – 2.28 (m, 1H), 2.23 (dd, *J* = 13.5, 5.4 Hz, 1H), 2.04 (t, *J* = 12.3 Hz, 2H), 1.94 (dd, *J* = 13.0, 3.2 Hz, 2H), 1.86 – 1.80 (m, 1H), 1.77 – 1.64 (m, 3H), 1.55 – 1.49 (m, 1H), 1.26 (s, 9H), 1.20 (d, *J* = 13.2 Hz, 1H), 1.11 – 1.03 (m, 21H) ppm;
^{13}C NMR (125 MHz, $CDCl_3$) δ 148.9, 145.1, 143.1, 136.3, 129.6, 127.0, 126.9, 124.8, 91.4, 82.6, 72.0, 50.9, 48.2, 45.6, 36.4, 34.3, 33.3, 32.8, 31.4, 29.2, 26.8, 25.9, 21.6, 18.3, 12.2 ppm;
HRMS (ESI) calcd. for $C_{38}H_{60}NO_4SSi$ $[M+H]^+$: 654.4007, found: 654.4005.

Synthesis of compound 3f

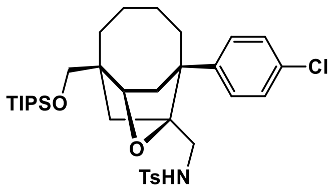

3f

3f (106 mg): white solid, 70% yield from **1f** (157 mg), purified by flash-column chromatography on silica gel (hexanes: EtOAc = 20: 1 ~ 5: 1);
R_f = 0.3 (silica gel, hexanes: EtOAc = 9: 1);
m.p. = 163 - 164 ºC;
IR (film) λ_{max} 3667, 3242, 2942, 2864, 1596, 1495, 1464, 1414, 1335, 1168, 1071, 992, 884, 813, 727, 690, 553 cm^{-1};
^{1}H NMR (500 MHz, $CDCl_3$) δ 7.40 (d, *J* = 8.2 Hz, 2H), 7.23 – 7.17 (m, 4H), 7.10 (d, *J* = 8.8 Hz, 2H), 4.41 (d, *J* = 5.3 Hz, 1H), 4.16 (dd, *J* = 9.9, 2.5 Hz, 1H), 3.48 (d, *J* = 8.9 Hz, 1H), 3.27 (d, *J* = 8.9 Hz, 1H), 2.54 (dt, *J* = 12.6, 4.5 Hz, 2H), 2.42 (s, 3H), 2.28 – 2.20 (m, 1H), 2.12 (dd, *J* = 13.6, 5.4 Hz, 1H), 2.04 – 1.92 (m, 3H), 1.87 – 1.80 (m, 1H), 1.80 – 1.63 (m, 4H), 1.55 – 1.47 (m, 1H), 1.21 (d, *J* = 13.3 Hz, 1H), 1.01 – 1.04 (m, 21H) ppm;
^{13}C NMR (125 MHz, $CDCl_3$) δ 146.8, 143.6, 135.3, 132.1, 129.6, 128.7, 127.8, 127.0, 91.2, 82.7, 71.9, 51.2, 48.2, 45.7, 36.8, 32.9, 32.7, 29.1, 26.8, 25.7, 21.6, 18.3, 18.2, 12.1 ppm;
HRMS (ESI) calcd. for $C_{34}H_{51}ClNO_4SSi$ $[M+H]^+$: 632.2991, found: 632.2989.
Note: The structure of **3f** was further confirmed by X-ray crystallographic analysis.

Synthesis of compound 3g

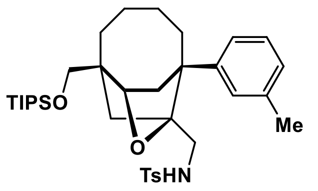

3g

3g (151 mg): white solid, 75% yield from **1g** (210 mg), purified by flash-column chromatography on silica gel (hexanes: EtOAc = 20: 1 ~ 5: 1);

R*f* = 0.3 (silica gel, hexanes: EtOAc = 9: 1);

m.p. = 182 - 184 ºC;

IR (film) λ_{max} 3649, 3245, 2938, 2862, 1600, 1463, 1410, 1336, 1167, 1070, 1013, 884, 809, 748, 688, 555 cm^{-1};

^{1}H NMR (500 MHz, $CDCl_3$) δ 7.41 (d, *J* = 8.3 Hz, 2H), 7.15 (d, *J* = 8.0 Hz, 2H), 7.11 – 7.06 (m, 3H), 6.96 – 6.91 (m, 1H), 4.42 (d, *J* = 5.3 Hz, 1H), 4.15 (dd, *J* = 9.7, 3.2 Hz, 1H), 3.49 (d, *J* = 8.9 Hz, 1H), 3.27 (d, *J* = 8.9 Hz, 1H), 2.58 (dd, *J* = 12.8, 9.7 Hz, 1H), 2.51 (d, *J* = 13.5 Hz, 1H), 2.39 (s, 3H), 2.34 – 2.28 (m, 1H), 2.26 (s, 3H), 2.22 (dd, *J* = 13.5, 5.4 Hz, 1H), 2.03 (d, *J* = 13.2 Hz, 2H), 1.98 – 1.90 (m, 2H), 1.86 – 1.80 (m, 1H), 1.76 – 1.62 (m, 3H), 1.56 – 1.48 (m, 1H), 1.16 (d, *J* = 13.2 Hz, 1H), 1.11 – 1.05 (m, 21H) ppm;

^{13}C NMR (125 MHz, $CDCl_3$) δ 148.1, 143.2, 137.4, 135.9, 129.5, 128.1, 127.9, 127.0, 126.7, 124.3, 91.2, 82.6, 72.0, 51.3, 48.2, 45.8, 36.4, 33.2, 33.0, 29.1, 26.8, 25.8, 21.8, 21.6, 18.26, 18.25, 12.1 ppm;

HRMS (ESI) calcd. for $C_{35}H_{54}NO_4SSi$ $[M+H]^+$: 612.3537, found: 612.3539.

Note: The structure of **3g** was further confirmed by X-ray crystallographic analysis.

Synthesis of compound 3h

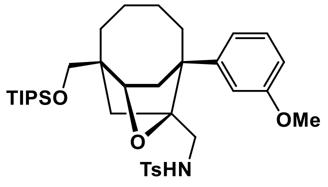

3h

3h (137 mg): white solid, 78% yield from **1h** (183 mg), purified by flash-column chromatography on silica gel (hexanes: EtOAc = 20: 1 ~ 5: 1);

R*f* = 0.3 (silica gel, hexanes: EtOAc = 9: 1);

m.p. = 159 - 160 ºC;

IR (film) λ_{max} 3273, 2941, 2864, 2359, 1599, 1472, 1335, 1256, 1165, 1069, 881, 704, 667 cm^{-1};

^{1}H NMR (500 MHz, $CDCl_3$) δ 7.43 (d, *J* = 8.3 Hz, 2H), 7.16 (d, *J* = 8.0 Hz, 2H), 7.11 (t, *J* = 8.0 Hz, 1H), 6.89 (dd, *J* = 7.9, 2.0 Hz, 1H), 6.84 (t, *J* = 2.2 Hz, 1H), 6.66 (dd, *J* = 8.1, 2.4 Hz, 1H), 4.40 (d, *J* = 5.3 Hz, 1H), 4.18 (dd, *J* = 9.6, 3.3 Hz, 1H), 3.74 (s, 3H), 3.48 (d, *J* = 8.9 Hz, 1H), 3.27 (d, *J* = 8.9 Hz, 1H), 2.61 (dd, *J* = 12.9, 9.6 Hz, 1H), 2.52 (d, *J* = 13.5 Hz, 1H), 2.39 (s, 3H), 2.31 (dt, *J* = 14.9, 3.9 Hz, 1H), 2.19 (dd, *J* = 13.5, 5.4 Hz, 1H), 2.05 – 1.92 (m, 4H), 1.87 – 1.78 (m, 1H), 1.75 – 1.65 (m, 2H), 1.54 – 1.47 (m, 1H), 1.16 (d, *J* = 13.2 Hz, 1H), 1.14 – 1.03 (m, 22H) ppm;

^{13}C NMR (125 MHz, $CDCl_3$) δ 159.4, 150.1, 143.2, 136.0, 129.6, 128.8, 127.0, 119.8, 113.9, 111.0, 91.2, 82.6, 72.0, 55.3, 51.5, 48.2, 45.7, 36.7, 33.0, 29.1, 26.8, 25.8, 21.6, 18.26, 18.25, 12.1 ppm;

HRMS (ESI) calcd. for $C_{35}H_{54}NO_5SSi$ $[M+H]^+$: 628.3486, found: 628.3484.

Note: The structure of **3h** was further confirmed by X-ray crystallographic analysis.

Synthesis of compound 3i

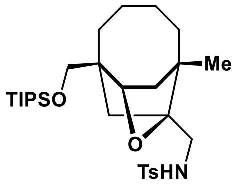

3i

3i (79 mg): colorless oil, 61% yield from **1i** (135 mg), purified by flash-column chromatography on silica gel (hexanes: EtOAc = 20: 1 ~ 5: 1);

R_f = 0.5 (silica gel, hexanes: EtOAc = 4: 1);

IR (film) λ_{max} 3273, 2924, 2864, 2361, 1598, 1512, 1458, 1339, 1261, 1163, 1094, 881, 816, 669, 552, 419 cm^{-1};

^{1}H NMR (500 MHz, $CDCl_3$) δ 7.73 (d, *J* = 8.3 Hz, 2H), 7.31 (d, *J* = 8.0 Hz, 2H), 4.53 (dd, *J* = 8.5, 4.2 Hz, 1H), 4.15 (d, *J* = 4.9 Hz, 1H), 3.43 (d, *J* = 8.9 Hz, 1H), 3.24 (d, *J* = 8.9 Hz, 1H), 3.15 – 3.09 (m, 2H), 2.42 (s, 3H), 2.31 (d, *J* = 12.5 Hz, 1H), 1.88 (d, *J* = 12.9 Hz, 1H), 1.78 – 1.66 (m, 3H), 1.57 – 1.46 (m, 3H), 1.42 – 1.31 (m, 2H), 1.13 (dd, *J* = 12.6, 4.8 Hz, 2H), 1.09 – 1.01 (m, 21H), 0.84 (s, 3H) ppm;

^{13}C NMR (125 MHz, $CDCl_3$) δ 143.6, 136.7, 129.9, 127.2, 89.4, 82.6, 72.1, 47.7, 43.5, 43.2, 38.7, 34.2, 32.9, 29.7, 29.4, 26.4, 25.8, 21.7, 18.2, 12.1 ppm;

HRMS (ESI) calcd. for $C_{29}H_{50}NO_4SSi$ $[M+H]^+$: 536.3224, found: 536.3226.

Synthesis of compound 3j

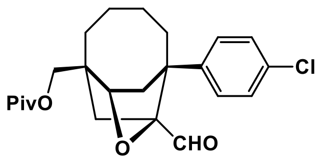

3j

3j (63 mg): colorless oil, 77% yield from **1j** (118 mg), purified by flash-column chromatography on silica gel (hexanes: EtOAc = 20: 1 ~ 5: 1);

R*f* = 0.2 (silica gel, hexanes: EtOAc = 4: 1);

IR (film) λ_{max} 2989, 2929, 1730, 1473,1284, 1151, 1012, 706 cm^{-1};

^{1}H NMR (500 MHz, $CDCl_3$) δ 9.10 (s, 1H), 7.37 (d, *J* = 8.8 Hz, 2H), 7.27 (d, *J* = 3.0 Hz, 2H), 4.51 (d, *J* = 5.2 Hz, 1H), 3.91 – 3.85 (m, 2H), 2.62 (d, *J* = 13.4 Hz, 1H), 2.45 (d, *J* = 13.2 Hz, 1H), 2.38 – 2.25 (m, 3H), 2.06 – 1.98 (m, 1H), 1.93 – 1.88 (m, 1H), 1.75 – 1.62 (m, 3H), 1.57 – 1.53 (m, 1H), 1.22 (s, 9H), 1.13 (d, *J* = 13.2 Hz, 1H) ppm;

^{13}C NMR (125 MHz, $CDCl_3$) δ 201.7, 178.6, 145.0, 132.6, 129.1, 128.4, 96.0, 83.4, 71.8, 52.2, 45.5, 39.1, 36.3, 33.7, 33.1, 28.4, 27.3, 26.2, 25.6 ppm;

HRMS (ESI) calcd. for $C_{23}H_{29}ClNaO_4$ $[M+Na]^+$: 427.1647, found: 427.1644.

Synthesis of compound 3k

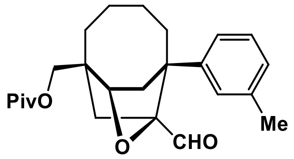

3k

3k (63 mg): colorless oil, 75% yield from **1k** (124 mg), purified by flash-column chromatography on silica gel (hexanes: EtOAc = 20: 1 ~ 5: 1);

R*f* = 0.3 (silica gel, hexanes: EtOAc = 9: 1);

IR (film) λ_{max} 3445, 2972, 2928, 2359, 1730, 1634, 1557, 1464, 1283, 1155, 1086, 1034, 800, 704, 530 cm^{-1};

^{1}H NMR (500 MHz, $CDCl_3$) δ 9.05 (s, 1H), 7.26 – 7.15 (m, 3H), 7.00 (d, *J* = 7.1 Hz, 1H), 4.50 (d, *J* = 5.3 Hz, 1H), 3.93 – 3.80 (m, 2H), 2.57 (d, *J* = 13.4 Hz, 1H), 2.46 (d, *J* = 13.1 Hz, 1H), 2.41 (dd, *J* = 13.4, 5.3 Hz, 1H), 2.39 – 2.34 (m, 1H), 2.33 (s, 3H), 2.30 – 2.23 (m, 1H), 2.02 – 1.96 (m, 1H), 1.92 – 1.85 (m, 1H), 1.75 – 1.60 (m, 3H), 1.58 – 1.51 (m, 1H), 1.21 (s, 9H), 1.12 (d, *J* = 13.2 Hz, 1H) ppm;

^{13}C NMR (125 MHz, $CDCl_3$) δ 201.8, 178.6, 146.2, 137.8, 128.3, 128.2, 127.3, 124.7, 96.0, 83.4, 71.9, 52.4, 45.5, 39.1, 35.8, 33.5, 33.3, 28.4, 27.3, 26.2, 25.7, 21.9 ppm;

HRMS (ESI) calcd. for $C_{24}H_{32}NaO_4$ $[M+Na]^+$: 407.2193, found: 407.2195.

Synthesis of compound 3l

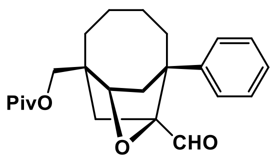

3l

3l (69 mg): colorless oil, 78% yield from **1l** (132 mg), purified by flash-column chromatography on silica gel (hexanes: EtOAc = 20: 1 ~ 5: 1);

R*f* = 0.3 (silica gel, hexanes: EtOAc = 9: 1);

IR (film) λ_{max} 2958, 2926, 1728, 1477, 1282, 1155, 746, 700 cm^{-1};

^{1}H NMR (500 MHz, $CDCl_3$) δ 9.04 (s, 1H), 7.43 (d, *J* = 7.4 Hz, 2H), 7.30 (t, *J* = 7.8 Hz, 2H), 7.19 (t, *J* = 7.3 Hz, 1H), 4.51 (d, *J* = 5.2 Hz, 1H), 3.90 – 3.83 (m, 2H), 2.60 (d, *J* = 13.4 Hz, 1H), 2.47 (d, *J* = 13.2 Hz, 1H), 2.44 – 2.35 (m, 2H), 2.33 – 2.27 (m, 1H), 2.03 – 1.97 (m, 1H), 1.74 – 1.68 (m, 3H), 1.27 – 1.23 (m, 1H), 1.21 (s, 9H), 1.11 (d, *J* = 13.2 Hz, 1H) ppm;

^{13}C NMR (125 MHz, $CDCl_3$) δ 201.8, 178.6, 146.3, 128.4, 127.6, 126.6, 96.1, 83.4, 71.9, 52.5, 45.5, 39.1, 35.9, 33.6, 33.2, 28.4, 27.3, 26.2, 25.7 ppm;

HRMS (ESI) calcd. for $C_{23}H_{30}NaO_4$ $[M+Na]^+$: 393.2036, found: 393.2037.

Synthesis of compound 3m

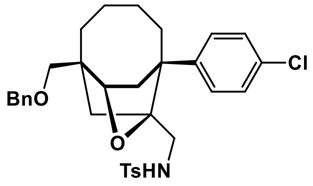

3m

3m (121 mg): colorless oil, 63% yield from **1m** (201 mg), purified by flash-column chromatography on silica gel (hexanes: EtOAc = 20: 1 ~ 5: 1);

R*f* = 0.6 (silica gel, hexanes: EtOAc = 4: 1);

IR (film) λ_{max} 2936, 2359, 1508, 1456, 1321, 1205, 1194, 1013, 818, 689, 678, 567, 419 cm^{-1};

^{1}H NMR (500 MHz, $CDCl_3$) δ 7.42 – 7.27 (m, 7H), 7.18 (dd, *J* = 16.7, 8.4 Hz, 4H), 7.10 (d, *J* = 8.7 Hz, 2H), 4.51 (q, *J* = 12.2 Hz, 2H), 4.40 (d, *J* = 5.3 Hz, 1H), 4.14 (d, *J* = 9.8 Hz, 1H), 3.25 (d, *J* = 8.3 Hz, 1H), 3.07 (d, *J* = 8.3 Hz, 1H), 2.55 (dt, *J* = 9.9, 4.4 Hz, 2H), 2.42 (s, 3H), 2.27 – 2.20 (m, 1H),

2.11 (dd, J = 13.6, 5.4 Hz, 1H), 2.08 (d, J = 13.3 Hz, 1H), 2.01 – 1.91 (m, 2H), 1.87 – 1.79 (m, 1H), 1.79 – 1.62 (m, 4H), 1.57 – 1.51 (m, 1H), 1.31 (d, J = 10.8 Hz, 1H) ppm;
^{13}C NMR (125 MHz, $CDCl_3$) δ 146.6, 143.6, 138.6, 135.3, 132.1, 129.7, 128.7, 128.6, 127.9, 127.74, 127.73, 127.0, 91.2, 83.1, 79.1, 73.5, 51.1, 46.9, 45.5, 36.7, 33.1, 32.8, 29.2, 26.7, 25.7, 21.7 ppm;
HRMS (ESI) calcd. for $C_{32}H_{36}ClNNaO_4S$ $[M+Na]^+$: 588.1946, found: 588.1948.

Synthesis of compound 3n

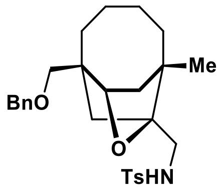

3n

3n (79 mg): colorless oil, 63% yield from **1n** (133 mg), purified by flash-column chromatography on silica gel (hexanes: EtOAc = 20: 1 ~ 5: 1);
R_f = 0.3 (silica gel, hexanes: EtOAc = 9: 1);
IR (film) λ_{max} 2924, 2858, 2359, 1605, 1508, 1456, 1352, 1190, 1016, 812, 748, 680, 511, 419 cm^{-1};
^{1}H NMR (500 MHz, $CDCl_3$) δ 7.73 (d, J = 8.4 Hz, 2H), 7.39 – 7.27 (m, 7H), 4.57 – 4.53 (m, 1H), 4.52 – 4.44 (m, 2H), 4.14 (d, J = 4.9 Hz, 1H), 3.21 (d, J = 8.2 Hz, 1H), 3.16 – 3.07 (m, 2H), 3.03 (d, J = 8.2 Hz, 1H), 2.42 (s, 3H), 2.31 (d, J = 12.5 Hz, 1H), 1.92 (d, J = 12.9 Hz, 1H), 1.76 – 1.65 (m, 3H), 1.57 – 1.40 (m, 4H), 1.34 – 1.29 (m, 1H), 1.21 (d, J = 13.0 Hz, 1H), 1.13 (dd, J = 12.6, 5.1 Hz, 1H), 0.84 (s, 3H) ppm;
^{13}C NMR (125 MHz, $CDCl_3$) δ 143.5, 138.7, 136.7, 129.9, 128.5, 127.7, 127.6, 127.2, 89.4, 83.0, 79.3, 73.4, 46.3, 43.3, 43.1, 38.6, 34.2, 33.4, 29.7, 29.6, 26.3, 25.8, 21.7 ppm;
HRMS (ESI) calcd. for $C_{27}H_{36}NO_4S$ $[M+H]^+$: 470.2360, found: 470.2361.

Synthesis of compound 3o

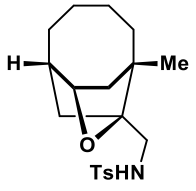

3o

3o (160 mg): colorless oil, 61% yield from **1o** (281 mg), purified by flash-column chromatography on silica gel (hexanes: EtOAc = 20: 1 ~ 5: 1);
R_f = 0.4 (silica gel, hexanes: EtOAc = 9: 1);

IR (film) λ_{max} 3271, 2924, 2859, 2359, 1597, 1506, 1456, 1339, 1163, 1092, 1036, 816, 662, 565, 552 cm^{-1};

^{1}H NMR (500 MHz, $CDCl_3$) δ 7.73 (d, *J* = 8.3 Hz, 2H), 7.31 (d, *J* = 8.0 Hz, 2H), 4.59 (dd, *J* = 8.5, 4.0 Hz, 1H), 4.25 (dd, *J* = 6.3, 4.8 Hz, 1H), 3.22 – 3.07 (m, 2H), 2.43 – 2.39 (m, 4H), 2.32 (d, *J* = 12.4 Hz, 1H), 1.85 (dd, *J* = 12.8, 2.7 Hz, 1H), 1.75 – 1.66 (m, 4H), 1.60 – 1.53 (m, 3H), 1.31 – 1.21 (m, 2H), 1.09 (ddd, *J* = 12.4, 4.9, 1.8 Hz, 1H), 0.84 (s, 3H) ppm;

^{13}C NMR (125 MHz, $CDCl_3$) δ 143.5, 136.8, 129.9, 127.2, 89.4, 81.6, 43.5, 42.7, 38.5, 36.2, 34.4, 29.9, 29.6, 26.4, 24.6, 24.2, 21.7 ppm;

HRMS (ESI) calcd. for $C_{19}H_{28}NO_3S$ $[M+H]^+$: 350.1784, found: 350.1782.

Synthesis of compound 3p

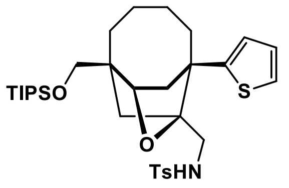

3p

3p (214 mg): white solid, 79% yield from **1p** (283 mg), purified by flash-column chromatography on silica gel (hexanes: EtOAc = 20: 1 ~ 5: 1);

R_f = 0.2 (silica gel, hexanes: EtOAc = 9: 1);

m.p. = 173 -174 ºC;

IR (film) λ_{max} 2940, 2854, 2359, 1597, 1508, 1458, 1331, 1240, 1163, 1096, 1067, 988, 881, 814, 689, 552 cm^{-1};

^{1}H NMR (500 MHz, $CDCl_3$) δ 7.51 (d, *J* = 8.1 Hz, 2H), 7.20 (d, *J* = 8.1 Hz, 2H), 7.04 (d, *J* = 4.8 Hz, 1H), 6.84 (dd, *J* = 4.9, 3.7 Hz, 1H), 6.77 (d, *J* = 3.1 Hz, 1H), 4.38 (d, *J* = 5.2 Hz, 1H), 4.28 (dd, *J* = 9.3, 3.5 Hz, 1H), 3.49 (d, *J* = 8.9 Hz, 1H), 3.28 (d, *J* = 8.9 Hz, 1H), 2.79 – 2.68 (m, 2H), 2.39 (s, 3H), 2.31 (dt, *J* = 14.9, 3.7 Hz, 1H), 2.22 – 2.16 (m, 1H), 2.12 – 2.01 (m, 3H), 1.94 – 1.87 (m, 1H), 1.83 (d, *J* = 14.1 Hz, 1H), 1.77 – 1.68 (m, 2H), 1.68 – 1.61 (m, 1H), 1.52 – 1.44 (m, 1H), 1.20 (d, *J* = 13.3 Hz, 1H), 1.11 – 1.04 (m, 21H) ppm;

^{13}C NMR (125 MHz, $CDCl_3$) δ 155.3, 143.3, 136.2, 129.7, 127.1, 126.8, 124.3, 123.4, 91.3, 82.4, 72.0, 50.8, 47.7, 45.2, 41.1, 34.1, 33.2, 29.2, 26.5, 25.7, 21.6, 18.3, 18.2, 12.1 ppm;

HRMS (ESI) calcd. for $C_{32}H_{50}NO_4S_2Si$ $[M+H]^+$: 604.2945, found: 604.2945.

Note: **3p** underwent removing TIPS group to give **3p'**. The structure of **3p'** was further confirmed by X-ray crystallographic analysis.

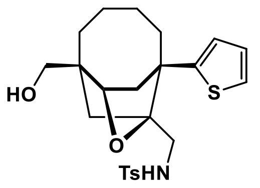

3p'

3p’ (12 mg): white solid, 85% yield from **3p** (19 mg), purified by flash-column chromatography on silica gel (hexanes: EtOAc = 5: 1 ~ 1: 2);

R*f* = 0.3 (silica gel, hexanes: EtOAc = 1: 1);

^{1}H NMR (500 MHz, $CDCl_3$) δ 7.52 (d, *J* = 8.3 Hz, 2H), 7.21 (d, *J* = 8.0 Hz, 2H), 7.05 (dd, *J* = 5.2, 1.2 Hz, 1H), 6.86 (dd, *J* = 5.1, 3.6 Hz, 1H), 6.78 (dd, *J* = 3.7, 1.2 Hz, 1H), 4.50 (dd, *J* = 9.3, 4.0 Hz, 1H), 4.31 (d, *J* = 5.2 Hz, 1H), 3.44 – 3.31 (m, 2H), 2.82 – 2.71 (m, 2H), 2.40 (s, 3H), 2.33 (dt, *J* = 15.1, 4.1 Hz, 1H), 2.25 (dd, *J* = 13.3, 4.0 Hz, 1H), 2.14 – 2.04 (m, 3H), 1.95 – 1.89 (m 1H), 1.86 – 1.81 (m, 1H), 1.78 – 1.65 (m, 3H), 1.61 – 1.56 (m, 1H), 1.49 – 1.42 (m, 2H) ppm;

^{13}C NMR (125 MHz, $CDCl_3$) δ 155.0, 143.4, 136.4, 129.7, 127.0, 126.9, 124.4, 123.6, 91.8, 83.0, 71.6, 50.6, 47.0, 45.0, 41.1, 34.1, 33.2, 28.6, 26.4, 25.6, 21.6 ppm;

HRMS (ESI) calcd. for $C_{23}H_{30}NO_4S_2$ $[M+H]^+$: 448.1611, found: 448.1615.

Synthesis of compound 3q

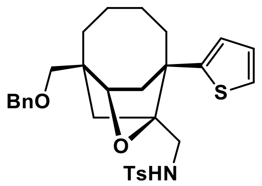

3q

3q (175 mg): colorless oil, 90% yield from **1q** (204 mg), purified by flash-column chromatography on silica gel (hexanes: EtOAc = 20: 1 ~ 5: 1);

R*f* = 0.6 (silica gel, hexanes: EtOAc = 4: 1);

IR (film) λ_{max} 3289, 2924, 2859, 2359, 1603, 1474, 1331, 1163, 1094, 1008, 814, 724, 696, 667, 550 cm^{-1};

^{1}H NMR (500 MHz, $CDCl_3$) δ 7.50 (d, *J* = 8.3 Hz, 2H), 7.38 – 7.29 (m, 5H), 7.20 (d, *J* = 8.0 Hz, 2H), 7.03 (dd, *J* = 5.2, 1.1 Hz, 1H), 6.84 (dd, *J* = 5.1, 3.6 Hz, 1H), 6.76 (dd, *J* = 3.6, 1.2 Hz, 1H), 4.55 – 4.46 (m, 2H), 4.36 (d, *J* = 5.2 Hz, 1H), 4.30 (dd, *J* = 9.4, 3.5 Hz, 1H), 3.26 (d, *J* = 8.3 Hz, 1H), 3.07 (d, *J* = 8.3 Hz, 1H), 2.78 – 2.69 (m, 2H), 2.39 (s, 3H), 2.31 (dt, *J* = 15.1, 4.1 Hz, 1H), 2.18 (dd, *J* = 13.1, 3.0 Hz, 1H), 2.11 – 2.01 (m, 3H), 1.93 – 1.87 (m, 1H), 1.84 – 1.78 (m, 1H), 1.77 – 1.69 (m, 2H), 1.65 – 1.57 (m, 1H), 1.56 – 1.49 (m, 1H), 1.30 (d, *J* = 13.3 Hz, 1H) ppm;

^{13}**C NMR** (125 MHz, $CDCl_3$) δ 155.1, 143.3, 138.6, 136.2, 129.7, 128.5, 127.73, 127.69, 127.0, 126.8, 124.3, 123.5, 91.3, 82.8, 79.2, 73.4, 50.7, 46.3, 45.0, 41.1, 34.0, 33.6, 29.4, 26.4, 25.7, 21.6 ppm;

HRMS (ESI) calcd. for $C_{30}H_{36}NO_4S_2$ $[M+H]^+$: 538.2080, found: 538.2078.

Synthesis of compound 3r

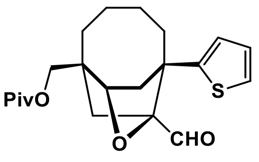

3r

3r (76 mg): colorless oil, 75% yield from **1r** (150 mg), purified by flash-column chromatography on silica gel (hexanes: EtOAc = 20: 1 ~ 5: 1);

R*f* = 0.3 (silica gel, hexanes: EtOAc = 4: 1);

IR (film) λ_{max} 2928, 2854, 2359, 1730, 1474, 1402, 1283, 1157, 1036, 1015, 811, 696, 553 cm^{-1};

^{1}**H NMR** (500 MHz, $CDCl_3$) δ 9.28 (s, 1H), 7.14 (dd, *J* = 5.1, 1.3 Hz, 1H), 6.93 (dd, *J* = 5.1, 3.6 Hz, 1H), 6.90 (dd, *J* = 3.6, 1.3 Hz, 1H), 4.46 (d, *J* = 5.1 Hz, 1H), 3.87 (s, 2H), 2.78 (d, *J* = 13.3 Hz, 1H), 2.44 (d, *J* = 13.2 Hz, 1H), 2.42 – 2.37 (m, 1H), 2.35 – 2.27 (m, 1H), 2.22 (dd, *J* = 13.3, 5.2 Hz, 1H), 2.00 – 1.93 (m, 1H), 1.90 – 1.85 (m, 1H), 1.77 – 1.65 (m, 2H), 1.62 – 1.49 (m, 2H), 1.20 (s, 9H), 1.15 (d, *J* = 13.2 Hz, 1H) ppm;

^{13}**C NMR** (125 MHz, $CDCl_3$) δ 201.0, 178.6, 153.3, 127.3, 124.8, 124.1, 95.9, 83.0, 71.8, 51.6, 45.0, 40.9, 39.1, 33.73, 33.70, 28.5, 27.3, 25.9, 25.5 ppm;

HRMS (ESI) calcd. for $C_{21}H_{28}NaO_4S$ $[M+Na]^+$: 399.1601, found: 399.1598.

Synthesis of compound 3s

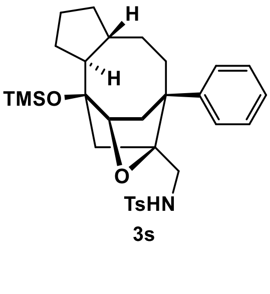

3s

3s (122 mg): white solid, 75% yield from **1s** (170 mg), purified by flash-column chromatography on silica gel (hexanes: EtOAc = 20: 1 ~ 5: 1);

R*f* = 0.3 (silica gel, hexanes: EtOAc = 4: 1);

m.p. = 179 - 180 ºC;

IR (film) λ_{max} 3244, 2945, 2851, 2359, 1653, 1506, 1387, 1188, 1096, 824, 712, 669, 554 cm^{-1};

^{1}H NMR (400 MHz, $CDCl_3$) δ 7.48 – 7.40 (m, 4H), 7.22 – 7.12 (m, 5H), 4.44 (d, *J* = 5.2 Hz, 1H), 4.17 (dd, *J* = 8.6, 4.9 Hz, 1H), 2.84 (d, *J* = 14.0 Hz, 1H), 2.67 (dd, *J* = 13.2, 8.6 Hz, 1H), 2.51 (d, *J* = 13.5 Hz, 1H), 2.45 – 2.36 (m, 5H), 2.32 – 2.10 (m, 5H), 2.01 – 1.94 (m, 1H), 1.86 – 1.75 (m, 2H), 1.70 – 1.60 (m, 3H), 1.32 – 1.14 (m, 2H), 0.17 (s, 9H) ppm;

^{13}C NMR (100 MHz, $CDCl_3$) δ 148.4, 143.3, 136.3, 129.7, 128.0, 127.1, 127.0, 126.2, 90.5, 86.4, 85.2, 56.0, 51.0, 49.3, 45.7, 43.3, 41.0, 34.0, 31.6, 30.7, 27.8, 21.6, 20.0, 2.7 ppm;

HRMS (ESI) calcd. for $C_{30}H_{41}NNaO_4SSi$ $[M+Na]^+$: 562.2418, found: 562.2416.

Note: The structure of **3s** was further confirmed by X-ray crystallographic analysis.

Synthesis of compound 3t

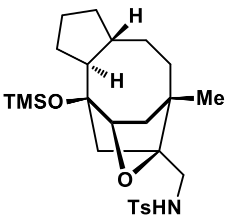

3t

3t (304 mg): white foam, 67% yield from **1t** (478 mg), purified by flash-column chromatography on silica gel (hexanes: EtOAc = 20: 1 ~ 5: 1);

R*f* = 0.5 (silica gel, hexanes: EtOAc = 4: 1);

IR (film) λ_{max} 3264, 2949, 2866, 2359, 1601, 1508, 1456, 1339, 1206, 1074, 1003, 839, 817, 732, 667, 563, 419 cm^{-1};

^{1}H NMR (500 MHz, $CDCl_3$) δ 7.78 – 7.70 (m, 2H), 7.32 (d, *J* = 7.7 Hz, 2H), 4.50 (dd, *J* = 9.5, 3.3 Hz, 1H), 4.18 (d, *J* = 4.9 Hz, 1H), 3.21 – 3.10 (m, 2H), 2.52 (d, *J* = 13.1 Hz, 1H), 2.43 (s, 3H), 2.30 (d, *J* = 13.0 Hz, 1H), 2.21 – 2.10 (m, 2H), 1.96 – 1.88 (m, 2H), 1.73 – 1.65 (m, 4H), 1.58 – 1.54 (m, 2H), 1.32 – 1.25 (m, 2H), 1.22 – 1.08 (m, 2H), 0.85 (s, 3H), 0.14 (s, 9H) ppm;

^{13}C NMR (125 MHz, $CDCl_3$) δ 143.6, 136.8, 129.9, 127.2, 88.4, 86.4, 85.1, 56.3, 48.3, 43.9, 43.5, 41.9, 41.0, 34.5, 33.9, 30.4, 30.1, 27.7, 21.7, 19.9, 2.7 ppm;

HRMS (ESI) calcd. for $C_{25}H_{39}NNaO_4SSi$ $[M+Na]^+$: 500.2261, found: 500.2262.

Note:**3t** underwent removing TMS group to give **3t'**. The structure of **3t'** was further confirmed by X-ray crystallographic analysis.

Synthesis of compound 3t'

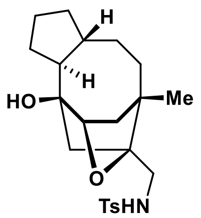

3t'

3t' (60 mg): white solid, 79% yield from **3t** (90 mg), purified by flash-column chromatography on silica gel (hexanes: EtOAc = 5: 1 ~ 1: 2);

R*f* = 0.3 (silica gel, hexanes: EtOAc = 1: 1);

¹H NMR (500 MHz, CD_3OD) δ 7.74 (d, *J* = 8.3 Hz, 2H), 7.38 (d, *J* = 8.0 Hz, 2H), 4.08 (d, *J* = 4.8 Hz, 1H), 3.30 (p, *J* = 1.6 Hz, 3H), 3.17 (d, *J* = 13.1 Hz, 1H), 2.97 (d, *J* = 13.1 Hz, 1H), 2.55 (d, *J* = 13.0 Hz, 1H), 2.47 (d, *J* = 13.0 Hz, 1H), 2.42 (s, 3H), 2.29 – 2.21 (m, 1H), 2.00 – 1.89 (m, 2H), 1.80 (d, *J* = 13.0 Hz, 1H), 1.78 – 1.65 (m, 4H), 1.60 – 1.54 (m, 1H), 1.33 – 1.28 (m, 2H), 1.25 – 1.17 (m, 1H), 1.16 – 1.08 (m, 1H), 0.90 (s, 3H) ppm;

¹³C NMR (125 MHz, CD_3OD) δ 144.8, 138.6, 130.8, 128.1, 89.7, 86.9, 82.6, 56.2, 50.7, 45.1, 44.8, 43.0, 42.4, 35.8, 34.6, 31.4, 30.0, 27.9, 21.4, 21.0 ppm;

HRMS (ESI) calcd. for $C_{22}H_{32}NO_4S$ $[M+H]^+$: 406.2047, found: 406.2045.

Synthesis of compound 3u'

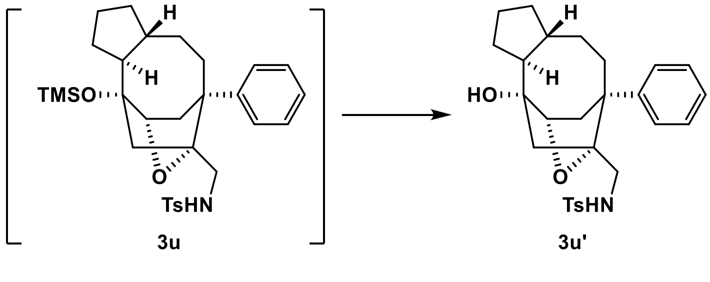

3u' (79 mg): colorless oil, 71% yield from **1u** (135 mg), purified by flash-column chromatography on silica gel (hexanes: EtOAc = 20: 1 ~ 3: 1);

R*f* = 0.3 (silica gel, hexanes: EtOAc = 4: 1);

IR (film) λ_{max} 2941, 2864, 2359, 1728, 1602, 1472, 1258, 1125, 1067, 998, 881, 773, 683, 572 cm^{-1};

¹H NMR (500 MHz, DMSO-d_6) δ 7.40 (d, *J* = 8.2 Hz, 2H), 7.29 – 7.27 (m, 3H), 7.20 (t, *J* = 7.6 Hz, 2H), 7.13 (d, *J* = 7.2 Hz, 1H), 7.10 (dt, *J* = 7.8, 3.2 Hz, 1H), 4.37 (s, 1H), 4.16 (d, *J* = 5.5 Hz, 1H), 2.45 (d, *J* = 13.4 Hz, 1H), 2.37 – 2.25 (m, 6H), 2.14 – 1.97 (m, 4H), 1.88 – 1.78 (m, 3H), 1.74 – 1.65 (m, 3H), 1.55 – 1.47 (m, 3H), 1.35 – 1.26 (m, 2H) ppm;

¹³C NMR (125 MHz, DMSO-d_6) δ 147.8, 142.4, 137.2, 129.4, 127.4, 127.3, 126.3, 125.6, 91.3, 88.2, 80.0, 49.8, 48.1, 45.4, 44.6, 40.5, 34.4, 34.2, 32.9, 30.7, 30.3, 20.9, 19.7 ppm;

HRMS (ESI) calcd. for $C_{27}H_{34}NO_4S$ $[M+H]^+$: 468.2203, found: 468.2201.

Note: The TMS group in target compound **3u** is sensitive to acid. When it was purified by silica gel (or dissolved in $CDCl_3$), **3u** underwent removing TMS group to give **3u'**.

Synthesis of compound 3v

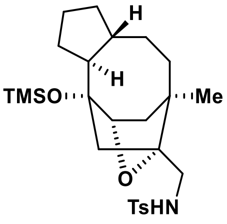

3v

3v (110 mg): white foam, 66% yield from **1v** (176 mg), purified by flash-column chromatography on silica gel (hexanes: EtOAc = 20: 1 ~ 5: 1);

R*f* = 0.5 (silica gel, hexanes: EtOAc = 4: 1);

IR (film) λ_{max} 3668, 3476, 3200, 2949, 1601, 1321, 1254, 1067, 991, 850, 658 cm^{-1};

^{1}H NMR (500 MHz, $CDCl_3$) δ 7.74 (d, *J* = 8.2 Hz, 2H), 7.32 (d, *J* = 8.0 Hz, 2H), 4.50 (dd, *J* = 8.6, 4.3 Hz, 1H), 4.15 (d, *J* = 5.3 Hz, 1H), 3.19 – 3.10 (m, 2H), 2.43 (s, 3H), 2.38 (d, *J* = 13.3 Hz, 1H), 2.31 (d, *J* = 12.8 Hz, 1H), 1.86 – 1.63 (m, 6H), 1.53 – 1.40 (m, 3H), 1.35 – 1.24 (m, 4H), 1.11 (dd, *J* = 12.9, 5.4 Hz, 1H), 0.85 (s, 3H), 0.12 (s, 9H) ppm;

^{13}C NMR (125 MHz, $CDCl_3$) δ 143.6, 136.8, 129.9, 127.2, 90.0, 88.9, 84.7, 50.1, 45.6, 43.4, 42.2, 41.6, 37.3, 35.0, 34.3, 31.9, 30.9, 29.7, 21.7, 19.8, 2.5 ppm;

HRMS (ESI) calcd. for $C_{25}H_{39}NNaO_4SSi$ $[M+Na]^+$: 500.2261, found: 500.2262.

Synthesis of compound 3w

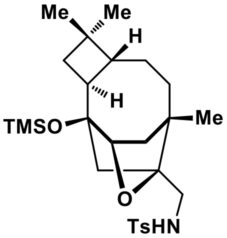

3w

3w (81 mg): white foam, 75% yield from **1w** (114 mg), purified by flash-column chromatography on silica gel (hexanes: EtOAc = 20: 1 ~ 5: 1);

R*f* = 0.3 (silica gel, hexanes: EtOAc = 5: 1);

IR (film) λ_{max} 2955, 1456, 1332, 1250, 1161, 841 cm^{-1};

^{1}H NMR (500 MHz, $CDCl_3$) δ 7.92 – 7.70 (m, 2H), 7.45 – 7.30 (m, 2H), 4.85 (s, 1H), 4.60 – 4.48 (m, 1H), 4.18 (d, *J* = 5.2 Hz, 1H), 3.20 – 3.08 (m, 2H), 2.49 (d, *J* = 13.1 Hz, 1H), 2.43 (s, 3H), 2.27 (d, *J* = 12.8 Hz, 1H), 2.23 – 2.17 (m, 1H), 1.82 (d, *J* = 13.2 Hz, 1H), 1.62 (dd, *J* = 12.4, 2.3 Hz, 1H), 1.53 (dd, *J* = 9.8, 7.8 Hz, 1H), 1.44 – 1.36 (m, 3H), 1.33 – 1.23 (m, 2H), 1.12 (dd, *J* = 12.9, 5.3 Hz, 1H), 1.01 (d, *J* = 7.3 Hz, 6H), 0.85 (s, 3H), 0.11 (s, 9H) ppm;

^{13}C NMR (125 MHz, $CDCl_3$) δ 143.6, 136.9, 129.9, 127.2, 90.4, 88.8, 83.1, 51.9, 43.6, 43.5, 42.5, 42.1, 38.4, 37.7, 34.5, 33.9, 30.9, 30.2, 24.7, 21.7, 20.6, 2.4 ppm;

HRMS (ESI) calcd. for $C_{26}H_{42}O_4NSSi$ $[M+H]^+$: 492.2598, found: 492.2598.

Note: **3w** underwent removing TMS group to give **3w'**. The structure of **3w'** was further confirmed by X-ray crystallographic analysis.

Synthesis of compound 3w'

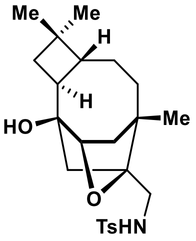

3w'

3w' (12 mg): white solid, 86% yield from **3w** (16 mg), purified by flash-column chromatography on silica gel (hexanes: EtOAc = 5: 1 ~ 1: 2);

R*f* = 0.2 (silica gel, hexanes: EtOAc = 1: 1);

^{1}H NMR (500 MHz, $CDCl_3$) δ 7.72 (d, *J* = 8.3 Hz, 2H), 7.31 (d, *J* = 8.0 Hz, 2H), 5.04 (d, *J* = 8.2 Hz, 1H), 4.23 (d, *J* = 4.5 Hz, 1H), 3.15 – 3.09 (m, 2H), 2.97 (d, *J* = 13.5 Hz, 1H), 2.58 – 2.45 (m, 2H), 2.43 (s, 3H), 2.31 – 2.21 (m, 1H), 2.18 – 2.16 (m, 2H), 1.80 – 1.69 (m, 4H), 1.44 – 1.36 (m, 3H), 1.05 (s, 3H), 0.98 (s, 3H), 0.87 (s, 3H) ppm;

^{13}C NMR (125 MHz, $CDCl_3$) δ 143.6, 136.9, 129.9, 127.1, 88.4, 87.4, 81.1, 52.4, 46.4, 45.9, 44.8, 43.2, 43.0, 38.0, 37.9, 33.6, 30.5, 30.4, 27.2, 21.7, 18.4 ppm;

HRMS (ESI) calcd. for $C_{23}H_{34}NO_4S$ $[M+H]^+$: 420.2203, found: 420.2205.

Synthesis of compound 3x

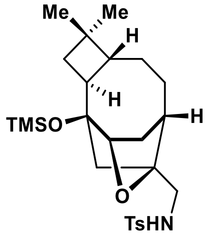

3x

3x (62 mg): white foam, 65% yield from **1x** (100 mg), purified by flash-column chromatography on silica gel (hexanes: EtOAc = 20: 1 ~ 5: 1);

R*f* = 0.3 (silica gel, hexanes: EtOAc = 5: 1);

IR (film) λ_{max} 2954, 1458, 1330, 1251, 1159, 840 cm^{-1};

^{1}H NMR (500 MHz, CD_2Cl_2) δ 7.72 (d, *J* = 8.3 Hz, 2H), 7.34 (d, *J* = 8.0 Hz, 2H), 4.79 (t, *J* = 6.3 Hz, 1H), 3.81 (d, *J* = 5.0 Hz, 1H), 3.24 – 3.15 (m, 2H), 2.43 (s, 3H), 2.24 – 2.12 (m, 2H), 2.06 (d, *J* = 12.7 Hz, 1H), 1.86 – 1.79 (m, 1H), 1.77 – 1.71 (m, 2H), 1.66 – 1.59 (m, 2H), 1.52 – 1.46 (m, 2H), 1.44 – 1.38 (m, 1H), 1.26 – 1.21 (m, 2H), 1.00 (s, 3H), 0.97 (s, 3H), 0.05 (s, 9H) ppm;

^{13}C NMR (125 MHz, CD_2Cl_2) δ 144.2, 137.3, 130.3, 127.5, 90.6, 87.6, 87.0, 47.4, 45.5, 43.7, 40.3, 38.0, 36.6, 35.3, 32.0, 30.6, 22.2, 22.0, 21.8, 20.5, 2.6 ppm;

HRMS (ESI) calcd. for $C_{25}H_{40}NO_4SSi$ $[M+H]^+$: 478.2442, found: 478.2446.

Note: **3x** underwent removing TMS group to give **3x'**. The structure of **3x'** was further confirmed by X-ray crystallographic analysis.

Synthesis of compound 3x'

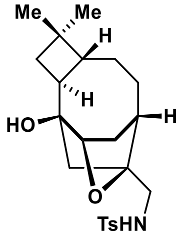

3x'

3x' (13 mg): white solid, 85% yield from **3x** (18 mg), purified by flash-column chromatography on silica gel (hexanes: EtOAc = 5: 1 ~ 1: 2);

R*f* = 0.2 (silica gel, hexanes: EtOAc = 1: 1);

^{1}H NMR (500 MHz, $CDCl_3$) δ 7.71 (d, *J* = 8.3 Hz, 2H), 7.30 (d, *J* = 8.1 Hz, 2H), 5.36 – 5.28 (m, 1H), 4.23 (d, *J* = 4.1 Hz, 1H), 3.17 – 3.09 (m, 2H), 2.76 (d, *J* = 13.3 Hz, 1H), 2.54 (t, *J* = 12.4 Hz, 1H), 2.49 – 2.44 (m, 1H), 2.42 (s, 3H), 2.28 – 2.23 (m, 1H), 2.08 (d, *J* = 12.5 Hz, 1H), 1.93 (dd, *J* =

12.5, 2.1 Hz, 1H), 1.85 – 1.76 (m, 3H), 1.68 (dd, J = 9.1, 6.9 Hz, 1H), 1.60 (qd, J = 7.5, 2.6 Hz, 1H), 1.49 – 1.44 (m, 1H), 1.36 – 1.32 (m, 1H), 1.04 (s, 3H), 0.97 (s, 3H) ppm;

^{13}C NMR (125 MHz, $CDCl_3$) δ 143.5, 136.9, 129.9, 127.1, 88.0, 87.7, 81.8, 53.3, 46.8, 46.6, 45.5, 39.8, 37.92, 37.85, 35.3, 30.4, 26.6, 23.9, 21.7, 18.4 ppm;

HRMS (ESI) calcd. for $C_{22}H_{32}NO_4S$ $[M+H]^+$: 406.2047, found: 406.2043.

Synthesis of compound 3y

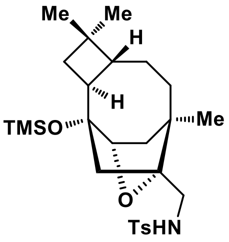

3y

3y (76 mg): white foam, 70% yield from **1y** (114 mg), purified by flash-column chromatography on silica gel (hexanes: EtOAc = 20: 1 ~ 5: 1);

R*f* = 0.4 (silica gel, hexanes: EtOAc = 5: 1);

IR (film) λ_{max} 2958, 2889, 1460, 1330, 1251, 1159, 1099, 1072, 840 cm^{-1};

^{1}H NMR (500 MHz, $CDCl_3$) δ 7.73 (d, J = 8.3 Hz, 2H), 7.31 (d, J = 8.5, 2H), 4.52 (dd, J = 9.4, 3.2 Hz, 1H), 4.19 (d, J = 4.6 Hz, 1H), 3.17 – 3.07 (m, 2H), 2.95 (d, J = 13.4 Hz, 1H), 2.51 – 2.45 (m, 1H), 2.43 (s, 3H), 2.41 – 2.36 (m, 1H), 2.30 (d, J = 12.7 Hz, 1H), 1.98 (d, J = 12.6 Hz, 1H), 1.76 – 1.70 (m, 1H), 1.64 – 1.62 (m, 1H), 1.69 – 1.58 (m, 1H), 1.51 – 1.45 (m, 1H), 1.36 – 1.25 (m, 3H), 1.03 (s, 3H), 0.95 (s, 3H), 0.86 (s, 3H), 0.13 (s, 9H) ppm;

^{13}C NMR (125 MHz, $CDCl_3$) δ 143.6, 136.8, 129.9, 127.2, 87.9, 87.4, 83.7, 52.2, 48.1, 46.3, 44.8, 43.4, 43.3, 39.2, 37.3, 33.6, 30.8, 30.4, 27.2, 21.7, 18.4, 2.6 ppm;

HRMS (ESI) calcd. for $C_{26}H_{42}O_4NSSi$ $[M+H]^+$: 492.2598, found: 492.2598.

Synthesis of compound 3z'

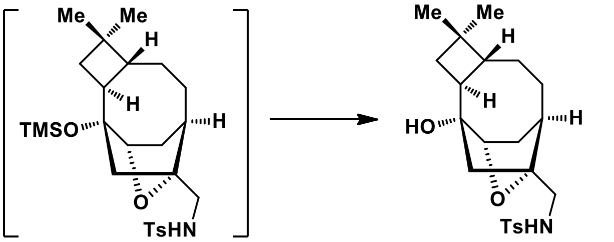

3z 3z'

3z' (48 mg): colorless oil, 66% yield from **1z** (91 mg), purified by flash-column chromatography on silica gel (hexanes: EtOAc = 5: 1 ~ 1: 1);

R*f* = 0.3 (silica gel, hexanes: EtOAc = 1: 1);

IR (film) λ_{max} 3661, 3494, 3237, 2949, 2863, 1598, 1317, 1154, 1093, 984, 840, 658, 540 cm^{-1};

^{1}H NMR (500 MHz, $CDCl_3$) δ 7.73 (d, *J* = 8.4 Hz, 2H), 7.31 (d, *J* = 8.0 Hz, 2H), 5.22 – 5.15 (m, 1H), 3.81 (d, *J* = 5.0 Hz, 1H), 3.23 (d, *J* = 6.3 Hz, 2H), 2.43 (s, 3H), 2.29 – 2.17 (m, 2H), 2.14 (d, *J* = 12.9 Hz, 1H), 1.89 – 1.83 (m, 1H), 1.77 – 1.72 (m, 3H), 1.70 – 1.64 (m, 2H), 1.55 – 1.49 (m, 2H), 1.46 – 1.41 (m, 1H), 1.28 – 1.24 (m, 2H), 1.02 (s, 3H), 1.00 (s, 3H) ppm;

^{13}C NMR (125 MHz, $CDCl_3$) δ 143.6, 136.9, 129.9, 127.2, 90.8, 87.3, 84.3, 46.6, 45.4, 43.8, 39.5, 36.1, 35.7, 35.3, 31.8, 30.5, 22.0, 21.7, 21.6, 20.2 ppm;

HRMS (ESI) calcd. for $C_{22}H_{32}NO_4S$ [M+H]$^+$: 406.2047, found: 406.2043.

Note: The TMS group in target compound **3z** is sensitive to acid. When it was purified by silica gel (or dissolved in $CDCl_3$), **3z** underwent removing TMS group to give **3z'**.

Synthesis of compound 3aa

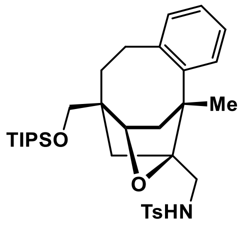

3aa

3aa (32 mg): colorless oil, 55% yield from **1aa** (61 mg), purified by flash-column chromatography on silica gel (hexanes: EtOAc = 10: 1 ~ 2: 1);

R*f* = 0.5 (silica gel, hexanes: EtOAc = 4: 1);

IR (film) λ_{max} 3273, 2941, 2864, 2359, 1603, 1513, 1458, 1337, 1254, 1163, 1096, 1069, 1011, 881, 814, 679, 554 cm^{-1};

^{1}H NMR (500 MHz, $CDCl_3$) δ 7.71 (d, *J* = 8.3 Hz, 2H), 7.30 (d, *J* = 8.0 Hz, 3H), 7.23 – 7.18 (m, 1H), 7.13 (d, *J* = 4.0 Hz, 2H), 4.45 (d, *J* = 4.6 Hz, 1H), 3.79 – 3.68 (m, 1H), 3.55 (d, *J* = 13.7 Hz, 1H), 3.45 (d, *J* = 9.2 Hz, 1H), 3.24 (dd, *J* = 12.4, 2.9 Hz, 1H), 3.15 – 3.09 (m, 2H), 2.88 (dd, *J* = 16.3, 7.9 Hz, 1H), 2.43 (s, 3H), 2.33 (dd, *J* = 14.8, 7.2 Hz, 1H), 1.87 – 1.75 (m, 2H), 1.62 (d, *J* = 4.7 Hz, 1H), 1.40 (s, 3H), 1.08 – 1.04 (m, 22H), 0.96 (d, *J* = 12.6 Hz, 1H) ppm;

^{13}C NMR (125 MHz, $CDCl_3$) δ 143.6, 142.7, 140.1, 136.6, 133.6, 129.9, 127.4, 127.3, 127.2, 127.1, 89.5, 82.4, 70.8, 50.2, 48.8, 42.9, 42.0, 37.9, 36.1, 30.7, 26.6, 21.7, 18.2, 12.1 ppm;

HRMS (ESI) calcd. for $C_{33}H_{50}NO_4SSi$ [M+H]$^+$: 584.3224, found: 584.3225.

Synthesis of compound 3ab

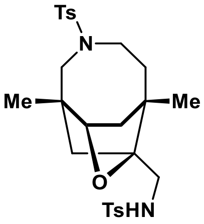

3ab

3ab (26 mg): colorless oil, 50% yield from **1ab** (54 mg), purified by flash-column chromatography on silica gel (hexanes: EtOAc = 5: 1 ~ 1: 1);

R$_f$ = 0.5 (silica gel, hexanes: EtOAc = 1: 1);

IR (film) λ_{max} 3449, 2253, 2127, 1653, 1051, 1026, 1005, 826, 764, 635 cm^{-1};

^{1}H NMR (400 MHz, DMSO-*d*$_6$) δ 7.73 (d, *J* = 8.2 Hz, 2H), 7.64 (d, *J* = 8.3 Hz, 2H), 7.39 (dd, *J* = 7.9, 5.1 Hz, 4H), 7.32 (dd, *J* = 7.8, 5.6 Hz, 1H), 3.75 (d, *J* = 4.6 Hz, 1H), 3.69 (dt, *J* = 15.2, 4.0 Hz, 1H), 3.27 (d, *J* = 15.0 Hz, 1H), 3.17 – 3.09 (m, 2H), 3.00 – 2.91 (m, 2H), 2.55 (d, *J* = 12.9 Hz, 1H), 2.44 – 2.37 (m, 7H), 1.95 – 1.85 (m, 1H), 1.40 – 1.32 (m, 1H), 1.22 (d, *J* = 12.8 Hz, 1H), 1.09 (dd, *J* = 12.9, 4.7 Hz, 1H), 1.01 (s, 3H), 0.91 (s, 3H) ppm;

^{13}C NMR (125 MHz, DMSO-*d*$_6$) δ 142.9, 142.6, 137.4, 129.9, 129.6, 126.7, 126.3, 89.4, 85.7, 52.3, 45.6, 44.0, 43.2, 42.2, 41.7, 35.2, 29.4, 29.1, 21.02, 20.98 ppm;

HRMS (ESI) calcd. for $C_{26}H_{35}N_2O_5S_2$ [M+H]$^+$: 519.1982, found: 519.1982.

Note: The ^{1}H-NMR spectra of **3ab** detected at 25 ºC using $CDCl_3$ (CD_2Cl_2, C_6D_6, DMSO-*d*$_6$) as the solvent.The methylene (CH_2) group at eight-membered ring showed broad singlet signals at 3.7 - 1.4 ppm due to presence of the Ts group at this ring. As the ^{1}H-NMR spectra of **3ab** detected in DMSO-*d*$_6$ at 85 ºC, the splitting in the resonance signal at 3.7 -1.4 ppm of the methylene groups were observed.

Synthesis of compounds 3ac and 3ad

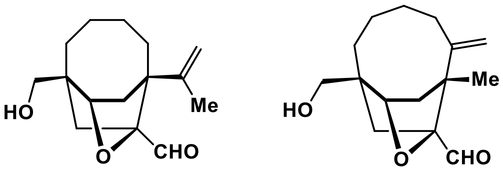

3ac 3ad

3ac (49 mg, 46%): colorless oil, **3ad** (24 mg, 22%): colorless oil, total 68% yield from **1ac** (252 mg), purified by flash-column chromatography on silica gel (hexanes: EtOAc = 5: 1 ~ 1: 1);

Data for **3ac**

R*f* = 0.6 (silica gel, hexanes: EtOAc = 1: 1);

IR (film) λ_{max} 3431, 2924, 2860, 1726, 1626, 1473, 1261, 1041, 910, 802, 723 cm^{-1};

^{1}H NMR (500 MHz, $CDCl_3$) δ 9.74 (s, 1H), 4.93 (d, *J* = 22.8 Hz, 2H), 4.38 (d, *J* = 5.2 Hz, 1H), 3.43 – 3.32 (m, 2H), 2.32 (dd, *J* = 22.6, 13.1 Hz, 2H), 2.10 – 2.00 (m, 2H), 1.93 – 1.81 (m, 4H), 1.79 (s, 3H), 1.62 – 1.52 (m, 3H), 1.48 – 1.40 (m, 1H), 1.15 (d, *J* = 13.0 Hz, 1H) ppm;

^{13}C NMR (125 MHz, $CDCl_3$) δ 201.2, 150.0, 114.3, 95.7, 83.6, 71.5, 53.6, 46.6, 34.0, 33.3, 31.5, 28.1, 26.3, 25.7, 23.1 ppm;

HRMS (ESI) calcd. for $C_{15}H_{22}NaO_3$ [M+Na]$^+$: 273.1461, found: 273.1462.

Data for **3ad**

R*f* = 0.6 (silica gel, hexanes: EtOAc = 1: 1);

IR (film) λ_{max} 3442, 2934, 2854, 1728, 1626, 1489, 1256, 1033, 921, 813, 736 cm^{-1};

^{1}H NMR (500 MHz, $CDCl_3$) δ 10.02 (s, 1H), 5.16 (s, 1H), 4.96 (s, 1H), 4.36 (d, *J* = 5.4 Hz, 1H), 3.42 – 3.34 (m, 1H), 3.23 (d, *J* = 10.2 Hz, 1H), 2.75 (d, *J* = 13.0 Hz, 1H), 2.51 – 2.44 (m, 1H), 2.40 (d, *J* = 12.9 Hz, 1H), 2.35 (dd, *J* = 7.8, 5.3 Hz, 1H), 2.07 – 1.94 (m, 2H), 1.89 – 1.71 (m, 4H), 1.66 – 1.60 (m, 2H), 1.30 – 1.25 (m, 4H) ppm;

^{13}C NMR (125 MHz, $CDCl_3$) δ 201.7, 154.5, 117.7, 94.9, 82.4, 72.1, 52.9, 48.3, 41.2, 38.7, 34.4, 32.3, 30.6, 29.4, 25.3 ppm;

HRMS (ESI) calcd. for $C_{15}H_{23}O_3$ [M+H]$^+$: 251.1642, found: 251.1642.

Note: Treatment of **1ac** with $Rh_2(Oct)_4$ followed by a wet base Al_2O_3 gave the corresponding aldehydes, which were hard to separate and then underwent removing TIPS group by TBAF in one pot, to give **3ac** and **3ad** in yields of 46% and 22%, respectively.

Synthesis of compound 3ae

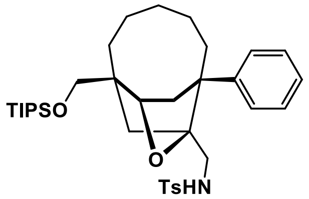

3ae

3ae (28 mg): white foam, 25% yield from **1ae** (115 mg), purified by flash-column chromatography on silica gel (hexanes: EtOAc = 20: 1 ~ 5: 1);

R*f* = 0.3 (silica gel, hexanes: EtOAc = 10: 1);

IR (film) λ_{max} 3259, 2941, 1598, 1463, 1332, 1165, 1068, 881, 1001, 812, 661, 551 cm^{-1};

^{1}H NMR (500 MHz, $CDCl_3$) δ 7.47 (d, *J* = 8.3 Hz, 2H), 7.34 (d, *J* = 7.3 Hz, 2H), 7.22 – 7.13 (m, 4H), 7.10 (t, *J* = 7.2 Hz, 1H), 4.42 (d, *J* = 6.0 Hz, 1H), 4.07 (dd, *J* = 9.8, 3.1 Hz, 1H), 3.51 (d, *J* = 8.9 Hz, 1H), 3.14 (d, *J* = 8.9 Hz, 1H), 2.78 – 2.68 (m, 2H), 2.42 – 2.29 (m, 6H), 2.19 (d, *J* = 13.5 Hz, 1H), 2.07 (dd, *J* = 12.9, 3.1 Hz, 2H), 1.97 – 1.77 (m, 7H), 1.29 (d, *J* = 9.1 Hz, 1H), 1.15 – 1.02 (m, 21H) ppm;

^{13}C NMR (125 MHz, $CDCl_3$) δ 148.7, 143.3, 136.3, 129.6, 127.8, 127.7, 127.0, 126.0, 90.6, 81.0, 71.4, 52.9, 50.3, 45.4, 38.1, 35.1, 34.2, 31.2, 30.0, 28.7, 27.2, 21.6, 18.3, 12.2 ppm;

HRMS (ESI) calcd. for $C_{35}H_{54}NO_4SSi$ $[M+H]^+$: 612.3537, found: 612.3538.

Synthesis of compound 3af

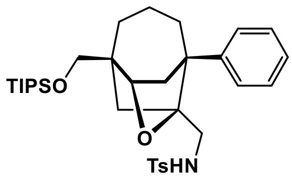

3af

3af (94 mg): white solid, 81% yield from **1af** (122 mg), purified by flash-column chromatography on silica gel (hexanes: EtOAc = 20: 1 ~ 5: 1);

R*f* = 0.3 (silica gel, hexanes: EtOAc = 9: 1);

m.p. = 153 - 155 ºC;

IR (film) λ_{max} 3271, 2941, 2866, 2359, 1599, 1503, 1464, 1337, 1165, 1096, 972, 883, 812, 662, 552 cm^{-1};

^{1}H NMR (500 MHz, $CDCl_3$) δ 7.38 (d, *J* = 8.2 Hz, 2H), 7.26 – 7.24 (m, 2H), 7.20 (t, *J* = 7.5 Hz, 2H), 7.17 – 7.12 (m, 3H), 4.49 (d, *J* = 4.3 Hz, 1H), 4.16 (dd, *J* = 8.7, 4.3 Hz, 1H), 3.56 (d, *J* = 9.3 Hz, 1H), 3.41 (d, *J* = 9.3 Hz, 1H), 2.59 (dd, *J* = 13.0, 8.7 Hz, 1H), 2.51 – 2.42 (m, 1H), 2.37 (s, 3H), 2.36 – 2.22 (m, 4H), 2.07 – 1.98 (m, 3H), 1.91 (d, *J* = 12.2 Hz, 1H), 1.83 – 1.76 (m, 1H), 1.37 (d, *J* = 12.2 Hz, 1H), 1.13 – 0.99 (m, 21H) ppm;

^{13}C NMR (125 MHz, $CDCl_3$) δ 146.0, 143.2, 136.1, 129.6, 128.1, 127.04, 126.97, 126.3, 91.5, 83.5, 70.4, 50.9, 48.5, 45.7, 42.9, 39.3, 33.1, 29.6, 21.6, 20.2, 18.2, 12.1 ppm;

HRMS (ESI) calcd. for $C_{33}H_{50}NO_4SSi$ $[M+H]^+$: 584.3224, found: 584.3224.

Note: The structure of **3af** was further confirmed by X-ray crystallographic analysis.

Synthesis of compound 3ag

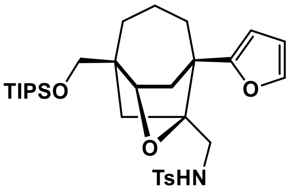

3ag

3ag (93 mg): white solid, 81% yield from **1ag** (120 mg), purified by flash-column chromatography on silica gel (hexanes: EtOAc = 20: 1 ~ 5: 1);

R*f* = 0.5 (silica gel, hexanes: EtOAc = 4: 1);

m.p. = 145 - 146 ºC;

IR (film) λ_{max} 3250, 2940, 1590, 1500, 1489, 1237, 1180, 1090, 881, 670, 551 cm^{-1};

^{1}H NMR (500 MHz, $CDCl_3$) δ 7.62 – 7.54 (m, 2H), 7.26 – 7.20 (m, 3H), 6.22 (dd, *J* = 3.3, 1.9 Hz, 1H), 5.99 (d, *J* = 3.2 Hz, 1H), 4.48 (dd, *J* = 8.5, 4.6 Hz, 1H), 4.41 (d, *J* = 4.1 Hz, 1H), 3.55 (d, *J* = 9.3 Hz, 1H), 3.41 (d, *J* = 9.3 Hz, 1H), 2.87 (dd, *J* = 12.9, 8.5 Hz, 1H), 2.47 (dd, *J* = 13.0, 4.6 Hz, 1H), 2.42 – 2.33 (m, 4H), 2.25 (d, *J* = 12.0 Hz, 1H), 2.12 (dd, *J* = 12.0, 4.2 Hz, 1H), 2.08 – 2.03 (m, 1H), 2.02 – 1.92 (m, 4H), 1.80 – 1.72 (m, 1H), 1.39 (d, *J* = 12.2 Hz, 1H), 1.09 – 1.03 (m, 21H) ppm;

^{13}C NMR (125 MHz, $CDCl_3$) δ 159.2, 143.4, 141.6, 136.5, 129.7, 127.1, 110.2, 105.9, 92.1, 83.0, 70.4, 48.6, 48.3, 44.8, 40.5, 39.0, 31.5, 29.5, 21.6, 19.8, 18.2, 12.1 ppm;

HRMS (ESI) calcd. for $C_{31}H_{48}NO_5SSi$ $[M+H]^+$: 574.3017, found: 574.3019.

Note: The structure of **3ag** was further confirmed by X-ray crystallographic analysis.

Synthesis of compound 3ah

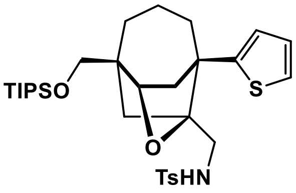

3ah

3ah (103 mg): white solid, 83% yield from **1ah** (129 mg), purified by flash-column chromatography on silica gel (hexanes: EtOAc = 20: 1 ~ 5: 1);

R*f* = 0.5 (silica gel, hexanes: EtOAc = 4: 1);

m.p. = 151 - 152 ºC;

IR (film) λ_{max} 3271, 2941, 2864, 2359, 1713, 1603, 1541, 1456, 1339, 1206, 1094, 881, 816, 689, 563, 419 cm^{-1};

^{1}H NMR (500 MHz, $CDCl_3$) δ 7.50 (d, *J* = 8.0 Hz, 2H), 7.20 (d, *J* = 8.0 Hz, 2H), 7.09 (d, *J* = 5.2 Hz, 1H), 6.89 (dd, *J* = 5.1, 3.5 Hz, 1H), 6.74 (d, *J* = 3.6 Hz, 1H), 4.44 (d, *J* = 4.2 Hz, 1H), 4.30 (dd, *J* =

9.0, 3.8 Hz, 1H), 3.56 (d, J = 9.3 Hz, 1H), 3.42 (d, J = 9.3 Hz, 1H), 2.83 (dd, J = 13.0, 9.0 Hz, 1H), 2.54 – 2.45 (m, 2H), 2.42 – 2.36 (m, 4H), 2.31 – 2.24 (m, 1H), 2.11 (dd, J = 12.3, 4.3 Hz, 1H), 2.07 – 1.95 (m, 3H), 1.91 (d, J = 12.2 Hz, 1H), 1.81 – 1.79 (m, 1H), 1.44 – 1.41 (m, 1H), 1.11 – 1.03 (m, 21H) ppm;

^{13}C NMR (125 MHz, $CDCl_3$) δ 152.1, 143.3, 136.3, 129.7, 127.03, 126.98, 124.1, 123.6, 91.5, 83.2, 70.4, 50.3, 48.2, 45.6, 45.1, 38.8, 34.6, 29.6, 21.6, 20.2, 18.2, 12.1 ppm;

HRMS (ESI) calcd. for $C_{31}H_{48}NO_4S_2Si$ $[M+H]^+$: 590.2789, found: 590.2794.

Note: The structure of **3ah** was further confirmed by X-ray crystallographic analysis.

Synthesis of compound 3ai

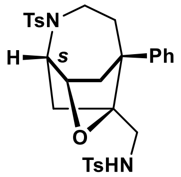

3ai

3ai (133 mg): colorless oil, 80% yield from **1ai** (174 mg), purified by flash-column chromatography on silica gel (hexanes: EtOAc = 5: 1 ~ 1: 2);

R$_f$ = 0.5 (silica gel, hexanes: EtOAc = 1: 1);

$[\alpha]_D^{25}$ = -7.50 (c = 0.5, CH_2Cl_2);

IR (film) λ_{max} 3635, 3269, 2929, 2882, 1728, 1598, 1495, 1448, 1334, 1162, 1092, 1043, 979, 909, 816, 667, 547 cm^{-1};

^{1}H NMR (500 MHz, $CDCl_3$) δ 7.68 (d, J = 8.2 Hz, 2H), 7.35 (dd, J = 15.4, 8.2 Hz, 4H), 7.25 – 7.18 (m, 5H), 7.16 (d, J = 8.1 Hz, 2H), 4.84 – 4.81 (m, 1H), 4.62 – 4.58 (m, 1H), 4.06 (dd, J = 8.4, 5.1 Hz, 1H), 3.98 (ddd, J = 12.7, 10.2, 6.8 Hz, 1H), 3.46 – 3.40 (m, 1H), 2.61 (dd, J = 13.5, 8.5 Hz, 1H), 2.47 (s, 1H), 2.45 (s, 3H), 2.41 (dd, J = 7.8, 5.8 Hz, 1H), 2.38 (s, 3H), 2.26 (dd, J = 12.5, 3.9 Hz, 1H), 1.88 – 1.86 (m,1H), 1.26 – 1.24 (m, 2H) ppm;

^{13}C NMR (125 MHz, $CDCl_3$) δ 144.1, 143.8, 143.6, 136.2, 135.7, 130.1, 129.7, 128.5, 127.1, 126.9, 126.8, 126.7, 90.7, 81.8, 56.7, 50.3, 45.0, 43.4, 43.0, 36.0, 32.2, 21.7, 21.6 ppm;

HRMS (ESI) calcd. for $C_{29}H_{33}N_2O_5S_2$ $[M+H]^+$: 553.1825, found: 553.1823.

Synthesis of compound 3aj

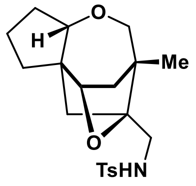

3aj

3aj (31 mg): colorless oil, 50% yield from **1aj** (65 mg), purified by flash-column chromatography on silica gel (hexanes: EtOAc = 10: 1 ~ 5: 1);

R*f* = 0.5 (silica gel, hexanes: EtOAc = 4: 1);

IR (film) λ_{max} 3257, 2954, 2870, 1450, 1332, 1163, 1093, 813, 659, 551 cm^{-1};

^{1}H NMR (500 MHz, $CDCl_3$) δ 7.74 (d, *J* = 8.3 Hz, 2H), 7.32 (d, *J* = 8.0 Hz, 2H), 4.57 (dd, *J* = 9.1, 3.4 Hz, 1H), 4.29 (t, *J* = 7.3 Hz, 1H), 4.11 (d, *J* = 3.5 Hz, 1H), 3.75 (d, *J* = 11.6 Hz, 1H), 3.67 (d, *J* = 11.6 Hz, 1H), 3.23 (dd, *J* = 12.7, 9.1 Hz, 1H), 3.14 (dd, *J* = 12.7, 3.4 Hz, 1H), 2.46 (d, *J* = 11.1 Hz, 1H), 2.43 (s, 3H), 2.33 (d, *J* = 11.7 Hz, 1H), 2.00 – 1.93 (m, 1H), 1.82 – 1.76 (m, 1H), 1.71 – 1.65 (m, 1H), 1.55 – 1.40 (m, 4H), 1.36 (d, *J* = 11.1 Hz, 1H), 0.85 (s, 3H) ppm;

^{13}C NMR (125 MHz, $CDCl_3$) δ 143.7, 136.7, 129.9, 127.2, 88.3, 84.8, 82.1, 75.0, 54.3, 46.2, 45.3, 43.8, 42.5, 37.8, 33.5, 21.9, 21.7, 20.9 ppm;

HRMS (ESI) calcd. for $C_{20}H_{28}NO_4S$ $[M+H]^+$: 378.1734, found: 378.1737.

4. Asymmetric Total Synthesis of Nakafuran-8

4.1 Comparison of the NMR Data of Natural and Synthetic Nakafuran-8.

Supplementary Table 2. ^{1}H NMR data of natural and synthetic nakafuran-8

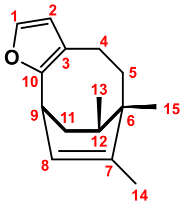

Position	Natural product[12]<br>CDCl$_3$, 220 MHz<br>δ H (m, *J* Hz)	Product by Yamamoto[13]<br>CDCl$_3$, 300 MHz<br>δ H (m, *J* Hz)	Product by us<br>CDCl$_3$, 500 MHz<br>δ H (m, *J* Hz)
1	7.13 (d, 1.5, 1H)	7.13 (d, l.5, 1H)	7.13 (d, 1.7, 1H)
2	6.07 (dd, 1.5, 1.0, 1H)	6.07 (d, 1.5, 1H)	6.07 (d, 1.6, 1H)
3	-	-	-
4	2.45 (ddd, 14.7, 3.5, 1H)<br>2.25 (ddd, 14.4, 4.0, 1H)	2.43 (dddd, 15.0, 10.8, 6.8, 1H)<br>2.27 (ddd, 15.0, 3.6, 3.6, 1H)	2.44 (ddd, 14.9, 12.0, 6.0, 1H)<br>2.26 (dt, 14.9, 3.7, 1H)
5	1.9 (qadd, 7.6, 6.0, 1H)<br>1.24 (ddd, 14.6, 4.0, 1H)	1.90 - 1.65 (m, 1H)<br>1.26 (ddd, 11.8, 11.8, 4.0, 1H)	1.90 - 1.76 (m, 1H)<br>1.26 (ddd, 14.6, 3.8, 1H)
6	-	-	-
7	-	-	-
8	5.95 (dd, 7.0, 1.0, 1H)	5.98 (brd, 7.0, 1H)	5.96 (d, 7.5, 1H)
9	3.45 (dddd, 7.4, 3.5, 1.0, 1H)	3.46 (ddd, 7.0, 3.5, 3.5, 1H)	3.45 (dt, 7.1, 3.4, 1H)
10	-		
11	1.8 (ddd, 14.7, 4.0, 1H)<br>1.28 (ddd, 14.6, 3.5, 1H)	1.90 - 1.65 (m, 2H)	1.9 - 1.76 (m, 2H)
12	2.0 (ddd, 14.4, 3.5, 1H)	1.90 - 1.65 (m, 1H)	1.9 - 1.76 (m, 1H)
13	0.89 (d, 7.0, 3H)	0.89 (d, 7.0, 3H)	0.89 (d, 7.1, 3H)
14	1.75 (d, 1.0, 3H)	1.70 (d, 1.2, 3H)	1.71 (d, 1.2, 3H)
15	1.06 (s, 3H)	1.06 (s, 3H)	1.06 (s, 3H)

Supplementary Table 3. ^{13}C NMR data of natural and synthetic nakafuran-8

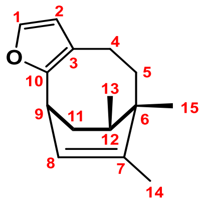

Position	Natural product[12] CDCl$_3$, 55 MHz	Product by Yamamoto[13] CDCl$_3$, 75 MHz	Product by us CDCl$_3$, 125 MHz
1	138.4	138.3	138.4
2	113.8	113.6	113.8
3	118.5	118.4	118.5
4	23.4	23.1	23.2
5	48.3	47.9	48.1
6	38.8	40.8	40.9
7	141.1	141.0	141.2
8	124.6	124.4	124.5
9	35.0	34.7	34.8
10	151.0	151.0	151.0
11	39.3	39.0	39.1
12	36.8	36.5	36.6
13	19.0	18.7	18.8
14	20.5	20.2	20.4
15	24.6	24.3	24.4

4.2 Experimental Details

Synthesis of compound S29

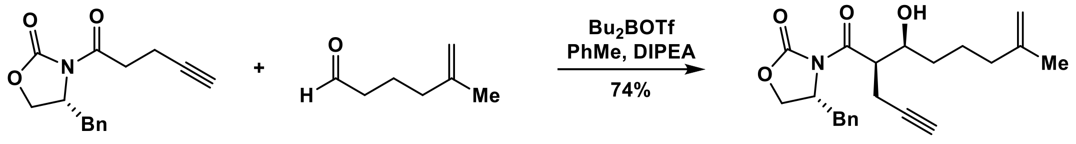

To a stirred solution of compound **4**[14] (13.0 g, 50.6 mmol, 1.0 equiv.) in toluene (253 mL, 0.2 M/L) at -20 ºC was added Bu_2BOTf (1.0 M in DCM, 55.7 mL, 55.7 mmol, 1.1 equiv.), the mixture was stirred for 15 minutes at -20 ºC. Then DIPEA (10.0 mL, 60.7 mmol, 1.2 equiv.) was added into the mixture at -20 ºC, and stirring was continued for 3 hours at 0 ºC. The solution of freshly prepared ketone **5**[15] (6.8 g, 60.7 mmol, 1.2 equiv.) in toluene (20 mL) was added to the mixture at -78 ºC. After 2 hours, the reaction mixture was warmed to -20 ºC for 1 hour. The reaction was quenched with pH 7 buffer (100 mL) and the aqueous layer was extracted with EtOAc (100 mL × 3). The combined organic extracts were washed with saturated brine (100 mL), dried over Na_2SO_4, filtered and concentrated under reduced pressure. The residue was purified by flash-column chromatography on silica gel (hexanes: EtOAc = 4: 1) to afford β-hydroxyl ketone **S29** (13.8 g, 74% yield) as a colorless oil.

R_f = 0.5 (silica gel, hexanes: EtOAc = 2: 1);

$[\alpha]_D^{25}$ = -39.67 (*c* = 3.0, CH_2Cl_2);

IR (film) λ_{max} 3522, 3291, 2938, 2120, 1771, 1694, 1385, 1209, 1101, 889, 748, 704, 642, 507 cm^{-1};

^{1}H NMR (500 MHz, $CDCl_3$) δ 7.34 (t, *J* = 7.2 Hz, 2H), 7.30 – 7.26 (m, 1H), 7.26 – 7.22 (m, 2H), 4.77 – 4.71 (m, 1H), 4.69 (d, *J* = 14.8 Hz, 2H), 4.25 – 4.16 (m, 3H), 4.05 – 3.98 (m, 1H), 3.35 (dd, *J* = 13.4, 3.2 Hz, 1H), 2.83 – 2.73 (m, 2H), 2.61 (ddd, *J* = 17.0, 5.1, 2.7 Hz, 1H), 2.48 (s, 1H), 2.09 – 1.99 (m, 3H), 1.71 (s, 3H), 1.67 – 1.63 (m, 1H), 1.55 – 1.45 (m, 3H) ppm;

^{13}C NMR (125 MHz, $CDCl_3$) δ 174.1, 153.5, 145.6, 135.3, 129.6, 129.1, 127.5, 110.3, 81.6, 71.3, 70.4, 66.3, 55.6, 47.0, 38.0, 37.6, 33.8, 24.0, 22.4, 16.4 ppm;

HRMS (ESI) calcd. for $C_{22}H_{28}NO_4$ $[M+H]^+$: 370.2013, found: 370.2009.

Synthesis of compound 6

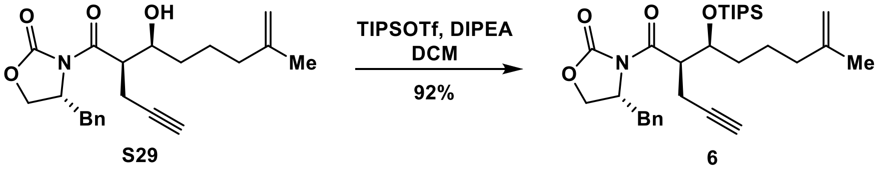

To a stirred solution of above β-hydroxyl ketone **S29** (30.0 g, 81.3 mmol, 1.0 equiv.) in DCM (813 mL, 0.1 M/L) at 0 ºC was added DIPEA (36.6 mL, 203.2 mmol, 2.5 equiv.) and TIPSOTf (43.7 mL, 162.6 mmol, 2.0 equiv.). Then the mixture was stirred at 25 ºC for 6 hours. The reaction was quenched by saturated aqueous NH_4Cl (200 mL) at 0 ºC. The aqueous phase was extracted by DCM (200 mL × 3) and the combined organic extracts were washed with saturated brine (200 mL), dried over Na_2SO_4, filtered and concentrated under reduced pressure. The residue was purified by flash-column chromatography on silica gel (hexanes: EtOAc = 20: 1) to afford **6** (39.1 g, 92% yield) as a colorless oil.

$\mathbf{R}_f$ = 0.4 (silica gel, hexanes: EtOAc = 10: 1);

$[\alpha]_D^{25}$ = -4.00 (*c* = 2.0, CH_2Cl_2);

IR (film) λ_{max} 3460, 2943, 2866, 1788, 1699, 1464, 1385, 1248, 1196, 1107, 1063, 1013, 995, 918, 883, 806, 677 cm^{-1};

^{1}H NMR (500 MHz, $CDCl_3$) δ 7.33 (t, *J* = 7.2 Hz, 2H), 7.28 (d, *J* = 7.3 Hz, 1H), 7.26 – 7.24 (m, 2H), 4.68 (d, *J* = 16.8 Hz, 2H), 4.66 – 4.61 (m, 1H), 4.29 – 4.25 (m, 1H), 4.22 – 4.10 (m, 3H), 3.29 (dd, *J* = 13.5, 2.9 Hz, 1H), 2.94 – 2.82 (m, 2H), 2.53 (ddd, *J* = 16.9, 4.1, 2.8 Hz, 1H), 2.01 (t, *J* = 7.4 Hz, 2H), 1.97 (t, *J* = 2.6 Hz, 1H), 1.70 (s, 3H), 1.66 – 1.59 (m, 2H), 1.55 – 1.48 (m, 2H), 1.04 (s, 21H) ppm;

^{13}C NMR (125 MHz, $CDCl_3$) δ 172.6, 153.2, 145.5, 135.3, 129.7, 129.1, 127.5, 110.4, 82.9, 72.5, 69.5, 66.2, 55.9, 47.9, 38.0, 37.6, 35.6, 22.8, 22.4, 18.3, 18.2, 15.3, 13.1 ppm;

HRMS (ESI) calcd. for $C_{31}H_{48}NO_4Si$ $[M+H]^+$: 526.3347, found: 526.3343.

Synthesis of compound S30

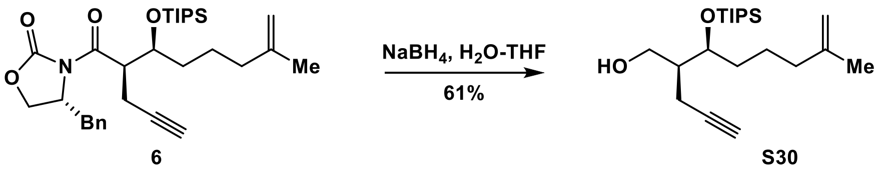

To a stirred solution of compound **6** (20.0 g, 38.1 mmol, 1.0 equiv.) in THF (1088 mL, 0.035 M/L) and H_2O (163 mL) at 0 ºC was added $NaBH_4$ (8.6 g, 228.6 mmol, 6.0 equiv.). Then the mixture was stirred at 25 ºC for 3 days. The reaction was quenched by saturated aqueous NH_4Cl (200 mL) at 0 ºC.

The aqueous phase was extracted by EtOAc (200 mL × 3) and the combined organic extracts were washed with saturated brine (200 mL), dried over Na_2SO_4, filtered and concentrated under reduced pressure. The residue was purified by flash-column chromatography on silica gel (hexanes: EtOAc = 10: 1) to afford alcohol **S30** (8.2 g, 61% yield) as a colorless oil.

R*f* = 0.3 (silica gel, hexanes: EtOAc = 10: 1);

$[\alpha]_D^{25}$ = +1.50 (c = 2.0, CH_2Cl_2);

IR (film) λ_{max} 3443, 3312, 2941, 2866, 2118, 1649, 1462, 1373, 1249, 1086, 883, 677, 635 cm^{-1};

^{1}H NMR (500 MHz, $CDCl_3$) δ 4.68 (d, J = 21.0 Hz, 2H), 4.17 – 4.11 (m, 1H), 3.84 – 3.72 (m, 2H), 2.29 – 2.17 (m, 2H), 2.09 – 1.99 (m, 3H), 1.98 (t, J = 2.7 Hz, 1H), 1.70 (s, 3H), 1.58 – 1.42 (m, 4H), 1.07 (s, 21H) ppm;

^{13}C NMR (125 MHz, $CDCl_3$) δ 145.6, 110.3, 83.3, 74.0, 69.7, 64.1, 44.3, 38.0, 33.2, 24.0, 22.4, 18.4, 18.3, 16.3, 13.0 ppm;

HRMS (ESI) calcd. for $C_{21}H_{41}O_2Si$ $[M+H]^+$: 353.2870, found: 353.2865.

Note: According to the above procedure, a total of 24.6 g of **S30** was prepared readily after 3 simple parallel operations.

Synthesis of compound 7

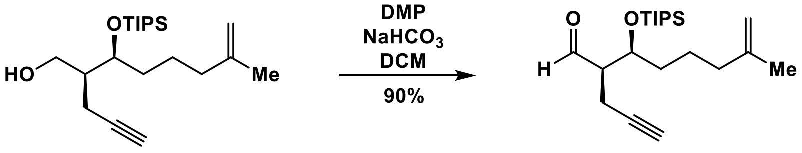

S30 7

To a stirred solution of **S30** (17.6 g, 50.0 mmol, 1.0 equiv.) in DCM (500 mL, 0.1 M/L) at 0 ºC was added $NaHCO_3$ (21.0 g, 250.0 mmol, 5.0 equiv.) and DMP (42.4 g, 100.0 mmol, 2.0 equiv.). When the reaction was stirred for 2 hours at 25 ºC, it was quenched by saturated aqueous $NaHCO_3$ (200 mL) and saturated aqueous $Na_2S_2O_3$ (100 mL) at 0 ºC. The mixture was stirred at 25 ºC until a clear solution was obtained. The aqueous phase was extracted by DCM (200 mL × 3) and the combined organic extracts were washed with saturated brine (200 mL), dried over Na_2SO_4, filtered and concentrated under reduced pressure. The residue was purified by flash-column chromatography on silica gel (hexanes: EtOAc = 50: 1) to afford **7** (15.8 g, 90% yield) as a colorless oil.

R*f* = 0.4 (silica gel, hexanes: EtOAc = 30: 1);

$[\alpha]_D^{25}$ = -8.00 (c = 1.0, CH_2Cl_2);

IR (film) λ_{max} 3314, 2943, 2868, 1724, 1464, 1369, 1261, 1101, 1021, 883, 733, 679, 463 cm^{-1};

^{1}H NMR (500 MHz, $CDCl_3$) δ 9.88 (s, 1H), 4.69 (d, *J* = 26.2 Hz, 2H), 4.47 – 4.41 (m, 1H), 2.75 – 2.68 (m, 2H), 2.32 (ddd, *J* = 19.3, 9.3, 2.7 Hz, 1H), 2.06 – 1.99 (m, 2H), 1.98 (t, *J* = 2.6 Hz, 1H), 1.70 (s, 3H), 1.64 – 1.59 (m, 1H), 1.53 – 1.44 (m, 3H), 1.06 (s, 21H) ppm;
^{13}C NMR (125 MHz, $CDCl_3$) δ 203.6, 145.3, 110.5, 82.2, 71.9, 70.0, 55.6, 37.8, 34.3, 23.7, 22.4, 18.32, 18.28, 13.2, 12.8 ppm;
HRMS (ESI) calcd. for $C_{21}H_{39}O_2Si$ $[M+H]^+$: 351.2714, found: 351.2710.

Synthesis of compound 8

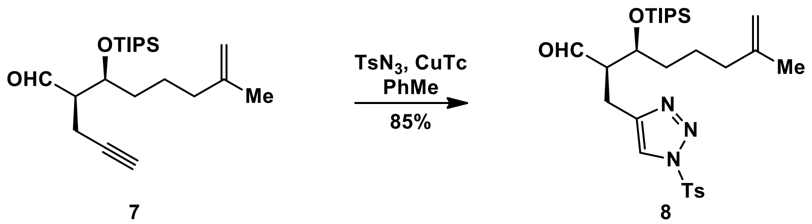

To a stirred solution of compound **7** (14.0 g, 40.0 mmol, 1.0 equiv.) in toluene (400 mL, 0.1 M/L) at 25 ºC was added cuprous thiophene-2-carboxylate (763 mg, 4.0 mmol, 0.1 equiv.) and tosyl azide (11.8 g, 60.0 mmol, 1.5 equiv.). Then the mixture was stirred at 25 ºC for 4 hours. The reaction was concentrated directly under reduced pressure and the residue was purified by flash-column chromatography on silica gel (hexanes: EtOAc = 15: 1) to afford **8** (18.6 g, 85% yield) as a colorless oil.
R_f = 0.2 (silica gel, hexanes: EtOAc = 10: 1);
$[\alpha]_D^{25}$ = -20.53 (*c* = 1.9, CH_2Cl_2);
IR (film) λ_{max} 3152, 3073, 2943, 2866, 2725, 1726, 1649, 1595, 1462, 1395, 1296, 1194, 1092, 883, 814, 754, 671, 584, 542 cm^{-1};
^{1}H NMR (500 MHz, $CDCl_3$) δ 9.83 (s, 1H), 7.97 (d, *J* = 8.4 Hz, 2H), 7.92 (s, 1H), 7.37 (d, *J* = 8.0 Hz, 2H), 4.67 (d, *J* = 33.2 Hz, 2H), 4.38 – 4.32 (m, 1H), 3.18 (dd, *J* = 15.3, 8.7 Hz, 1H), 3.00 – 2.93 (m, 1H), 2.96 (dt, *J* = 8.3, 3.8 Hz, 1H), 2.77 (dd, *J* = 15.3, 4.4 Hz, 1H), 2.44 (s, 3H), 1.97 (t, *J* = 7.4 Hz, 2H), 1.68 (s, 3H), 1.62 – 1.54 (m, 2H), 1.53 – 1.43(m, 2H), 1.02 (s, 21H) ppm;
^{13}C NMR (125 MHz, $CDCl_3$) δ 203.5, 147.2, 146.0, 145.1, 133.2, 130.5, 128.7, 121.7, 110.5, 72.1, 56.4, 37.6, 34.4, 23.5, 22.3, 21.9, 19.3, 18.22, 18.18, 12.8 ppm;
HRMS (ESI) calcd. for $C_{28}H_{46}N_3O_4SSi$ $[M+H]^+$: 548.2973, found: 548.2972.

Synthesis of compound 10

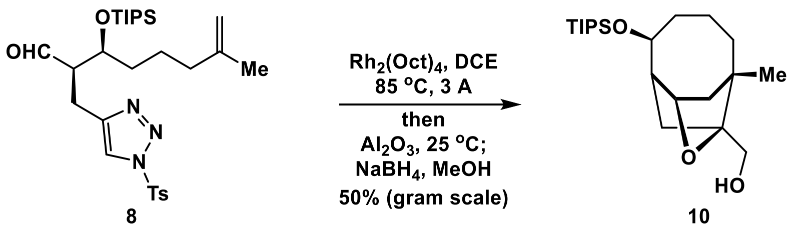

An oven-dried tube was charged with triazole **8** (3.83 g, 7.0 mmol, 1.0 equiv.), 3Å MS (1.6 g), and $Rh_2(Oct)_4$ (272 mg, 0.35 mmol, 0.05 equiv.). The tube was evacuated and backfilled with argon (repeated three times) and DCE (140 mL, 0.05 M/L) was added via syringe. After stirred at 85 ºC for 3 hours, the reaction mixture was cooled to 25 ºC, and Al_2O_3 (35.0 g) was added. The reaction mixture was stirred for 1.5 hours and was cooled to 0 ºC. Then MeOH (70 mL), $NaBH_4$ (583 mg, 15.4 mmol, 2.2 equiv.) was added and the mixture was stirred for 3 hours at 0 ºC. The reaction was quenched with saturated aqueous NH_4Cl (100 mL) and extracted with EtOAc (100 mL × 3). The combined extracts were washed with saturated brine (50 mL), dried by Na_2SO_4, filtered and concentrated in vacuum. The residue was purified by a flash-column chromatography on silica gel (hexanes: EtOAc = 10: 1) to afford **10** (1.3 g, 50% yield) as a colorless oil.

R*f* = 0.4 (silica gel, hexanes: EtOAc = 4: 1);

$[\alpha]_D^{25}$ = -11.25 (*c* = 1.0, CH_2Cl_2);

IR (film) λ_{max} 3311, 2941, 2866, 1471, 1114, 881, 677 cm^{-1};

^{1}H NMR (500 MHz, $CDCl_3$) δ 4.48 – 4.41 (m, 2H), 3.98 (dd, *J* = 12.0, 3.6 Hz, 1H), 3.64 (dd, *J* = 12.0, 8.5 Hz, 1H), 2.76 (d, *J* = 12.2 Hz, 2H), 1.95 – 1.83 (m, 3H), 1.83 – 1.76 (m, 1H), 1.76 – 1.69 (m, 1H), 1.66 – 1.57 (m, 2H), 1.43 – 1.36 (m, 1H), 1.29 (ddd, *J* = 12.3, 5.0, 1.6 Hz, 1H), 1.06 (s, 21H), 0.95 (s, 3H) ppm;

^{13}C NMR (125 MHz, $CDCl_3$) δ 91.2, 80.7, 70.3, 62.1, 43.4, 41.9, 41.3, 34.8, 34.1, 31.5, 29.5, 21.5, 18.28, 18.25, 12.5 ppm;

HRMS (ESI) calcd. for $C_{21}H_{41}O_3Si$ $[M+H]^+$: 369.2819, found: 369.2816.

Synthesis of compound S31

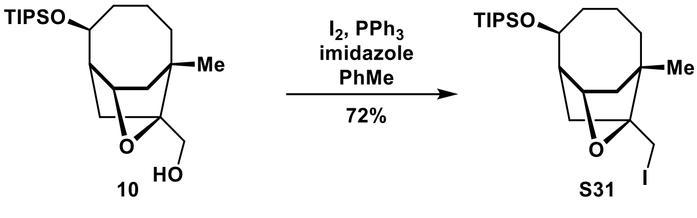

To a solution of compound **10** (1.47 g, 4.0 mmol, 1.0 equiv.) in dry toluene (40 mL, 0.1 M/L) was

added Ph_3P (1.57 g, 6.0 mmol, 1.5 equiv.), imidazole (953 mg, 14.0 mmol, 3.5 equiv.), and I_2 (2.03 g, 8.0 mmol, 2.0 equiv.). The mixture was refluxed for 5 hours. The reaction mixture was concentrated directly under reduced pressure. The residue was purified by column chromatography (hexanes: EtOAc = 50: 1) to give product iodo compound **S31** (1.38 g, 72% yield) as a colorless oil.

R_f = 0.3 (silica gel, hexanes: EtOAc = 30: 1);

$[\alpha]_D^{25}$ = -22.86 (*c* = 1.4, CH_2Cl_2);

IR (film) λ_{max} 2941, 2866, 1462, 1112, 1060, 881, 678 cm^{-1};

^{1}H NMR (500 MHz, $CDCl_3$) δ 4.50 – 4.38 (m, 2H), 3.54 (d, *J* = 10.5 Hz, 1H), 3.35 (d, *J* = 10.5 Hz, 1H), 2.86 (d, *J* = 12.3 Hz, 1H), 2.84 – 2.77 (m, 1H), 1.95 (dd, *J* = 12.5, 2.8 Hz, 1H), 1.92 – 1.77 (m, 4H), 1.73 (t, *J* = 12.3 Hz, 1H), 1.62 – 1.56 (m, 1H), 1.46 – 1.40 (m, 1H), 1.34 (ddd, *J* = 12.2, 5.0, 1.6 Hz, 1H), 1.05 (s, 21H), 1.03 (s, 3H) ppm;

^{13}C NMR (125 MHz, $CDCl_3$) δ 88.3, 80.5, 70.1, 43.8, 42.4, 42.3, 37.3, 35.4, 34.1, 30.3, 21.5, 18.3, 18.2, 12.4, 7.1 ppm;

HRMS (ESI) calcd. for $C_{21}H_{40}IO_2Si$ $[M+H]^+$: 479.1837, found: 479.1834.

Synthesis of compound 11

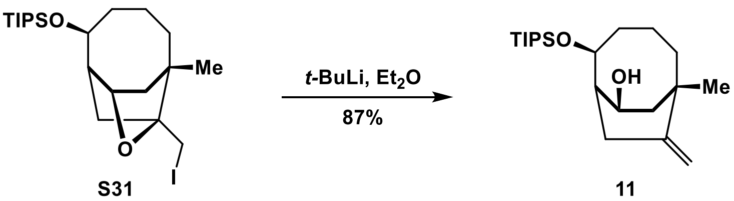

S31 11

To a solution of compound **S31** (2.4 g, 5.0 mmol, 1.0 equiv.) in Et_2O (50 mL, 0.1 M/L) was added *t*-BuLi (1.3 M in hexane, 11.5 mL, 15.0 mmol, 3.0 equiv.) dropwise at -78 ºC through a syringe and the mixture was stirred for another 30 minutes. TLC showed the consumption of the starting material. The reaction was quenched by saturated aqueous NH_4Cl (50 mL). The aqueous phase was extracted by Et_2O (50 mL × 3) and the combined organic extracts were washed with saturated brine (50 mL), dried over Na_2SO_4, filtered and concentrated under reduced pressure. The residue was purified by flash-column chromatography on silica gel (hexanes: EtOAc = 10: 1) to give **11** (1.53 g, 87% yield) as a colorless oil.

R_f = 0.4 (silica gel, hexanes: EtOAc = 9: 1);

$[\alpha]_D^{25}$ = +3.33 (*c* = 0.3, CH_2Cl_2);

IR (film) λ_{max} 3253, 2941, 2864, 1458, 1012, 875, 669 cm^{-1};

^{1}H NMR (500 MHz, $CDCl_3$) δ 4.85 (dd, *J* = 11.0, 2.2 Hz, 2H), 4.77 (td, *J* = 8.7, 3.5 Hz, 1H), 3.97 – 3.91 (m, 1H), 2.74 (ddt, *J* = 16.0, 5.9, 2.9 Hz, 1H), 2.10 – 2.01 (m, 2H), 1.91 – 1.80 (m, 2H), 1.73 – 1.63 (m, 2H), 1.54 – 1.43 (m, 4H), 1.35 (d, *J* = 3.7 Hz, 1H), 1.09 (s, 3H), 1.06 (s, 21H) ppm;
^{13}C NMR (125 MHz, $CDCl_3$) δ 155.2, 108.6, 72.7, 64.3, 49.2, 46.0, 43.2, 38.0, 33.5, 32.1, 31.3, 19.9, 18.4, 18.3, 12.6 ppm;
HRMS (ESI) calcd. for $C_{21}H_{41}O_2Si$ $[M+H]^+$: 353.2870, found: 353.2865.

Synthesis of compound 11'

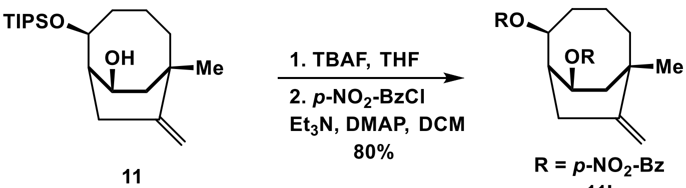

To a stirred solution of compounds **11** (25 mg, 0.07 mmol, 1.0 equiv.) in THF (3.5 mL, 0.02 M/L) was added TBAF (1.0 M in THF, 0.14 mL, 0.14 mmol, 2.0 equiv.) at 25 ºC. Then the reaction was stirred for 2 hours at 25 ºC. The solvent was evaporated under reduced pressure to afford the crude product, which was used for the next step without further purification.

A solution of the above crude product in anhydrous DCM (7 mL, 0.1 M/L) was added Et_3N (0.015 mL, 0.11 mmol, 1.5 equiv.) and DMAP (8 mg, 0.07 mmol, 1.0 equiv.) at 0 ºC. After 5 minutes, *p*-nitrobenzoyl chloride (26 mg, 0.14 mmol, 2.0 equiv.) was added to the reaction. The mixture was warmed to 25 ºC and stirred for 30 minutes. The reaction was quenched by addition of saturated NH_4Cl (5 mL). Then the aqueous layer was extracted with DCM (5 mL × 3). The combined organic layers were washed with saturated brine (5 mL) and dried over Na_2SO_4. The solution was filtered and concentrated. The residue was purified by flash-column chromatography on silica gel (hexanes: EtOAc = 5: 1) to afford **11'** (28 mg, 80% yield for 2 steps), as a white solid for X-ray analysis.
R_f= 0.6 (silica gel, hexanes: EtOAc = 10: 1);
m.p. = 168 - 169 ºC;
IR (film) λ_{max} 3421, 2925, 2875, 1721, 1609, 1536, 1440, 1345, 1280, 1104, 999, 942, 870, 714 cm^{-1};
^{1}H NMR (500 MHz, $CDCl_3$) δ 8.33 – 8.28 (m, 4H), 8.23 (d, *J* = 10.0 Hz, 2H), 8.11 (d, *J* = 10.0 Hz, 2H), 6.14 (t, *J* = 7.5 Hz, 1H), 5.31 – 5.25 (m, 1H), 5.04 (d, *J* = 2.7 Hz, 2H), 2.92 (ddt, *J* = 16.2, 5.8, 2.9 Hz, 1H), 2.62 – 2.56 (m, 1H), 2.36 (d, *J* = 20.0 Hz, 1H), 2.18 – 2.09 (m, 2H), 2.01 – 1.94 (m, 1H), 1.91 – 1.73 (m, 3H), 1.72 – 1.61 (m, 2H), 1.20 (s, 3H) ppm;

^{13}C NMR (125 MHz, $CDCl_3$) δ 164.0, 163.9, 152.7, 150.60, 150.58, 136.4, 135.9, 131.0, 130.8, 123.63, 123.59, 110.6, 76.0, 70.2, 45.7, 42.3, 39.5, 37.8, 33.5, 31.3, 29.0, 20.7 ppm;
HRMS (ESI) calcd. for $C_{26}H_{27}N_2O_8$ $[M+H]^+$: 495.1762, found: 495.1757.

Synthesis of compound S32

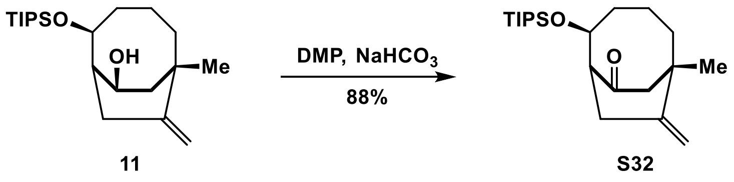

To a stirred solution of compound **11** (2.11 g, 6.0 mmol, 1.0 equiv.) in DCM (60 mL, 0.1 M/L) at 0 ºC was added $NaHCO_3$ (2.52 g, 30.0 mmol, 5.0 equiv.) and DMP (5.09 g, 12.0 mmol, 2.0 equiv.). Then the mixture was stirred for 2 hours at 25 ºC. TLC showed the consumption of the starting material. The reaction was quenched by saturated aqueous $Na_2S_2O_3$ (20 mL) and saturated aqueous $NaHCO_3$ (20 mL) at 0 ºC. The mixture was stirred at 25 ºC until a clear solution was obtained, then extracted by DCM (50 mL × 3) and the combined organic extracts were washed with saturated brine (50 mL), dried over Na_2SO_4, filtered and concentrated under reduced pressure. The residue was purified by flash-column chromatography on silica gel (hexanes: EtOAc = 100: 1) to afford ketone **S32** (1.85 g, 88% yield) as a colorless oil.
R*f* = 0.3 (silica gel, hexanes: EtOAc = 30: 1);
$[\alpha]_D^{25}$ = -56.12 (*c* = 2.9, CH_2Cl_2);
IR (film) λ_{max} 2941, 2866, 1712, 1460, 1132, 883, 682 cm^{-1};
^{1}H NMR (500 MHz, $CDCl_3$) δ 5.02 (dd, *J* = 8.0, 1.8 Hz, 2H), 4.11 – 4.02 (m, 1H), 2.79 – 2.70 (m, 2H), 2.38 (d, *J* = 15.1 Hz, 1H), 2.31 (dd, *J* = 17.8, 0.7 Hz, 1H), 2.18 (d, *J* = 17.8 Hz, 1H), 2.00 – 1.92 (m, 1H), 1.80 – 1.72 (m, 1H), 1.63 – 1.44 (m, 4H), 1.15 (s, 3H), 1.07 (s, 21H) ppm;
^{13}C NMR (125 MHz, $CDCl_3$) δ 211.7, 152.5, 110.3, 75.2, 52.2, 50.2, 47.1, 38.5, 33.5, 32.5, 32.2, 19.3, 18.3, 18.2, 12.6 ppm;
HRMS (ESI) calcd. for $C_{21}H_{39}O_2Si$ $[M+H]^+$: 351.2714, found: 351.2710.

Synthesis of compound S33

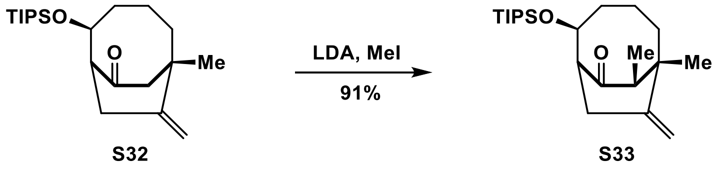

To a stirred solution of ketone **S32** (1.40 g, 4.0 mmol, 1.0 equiv.) in THF (200 mL, 0.02 M/L) was added lithium diisopropylamide (LDA, 2.0 M in THF, 10.0 mL, 20.0 mmol, 5.0 equiv.) at -78 ºC. After stirring for 40 minutes, methyl iodide (2.5 mL, 40.0 mmol, 10.0 equiv.) was added and the mixture was stirred for 3 hours at 25 ºC. Then the reaction was quenched by saturated aqueous NH_4Cl (100 mL) at 0 ºC. The aqueous phase was extracted by EtOAc (100 mL × 3) and the combined organic extracts were washed with saturated brine (100 mL), dried over Na_2SO_4, filtered and concentrated under reduced pressure. The residue was purified by flash-column chromatography on silica gel (hexanes: EtOAc = 100: 1) to afford **S33** (1.32 g, 91% yield) as a colorless oil.

$\mathbf{R}_f$ = 0.5 (silica gel, hexanes: EtOAc = 30: 1);

$[\alpha]_D^{25}$ = -30.0 (c = 0.4, CH_2Cl_2);

IR (film) λ_{max} 2939, 2866, 1705, 1456, 1259, 1095, 1031, 883, 630 cm^{-1};

^{1}H NMR (500 MHz, $CDCl_3$) δ 4.85 (dd, J = 11.0, 2.2 Hz, 2H), 4.77 (d, J = 3.2 Hz, 1H), 4.77 (td, J = 8.6, 3.3 Hz, 1H), 3.97 – 3.91 (m, 1H), 2.74 (ddt, J = 16.0, 5.9, 2.9 Hz, 1H), 2.10 – 2.01 (m, 2H), 1.91 – 1.80 (m, 2H), 1.73 – 1.63 (m, 2H), 1.54 – 1.43 (m, 4H), 1.35 (d, J = 3.7 Hz, 1H), 1.09 (s, 3H), 1.06 (s, 21H) ppm;

^{13}C NMR (125 MHz, $CDCl_3$) δ 215.5, 151.5, 111.3, 75.4, 52.3, 50.8, 49.2, 41.1, 32.8, 32.1, 29.3, 18.6, 18.3, 18.2, 17.3, 12.6 ppm;

HRMS (ESI) calcd. for $C_{22}H_{41}O_2Si$ $[M+H]^+$: 365.2870, found: 365.2866.

Synthesis of compound 12

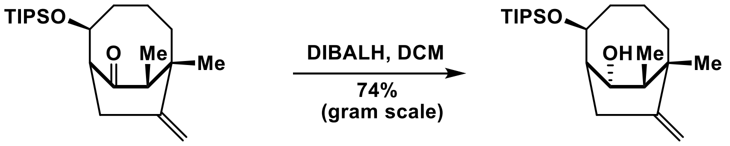

To a stirred solution of **S33** (1.46 g, 4.0 mmol, 1.0 equiv.) in DCM (40 mL, 0.1 M/L) was added diisobutylaluminium hydride (DIBAL, 1.5 M in toluene, 13.3 mL, 20.0 mmol, 5.0 equiv.) dropwise at -78 ºC. Stirring was continued at the same temperature for 5 hours. The reaction was quenched with saturated aqueous potassium sodium tartrate (20 mL) at -78 ºC, stirred for 1 hour at 25 ºC and diluted with DCM (20 mL). The layers were separated, and the aqueous phase was extracted with DCM (20 mL × 3). The combined organic extracts were washed with saturated brine (20 mL), dried over anhydrous Na_2SO_4, concentrated in vacuum. The residue was chromatographed on silica gel (hexanes: EtOAc = 100: 1) to give **12** (1.08 g, 74% yield) as a colorless oil.

$\mathbf{R}_f$ = 0.5 (silica gel, hexanes: EtOAc = 30: 1);

$[\alpha]_D^{25}$ = -12.86 (c = 0.7, CH_2Cl_2);

IR (film) λ_{max} 3500, 2943, 2869, 1458, 1053, 883, 680 cm^{-1};

^{1}H NMR (500 MHz, $CDCl_3$) δ 5.08 (s, 1H), 4.81 (s, 2H), 4.13 – 4.02 (m, 1H), 3.59 (q, J = 6.2 Hz, 1H), 2.77 – 2.69 (m, 1H), 2.64 (dd, J = 17.4, 9.6 Hz, 1H), 2.48 – 2.36 (m, 1H), 2.06 – 1.90 (m, 4H), 1.77 – 1.70 (m, 1H), 1.57 – 1.47 (m, 2H), 1.13 – 1.05 (m, 24H), 1.00 (d, J = 7.0 Hz, 3H) ppm;

^{13}C NMR (125 MHz, $CDCl_3$) δ 151.1, 109.8, 79.6, 78.4, 49.2, 46.3, 41.6, 41.1, 34.2, 33.4, 28.3, 21.2, 19.4, 18.3, 18.2, 12.4 ppm;

HRMS (ESI) calcd. for $C_{22}H_{43}O_2Si$ $[M+H]^+$: 367.3027, found: 367.3024.

Synthesis of compound 12'

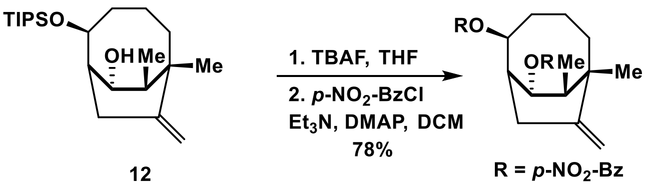

To a stirred solution of compound **12** (18 mg, 0.05 mmol, 1.0 equiv.) in THF (2.5 mL, 0.02 M/L) was added TBAF (1.0 M in THF, 0.1 mL, 0.1 mmol, 2.0 equiv.) at 25 ºC. Then the reaction was stirred for 2 hours at 25 ºC. The solvent was evaporated under reduced pressure afford the crude product, which was used for the next step without further purification.

A solution of the above crude product in anhydrous DCM (5 mL, 0.01 M/L) was added Et_3N (0.014 mL, 0.1 mmol, 2.0 equiv.) and DMAP (6 mg, 0.05 mmol, 1.0 equiv.) at 0 ºC. After being stirred at the same temperature for 5 minutes, *p*-nitrobenzoyl chloride (19 mg, 0.1 mmol, 2.0 equiv.) was added to the reaction. The mixture was warmed to 25 ºC and stirred for 30 minutes. The reaction was quenched by addition of saturated aqueous NH_4Cl (5 mL). Then the aqueous layer was extracted with DCM (5 mL × 3). The combined organic layers were washed with saturated brine (5 mL) and dried over Na_2SO_4. The solution was filtered and concentrated. The residue obtained was purified by flash-column chromatography on silica gel (hexanes: EtOAc = 5: 1) to afford **12'** (20 mg, 78% yield for 2 steps) as a white solid suitable for X-ray analysis.

R_f= 0.6 (silica gel, hexanes: EtOAc = 10: 1);

m.p. = 183 - 184 ºC;

IR (film) λ_{max} 3671, 2942, 2898, 1727, 1524, 1288, 1121, 1015, 953, 871, 848, 715 cm^{-1};

^{1}H NMR (500 MHz, $CDCl_3$) δ 8.16 (d, J = 10.3 Hz, 2H), 8.04 (d, J = 8.7 Hz, 2H), 7.77 (d, J = 8.8 Hz, 2H), 7.66 (d, J = 8.7 Hz, 2H), 5.19 (t, J = 7.5 Hz, 1H), 5.03 (s, 1H), 5.01 – 4.93 (m, 2H), 3.22 (t,

J = 10.0 Hz, 1H), 2.89 (dd, J = 17.5, 8.8 Hz, 1H), 2.70 (d, J = 17.9 Hz, 1H), 2.30 (p, J = 7.0 Hz, 1H), 2.24 – 2.14 (m, 2H), 2.12 – 2.04 (m, 1H), 1.94 – 1.85 (m, 1H), 1.77 – 1.67 (m, 2H), 1.20 (s, 3H), 1.06 (d, J = 7.1 Hz, 3H) ppm;

^{13}C NMR (125 MHz, $CDCl_3$) δ 164.4, 164.1, 150.6, 150.1, 148.5, 135.8, 135.7, 130.7, 130.3, 123.5, 122.9, 111.5, 81.2, 81.0, 49.3, 43.0, 41.6, 36.5, 34.2, 29.9, 28.8, 21.7, 20.0 ppm;

HRMS (ESI) calcd. for $C_{27}H_{28}N_2NaO_8$ $[M+Na]^+$: 531.1738, found: 531.1739.

Synthesis of compound S34

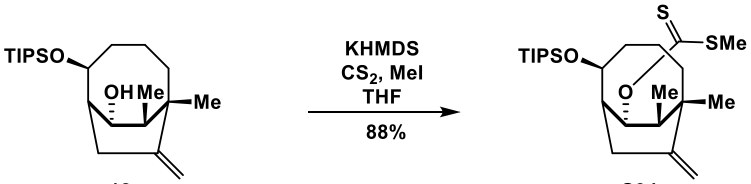

12 S34

To a stirred solution of compound **12** (1.1 g, 3.0 mmol, 1.0 equiv.) in THF (30.0 mL, 0.1 M/L) was added potassium bis(trimethylsilyl)amide (KHMDS, 0.5 M in toluene, 24.0 mL, 12.0 mmol, 4.0 equiv.) at -78 ºC. The reaction mixture was stirred for 10 minutes at the same temperature, followed by dropwise addition of CS_2 (0.91 mL, 15.0 mmol, 5.0 equiv.) and MeI (0.93 mL, 15.0 mmol, 5.0 equiv.). Stirring was continued for 30 minutes and then the reaction was quenched with saturated aqueous NH_4Cl (20 mL). The layers were separated, and the aqueous phase was extracted with EtOAc (20 mL × 3). The combined organic extracts were washed with saturated brine (20 mL), dried over anhydrous Na_2SO_4, concentrated in vacuo. The residue was purified by flash-column chromatography on silica gel (hexanes) to afford thioester **S34** (1.2 g, 88% yield) as a colorless oil.

R_f = 0.4 (silica gel, hexanes: EtOAc = 100: 1);

$[\alpha]_D^{25}$ = -17.69 (c = 1.3, CH_2Cl_2);

IR (film) λ_{max} 2941, 2864, 1625, 1462,1224, 1064, 883, 682 cm^{-1};

^{1}H NMR (500 MHz, $CDCl_3$) δ 5.61 – 5.52 (m, 1H), 4.91 – 4.83 (m, 2H), 3.81 (ddd, J = 10.3, 5.2, 2.0 Hz, 1H), 2.96 (t, J = 8.2 Hz, 1H), 2.80 (dd, J = 17.9, 9.8 Hz, 1H), 2.55 – 2.49 (m, 4H), 2.38 – 2.29 (m, 1H), 2.09 – 1.95 (m, 2H), 1.89 – 1.74 (m, 2H), 1.62 – 1.57 (m, 1H), 1.53 – 1.43 (m, 1H), 1.11 (s, 3H), 1.03 (s, 21H), 0.94 (d, J = 7.0 Hz, 3H) ppm;

^{13}C NMR (125 MHz, $CDCl_3$) δ 215.6, 149.1, 110.4, 88.6, 79.0, 49.3, 43.1, 41.9, 40.0, 35.1, 33.9, 28.0, 22.5, 18.7, 18.6, 18.44, 18.36, 12.7 ppm;

HRMS (ESI) calcd. for $C_{24}H_{45}O_2S_2Si$ $[M+H]^+$: 457.2625, found: 457.2621.

Synthesis of compound 13

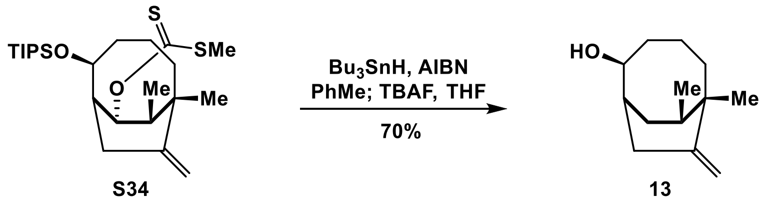

To a stirred solution of compound **S34** (1.19 g, 2.6 mmol, 1.0 equiv.) in toluene (26 mL, 0.1 M/L) was sequentially added 2,2'-azobis (2-methylpropionitrile (AIBN, 43 mg, 0.26 mmol, 0.1 equiv.) and *n*-Bu_3SnH (1.4 mL, 5.2 mmol, 2.0 equiv.) at 25 ºC. The mixture was degassed under argon for 30 minutes and then heated to 80 ºC for 2 hours. The solvent was evaporated in vacuo and the residue was dissolved in THF (26 mL, 0.1 M/L). Then TBAF (1.0 M in THF, 5.2 mL, 5.2 mmol, 2.0 equiv.) was added at 25 ºC. The reaction was heated to 50 ºC for 2 hours. Then the reaction mixture was cooled to 25 ºC and the solvent was evaporated under reduced pressure. The residue was chromatographed on silica gel (hexanes: EtOAc = 10: 1) to give **13** (353 mg, 70% yield) as a colorless oil.

$\mathbf{R}_f$ = 0.4 (silica gel, hexanes: EtOAc = 5: 1);

$[\alpha]_D^{25}$ = -13.33 (c = 0.3, CH_2Cl_2);

IR (film) λ_{max} 3346, 2924, 1444, 1010, 837 cm^{-1};

^{1}H NMR (500 MHz, $CDCl_3$) δ 4.86 (dd, *J* = 4.4, 2.8 Hz, 2H), 4.91 – 4.81 (m, 2H), 3.74 (q, *J* = 4.3 Hz, 1H), 2.71 (ddt, *J* = 16.7, 6.7, 2.9 Hz, 1H), 2.76 – 2.66 (m, 1H), 2.53 (dd, *J* = 15.6, 9.2 Hz, 1H), 2.23 (dt, *J* = 11.3, 5.8 Hz, 1H), 2.28 – 2.18 (m, 1H), 2.15 (d, *J* = 16.8 Hz, 1H), 1.95 – 1.86 (m, 2H), 1.73 – 1.56 (m, 4H), 1.46 – 1.35 (m, 2H), 1.27 – 1.21 (m, 1H), 1.08 (s, 3H), 0.99 (d, *J* = 7.3 Hz, 3H) ppm;

^{13}C NMR (125 MHz, $CDCl_3$) δ 154.2, 110.0, 74.0, 49.5, 41.1, 35.4, 35.3, 33.6, 32.3, 31.2, 27.6, 24.0, 20.1 ppm;

HRMS (ESI) calcd. for $C_{13}H_{21}$ $[M+H-H_2O]^+$: 177.1638, found: 177.1637.

Synthesis of compound 14

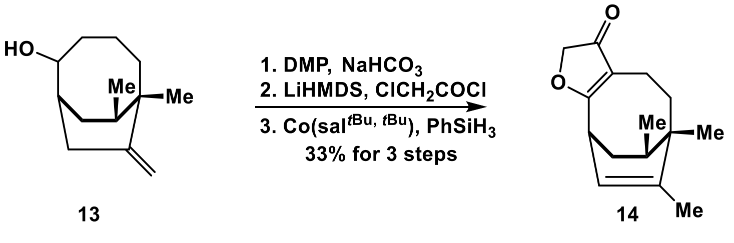

A solution of compound **13** (427 mg, 2.2 mmol, 1.0 equiv.) in DCM (22 mL, 0.1 M/L) was added $NaHCO_3$ (924 mg, 11.0 mmol, 5.0 equiv.) and DMP (1.87 g, 4.4 mmol, 2.0 equiv.) at 0 ºC. Then the

reaction was stirred for 2 hours at 25 ºC. TLC showed the consumption of the starting material. The reaction was quenched by saturated aqueous $Na_2S_2O_3$ (20 mL) and saturated aqueous $NaHCO_3$ (20 mL) at 0 ºC. The mixture was stirred at 25 ºC until a clear solution was obtained, then extracted by DCM (50 mL × 3) and the combined organic extracts were washed with saturated brine (50 mL), dried over Na_2SO_4, filtered and concentrated under reduced pressure. The residue was purified quickly by column chromatography on silica gel (hexanes: EtOAc = 10: 1) to afford the desired ketone (**R_f**= 0.5 (hexanes: EtOAc = 5: 1)) and an inseparable mixture of unidentified byproducts. The desired ketone was not characterized and was used directly in the next step.

To a stirred solution of above ketone (371 mg, 1.9 mmol, 1.0 equiv.) in THF (19 mL, 0.1 M/L) was added LiHMDS (1.0 M in THF, 2.9 mL, 2.9 mmol, 1.5 equiv.) at -78 ºC, and the mixture was stirred for 1 hour at -78 ºC. Then the reaction mixture was added freshly distilled chloroacetyl chloride (0.46 mL, 5.8 mmol, 2.0 equiv.) in THF (6 mL) at -78 ºC. After being stirred at the same temperature for 20 minutes, the mixture was warmed to 25 ºC and stirring was continued for another 1 hour. The reaction mixture was poured into a slurry of ammonia (20 mL) and crushed ice (20 g), and stirred for 1 hour. Then the mixture was extracted with Et_2O (20 mL × 3) and the combined organic extracts were washed with saturated brine (20 mL), dried over Na_2SO_4, filtered and concentrated under reduced pressure. The residue was purified quickly by column chromatography on silica gel (hexanes: EtOAc = 10: 1) to afford the desired compound (**R_f** = 0.5 (hexanes: EtOAc = 4: 1)) and an inseparable mixture of unidentified byproducts. The desired ketone was not characterized and was used directly in the next step.

To a solution of above compound (238 mg, 1.0 mmol, 1.0 equiv.) in acetone (10 mL, 0.1 M/L) was added Co (II) catalyst[16] (62 mg, 0.1 mmol, 0.1 equiv.) and $PhSiH_3$ (25 μL, 0.2 mmol, 0.2 equiv.) at 25 ºC. The mixture was degassed with argon for 30 minutes and stirring was continued under argon for 24 hours. The solvent was removed under reduced pressure and the residue was purified by flash chromatography (hexanes: EtOAc = 10: 1) to give compound **14** (169 mg, 33% yield for 3 steps) as a colorless oil.

R_f= 0.5 (silica gel, hexanes: EtOAc = 10: 1);

$[\alpha]_D^{25}$= +64.17 (*c* = 1.2, $CHCl_3$);

IR (film) λ_{max} 2920, 2851, 2361, 1697, 1612, 1404, 1294, 1107, 1001, 842, 581 cm^{-1};

^1H NMR (500 MHz, $CDCl_3$) δ 5.79 (d, *J* = 7.1 Hz, 1H), 4.40 (d, *J* = 1.7 Hz, 2H), 4.43 – 4.35 (m, 2H), 3.20 – 3.08 (m, 1H), 2.38 (dt, *J* = 15.3, 4.1 Hz, 1H), 1.99 – 1.87 (m, 3H), 1.75 – 1.71 (m, 1H),

1.70 (d, J = 1.2 Hz, 3H), 1.64 (ddd, J = 14.0, 12.2, 3.9 Hz, 1H), 1.45 (ddd, J = 12.6, 9.6, 5.6 Hz, 1H), 1.06 (s, 3H), 0.91 (d, J = 6.9 Hz, 3H) ppm;

^{13}C NMR (125 MHz, $CDCl_3$) δ 202.4, 191.8, 144.0, 120.8, 114.4, 74.0, 45.8, 41.3, 37.2, 37.1, 36.9, 25.0, 20.5, 19.4, 16.8 ppm;

HRMS (ESI) calcd. for $C_{15}H_{21}O_2$ $[M+H]^+$: 233.1536, found: 233.1536.

Synthesis of nakafuran-8

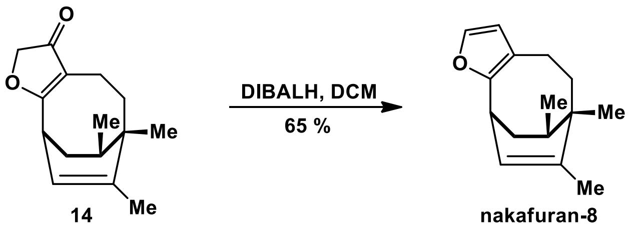

To a solution of lactone **14** (66 mg, 0.28 mmol, 1.0 equiv.) in DCM (10 mL, 0.028 M/L) was added a solution of diisobutylaluminium hydride (DIBAL, 1.0 M in toluene, 1.4 mL, 1.4 mmol, 5.0 equiv.) dropwise at -20 ºC. Stirring was continued for 30 minutes at the same temperature and then the mixture was allowed to warm to 0 ºC for 90 minutes. The reaction was quenched by addition of a saturated aqueous potassium sodium tartrate (10 mL) and the mixture was stirred at 25 ºC until a clear solution was obtained. The product was extracted with DCM (10 mL × 3), then combined organic extracts were washed with saturated brine (10 mL), dried with anhydrous Na_2SO_4, concentrated under reduced pressure. The residue was purified by flash-column chromatography on silica gel (hexanes: EtOAc = 100: 1, or DCM 100%) gave the title compound **nakafuran-8** (40 mg, 65% yield) as a colorless oil.

R_f= 0.6 (silica gel, hexanes: EtOAc = 100: 1);

$[\alpha]_D^{25}$= +17.20 (c = 2.5, $CHCl_3$);

IR (film) λ_{max} 3020, 2924, 1508, 1456, 1261, 1016, 800 cm^{-1};

^{1}H NMR (500 MHz, $CDCl_3$) δ 7.13 (d, J = 1.7 Hz, 1H), 6.07 (d, J = 1.6 Hz, 1H), 5.96 (d, J = 7.5 Hz, 1H), 3.45 (dt, J = 7.1, 3.4 Hz, 1H), 2.44 (ddd, J = 14.9, 12.0, 6.0 Hz, 1H), 2.26 (dt, J = 14.9, 3.7 Hz, 1H), 1.90 – 1.76 (m, 4H), 1.71 (d, J = 1.2 Hz, 3H), 1.26 (ddd, J = 14.6, 3.8 Hz, 1H), 1.06 (s, 3H), 0.89 (d, J = 7.1 Hz, 3H) ppm;

^{13}C NMR (125 MHz, $CDCl_3$) δ 151.0, 141.2, 138.4, 124.5, 118.5, 113.8, 48.1, 40.9, 39.1, 36.6, 34.8, 24.4, 23.2, 20.4, 18.8 ppm;

HRMS (ESI) calcd. for $C_{15}H_{21}O$ $[M+H]^+$: 217.1587, found: 217.1585.

Note: According to the above procedure, a total of 160 mg of **nakafuran-8** was prepared readily

after 4 simple parallel operations.

5. X-ray Crystallographic Data

Crystallographic Data of 3a (CCDC 2065575)

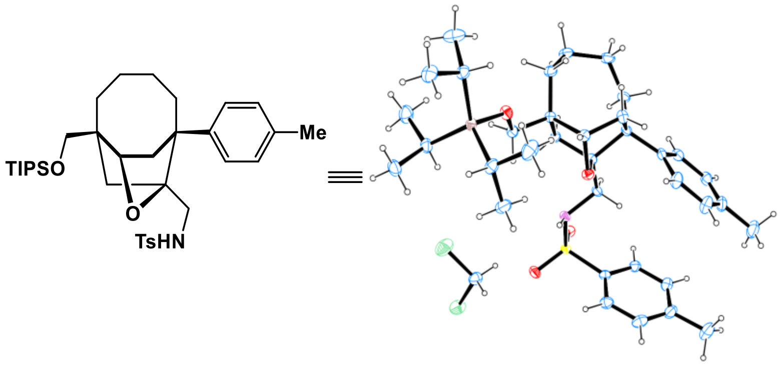

Supplementary Table 4. Crystal data and structure refinement for compound 3a.

Identification code	**3a**
Empirical formula	$C_{36}H_{55}Cl_2NO_4SSi$
Formula weight	696.86
Temperature/K	100
Crystal system	triclinic
Space group	P-1
a/Å	9.5902(3)
b/Å	13.6666(4)
c/Å	15.0123(5)
α/°	71.7120(10)
β/°	88.8460(10)
γ/°	85.6510(10)
Volume/Å^3	1862.82(10)
Z	2
ρ_{calc}g/cm^3	1.242
μ/mm^{-1}	0.300
F(000)	748.0
Crystal size/mm^3	0.36 × 0.35 × 0.22
Radiation	MoKα (λ = 0.71073)
2Θ range for data collection/°	4.87 to 56.756
Index ranges	$-12 \leq h \leq 12$, $-18 \leq k \leq 13$, $-20 \leq l \leq 20$
Reflections collected	21234
Independent reflections	9219 [R_{int} = 0.0392, R_{sigma} = 0.0508]
Data/restraints/parameters	9219/1/417
Goodness-of-fit on F^2	1.029
Final R indexes [I>=2σ (I)]	R_1 = 0.0395, wR_2 = 0.0974
Final R indexes [all data]	R_1 = 0.0511, wR_2 = 0.1064
Largest diff. peak/hole / e Å^{-3}	0.38/-0.50

Crystallographic Data of 3b (CCDC 2068651)

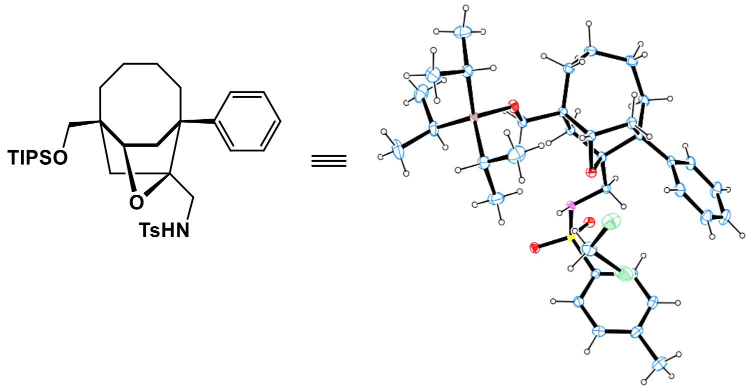

Supplementary Table 5. Crystal data and structure refinement for compound 3b.

Identification code	**3b**
Empirical formula	$C_{35}H_{53}Cl_2NO_4SSi$
Formula weight	682.83
Temperature/K	100
Crystal system	monoclinic
Space group	$P2_1/n$
a/Å	15.0662(3)
b/Å	9.6677(2)
c/Å	25.4719(6)
α/°	90
β/°	105.2520(10)
γ/°	90
Volume/Å^3	3579.44(13)
Z	4
ρ_{calc}g/cm^3	1.267
μ/mm^{-1}	2.793
F(000)	1464.0
Crystal size/mm^3	0.32 × 0.21 × 0.19
Radiation	CuKα (λ = 1.54178)
2Θ range for data collection/°	6.196 to 136.594
Index ranges	$-15 \leq h \leq 18$, $-11 \leq k \leq 11$, $-30 \leq l \leq 30$
Reflections collected	43453
Independent reflections	6565 [R_{int} = 0.0466, R_{sigma} = 0.0272]
Data/restraints/parameters	6565/1/408
Goodness-of-fit on F^2	1.026
Final R indexes [I>=2σ (I)]	R_1 = 0.0321, wR_2 = 0.0838
Final R indexes [all data]	R_1 = 0.0342, wR_2 = 0.0848
Largest diff. peak/hole / e Å^{-3}	0.58/-0.35

Crystallographic Data of 3d (CCDC 2053925)

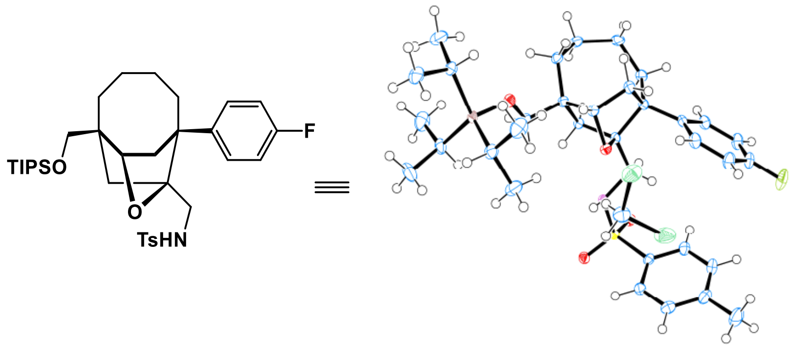

Supplementary Table 6. Crystal data and structure refinement for compound 3d.

Identification code	**3d**
Empirical formula	$C_{35}H_{52}Cl_2FNO_4SSi$
Formula weight	700.82
Temperature/K	100
Crystal system	monoclinic
Space group	$P2_1/n$
a/Å	15.0074(3)
b/Å	9.7398(2)
c/Å	25.6723(5)
α/°	90
β/°	105.0260(10)
γ/°	90
Volume/Å^3	3624.19(13)
Z	4
ρ_{calc}g/cm^3	1.284
μ/mm^{-1}	2.813
F(000)	1496.0
Crystal size/mm^3	0.28 × 0.24 × 0.24
Radiation	CuKα (λ = 1.54178)
2Θ range for data collection/°	6.214 to 136.564
Index ranges	$-18 \leq h \leq 18$, $-11 \leq k \leq 10$, $-29 \leq l \leq 30$
Reflections collected	41499
Independent reflections	6646 [R_{int} = 0.0503, R_{sigma} = 0.0282]
Data/restraints/parameters	6646/0/417
Goodness-of-fit on F^2	1.032
Final R indexes [I>=2σ (I)]	R_1 = 0.0318, wR_2 = 0.0812
Final R indexes [all data]	R_1 = 0.0353, wR_2 = 0.0832
Largest diff. peak/hole / e Å^{-3}	0.38/-0.38

Crystallographic Data of 3f (CCDC 2053921)

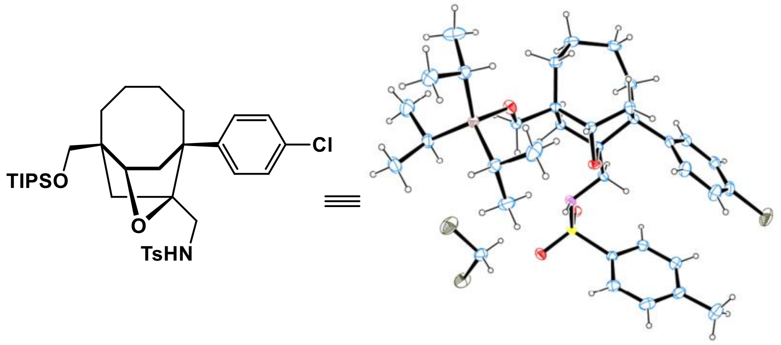

Supplementary Table 7. Crystal data and structure refinement for compound 3f.

Identification code	**3f**
Empirical formula	$C_{35}H_{52}Cl_3NO_4SSi$
Formula weight	717.27
Temperature/K	100
Crystal system	triclinic
Space group	P-1
a/Å	9.5822(6)
b/Å	13.6805(9)
c/Å	14.8952(10)
α/°	71.774(2)
β/°	88.066(2)
γ/°	85.218(2)
Volume/Å^3	1848.1(2)
Z	2
ρ_{calc}g/cm^3	1.289
μ/mm^{-1}	0.374
F(000)	764.0
Crystal size/mm^3	0.35 × 0.33 × 0.28
Radiation	MoKα (λ = 0.71073)
2Θ range for data collection/°	4.266 to 56.54
Index ranges	$-12 \leq h \leq 12$, $-18 \leq k \leq 18$, $-19 \leq l \leq 19$
Reflections collected	26663
Independent reflections	9131 [R_{int} = 0.0344, R_{sigma} = 0.0421]
Data/restraints/parameters	9131/0/418
Goodness-of-fit on F^2	1.052
Final R indexes [I>=2σ (I)]	R_1 = 0.0412, wR_2 = 0.0946
Final R indexes [all data]	R_1 = 0.0612, wR_2 = 0.1050
Largest diff. peak/hole / e Å^{-3}	0.46/-0.45

Crystallographic Data of 3g (CCDC 2053927**)**

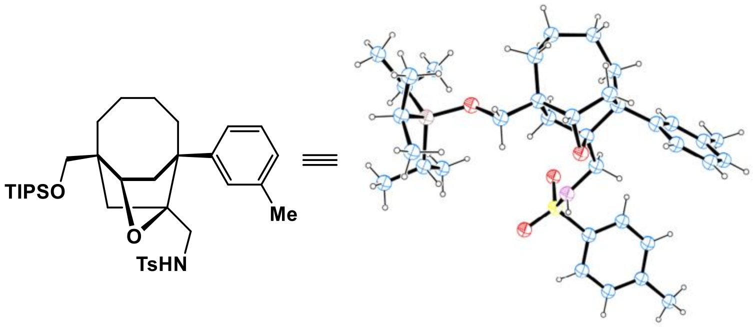

Supplementary Table 8. Crystal data and structure refinement for compound 3g.

Identification code	**3g**
Empirical formula	$C_{35}H_{53}NO_4SSi$
Formula weight	611.93
Temperature/K	100.0
Crystal system	triclinic
Space group	P-1
a/Å	10.0861(5)
b/Å	10.7996(5)
c/Å	32.1990(17)
α/°	93.896(2)
β/°	93.313(2)
γ/°	96.997(2)
Volume/Å^3	3465.4(3)
Z	4
ρ_{calc}g/cm^3	1.173
μ/mm^{-1}	1.444
F(000)	1328.0
Crystal size/mm^3	0.28 × 0.16 × 0.11
Radiation	CuKα (λ = 1.54178)
2Θ range for data collection/°	8.272 to 136.632
Index ranges	$-10 \le h \le 12$, $-13 \le k \le 12$, $-38 \le l \le 38$
Reflections collected	64998
Independent reflections	12658 [R_{int} = 0.0328, R_{sigma} = 0.0249]
Data/restraints/parameters	12658/2/790
Goodness-of-fit on F^2	1.020
Final R indexes [I>=2σ (I)]	R_1 = 0.0377, wR_2 = 0.0990
Final R indexes [all data]	R_1 = 0.0412, wR_2 = 0.1016
Largest diff. peak/hole / e Å^{-3}	0.53/-0.40

Crystallographic Data of 3h (CCDC 2053929)

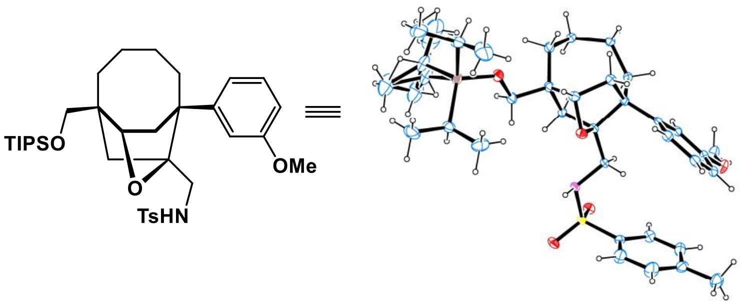

Supplementary Table 9. Crystal data and structure refinement for compound 3h.

Identification code	**3h**
Empirical formula	$C_{35}H_{53}NO_5SSi$
Formula weight	627.93
Temperature/K	100
Crystal system	monoclinic
Space group	$P2_1/c$
a/Å	8.9984(4)
b/Å	13.4031(6)
c/Å	28.7866(12)
α/°	90
β/°	92.208(2)
γ/°	90
Volume/$Å^3$	3469.3(3)
Z	4
$\rho_{calc}g/cm^3$	1.202
μ/mm^{-1}	0.168
F(000)	1360.0
Crystal size/mm^3	0.26 × 0.22 × 0.22
Radiation	MoKα (λ = 0.71073)
2Θ range for data collection/°	4.53 to 55.264
Index ranges	$-11 \leq h \leq 11, -17 \leq k \leq 17, -37 \leq l \leq 37$
Reflections collected	49213
Independent reflections	8064 [R_{int} = 0.0429, R_{sigma} = 0.0316]
Data/restraints/parameters	8064/7/409
Goodness-of-fit on F^2	1.049
Final R indexes [I>=2σ (I)]	R_1 = 0.0418, wR_2 = 0.0972
Final R indexes [all data]	R_1 = 0.0570, wR_2 = 0.1049
Largest diff. peak/hole / e $Å^{-3}$	0.40/-0.43

Crystallographic Data of 3p' (CCDC 2053972)

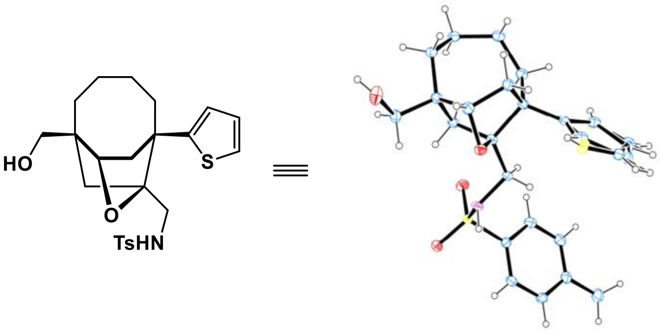

Supplementary Table 10. Crystal data and structure refinement for compound 3p'.

Identification code	**3p'**
Empirical formula	$C_{23}H_{29}NO_4S_2$
Formula weight	447.59
Temperature/K	100
Crystal system	triclinic
Space group	P-1
a/Å	8.7270(4)
b/Å	10.5592(5)
c/Å	12.8921(6)
α/°	71.566(2)
β/°	78.640(2)
γ/°	69.435(2)
Volume/Å^3	1050.28(9)
Z	2
ρ_{calc}g/cm^3	1.415
μ/mm^{-1}	0.285
F(000)	476.0
Crystal size/mm^3	0.41 × 0.38 × 0.36
Radiation	MoKα (λ = 0.71073)
2Θ range for data collection/°	4.668 to 56.792
Index ranges	-11 ≤ h ≤ 11, -14 ≤ k ≤ 14, -17 ≤ l ≤ 17
Reflections collected	20731
Independent reflections	5269 [R_{int} = 0.0505, R_{sigma} = 0.0450]
Data/restraints/parameters	5269/50/286
Goodness-of-fit on F^2	1.028
Final R indexes [I>=2σ (I)]	R_1 = 0.0409, wR_2 = 0.0989
Final R indexes [all data]	R_1 = 0.0583, wR_2 = 0.1078
Largest diff. peak/hole / e Å^{-3}	0.43/-0.44

Crystallographic Data of 3s (CCDC 2054598)

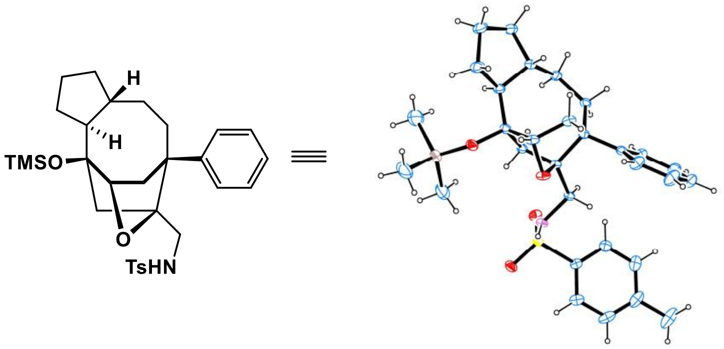

Supplementary Table 11. Crystal data and structure refinement for compound 3s.

Identification code	**3s**
Empirical formula	$C_{30}H_{41}NO_4SSi$
Formula weight	539.79
Temperature/K	100
Crystal system	triclinic
Space group	P-1
a/Å	10.4649(6)
b/Å	10.4887(6)
c/Å	13.8168(8)
α/°	109.158(2)
β/°	91.380(2)
γ/°	94.239(2)
Volume/Å^3	1426.78(14)
Z	2
ρ_{calc}g/cm^3	1.256
μ/mm^{-1}	0.191
F(000)	580.0
Crystal size/mm^3	0.31 × 0.25 × 0.22
Radiation	MoKα (λ = 0.71073)
2Θ range for data collection/°	5.128 to 61.296
Index ranges	$-14 \leq h \leq 14$, $-15 \leq k \leq 15$, $-19 \leq l \leq 19$
Reflections collected	24536
Independent reflections	8782 [R_{int} = 0.0398, R_{sigma} = 0.0562]
Data/restraints/parameters	8782/2/350
Goodness-of-fit on F^2	1.025
Final R indexes [I>=2σ (I)]	R_1 = 0.0513, wR_2 = 0.1147
Final R indexes [all data]	R_1 = 0.0822, wR_2 = 0.1294
Largest diff. peak/hole / e Å^{-3}	0.50/-0.53

Crystallographic Data of 3t' (CCDC 2054000)

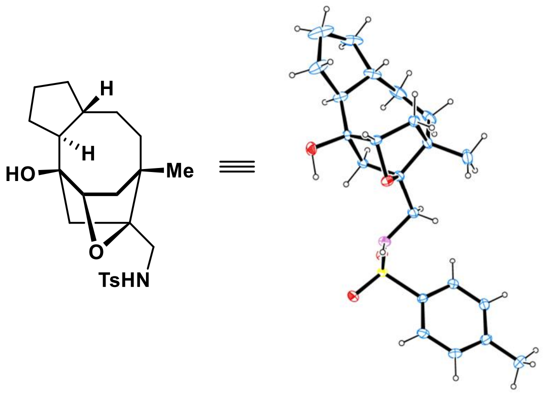

Supplementary Table 12. Crystal data and structure refinement for compound 3t'.

Identification code	**3t'**
Empirical formula	$C_{22.5}ClH_{32}NO_4S$
Formula weight	448.00
Temperature/K	100
Crystal system	triclinic
Space group	P-1
a/Å	9.8573(4)
b/Å	10.6106(4)
c/Å	11.4503(4)
α/°	80.4560(10)
β/°	80.9340(10)
γ/°	81.7810(10)
Volume/Å^3	1157.85(8)
Z	2
ρ_{calc}g/cm^3	1.285
μ/mm^{-1}	0.283
F(000)	478.0
Crystal size/mm^3	0.35 × 0.32 × 0.28
Radiation	MoKα (λ = 0.71073)
2Θ range for data collection/°	4.944 to 55.114
Index ranges	-12 ≤ h ≤ 12, -13 ≤ k ≤ 13, -14 ≤ l ≤ 14
Reflections collected	22351
Independent reflections	5331 [R_{int} = 0.0365, R_{sigma} = 0.0320]
Data/restraints/parameters	5331/1/264
Goodness-of-fit on F^2	1.033
Final R indexes [I>=2σ (I)]	R_1 = 0.0402, wR_2 = 0.1069
Final R indexes [all data]	R_1 = 0.0524, wR_2 = 0.1137
Largest diff. peak/hole / e Å^{-3}	0.38/-0.42

Crystallographic Data of 3w' (CCDC 1920650)

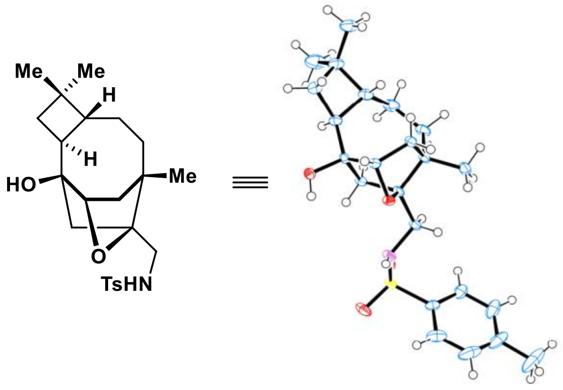

Supplementary Table 13. Crystal data and structure refinement for compound 3w'.

Identification code	**3w'**
Empirical formula	$C_{23.5}H_{33}ClNO_4S$
Formula weight	461.02
Temperature/K	100
Crystal system	triclinic
Space group	P-1
a/Å	10.265(11)
b/Å	10.431(9)
c/Å	11.530(11)
α/°	89.429(15)
β/°	80.36(3)
γ/°	83.76(3)
Volume/Å^3	1210(2)
Z	2
ρ_{calc}g/cm^3	1.265
μ/mm^{-1}	0.273
F(000)	492.0
Crystal size/mm^3	0.4 × 0.38 × 0.3
Radiation	MoKα (λ = 0.71073)
2Θ range for data collection/°	4.938 to 55.138
Index ranges	$-13 \le h \le 13$, $-12 \le k \le 13$, $-14 \le l \le 15$
Reflections collected	52603
Independent reflections	5548 [R_{int} = 0.0365, R_{sigma} = 0.0186]
Data/restraints/parameters	5548/0/300
Goodness-of-fit on F^2	1.032
Final R indexes [I>=2σ (I)]	R_1 = 0.0559, wR_2 = 0.1506
Final R indexes [all data]	R_1 = 0.0629, wR_2 = 0.1563
Largest diff. peak/hole / e Å^{-3}	1.04/-0.80

Crystallographic Data of 3x' (CCDC 1920646)

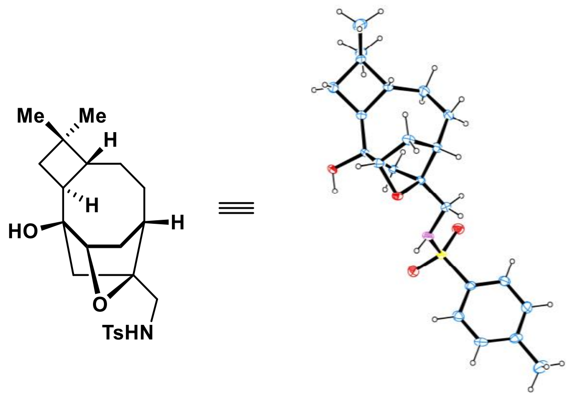

Supplementary Table 14. Crystal data and structure refinement for compound 3x'.

Identification code	**3x'**
Empirical formula	$C_{22}H_{31}NO_4S$
Formula weight	405.54
Temperature/K	100.0
Crystal system	monoclinic
Space group	$P2_1/c$
a/Å	15.0110(7)
b/Å	12.0638(6)
c/Å	11.8356(6)
α/°	90
β/°	108.4450(10)
γ/°	90
Volume/Å^3	2033.20(17)
Z	4
ρ_{calc}g/cm^3	1.325
μ/mm^{-1}	0.188
F(000)	872.0
Crystal size/mm^3	0.41 × 0.38 × 0.29
Radiation	MoKα (λ = 0.71073)
2Θ range for data collection/°	4.426 to 55.156
Index ranges	$-19 \le h \le 19$, $-15 \le k \le 15$, $-15 \le l \le 15$
Reflections collected	42455
Independent reflections	4682 [R_{int} = 0.0430, R_{sigma} = 0.0232]
Data/restraints/parameters	4682/6/262
Goodness-of-fit on F^2	1.049
Final R indexes [I>=2σ (I)]	R_1 = 0.0466, wR_2 = 0.1155
Final R indexes [all data]	R_1 = 0.0539, wR_2 = 0.1201
Largest diff. peak/hole / e Å^{-3}	0.89/-0.56

Crystallographic Data of 3af (CCDC 2053961**)**

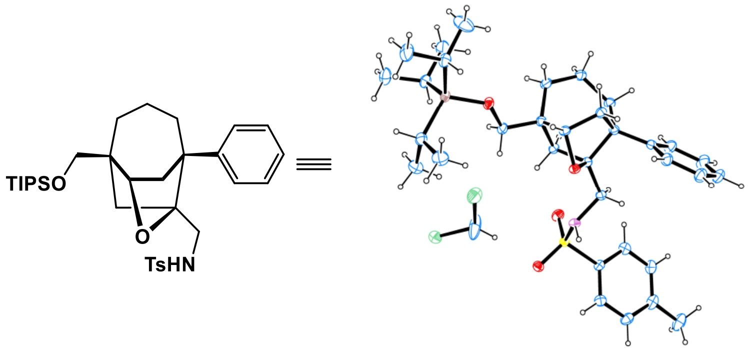

Supplementary Table 15. Crystal data and structure refinement for compound 3af.

Identification code	**3af**
Empirical formula	$C_{34}H_{50}Cl_2NO_4SSi$
Formula weight	667.80
Temperature/K	100
Crystal system	monoclinic
Space group	C2/c
a/Å	15.9108(6)
b/Å	13.8858(5)
c/Å	32.6458(12)
α/°	90
β/°	102.642(2)
γ/°	90
Volume/Å^3	7037.7(5)
Z	8
ρ_{calc}g/cm^3	1.261
μ/mm^{-1}	2.830
F(000)	2856.0
Crystal size/mm^3	0.35 × 0.26 × 0.17
Radiation	CuKα (λ = 1.54178)
2Θ range for data collection/°	5.548 to 137.014
Index ranges	-19 ≤ h ≤ 19, -15 ≤ k ≤ 16, -39 ≤ l ≤ 39
Reflections collected	35181
Independent reflections	6458 [R_{int} = 0.0511, R_{sigma} = 0.0304]
Data/restraints/parameters	6458/0/395
Goodness-of-fit on F^2	1.048
Final R indexes [I>=2σ (I)]	R_1 = 0.0384, wR_2 = 0.1065
Final R indexes [all data]	R_1 = 0.0416, wR_2 = 0.1084
Largest diff. peak/hole / e Å^{-3}	0.63/-0.46

Crystallographic Data of 3ag (CCDC 2053962)

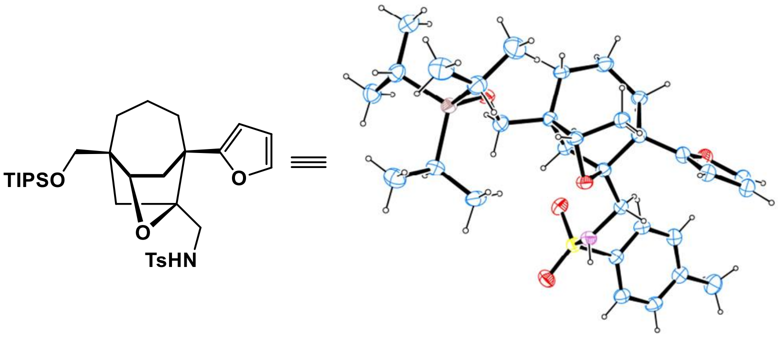

Supplementary Table 16. Crystal data and structure refinement for compound 3ag.

Identification code	**3ag**
Empirical formula	$C_{31}H_{47}NO_5SSi$
Formula weight	573.84
Temperature/K	100.15
Crystal system	triclinic
Space group	P-1
a/Å	9.4933(5)
b/Å	10.9111(5)
c/Å	16.0523(8)
α/°	74.453(2)
β/°	80.016(2)
γ/°	70.977(2)
Volume/$Å^3$	1507.65(13)
Z	2
$\rho_{calc}g/cm^3$	1.264
μ/mm^{-1}	1.652
F(000)	620.0
Crystal size/mm^3	0.36 × 0.35 × 0.08
Radiation	CuKα (λ = 1.54178)
2Θ range for data collection/°	5.74 to 136.732
Index ranges	$-11 \leq h \leq 11$, $-12 \leq k \leq 13$, $0 \leq l \leq 19$
Reflections collected	10456
Independent reflections	10456 [R_{int} = ?, R_{sigma} = 0.0706]
Data/restraints/parameters	10456/0/360
Goodness-of-fit on F^2	1.076
Final R indexes [I>=2σ (I)]	R_1 = 0.0924, wR_2 = 0.2732
Final R indexes [all data]	R_1 = 0.0963, wR_2 = 0.2770
Largest diff. peak/hole / e $Å^{-3}$	0.48/-0.50

Crystallographic Data of 3ah (CCDC 2053964)

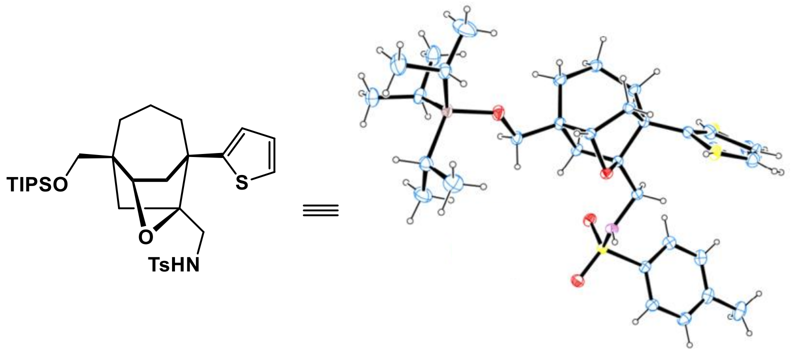

Supplementary Table 17. Crystal data and structure refinement for compound 3ah.

Identification code	**3ah**
Empirical formula	$C_{32}H_{49}Cl_2NO_4S_2Si$
Formula weight	674.83
Temperature/K	100
Crystal system	monoclinic
Space group	C2/c
a/Å	15.9998(11)
b/Å	13.5574(11)
c/Å	32.685(2)
α/°	90
β/°	102.000(3)
γ/°	90
Volume/Å^3	6935.0(9)
Z	8
ρ_{calc}g/cm^3	1.293
μ/mm^{-1}	0.378
F(000)	2880.0
Crystal size/mm^3	0.36 × 0.35 × 0.08
Radiation	MoKα (λ = 0.71073)
2Θ range for data collection/°	4.336 to 55.212
Index ranges	-20 ≤ h ≤ 20, -17 ≤ k ≤ 17, -42 ≤ l ≤ 42
Reflections collected	94462
Independent reflections	8098 [R_{int} = 0.0844, R_{sigma} = 0.0374]
Data/restraints/parameters	8098/136/403
Goodness-of-fit on F^2	1.095
Final R indexes [I>=2σ (I)]	R_1 = 0.0582, wR_2 = 0.1642
Final R indexes [all data]	R_1 = 0.0660, wR_2 = 0.1694
Largest diff. peak/hole / e Å^{-3}	0.54/-0.48

Crystallographic Data of 11’ (CCDC 2054002)

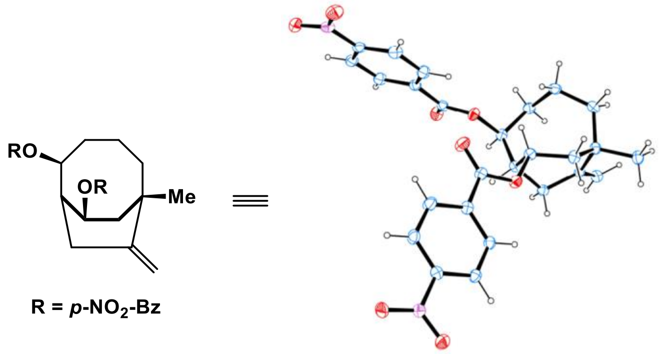

Supplementary Table 18. Crystal data and structure refinement for compound 11’.

Identification code	**11’**
Empirical formula	$C_{26}H_{26}N_2O_8$
Formula weight	494.49
Temperature/K	100
Crystal system	orthorhombic
Space group	$P2_12_12_1$
a/Å	7.3579(3)
b/Å	15.6959(7)
c/Å	19.8263(9)
α/°	90
β/°	90
γ/°	90
Volume/Å^3	2289.72(17)
Z	4
ρ_{calc}g/cm^3	1.434
μ/mm^{-1}	0.897
F(000)	1040.0
Crystal size/mm^3	0.31 × 0.28 × 0.24
Radiation	CuKα (λ = 1.54178)
2Θ range for data collection/°	7.182 to 136.648
Index ranges	$-8 \leq h \leq 8$, $-18 \leq k \leq 18$, $-23 \leq l \leq 23$
Reflections collected	44920
Independent reflections	4195 [R_{int} = 0.0304, R_{sigma} = 0.0144]
Data/restraints/parameters	4195/0/327
Goodness-of-fit on F^2	1.036
Final R indexes [I>=2σ (I)]	R_1 = 0.0213, wR_2 = 0.0547
Final R indexes [all data]	R_1 = 0.0214, wR_2 = 0.0548
Largest diff. peak/hole / e Å^{-3}	0.17/-0.13
Flack parameter	0.00(2)

Crystallographic Data of 12’ (CCDC 2054003)

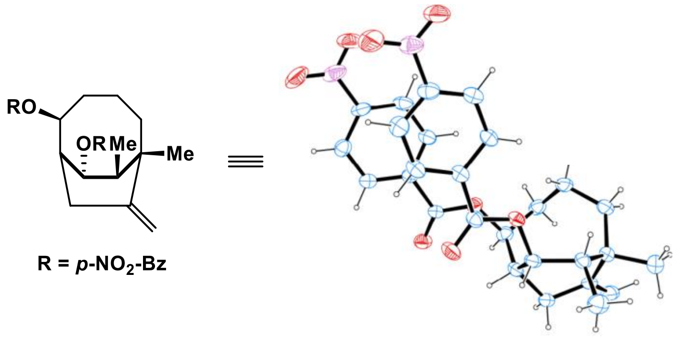

Supplementary Table 19. Crystal data and structure refinement for compound 12’.

Identification code	**12’**
Empirical formula	$C_{27}H_{28}N_2O_8$
Formula weight	508.51
Temperature/K	100.0
Crystal system	monoclinic
Space group	$P2_1$
a/Å	7.1746(5)
b/Å	28.7980(18)
c/Å	11.8165(7)
α/°	90
β/°	92.990(2)
γ/°	90
Volume/Å^3	2438.1(3)
Z	4
ρ_{calc}g/cm^3	1.385
μ/mm^{-1}	0.857
F(000)	1072.0
Crystal size/mm^3	0.32 × 0.24 × 0.19
Radiation	CuKα (λ = 1.54178)
2Θ range for data collection/°	6.138 to 136.598
Index ranges	$-8 \leq h \leq 8$, $-34 \leq k \leq 34$, $-14 \leq l \leq 14$
Reflections collected	8942
Independent reflections	8942 [R_{int} = 0.068, R_{sigma} = 0.0235]
Data/restraints/parameters	8942/1/672
Goodness-of-fit on F^2	1.134
Final R indexes [I>=2σ (I)]	R_1 = 0.0434, wR_2 = 0.1148
Final R indexes [all data]	R_1 = 0.0437, wR_2 = 0.1149
Largest diff. peak/hole / e Å^{-3}	0.24/-0.20
Flack parameter	0.04(4)

6. Computational Section

6.1 Computational Methods

All calculations were carried out with the Gaussian 16[17] software package. Geometries, Hirshfeld charges, molecular orbitals, and energies of ground states and transition states were calculated with the ωB97X-D[18] functional and the 6-311+G (d, p) basis set. A CPCM[19,20,21] implicit solvent model in dichloroethane. Frequency calculations were carried out at the same level of theory to ensure that stationary points were truly minima or saddle points on the potential energy surface. Intrinsic reaction coordinate (IRC) calculations were carried out at this level of theory to confirm all transition states connected reactants and products. Conformational searches were carried out using the CREST conformer-rotamer ensemble sampling tool[22,23], version 2.7.1 with XTB version 6.2 RC2 (SAW190805)[24,25,26]. Images of molecular structures were rendered in CYLview[27].

6.2 Computed Energies

Supplementary Table 20.

Structure	**E**(DCE)	**ΔG**	**G**(DCE)	**ΔH**	**H**(DCE)	Imaginary Frequency
**Ylide**	-1456.813977	0.374932	-1456.439046	0.464771	-1456.349206	
**TS-FAV**	-1456.797382	0.385348	-1456.412033	0.464844	-1456.332538	-331.917
**TS-DISFAV**	-1456.793956	0.385724	-1456.408232	0.464521	-1456.329436	-409.316
**TS-DISFAV-DIA**	-1456.756194	0.382750	-1456.373444	0.464395	-1456.291799	-577.777
**15**	-1456.895981	0.391979	-1456.504002	0.468991	-1456.426990	
**16**	-1456.898401	0.389394	-1456.509008	0.468580	-1456.429821	
**16-dia**	-1456.831250	0.391435	-1456.439815	0.468926	-1456.362324	
**17**	-1456.875162	0.394347	-1456.480816	0.469592	-1456.405570	
**18**	-1456.856478	0.394643	-1456.461835	0.469633	-1456.386844	

6.3. Cartesian Coordinates

Ylide
C -0.25230500 0.05284900 0.44225400
N -0.94218000 -0.37975300 -0.62491500
S -1.99034900 -1.58474700 -0.36889800
O -2.08474000 -2.35965500 -1.60620900
O -1.71058800 -2.33096400 0.86672100
C -3.57437600 -0.78619400 -0.13461100
C -4.01826400 -0.50793900 1.15195500
C -5.22041000 0.16611100 1.32557700
C -5.98773800 0.56651500 0.23056700
C -5.51735800 0.27668700 -1.05255100
C -4.31827900 -0.39591300 -1.24217600
H -3.96445800 -0.62165900 -2.24108900
H -6.09903600 0.58008300 -1.91684400
C -7.30548700 1.26675500 0.42654800
H -7.32124000 1.82697100 1.36336500
H -8.12224900 0.53911800 0.46153500
H -7.51320400 1.95624200 -0.39408600
H -5.56841100 0.38110700 2.33057300
H -3.43306400 -0.82154500 2.00803200
C 0.61140100 1.08992800 0.35559100
O 1.31840200 1.42933700 1.52010300
C 2.21034700 2.28753000 1.29220300
C 2.34256100 2.66060800 -0.14231400
C 2.39690500 4.17945600 -0.33480400
H 1.50391000 4.66225800 0.06671600
H 2.45407500 4.39675200 -1.40370100
H 3.27737300 4.60673100 0.15041800
C 3.65491200 2.01129500 -0.66482200
C 3.73249800 0.49214200 -0.53175100
C 5.05468200 -0.06589700 -1.04996300
H 5.88341800 0.35100100 -0.46666900
H 5.20926300 0.26363500 -2.08295600
C 5.10300400 -1.60026700 -1.01573600
C 4.87853500 -2.18753200 0.35699300
C 3.85264600 -2.99889900 0.61235000
H 3.69718700 -3.42387900 1.59942300
H 3.13572100 -3.26349100 -0.15902300
C 5.87117000 -1.79819100 1.41860100
H 5.81858400 -0.72428400 1.62813200
H 6.89405100 -2.00504300 1.08641600
H 5.69209900 -2.33430200 2.35222200
H 6.08386700 -1.92513100 -1.38339900
H 4.35334900 -1.99810700 -1.70691200
H 2.91042700 0.02034900 -1.08099300
H 3.60887900 0.19558200 0.51724800
H 3.73394700 2.29899100 -1.71905500
H 4.50258500 2.47302800 -0.14626800
C 1.06583500 2.00268500 -0.73662900
H 1.27438700 1.46405200 -1.66284000
H 0.30722700 2.76043700 -0.95987700
H 2.80669700 2.61535200 2.13608200
H -0.35082500 -0.44012800 1.41170400

TS-FAV
C 0.22717600 -1.78375100 0.53021400
N 0.97505100 -2.05130400 -0.57720900
S 2.56219200 -2.05743700 -0.35664300
O 2.97998900 -2.73723100 0.88157100
O 3.17993700 -2.52573200 -1.59999500
C 3.03062200 -0.33635400 -0.14891800
C 3.11291800 0.48951300 -1.26525700
C 3.36356000 1.84269600 -1.09554600
C 3.52918800 2.39259200 0.17901400
C 3.45627200 1.54287900 1.28169800
C 3.20735100 0.18343800 1.12587500
H 3.15527900 -0.47008900 1.98827900
H 3.59479400 1.94632500 2.27949400
C 3.75008500 3.87124700 0.35206200
H 2.79389900 4.40400500 0.33012800
H 4.36946800 4.27606100 -0.45113400
H 4.23164200 4.09280200 1.30622600
H 3.42979400 2.48529300 -1.96761700
H 2.98056100 0.07453600 -2.25769000
C -1.08327600 -1.51169800 0.43612700
O -1.76921700 -1.12774900 1.60125900
C -2.85793900 -0.48726000 1.27279700
C -3.33854400 -0.97047900 -0.09171600
C -4.09225400 -2.27855700 0.27149000
H -3.47225100 -2.96325300 0.85490700
H -4.37162000 -2.78101000 -0.65749900
H -5.00387900 -2.06001800 0.83328200
C -4.27942300 -0.09886100 -0.96687700
C -4.71919900 1.27467800 -0.44452400
C -3.82486200 2.46454500 -0.84431000
H -4.00170700 3.29595100 -0.15447200
H -4.14867400 2.81802800 -1.82735800
C -2.32439300 2.20732500 -0.96034800
C -1.58394100 1.73565300 0.24555600
C -2.21778900 1.49022400 1.43318800
H -1.62089700 1.30478400 2.31893200
H -3.20330800 1.90444300 1.60328100
C -0.10637300 1.58944800 0.08313600
H 0.31769600 2.58128700 -0.11388600
H 0.14533100 0.96837100 -0.78140500
H 0.37358000 1.17500400 0.96782900
H -1.82617500 3.12707900 -1.29233500
H -2.11888300 1.49694500 -1.77226700
H -4.86062400 1.22758400 0.63810200
H -5.71619900 1.47737400 -0.84320900
H -3.82623000 0.01322600 -1.95597300
H -5.18392000 -0.68587900 -1.13422500
C -1.98687500 -1.34820900 -0.74307300
H -1.61698500 -0.56939300 -1.41858900
H -2.07577900 -2.26186300 -1.33569300
H -3.54164700 -0.32553200 2.09854200
H 0.67681200 -1.76266400 1.52487000

TS-DISFAV
C 0.58662000 -1.76609800 0.63014300
N 1.42774100 -2.16949700 -0.33311400
S 2.98582700 -1.80340800 -0.06912700
O 3.44179100 -2.15696500 1.28171100
O 3.75180600 -2.34012300 -1.19171100
C 3.01071200 -0.01500900 -0.16491500
C 2.84363100 0.60144300 -1.40100900
C 2.67943700 1.97656000 -1.45734500
C 2.67256500 2.75195600 -0.29369800
C 2.85677800 2.11209500 0.93223300
C 3.02483000 0.73403400 1.00443200
H 3.15373200 0.24187900 1.96077500
H 2.86009400 2.69590400 1.84669500
C 2.49769200 4.24449200 -0.36899100
H 1.80696100 4.52075300 -1.16830600
H 3.45554600 4.73054200 -0.57847800
H 2.11924900 4.64727200 0.57216900
H 2.54178600 2.45695700 -2.42046600
H 2.82680300 0.00776700 -2.30754200
C -0.75333800 -1.65147100 0.41678100
O -1.52340200 -1.16753600 1.44874700
C -2.56329000 -0.56099800 0.92654000
C -2.96234000 -1.24479600 -0.37027500
C -3.99464700 -2.31588800 0.04178300
H -3.60079500 -2.96938100 0.82387300
H -4.22934800 -2.93199600 -0.83025600
H -4.91950500 -1.86318600 0.40611600
C -3.52237400 -0.35763700 -1.48363500
C -4.66314700 0.58965200 -1.11349100
C -4.31489800 1.64920500 -0.05372300
H -4.50970900 1.27668500 0.95633400
H -4.99794200 2.49316800 -0.17882600
C -2.87538600 2.16544500 -0.15890500
C -1.77591600 1.38875900 0.57784200
C -0.64435200 1.00235300 -0.10498900
H 0.30302800 0.86594200 0.39869500
H -0.62768100 0.94174000 -1.18769500
C -1.61827600 1.77458800 2.03909800
H -2.57945200 1.78078300 2.56033800
H -1.20762800 2.78663100 2.10171700
H -0.94069700 1.09845900 2.56304300
H -2.83414400 3.17426300 0.26460400
H -2.60654000 2.27762700 -1.21282000
H -5.54520600 0.02584000 -0.79577500
H -4.95215300 1.10555100 -2.03468200
H -2.70034500 0.22012600 -1.91313900
H -3.85993200 -1.02465700 -2.28302400
C -1.61844500 -1.91616000 -0.77471500
H -1.18878700 -1.48079200 -1.67895700
H -1.74835700 -2.98692600 -0.95805500
H -3.28862000 -0.23364200 1.66186400
H 0.94552800 -1.47445800 1.62068800

TS-DISFAV-DIA
C -0.08025200 -1.46716500 -0.56561400
N -0.98324800 -1.52972900 0.36548300
S -2.51559900 -1.96125900 -0.11898400
O -2.54611800 -2.39266900 -1.51658300
O -3.03234200 -2.87382400 0.89210800
C -3.39234600 -0.41905800 0.00558500
C -3.48839600 0.39903200 -1.11356000
C -4.12700400 1.62521900 -0.99729800
C -4.67135200 2.04266200 0.21956300
C -4.55844500 1.19897700 1.32700100
C -3.92249600 -0.03053300 1.22963700
H -3.84147100 -0.68194600 2.09131300
H -4.97447100 1.50734800 2.28011000
C -5.38940900 3.36007300 0.32785400
H -4.99550400 4.08570400 -0.38606000
H -5.30268900 3.77703900 1.33286100
H -6.45467800 3.22912200 0.11413300
H -4.20535300 2.26771500 -1.86772800
H -3.07088600 0.08047400 -2.06111500
C 1.20236500 -0.94029100 -0.29242800
O 2.12953500 -1.11321800 -1.28515100
C 3.24095300 -0.57042200 -0.83604500
C 3.43779300 -0.89526700 0.60804500
C 4.03672800 -2.31859900 0.68629000
H 3.42868200 -3.04149100 0.13592500
H 4.08150500 -2.63574600 1.73266900
H 5.05123600 -2.33305600 0.27750500
C 4.40066800 0.10506400 1.29040700
C 5.27022300 0.93896600 0.31841700
C 4.69360500 2.21973800 -0.33430700
H 5.47830800 2.62614700 -0.98141500
H 4.56477300 2.96342800 0.45887300
C 3.38502900 2.18009300 -1.15652600
C 2.21702800 1.61670000 -0.37044400
C 1.03724000 1.05853000 -0.86054000
H 0.93680800 0.84243400 -1.91982500
H 0.10407900 1.28770000 -0.35335800
C 2.02360600 2.32967100 0.96454600
H 2.86813500 2.28835700 1.64730900
H 1.86449200 3.39496000 0.74460300
H 1.14039200 1.97443900 1.49723100
H 3.52299300 1.65417400 -2.10370400
H 3.16167300 3.22814100 -1.41410400
H 5.68124500 0.27702800 -0.45191300
H 6.14416200 1.27365700 0.88648300
H 3.85724600 0.75732100 1.97073600
H 5.08307300 -0.46768800 1.92467900
C 1.93660100 -0.94043100 1.04570600
H 1.64820600 -0.09449700 1.66533800
H 1.71208200 -1.85325600 1.60250300
H 4.02959100 -0.51190500 -1.57440300
H -0.30273800 -1.72743100 -1.60401000

PROD-FAV
C -0.35173400 -1.37450700 0.46972400

N 0.43832000 -1.28605700 -0.52363600
S 1.93494900 -2.07025500 -0.36574100
O 2.16874000 -2.73793800 -1.63505400
O 2.00365200 -2.85025400 0.86463000
C 3.00138200 -0.66200500 -0.21900300
C 3.36041800 0.03969100 -1.36436700
C 4.14100800 1.17778100 -1.23329000
C 4.56566200 1.62120200 0.02242100
C 4.18721800 0.89491300 1.15332000
C 3.40557600 -0.24628600 1.04331800
H 3.11200300 -0.80496600 1.92357600
H 4.50684600 1.22616000 2.13522500
C 5.43445200 2.84216600 0.14811800
H 5.32253600 3.30928300 1.12796900
H 5.19382700 3.57908300 -0.62035800
H 6.48740400 2.56990100 0.02706400
H 4.42468800 1.73207500 -2.12124900
H 3.03500300 -0.29933000 -2.34037500
C -1.62597700 -0.61215800 0.45999100
C -2.46950200 -0.74293700 -0.80836300
H -2.07469600 -0.17925300 -1.64745500
H -2.49802000 -1.79428000 -1.10325500
C -3.85797300 -0.27185200 -0.29458700
C -4.90355700 -1.37469300 -0.48585400
H -4.62058300 -2.27830400 0.06127400
H -5.00145700 -1.63325500 -1.54453800
H -5.88322900 -1.04566400 -0.12435100
C -4.37096100 1.03273800 -0.95499900
H -4.91659800 1.62081500 -0.20744400
H -5.12407000 0.74526300 -1.69576200
C -3.37935100 1.94088100 -1.69982500
H -3.98308100 2.72071800 -2.17694600
C -2.27228400 2.66402500 -0.92279500
H -1.79786400 3.35456100 -1.62865700
H -2.72824700 3.30667000 -0.16182800
C -1.12746500 1.85983700 -0.28669800
H -0.58523900 1.30474200 -1.06064300
H -0.41180700 2.60190500 0.08108700
C -1.43426200 0.90647700 0.89431600
C -0.30308300 1.02142400 1.92498600
H 0.68026200 0.89467100 1.46088700
H -0.41245300 0.28246700 2.72411400
H -0.32641800 2.01421200 2.38288000
C -2.80879100 1.13731600 1.58290500
H -3.31541700 2.04299100 1.26107700
C -3.55442200 -0.14177100 1.23288400
H -4.40952100 -0.37022500 1.86803800
O -2.52138700 -1.12007400 1.44842500
H -2.68870400 1.19323000 2.66672200
H -2.93123300 1.38358400 -2.52957800
H -0.10751700 -1.95789500 1.36260400

PROD-DISFAV

C -0.09474100 -1.08910800 0.64834500
N -0.99599700 -1.19299000 -0.24215900
S -2.49695400 -1.79741300 0.27958300
O -2.48341600 -2.09669000 1.70685700
O -2.83497500 -2.85691500 -0.65652200
C -3.54099800 -0.39527300 -0.00600300
C -3.85113800 0.44779200 1.05362500
C -4.64270800 1.56154200 0.81213500
C -5.12495000 1.83954900 -0.46856000
C -4.79201200 0.97361200 -1.51396800
C -4.00270300 -0.14445800 -1.29335200
H -3.75241600 -0.81498100 -2.10643300
H -5.15682900 1.17684900 -2.51483400
C -6.00856400 3.03105700 -0.71547300
H -7.06117500 2.74176800 -0.63908200
H -5.82657600 3.81870000 0.01755200
H -5.85044500 3.43992100 -1.71512100
H -4.89082400 2.22416900 1.63398000
H -3.48154300 0.23450900 2.04905900
C 1.23416600 -0.51434800 0.31632800
O 2.22735900 -1.04237500 1.19754500
C 3.32519500 -0.28462400 0.66403800
H 4.22979300 -0.53151800 1.22005300
C 3.34258500 -0.76784100 -0.81099700
C 3.90404600 -2.19716800 -0.82594900
H 4.97674300 -2.19178500 -0.60586600
H 3.40408500 -2.82884400 -0.08915000
H 3.76432000 -2.64536600 -1.81410900
C 4.17719400 0.09149800 -1.78685600
H 3.54951500 0.81716700 -2.30954200
C 5.34851600 0.81056400 -1.10867700
H 5.84876200 0.10761000 -0.43398400
H 6.09408600 1.07134600 -1.86440700
C 4.94420900 2.09296900 -0.33914800
C 3.44559000 2.22883800 -0.06094200
H 2.90687200 2.22394300 -1.01164600
C 2.83949500 1.17669800 0.88174900
C 3.11344800 1.56421200 2.33912500
H 2.61193200 2.50535900 2.58356700
H 2.74786000 0.79477900 3.02345900
H 4.18567900 1.69570900 2.51354700
C 1.30897800 0.99602800 0.63957100
H 0.92052000 1.60537700 -0.17755000
H 0.73556100 1.22263600 1.54208100
H 3.25559700 3.21589100 0.37430200
H 5.50428900 2.15319500 0.59943400
H 5.24481900 2.96838800 -0.92264100
H 4.56835700 -0.56915000 -2.56614900
C 1.81026800 -0.78905100 -1.08811900
H 1.49567700 -0.02781000 -1.80330000
H 1.47867100 -1.76050500 -1.45957100
H -0.26707700 -1.36491000 1.69386800

PROD-DISFAV-DIA

C 0.07165800 -1.25815200 0.45332800
N 0.98210400 -1.17071700 -0.42937000
S 2.47049600 -1.88948500 -0.03309700

O 2.79788100 -2.75399300 -1.15548100
O 2.45014900 -2.46049800 1.30954300
C 3.53503600 -0.47348600 -0.04037400
C 3.97555600 0.03735400 -1.25652100
C 4.78092100 1.16540300 -1.25483400
C 5.15072900 1.78653400 -0.05831600
C 4.68978000 1.25061100 1.14563400
C 3.88229000 0.12202800 1.16522200
H 3.52858400 -0.29179100 2.10145700
H 4.96654900 1.72096000 2.08276200
C 6.05014500 2.99169700 -0.07016400
H 7.09814500 2.67973800 -0.11312600
H 5.85726800 3.61963800 -0.94203500
H 5.91892400 3.59399200 0.83024500
H 5.12887500 1.57067000 -2.19871900
H 3.69600700 -0.44176500 -2.18693500
C -1.27921500 -0.68601400 0.24508100
O -1.46711400 -0.07969000 -1.01406800
C -2.76023100 0.43618500 -0.70956900
C -2.51006200 1.45724400 0.36205200
C -1.70780700 2.65289700 -0.16042400
H -1.45314800 3.32584600 0.66445400
H -2.29495300 3.22085900 -0.88960000
H -0.78147400 2.33209500 -0.64103800
C -3.91978200 1.94557400 0.80891400
H -4.23788700 1.50965200 1.75155500
H -3.86190000 3.02211000 0.99557600
C -5.00876600 1.78016800 -0.31338100
H -4.63194600 2.31300300 -1.19294300
H -5.87075300 2.36828800 0.01718800
C -5.63524800 0.41035200 -0.79781600
H -6.41000800 0.13599100 -0.07885600
H -6.17625600 0.65224400 -1.71762800
C -4.76607900 -0.87237500 -1.06307200
C -3.59024900 -0.78899900 -0.08837700
C -2.40083900 -1.76255200 0.12029600
H -2.52346500 -2.40517100 0.99157900
H -2.14374800 -2.37845100 -0.74468000
C -4.27434800 -0.83877400 1.31884000
H -5.12570400 -0.18438400 1.45530300
H -3.59972800 -0.69451300 2.15823400
H -4.65616900 -1.86156800 1.39985700
H -4.41899800 -0.90636900 -2.09939900
H -5.39300800 -1.75478300 -0.90110900
C -1.57076300 0.53013400 1.23722600
H -1.97480400 0.21859300 2.19643800
H -0.62536300 1.04043600 1.43468500
H -3.18152700 0.81351000 -1.63557600
H 0.24015000 -1.75357500 1.41500100

PROD-(5+2)-FAV

C -0.26587100 -0.82644700 -1.40296600
C -1.55904200 -0.83743000 -1.71971500
C -2.35227200 0.22951500 -2.37982300
H -1.75127000 0.99103300 -2.87562100
H -3.08716400 -0.17578700 -3.07815300
C -3.05666500 0.77486500 -1.07470400
C -2.37937300 2.09485400 -0.67506700
H -1.29079100 1.96826900 -0.69702700
C -2.78512200 2.71537000 0.66439200
C -2.08318200 2.12630000 1.88463400
H -2.36856200 2.71400100 2.76343200
C -2.40727200 0.66528600 2.19699300
H -3.49035200 0.56939500 2.34895500
H -1.94922900 0.41756100 3.16130500
C -1.98713000 -0.43318300 1.19686400
C -2.22847200 -1.78841400 1.88990700
H -1.93806400 -2.61603200 1.24259700
H -1.64413700 -1.85063300 2.81204100
H -3.28402500 -1.90625500 2.15220100
C -0.46123800 -0.32545200 0.92991400
N 0.06085000 -1.27104200 -0.08882800
S 1.57711200 -1.90466100 0.15204600
O 1.82405100 -2.79905500 -0.96599600
O 1.61487900 -2.40434600 1.51644100
C 2.71157200 -0.53991700 0.03279800
C 2.96471700 0.23790200 1.15848300
C 3.78810300 1.34735800 1.03910300
C 4.36533500 1.69003800 -0.18621700
C 4.09664400 0.88990300 -1.29820200
C 3.27411700 -0.22372100 -1.19895300
H 3.08057200 -0.84675200 -2.06359700
H 4.53919200 1.13802100 -2.25681800
C 5.28137700 2.87797100 -0.29572000
H 4.98976400 3.66735100 0.39954500
H 5.28150400 3.28647600 -1.30772000
H 6.30829900 2.58706800 -0.05425900
H 3.98893500 1.95533300 1.91458400
H 2.53354600 -0.02642200 2.11626400
H 0.03767700 -0.54024200 1.87725000
H -0.17777100 0.68429000 0.62450400
C -2.90361300 -0.43702500 -0.06209900
H -3.89742700 -0.71995400 0.29328300
O -2.43601800 -1.51014000 -0.89958300
H -1.00111400 2.26388800 1.77960600
H -3.87051700 2.64941000 0.80056800
H -2.55412800 3.78450000 0.62464500
H -2.61004400 2.80894200 -1.47329000
C -4.54797200 0.99544900 -1.34669200
H -5.01986800 0.06432200 -1.67267200
H -4.68781400 1.74122800 -2.13476000
H -5.07395600 1.34126700 -0.45297700
H 0.46586200 -0.26943000 -1.97929000

PROD-(5+2)-DISFAV

C -1.10014200 -0.78094500 1.49185700
C -1.68854600 0.40978400 1.58314100
C -3.14711800 0.71920000 1.63661900
H -3.76448200 -0.16997000 1.75340800
H -3.39847300 1.43521400 2.42149000

C -3.27754900 1.40152300 0.21931300
C -3.86336300 2.81007700 0.40775200
H -3.98174200 3.31333000 -0.55698300
H -3.21046800 3.42158900 1.03692600
H -4.84483000 2.75713200 0.88733100
C -4.17718600 0.66544800 -0.79168900
H -4.02569500 1.13455100 -1.77283500
H -5.20548000 0.91719900 -0.50993600
C -4.14585500 -0.85944500 -0.96262400
H -4.17725100 -1.35085000 0.01666300
H -5.09863600 -1.11171900 -1.43907800
C -3.04407700 -1.51746500 -1.80732900
C -1.75847900 -1.87781900 -1.05986300
H -2.05458200 -2.23229500 -0.07280100
H -1.29961400 -2.73996400 -1.55125400
C -0.63864400 -0.80338000 -0.91425100
N -0.09324800 -0.91439300 0.49501400
S 1.36216400 -1.55502900 0.93350300
O 1.63874000 -2.82421800 0.27001900
O 1.35488000 -1.55135700 2.39223100
C 2.55496400 -0.36962900 0.35050600
C 3.73968700 -0.81721200 -0.21632400
C 4.67309200 0.11727800 -0.64628300
C 4.43434600 1.48620200 -0.51794200
C 3.22830900 1.90451800 0.05262200
C 2.28560400 0.98756100 0.49119900
H 1.33755200 1.31653600 0.90296500
H 3.02067300 2.96519500 0.14660000
C 5.45793700 2.49363600 -0.96565400
H 6.12067400 2.07607900 -1.72553300
H 4.98076600 3.38759900 -1.37215300
H 6.07670600 2.80634900 -0.11893100
H 5.59815200 -0.22566900 -1.09703200
H 3.92082700 -1.87812000 -0.33607800
C 0.42953900 -1.11117500 -1.97529500
H 1.25834900 -0.40169400 -1.95260600
H -0.05293400 -1.02817900 -2.95262800
H 0.82261600 -2.12193200 -1.87667100
C -1.07242700 0.66792600 -1.22695800
H -1.63439800 0.65460700 -2.16333200
H -0.14449100 1.20186200 -1.45083800
C -1.78388200 1.58609800 -0.22495000
H -1.65489800 2.60254400 -0.60295400
O -1.06177000 1.49182500 1.00965600
H -2.84132800 -0.92382900 -2.70576300
H -3.45604500 -2.46164000 -2.17653100
H -1.52492900 -1.66577700 1.94907100

7. ^{1}H, ^{13}C NMR and 2D-NMR Spectra

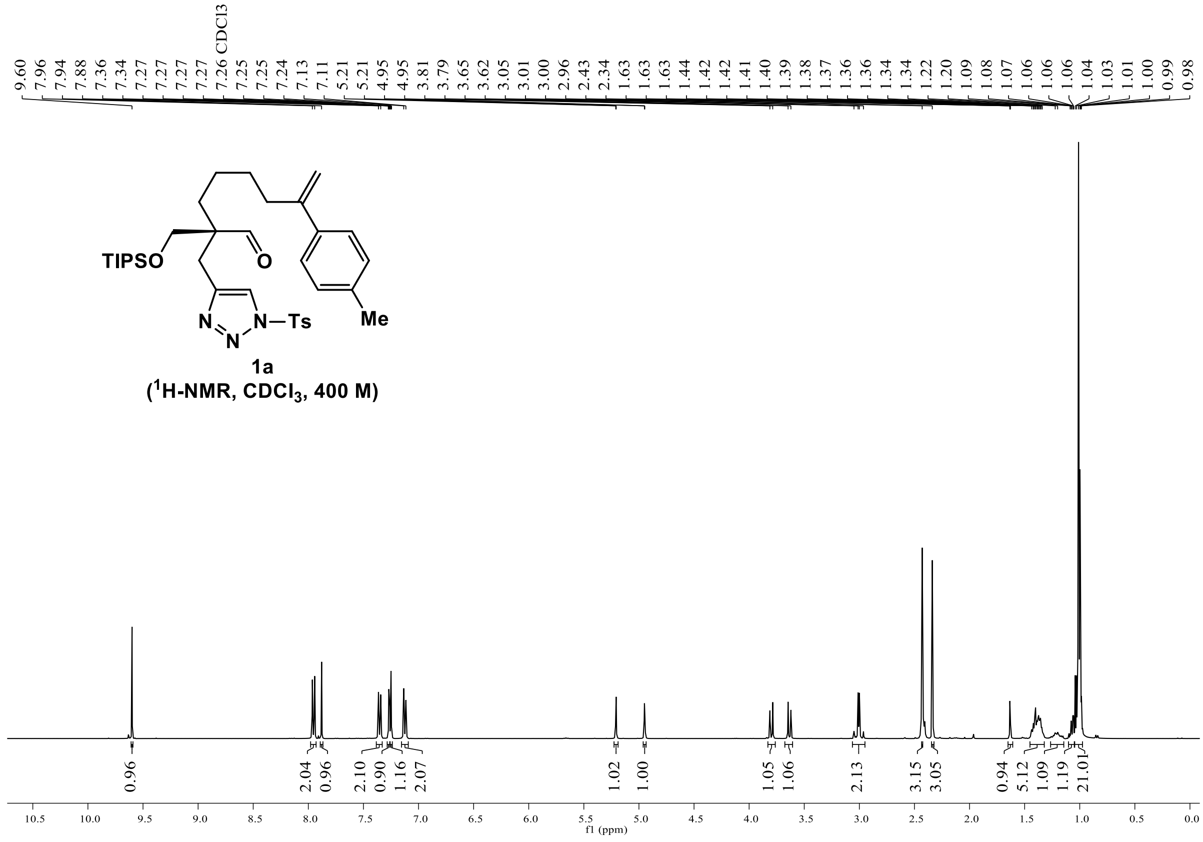

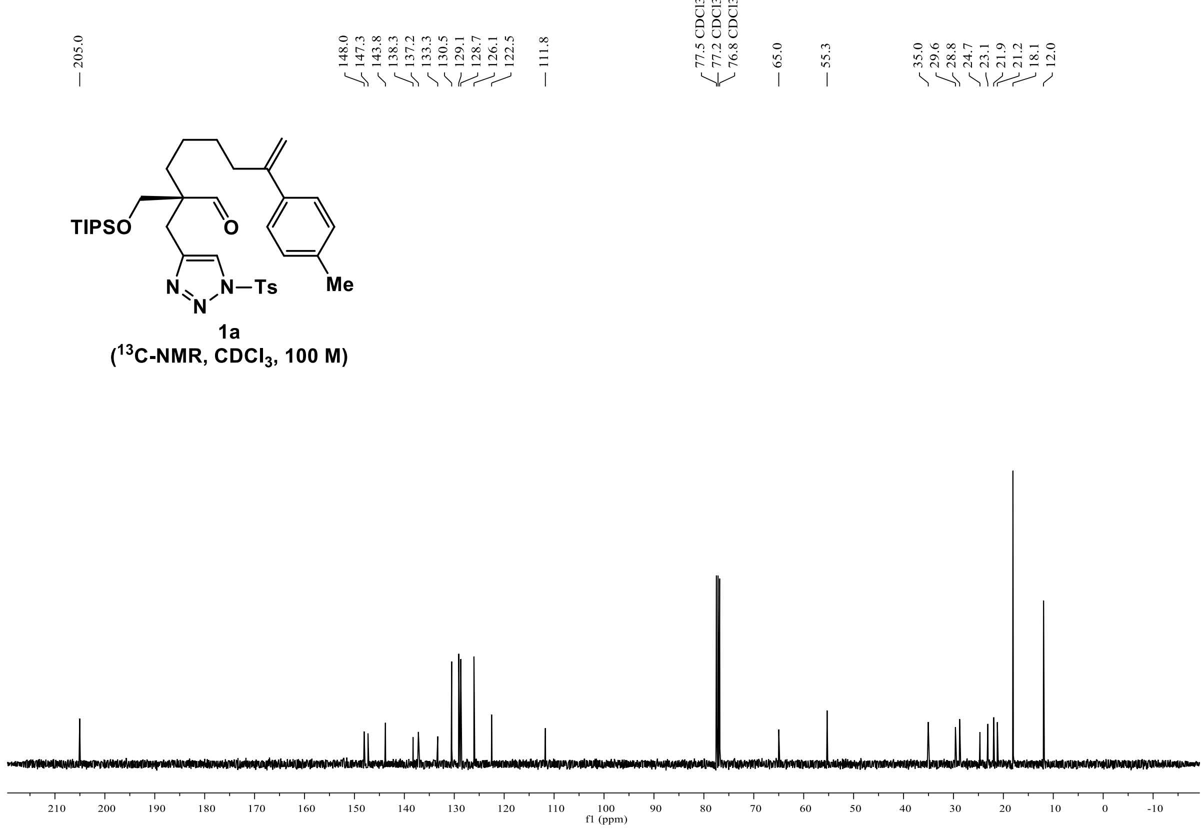

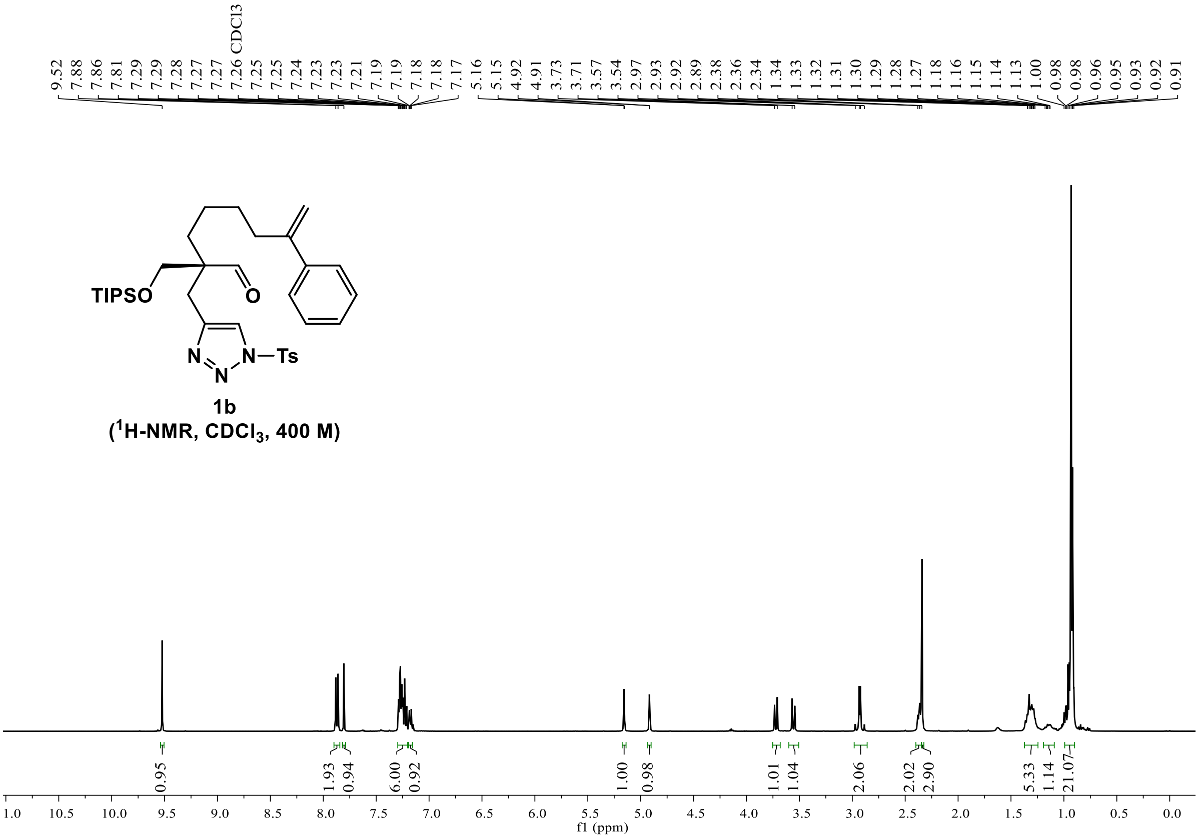
9.52 7.88 7.86 7.81 7.29 7.29 7.28 7.27 7.27 7.26 CDCl3 7.25 7.25 7.24 7.23 7.23 7.21 7.19 7.18 7.18 7.17 5.16 5.15 4.92 4.91 3.73 3.71 3.57 3.54 2.97 2.93 2.92 2.89 2.38 2.36 2.34 1.34 1.33 1.32 1.31 1.30 1.29 1.28 1.27 1.18 1.16 1.15 1.14 1.13 1.00 0.98 0.98 0.96 0.95 0.93 0.92 0.91
TIPSO
O
N
N
N—Ts
1b
(1H-NMR, CDCl3, 400 M)
0.95 1.93 0.94 6.00 0.92 1.00 0.98 1.01 1.04 2.06 2.02 2.90 5.33 1.14 21.07
f1 (ppm)

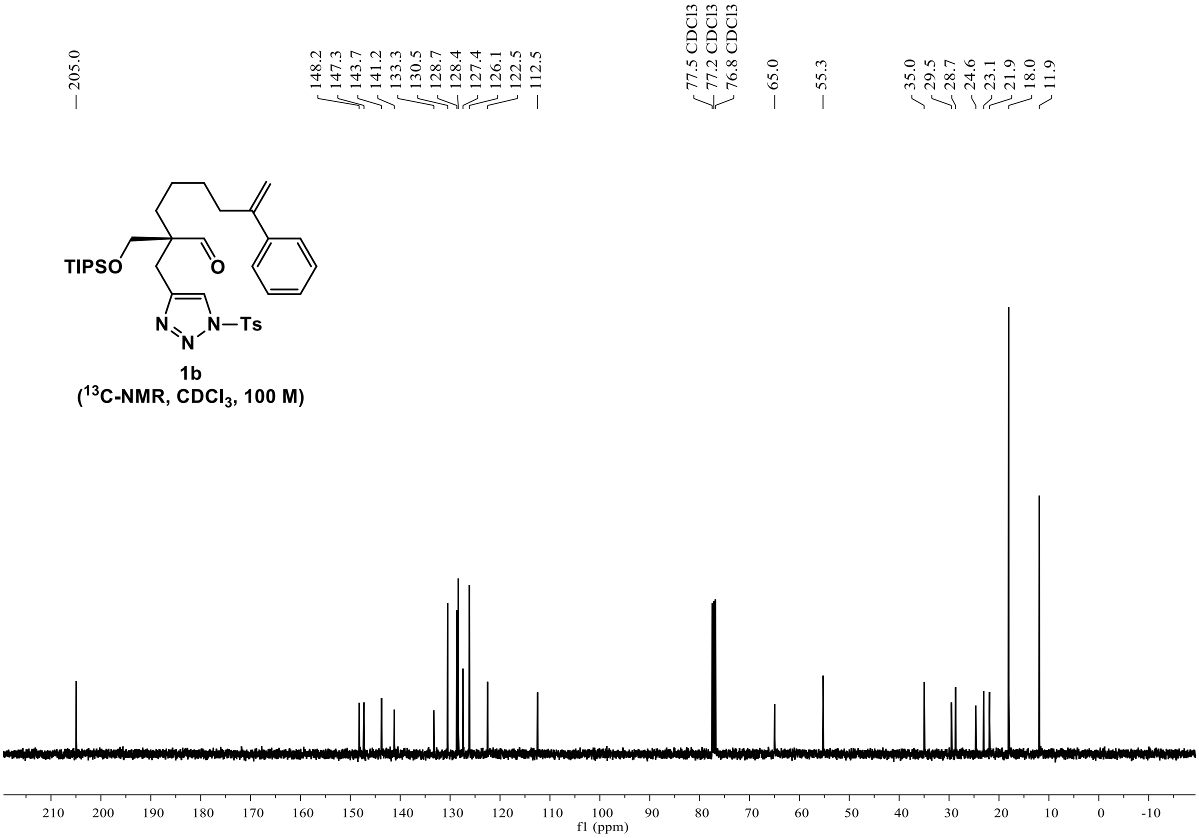
205.0 148.2 147.3 143.7 141.2 133.3 130.5 128.7 128.4 127.4 126.1 122.5 112.5 77.5 CDCl3 77.2 CDCl3 76.8 CDCl3 65.0 55.3 35.0 29.5 28.7 24.6 23.1 21.9 18.0 11.9
TIPSO
O
N
N
N—Ts
1b
(13C-NMR, CDCl3, 100 M)
f1 (ppm)

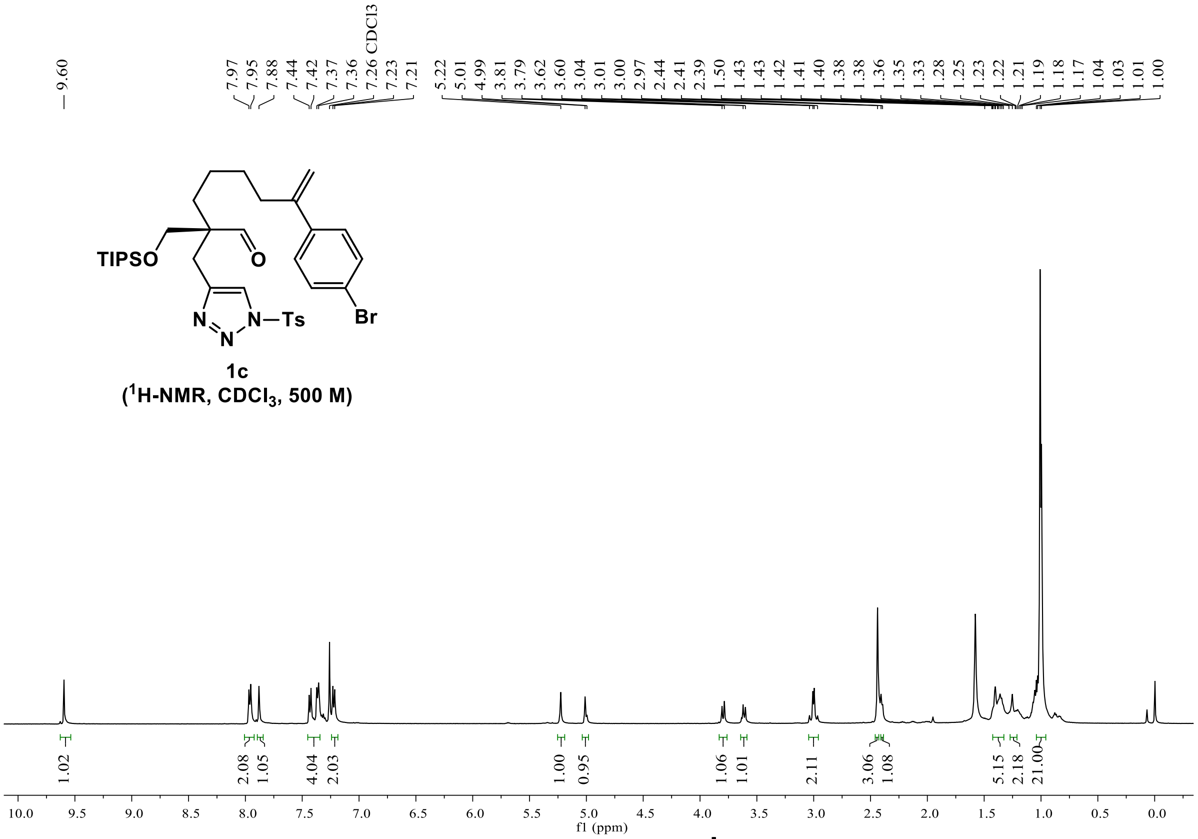
TIPSO
O
N
N
N—Ts
Br
1c
(¹H-NMR, CDCl₃, 500 M)
f1 (ppm)

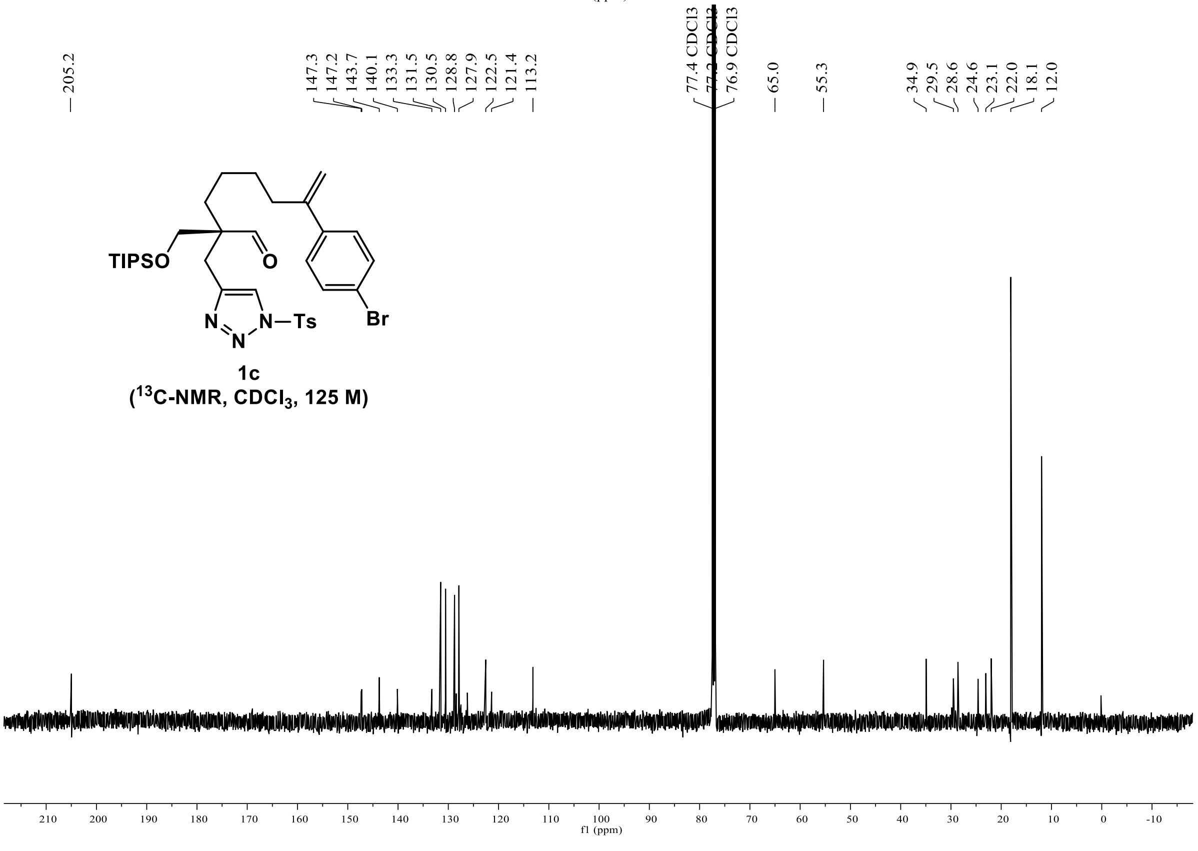
TIPSO
O
N
N
N—Ts
Br
1c
(¹³C-NMR, CDCl₃, 125 M)
f1 (ppm)

1d
(^{1}H-NMR, $CDCl_3$, 400 M)

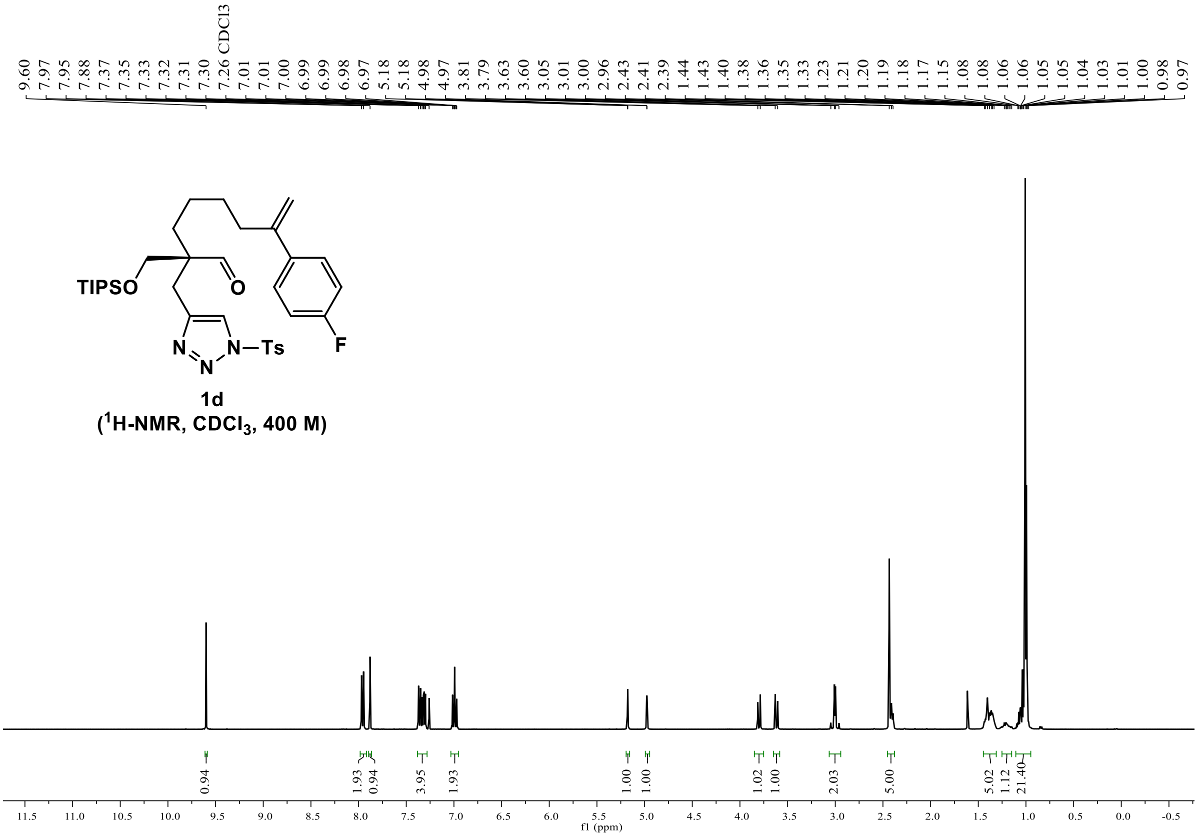

1d
(^{13}C-NMR, $CDCl_3$, 100 M)

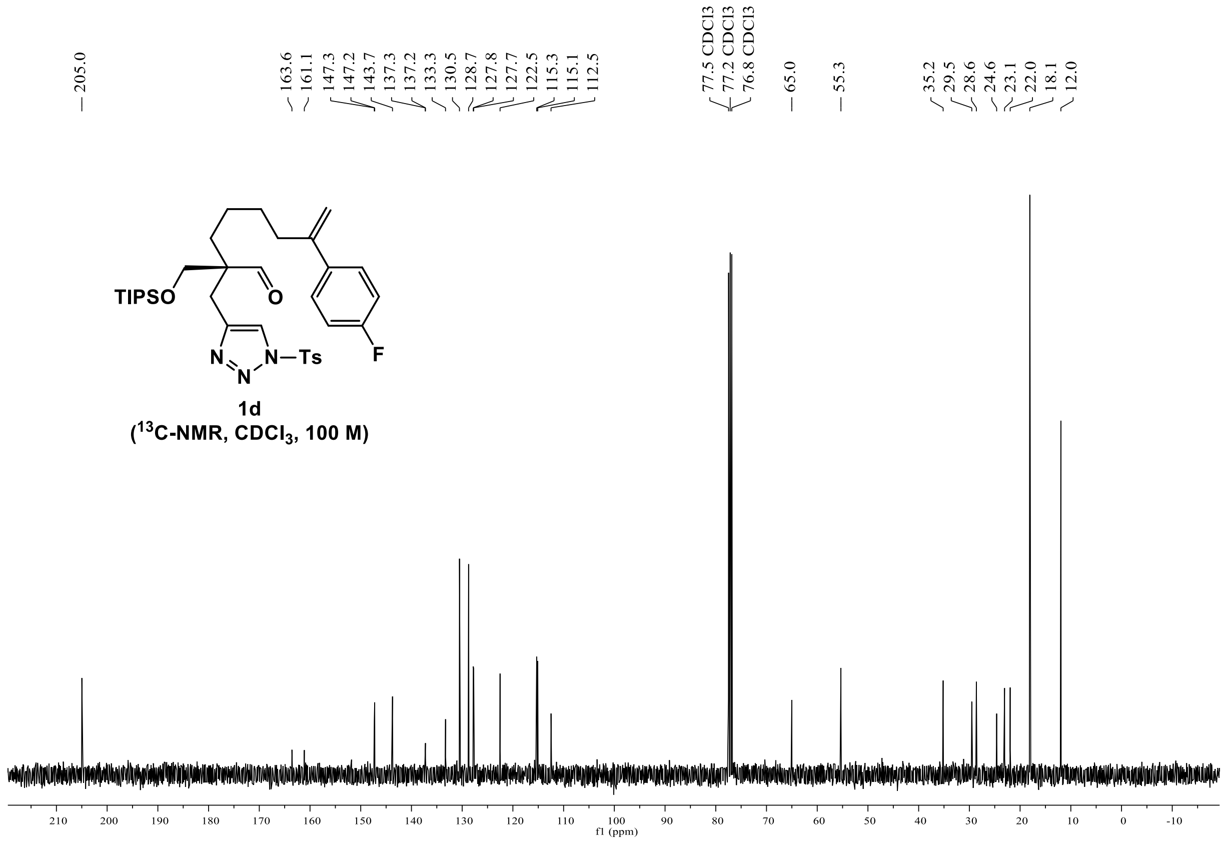

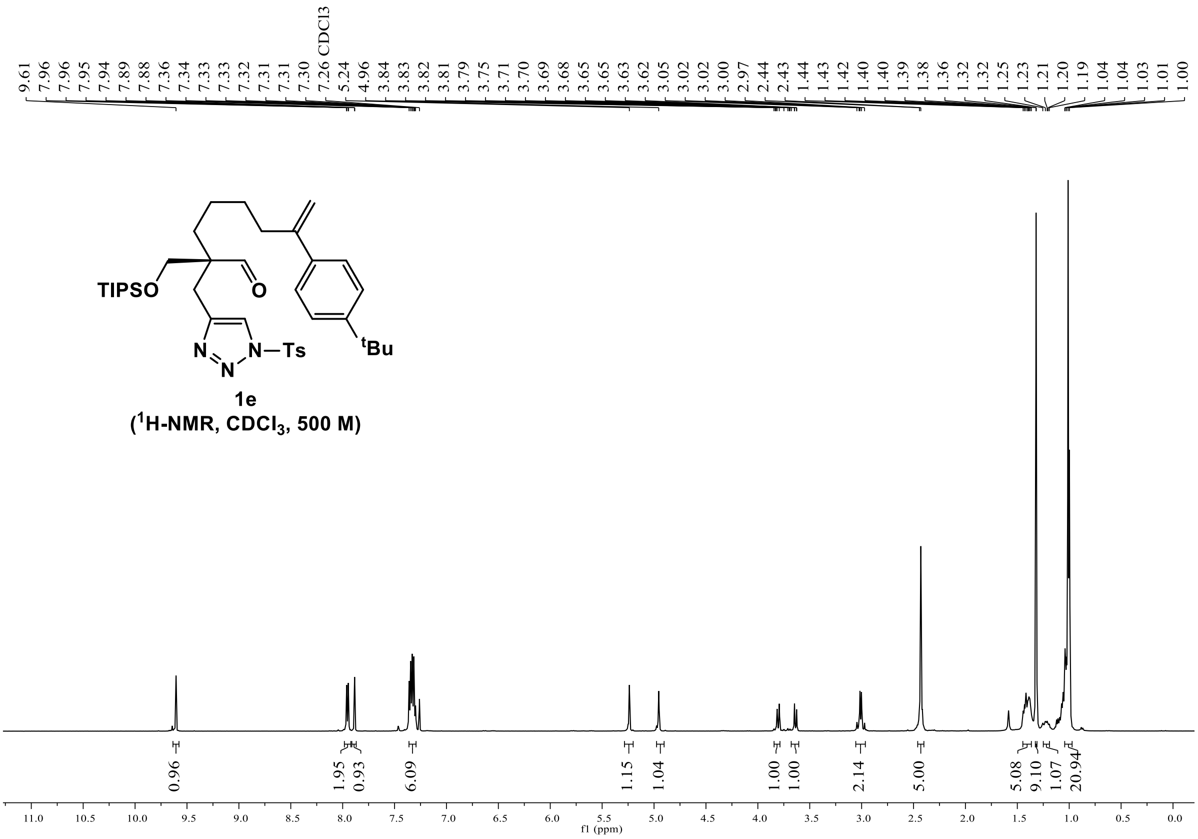
9.61 7.96 7.96 7.95 7.94 7.89 7.88 7.36 7.34 7.33 7.33 7.32 7.31 7.31 7.30 7.26 CDCl3 5.24 4.96 3.84 3.83 3.82 3.81 3.79 3.75 3.71 3.70 3.69 3.68 3.65 3.65 3.63 3.62 3.05 3.02 3.02 3.00 2.97 2.44 2.43 1.44 1.43 1.42 1.40 1.40 1.39 1.38 1.36 1.32 1.32 1.25 1.23 1.21 1.20 1.19 1.04 1.04 1.03 1.01 1.00
TIPSO
O
N
N
N—Ts
tBu
1e
(^{1}H-NMR, $CDCl_3$, 500 M)
0.96
1.95
0.93
6.09
1.15
1.04
1.00
1.00
2.14
5.00
5.08
9.10
1.07
20.94
f1 (ppm)

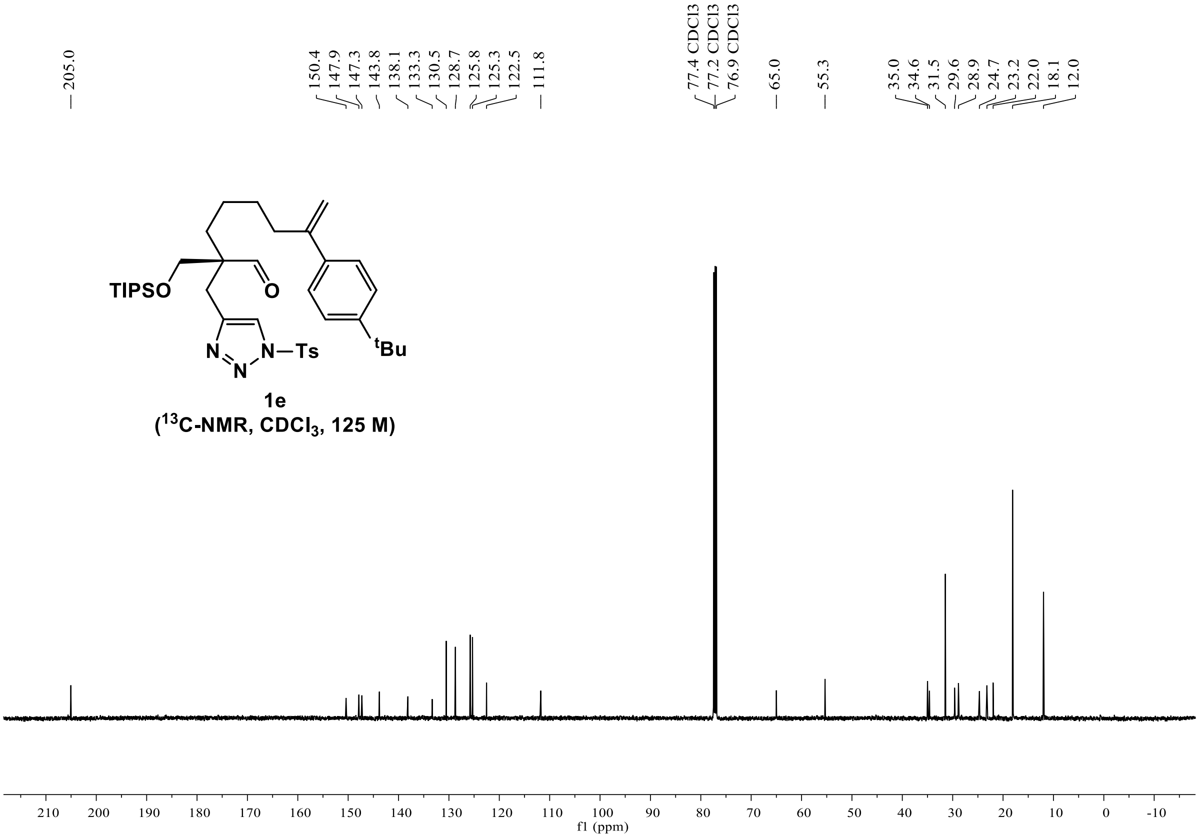
205.0
150.4 147.9 147.3 143.8 138.1 133.3 130.5 128.7 125.8 125.3 122.5 111.8
77.4 CDCl3 77.2 CDCl3 76.9 CDCl3
65.0
55.3
35.0 34.6 31.5 29.6 28.9 24.7 23.2 22.0 18.1 12.0
TIPSO
O
N
N
N—Ts
tBu
1e
(^{13}C-NMR, $CDCl_3$, 125 M)
f1 (ppm)

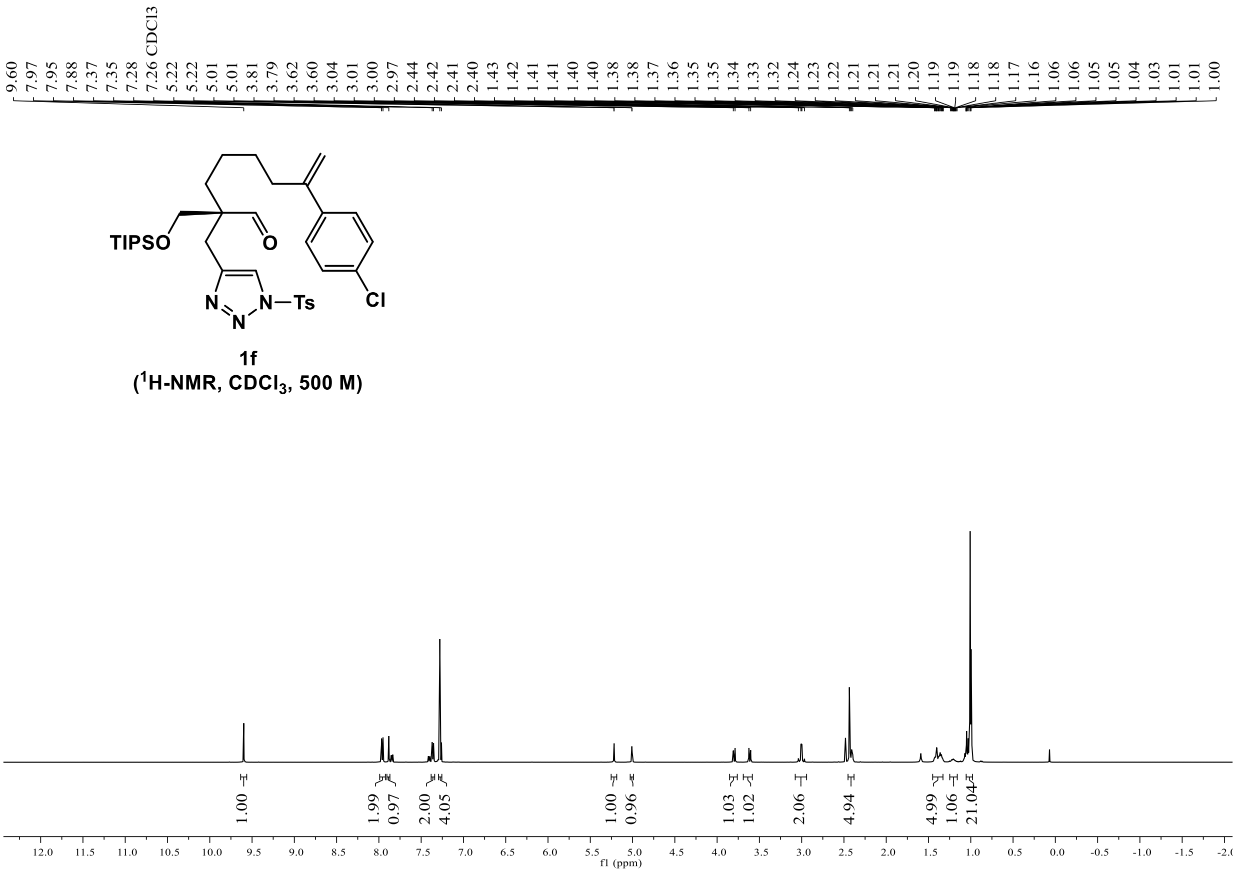

1f
(^{1}H-NMR, $CDCl_3$, 500 M)

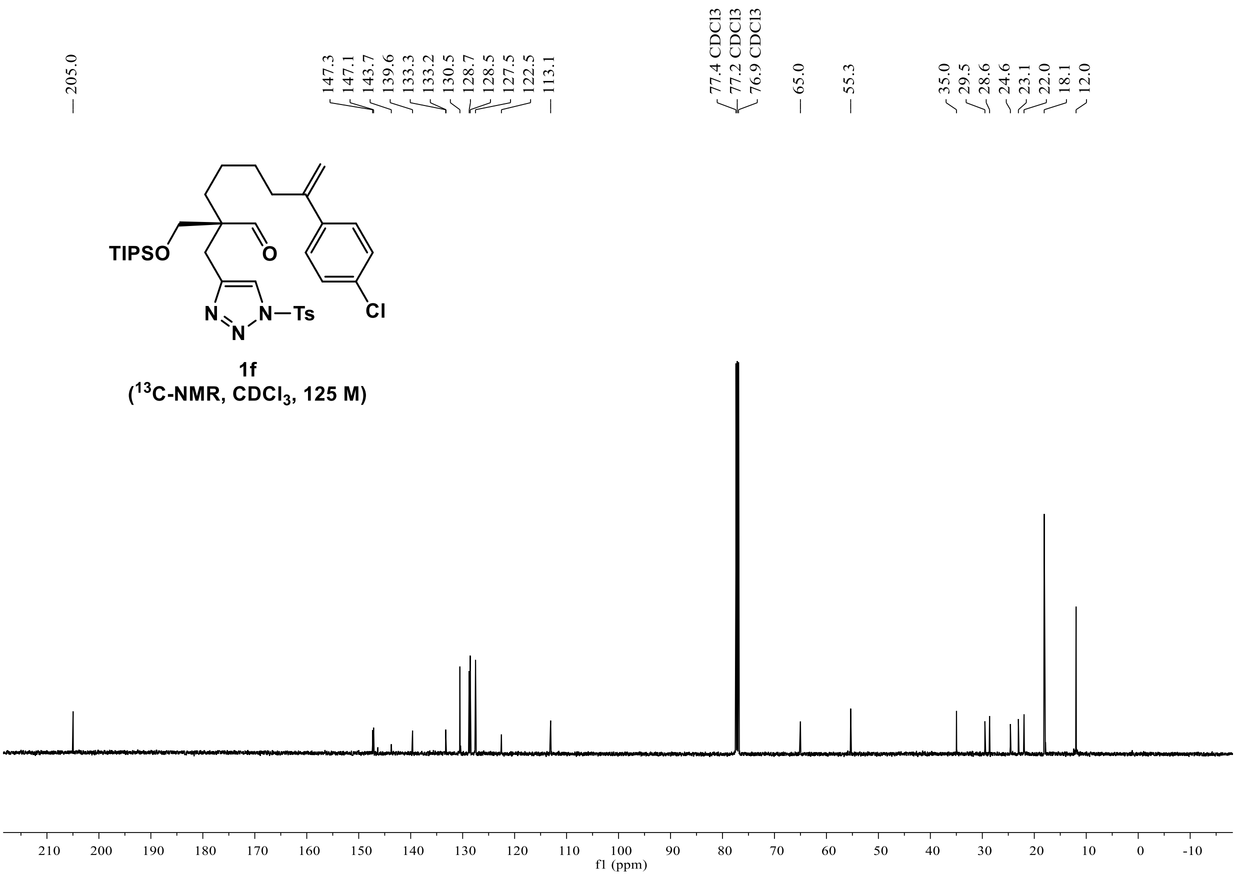

1f
(^{13}C-NMR, $CDCl_3$, 125 M)

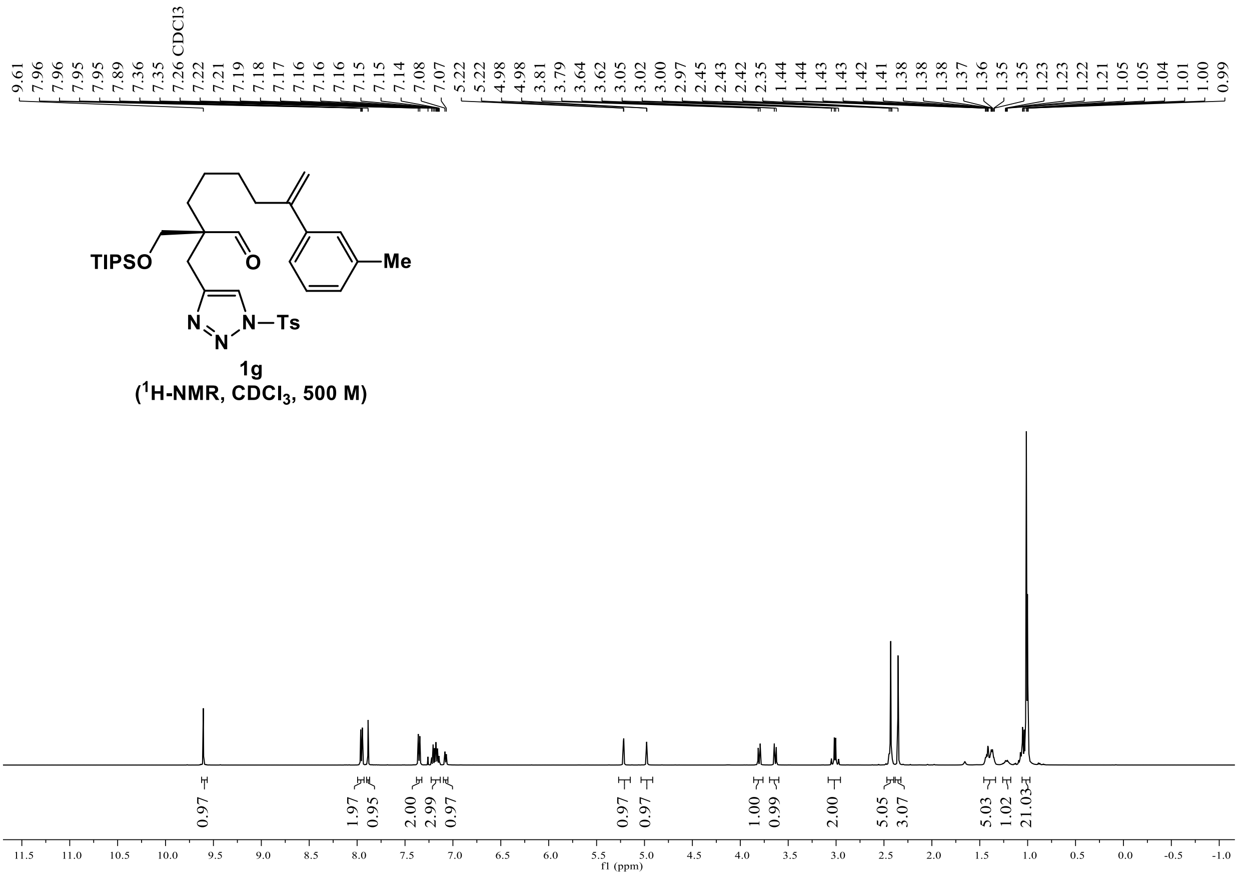

1g
(^{1}H-NMR, CDCl$_3$, 500 M)

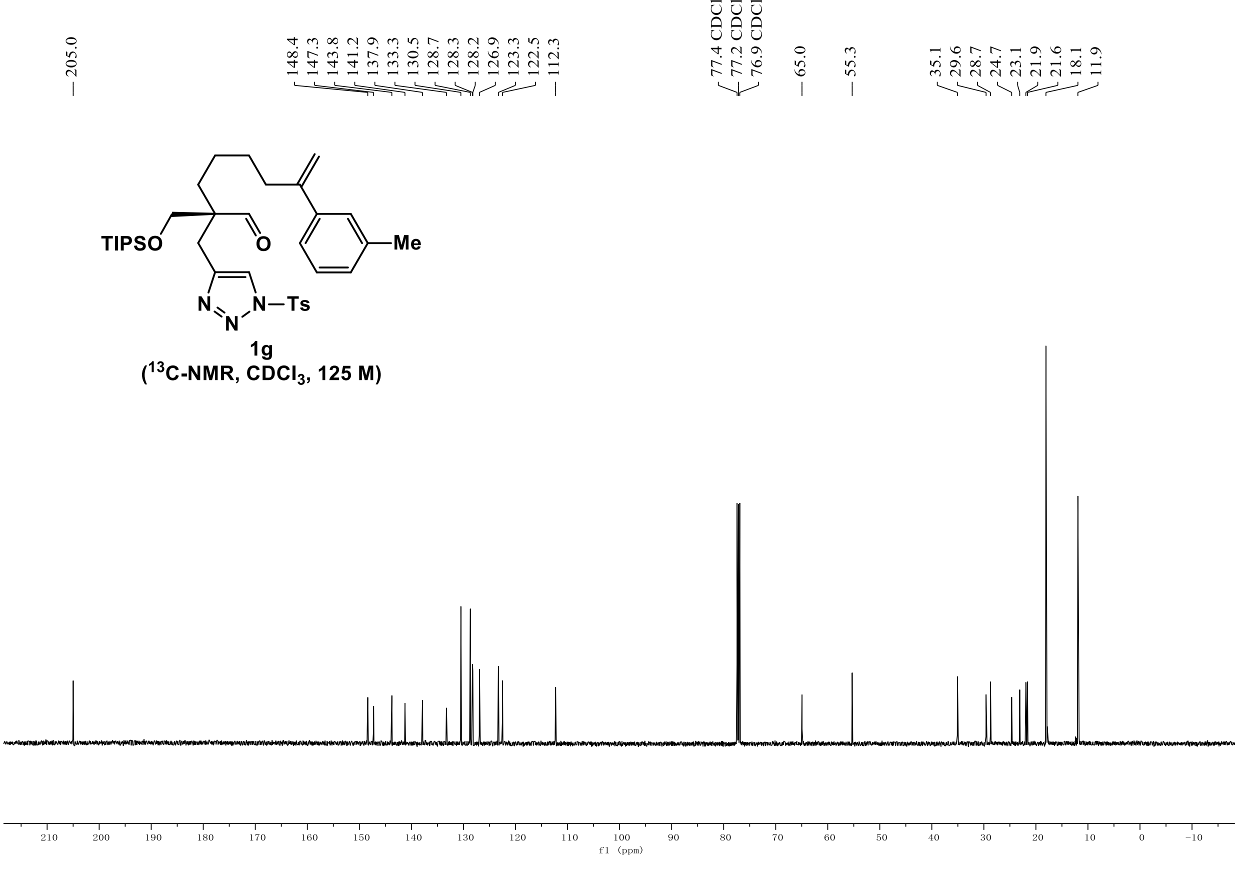

1g
(^{13}C-NMR, CDCl$_3$, 125 M)

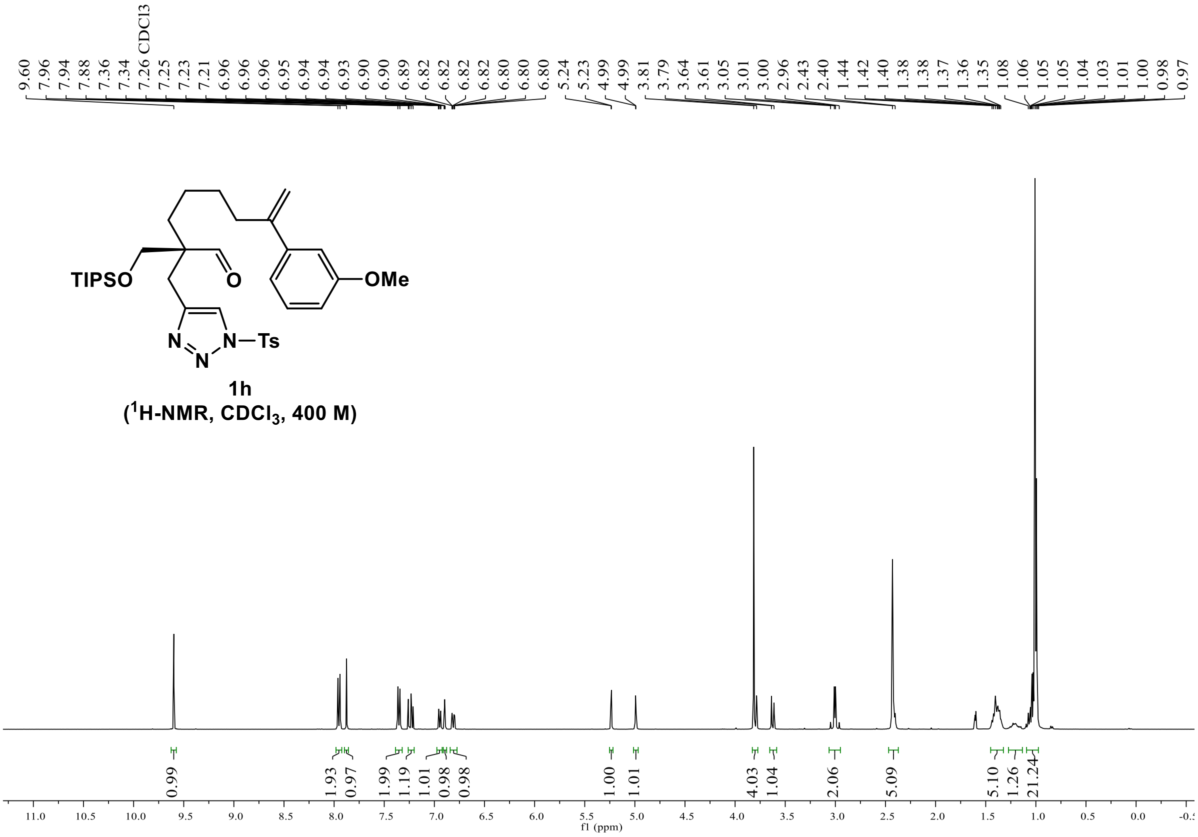
9.60 7.96 7.94 7.88 7.36 7.34 7.26 CDCl3 7.25 7.23 7.21 6.96 6.96 6.95 6.94 6.94 6.93 6.90 6.90 6.89 6.82 6.82 6.82 6.82 6.80 6.80 6.80 5.24 5.23 4.99 4.99 3.81 3.79 3.64 3.61 3.05 3.01 3.00 2.96 2.43 2.40 1.44 1.42 1.40 1.38 1.37 1.36 1.35 1.08 1.06 1.05 1.05 1.04 1.03 1.01 1.00 0.98 0.97
TIPSO
O
OMe
N
N
N—Ts
1h
(1H-NMR, CDCl3, 400 M)
0.99 1.93 0.97 1.99 1.19 1.01 0.98 0.98 1.00 1.01 4.03 1.04 2.06 5.09 5.10 1.26 21.24
f1 (ppm)

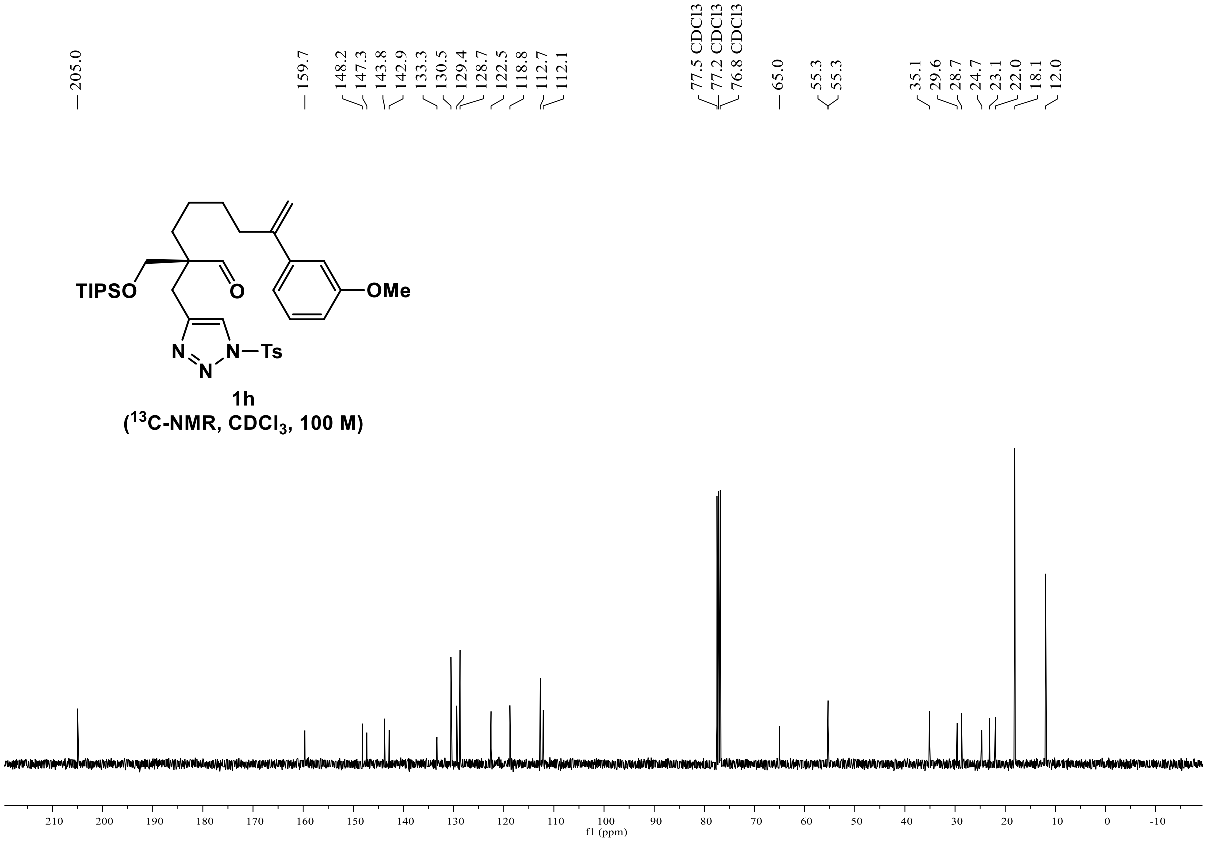
205.0 159.7 148.2 147.3 143.8 142.9 133.3 130.5 129.4 128.7 122.5 118.8 112.7 112.1 77.5 CDCl3 77.2 CDCl3 76.8 CDCl3 65.0 55.3 55.3 35.1 29.6 28.7 24.7 23.1 22.0 18.1 12.0
TIPSO
O
OMe
N
N
N—Ts
1h
(13C-NMR, CDCl3, 100 M)
f1 (ppm)

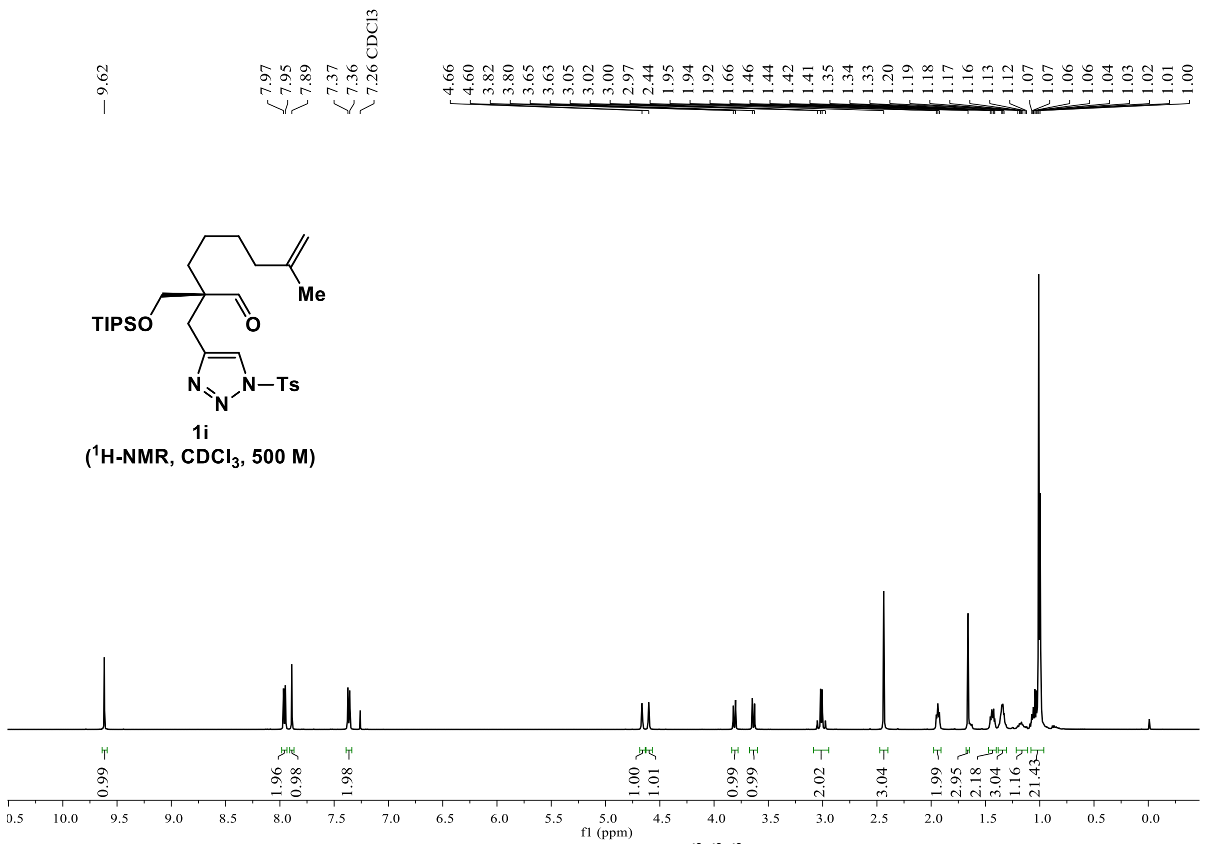
9.62
7.97
7.95
7.89
7.37
7.36
7.26 CDCl3
4.66
4.60
3.82
3.80
3.65
3.63
3.05
3.02
3.00
2.97
2.44
1.95
1.94
1.92
1.66
1.46
1.44
1.42
1.41
1.35
1.34
1.33
1.20
1.19
1.18
1.17
1.16
1.13
1.12
1.07
1.07
1.06
1.06
1.04
1.03
1.02
1.01
1.00
Me
TIPSO
O
N
N
N—Ts
1i
(^{1}H-NMR, $CDCl_3$, 500 M)
0.99
1.96
0.98
1.98
1.00
1.01
0.99
0.99
2.02
3.04
1.99
2.95
2.18
3.04
1.16
21.43
f1 (ppm)

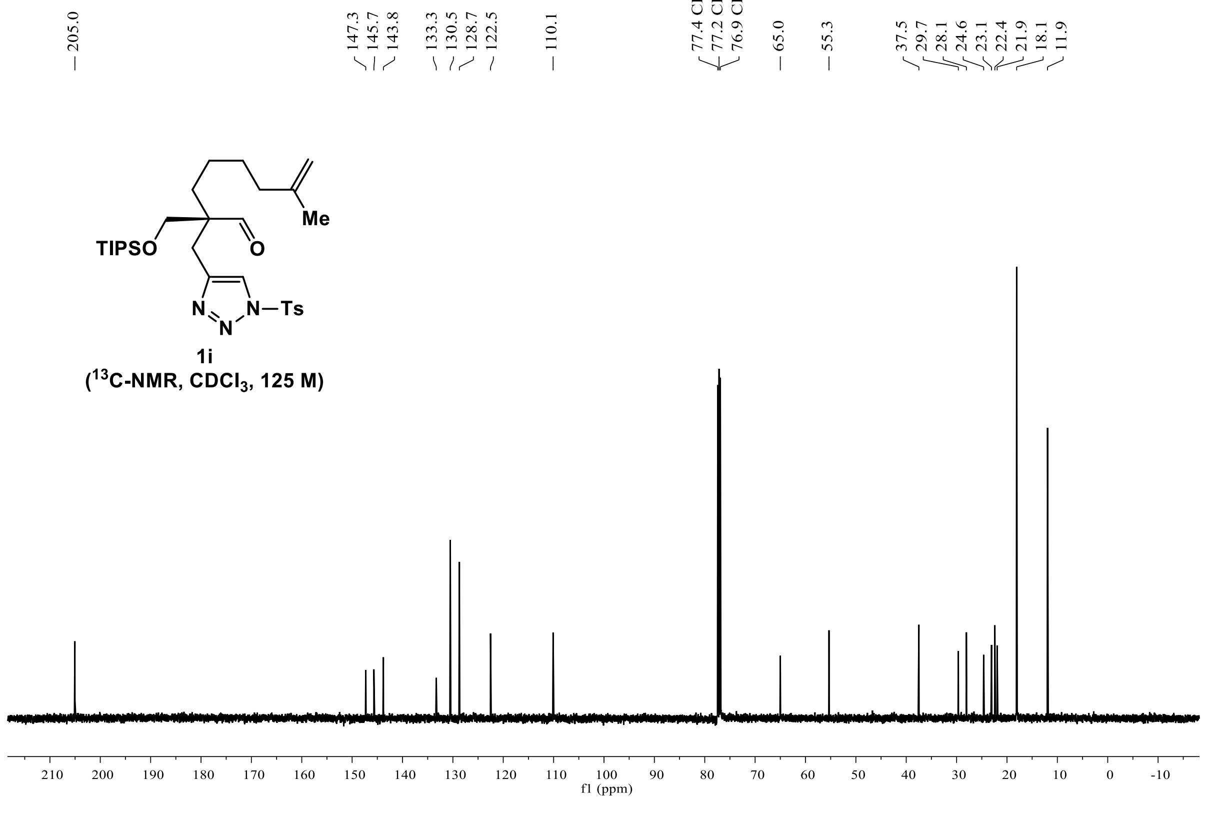
205.0
147.3
145.7
143.8
133.3
130.5
128.7
122.5
110.1
77.4 CDCl3
77.2 CDCl3
76.9 CDCl3
65.0
55.3
37.5
29.7
28.1
24.6
23.1
22.4
21.9
18.1
11.9
Me
TIPSO
O
N
N
N—Ts
1i
(^{13}C-NMR, $CDCl_3$, 125 M)
f1 (ppm)

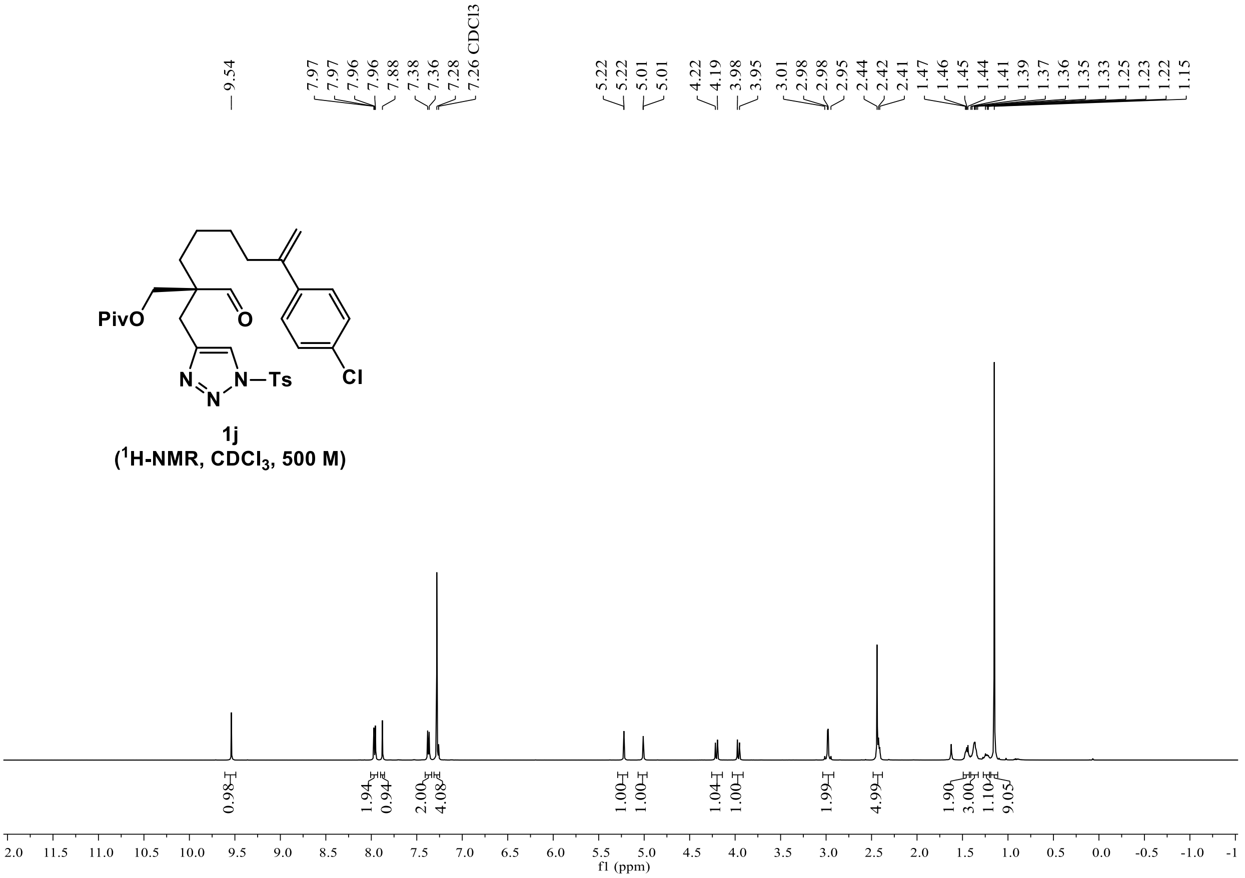

1j

(^{1}H-NMR, $CDCl_3$, 500 M)

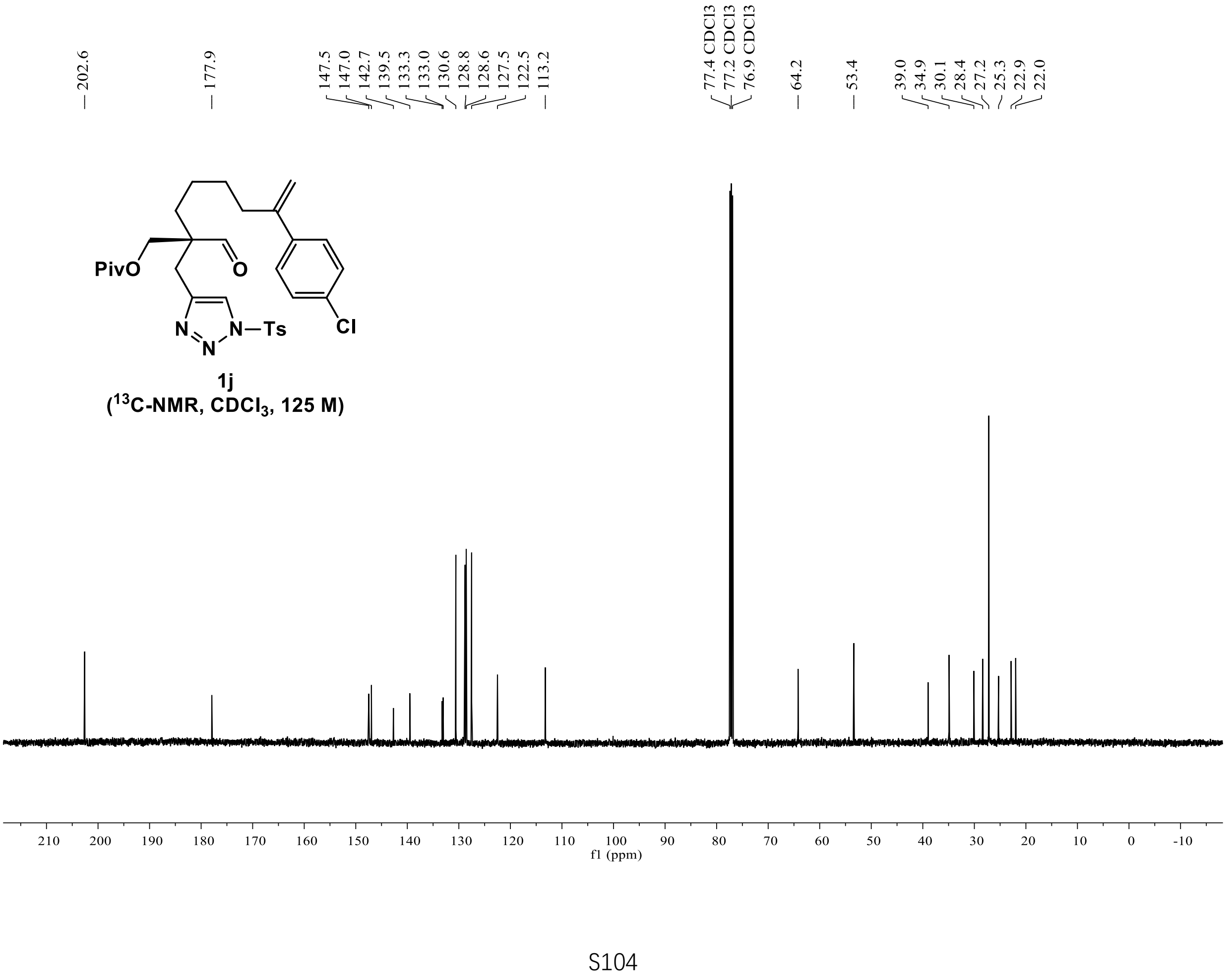

1j

(^{13}C-NMR, $CDCl_3$, 125 M)

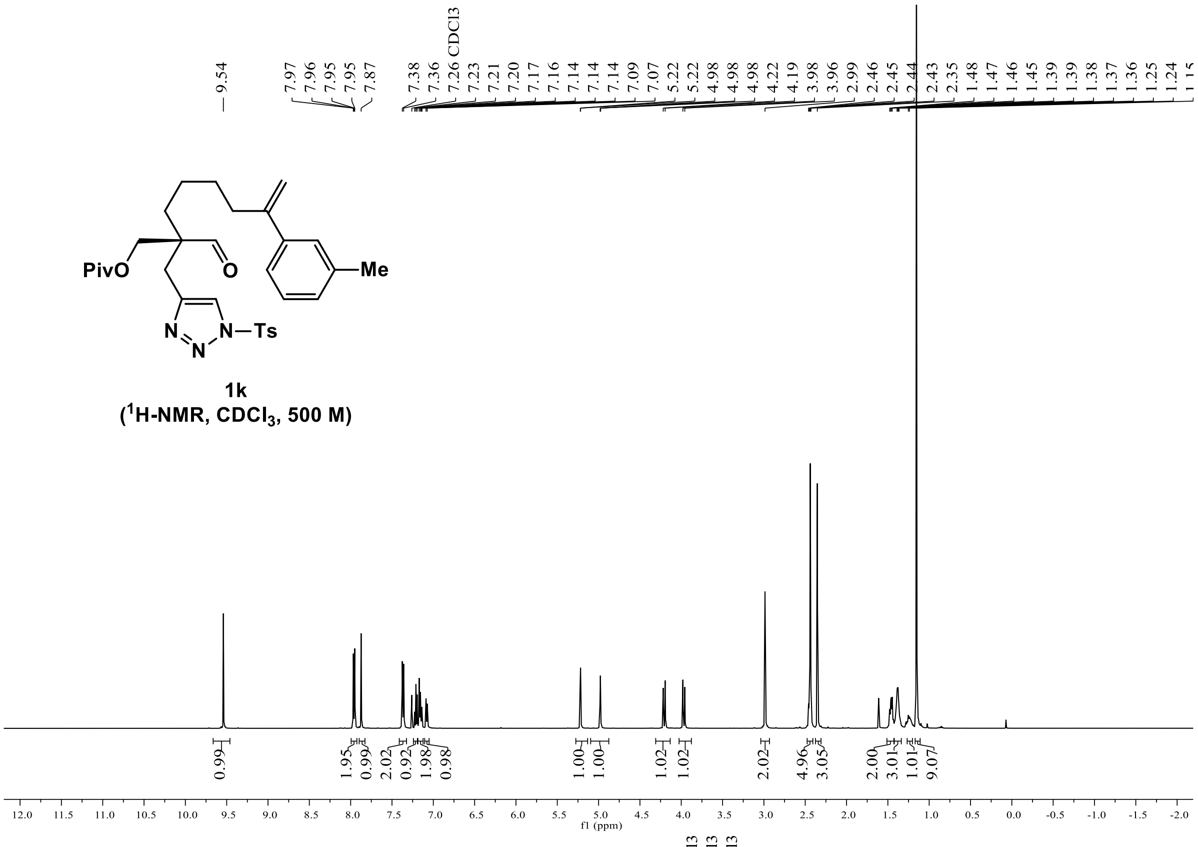

1k
(^{1}H-NMR, $CDCl_3$, 500 M)

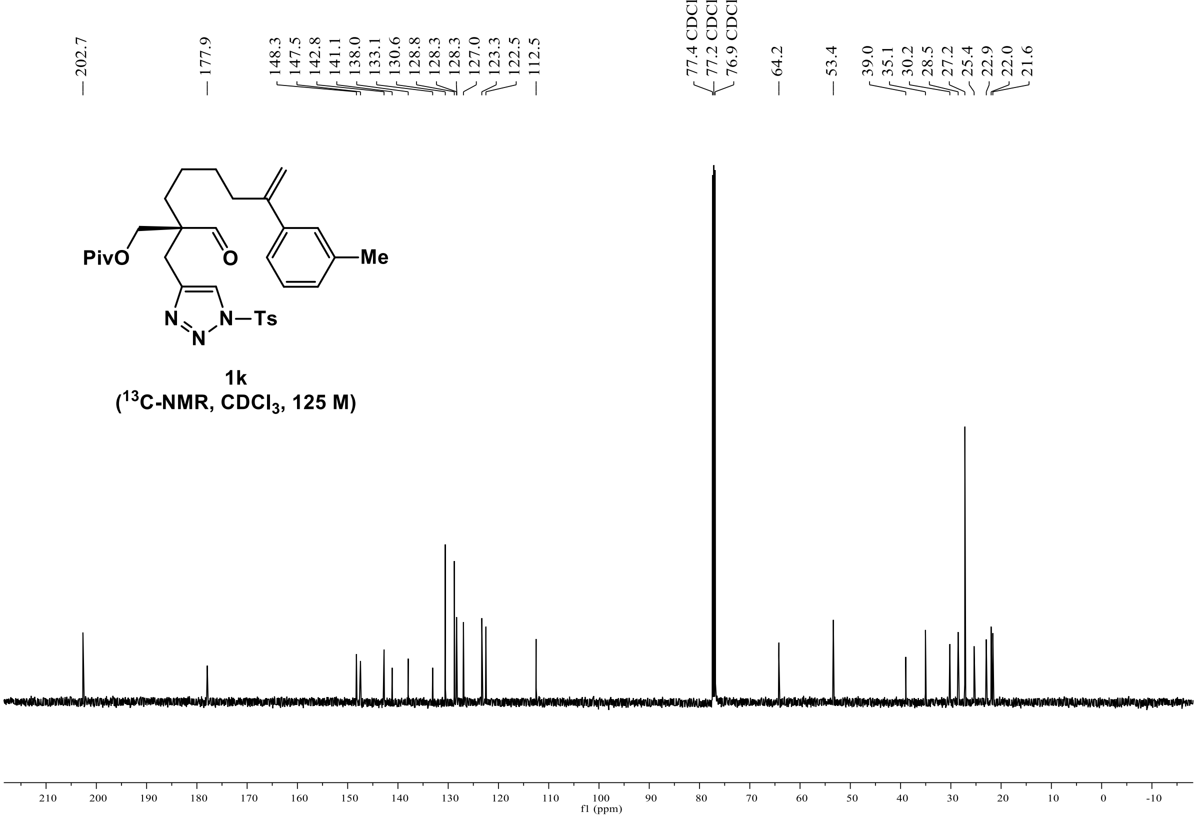

1k
(^{13}C-NMR, $CDCl_3$, 125 M)

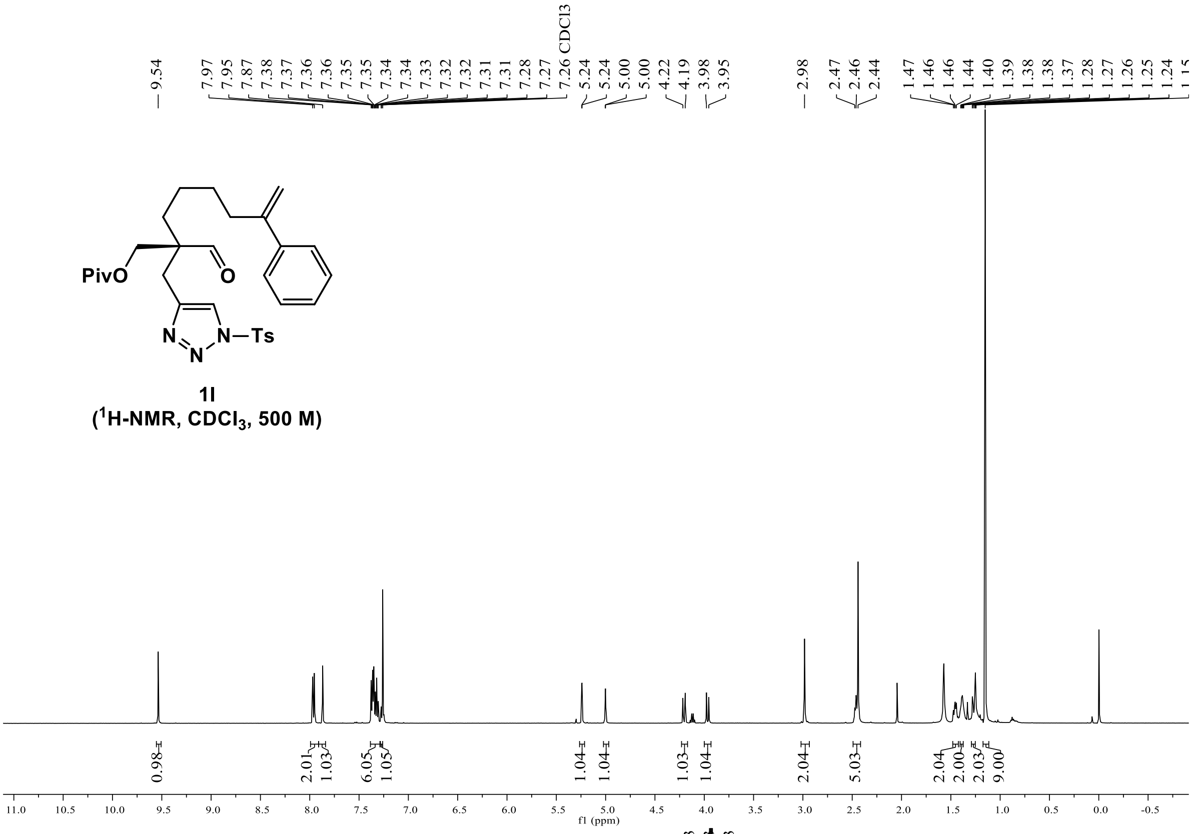

1l
(^{1}H-NMR, CDCl$_3$, 500 M)

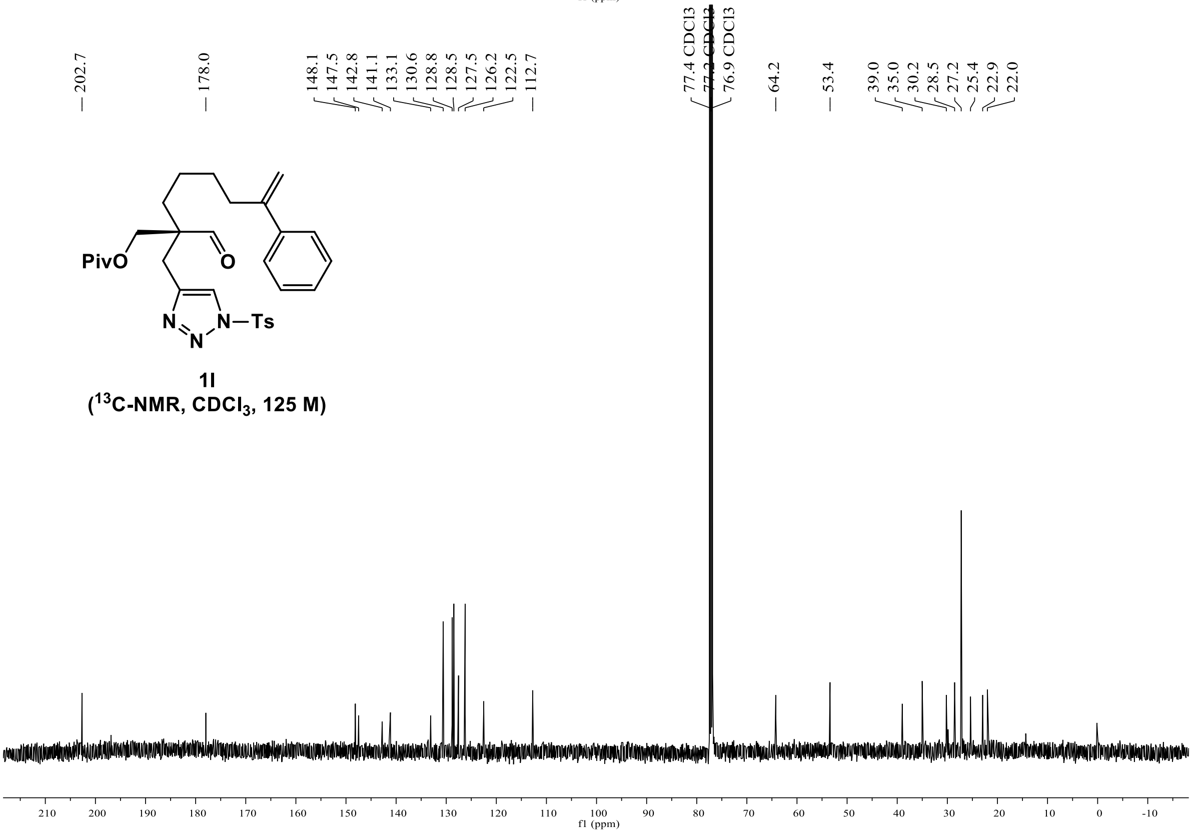

1l
(^{13}C-NMR, CDCl$_3$, 125 M)

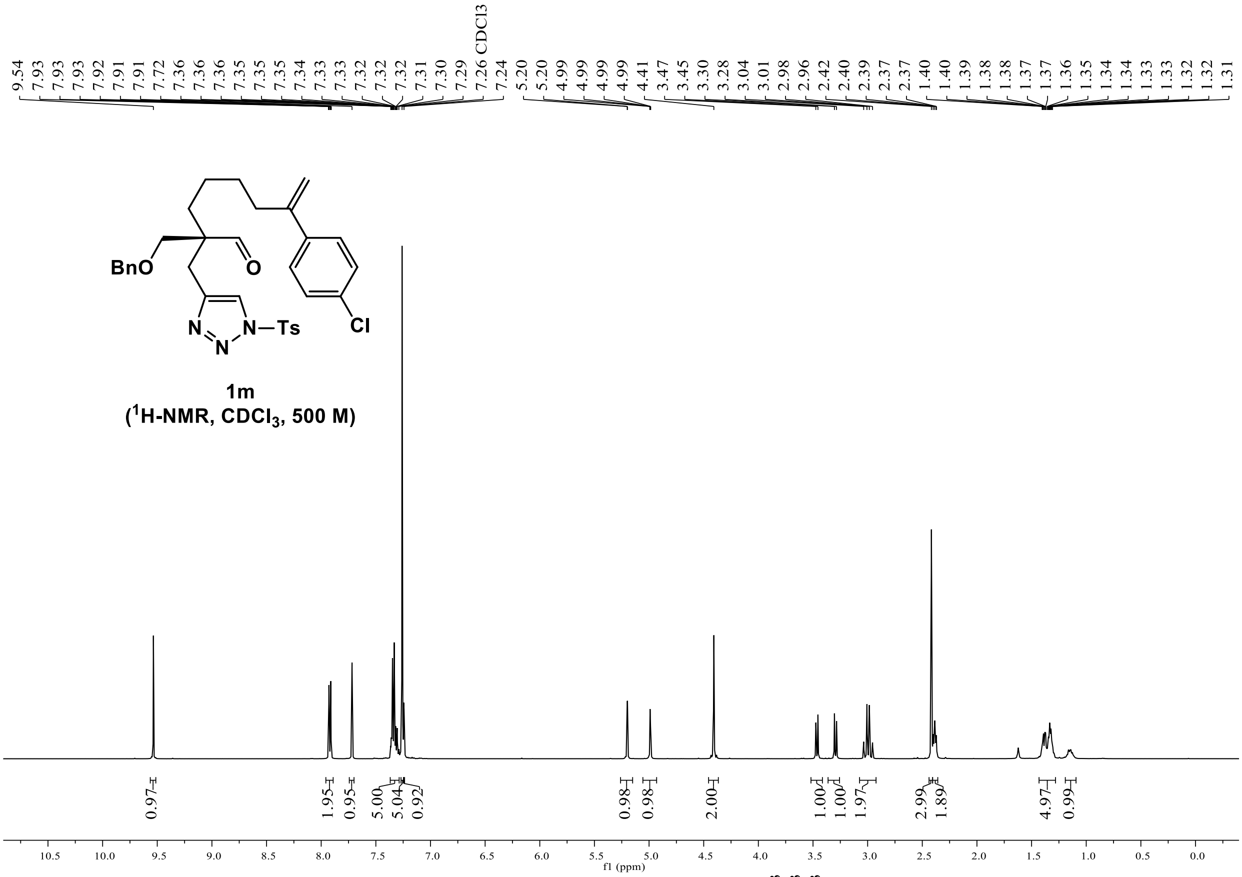

1m
(^{1}H-NMR, $CDCl_3$, 500 M)

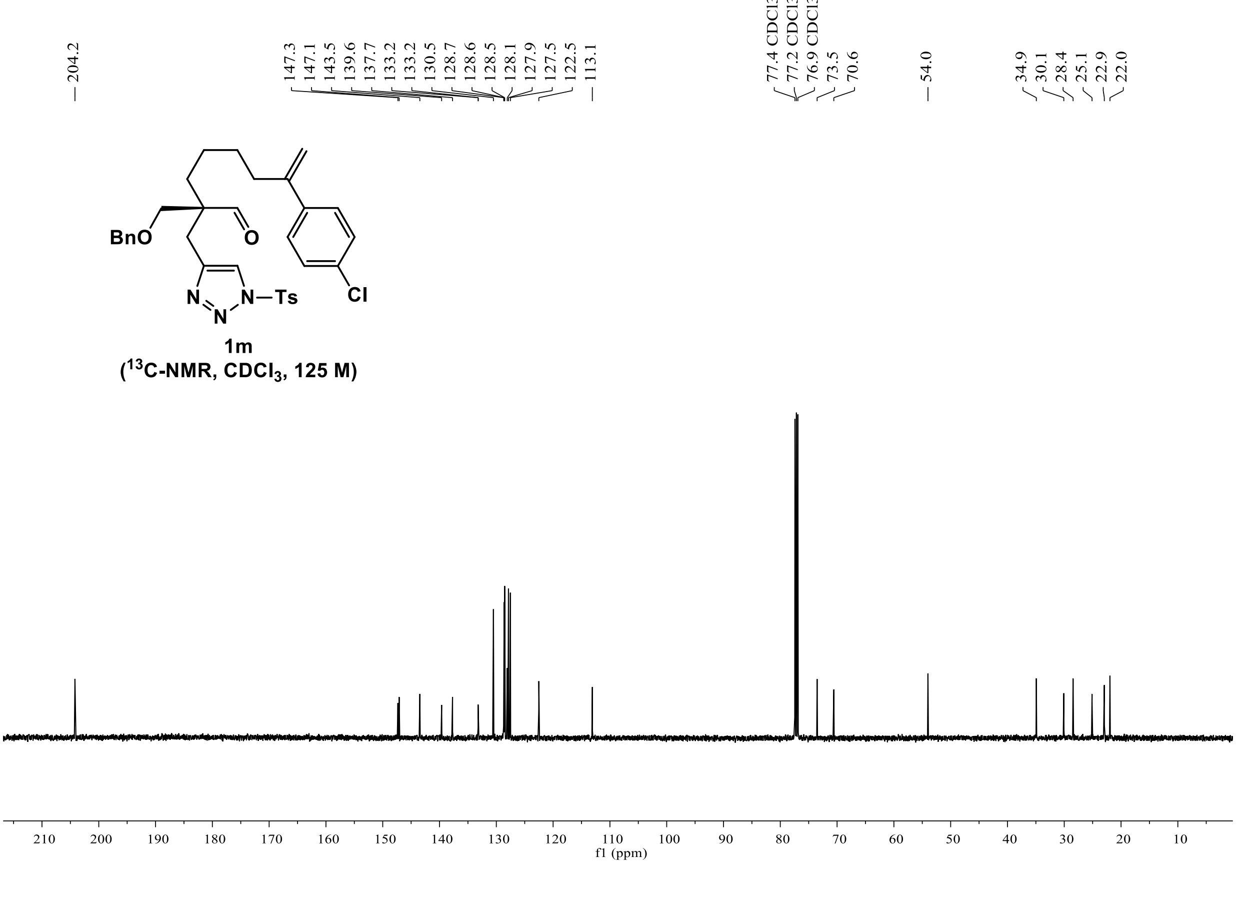

1m
(^{13}C-NMR, $CDCl_3$, 125 M)

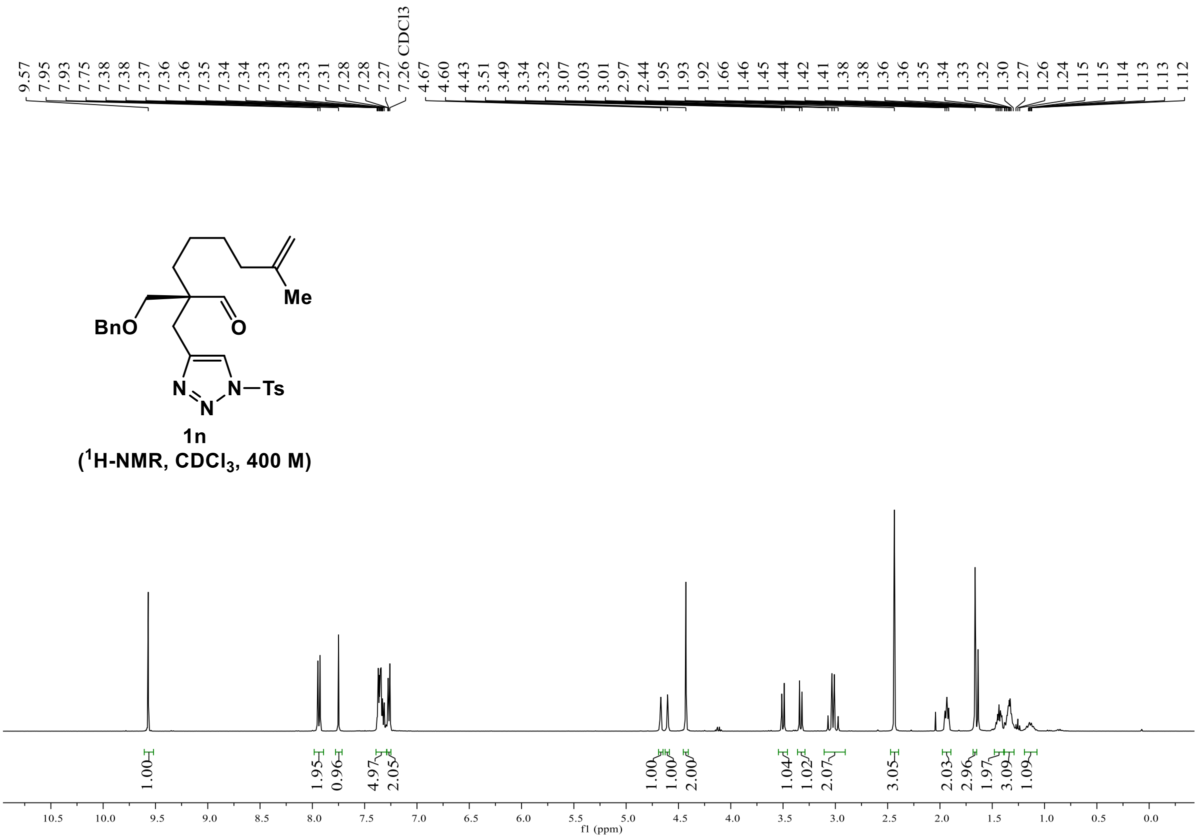
9.57 7.95 7.93 7.75 7.38 7.38 7.37 7.36 7.36 7.35 7.34 7.34 7.33 7.33 7.33 7.31 7.28 7.28 7.27 7.26 CDCl3
4.67 4.60 4.43 3.51 3.49 3.34 3.32 3.07 3.03 3.01 2.97 2.44 1.95 1.93 1.92 1.66 1.46 1.45 1.44 1.42 1.41 1.38 1.38 1.36 1.36 1.35 1.34 1.33 1.32 1.30 1.27 1.26 1.24 1.15 1.15 1.14 1.13 1.13 1.12
BnO
Me
O
N
N
N—Ts
1n
(^{1}H-NMR, $CDCl_3$, 400 M)
1.00 1.95 0.96 4.97 2.05 1.00 1.00 2.00 1.04 1.02 2.07 3.05 2.03 2.96 1.97 3.09 1.09
f1 (ppm)

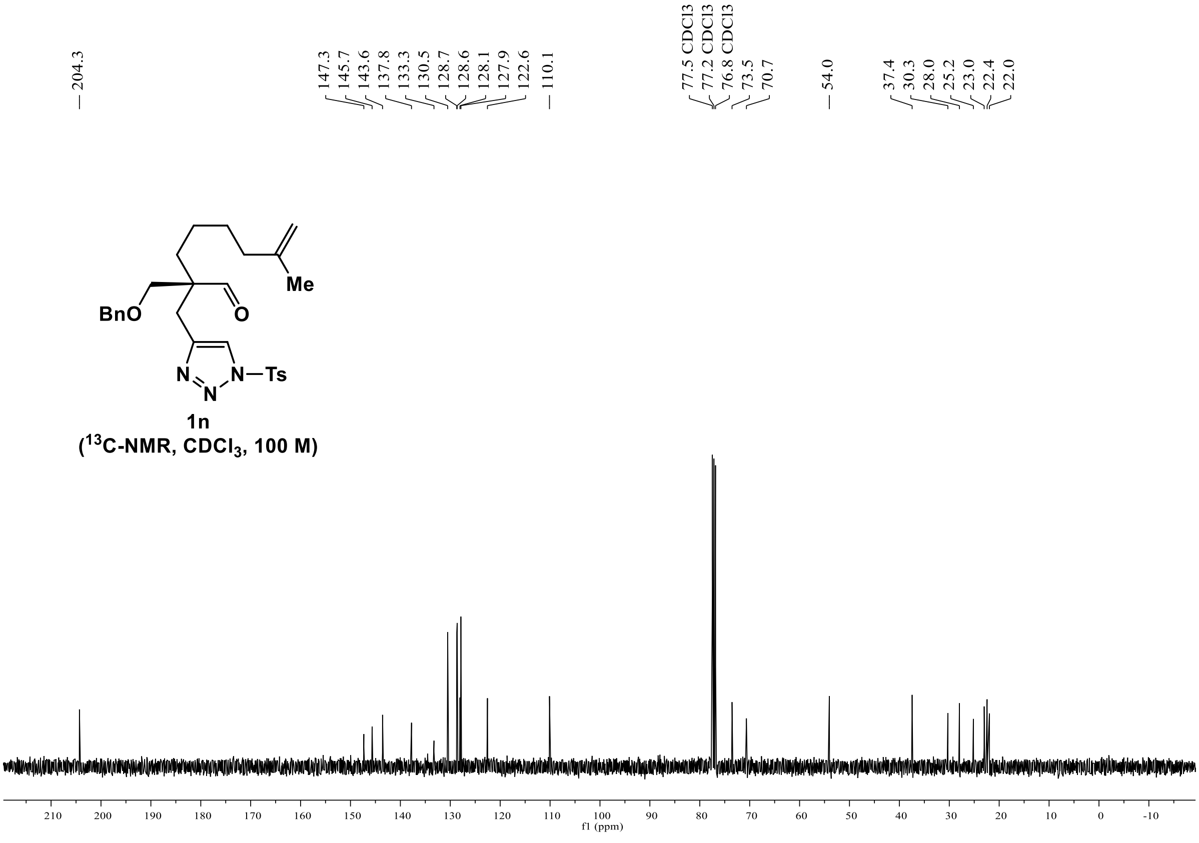
204.3
147.3 145.7 143.6 137.8 133.3 130.5 128.7 128.6 128.1 127.9 122.6 110.1
77.5 CDCl3 77.2 CDCl3 76.8 CDCl3 73.5 70.7
54.0
37.4 30.3 28.0 25.2 23.0 22.4 22.0
BnO
Me
O
N
N
N—Ts
1n
(^{13}C-NMR, $CDCl_3$, 100 M)
f1 (ppm)

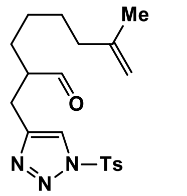

1o
(^{1}H-NMR, $CDCl_3$, 400 M)

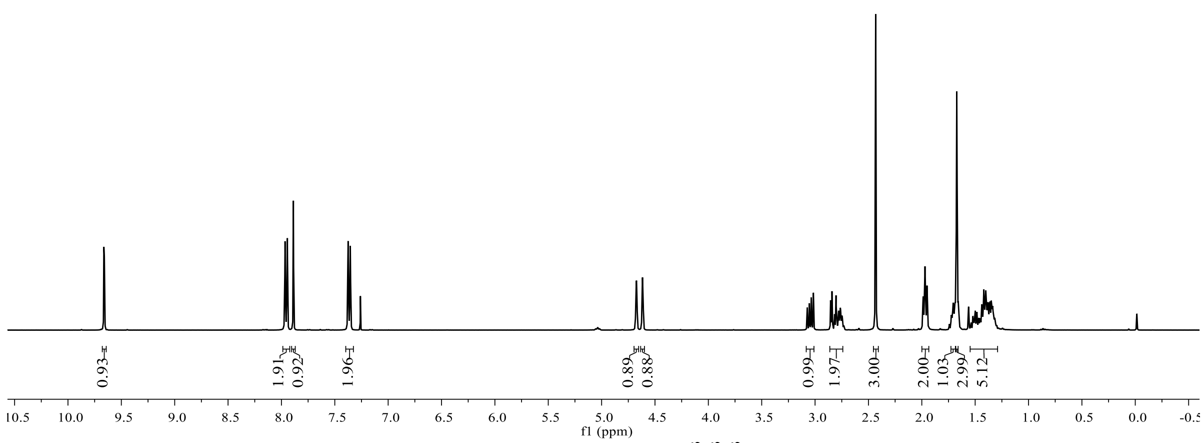

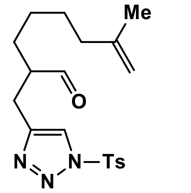

1o
(^{13}C-NMR, $CDCl_3$, 100 M)

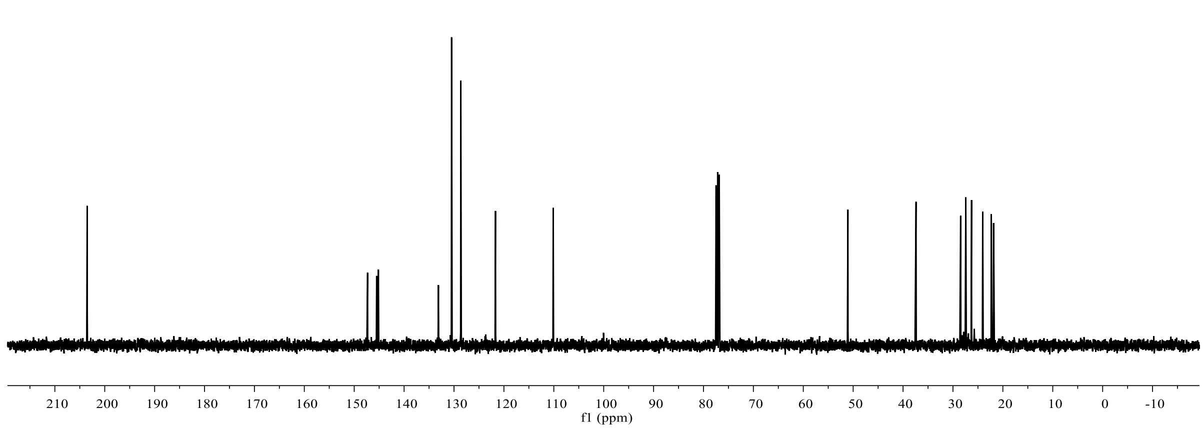

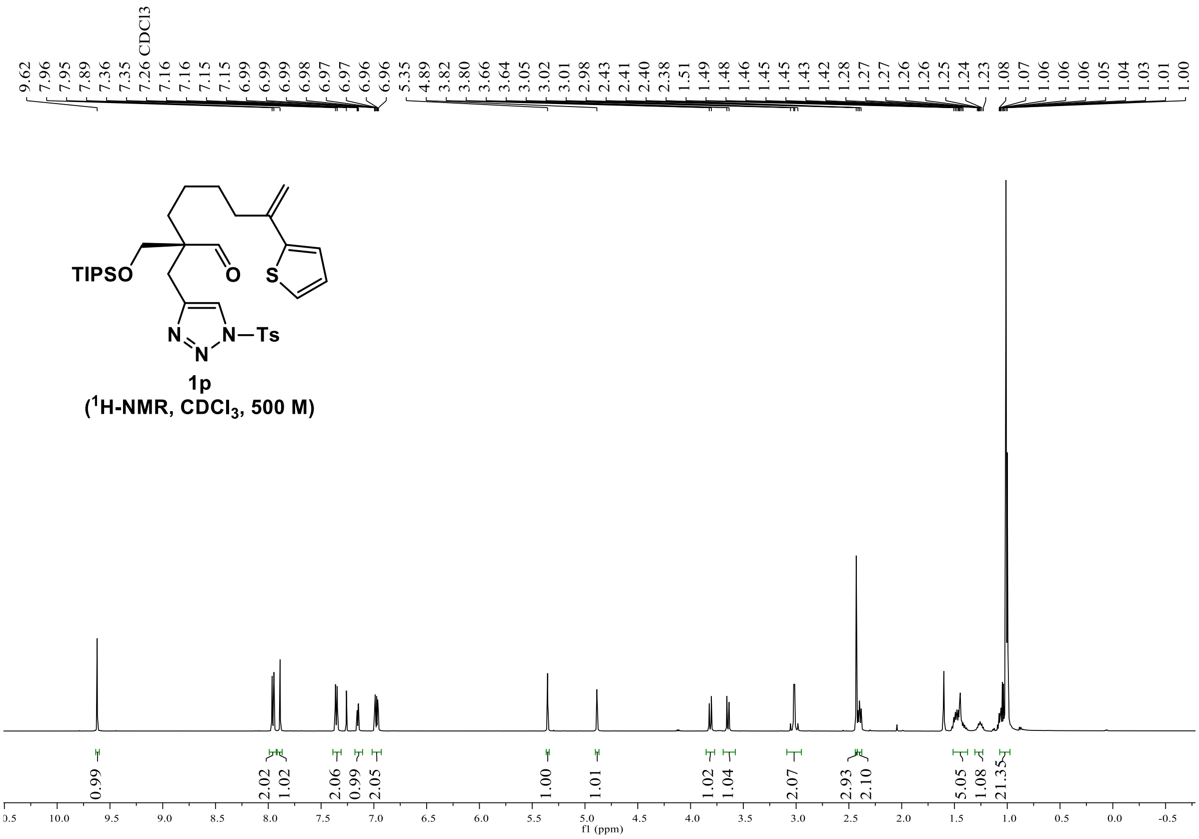

1p
(^{1}H-NMR, CDCl$_3$, 500 M)

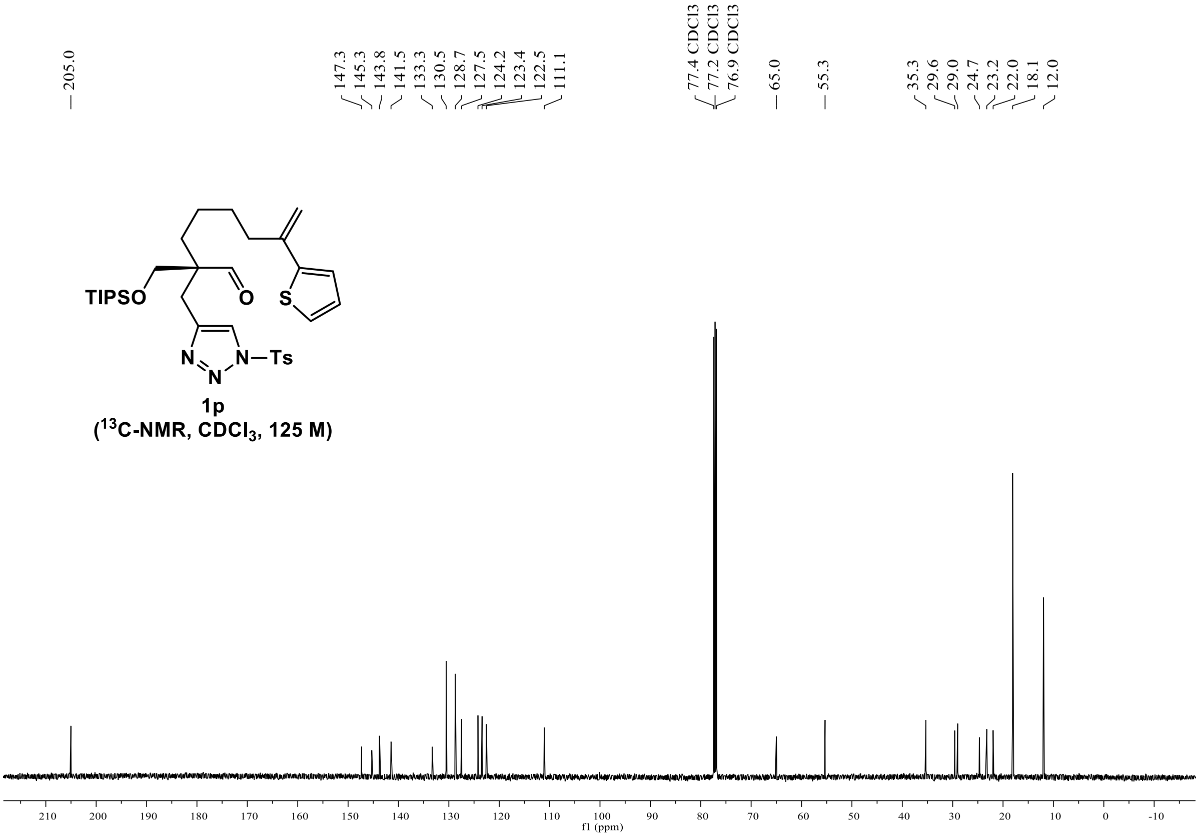

1p
(^{13}C-NMR, CDCl$_3$, 125 M)

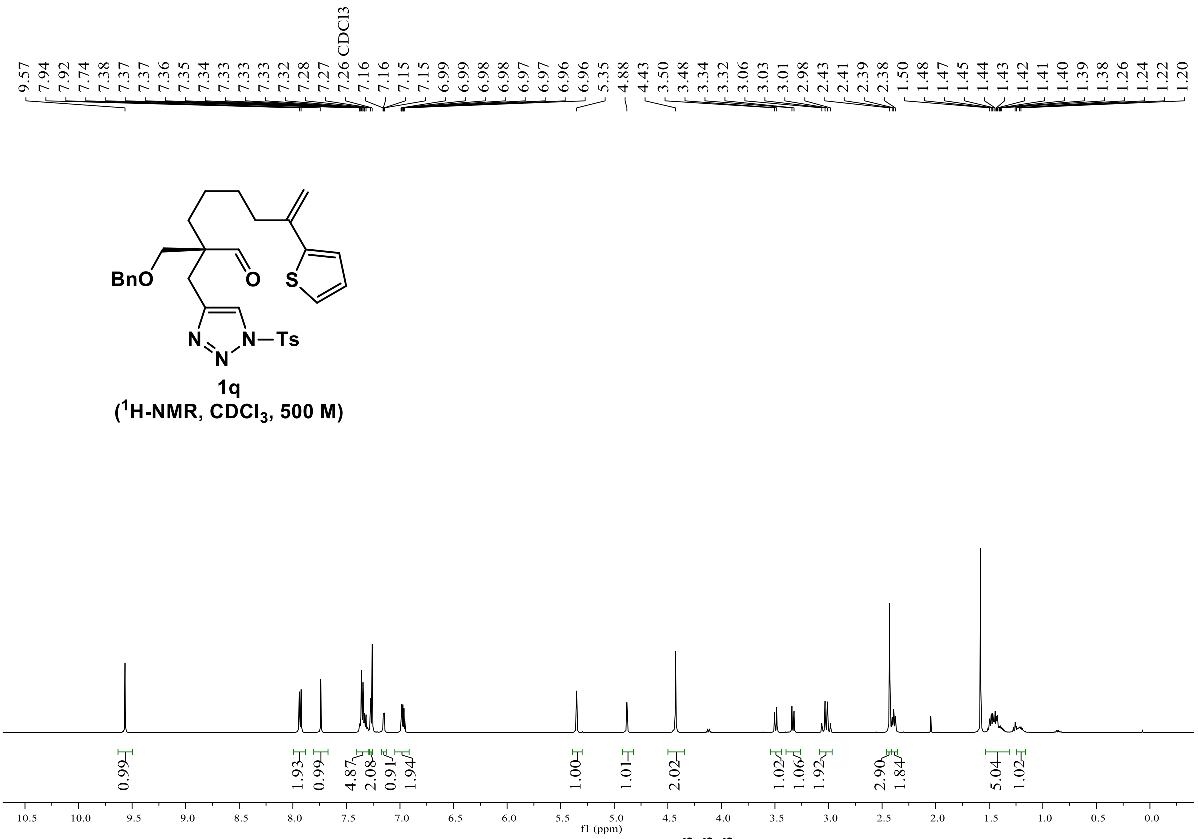
BnO
O
S
N
N
N—Ts
1q
(¹H-NMR, CDCl₃, 500 M)
f1 (ppm)

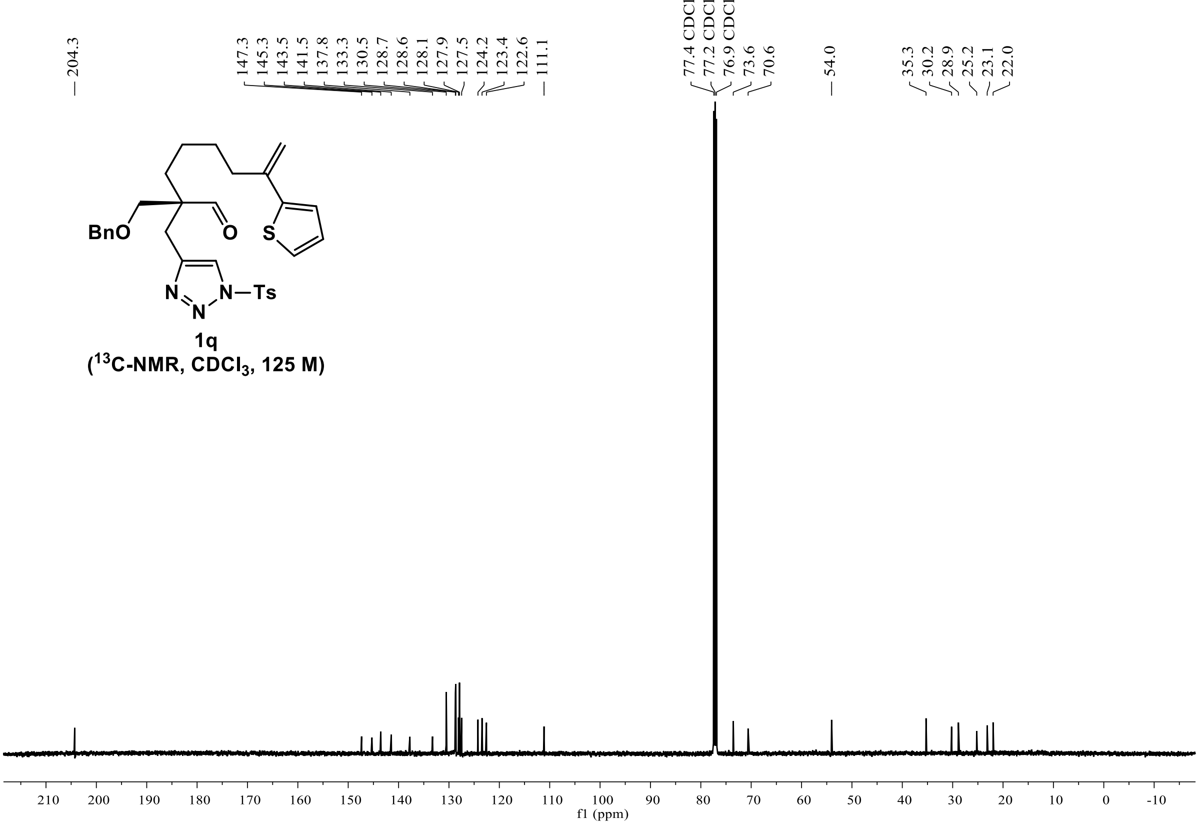
BnO
O
S
N
N
N—Ts
1q
(¹³C-NMR, CDCl₃, 125 M)
f1 (ppm)

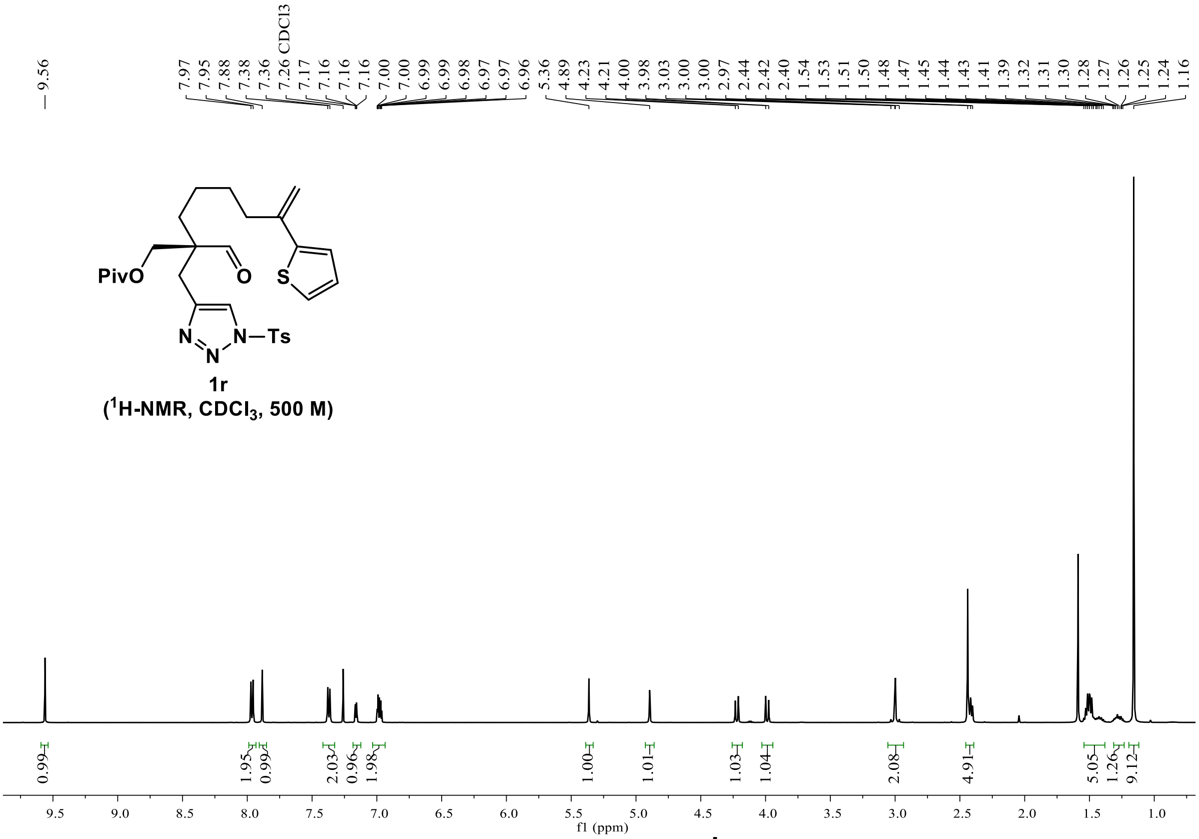
PivO
O
S
N
N
N—Ts
1r
(¹H-NMR, CDCl₃, 500 M)
f1 (ppm)

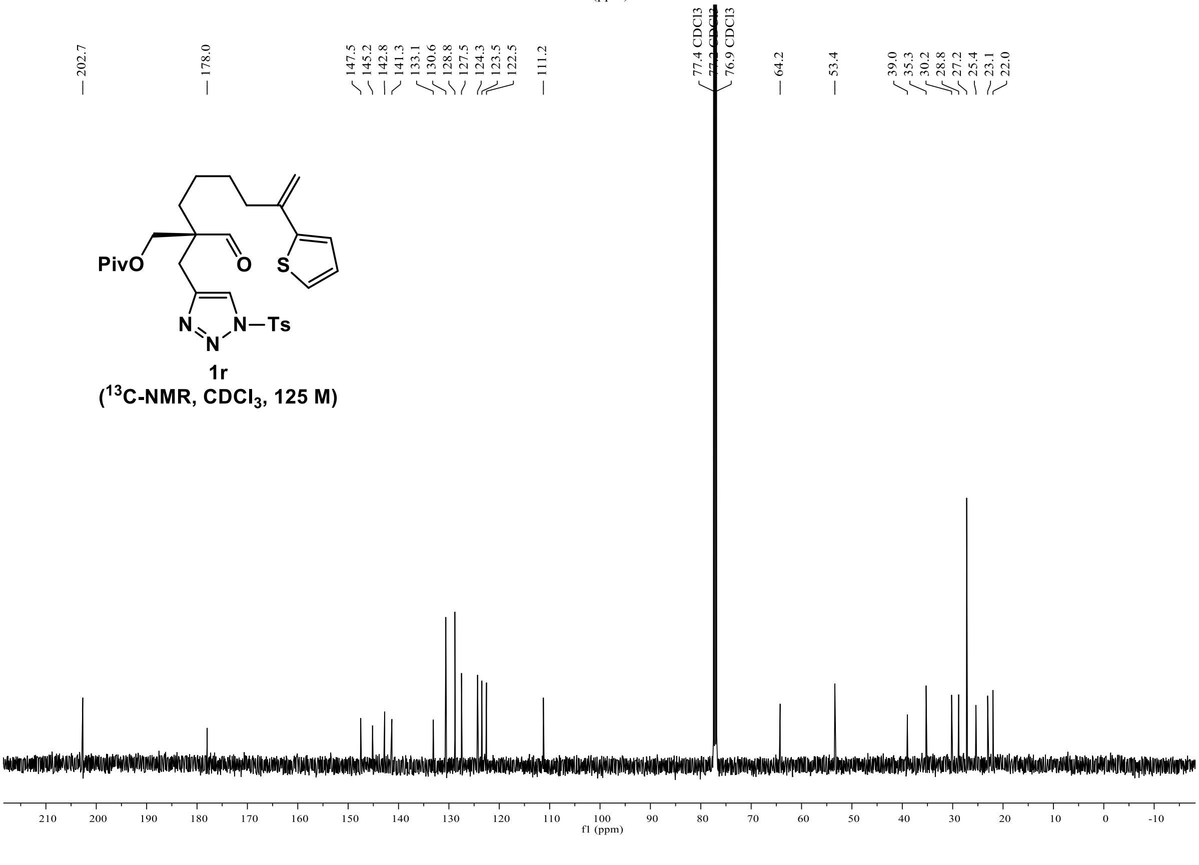
PivO
O
S
N
N
N—Ts
1r
(¹³C-NMR, CDCl₃, 125 M)
f1 (ppm)

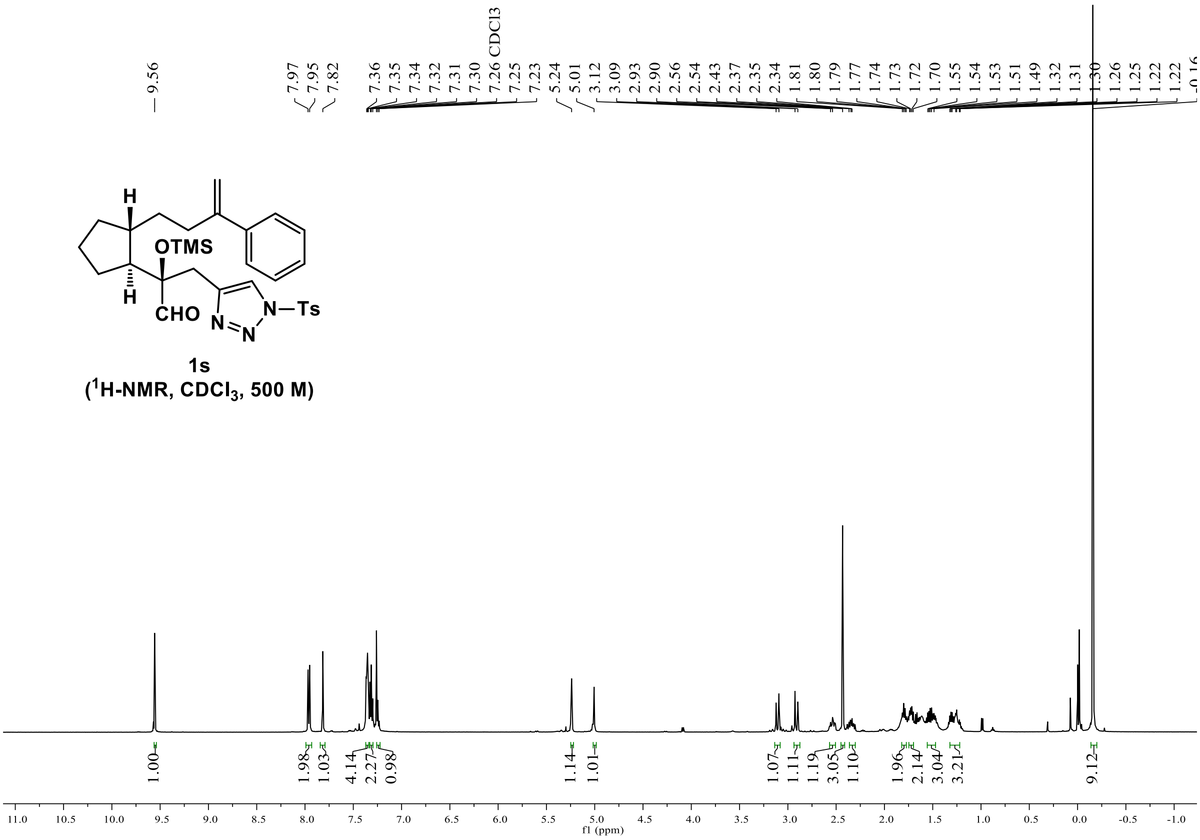

1s
(^{1}H-NMR, $CDCl_3$, 500 M)

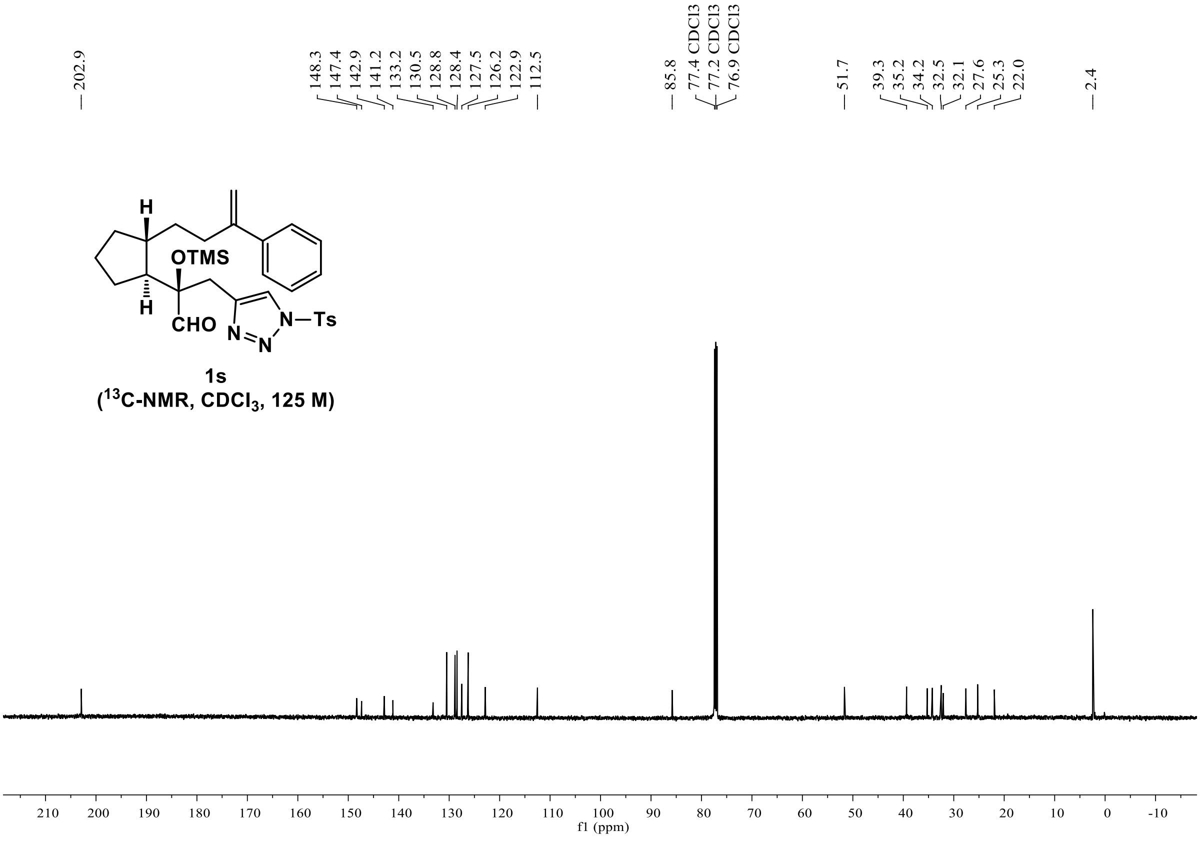

1s
(^{13}C-NMR, $CDCl_3$, 125 M)

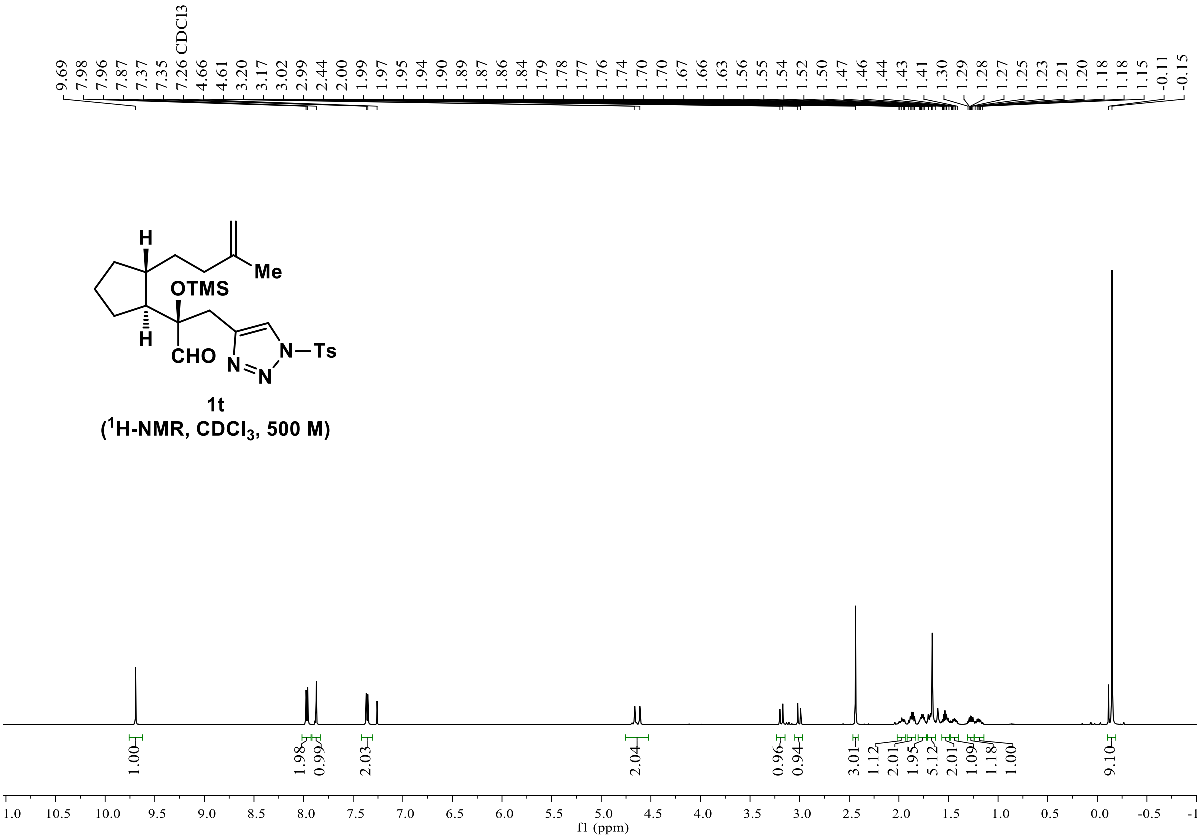

1t
(^{1}H-NMR, $CDCl_3$, 500 M)

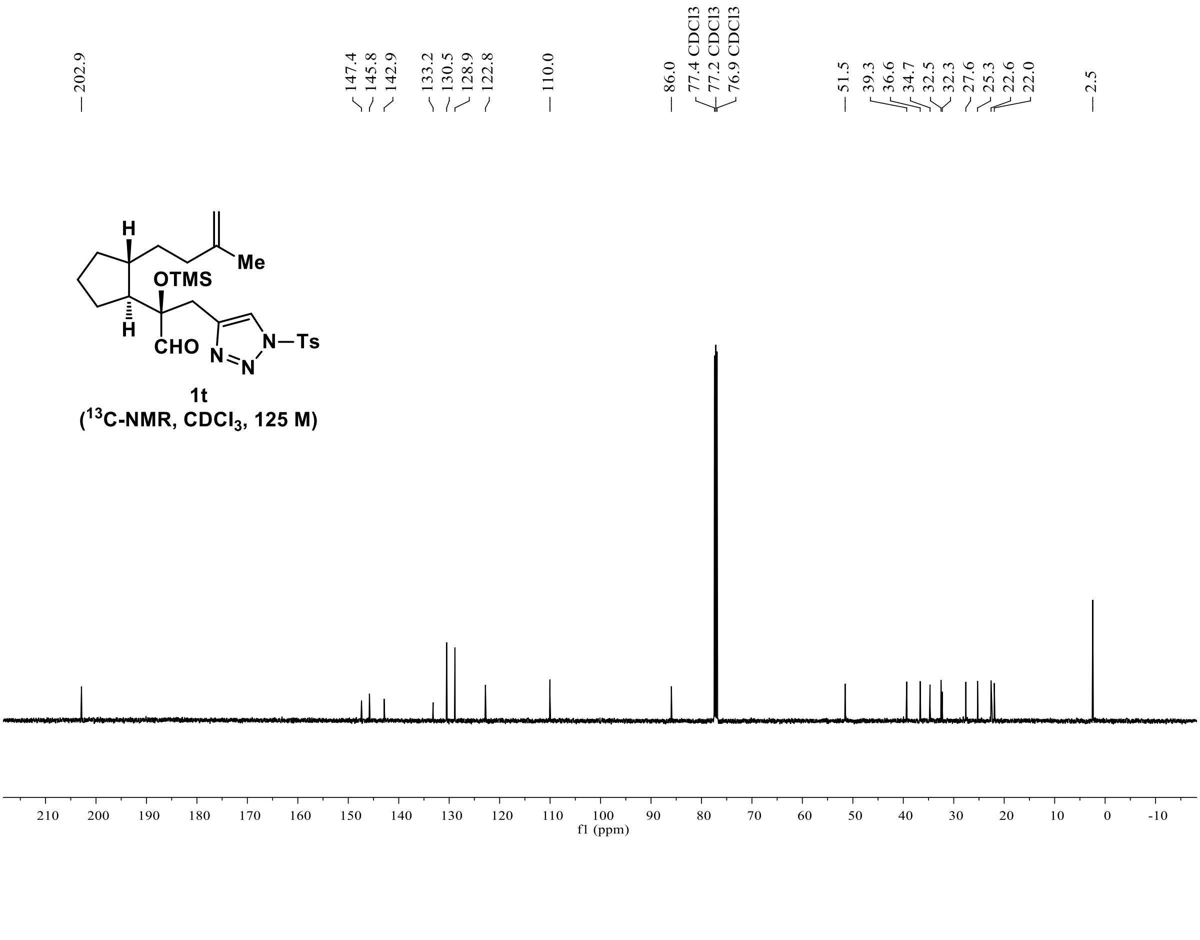

1t
(^{13}C-NMR, $CDCl_3$, 125 M)

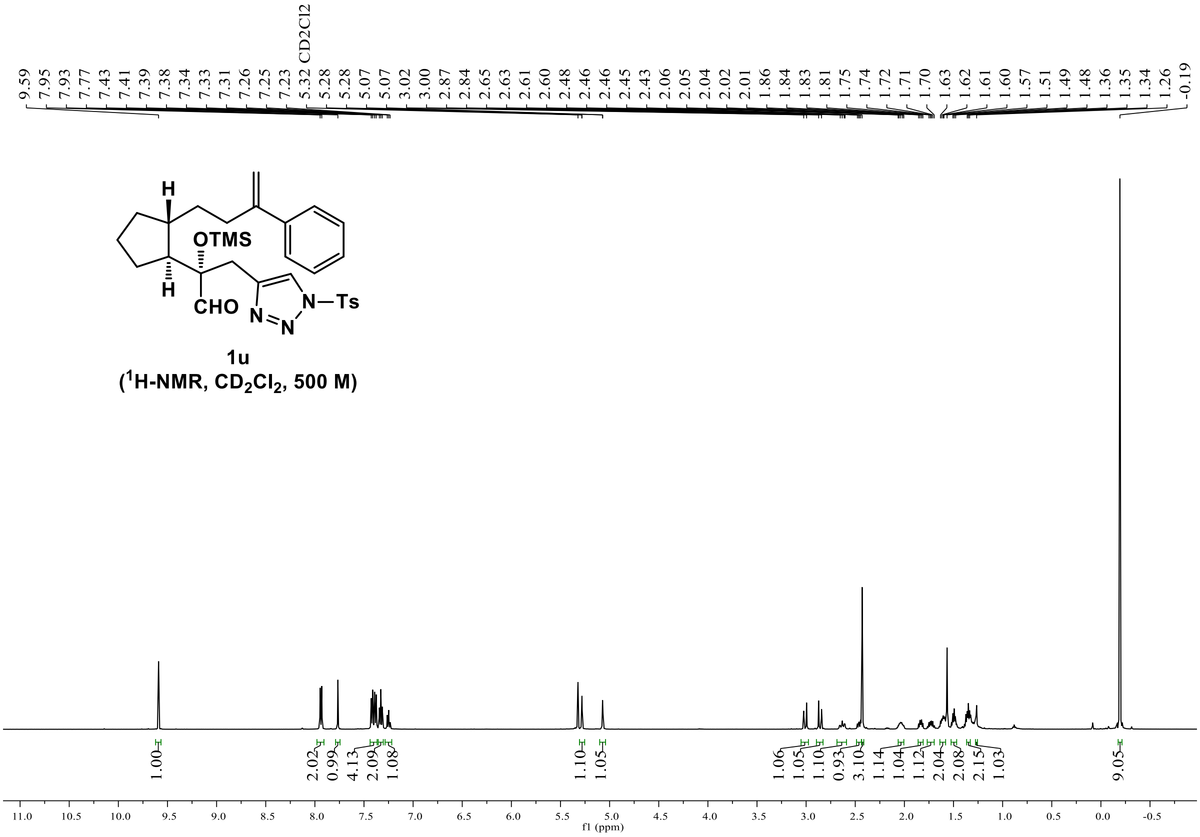

1u

(^{1}H-NMR, CD_2Cl_2, 500 M)

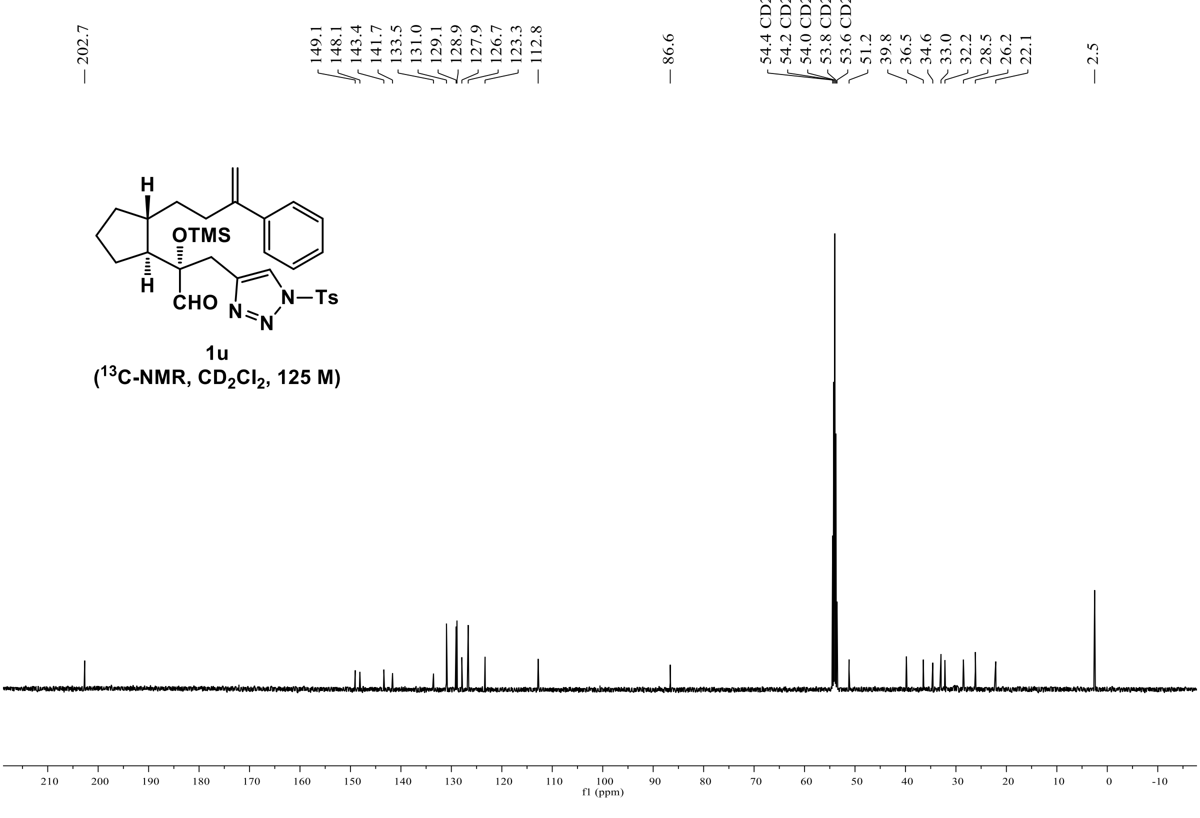

1u

(^{13}C-NMR, CD_2Cl_2, 125 M)

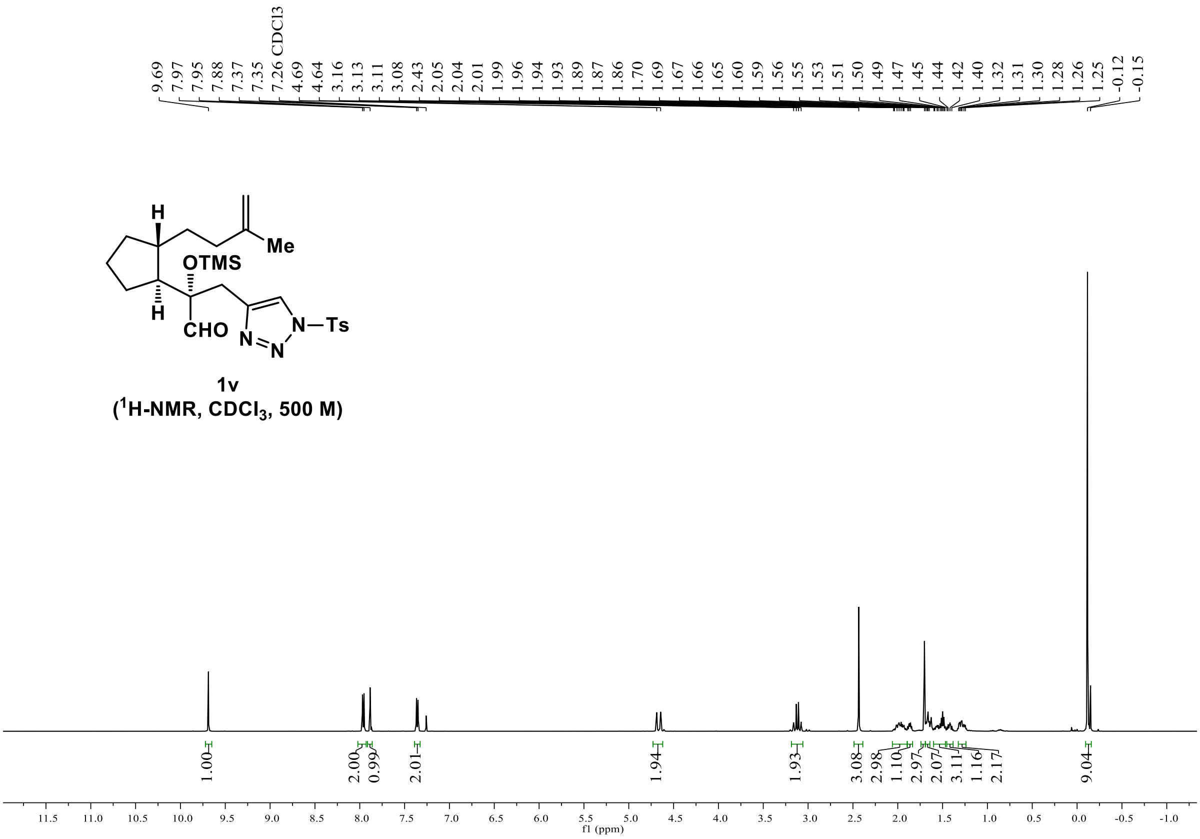

1v
(^{1}H-NMR, CDCl$_3$, 500 M)

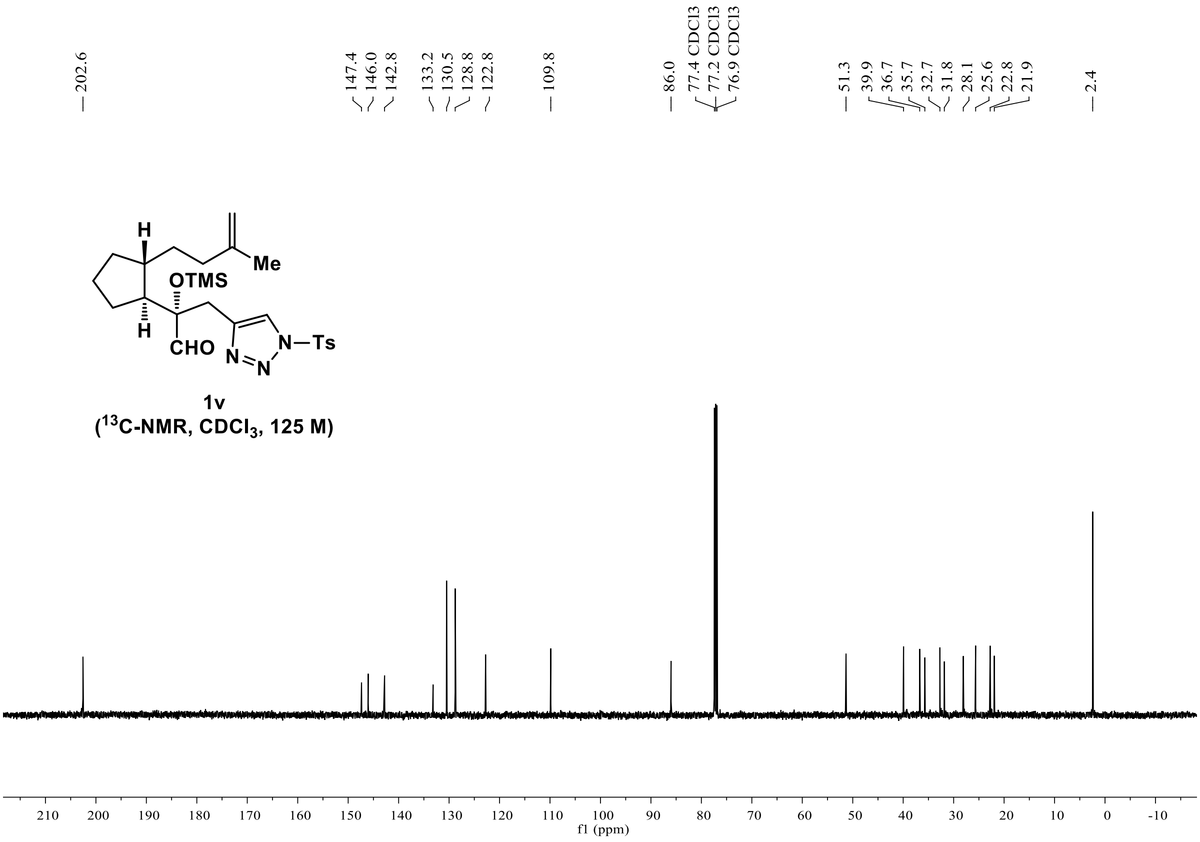

1v
(^{13}C-NMR, CDCl$_3$, 125 M)

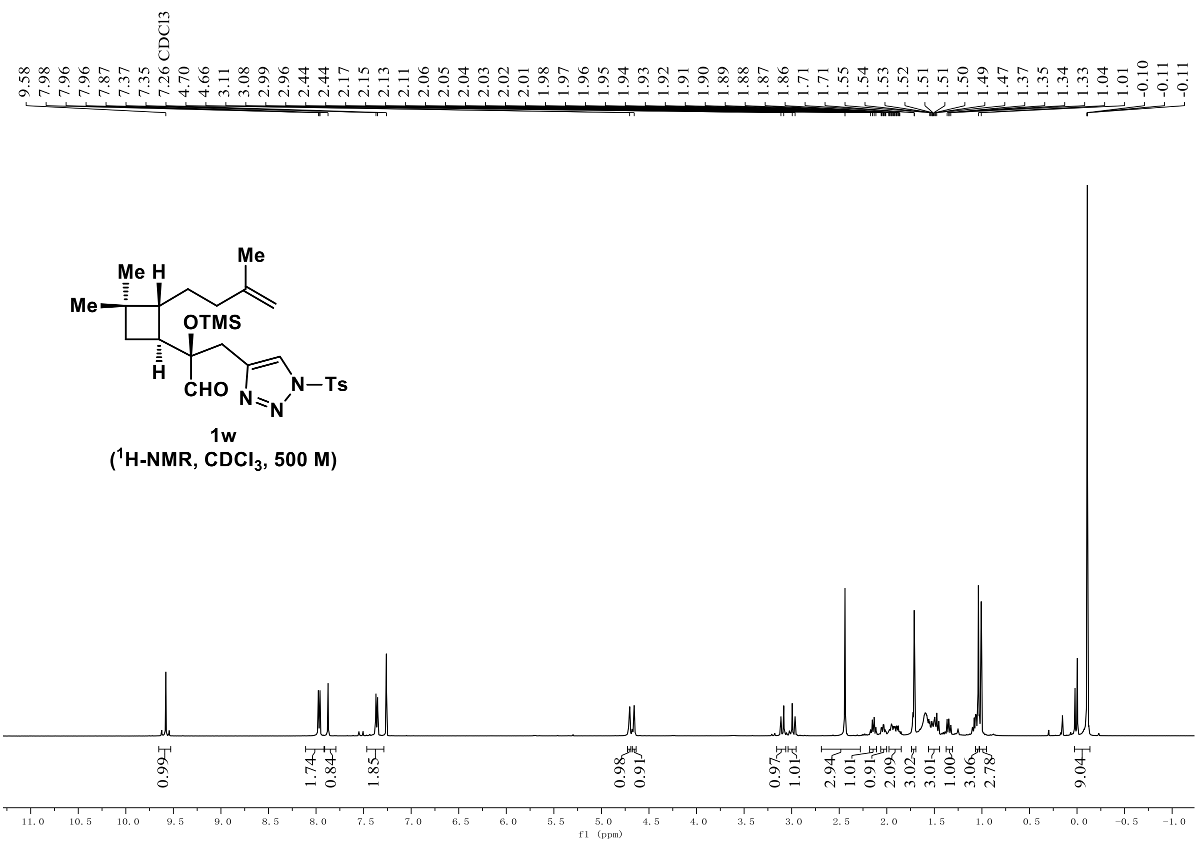
9.58 7.98 7.96 7.96 7.87 7.37 7.35 7.26 CDCl3 4.70 4.66 3.11 3.08 2.99 2.96 2.44 2.44 2.17 2.15 2.13 2.11 2.06 2.05 2.04 2.03 2.02 2.01 1.98 1.97 1.96 1.95 1.94 1.93 1.92 1.91 1.90 1.89 1.88 1.87 1.86 1.71 1.71 1.55 1.54 1.53 1.52 1.51 1.51 1.50 1.49 1.47 1.37 1.35 1.34 1.33 1.04 1.01 -0.10 -0.11 -0.11
Me H
Me
OTMS
H
CHO
N—Ts
N
N
1w
(¹H-NMR, CDCl₃, 500 M)
0.99 1.74 0.84 1.85 0.98 0.91 0.97 1.01 2.94 1.01 0.91 0.91 2.09 3.02 3.01 1.00 3.06 2.78 9.04
11.0 10.5 10.0 9.5 9.0 8.5 8.0 7.5 7.0 6.5 6.0 5.5 5.0 4.5 4.0 3.5 3.0 2.5 2.0 1.5 1.0 0.5 0.0 -0.5 -1.0
f1 (ppm)

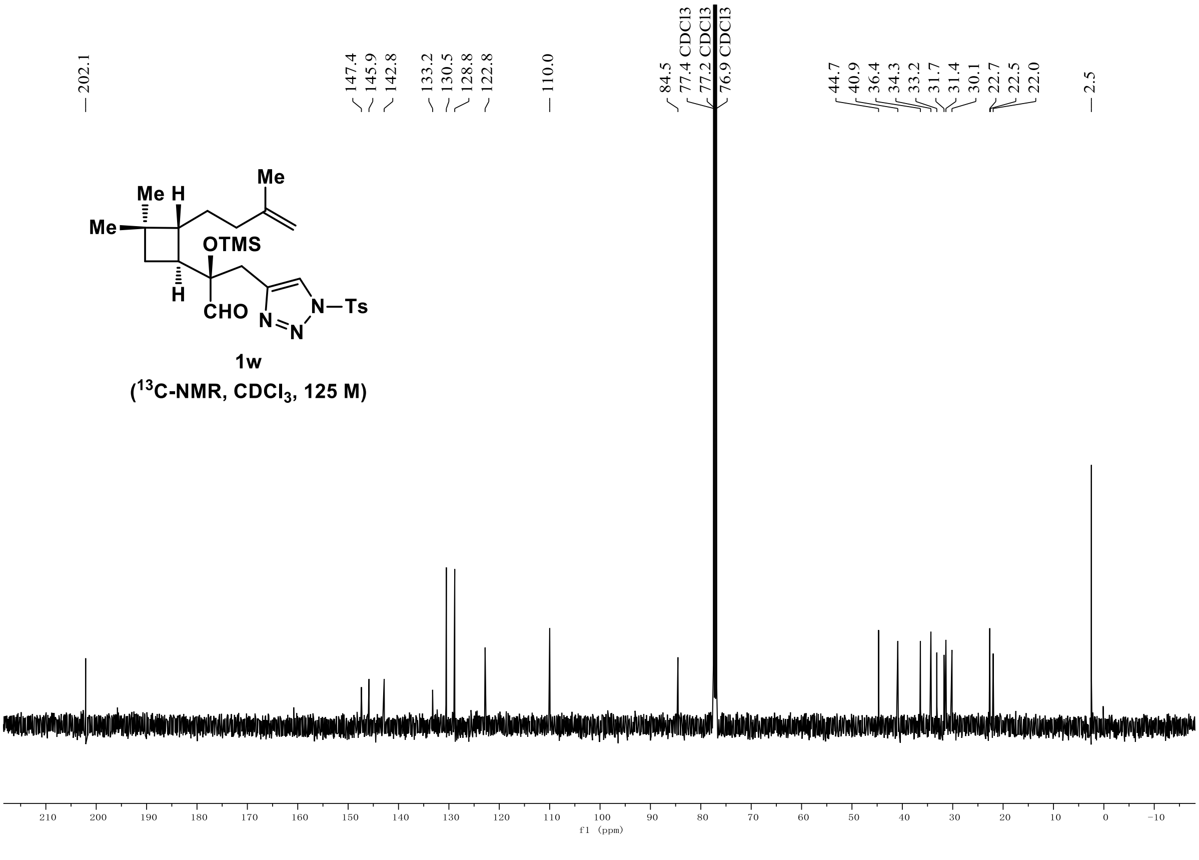
202.1
147.4 145.9 142.8 133.2 130.5 128.8 122.8 110.0
84.5 77.4 CDCl3 77.2 CDCl3 76.9 CDCl3
44.7 40.9 36.4 34.3 33.2 31.7 31.4 30.1 22.7 22.5 22.0 2.5
Me H
Me
OTMS
H
CHO
N—Ts
N
N
1w
(¹³C-NMR, CDCl₃, 125 M)
210 200 190 180 170 160 150 140 130 120 110 100 90 80 70 60 50 40 30 20 10 0 -10
f1 (ppm)

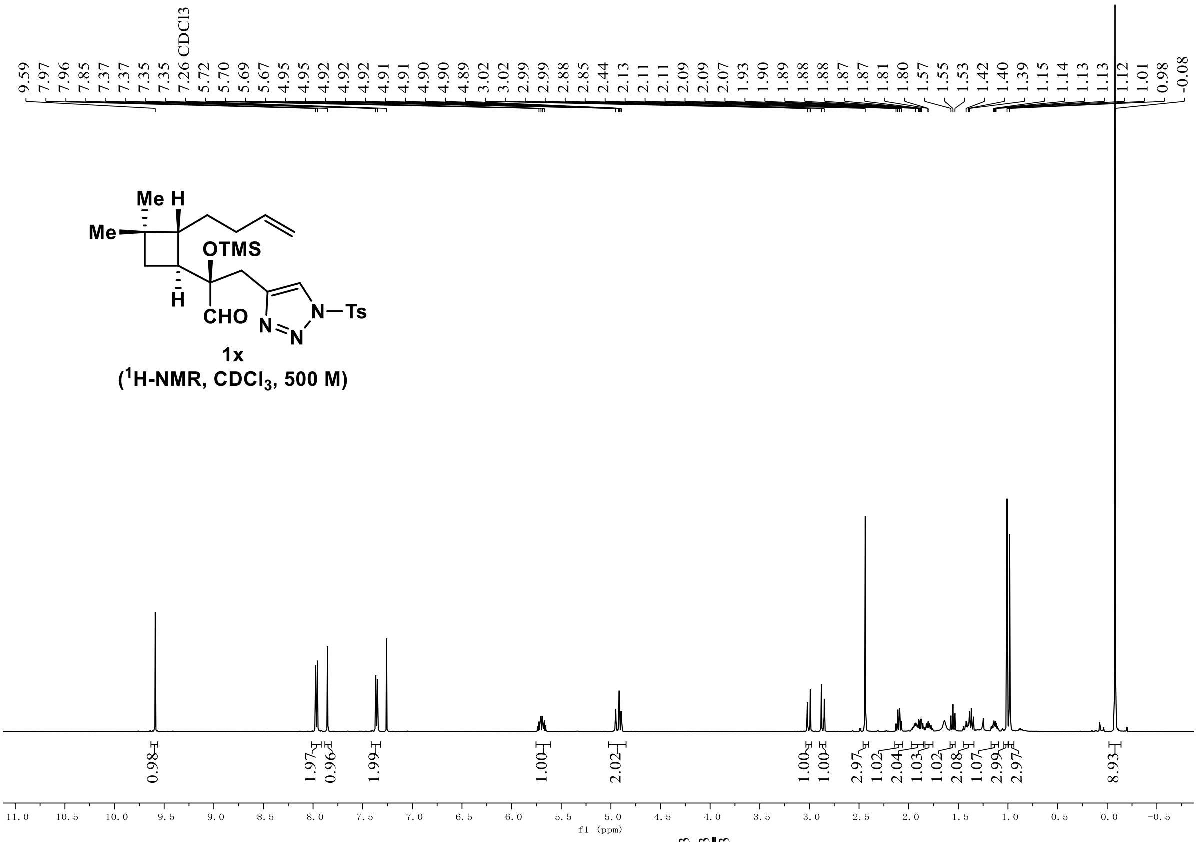

1x
(^{1}H-NMR, $CDCl_3$, 500 M)

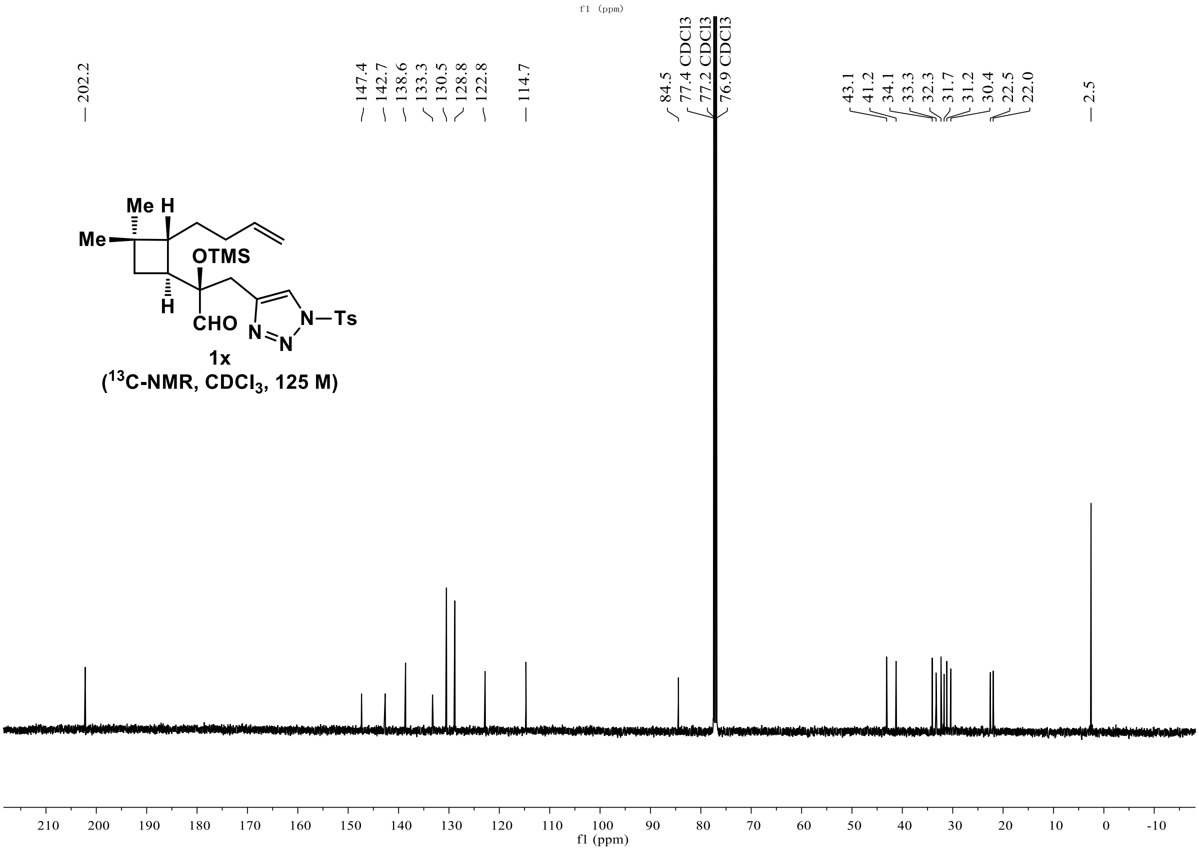

1x
(^{13}C-NMR, $CDCl_3$, 125 M)

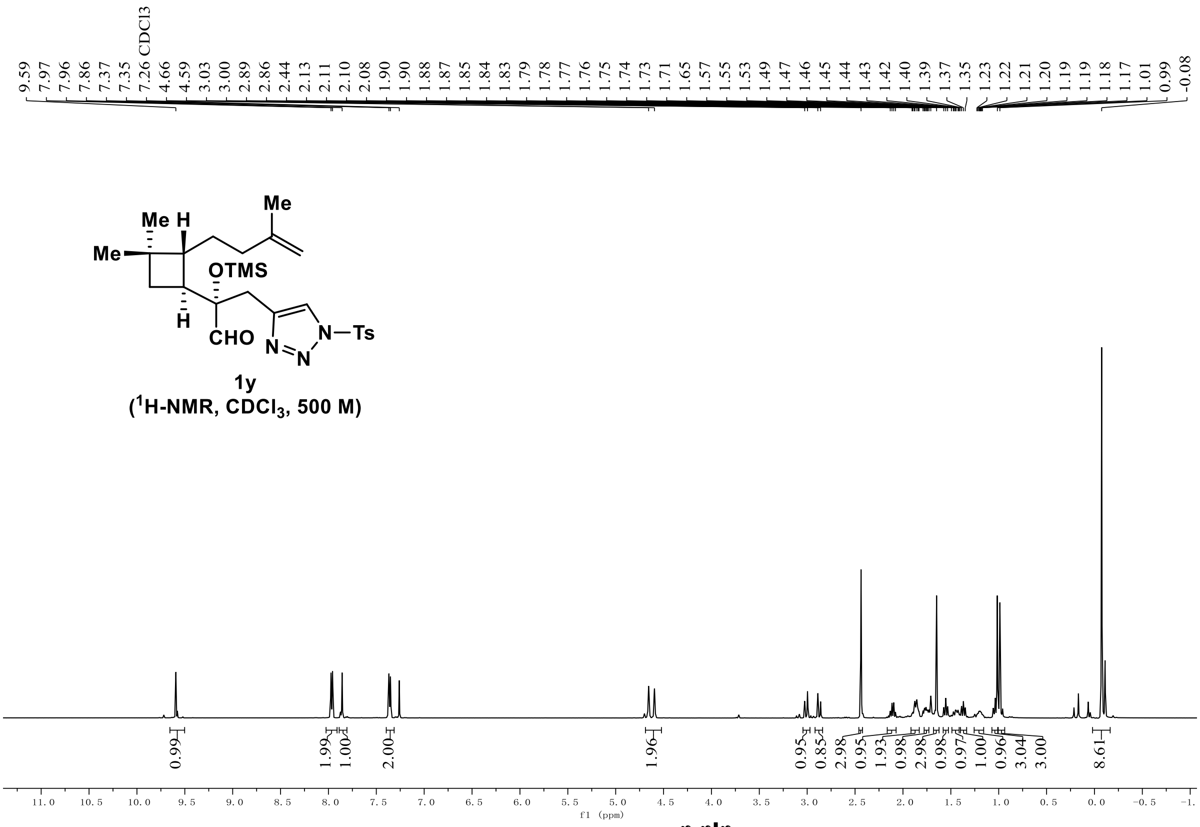

1y
(^{1}H-NMR, $CDCl_3$, 500 M)

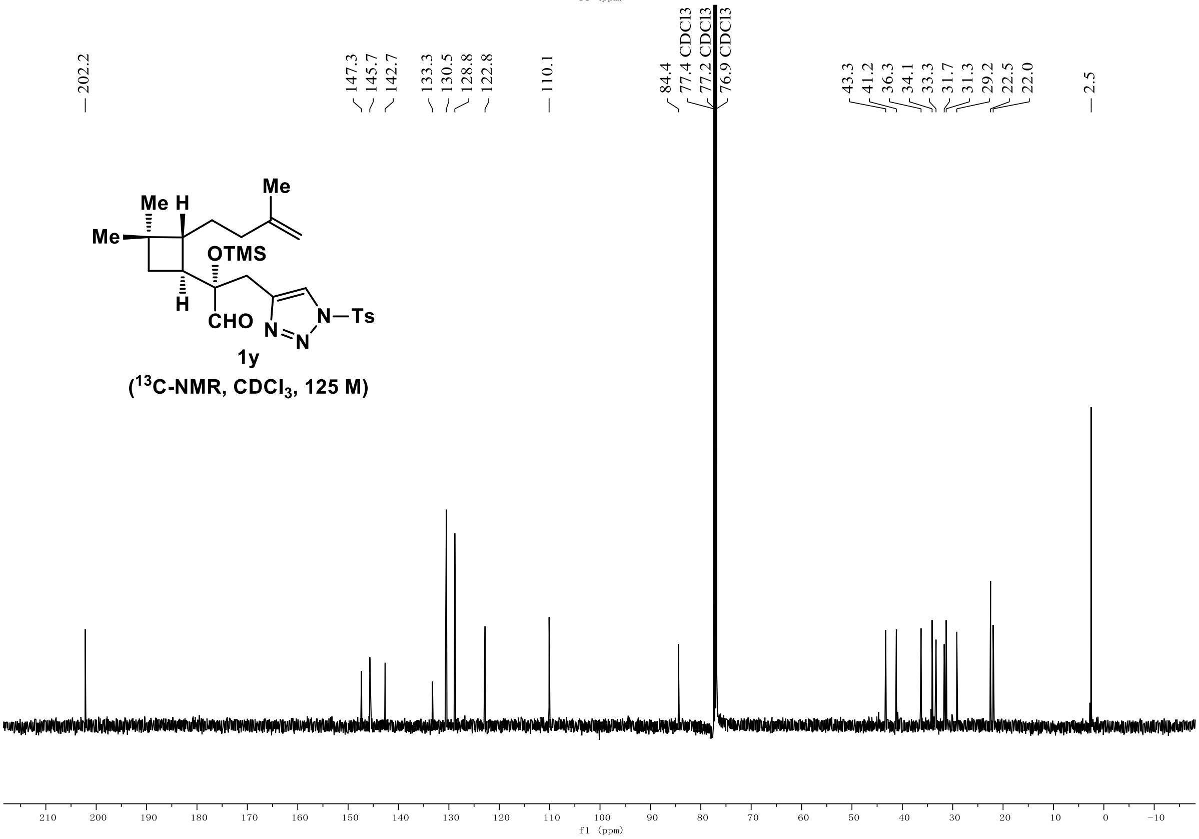

1y
(^{13}C-NMR, $CDCl_3$, 125 M)

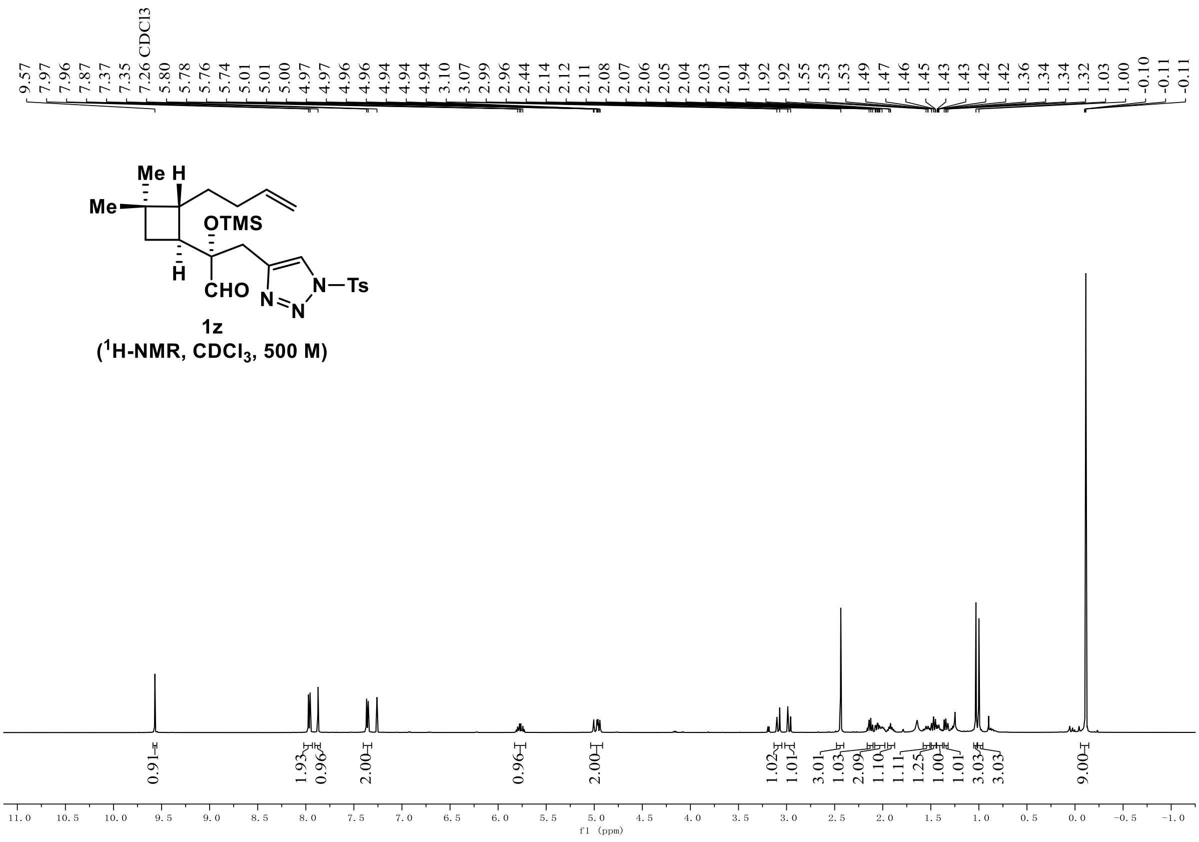

1z
(^{1}H-NMR, $CDCl_3$, 500 M)

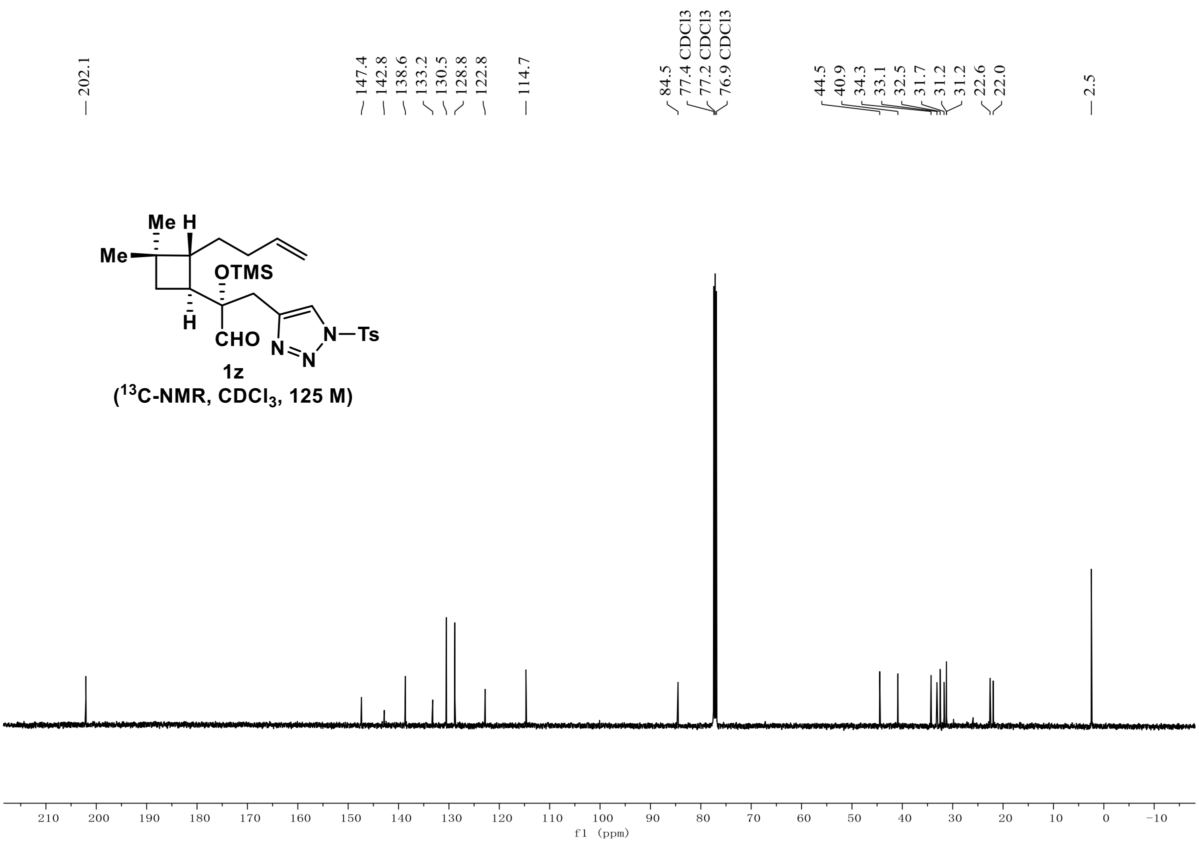

1z
(^{13}C-NMR, $CDCl_3$, 125 M)

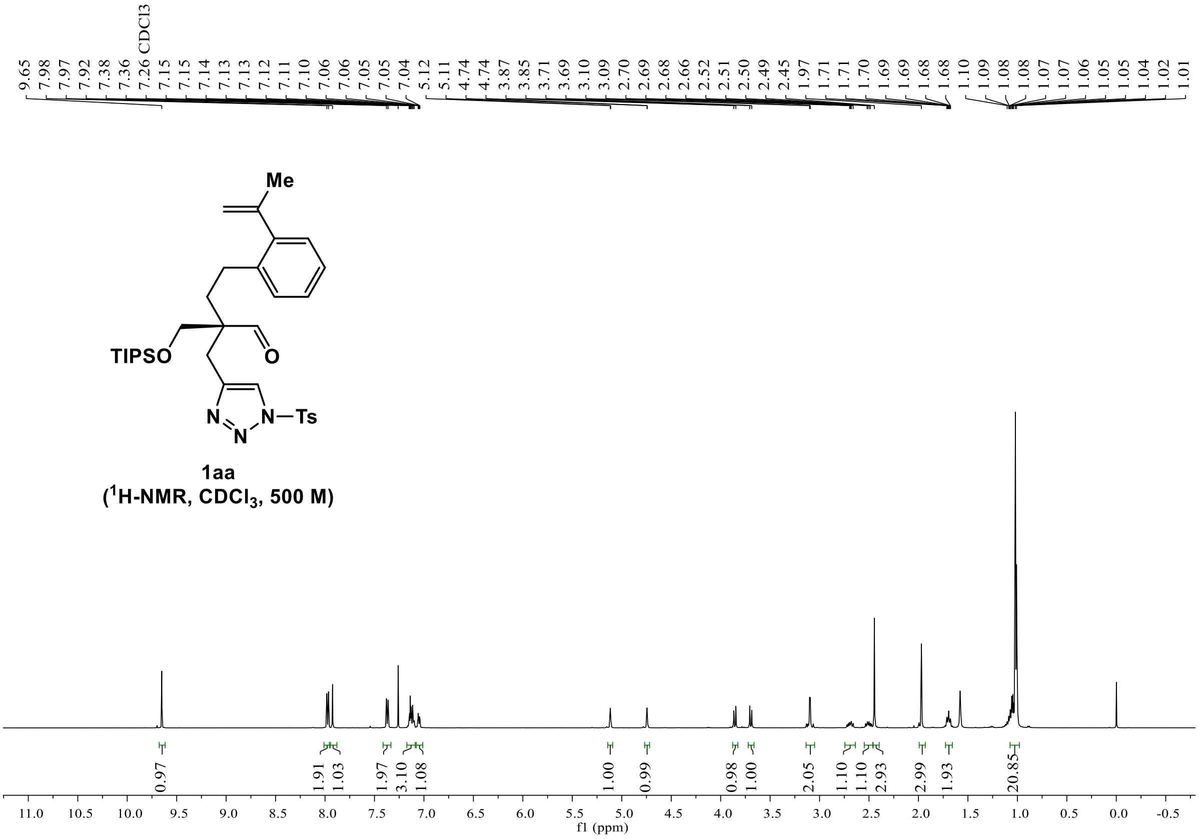

1aa
(^{1}H-NMR, $CDCl_3$, 500 M)

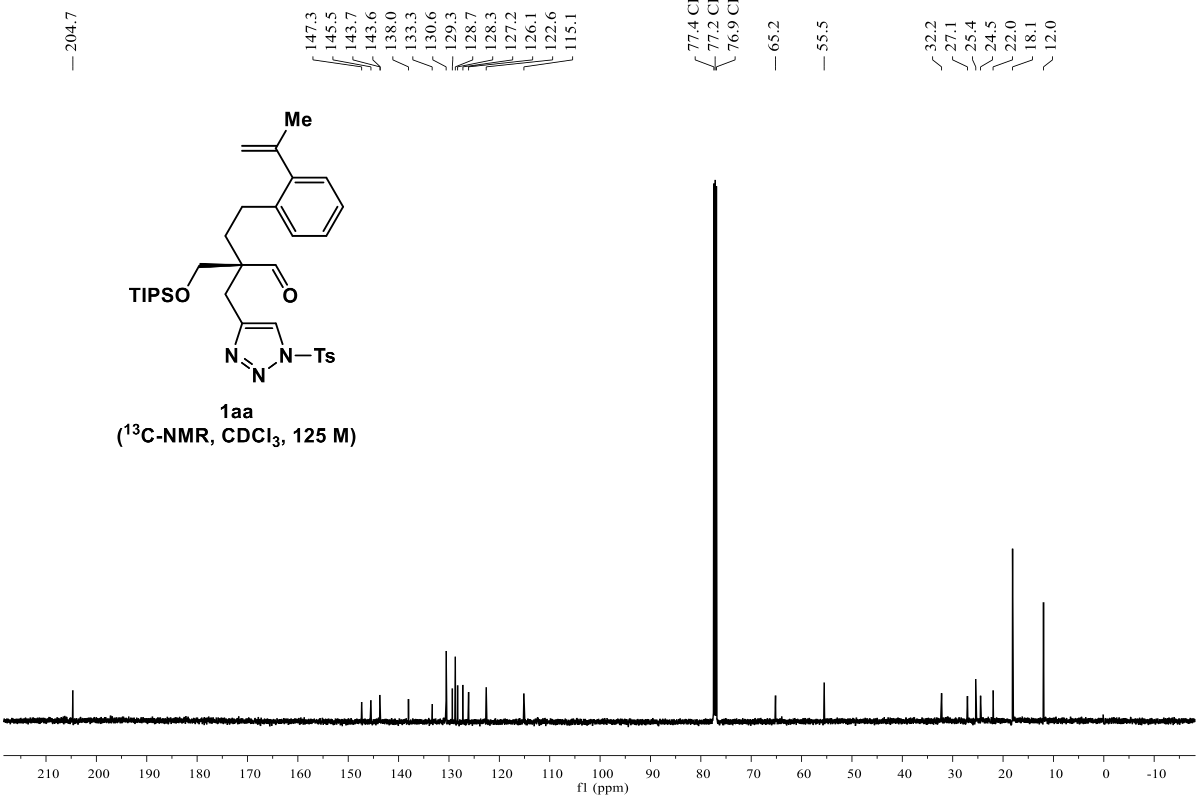

1aa
(^{13}C-NMR, $CDCl_3$, 125 M)

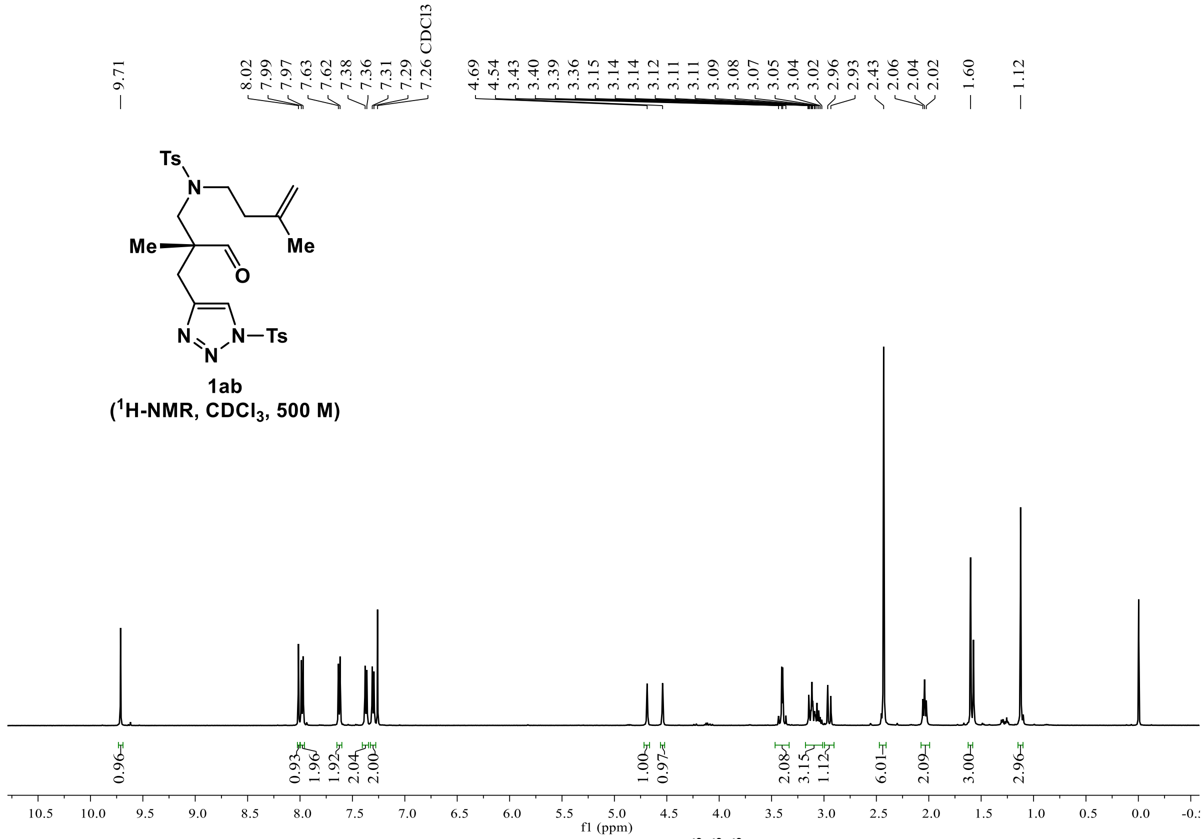
9.71
8.02
7.99
7.97
7.63
7.62
7.38
7.36
7.31
7.29
7.26 CDCl3
4.69
4.54
3.43
3.40
3.39
3.36
3.15
3.14
3.14
3.12
3.11
3.11
3.09
3.08
3.07
3.05
3.04
3.02
2.96
2.93
2.43
2.06
2.04
2.02
1.60
1.12
Ts
N
Me
Me
O
N
N
N
Ts
1ab
(1H-NMR, CDCl3, 500 M)
0.96
0.93
1.96
1.92
2.04
2.00
1.00
0.97
2.08
3.15
1.12
6.01
2.09
3.00
2.96
f1 (ppm)

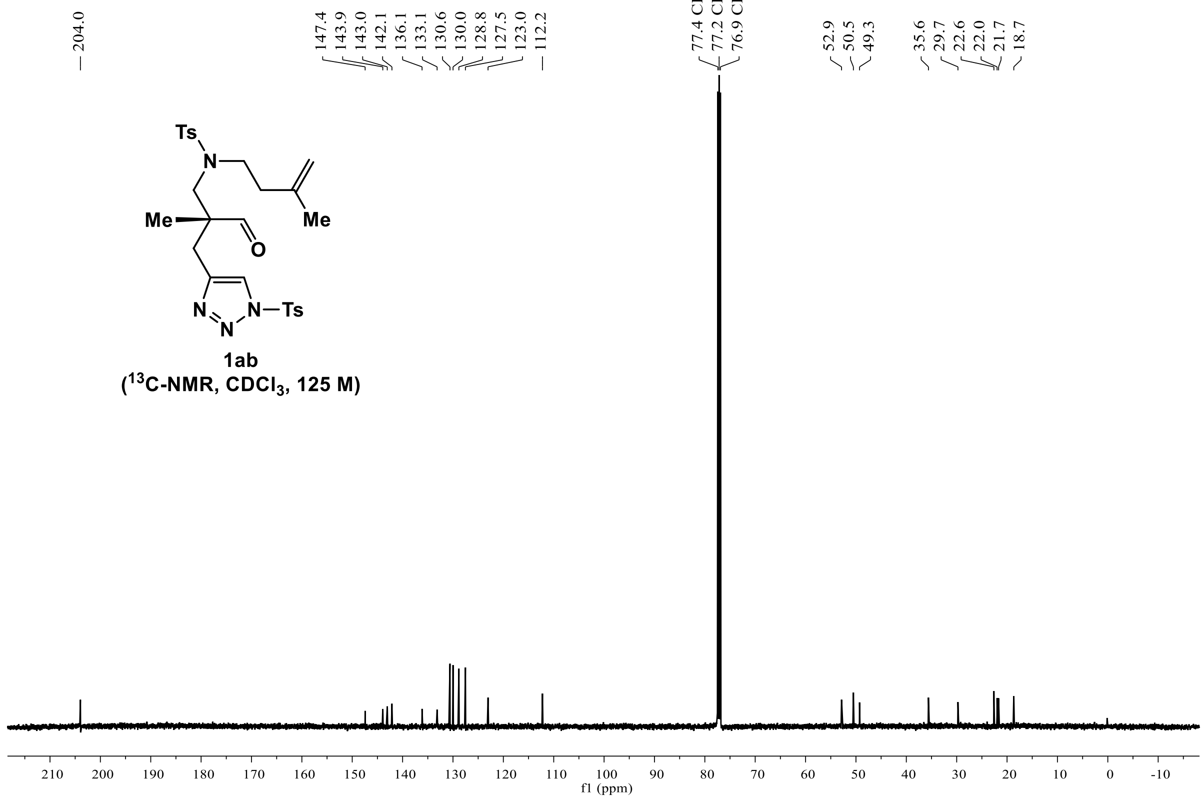
204.0
147.4
143.9
143.0
142.1
136.1
133.1
130.6
130.0
128.8
127.5
123.0
112.2
77.4 CDCl3
77.2 CDCl3
76.9 CDCl3
52.9
50.5
49.3
35.6
29.7
22.6
22.0
21.7
18.7
Ts
N
Me
Me
O
N
N
N
Ts
1ab
(13C-NMR, CDCl3, 125 M)
f1 (ppm)

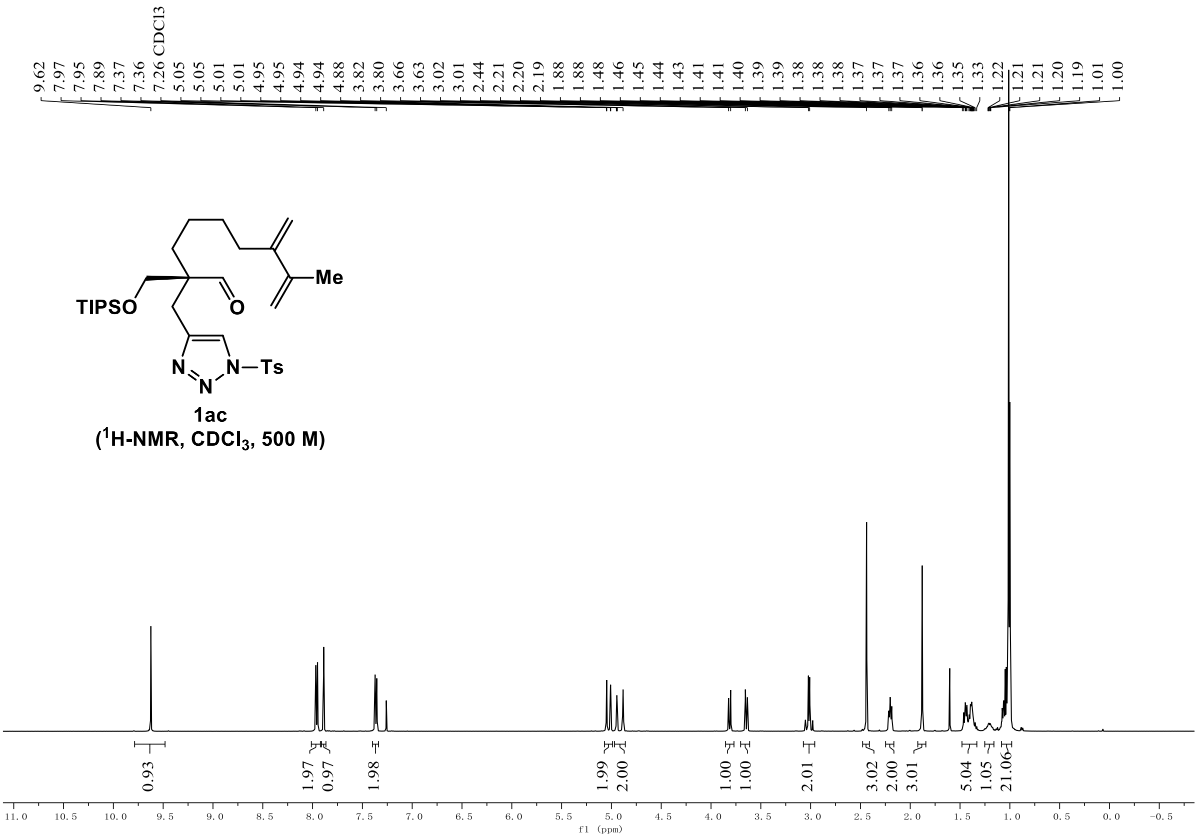
9.62 7.97 7.95 7.89 7.37 7.36 7.26 CDCl3 5.05 5.05 5.01 5.01 4.95 4.95 4.94 4.94 4.88 3.82 3.80 3.66 3.63 3.02 3.01 2.44 2.21 2.20 2.19 1.88 1.88 1.48 1.46 1.45 1.44 1.43 1.41 1.41 1.40 1.39 1.39 1.38 1.38 1.38 1.37 1.37 1.37 1.36 1.36 1.35 1.33 1.22 1.21 1.21 1.20 1.19 1.01 1.00
TIPSO
O
Me
N
N
N—Ts
1ac
(¹H-NMR, CDCl3, 500 M)
f1 (ppm)

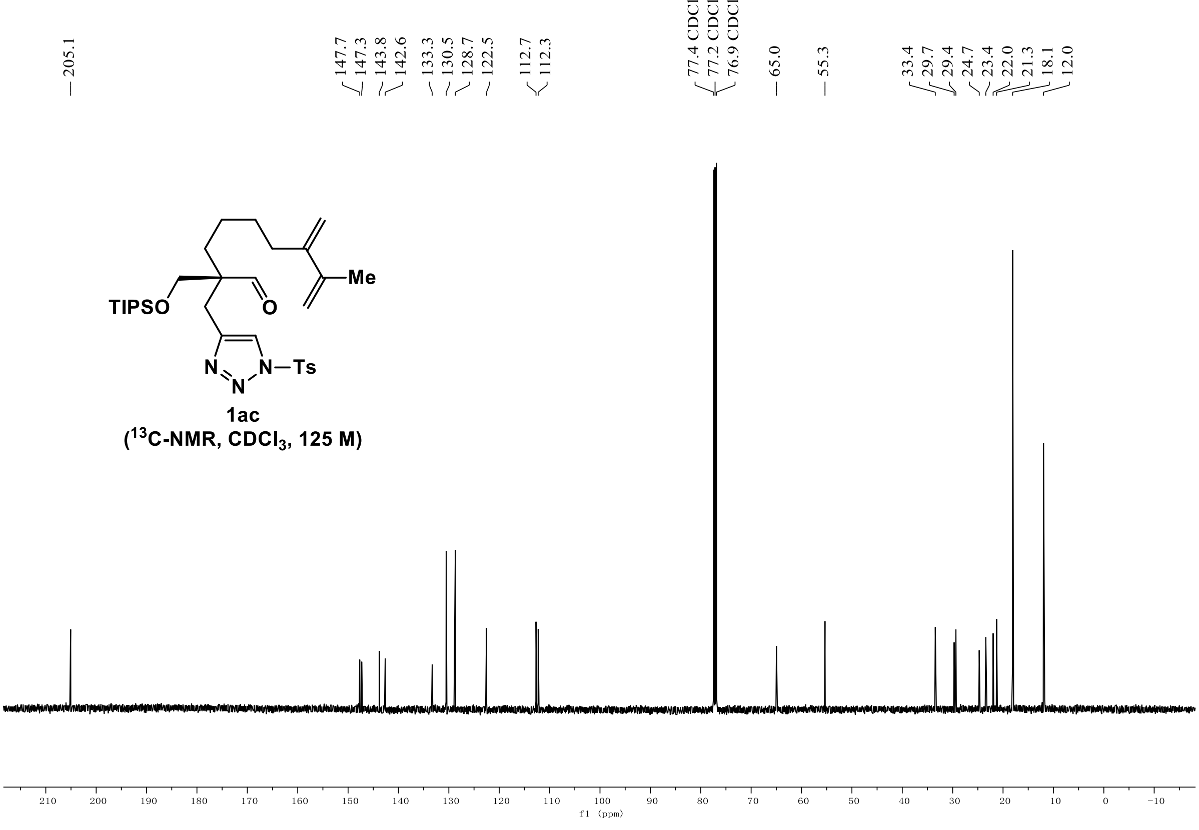
205.1
147.7 147.3 143.8 142.6 133.3 130.5 128.7 122.5 112.7 112.3
77.4 CDCl3 77.2 CDCl3 76.9 CDCl3
65.0 55.3
33.4 29.7 29.4 24.7 23.4 22.0 21.3 18.1 12.0
TIPSO
O
Me
N
N
N—Ts
1ac
(¹³C-NMR, CDCl3, 125 M)
f1 (ppm)

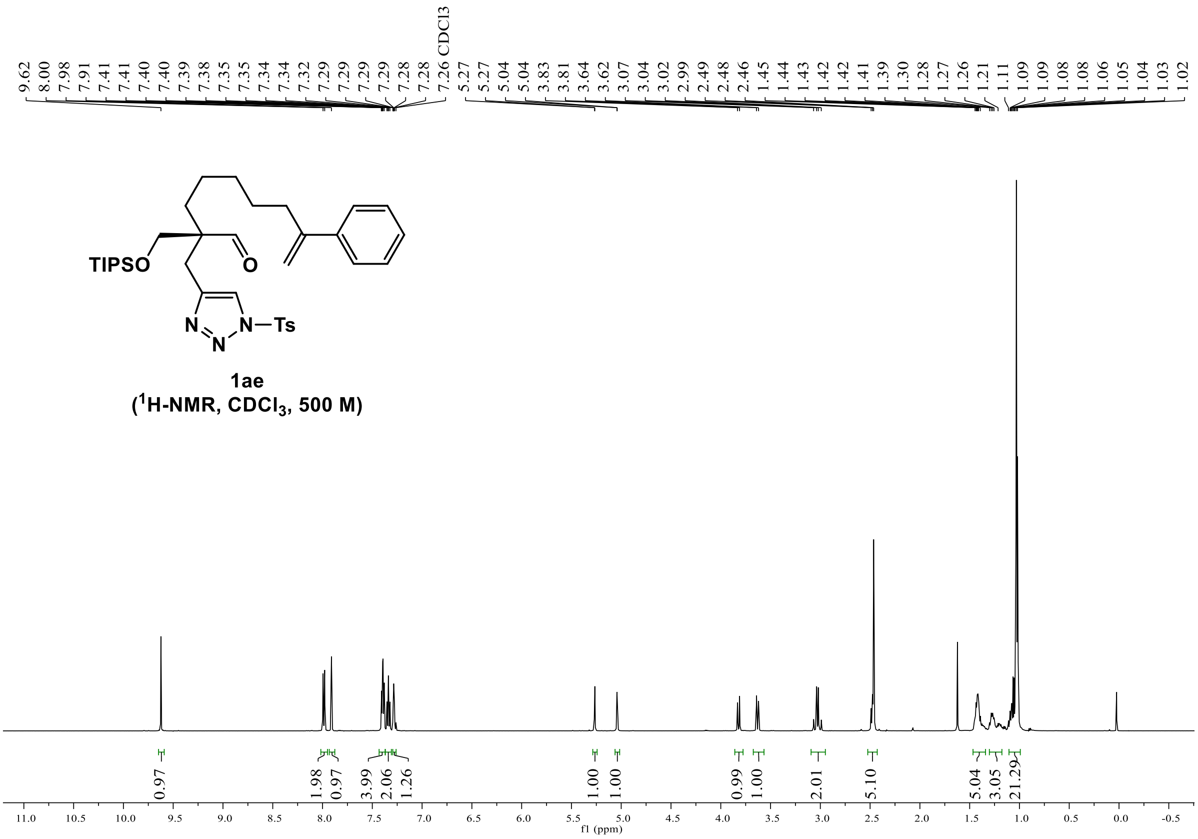
9.62 8.00 7.98 7.91 7.41 7.41 7.40 7.39 7.38 7.35 7.35 7.34 7.34 7.32 7.29 7.29 7.29 7.29 7.28 7.28 7.26 CDCl3 5.27 5.27 5.04 5.04 3.83 3.81 3.64 3.62 3.07 3.04 3.02 2.99 2.49 2.48 2.46 1.45 1.44 1.43 1.42 1.42 1.41 1.39 1.30 1.28 1.27 1.26 1.21 1.11 1.09 1.09 1.08 1.08 1.06 1.05 1.04 1.03 1.02
TIPSO
O
N
N
N—Ts
1ae
(^{1}H-NMR, $CDCl_3$, 500 M)
0.97
1.98
0.97
3.99
2.06
1.26
1.00
1.00
0.99
1.00
2.01
5.10
5.04
3.05
21.29
11.0 10.5 10.0 9.5 9.0 8.5 8.0 7.5 7.0 6.5 6.0 5.5 5.0 4.5 4.0 3.5 3.0 2.5 2.0 1.5 1.0 0.5 0.0 -0.5
f1 (ppm)

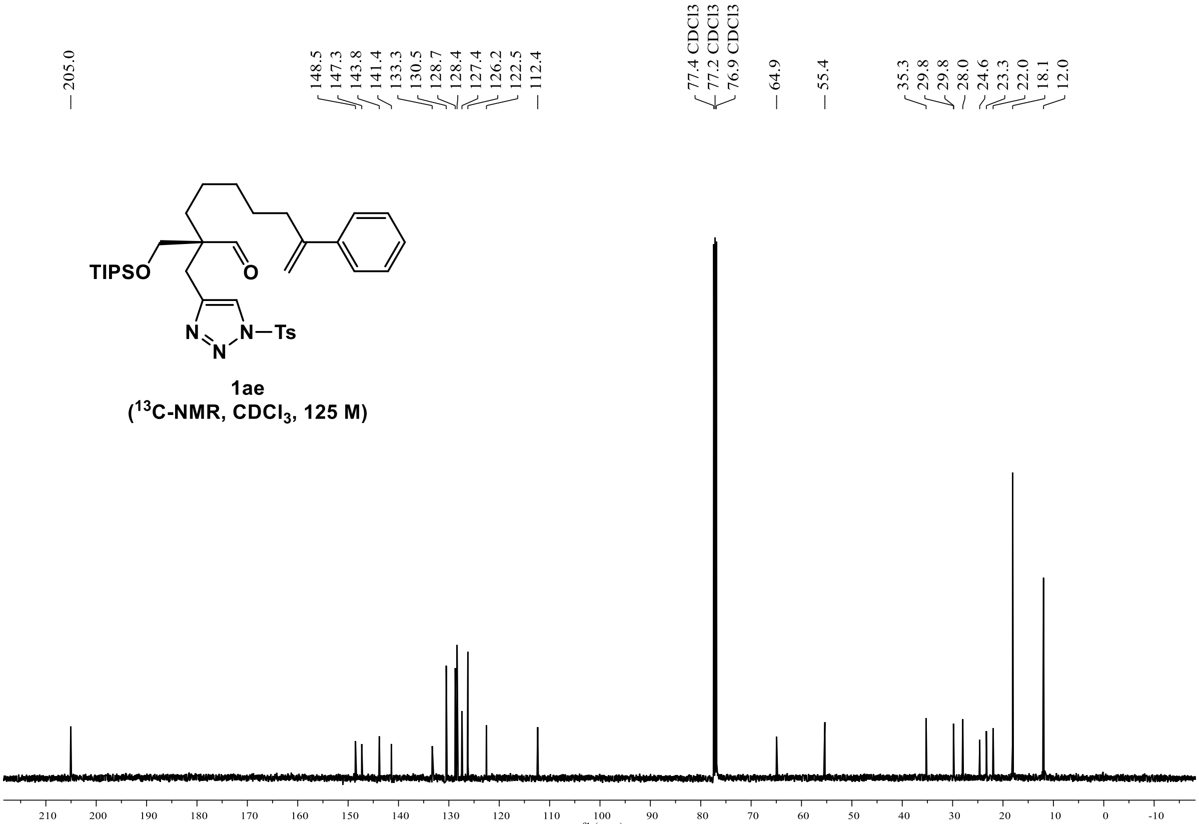
205.0
148.5 147.3 143.8 141.4 133.3 130.5 128.7 128.4 127.4 126.2 122.5 112.4
77.4 CDCl3 77.2 CDCl3 76.9 CDCl3
64.9
55.4
35.3 29.8 29.8 28.0 24.6 23.3 22.0 18.1 12.0
TIPSO
O
N
N
N—Ts
1ae
(^{13}C-NMR, $CDCl_3$, 125 M)
210 200 190 180 170 160 150 140 130 120 110 100 90 80 70 60 50 40 30 20 10 0 -10
f1 (ppm)

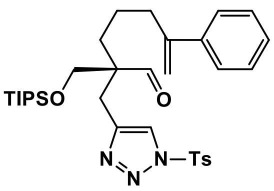

1af

(^{1}H-NMR, $CDCl_3$, 500 M)

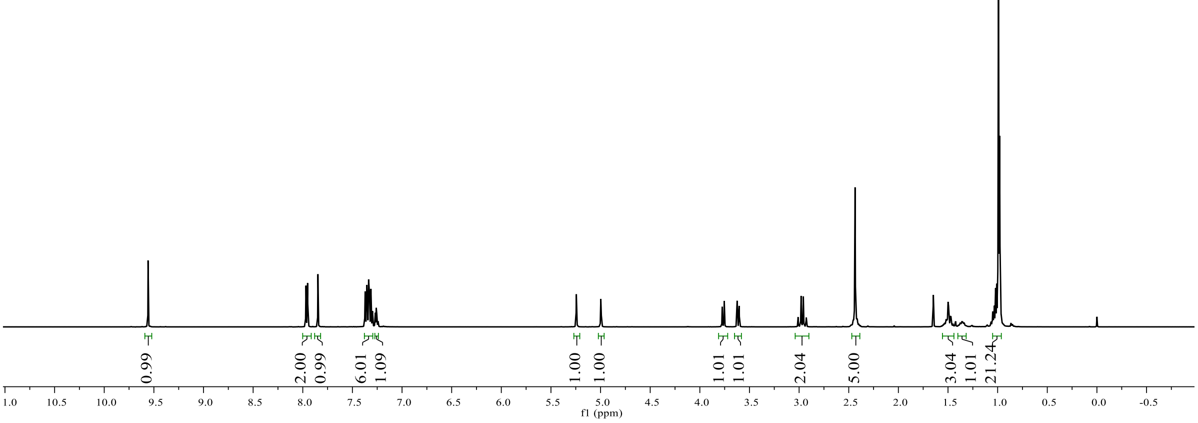

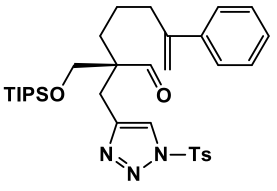

1af

(^{13}C-NMR, $CDCl_3$, 125 M)

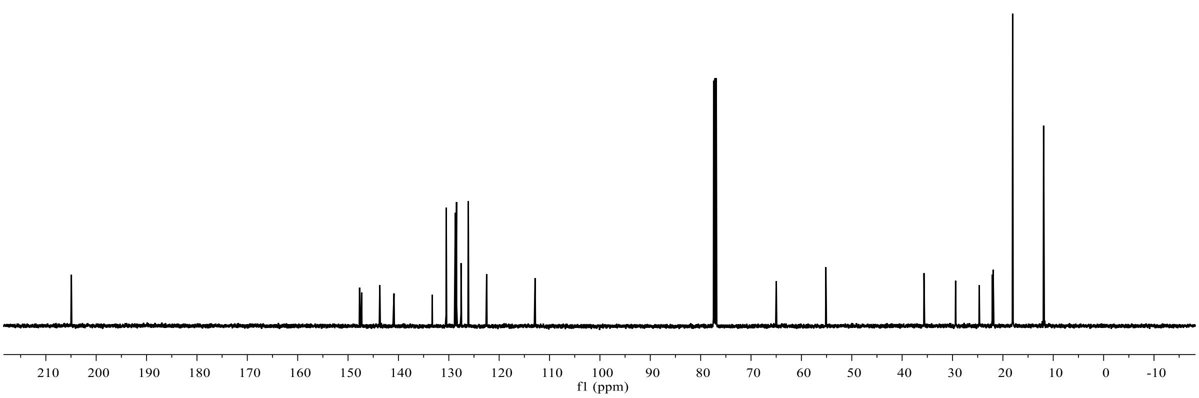

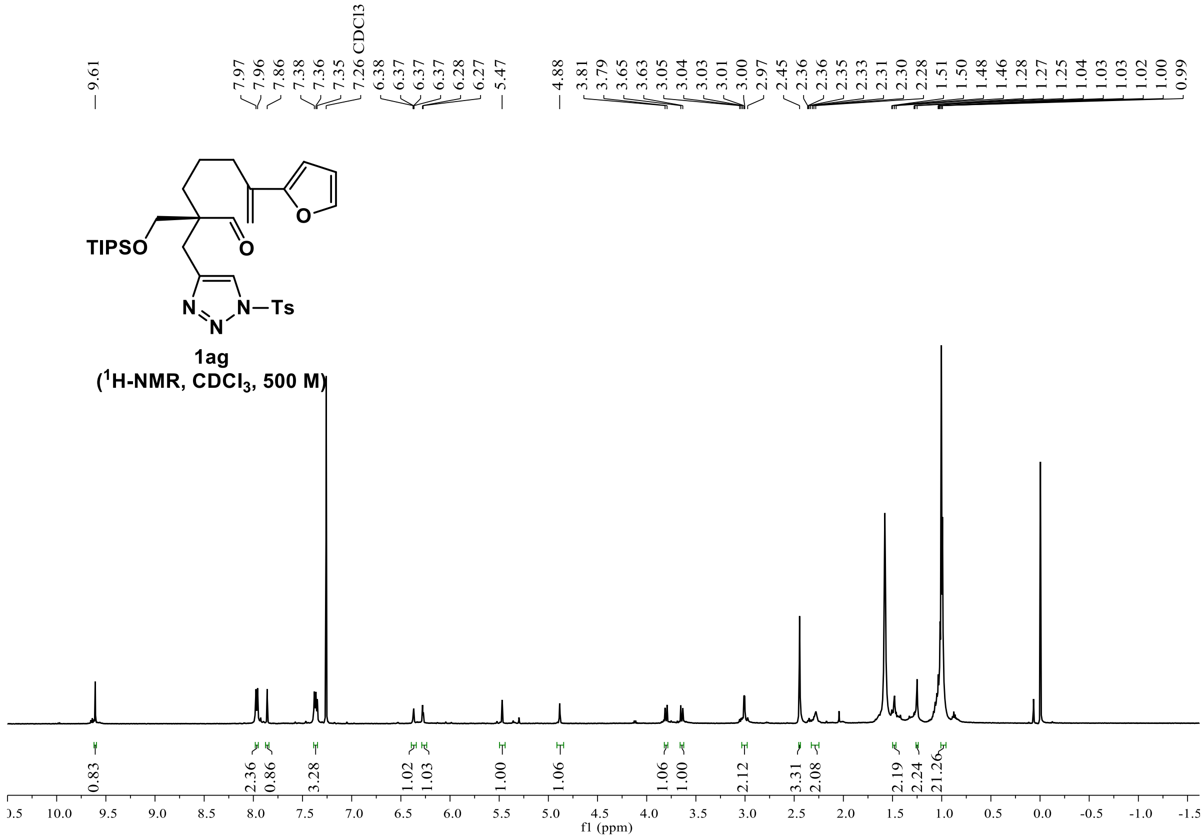
9.61
7.97
7.96
7.86
7.38
7.36
7.35
7.26 CDCl3
6.38
6.37
6.37
6.37
6.28
6.27
5.47
4.88
3.81
3.79
3.65
3.63
3.05
3.04
3.03
3.01
3.00
2.97
2.45
2.36
2.36
2.35
2.33
2.31
2.30
2.28
1.51
1.50
1.48
1.46
1.28
1.27
1.25
1.04
1.03
1.03
1.02
1.00
0.99
TIPSO
O
O
N
N
N—Ts
1ag
(1H-NMR, CDCl3, 500 M)
0.83
2.36
0.86
3.28
1.02
1.03
1.00
1.06
1.06
1.00
2.12
3.31
2.08
2.19
2.24
21.26
f1 (ppm)

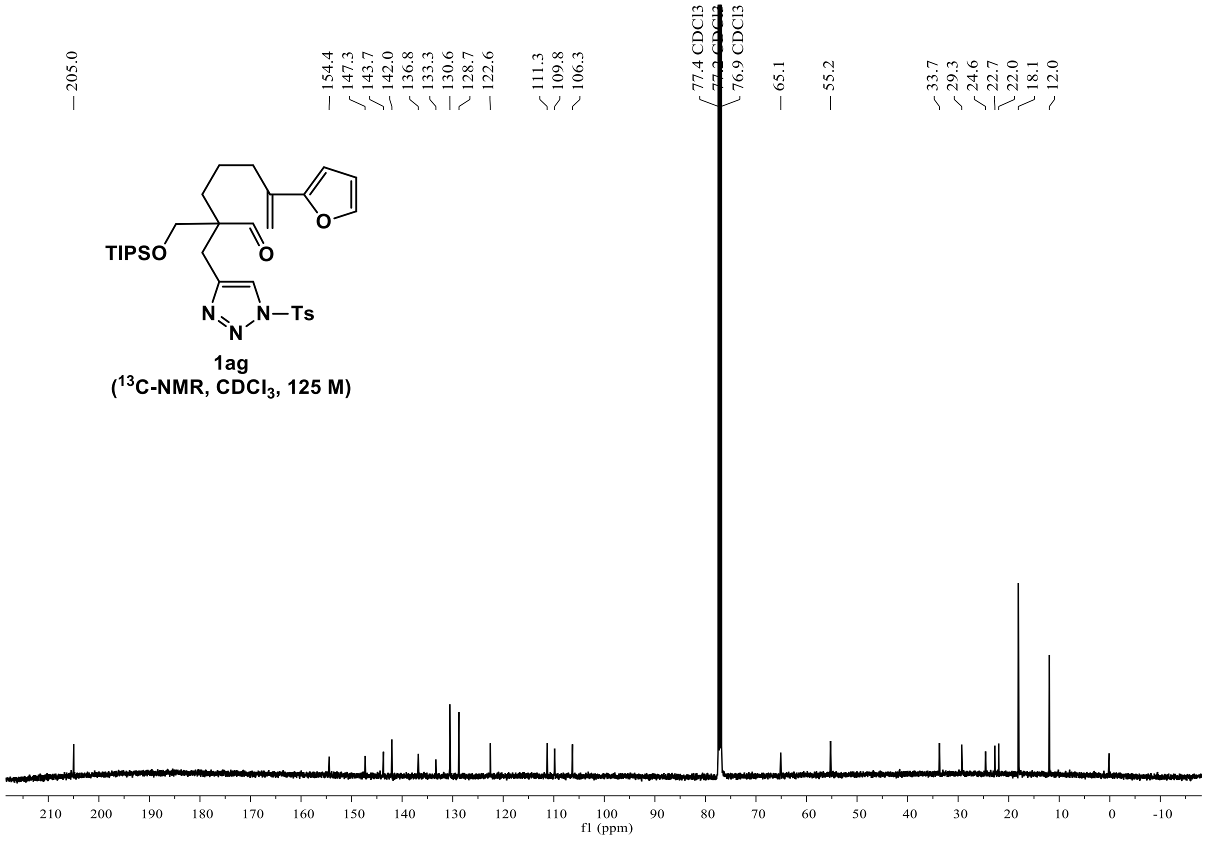
205.0
154.4
147.3
143.7
142.0
136.8
133.3
130.6
128.7
122.6
111.3
109.8
106.3
77.4 CDCl3
77.2 CDCl3
76.9 CDCl3
65.1
55.2
33.7
29.3
24.6
22.7
22.0
18.1
12.0
TIPSO
O
O
N
N
N—Ts
1ag
(13C-NMR, CDCl3, 125 M)
f1 (ppm)

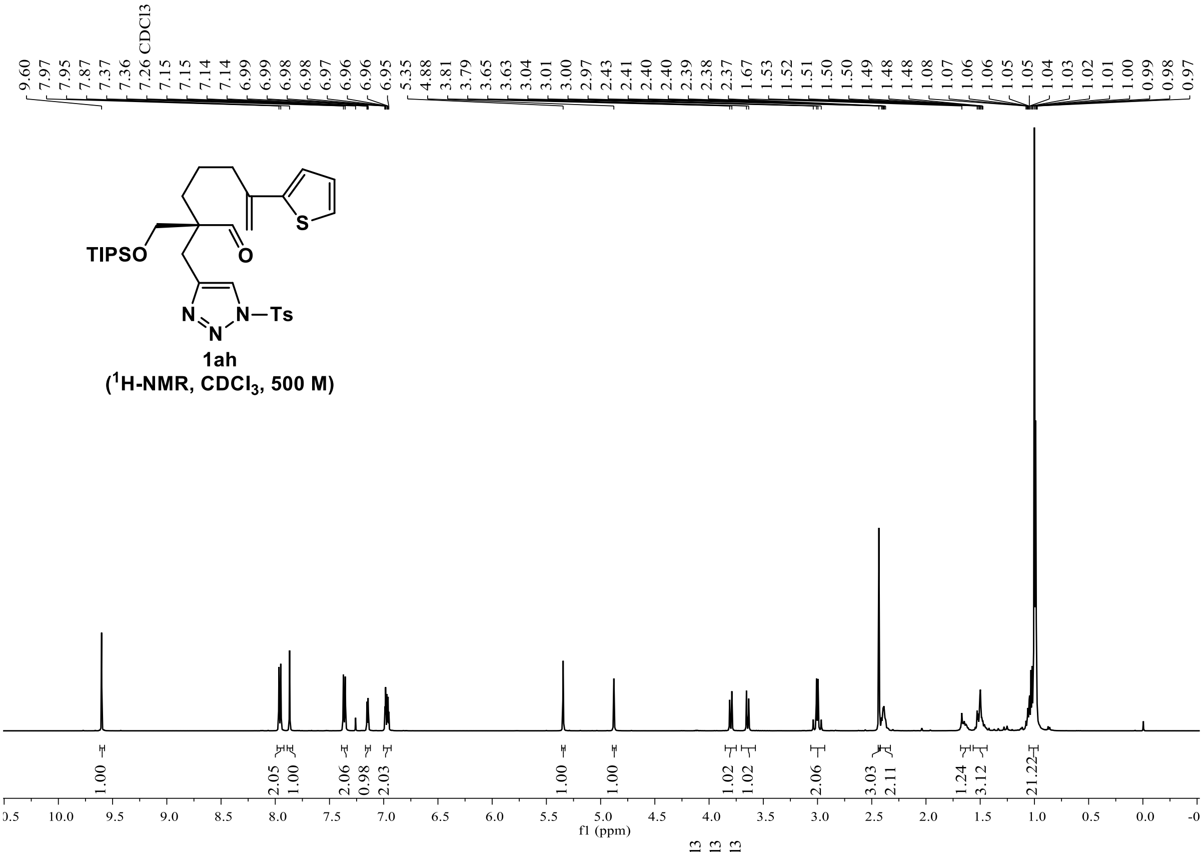
TIPSO
O
S
N
N
N–Ts
1ah
(¹H-NMR, CDCl3, 500 M)
9.60 7.97 7.95 7.87 7.37 7.36 7.26 CDCl3 7.15 7.15 7.14 7.14 6.99 6.99 6.98 6.98 6.97 6.96 6.96 6.95 5.35 4.88 3.81 3.79 3.65 3.63 3.04 3.01 3.00 2.97 2.43 2.41 2.40 2.40 2.39 2.38 2.37 1.67 1.53 1.52 1.51 1.50 1.50 1.49 1.48 1.48 1.08 1.07 1.06 1.06 1.05 1.05 1.04 1.03 1.02 1.01 1.00 0.99 0.98 0.97
1.00 2.05 1.00 2.06 0.98 2.03 1.00 1.00 1.02 1.02 2.06 3.03 2.11 1.24 3.12 21.22
f1 (ppm)

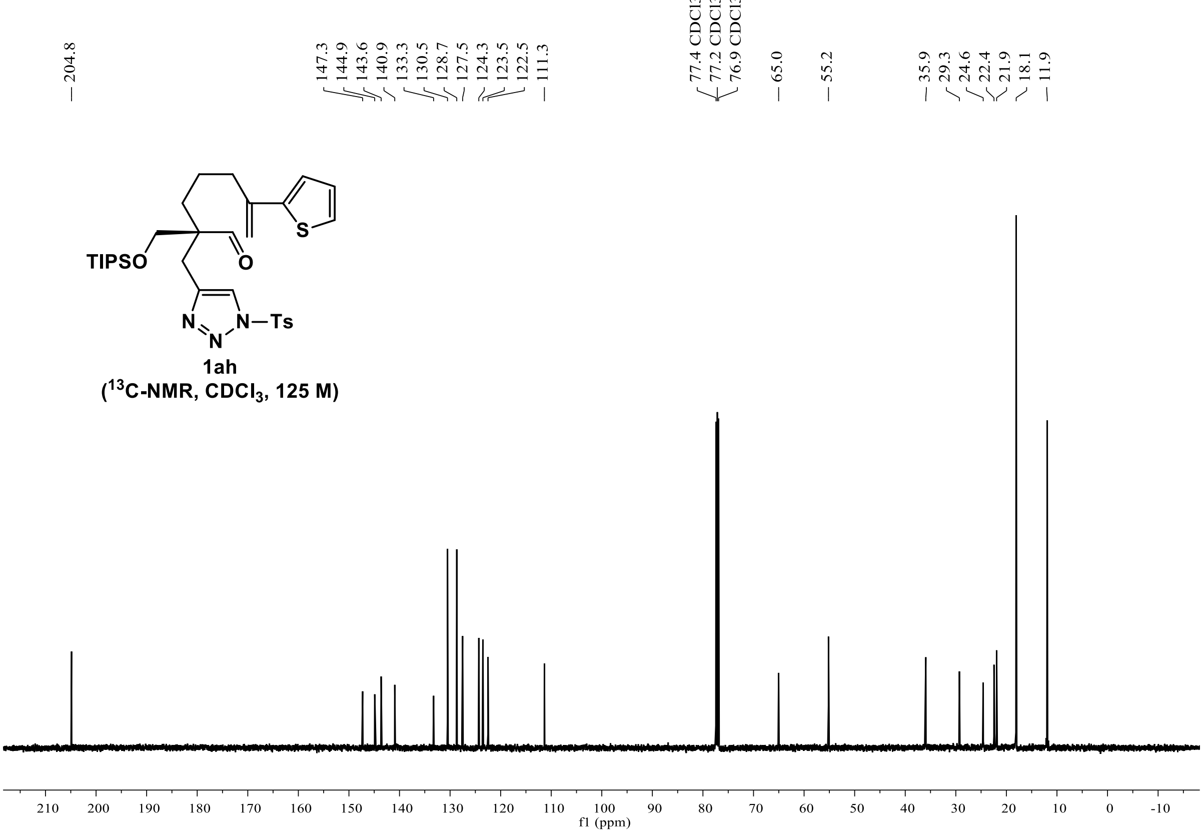
TIPSO
O
S
N
N
N–Ts
1ah
(¹³C-NMR, CDCl3, 125 M)
204.8 147.3 144.9 143.6 140.9 133.3 130.5 128.7 127.5 124.3 123.5 122.5 111.3 77.4 CDCl3 77.2 CDCl3 76.9 CDCl3 65.0 55.2 35.9 29.3 24.6 22.4 21.9 18.1 11.9
f1 (ppm)

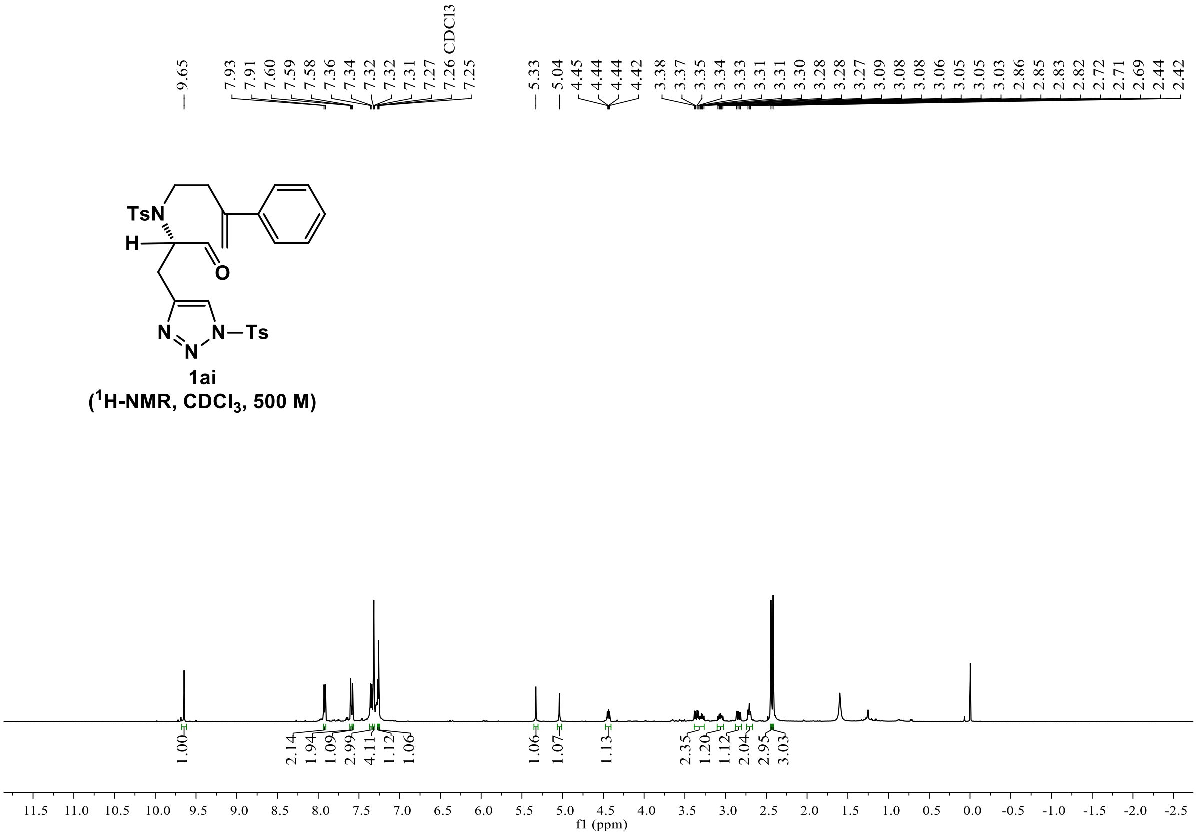

1ai
(^{1}H-NMR, $CDCl_3$, 500 M)

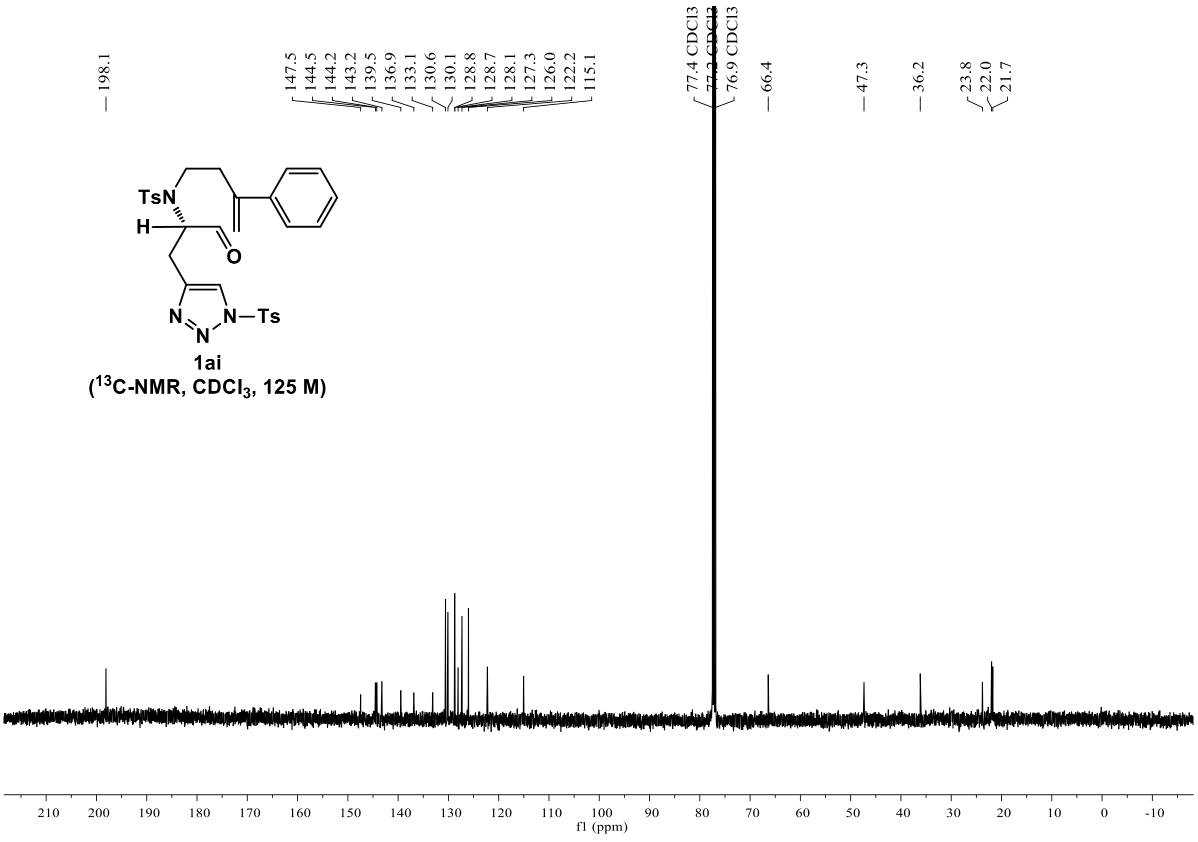

1ai
(^{13}C-NMR, $CDCl_3$, 125 M)

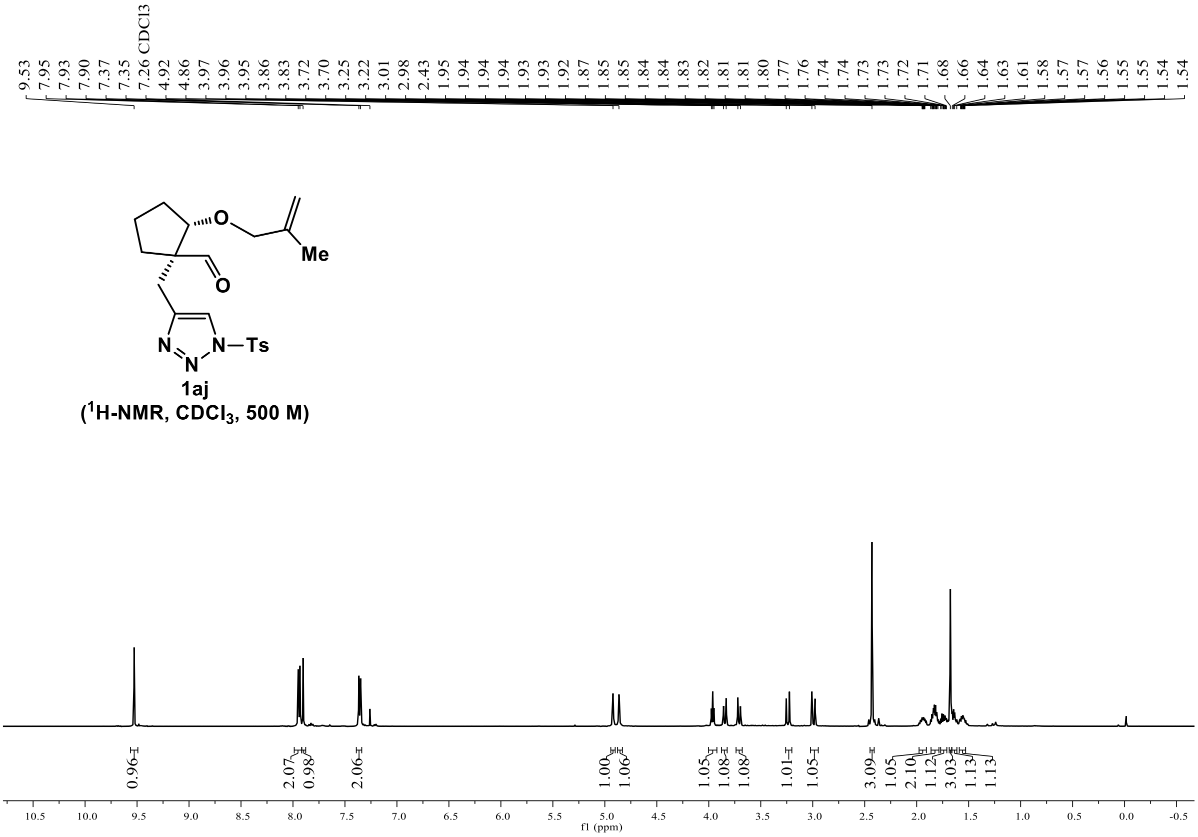

1aj
(^{1}H-NMR, $CDCl_3$, 500 M)

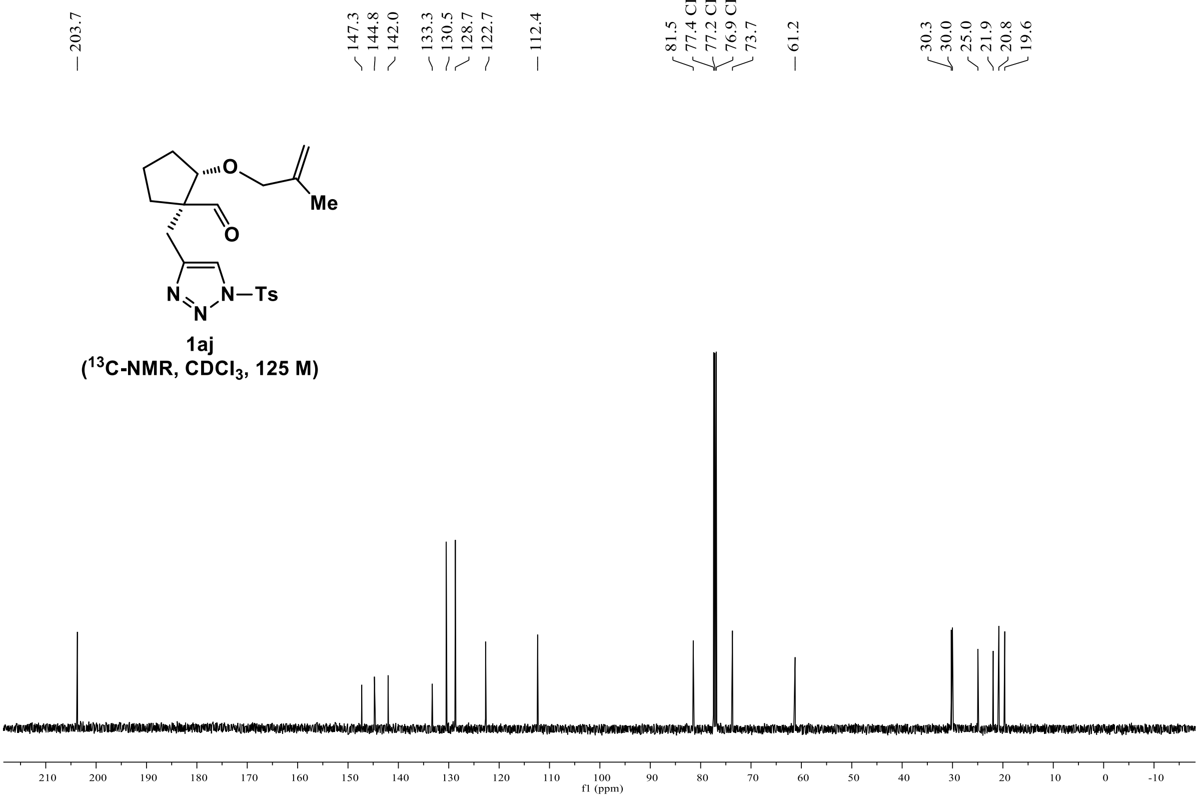

1aj
(^{13}C-NMR, $CDCl_3$, 125 M)

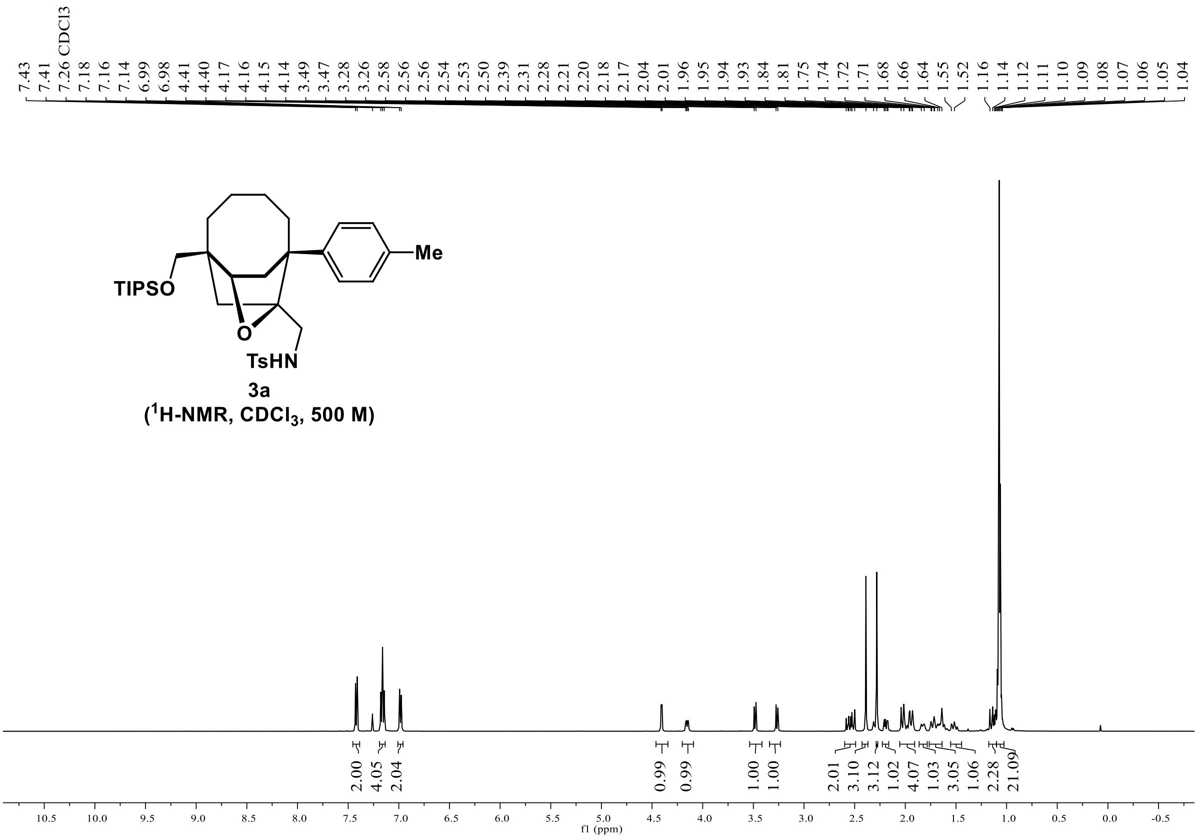

3a
(^{1}H-NMR, $CDCl_3$, 500 M)

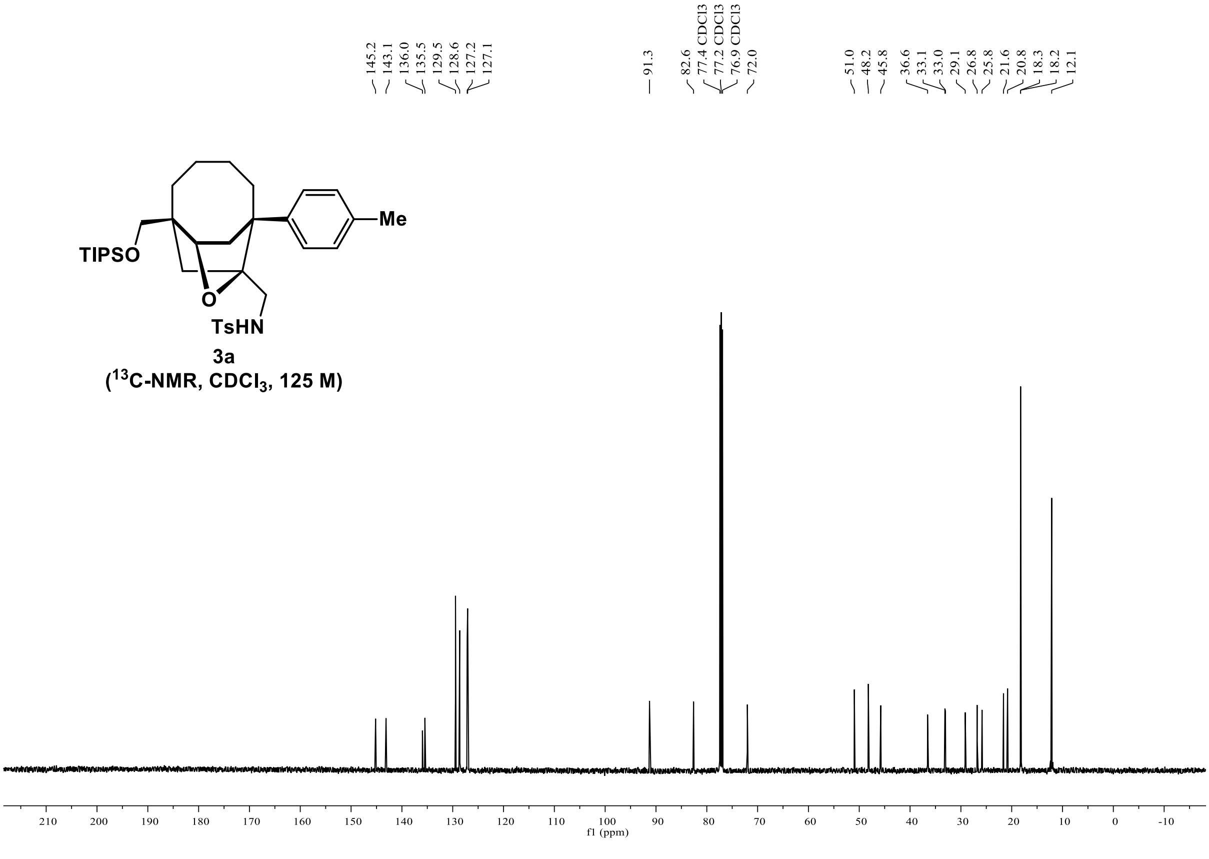

3a
(^{13}C-NMR, $CDCl_3$, 125 M)

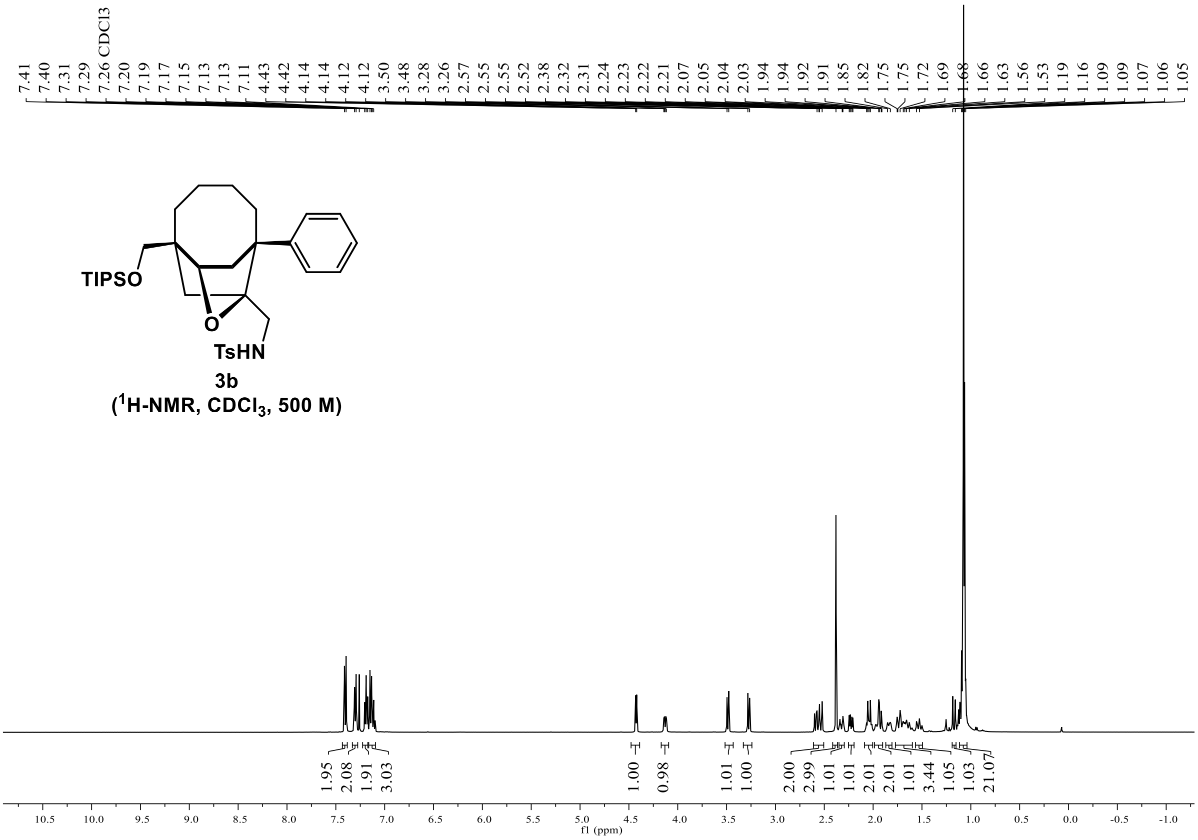

3b
(^{1}H-NMR, $CDCl_3$, 500 M)

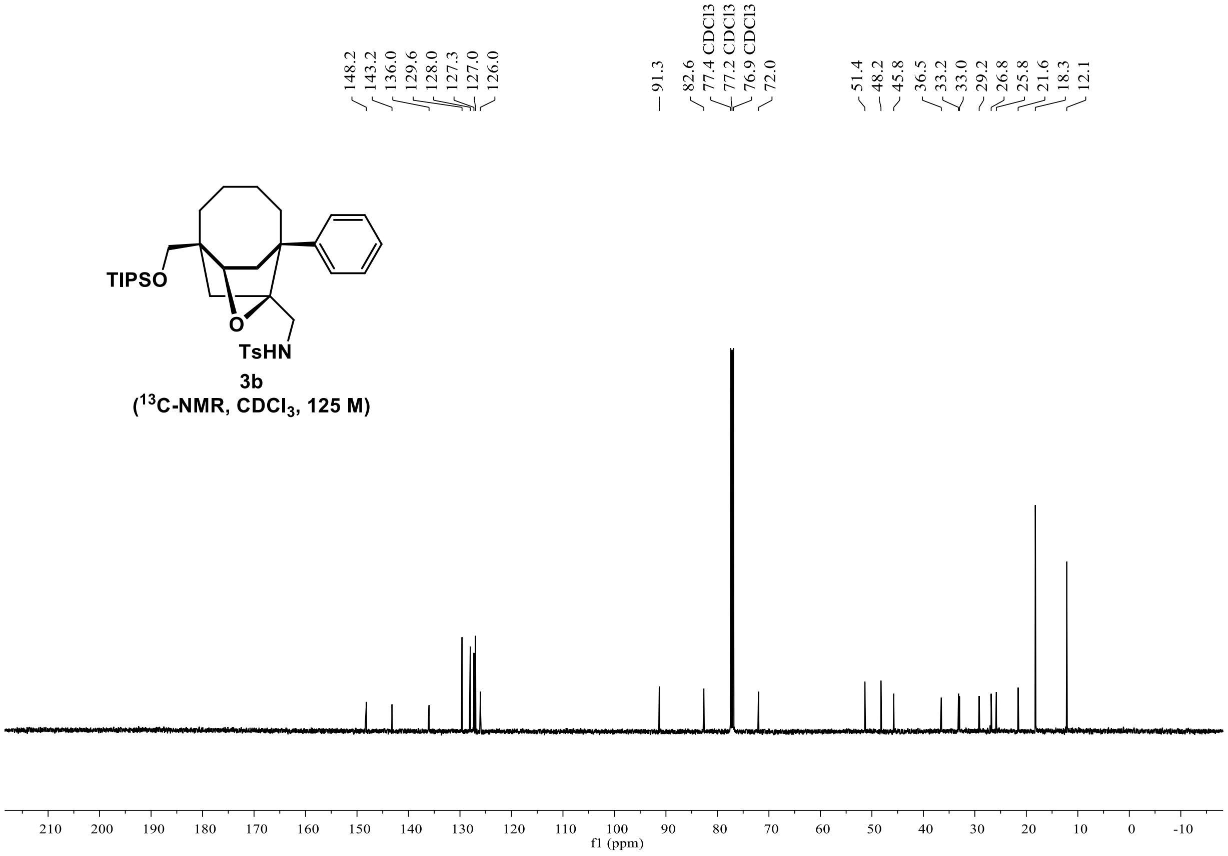

3b
(^{13}C-NMR, $CDCl_3$, 125 M)

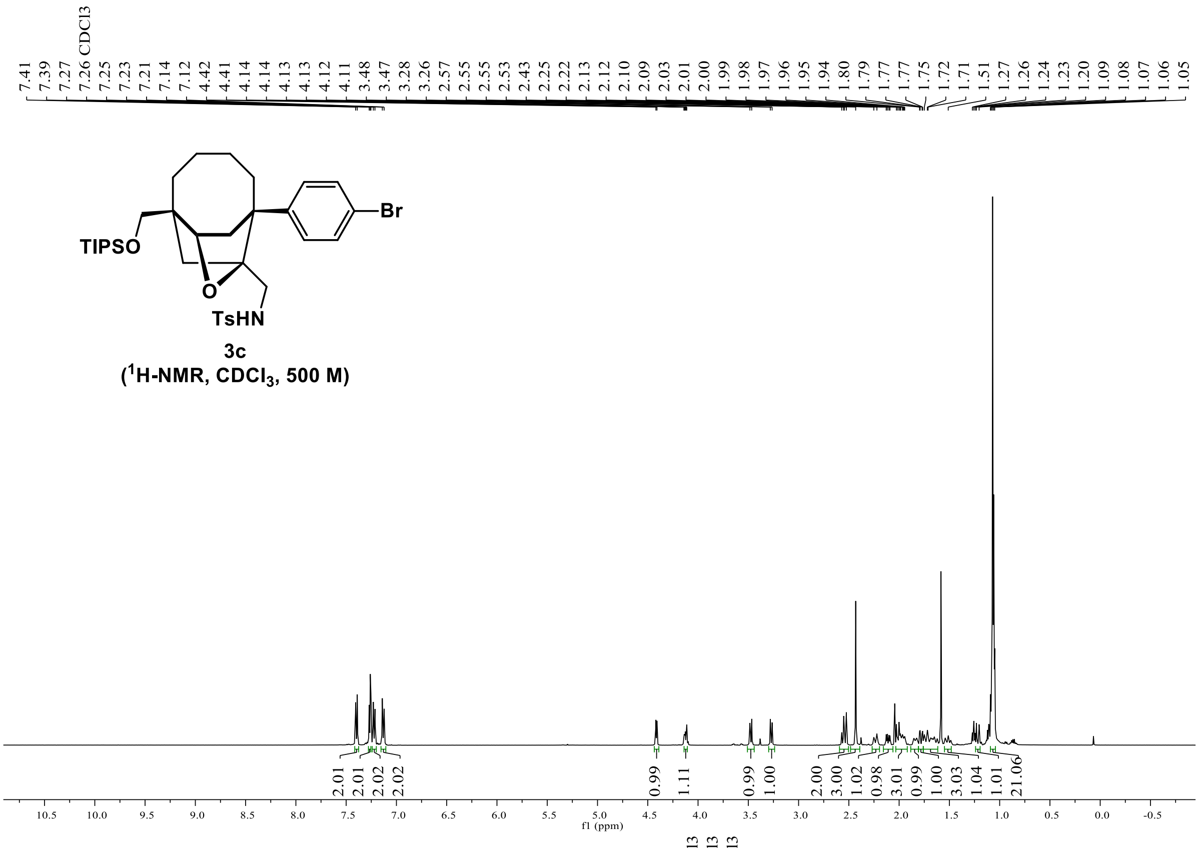

3c
(^{1}H-NMR, $CDCl_3$, 500 M)

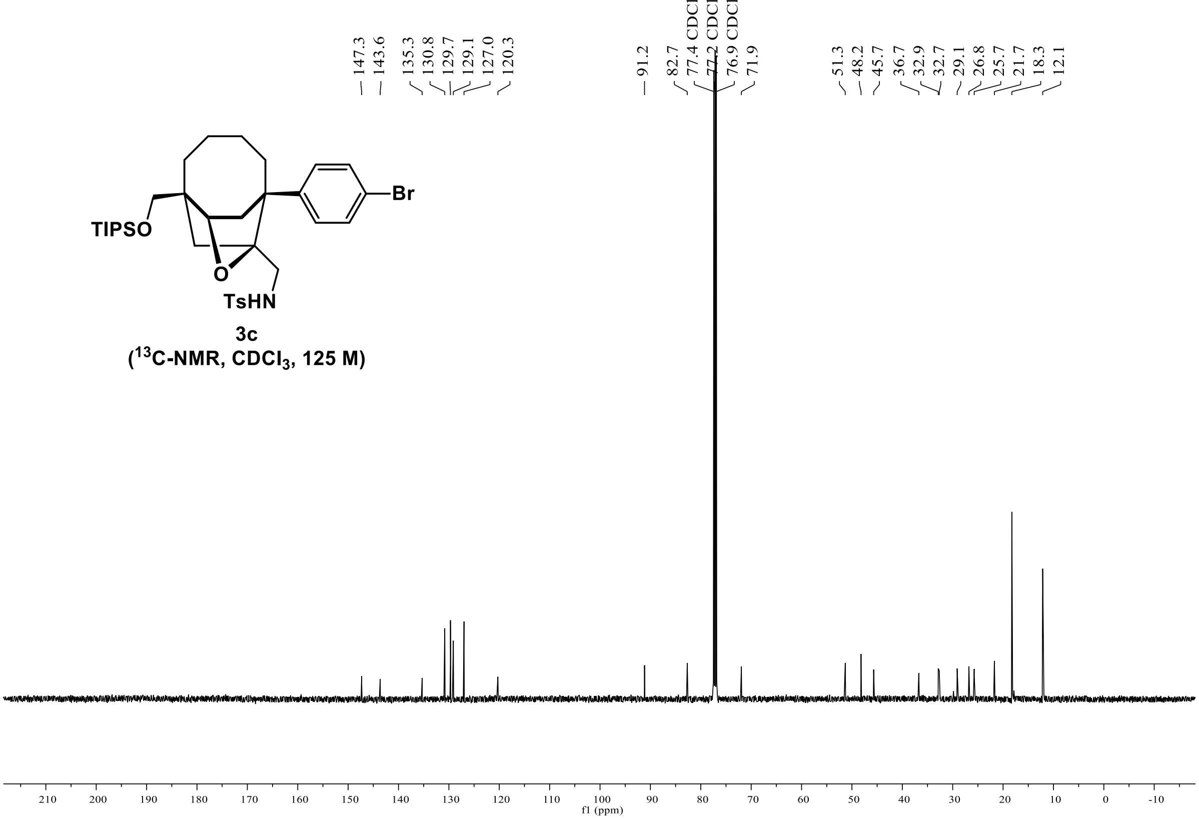

3c
(^{13}C-NMR, $CDCl_3$, 125 M)

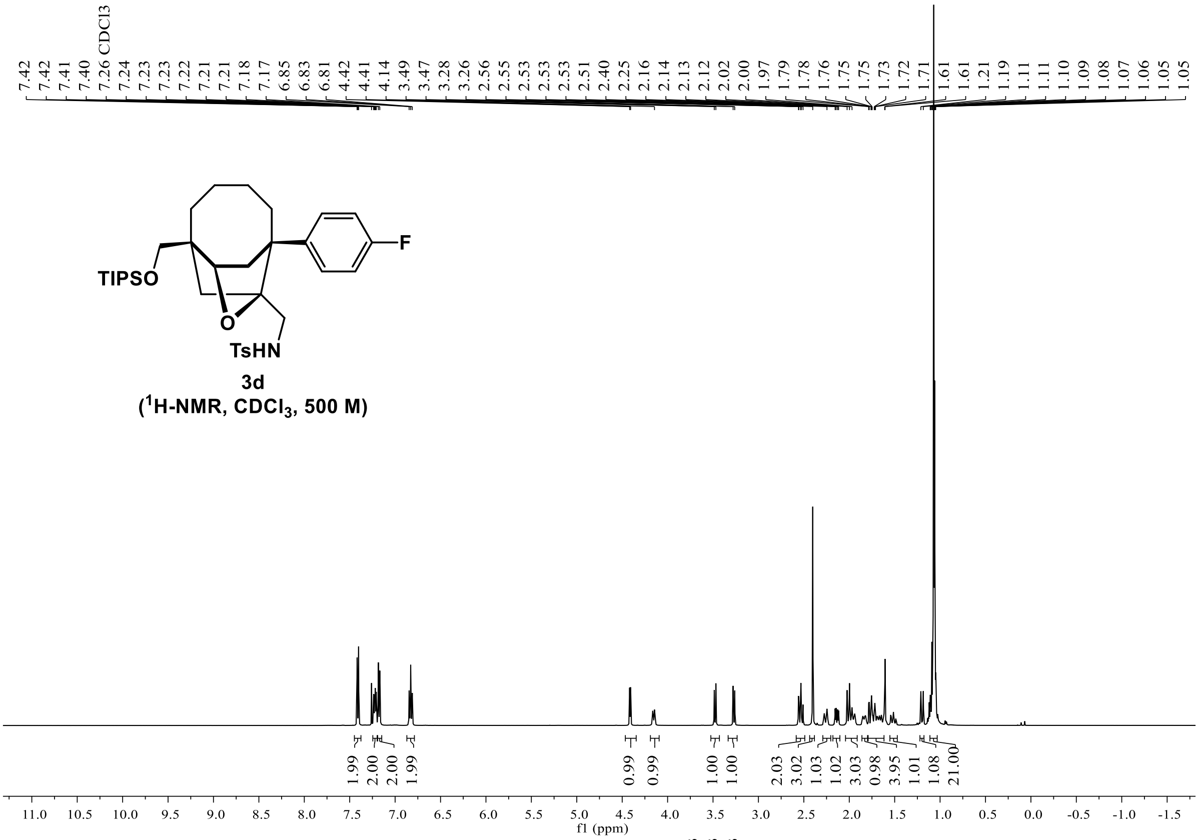

3d
(^{1}H-NMR, $CDCl_3$, 500 M)

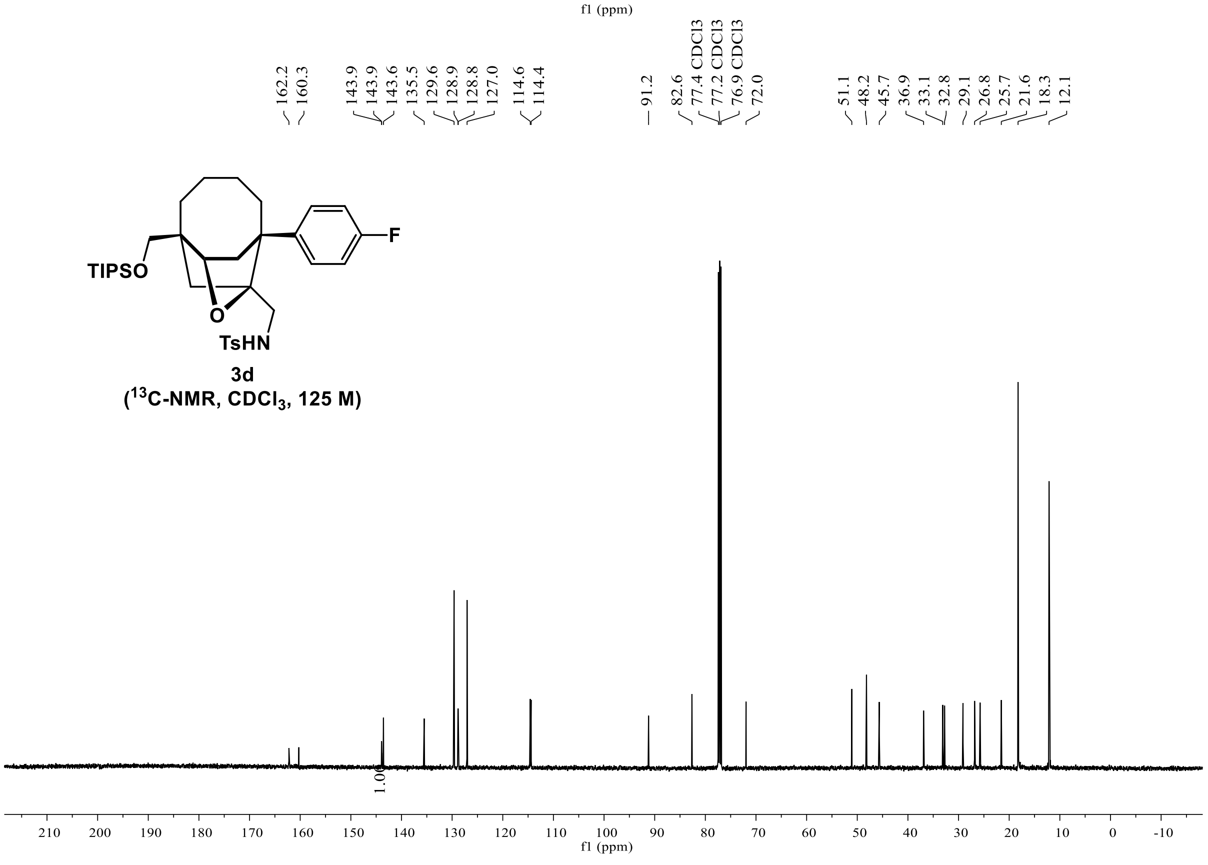

3d
(^{13}C-NMR, $CDCl_3$, 125 M)

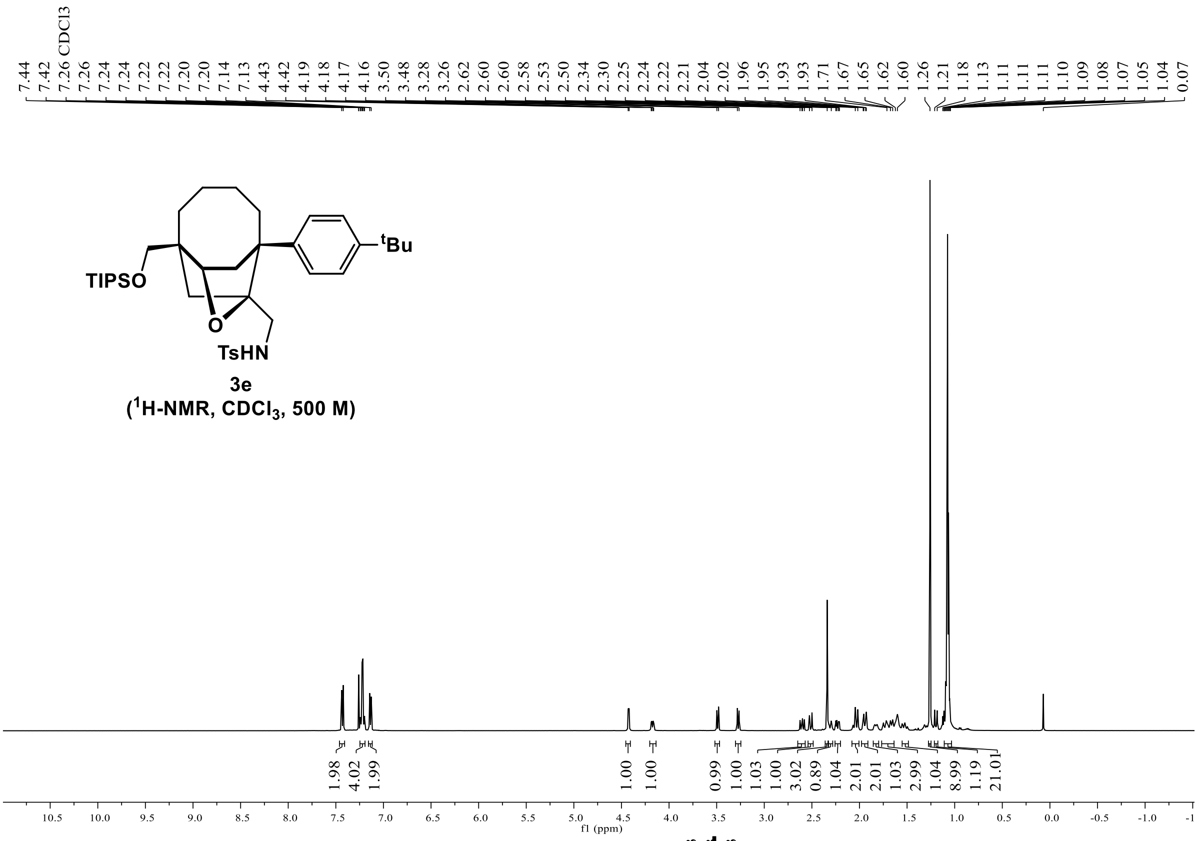

3e
(^{1}H-NMR, $CDCl_3$, 500 M)

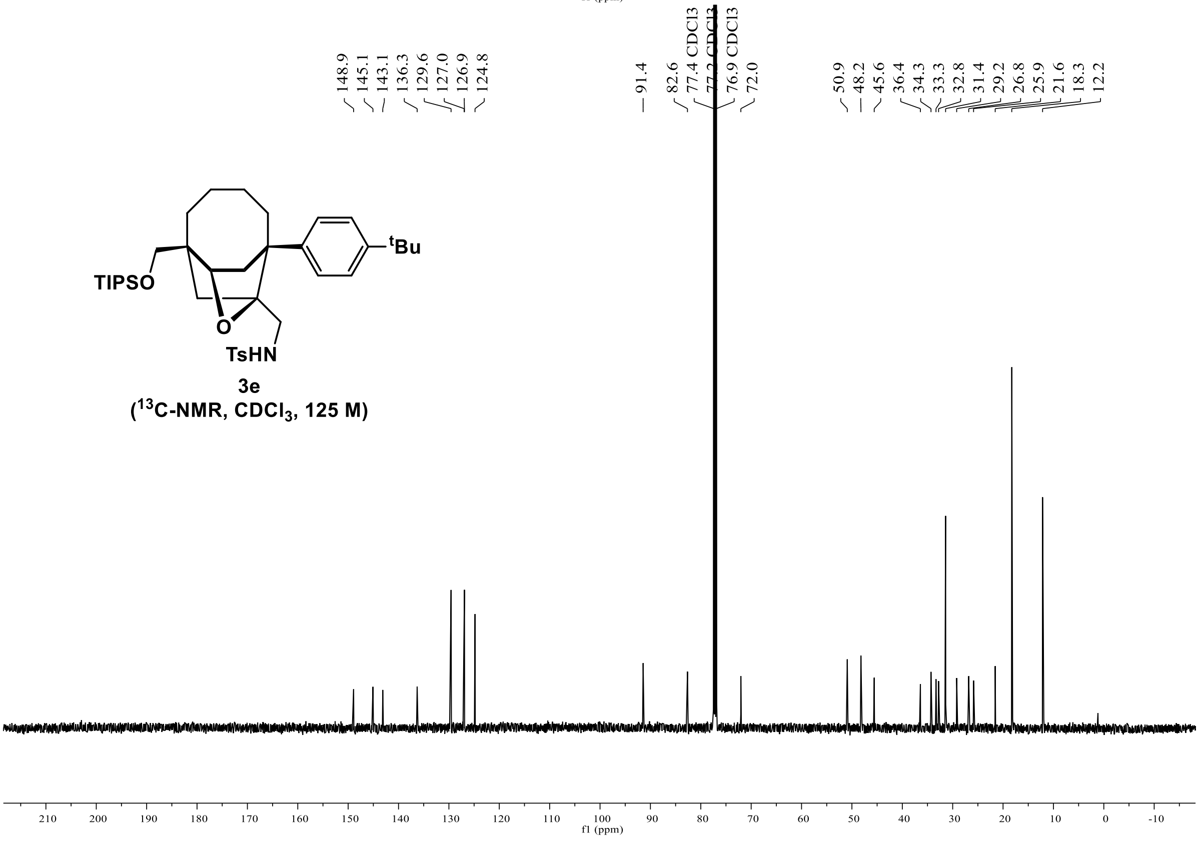

3e
(^{13}C-NMR, $CDCl_3$, 125 M)

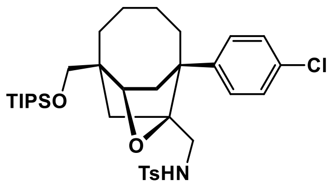

3f
(^{1}H-NMR, $CDCl_3$, 500 M)

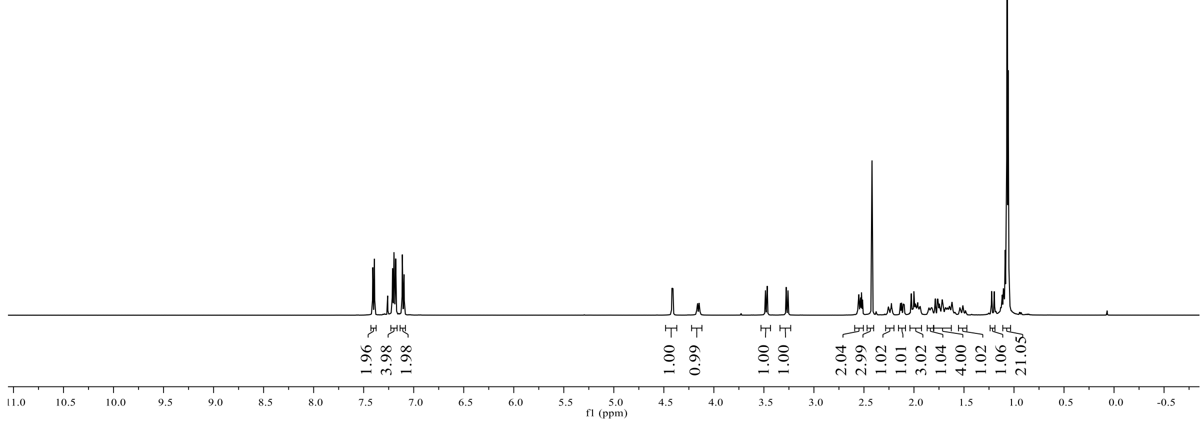

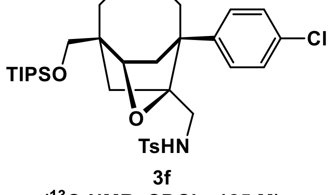

3f
(^{13}C-NMR, $CDCl_3$, 125 M)

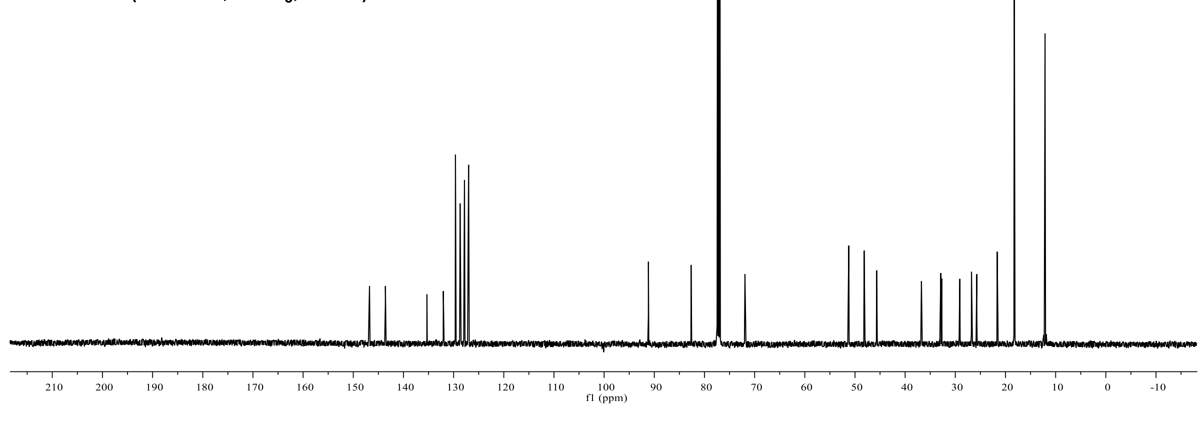

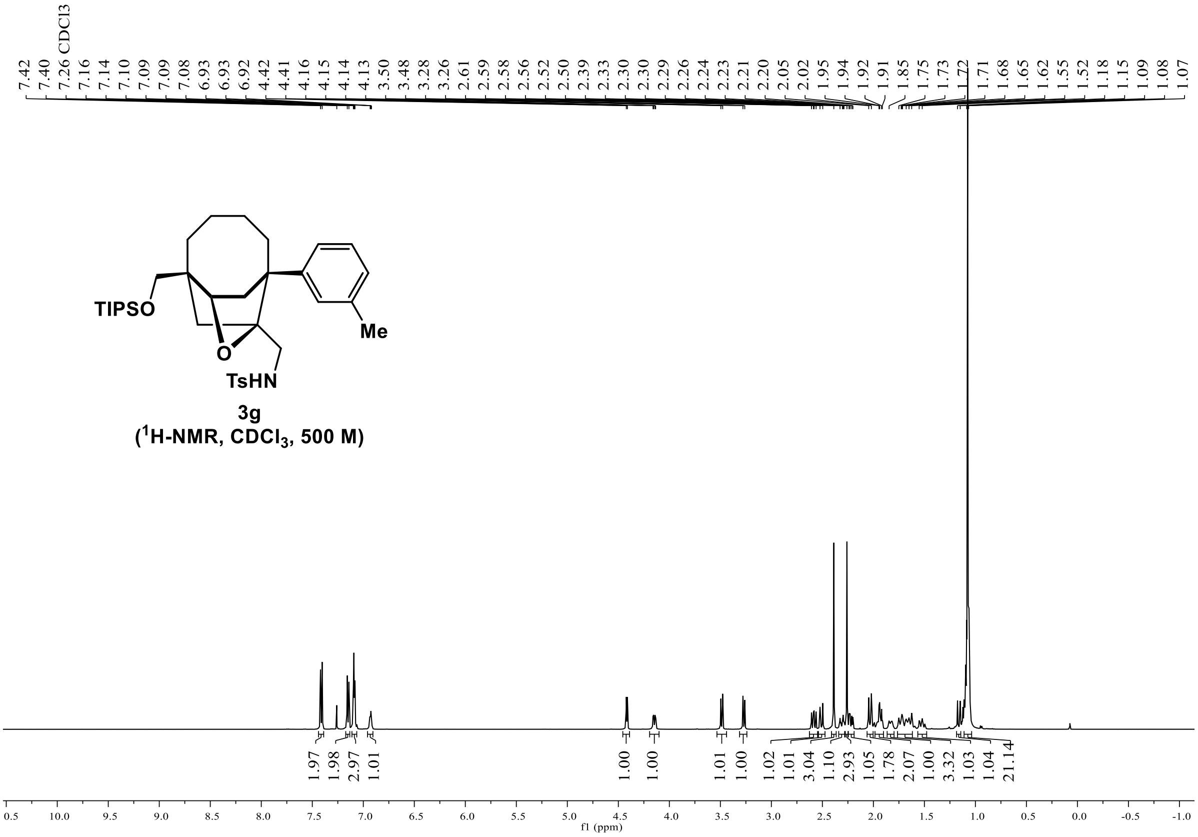

3g
(^{1}H-NMR, $CDCl_3$, 500 M)

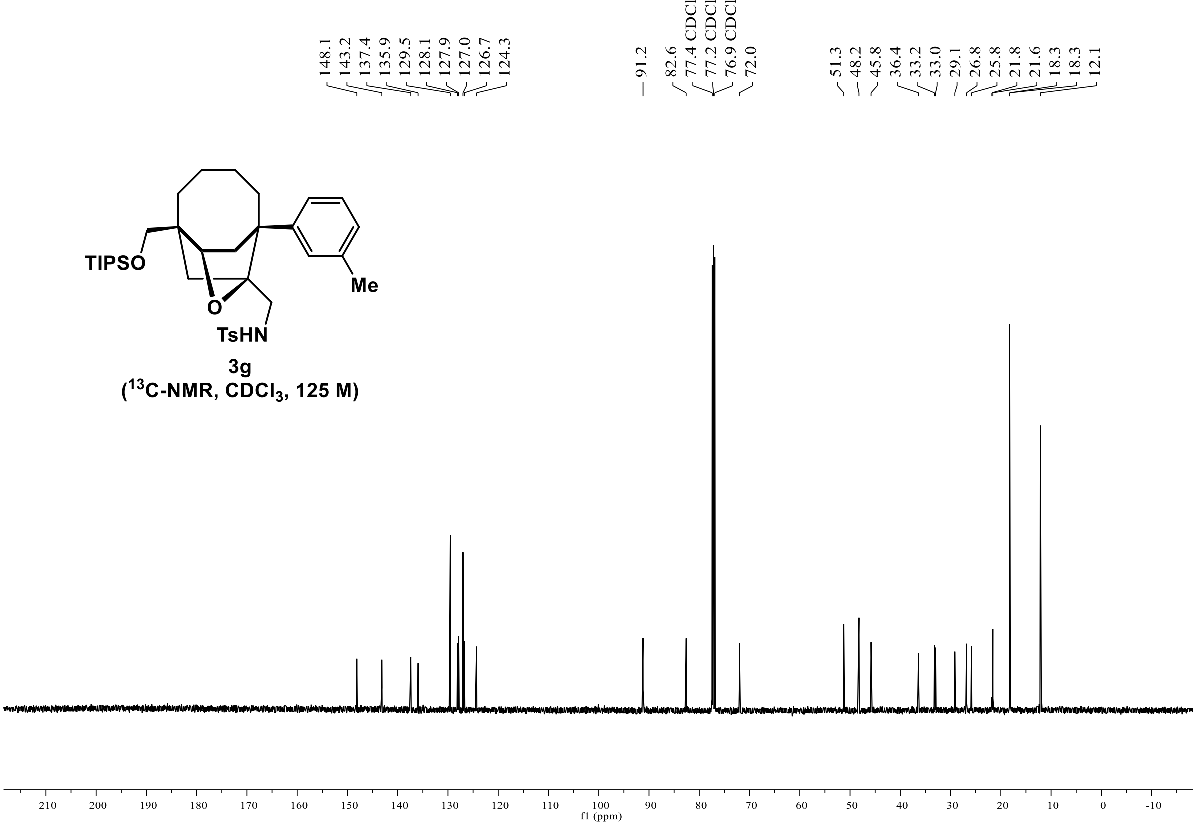

3g
(^{13}C-NMR, $CDCl_3$, 125 M)

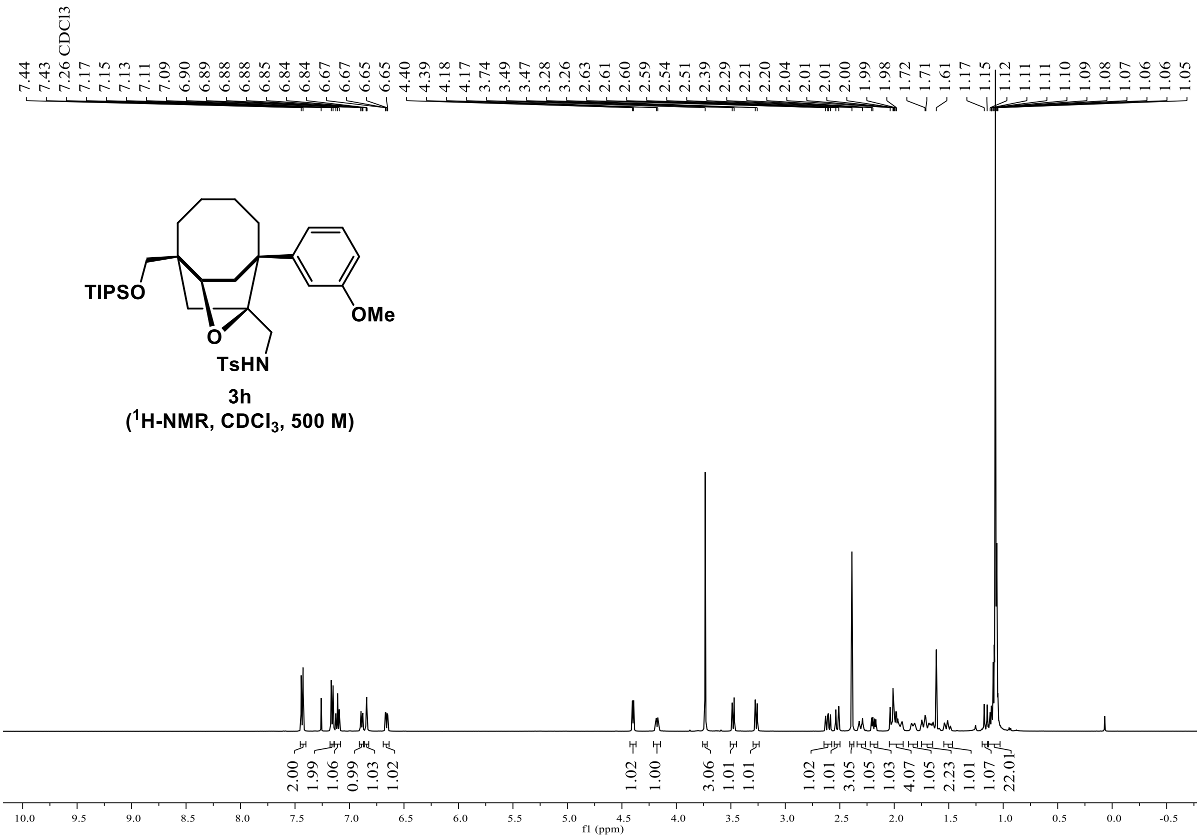

3h
(^{1}H-NMR, $CDCl_3$, 500 M)

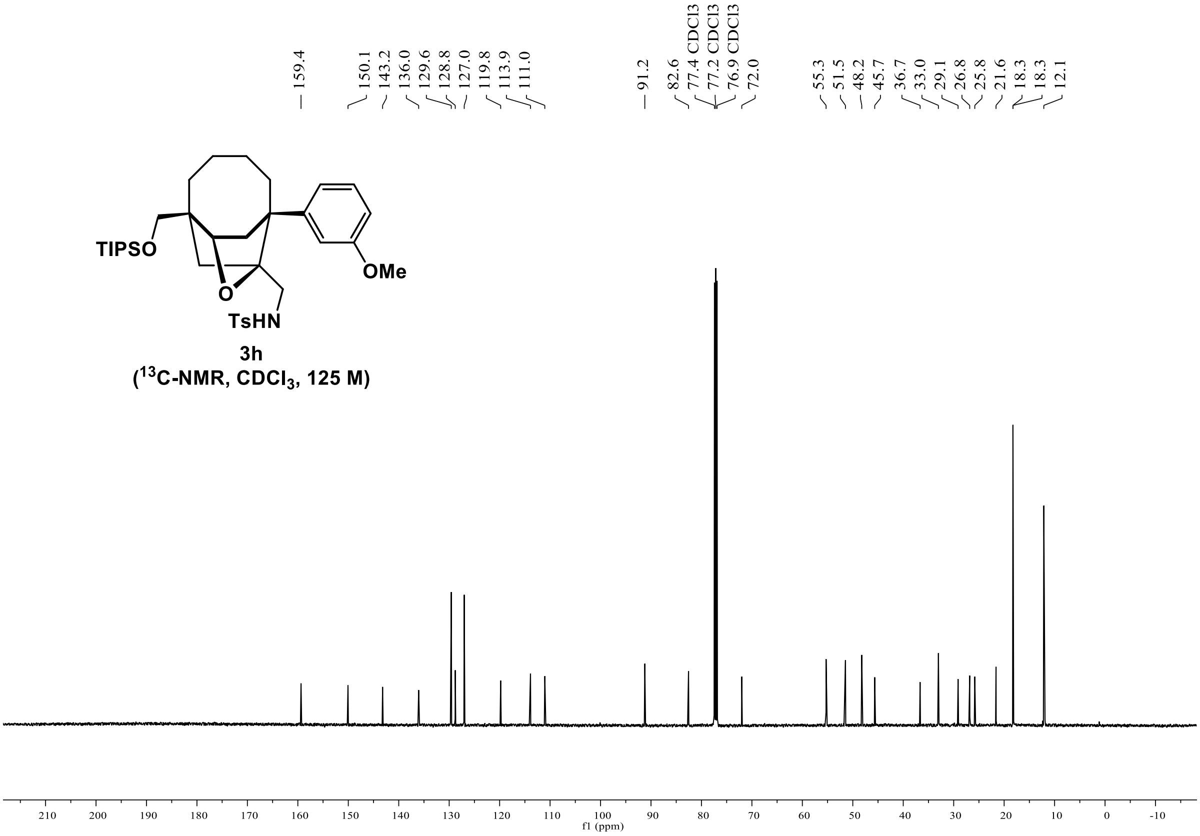

3h
(^{13}C-NMR, $CDCl_3$, 125 M)

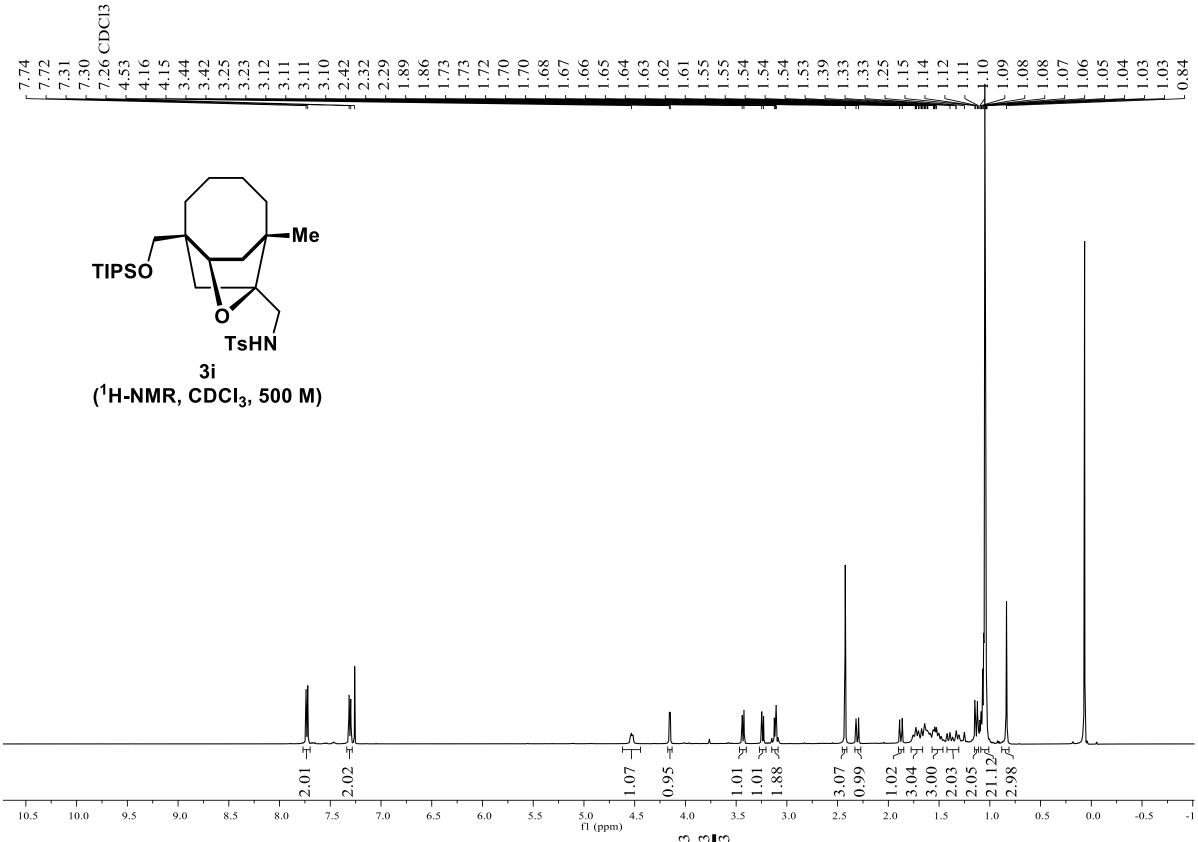
7.74 7.72 7.31 7.30 7.26 CDCl3 4.53 4.16 4.15 3.44 3.42 3.25 3.23 3.12 3.11 3.11 3.10 2.42 2.32 2.29 1.89 1.86 1.73 1.73 1.72 1.70 1.70 1.68 1.67 1.66 1.65 1.64 1.63 1.62 1.61 1.55 1.55 1.54 1.54 1.54 1.53 1.39 1.33 1.33 1.25 1.15 1.14 1.12 1.11 1.10 1.09 1.08 1.08 1.07 1.06 1.05 1.04 1.03 1.03 0.84
Me
TIPSO
O
TsHN
3i
(1H-NMR, CDCl3, 500 M)
2.01 2.02 1.07 0.95 1.01 1.01 1.88 3.07 0.99 1.02 3.04 3.00 2.03 2.05 21.12 2.98
10.5 10.0 9.5 9.0 8.5 8.0 7.5 7.0 6.5 6.0 5.5 5.0 4.5 4.0 3.5 3.0 2.5 2.0 1.5 1.0 0.5 0.0 -0.5 -1
f1 (ppm)

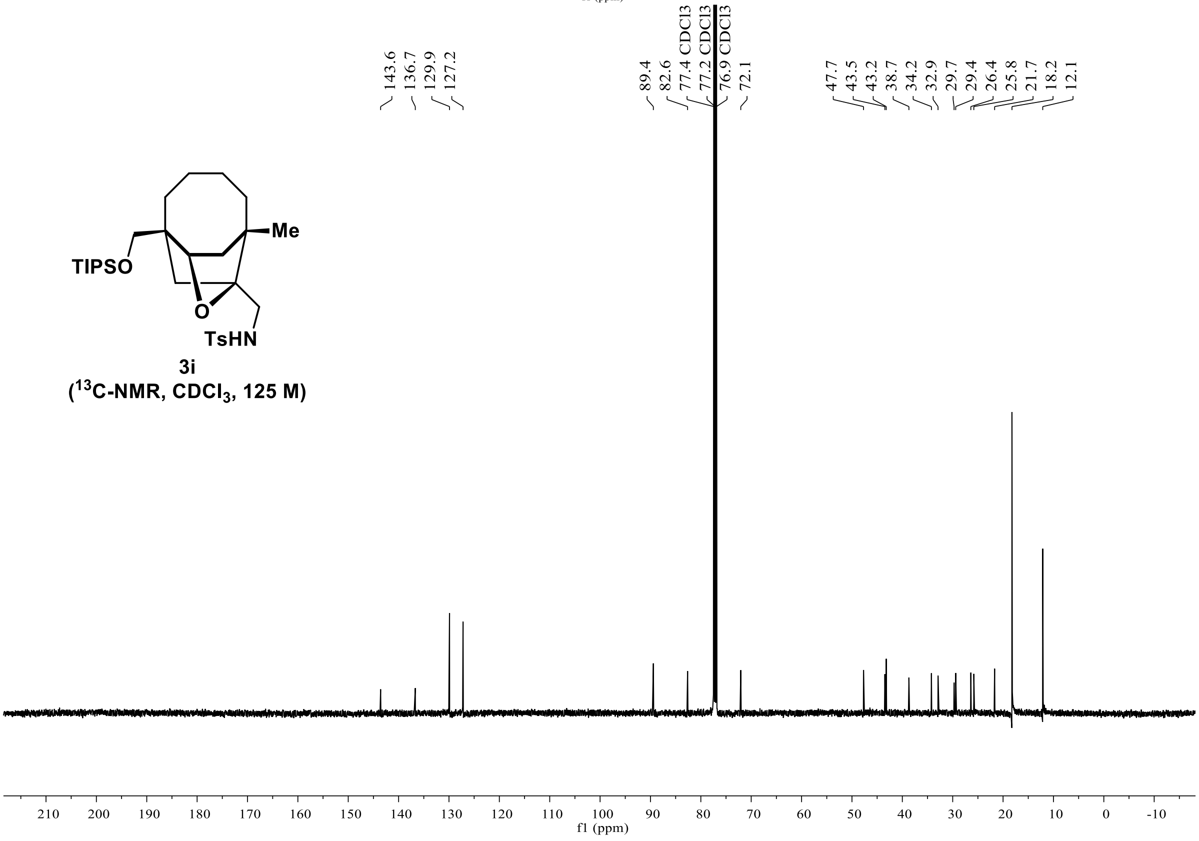
143.6 136.7 129.9 127.2 89.4 82.6 77.4 CDCl3 77.2 CDCl3 76.9 CDCl3 72.1 47.7 43.5 43.2 38.7 34.2 32.9 29.7 29.4 26.4 25.8 21.7 18.2 12.1
Me
TIPSO
O
TsHN
3i
(13C-NMR, CDCl3, 125 M)
210 200 190 180 170 160 150 140 130 120 110 100 90 80 70 60 50 40 30 20 10 0 -10
f1 (ppm)

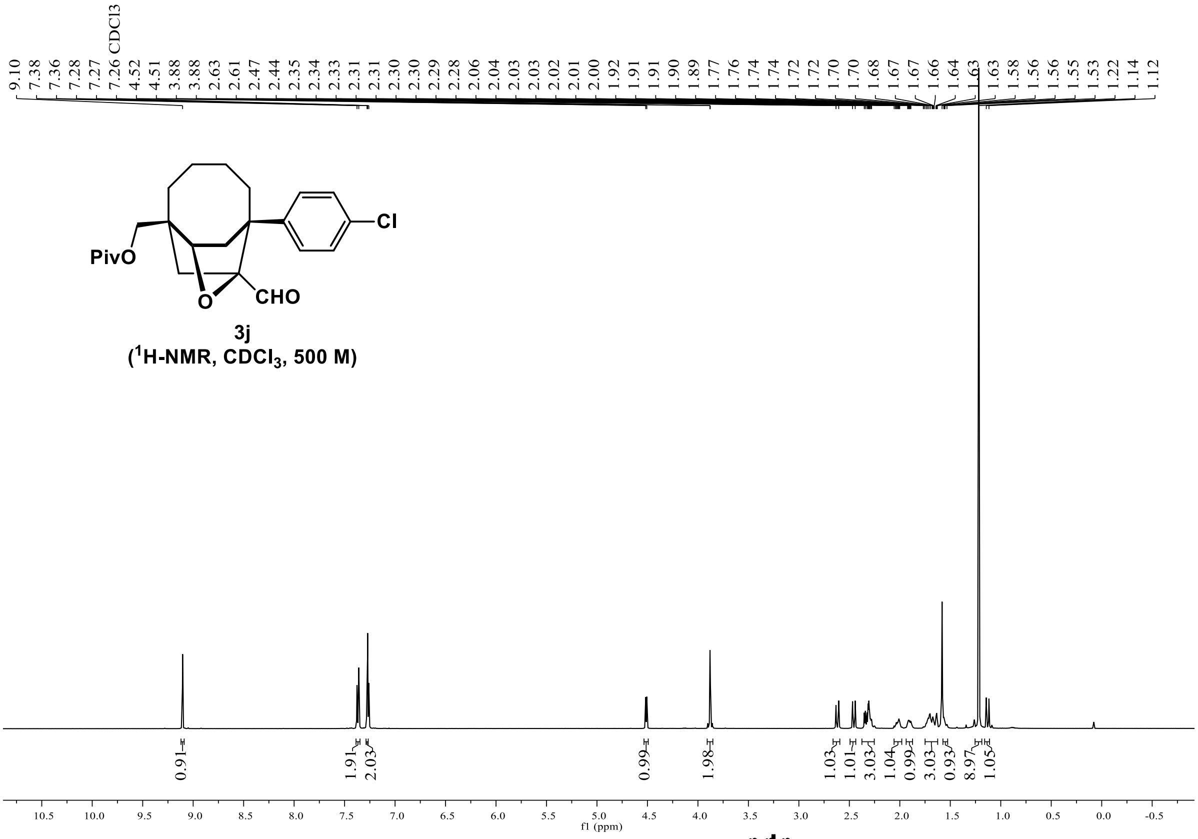
9.10
7.38
7.36
7.28
7.27
7.26 CDCl3
4.52
4.51
3.88
3.88
2.63
2.61
2.47
2.44
2.35
2.34
2.33
2.31
2.31
2.30
2.30
2.29
2.28
2.06
2.04
2.03
2.03
2.02
2.01
2.00
1.92
1.91
1.91
1.90
1.89
1.77
1.76
1.74
1.74
1.72
1.72
1.70
1.70
1.68
1.67
1.67
1.66
1.64
1.63
1.63
1.58
1.56
1.56
1.55
1.53
1.22
1.14
1.12
Cl
PivO
O
CHO
3j
(1H-NMR, CDCl3, 500 M)
0.91
1.91
2.03
0.99
1.98
1.03
1.01
3.03
1.04
0.99
3.03
0.93
8.97
1.05
f1 (ppm)

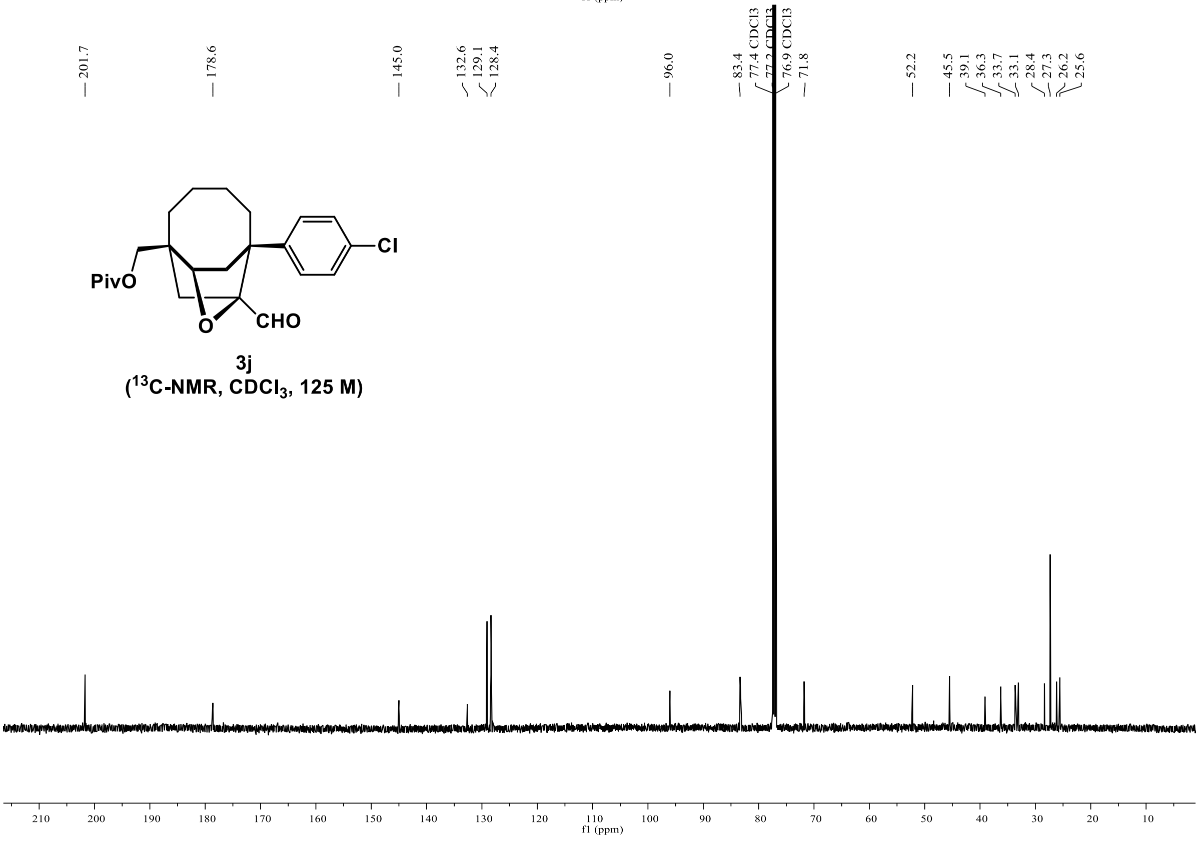
201.7
178.6
145.0
132.6
129.1
128.4
96.0
83.4
77.4 CDCl3
77.2 CDCl3
76.9 CDCl3
71.8
52.2
45.5
39.1
36.3
33.7
33.1
28.4
27.3
26.2
25.6
Cl
PivO
O
CHO
3j
(13C-NMR, CDCl3, 125 M)
f1 (ppm)

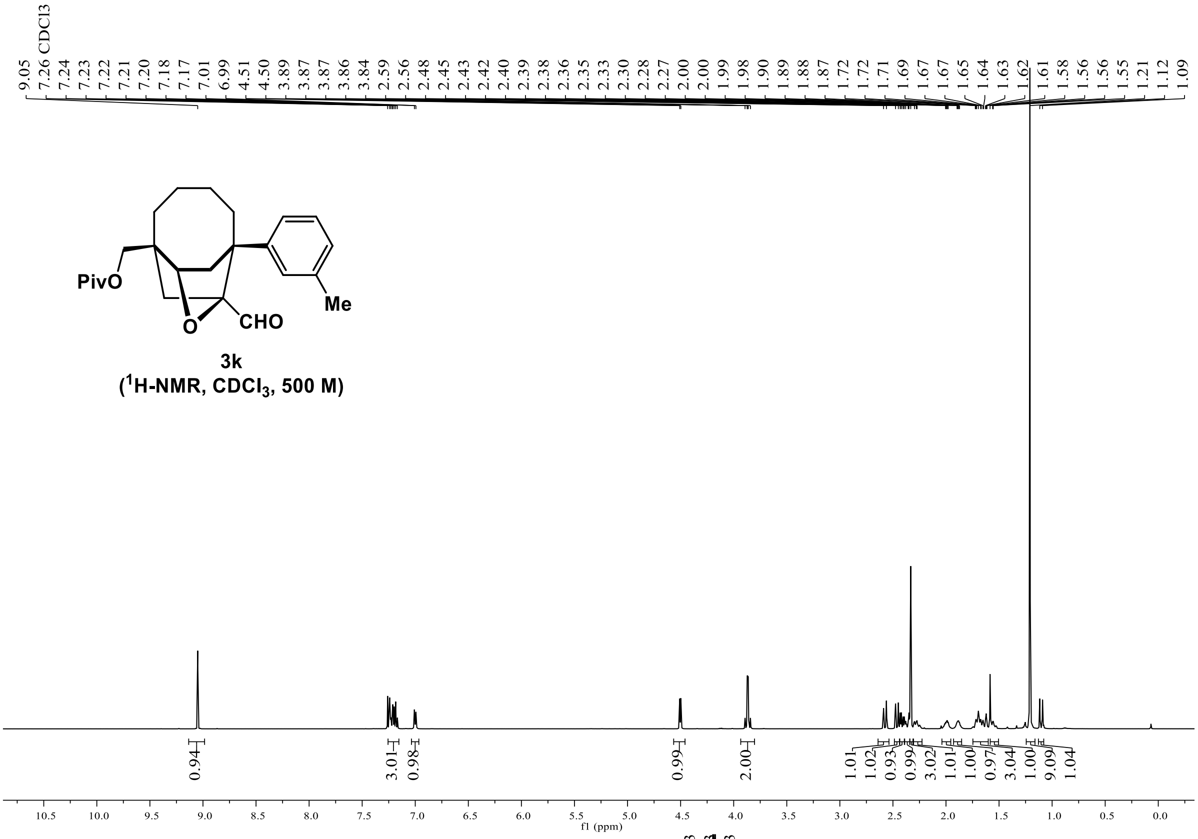
9.05 7.26 CDCl3 7.24 7.23 7.22 7.21 7.20 7.18 7.17 7.01 6.99 4.51 4.50 3.89 3.87 3.87 3.86 3.84 2.59 2.56 2.48 2.45 2.43 2.42 2.40 2.39 2.38 2.36 2.35 2.33 2.30 2.28 2.27 2.00 2.00 1.99 1.98 1.90 1.89 1.88 1.87 1.72 1.72 1.71 1.69 1.67 1.67 1.65 1.64 1.63 1.62 1.61 1.58 1.56 1.55 1.21 1.12 1.09
PivO
Me
O
CHO
3k
(1H-NMR, CDCl3, 500 M)
0.94 3.01 0.98 0.99 2.00 1.01 1.02 0.93 0.99 3.02 1.01 1.00 0.97 3.04 1.00 9.09 1.04
f1 (ppm)

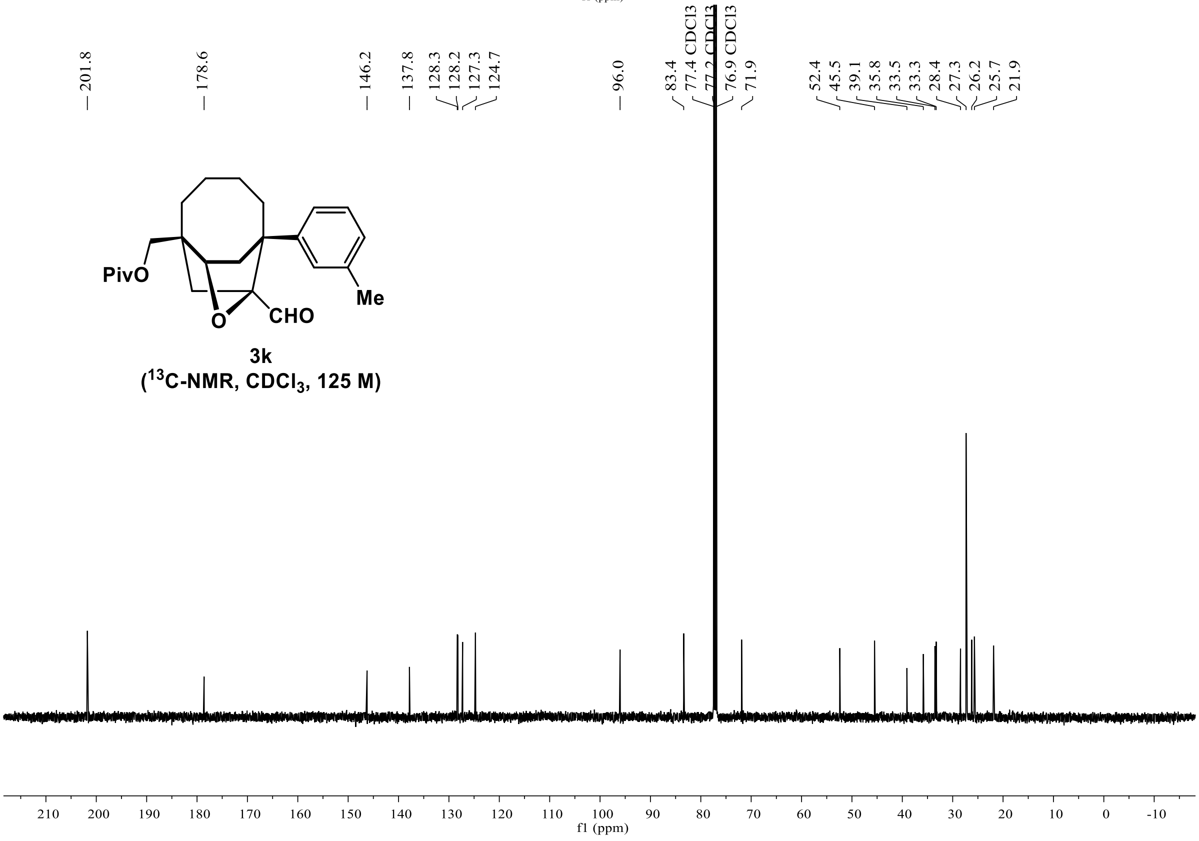
201.8 178.6 146.2 137.8 128.3 128.2 127.3 124.7 96.0 83.4 77.4 CDCl3 77.2 CDCl3 76.9 CDCl3 71.9 52.4 45.5 39.1 35.8 33.5 33.3 28.4 27.3 26.2 25.7 21.9
PivO
Me
O
CHO
3k
(13C-NMR, CDCl3, 125 M)
f1 (ppm)

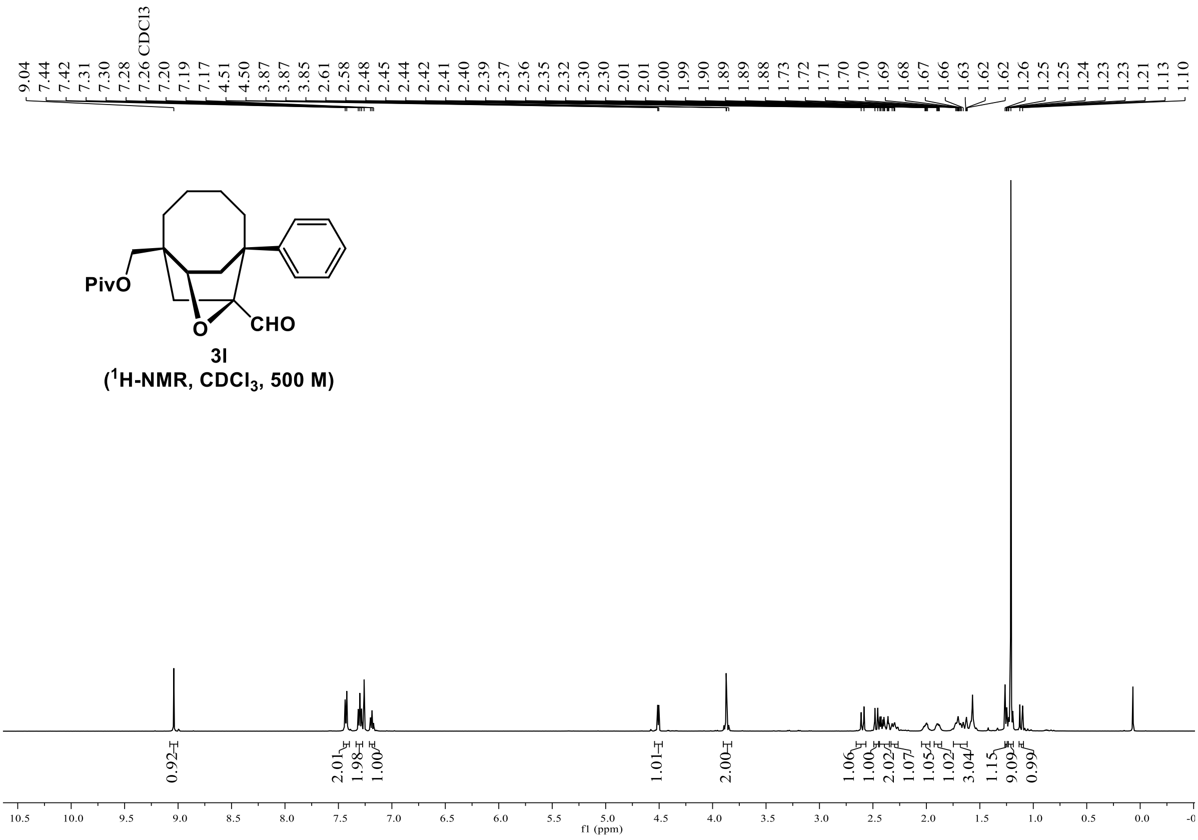
PivO
O
CHO
3l
(1H-NMR, CDCl3, 500 M)

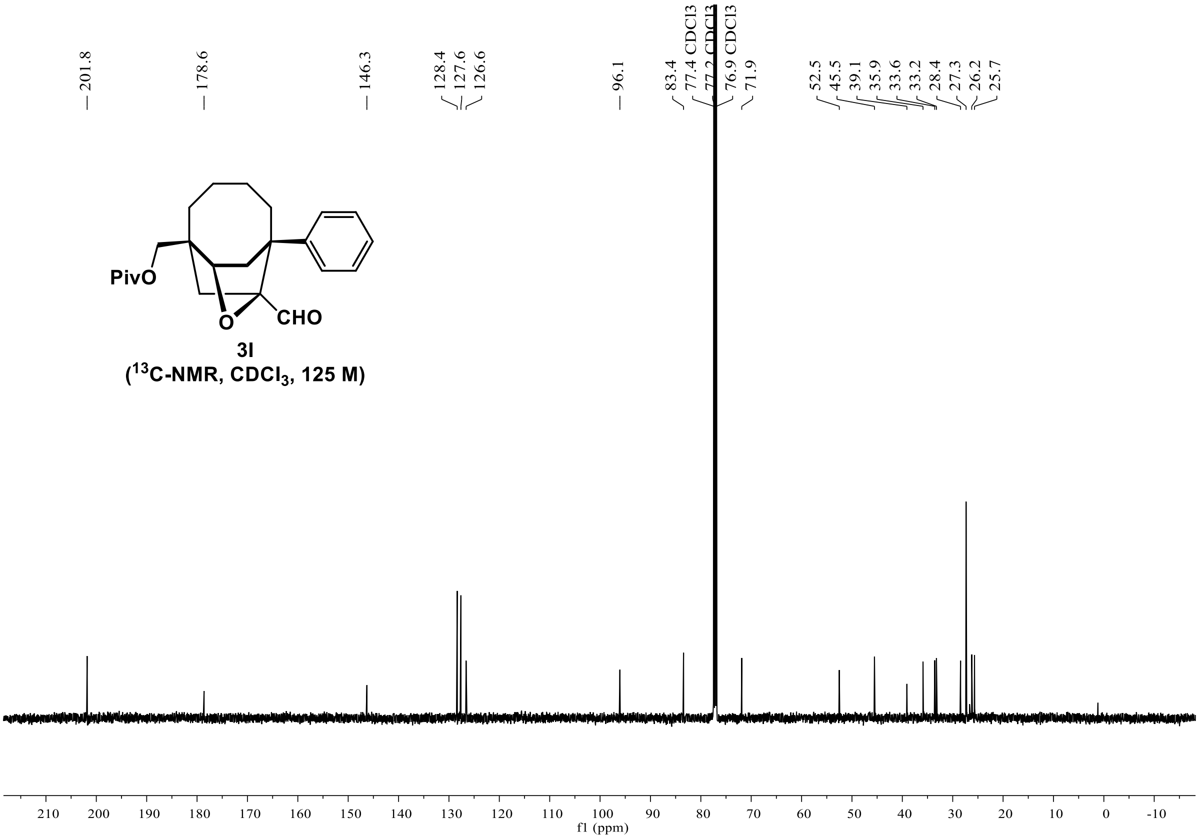
PivO
O
CHO
3l
(13C-NMR, CDCl3, 125 M)

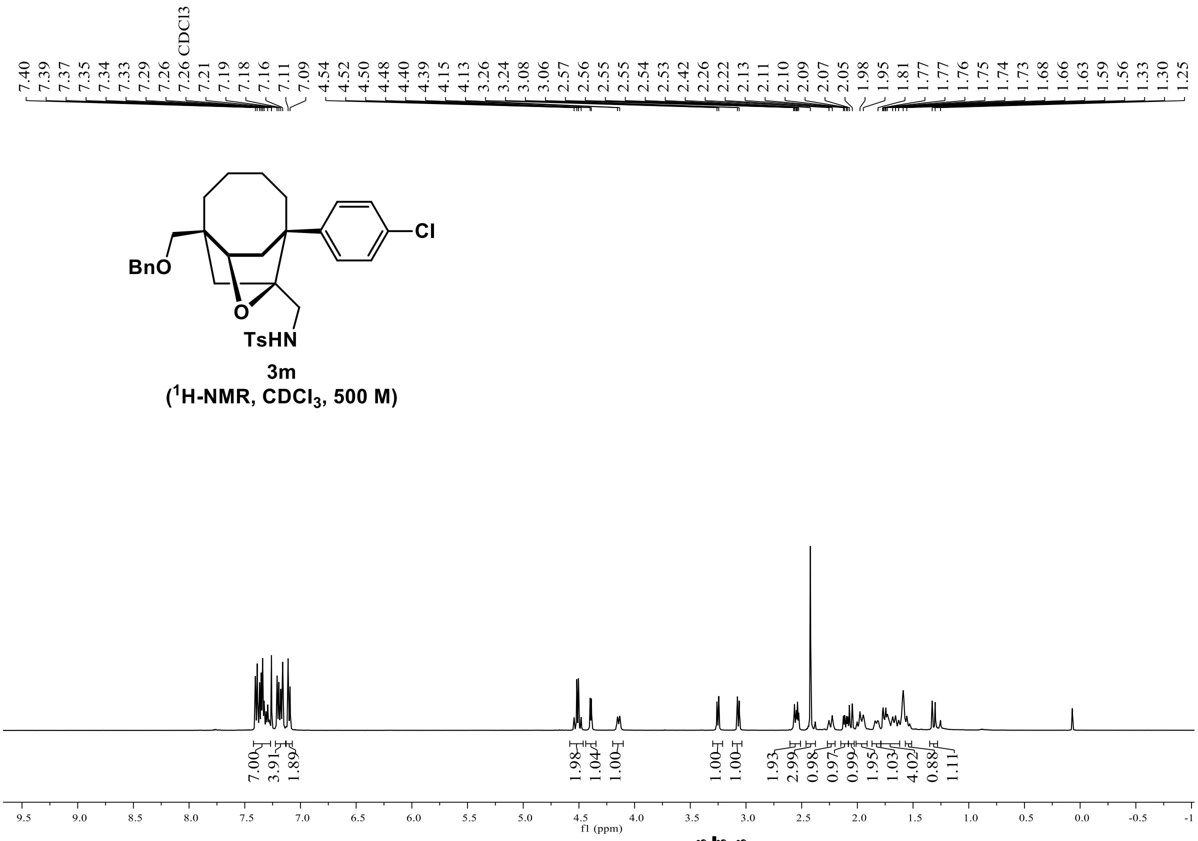

3m
(^{1}H-NMR, $CDCl_3$, 500 M)

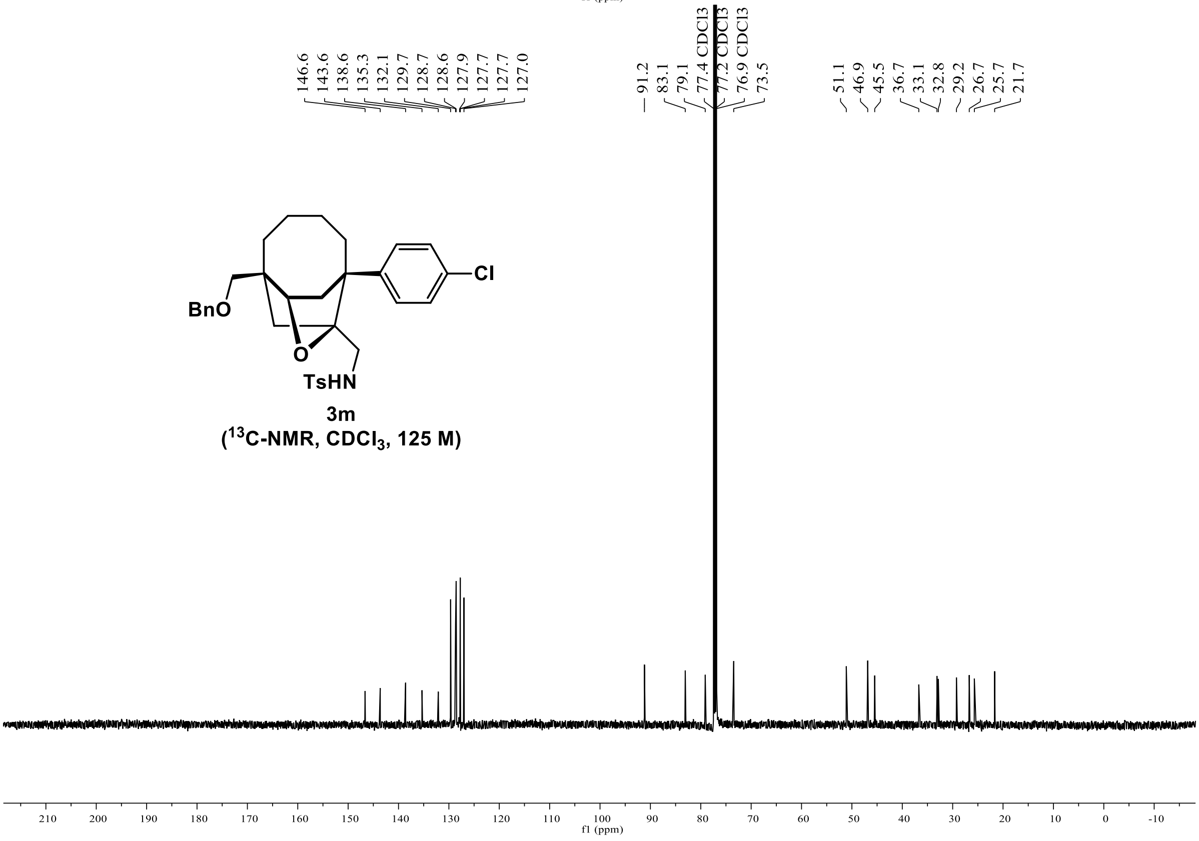

3m
(^{13}C-NMR, $CDCl_3$, 125 M)

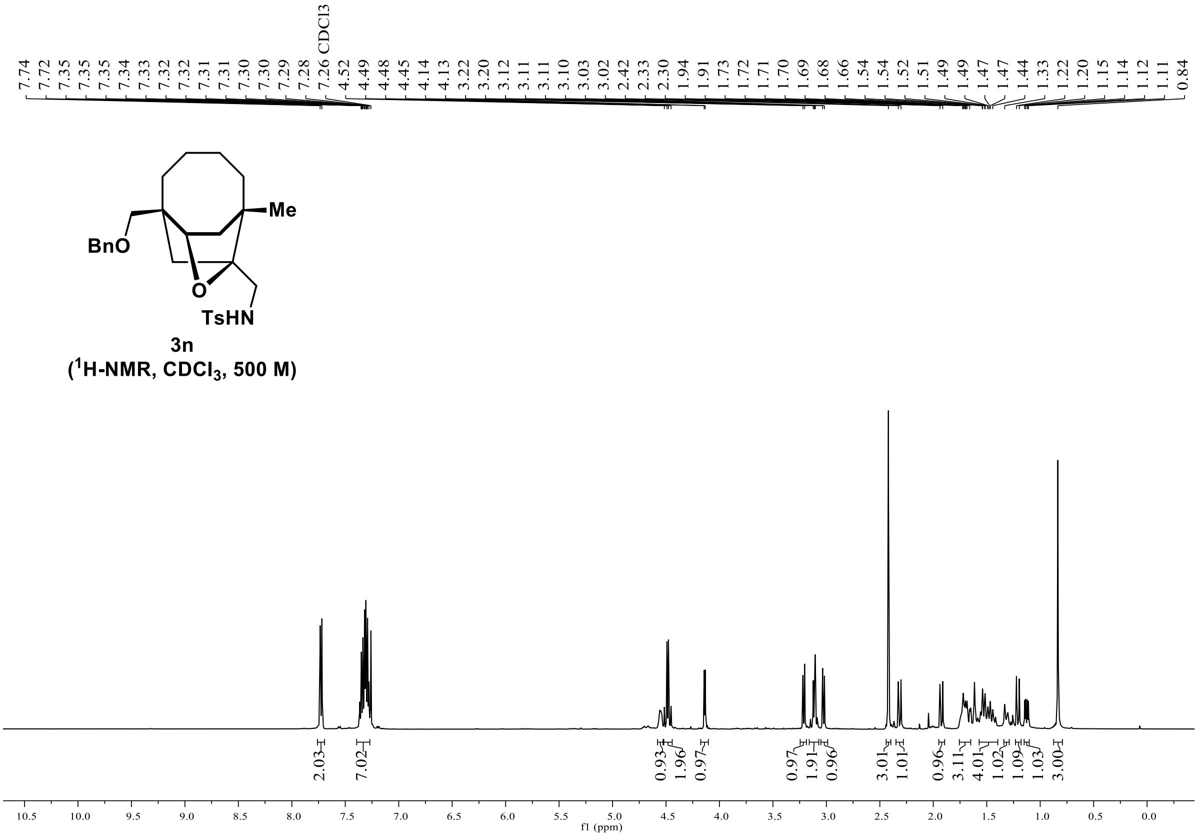
7.74 7.72 7.35 7.35 7.34 7.33 7.32 7.32 7.31 7.31 7.30 7.30 7.29 7.28 7.26 CDCl3 4.52 4.49 4.48 4.45 4.14 4.13 3.22 3.20 3.12 3.11 3.11 3.10 3.03 3.02 2.42 2.33 2.30 1.94 1.91 1.73 1.72 1.71 1.70 1.69 1.68 1.66 1.54 1.54 1.52 1.51 1.49 1.49 1.47 1.47 1.44 1.33 1.22 1.20 1.15 1.14 1.12 1.11 0.84
BnO
Me
O
TsHN
3n
(1H-NMR, CDCl3, 500 M)
2.03 7.02 0.93 1.96 0.97 0.97 1.91 0.96 3.01 1.01 0.96 3.11 4.01 1.02 1.09 1.03 3.00
f1 (ppm)

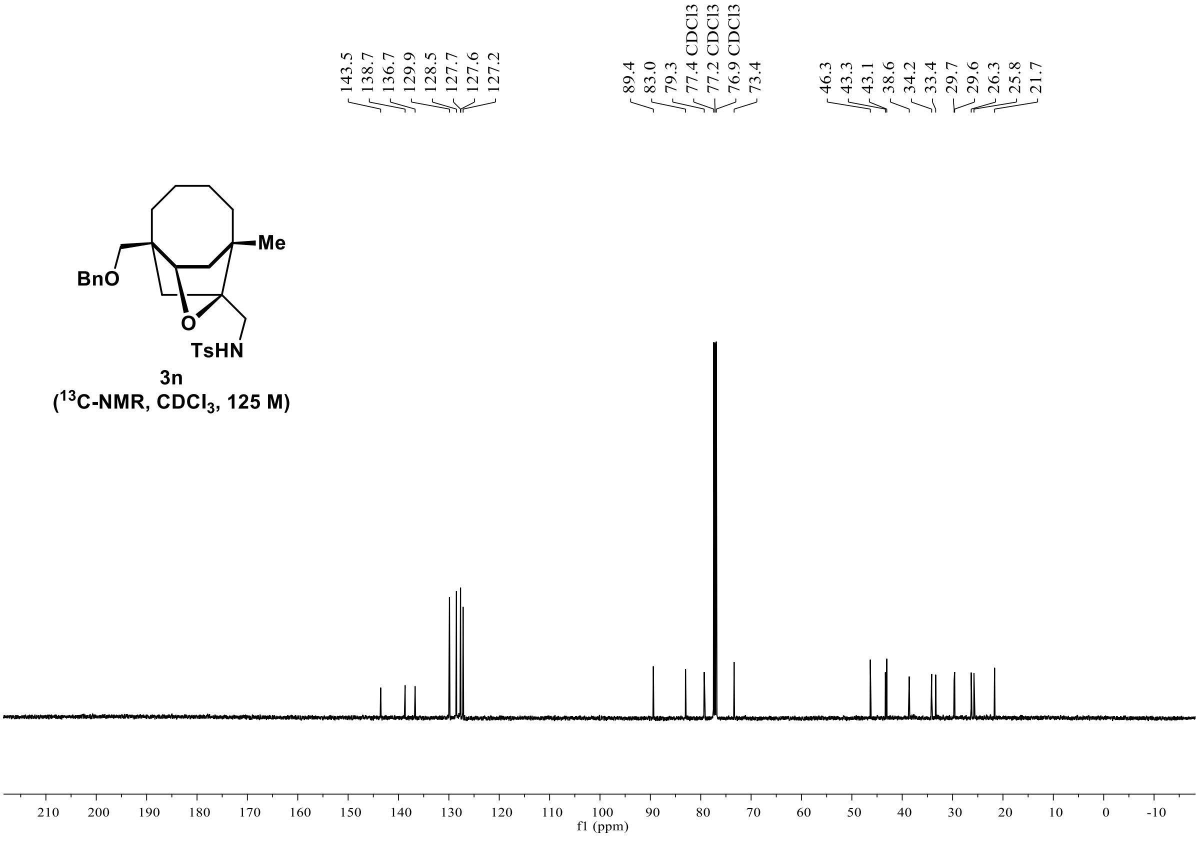
143.5 138.7 136.7 129.9 128.5 127.7 127.6 127.2
89.4 83.0 79.3 77.4 CDCl3 77.2 CDCl3 76.9 CDCl3 73.4
46.3 43.3 43.1 38.6 34.2 33.4 29.7 29.6 26.3 25.8 21.7
BnO
Me
O
TsHN
3n
(13C-NMR, CDCl3, 125 M)
f1 (ppm)

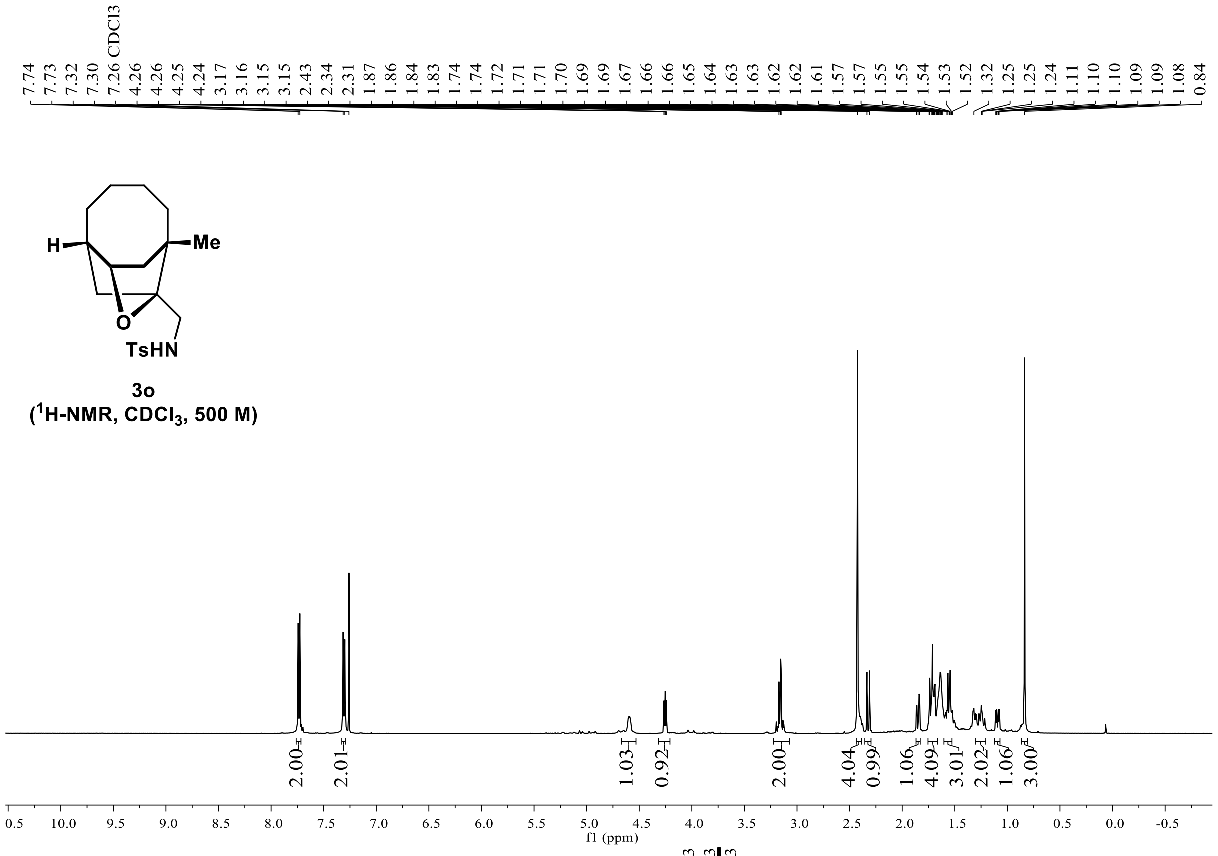

3o
(^{1}H-NMR, $CDCl_3$, 500 M)

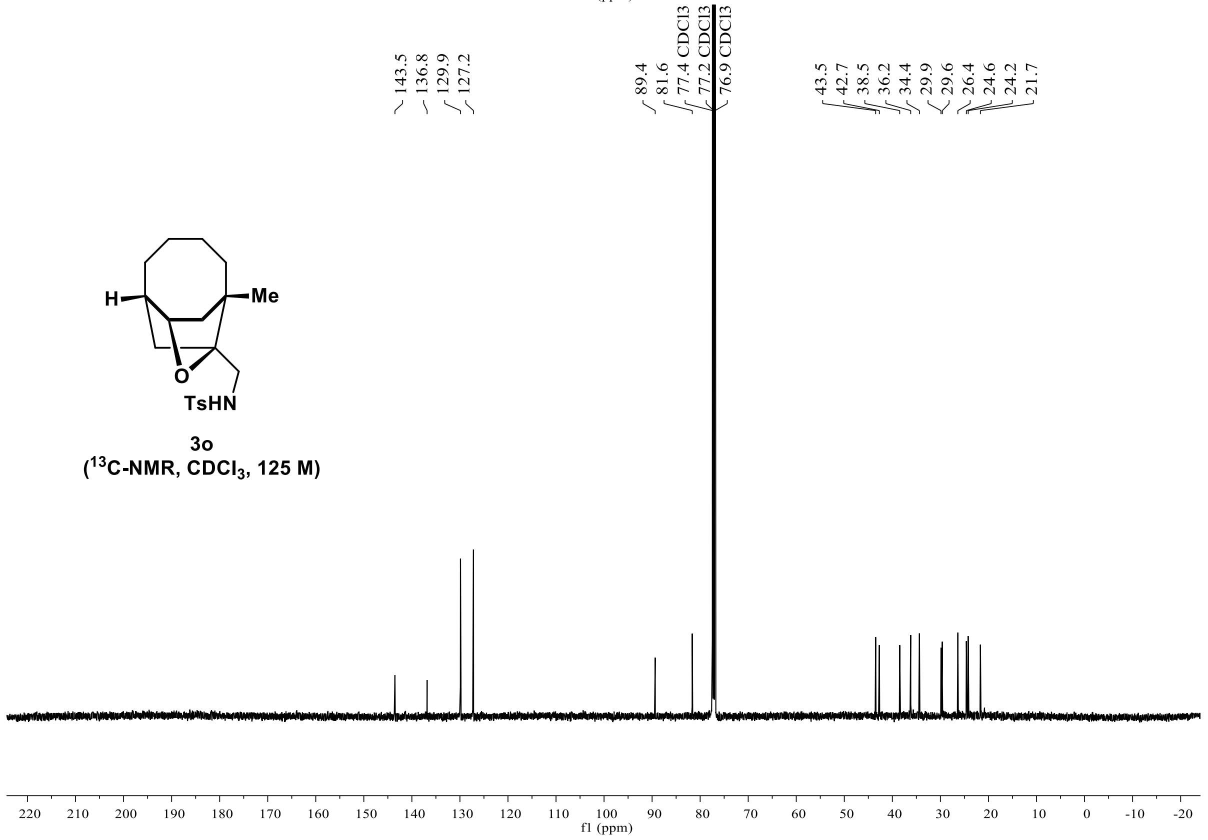

3o
(^{13}C-NMR, $CDCl_3$, 125 M)

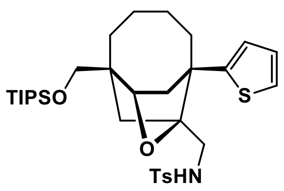

3p
(^{1}H-NMR, CDCl$_3$, 500 M)

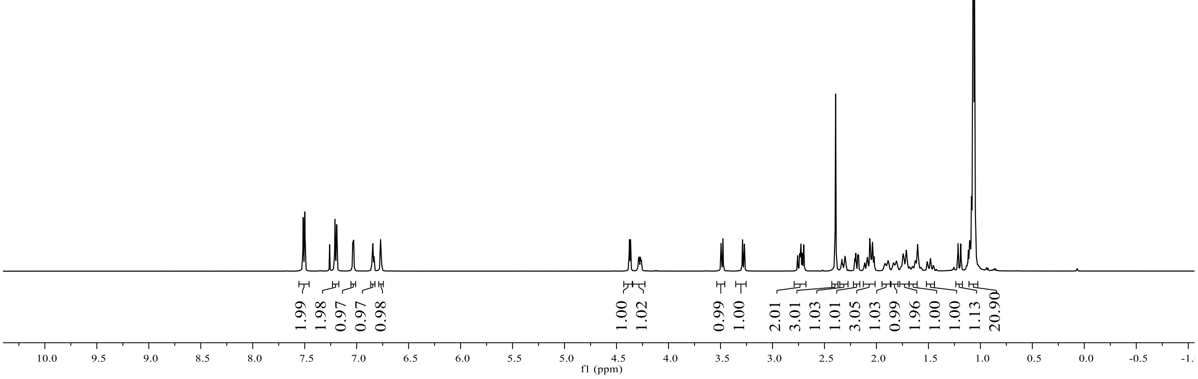

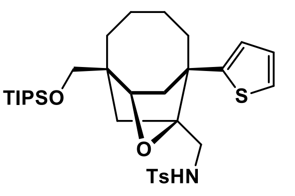

3p
(^{13}C-NMR, CDCl$_3$, 125 M)

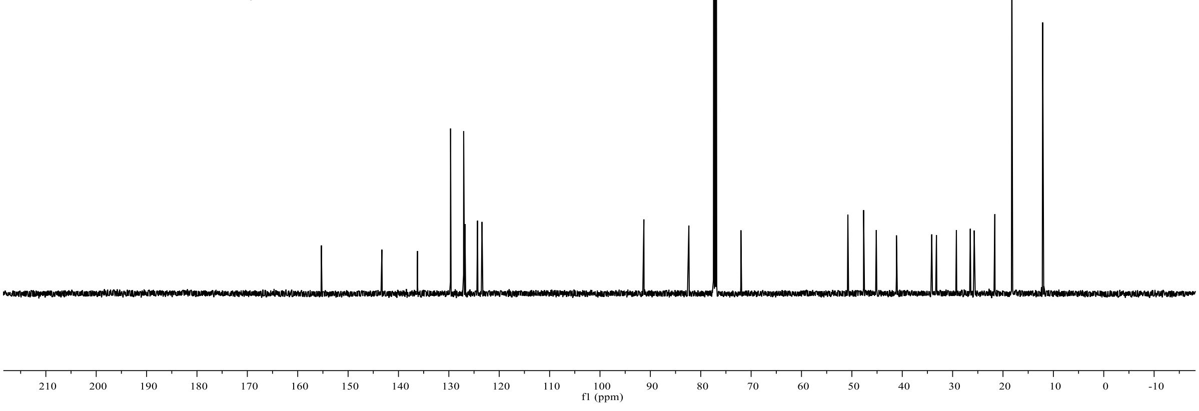

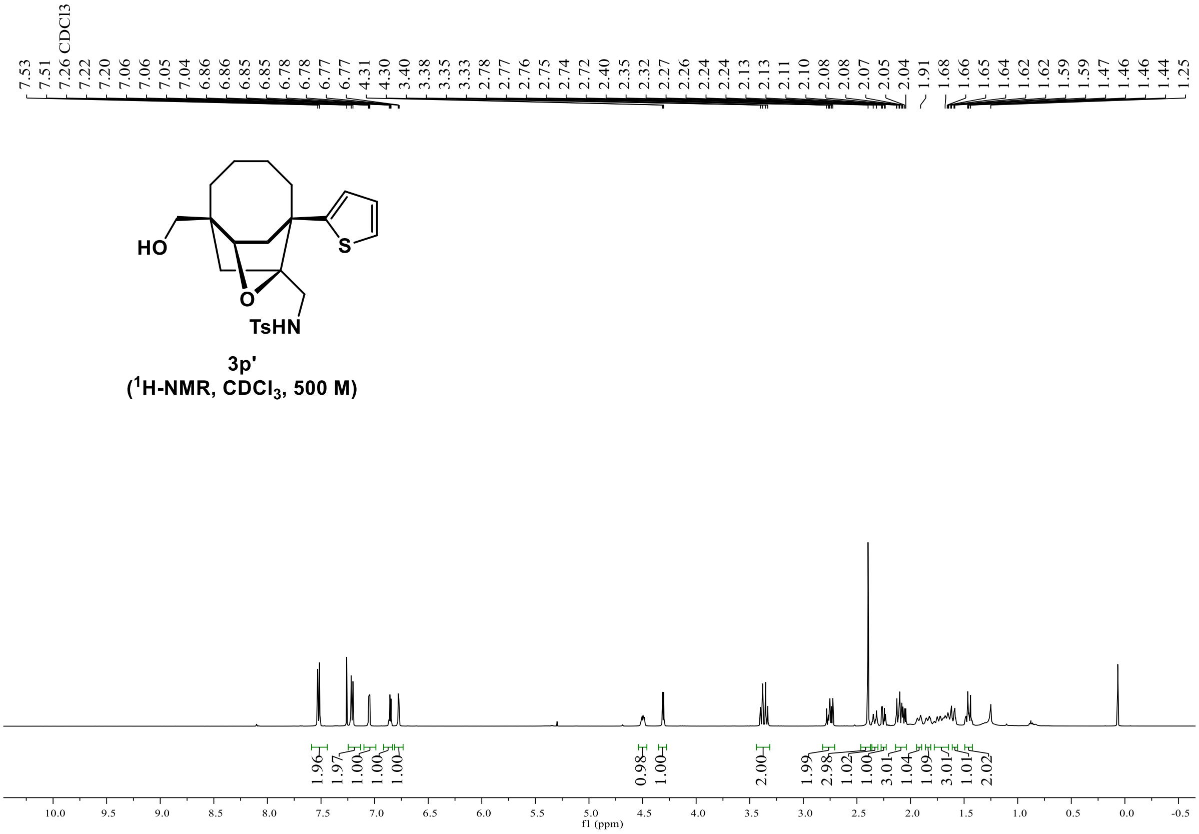

3p'
(^{1}H-NMR, $CDCl_3$, 500 M)

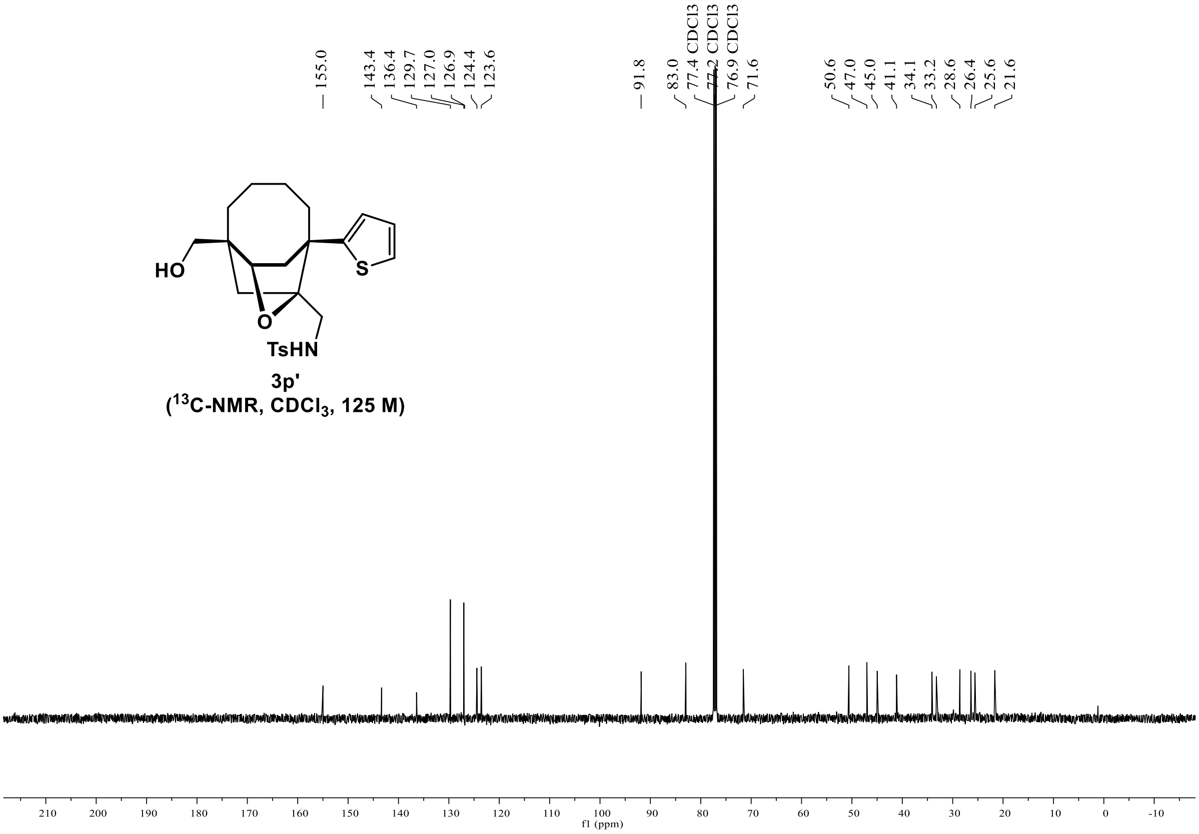

3p'
(^{13}C-NMR, $CDCl_3$, 125 M)

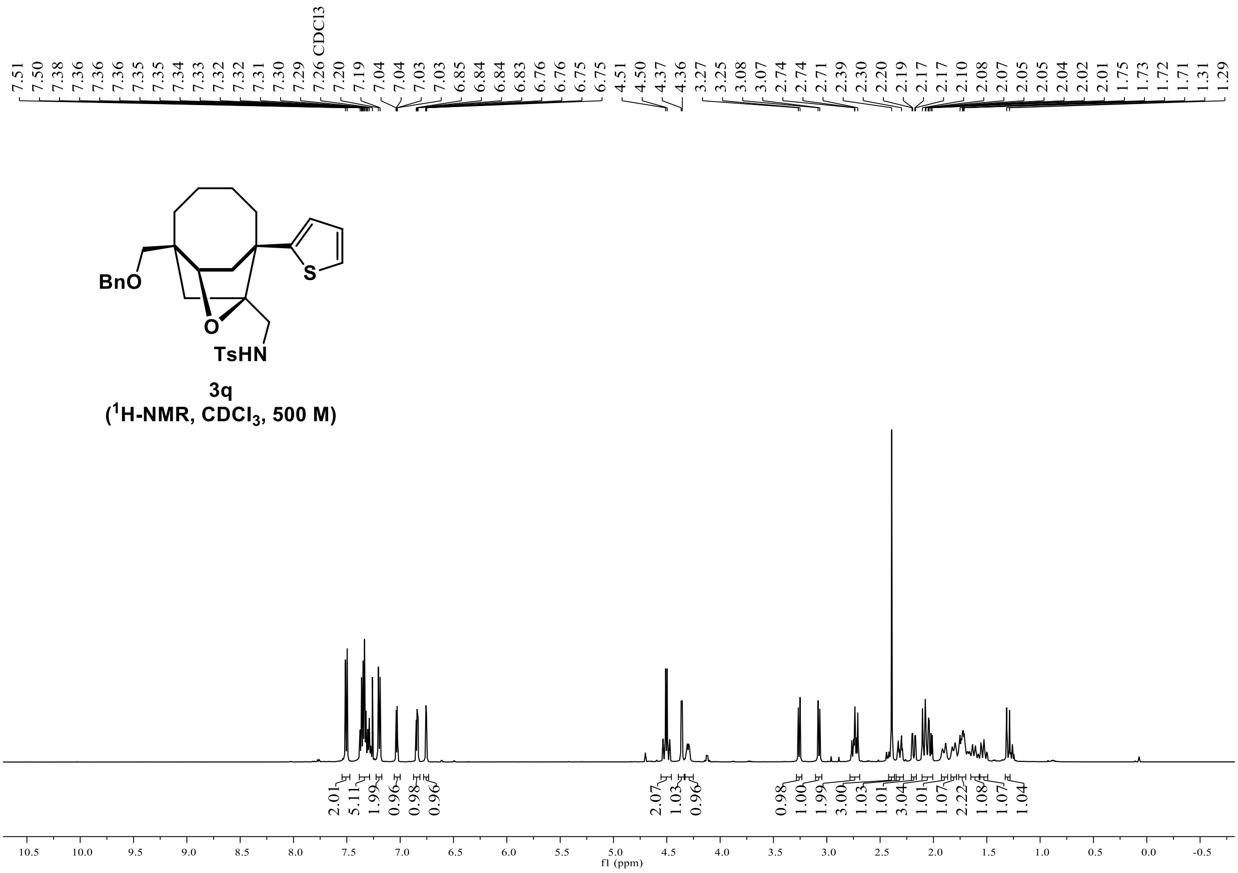

3q
(^{1}H-NMR, CDCl$_3$, 500 M)

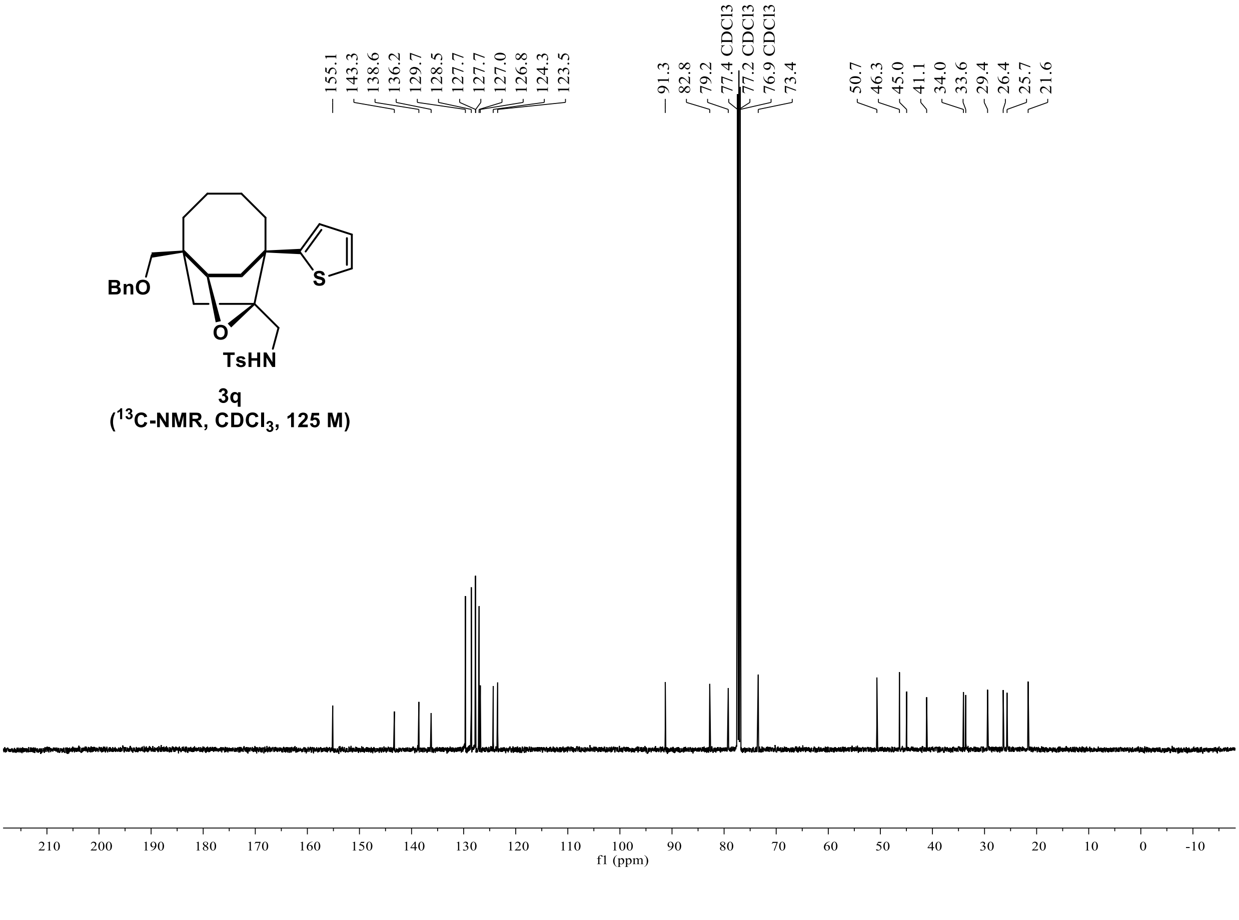

3q
(^{13}C-NMR, CDCl$_3$, 125 M)

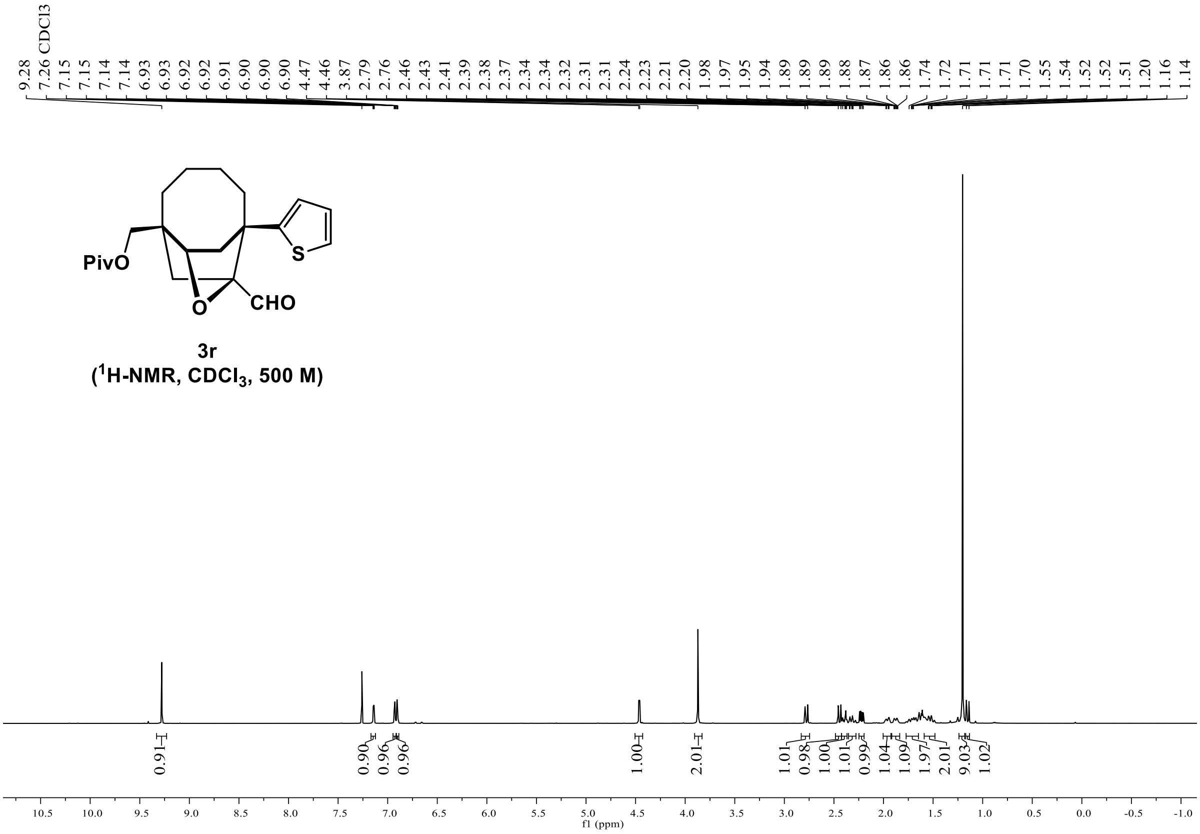

3r
(^{1}H-NMR, $CDCl_3$, 500 M)

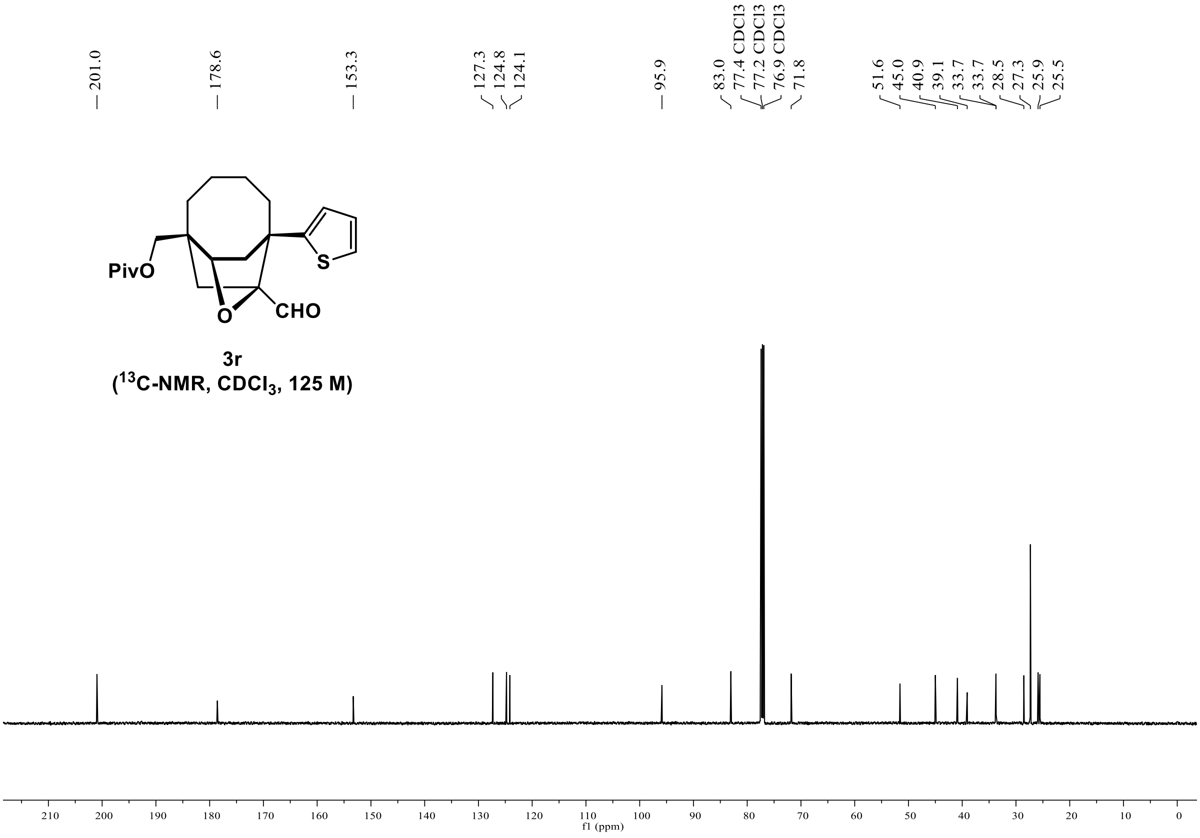

3r
(^{13}C-NMR, $CDCl_3$, 125 M)

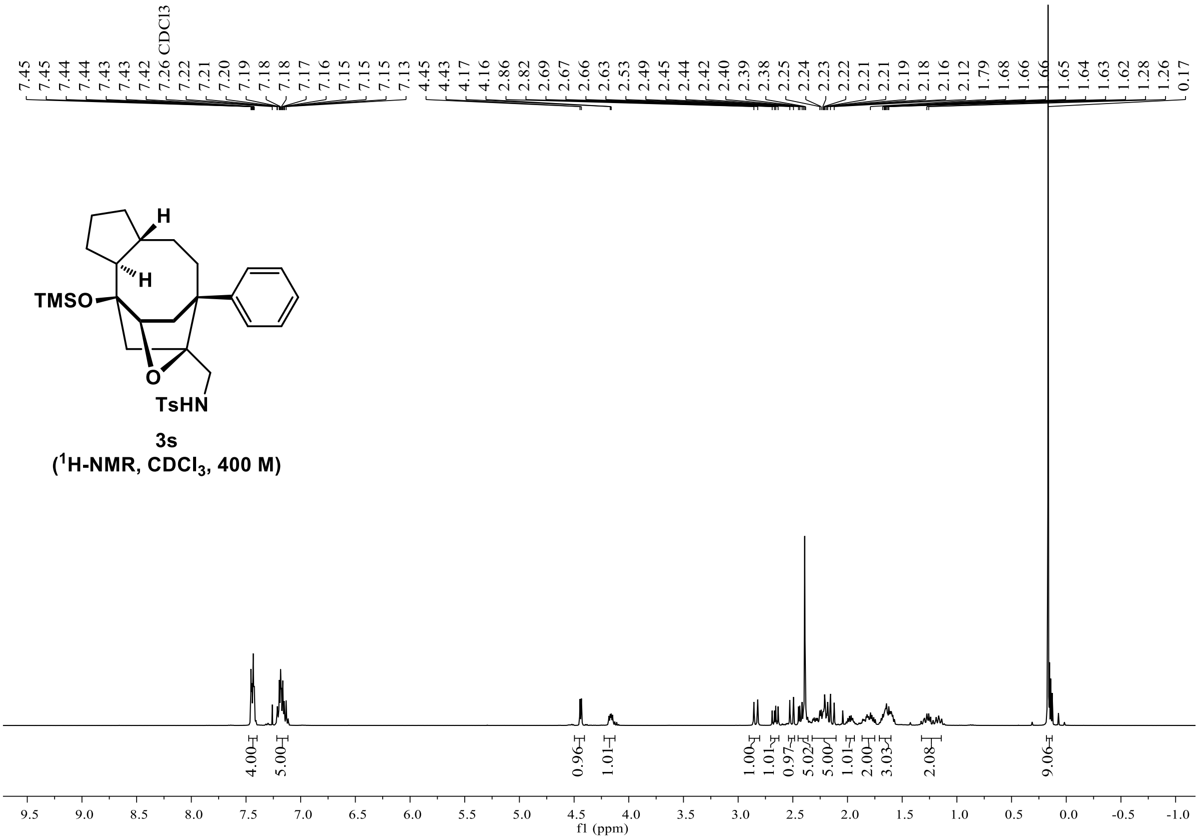

3s
(^{1}H-NMR, $CDCl_3$, 400 M)

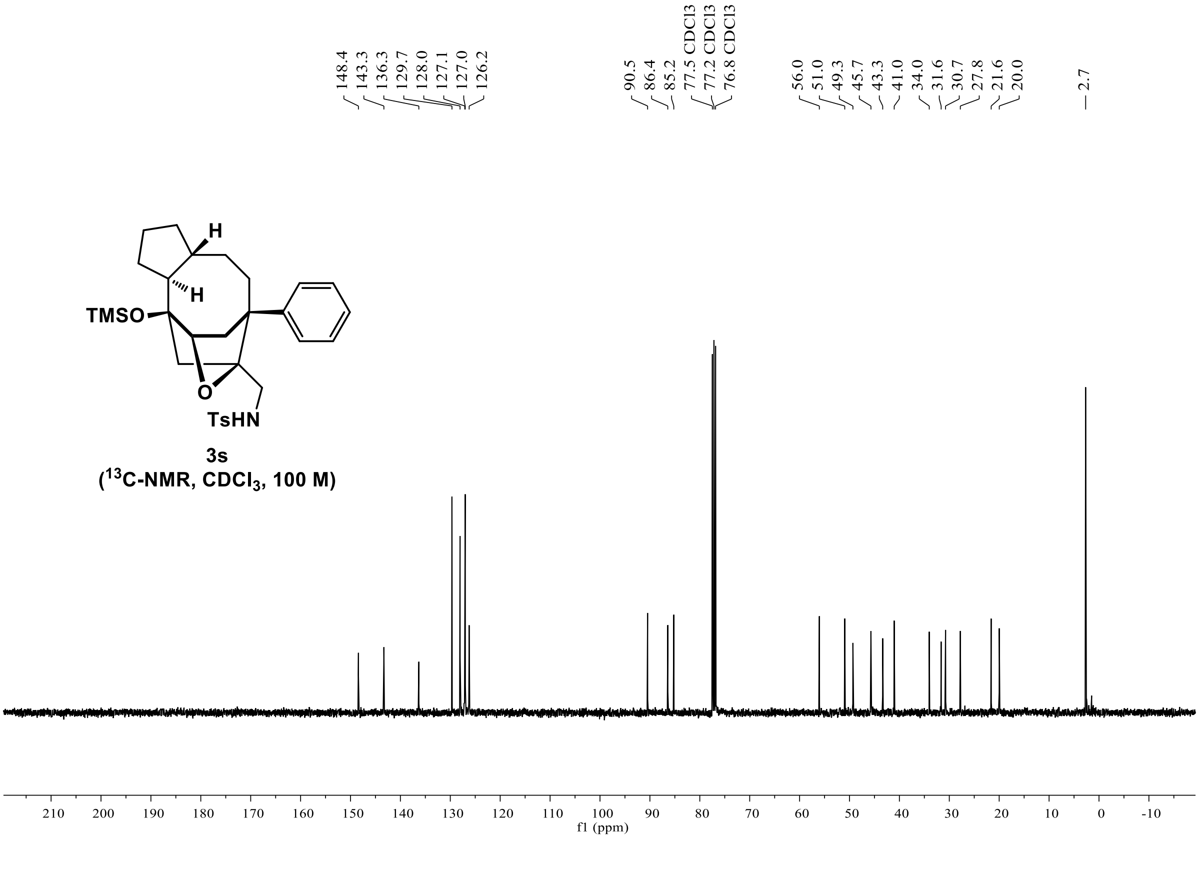

3s
(^{13}C-NMR, $CDCl_3$, 100 M)

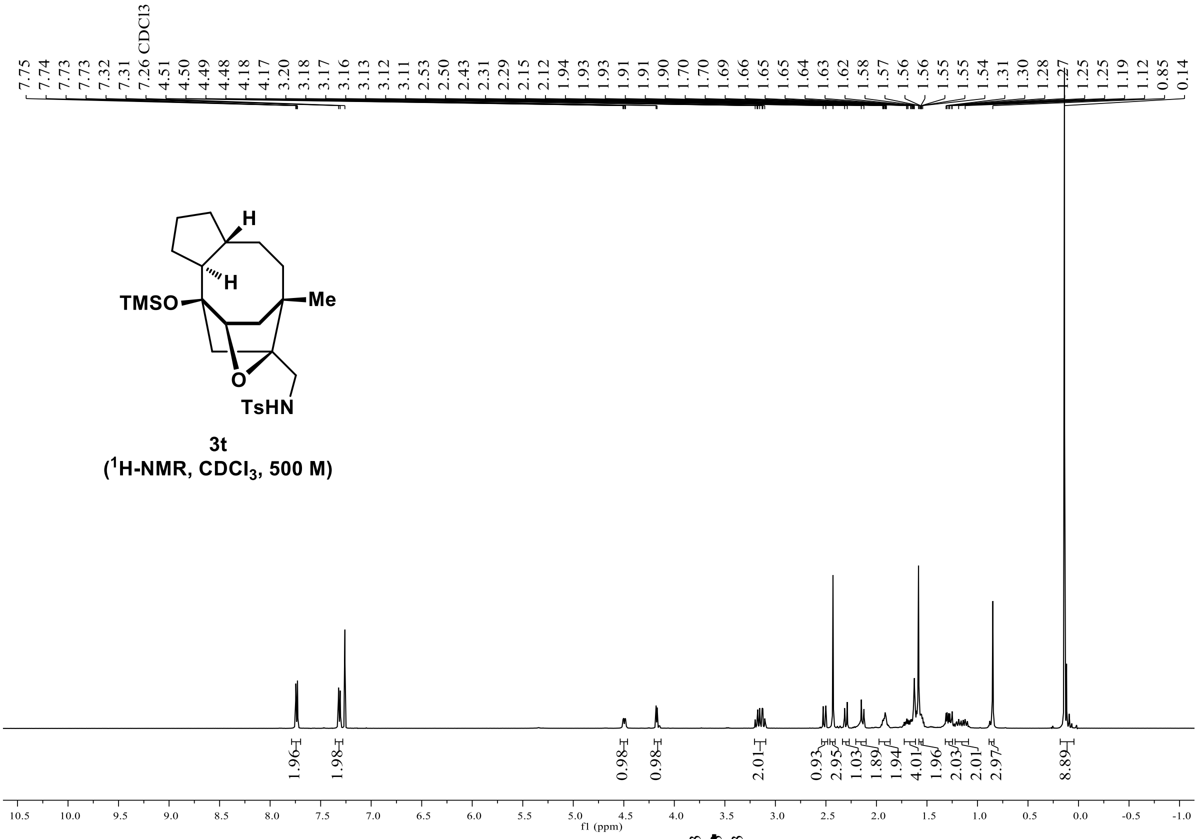

3t
(^{1}H-NMR, CDCl$_3$, 500 M)

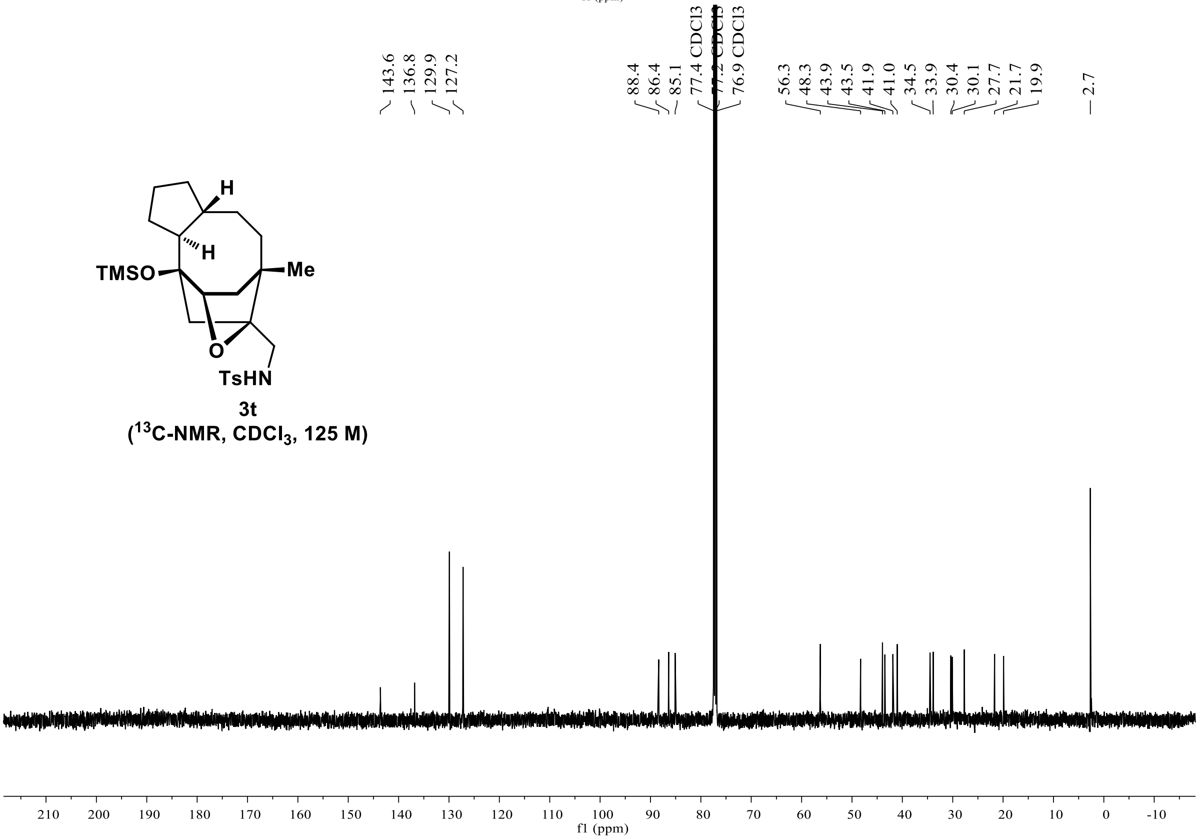

3t
(^{13}C-NMR, CDCl$_3$, 125 M)

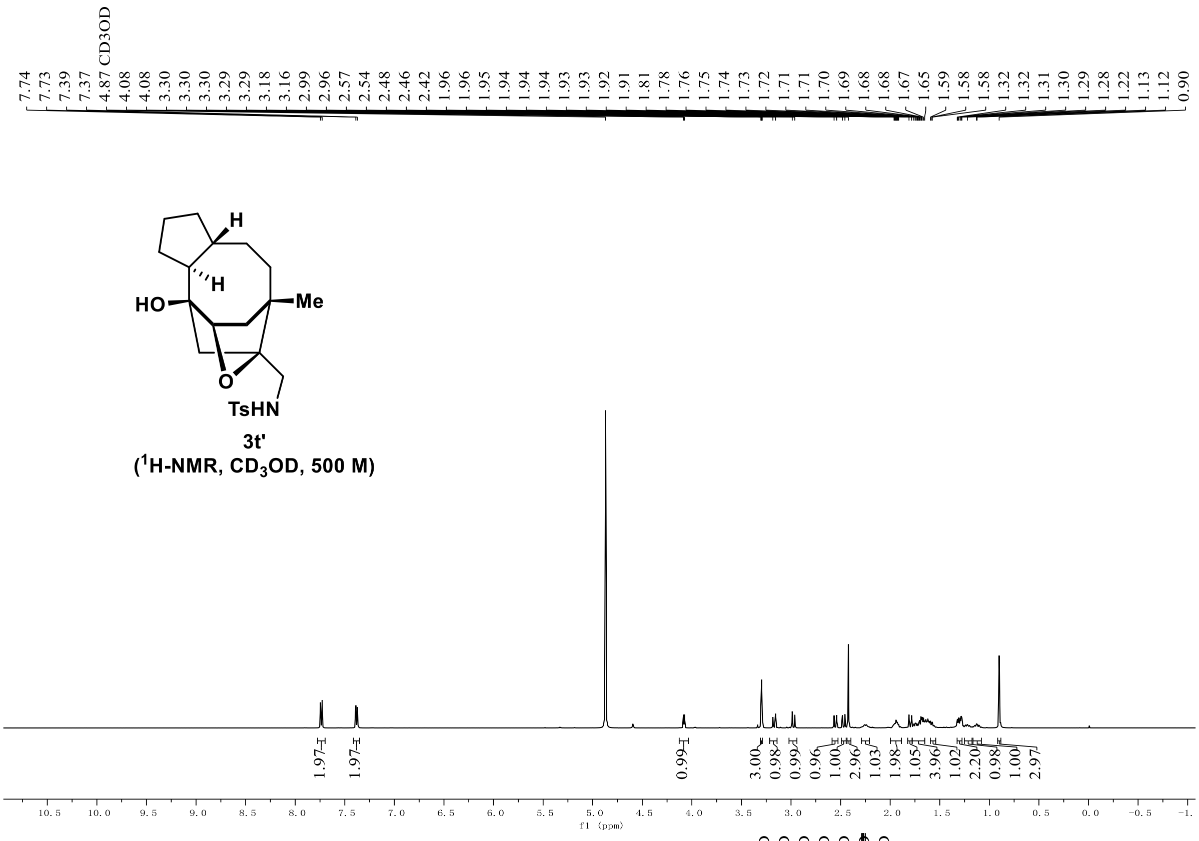

3t'
(^{1}H-NMR, CD_3OD, 500 M)

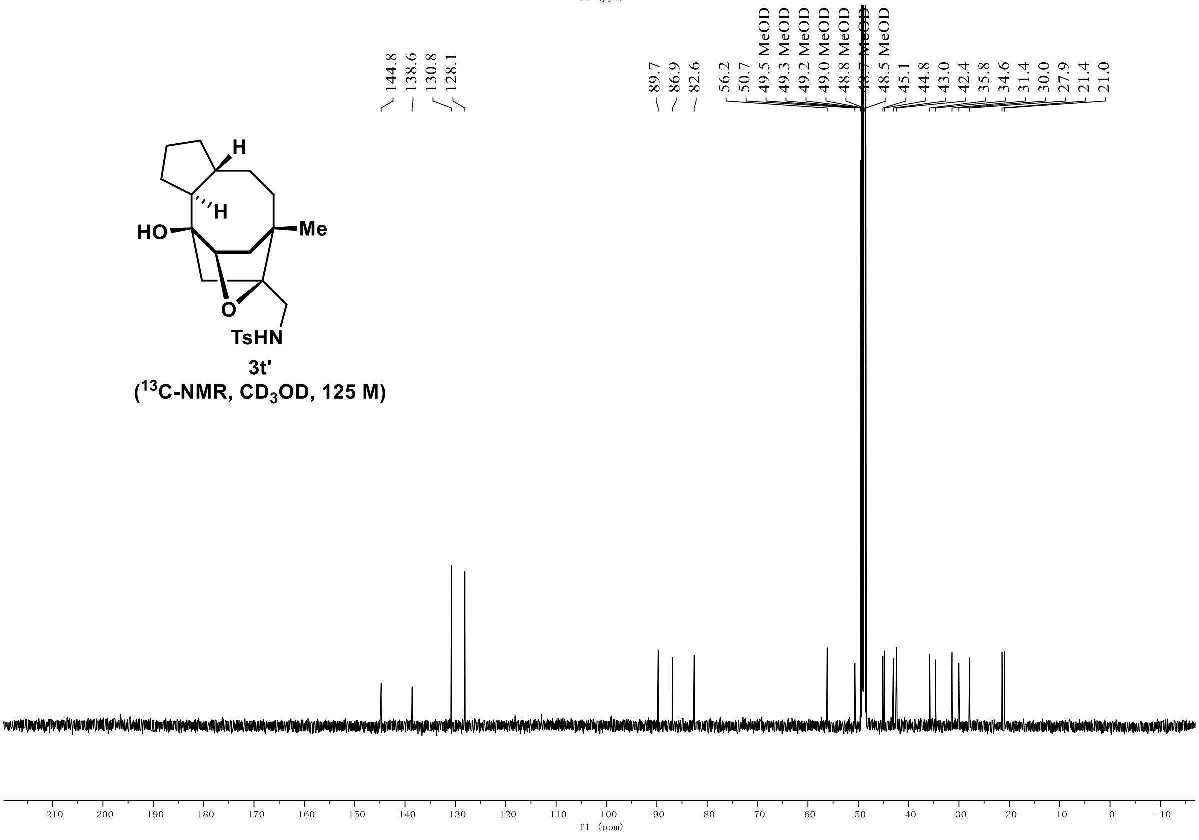

3t'
(^{13}C-NMR, CD_3OD, 125 M)

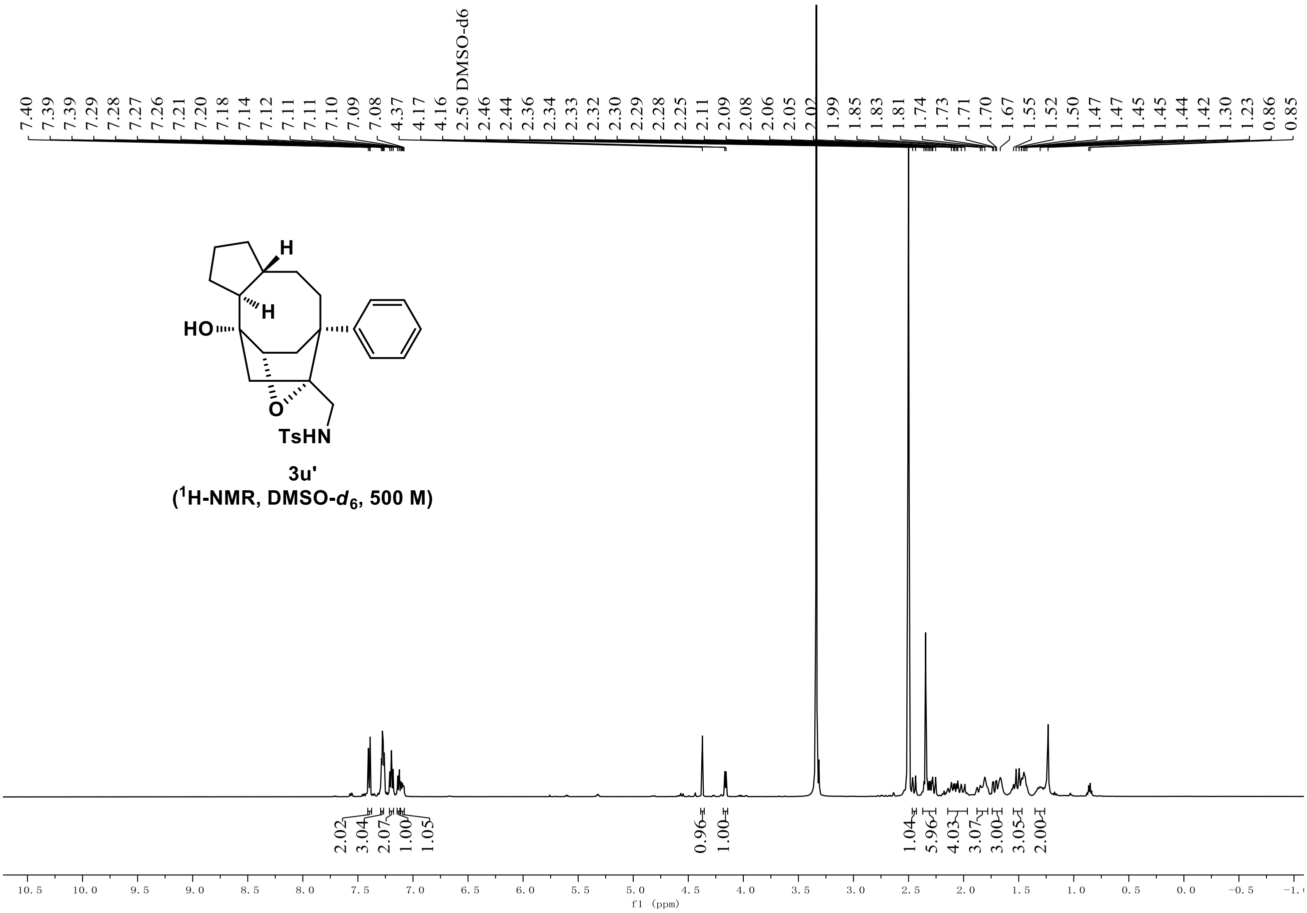

3u'
(^{1}H-NMR, DMSO-d_6, 500 M)

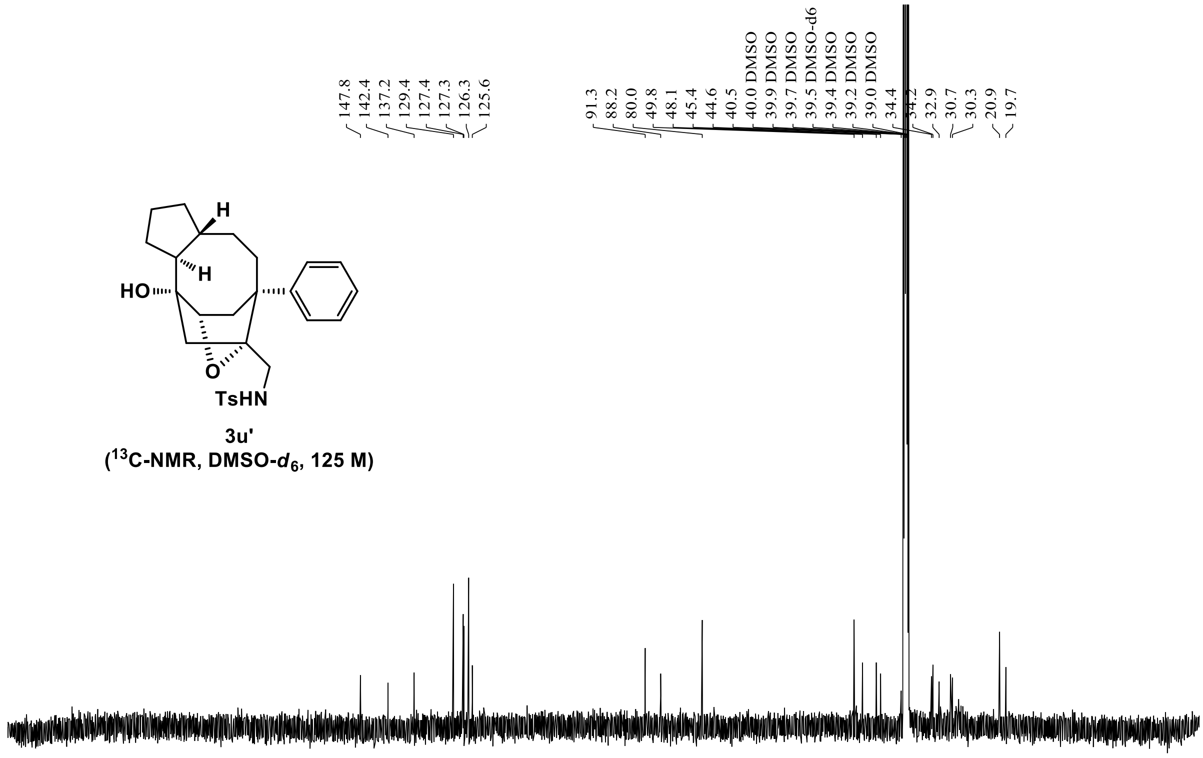

3u'
(^{13}C-NMR, DMSO-d_6, 125 M)

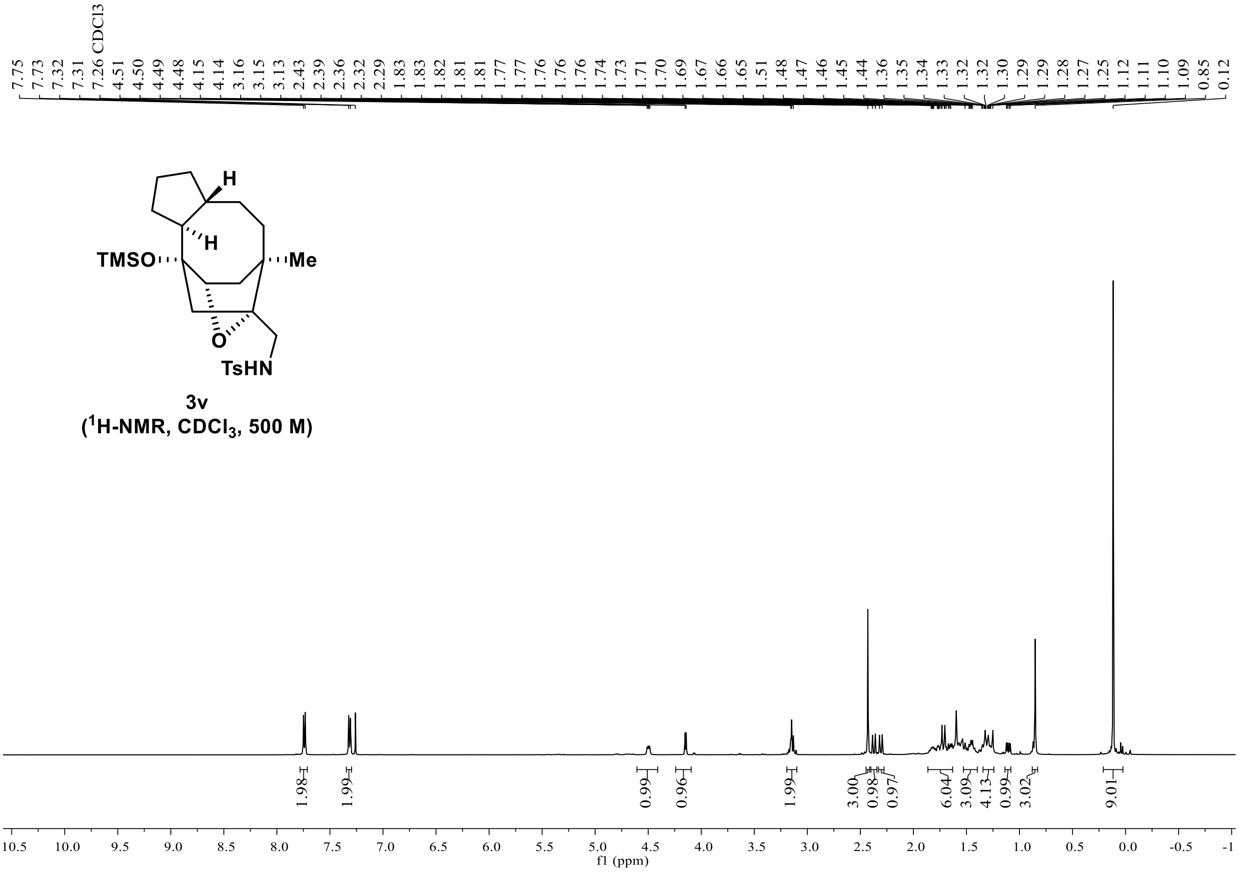
7.75 7.73 7.32 7.31 7.26 CDCl3 4.51 4.50 4.49 4.48 4.15 4.14 3.16 3.15 3.13 2.43 2.39 2.36 2.32 2.29 1.83 1.83 1.82 1.81 1.81 1.77 1.77 1.76 1.76 1.76 1.74 1.73 1.71 1.70 1.69 1.67 1.66 1.65 1.51 1.48 1.47 1.46 1.45 1.44 1.36 1.35 1.34 1.33 1.32 1.32 1.30 1.29 1.29 1.28 1.27 1.25 1.12 1.11 1.10 1.09 0.85 0.12
H
H
TMSO
Me
O
TsHN
3v
(1H-NMR, CDCl3, 500 M)
1.98 1.99 0.99 0.96 1.99 3.00 0.98 0.97 6.04 3.09 4.13 0.99 3.02 9.01
10.5 10.0 9.5 9.0 8.5 8.0 7.5 7.0 6.5 6.0 5.5 5.0 4.5 4.0 3.5 3.0 2.5 2.0 1.5 1.0 0.5 0.0 -0.5 -1
f1 (ppm)

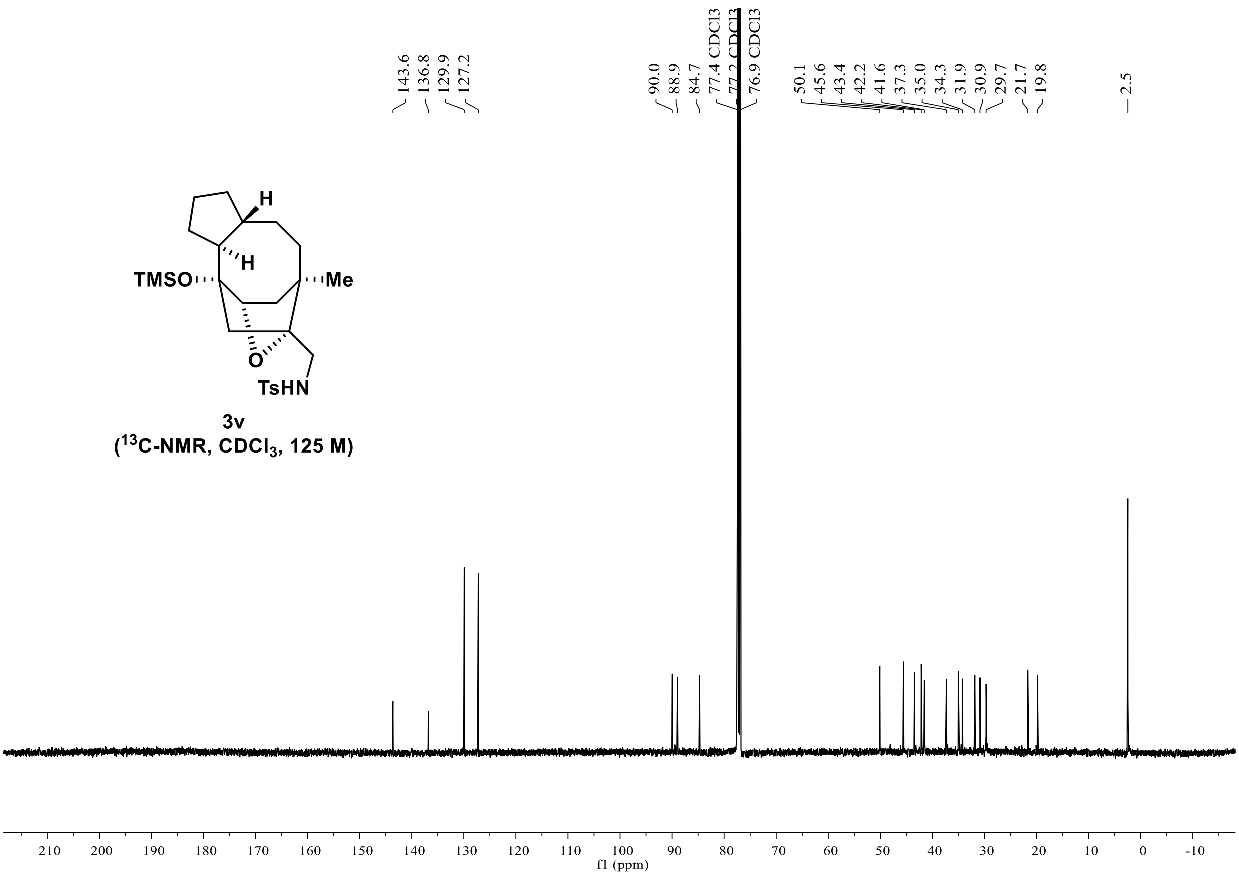
143.6 136.8 129.9 127.2 90.0 88.9 84.7 77.4 CDCl3 77.2 CDCl3 76.9 CDCl3 50.1 45.6 43.4 42.2 41.6 37.3 35.0 34.3 31.9 30.9 29.7 21.7 19.8 2.5
H
H
TMSO
Me
O
TsHN
3v
(13C-NMR, CDCl3, 125 M)
210 200 190 180 170 160 150 140 130 120 110 100 90 80 70 60 50 40 30 20 10 0 -10
f1 (ppm)

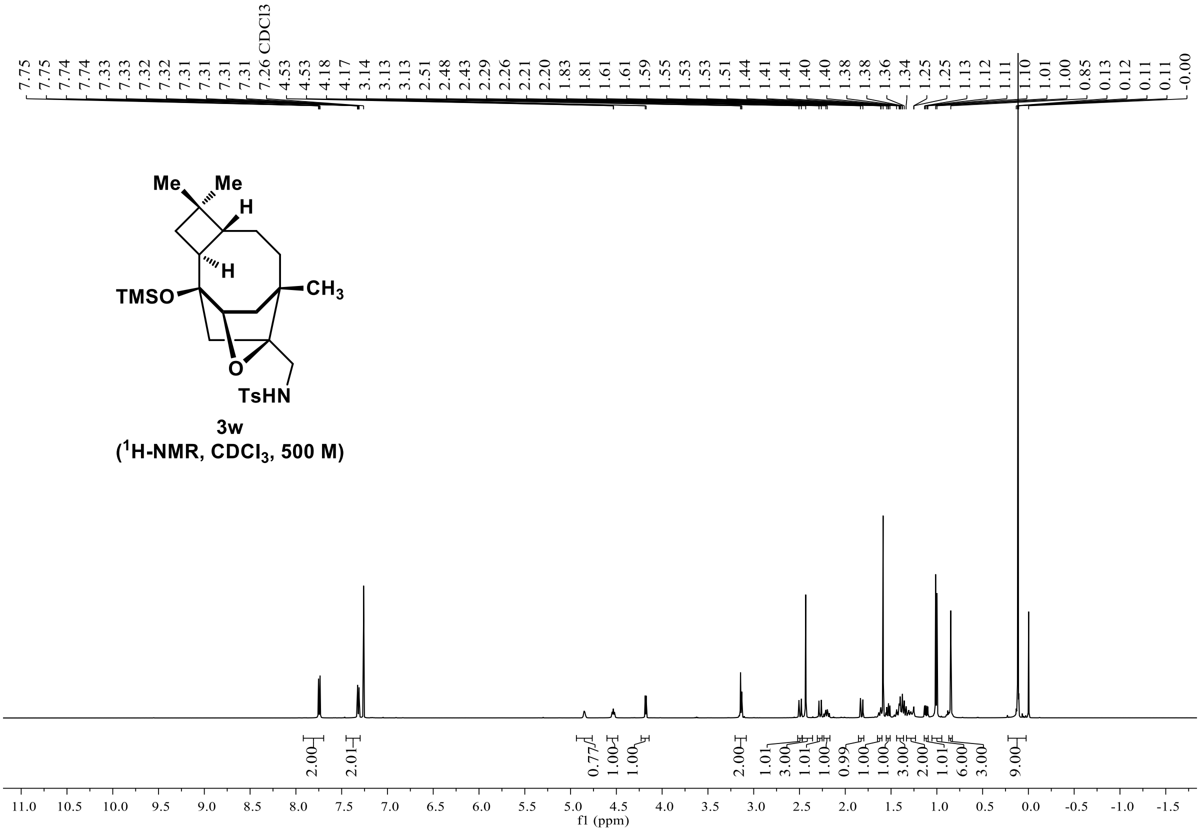
7.75 7.75 7.74 7.74 7.33 7.33 7.32 7.32 7.31 7.31 7.31 7.31 7.26 CDCl3 4.53 4.53 4.18 4.17 3.14 3.13 3.13 2.51 2.48 2.43 2.29 2.26 2.21 2.20 1.83 1.81 1.61 1.61 1.59 1.55 1.53 1.53 1.51 1.44 1.41 1.41 1.40 1.40 1.38 1.38 1.36 1.34 1.25 1.25 1.13 1.12 1.11 1.10 1.01 1.00 0.85 0.13 0.12 0.11 0.11 -0.00
Me
Me
H
H
TMSO
CH3
O
TsHN
3w
(1H-NMR, CDCl3, 500 M)
2.00 2.01 0.77 1.00 1.00 2.00 1.01 3.00 1.01 1.00 0.99 1.00 1.00 3.00 2.00 1.01 6.00 3.00 9.00
f1 (ppm)

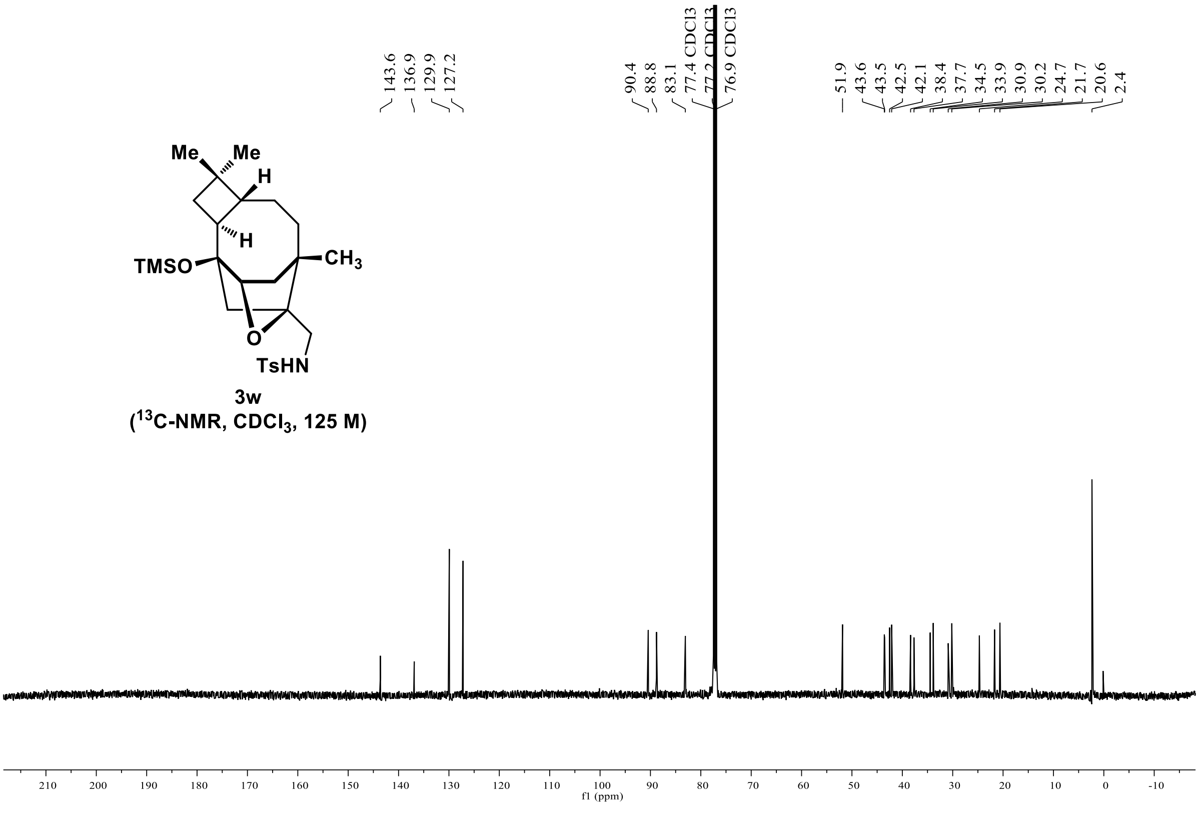
143.6 136.9 129.9 127.2 90.4 88.8 83.1 77.4 CDCl3 77.2 CDCl3 76.9 CDCl3 51.9 43.6 43.5 42.5 42.1 38.4 37.7 34.5 33.9 30.9 30.2 24.7 21.7 20.6 2.4
Me
Me
H
H
TMSO
CH3
O
TsHN
3w
(13C-NMR, CDCl3, 125 M)
f1 (ppm)

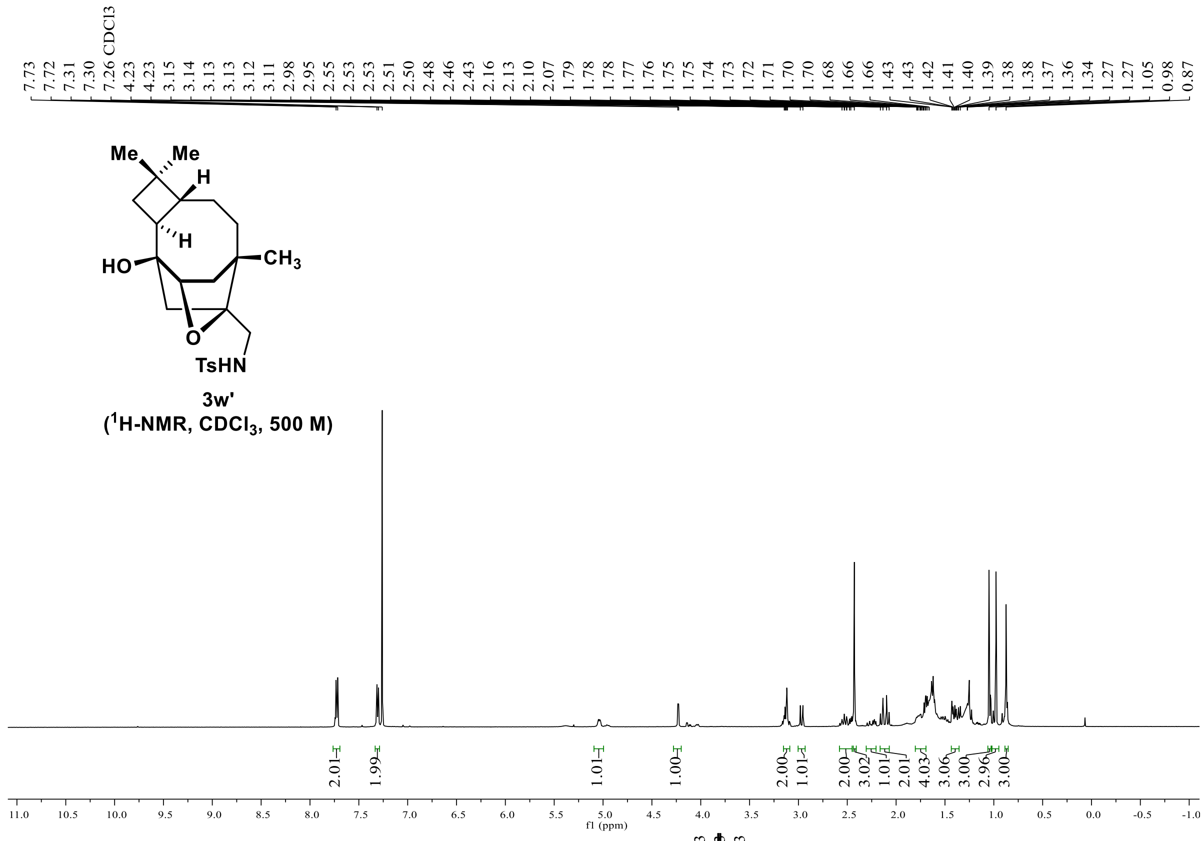
7.73 7.72 7.31 7.30 7.26 CDCl3 4.23 4.23 3.15 3.14 3.13 3.13 3.12 3.11 2.98 2.95 2.55 2.53 2.53 2.51 2.50 2.48 2.46 2.43 2.16 2.13 2.10 2.07 1.79 1.78 1.78 1.77 1.76 1.75 1.75 1.74 1.73 1.72 1.71 1.70 1.70 1.68 1.66 1.66 1.43 1.43 1.42 1.41 1.40 1.39 1.38 1.38 1.37 1.36 1.34 1.27 1.27 1.05 0.98 0.87
Me
Me
H
H
HO
CH3
O
TsHN
3w'
(1H-NMR, CDCl3, 500 M)
2.01 1.99 1.01 1.00 2.00 1.01 2.00 3.02 1.01 2.01 4.03 3.06 3.00 2.96 3.00
f1 (ppm)

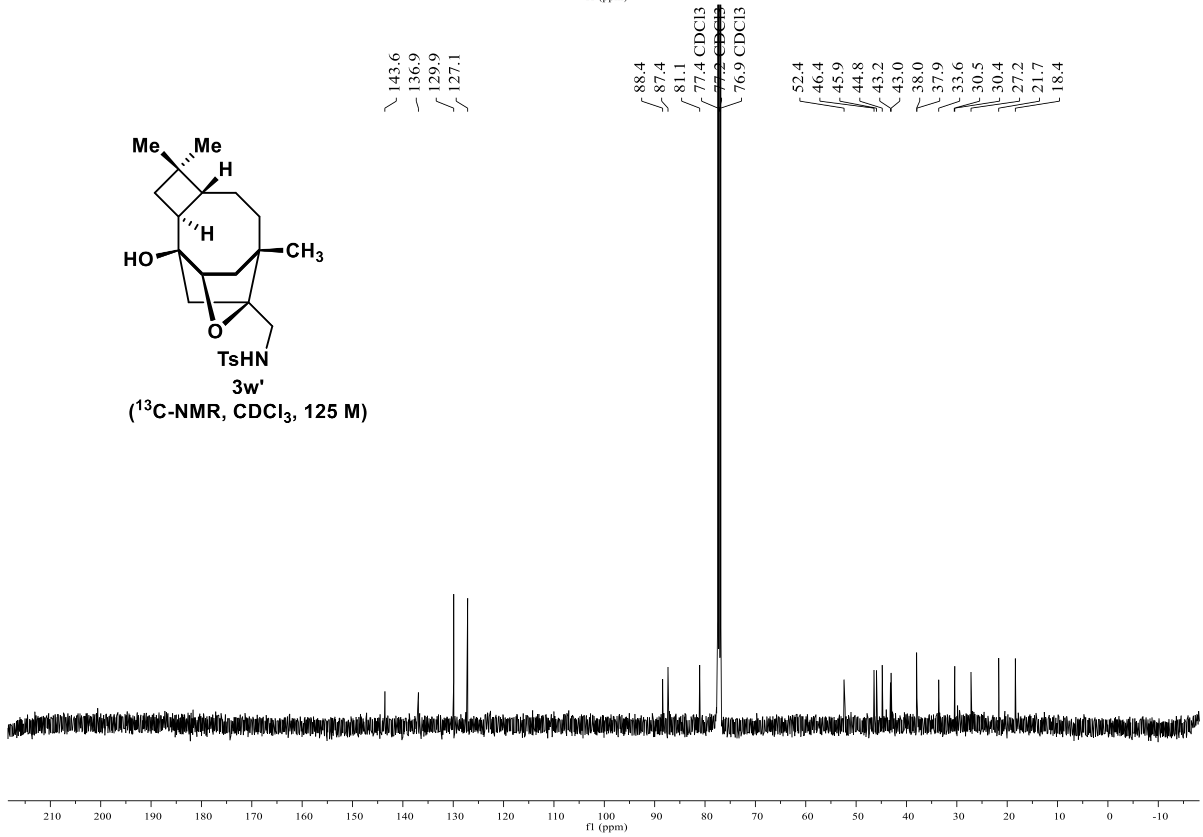
143.6 136.9 129.9 127.1
88.4 87.4 81.1 77.4 CDCl3 77.2 CDCl3 76.9 CDCl3
52.4 46.4 45.9 44.8 43.2 43.0 38.0 37.9 33.6 30.5 30.4 27.2 21.7 18.4
Me
Me
H
H
HO
CH3
O
TsHN
3w'
(13C-NMR, CDCl3, 125 M)
f1 (ppm)

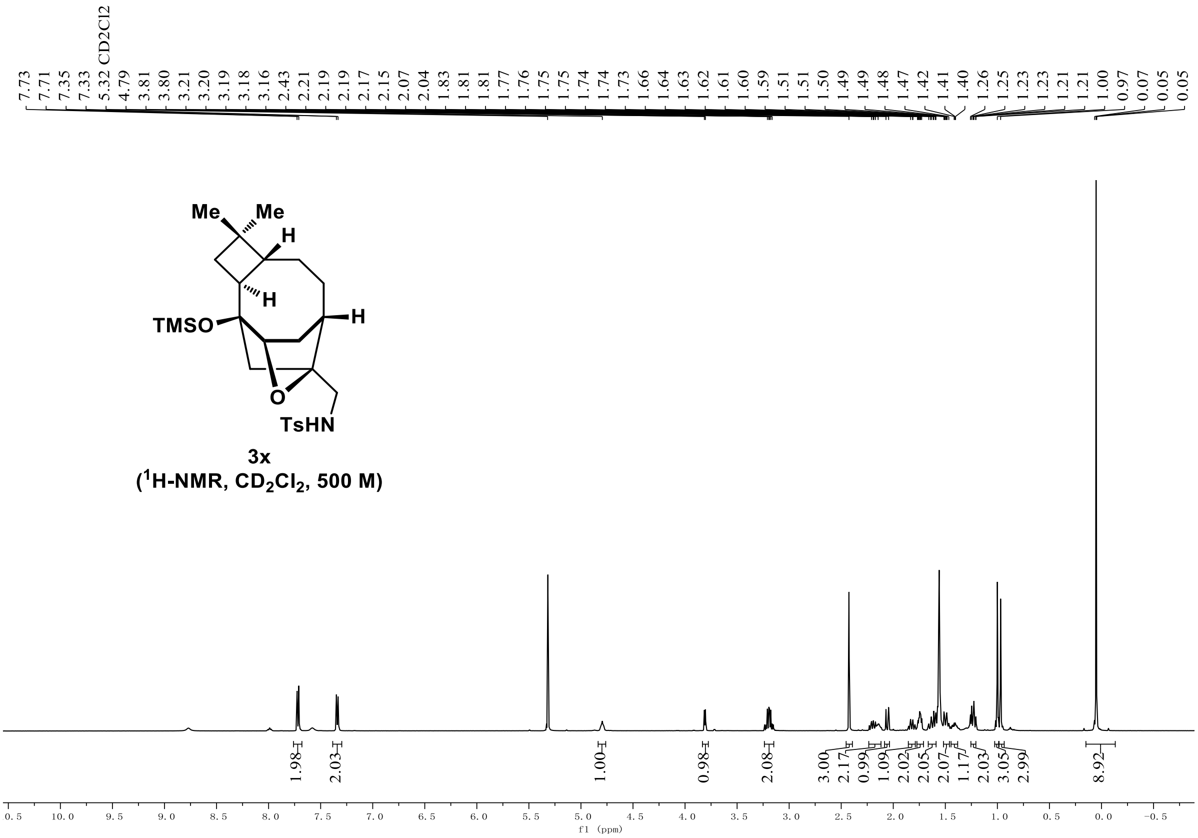

3x
(^{1}H-NMR, CD_2Cl_2, 500 M)

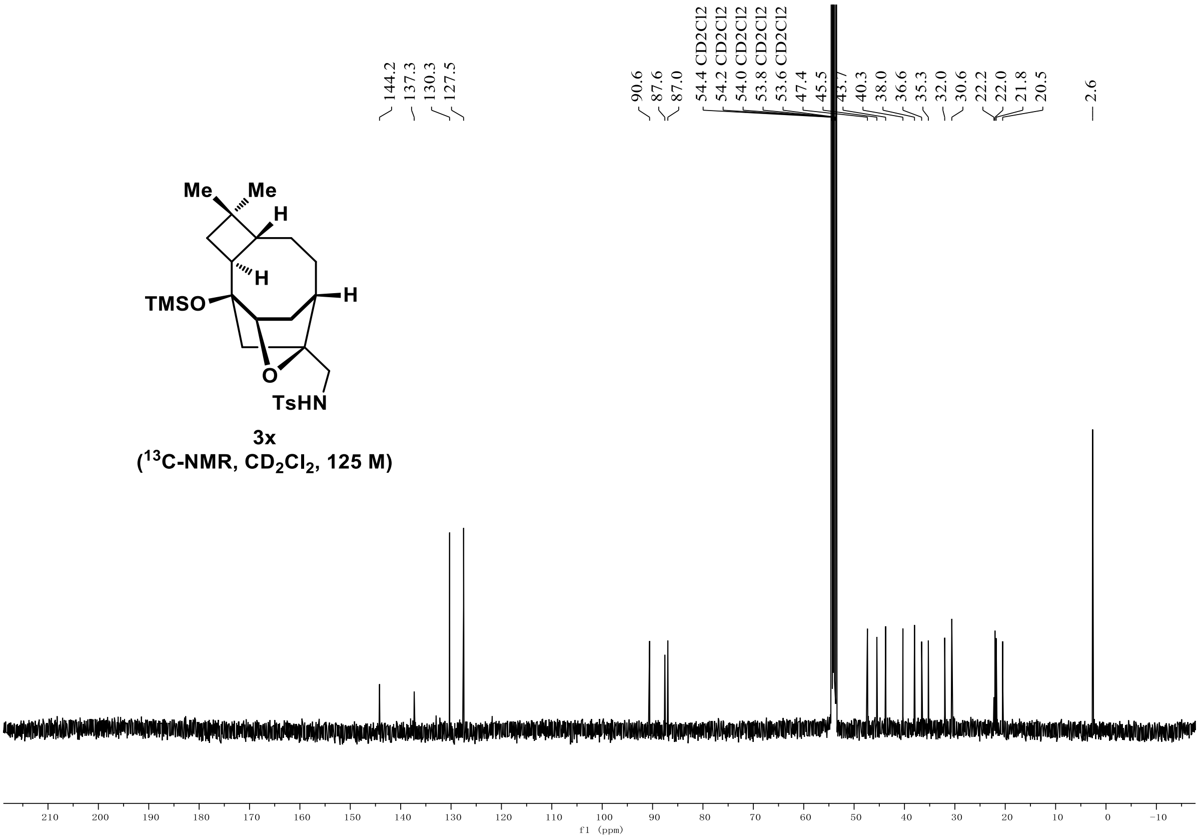

3x
(^{13}C-NMR, CD_2Cl_2, 125 M)

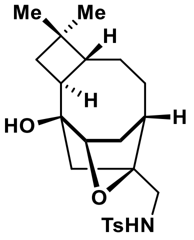

3x'
(^{1}H-NMR, $CDCl_3$, 500 M)

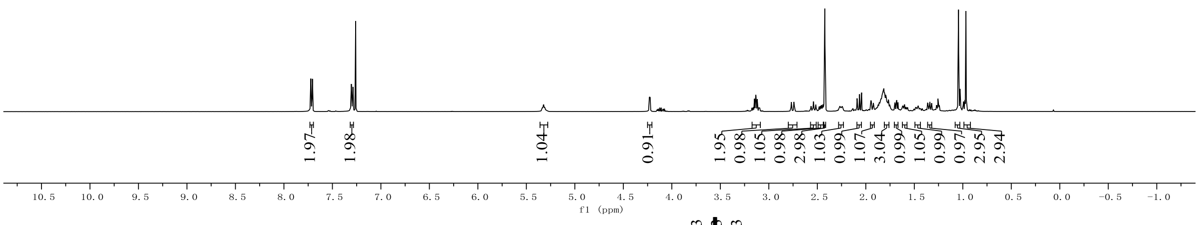

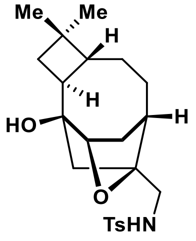

3x'
(^{13}C-NMR, $CDCl_3$, 125 M)

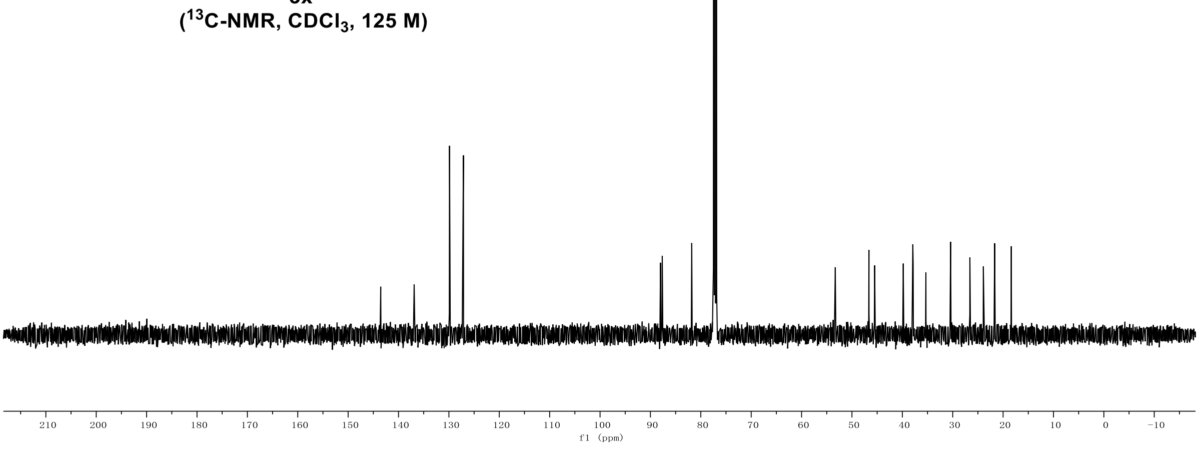

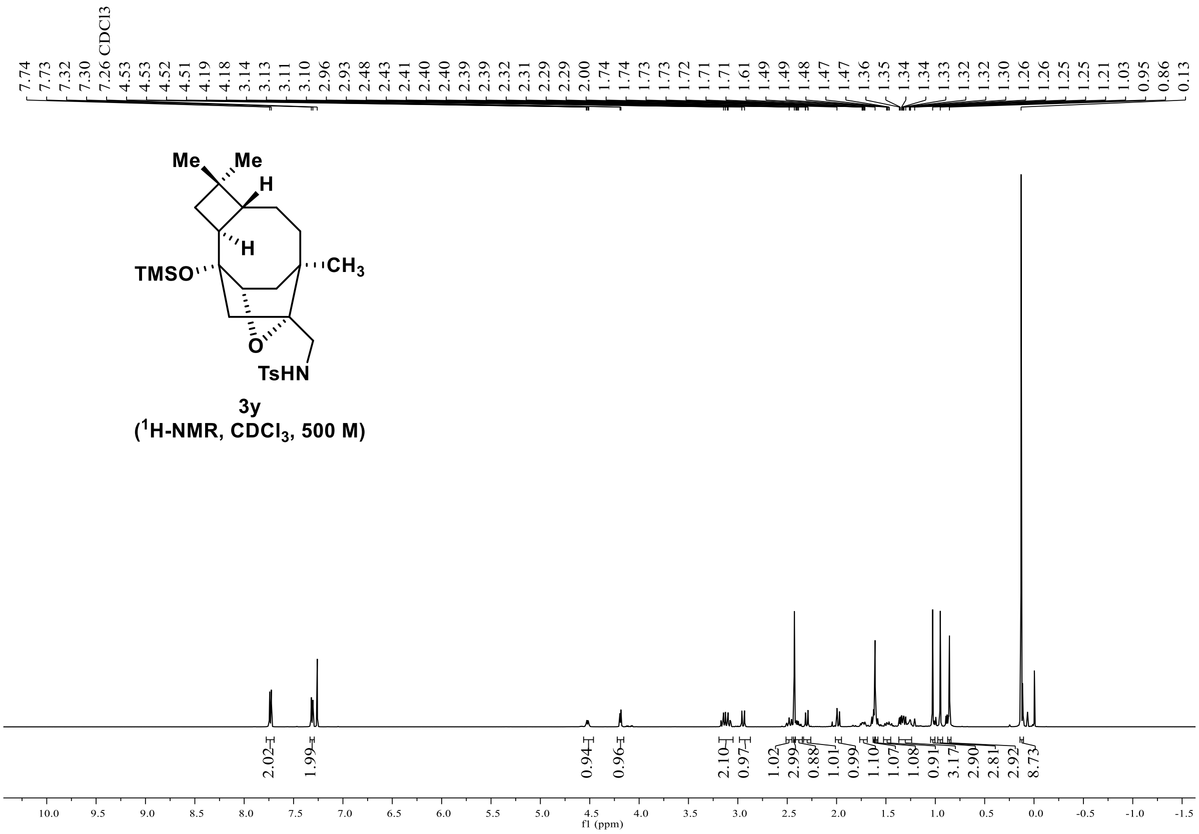

3y
(^{1}H-NMR, $CDCl_3$, 500 M)

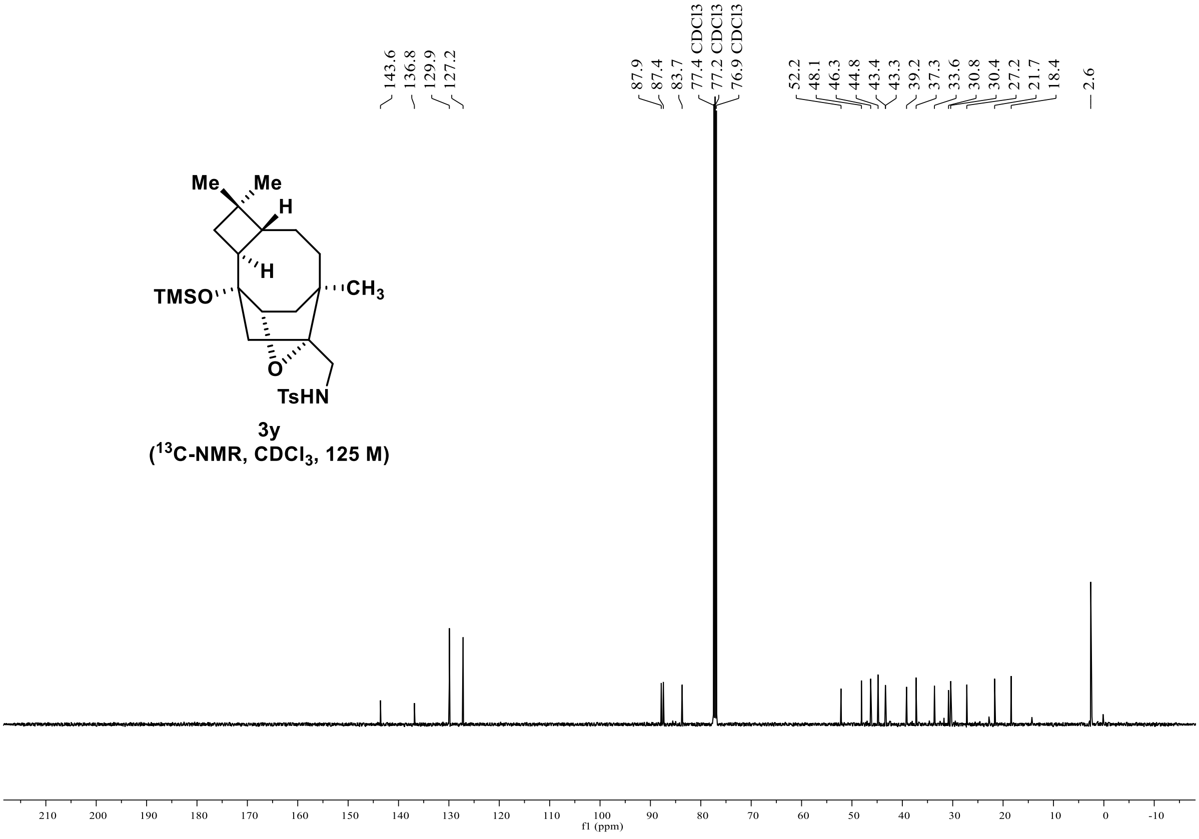

3y
(^{13}C-NMR, $CDCl_3$, 125 M)

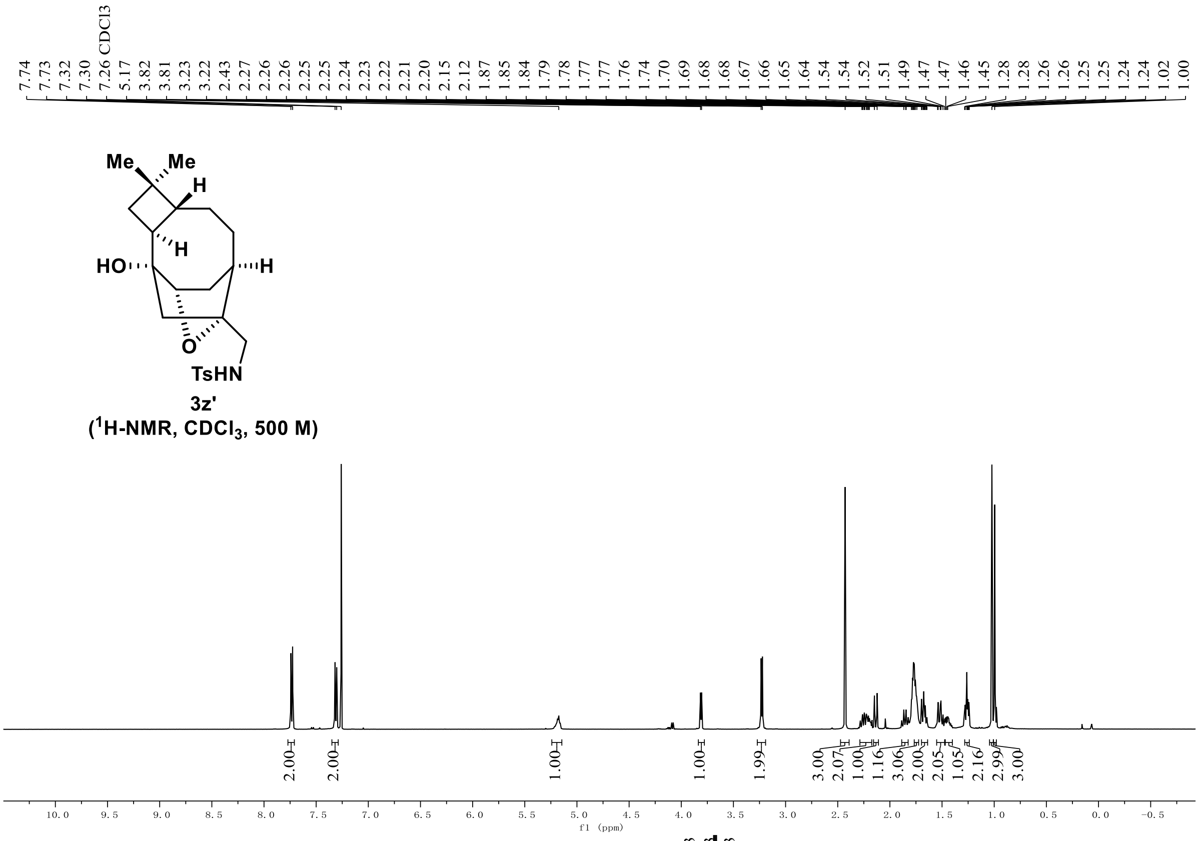
7.74 7.73 7.32 7.30 7.26 CDCl3 5.17 3.82 3.81 3.23 3.22 2.43 2.27 2.26 2.26 2.25 2.25 2.24 2.23 2.22 2.21 2.20 2.15 2.12 1.87 1.85 1.84 1.79 1.78 1.77 1.77 1.76 1.74 1.70 1.69 1.68 1.68 1.67 1.66 1.65 1.64 1.54 1.54 1.52 1.51 1.49 1.47 1.47 1.46 1.45 1.28 1.28 1.26 1.26 1.25 1.25 1.24 1.24 1.02 1.00
Me
Me
H
H
HO
H
O
TsHN
3z'
(1H-NMR, CDCl3, 500 M)
2.00 2.00 1.00 1.00 1.99 3.00 2.07 1.00 1.16 3.06 2.00 2.05 1.05 2.16 2.99 3.00
f1 (ppm)

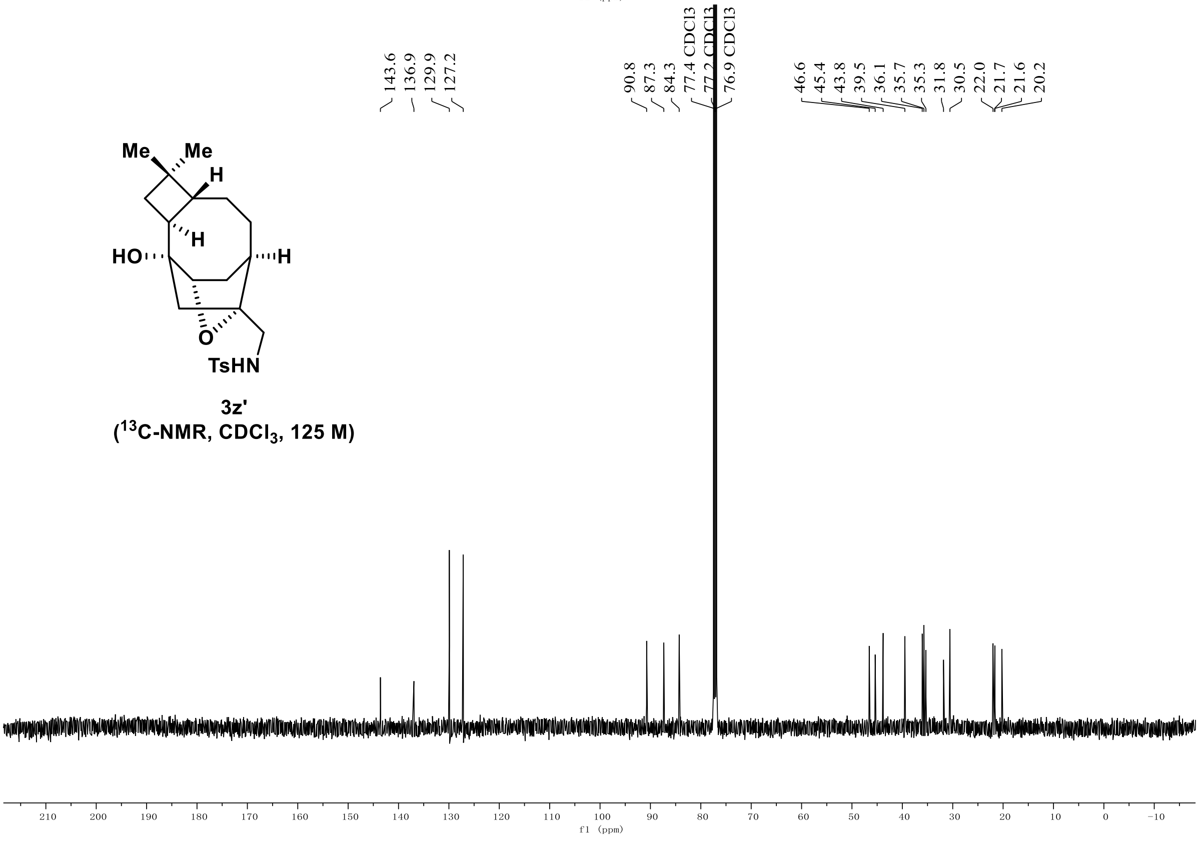
143.6 136.9 129.9 127.2 90.8 87.3 84.3 77.4 CDCl3 77.2 CDCl3 76.9 CDCl3 46.6 45.4 43.8 39.5 36.1 35.7 35.3 31.8 30.5 22.0 21.7 21.6 20.2
Me
Me
H
H
HO
H
O
TsHN
3z'
(13C-NMR, CDCl3, 125 M)
f1 (ppm)

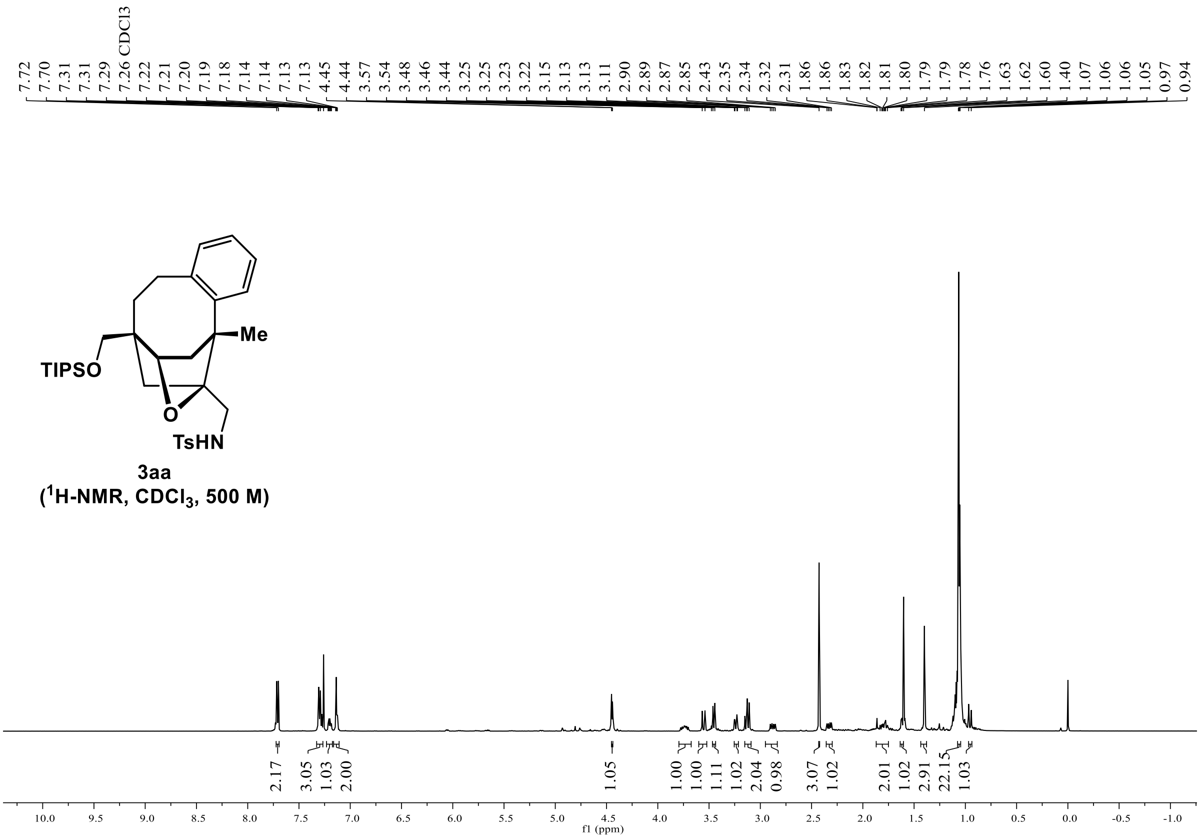
7.72 7.70 7.31 7.31 7.29 7.26 CDCl3 7.22 7.21 7.20 7.19 7.18 7.14 7.14 7.13 7.13 4.45 4.44 3.57 3.54 3.48 3.46 3.44 3.25 3.25 3.23 3.22 3.15 3.13 3.13 3.11 2.90 2.89 2.87 2.85 2.43 2.35 2.34 2.32 2.31 1.86 1.86 1.83 1.82 1.81 1.80 1.79 1.79 1.78 1.76 1.63 1.62 1.60 1.40 1.07 1.06 1.06 1.05 0.97 0.94
Me
TIPSO
O
TsHN
3aa
(^{1}H-NMR, CDCl$_3$, 500 M)
2.17 3.05 1.03 2.00 1.05 1.00 1.00 1.11 1.02 2.04 0.98 3.07 1.02 2.01 1.02 2.91 22.15 1.03
10.0 9.5 9.0 8.5 8.0 7.5 7.0 6.5 6.0 5.5 5.0 4.5 4.0 3.5 3.0 2.5 2.0 1.5 1.0 0.5 0.0 -0.5 -1.0
f1 (ppm)

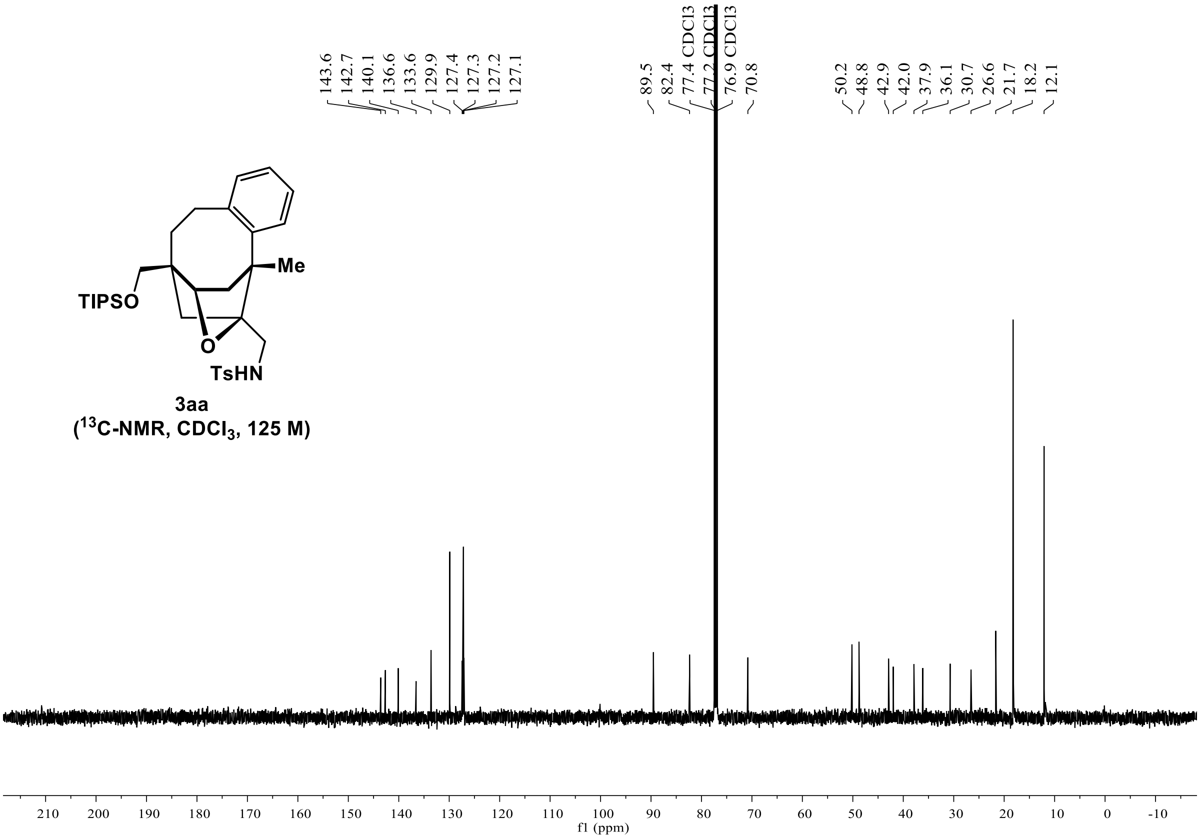
143.6 142.7 140.1 136.6 133.6 129.9 127.4 127.3 127.2 127.1 89.5 82.4 77.4 CDCl3 77.2 CDCl3 76.9 CDCl3 70.8 50.2 48.8 42.9 42.0 37.9 36.1 30.7 26.6 21.7 18.2 12.1
Me
TIPSO
O
TsHN
3aa
(^{13}C-NMR, CDCl$_3$, 125 M)
210 200 190 180 170 160 150 140 130 120 110 100 90 80 70 60 50 40 30 20 10 0 -10
f1 (ppm)

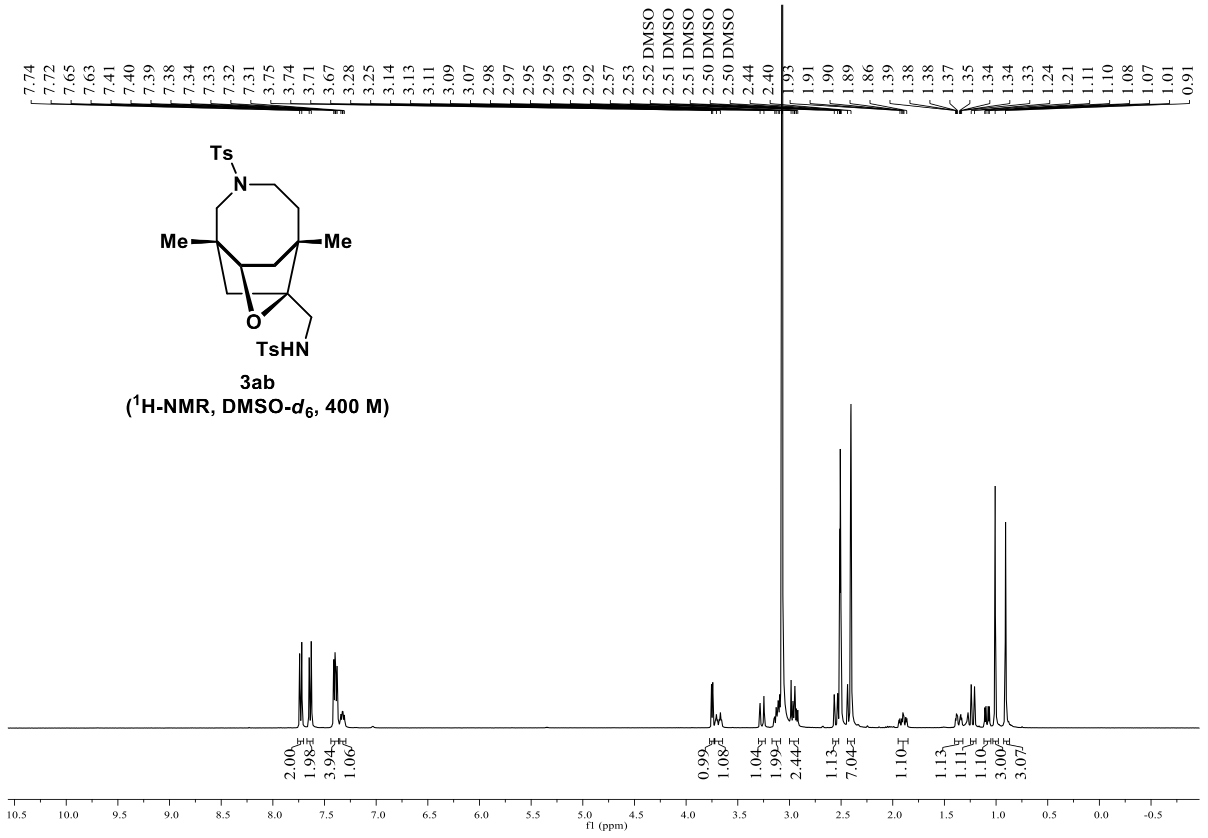
7.74
7.72
7.65
7.63
7.41
7.40
7.39
7.38
7.34
7.33
7.32
7.31
3.75
3.74
3.71
3.67
3.28
3.25
3.14
3.13
3.11
3.09
3.07
2.98
2.97
2.95
2.95
2.93
2.92
2.57
2.53
2.52 DMSO
2.51 DMSO
2.51 DMSO
2.50 DMSO
2.50 DMSO
2.44
2.40
1.93
1.91
1.90
1.89
1.86
1.39
1.38
1.37
1.35
1.34
1.34
1.33
1.24
1.21
1.11
1.10
1.08
1.07
1.01
0.91
Ts
N
Me
Me
O
TsHN
3ab
(^{1}H-NMR, DMSO-d_6, 400 M)
2.00
1.98
3.94
1.06
0.99
1.08
1.04
1.99
2.44
1.13
7.04
1.10
1.13
1.11
1.10
3.00
3.07
10.5 10.0 9.5 9.0 8.5 8.0 7.5 7.0 6.5 6.0 5.5 5.0 4.5 4.0 3.5 3.0 2.5 2.0 1.5 1.0 0.5 0.0 -0.5
f1 (ppm)

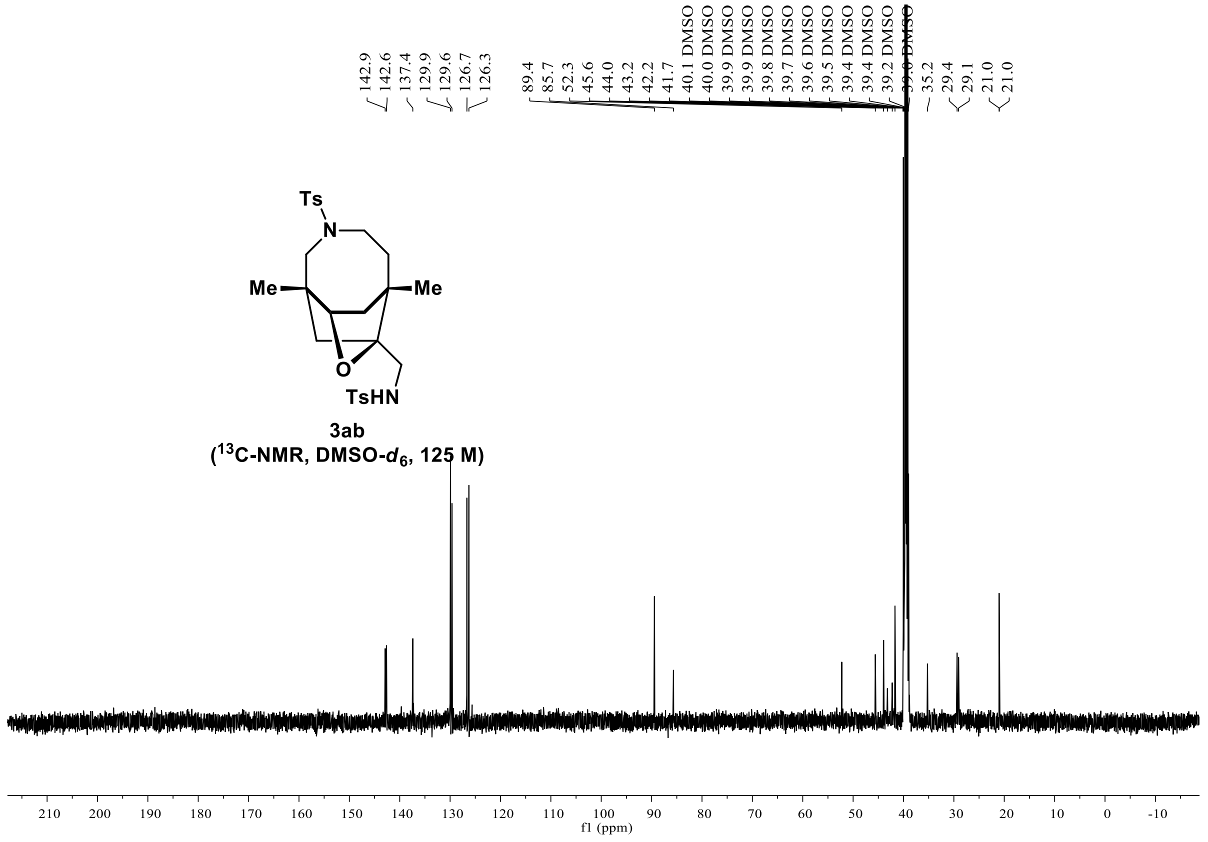
142.9
142.6
137.4
129.9
129.6
126.7
126.3
89.4
85.7
52.3
45.6
44.0
43.2
42.2
41.7
40.1 DMSO
40.0 DMSO
39.9 DMSO
39.9 DMSO
39.8 DMSO
39.7 DMSO
39.6 DMSO
39.5 DMSO
39.4 DMSO
39.4 DMSO
39.2 DMSO
[illegible] DMSO
35.2
29.4
29.1
21.0
21.0
Ts
N
Me
Me
O
TsHN
3ab
(^{13}C-NMR, DMSO-d_6, 125 M)
210 200 190 180 170 160 150 140 130 120 110 100 90 80 70 60 50 40 30 20 10 0 -10
f1 (ppm)

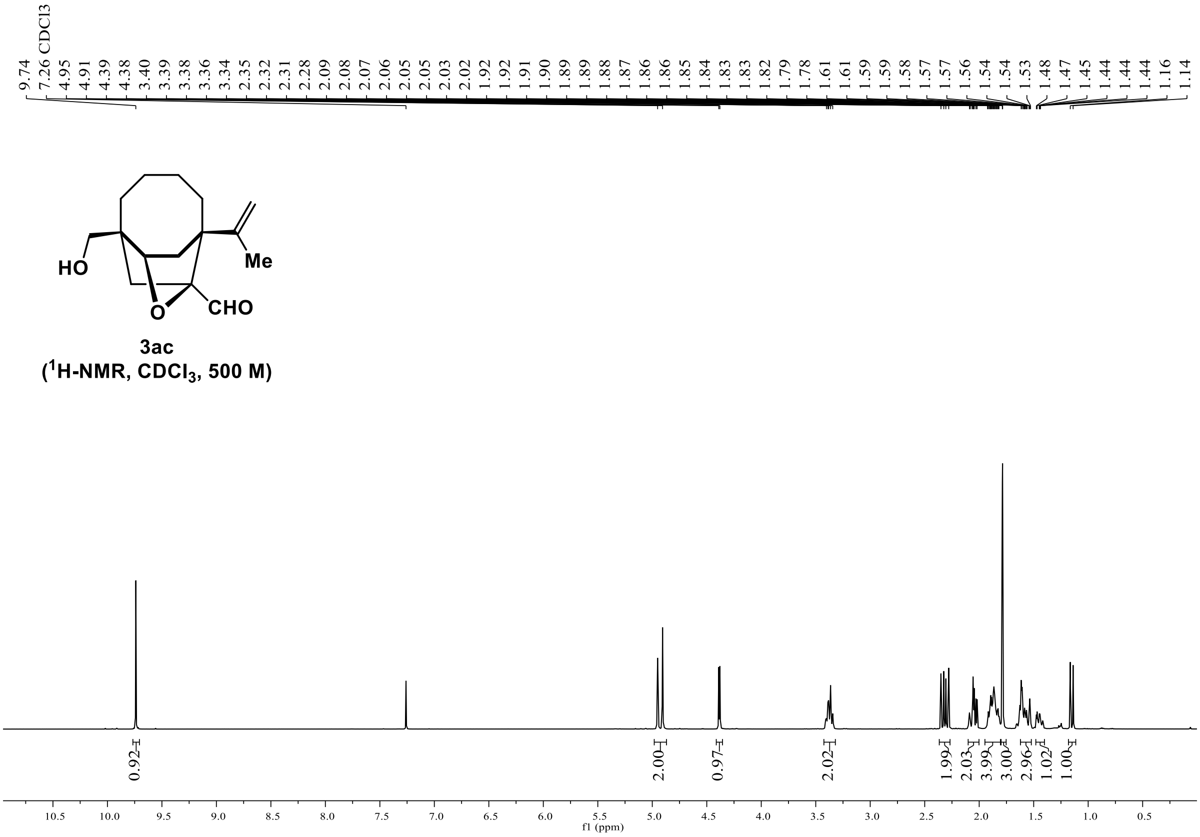

3ac
(^{1}H-NMR, $CDCl_3$, 500 M)

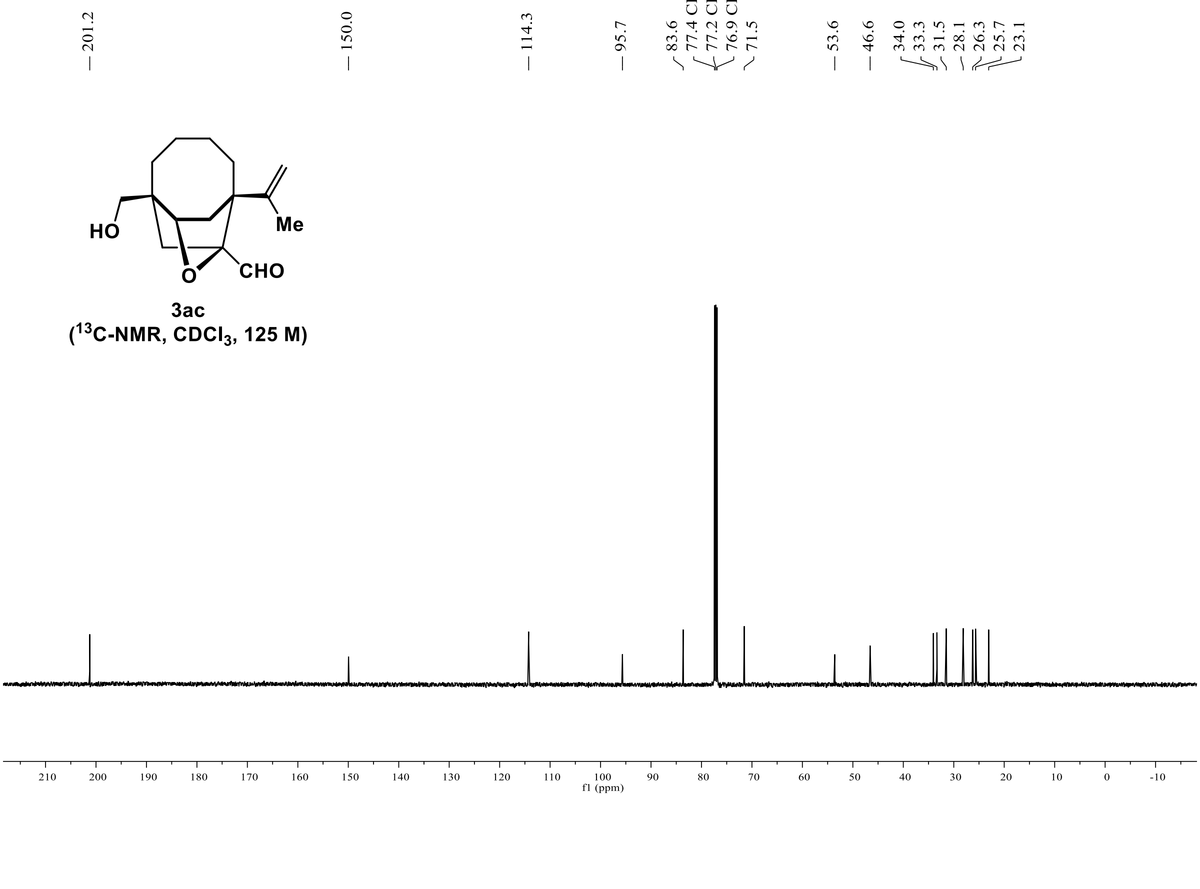

3ac
(^{13}C-NMR, $CDCl_3$, 125 M)

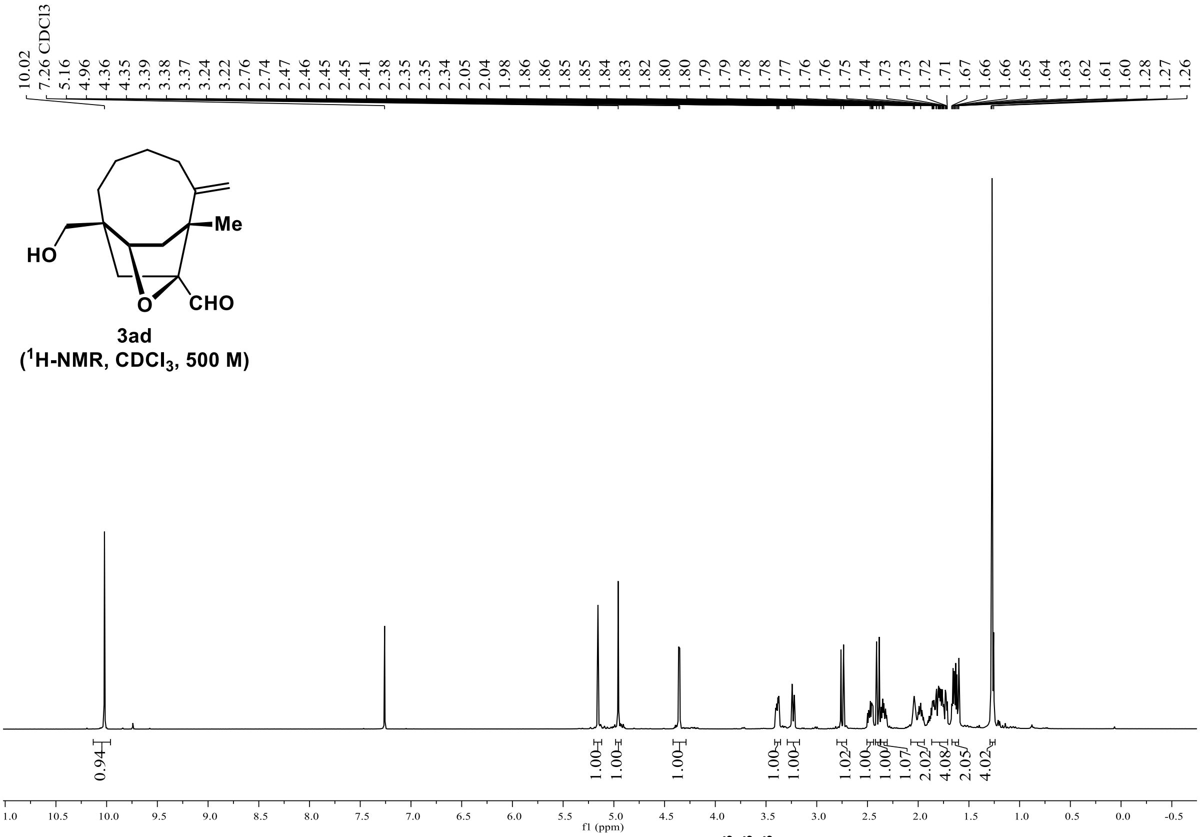
10.02
7.26 CDCl3
5.16
4.96
4.36
4.35
3.39
3.38
3.37
3.24
3.22
2.76
2.74
2.47
2.46
2.45
2.45
2.41
2.38
2.35
2.35
2.34
2.05
2.04
1.98
1.86
1.86
1.85
1.85
1.84
1.83
1.82
1.80
1.80
1.79
1.78
1.78
1.77
1.76
1.76
1.75
1.74
1.73
1.73
1.72
1.71
1.67
1.66
1.66
1.65
1.64
1.63
1.62
1.61
1.60
1.28
1.27
1.26
Me
HO
O
CHO
3ad
(^{1}H-NMR, $CDCl_3$, 500 M)
0.94
1.00
1.00
1.00
1.00
1.00
1.02
1.00
1.00
1.07
2.02
4.08
2.05
4.02
f1 (ppm)

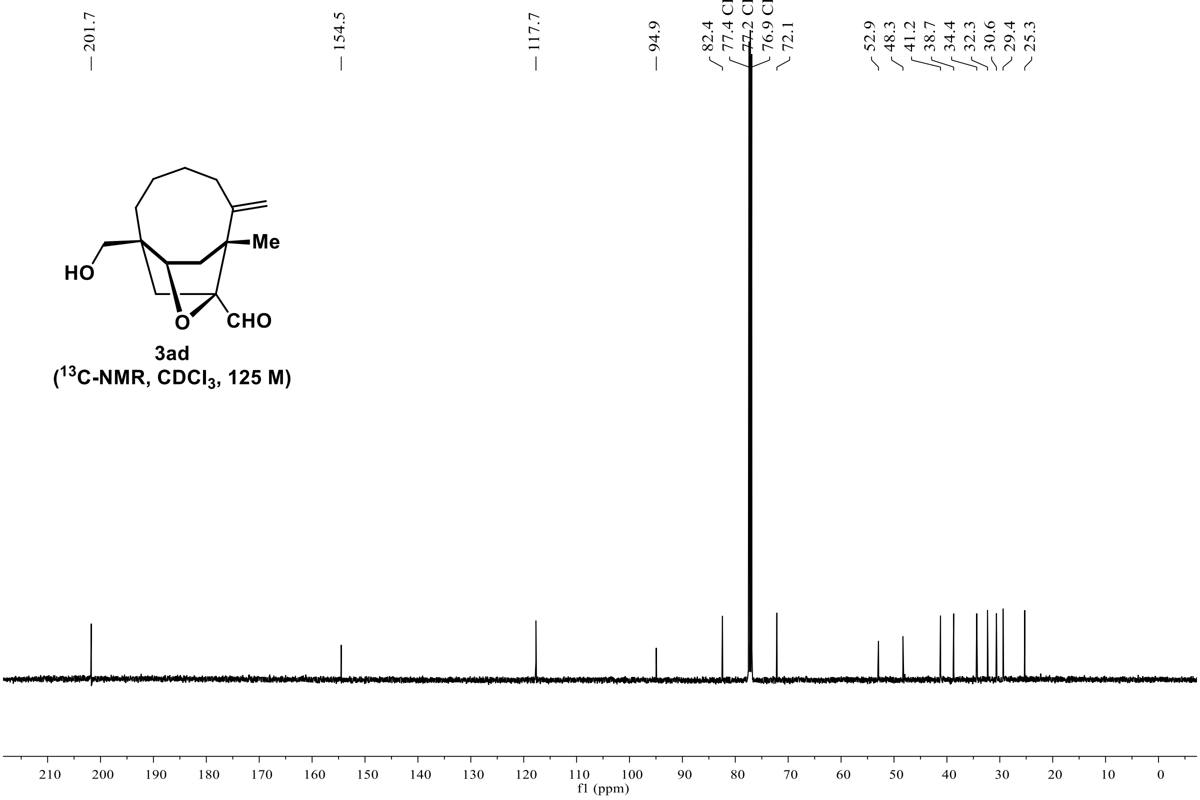
201.7
154.5
117.7
94.9
82.4
77.4 CDCl3
77.2 CDCl3
76.9 CDCl3
72.1
52.9
48.3
41.2
38.7
34.4
32.3
30.6
29.4
25.3
Me
HO
O
CHO
3ad
(^{13}C-NMR, $CDCl_3$, 125 M)
f1 (ppm)

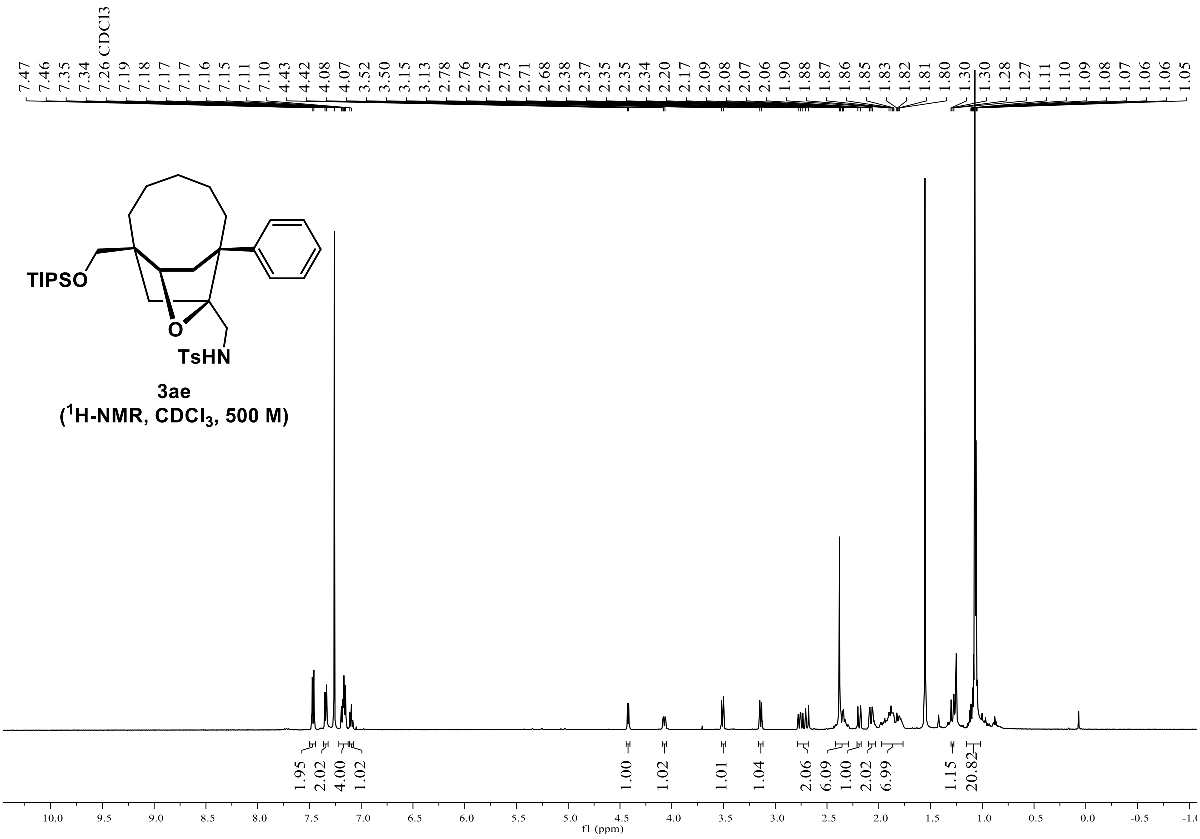

3ae
(^{1}H-NMR, $CDCl_3$, 500 M)

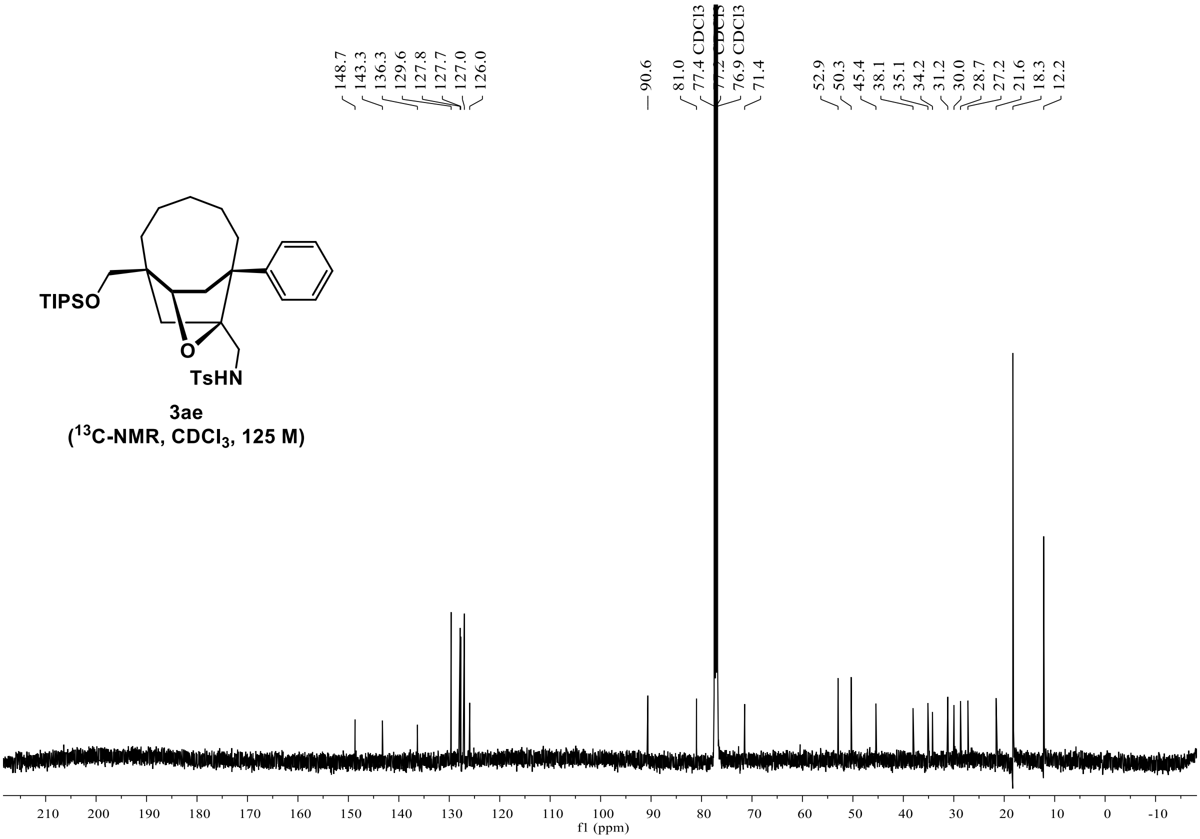

3ae
(^{13}C-NMR, $CDCl_3$, 125 M)

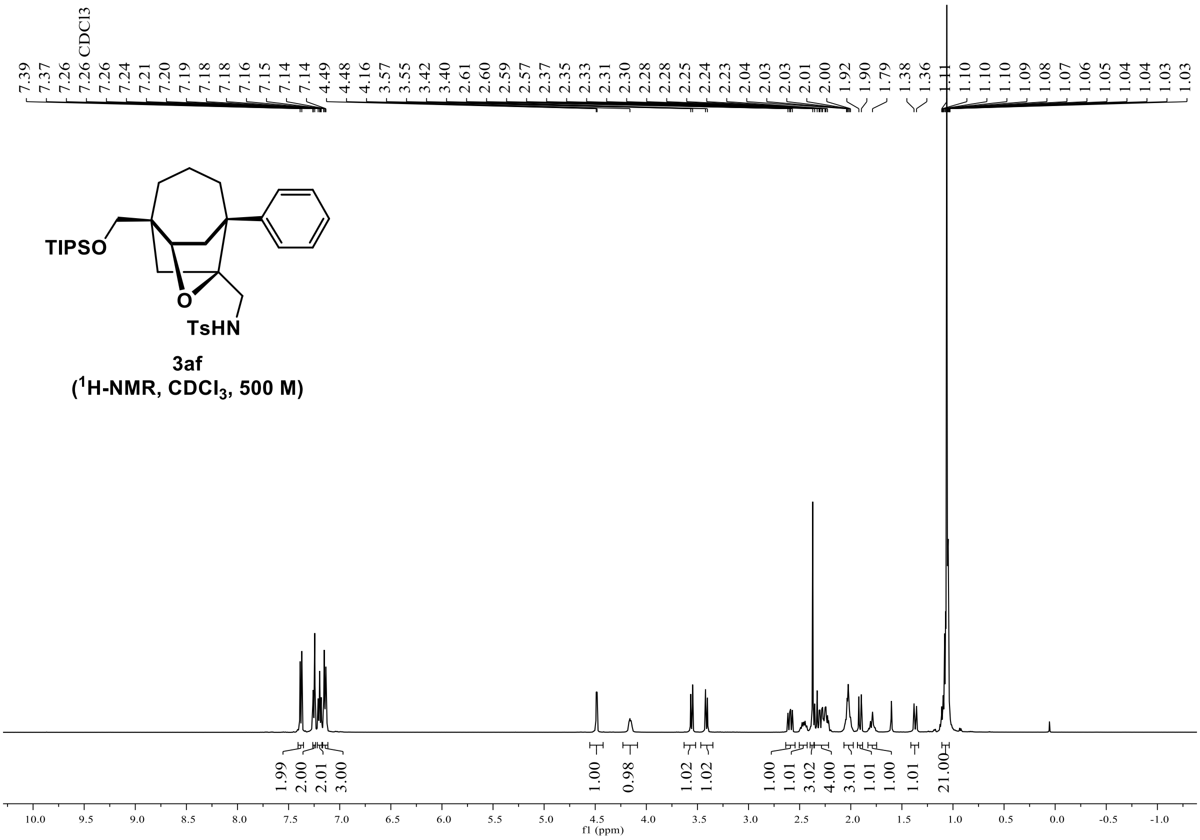

3af
(^{1}H-NMR, $CDCl_3$, 500 M)

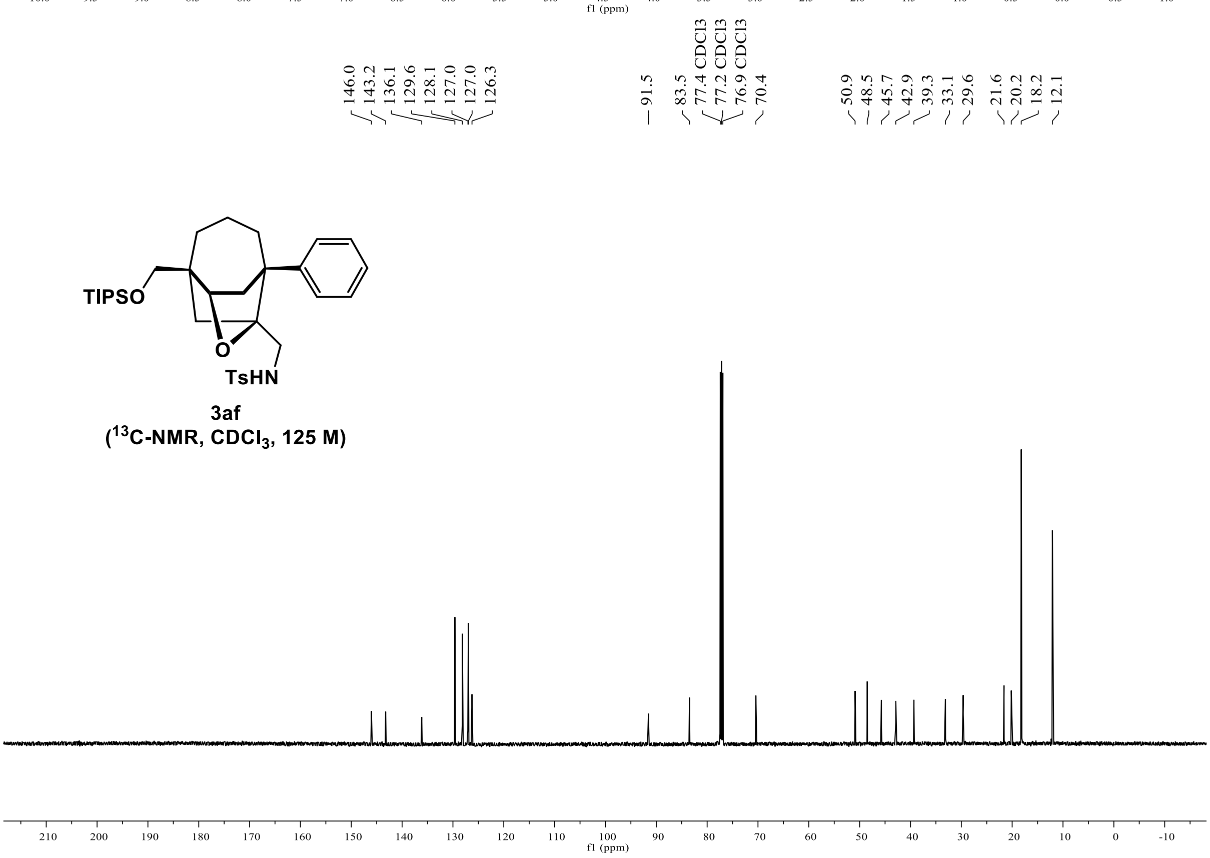

3af
(^{13}C-NMR, $CDCl_3$, 125 M)

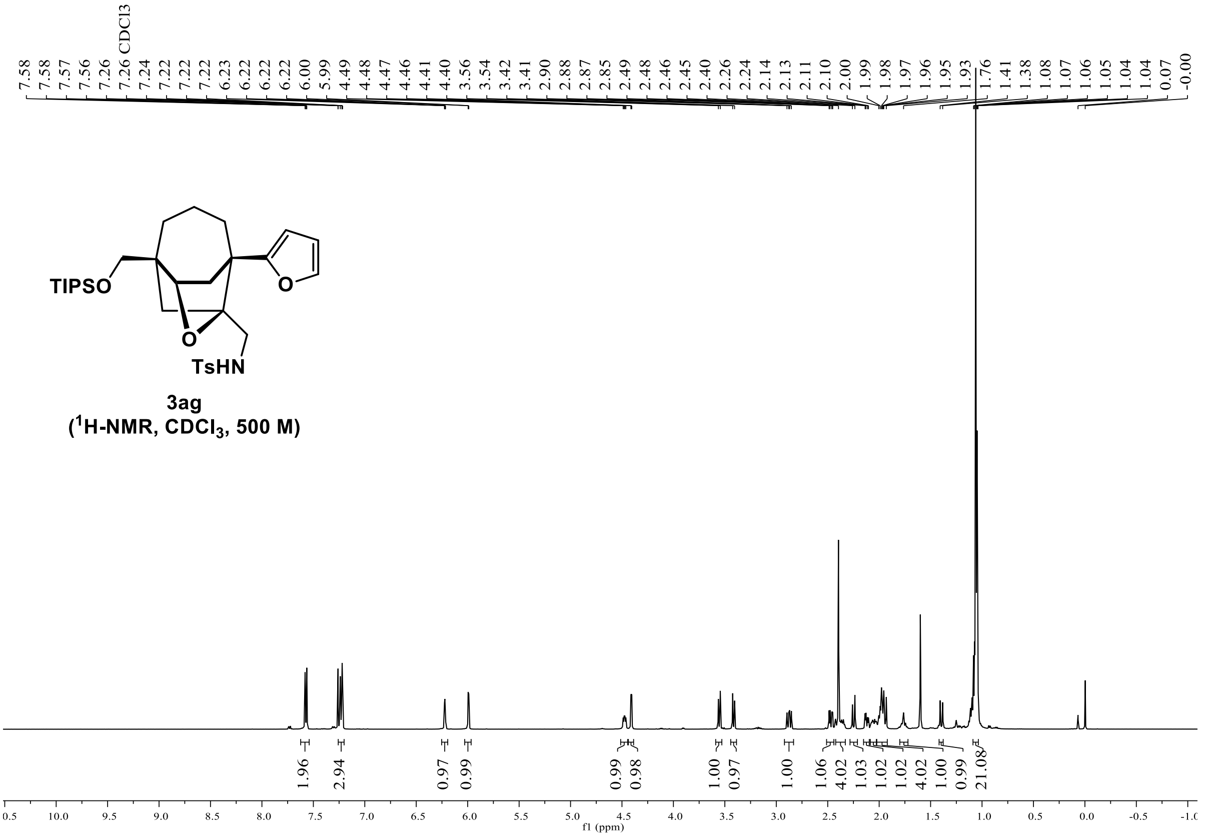

3ag
(^{1}H-NMR, $CDCl_3$, 500 M)

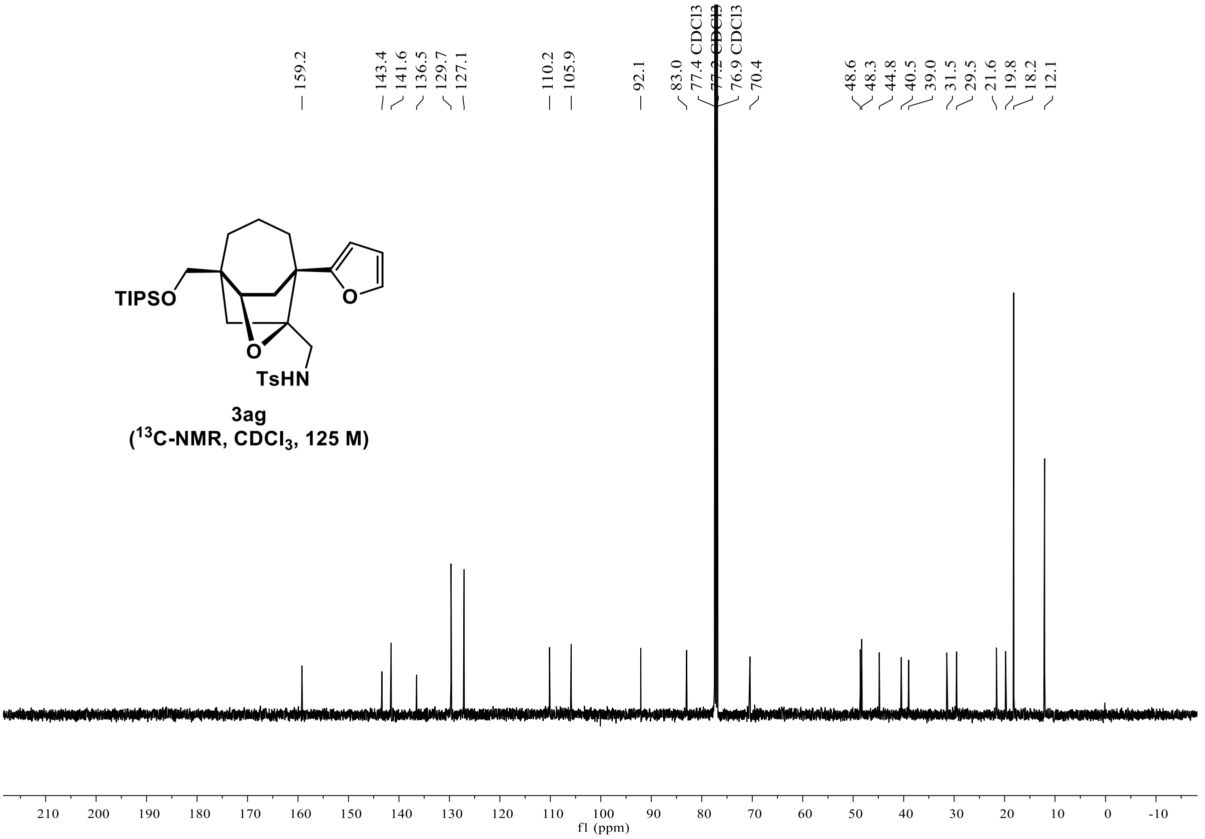

3ag
(^{13}C-NMR, $CDCl_3$, 125 M)

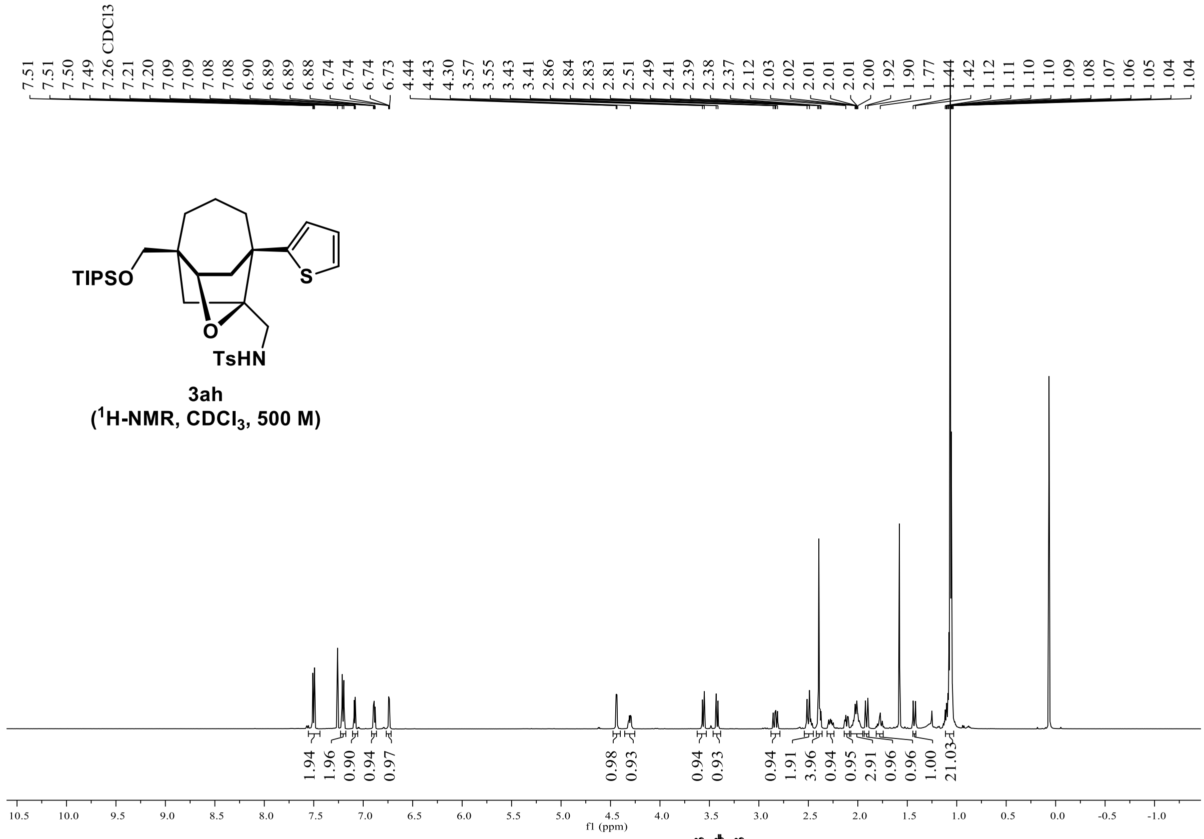
7.51 7.51 7.50 7.49 7.26 CDCl3 7.21 7.20 7.09 7.09 7.08 7.08 6.90 6.89 6.89 6.88 6.74 6.74 6.74 6.73 4.44 4.43 4.30 3.57 3.55 3.43 3.41 2.86 2.84 2.83 2.81 2.51 2.49 2.41 2.39 2.38 2.37 2.12 2.03 2.02 2.01 2.01 2.01 2.00 1.92 1.90 1.77 1.44 1.42 1.12 1.11 1.10 1.10 1.09 1.08 1.07 1.06 1.05 1.04 1.04
TIPSO
S
O
TsHN
3ah
(^{1}H-NMR, CDCl$_3$, 500 M)
1.94 1.96 0.90 0.94 0.97 0.98 0.93 0.94 0.93 0.94 1.91 3.96 0.94 0.95 2.91 0.96 0.96 1.00 21.03
f1 (ppm)

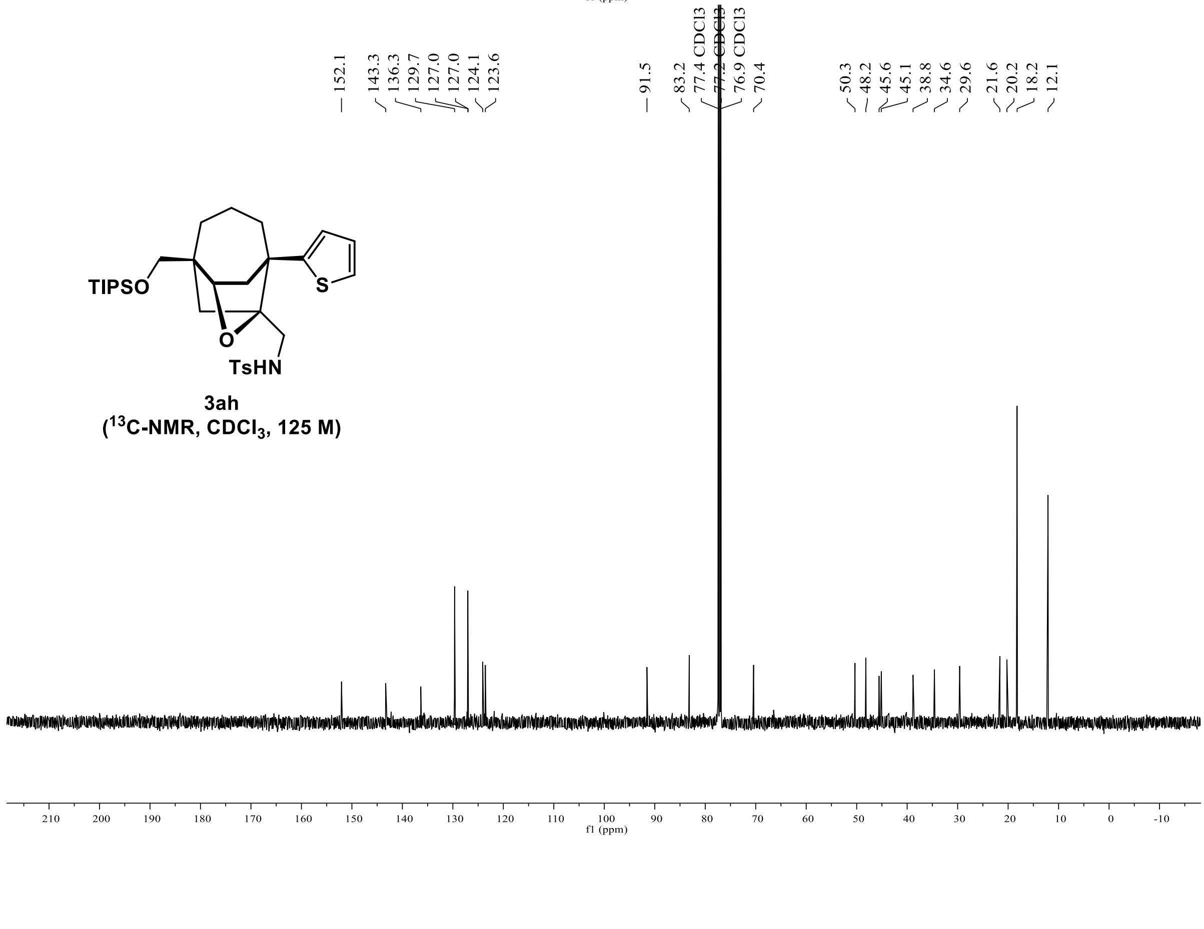
152.1 143.3 136.3 129.7 127.0 127.0 124.1 123.6 91.5 83.2 77.4 CDCl3 77.2 CDCl3 76.9 CDCl3 70.4 50.3 48.2 45.6 45.1 38.8 34.6 29.6 21.6 20.2 18.2 12.1
TIPSO
S
O
TsHN
3ah
(^{13}C-NMR, CDCl$_3$, 125 M)
f1 (ppm)

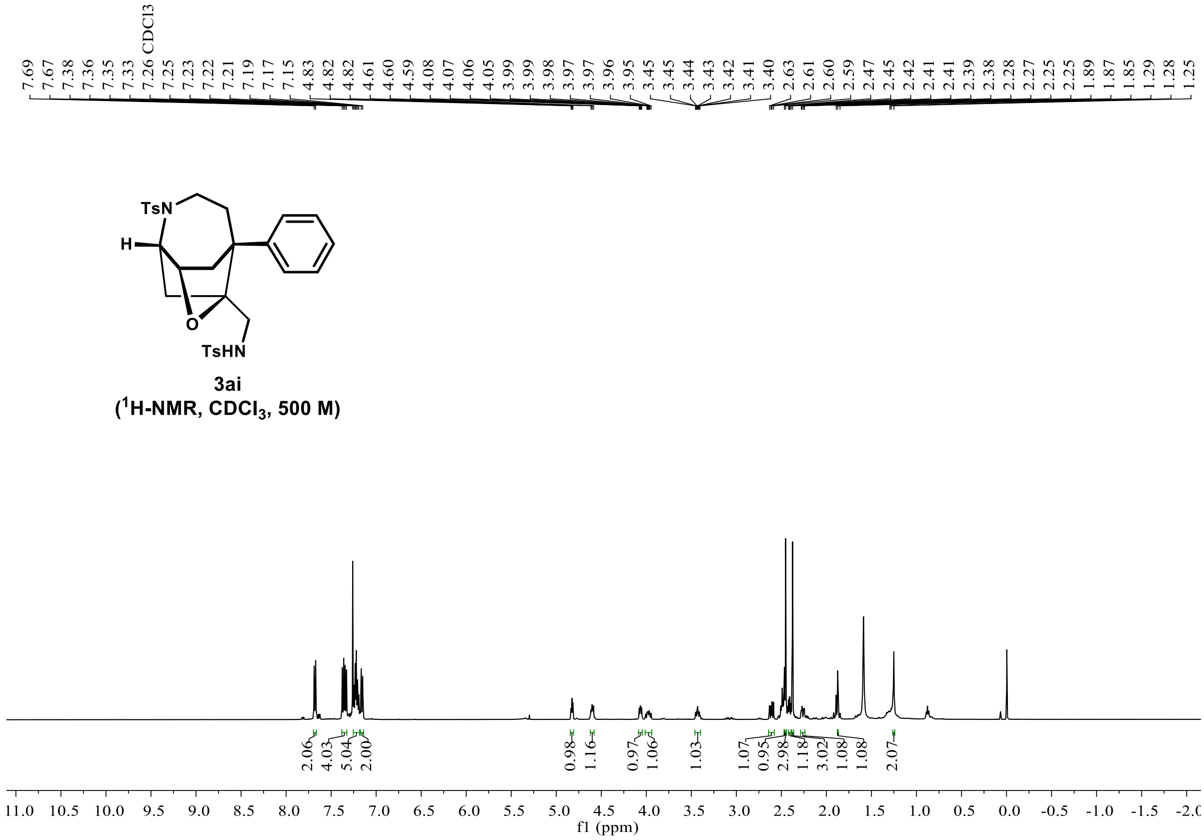

3ai
(^{1}H-NMR, CDCl$_3$, 500 M)

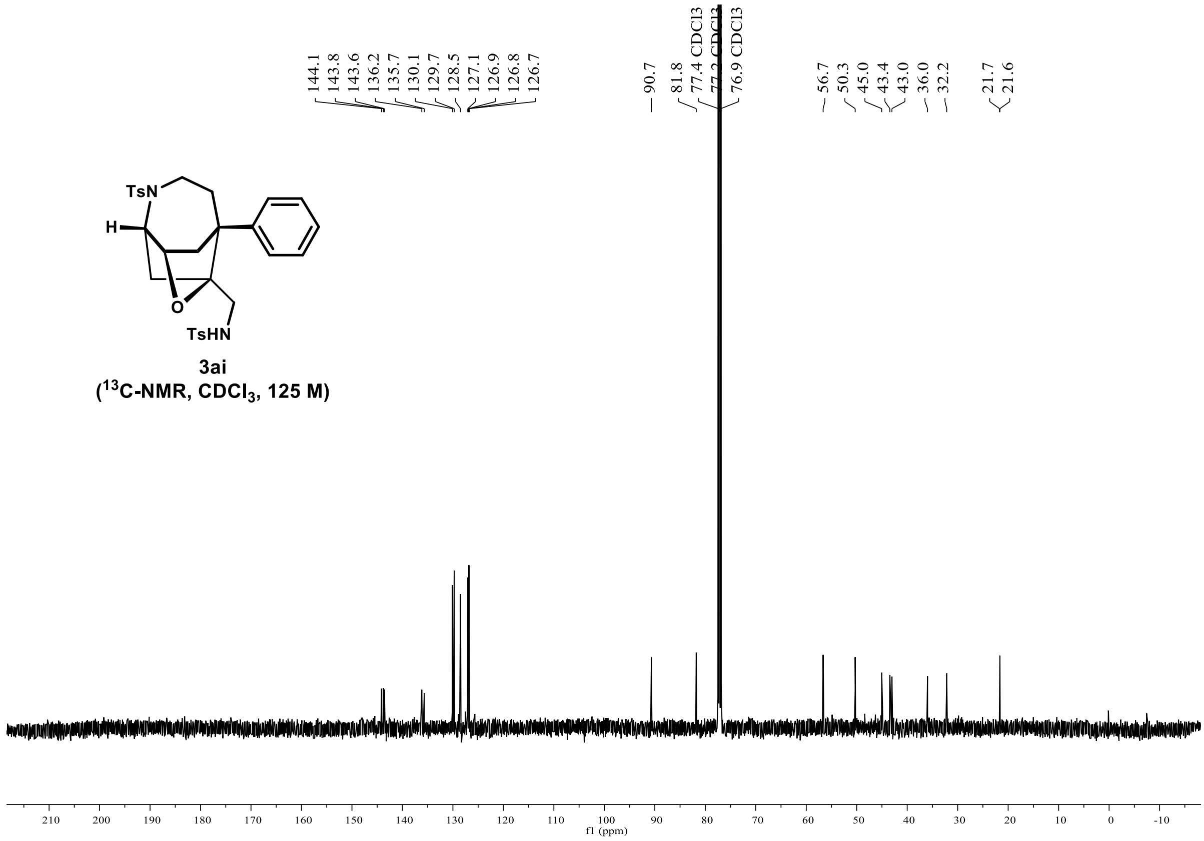

3ai
(^{13}C-NMR, CDCl$_3$, 125 M)

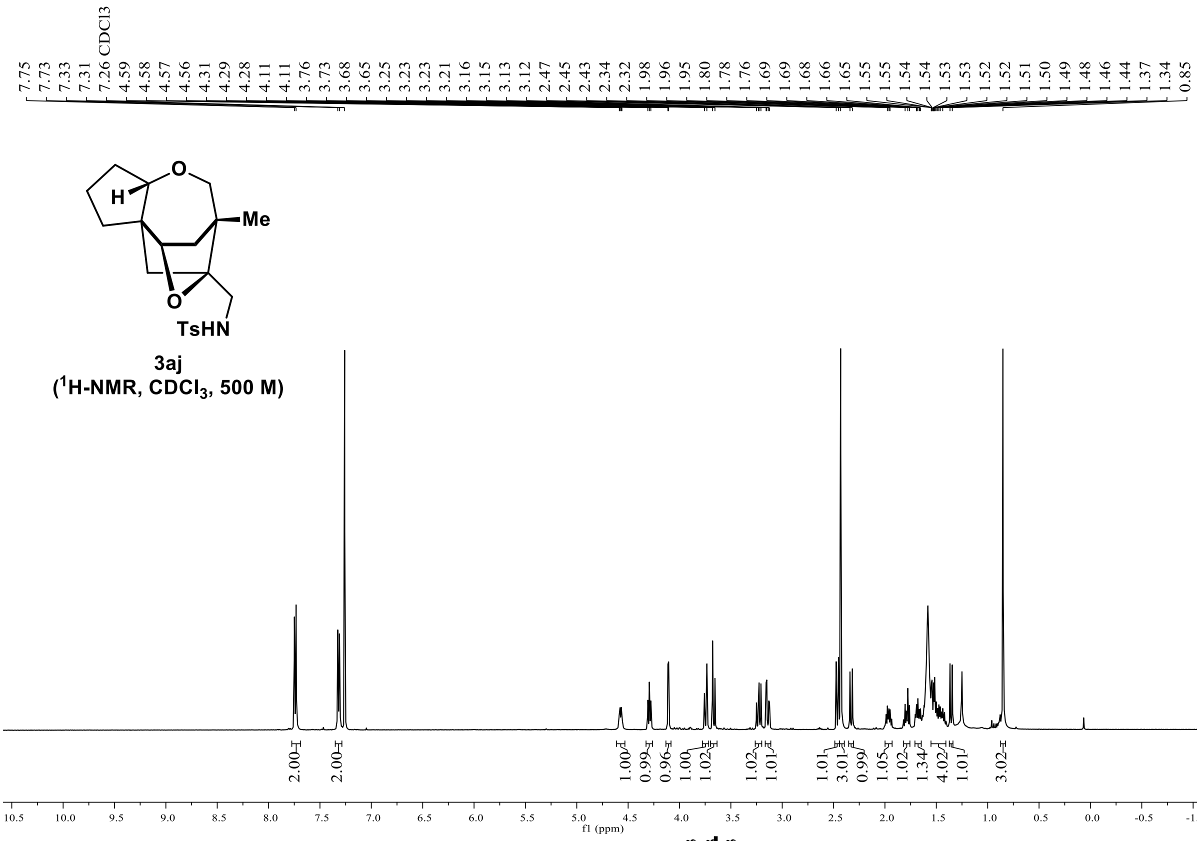

3aj
(^{1}H-NMR, $CDCl_3$, 500 M)

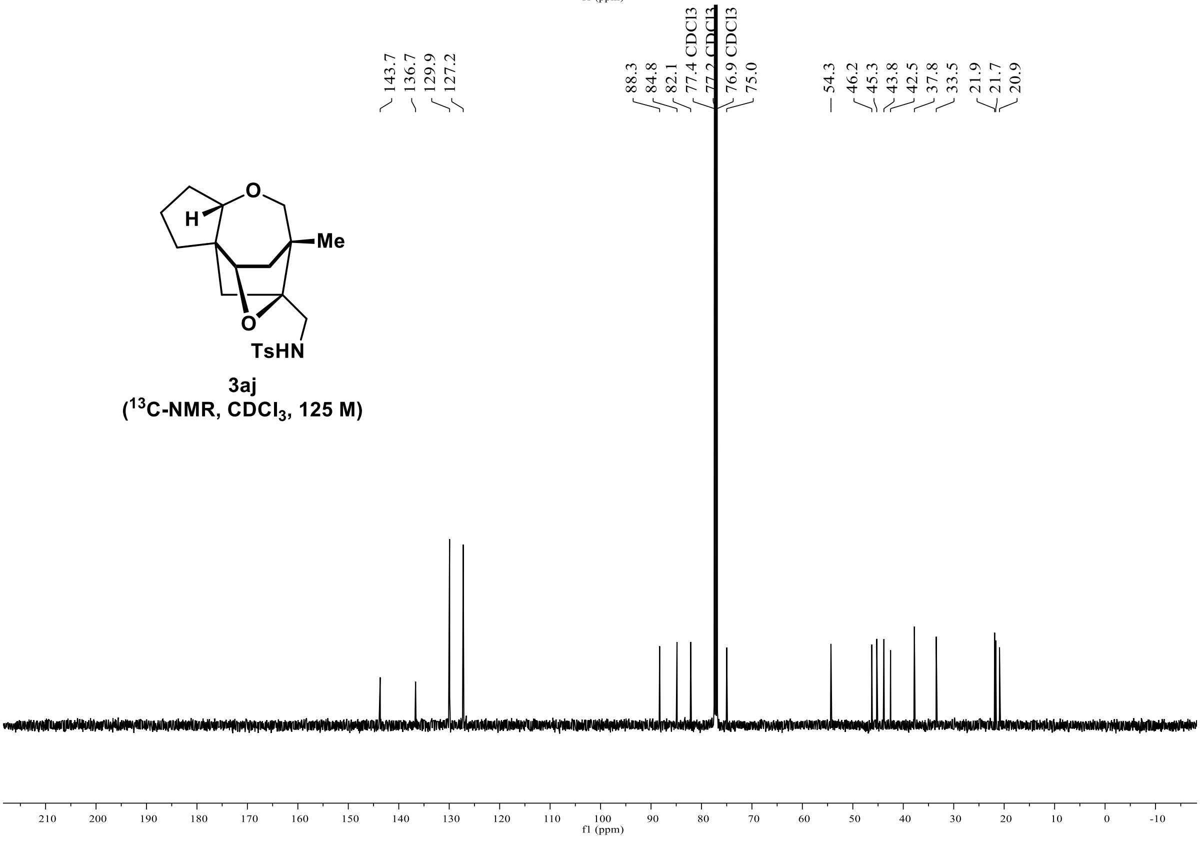

3aj
(^{13}C-NMR, $CDCl_3$, 125 M)

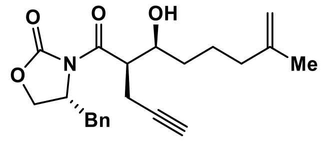

S29
(^{1}H-NMR, $CDCl_3$, 500 M)

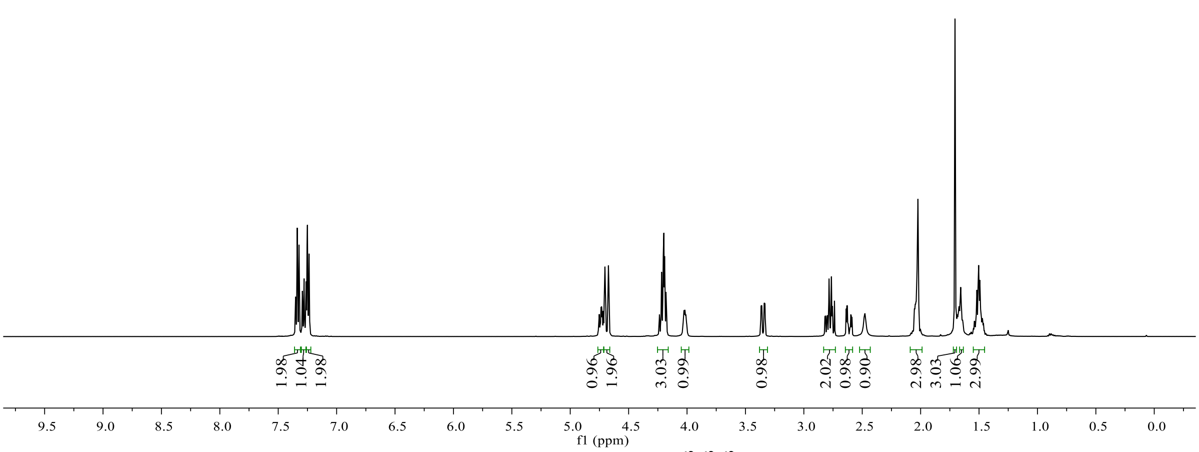

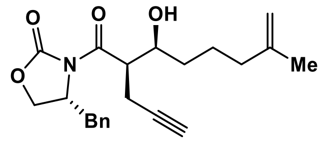

S29
(^{13}C-NMR, $CDCl_3$, 125 M)

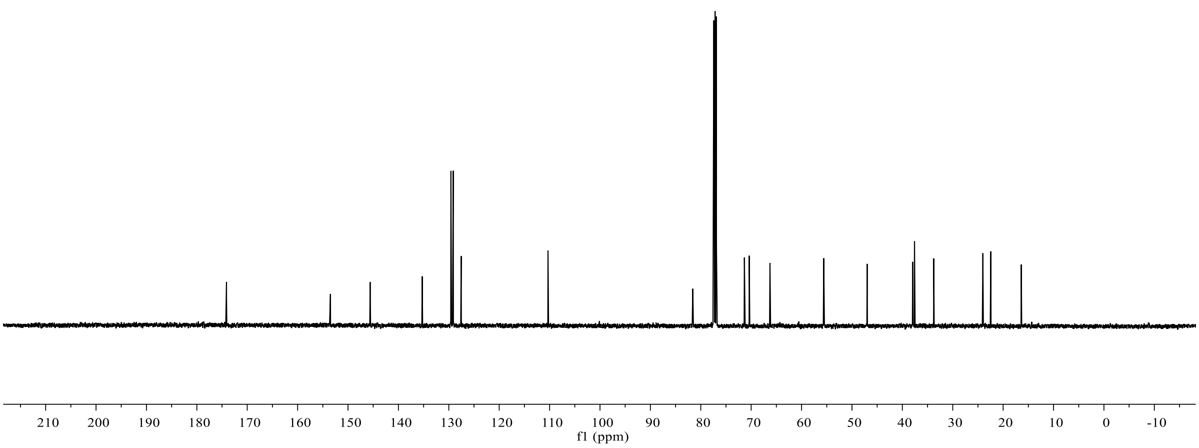

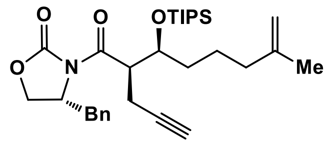

6
(^{1}H-NMR, CDCl$_3$, 500 M)

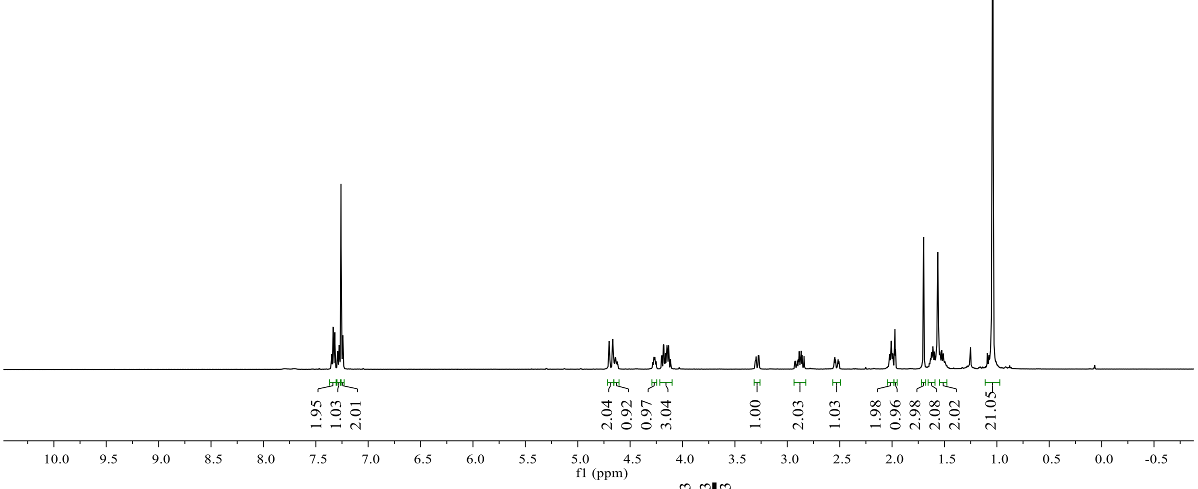

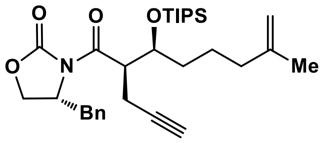

6
(^{13}C-NMR, CDCl$_3$, 125 M)

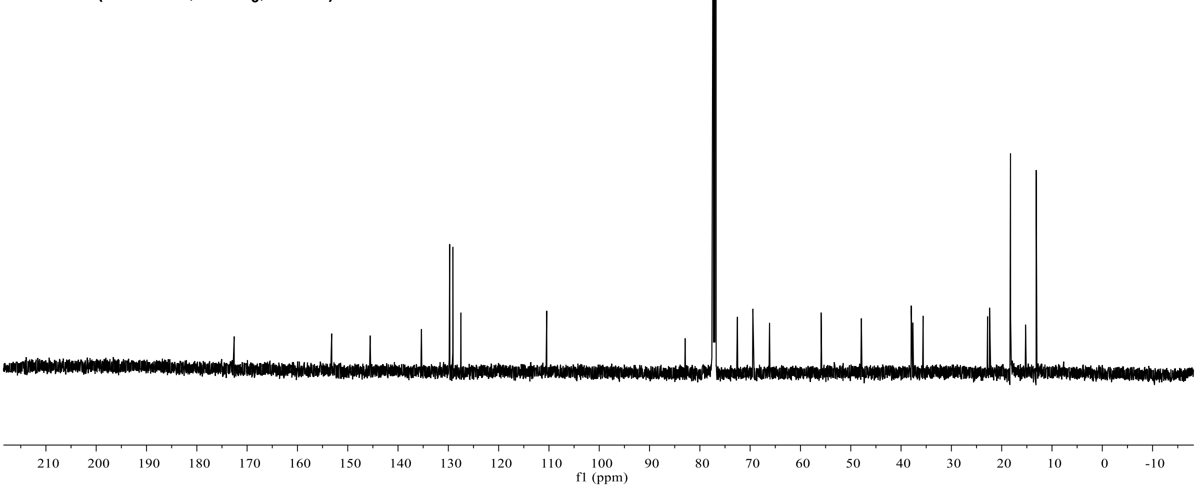

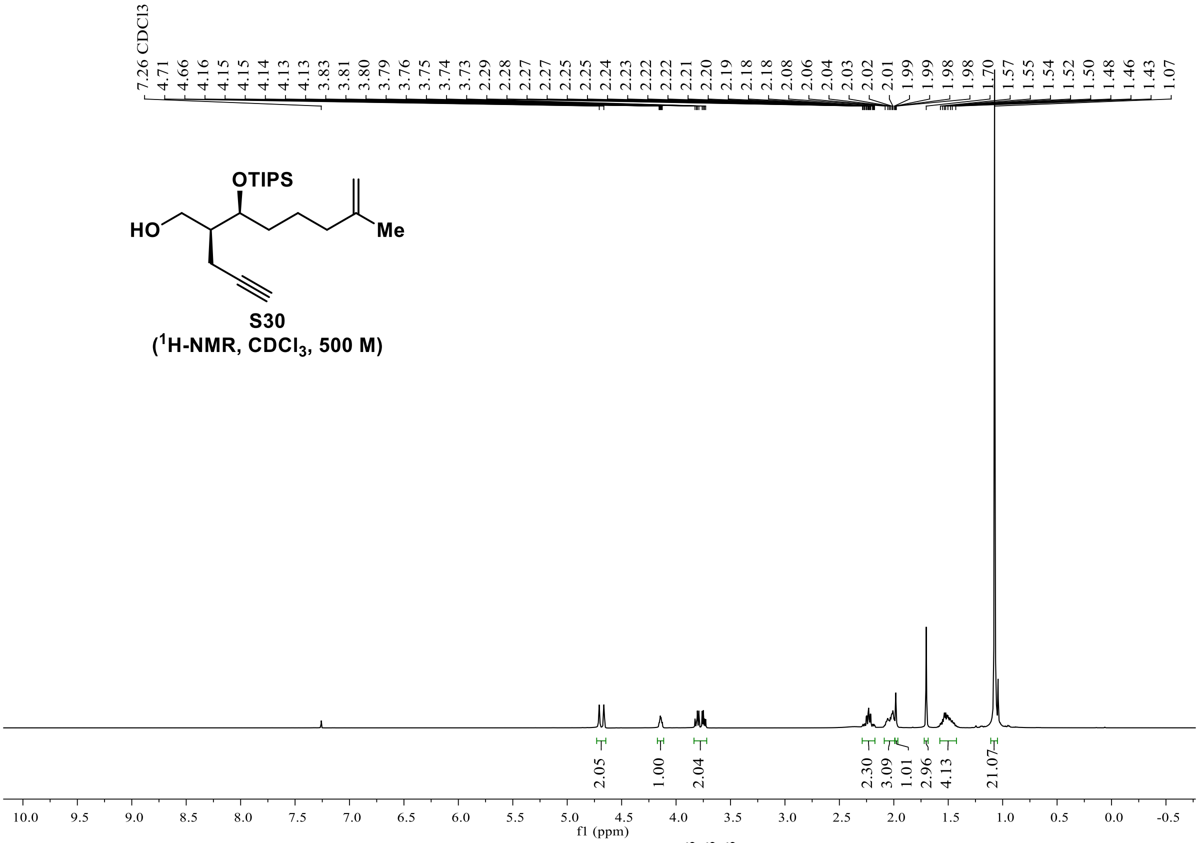
7.26 CDCl3
4.71 4.66 4.16 4.15 4.15 4.14 4.13 4.13 3.83 3.81 3.80 3.79 3.76 3.75 3.74 3.73 2.29 2.28 2.27 2.27 2.25 2.25 2.24 2.23 2.22 2.22 2.21 2.20 2.19 2.18 2.18 2.08 2.06 2.04 2.03 2.02 2.01 1.99 1.99 1.98 1.98 1.70 1.57 1.55 1.54 1.52 1.50 1.48 1.46 1.43 1.07
OTIPS
HO
Me
S30
(1H-NMR, CDCl3, 500 M)
2.05 1.00 2.04 2.30 3.09 1.01 2.96 4.13 21.07
10.0 9.5 9.0 8.5 8.0 7.5 7.0 6.5 6.0 5.5 5.0 4.5 4.0 3.5 3.0 2.5 2.0 1.5 1.0 0.5 0.0 -0.5
f1 (ppm)

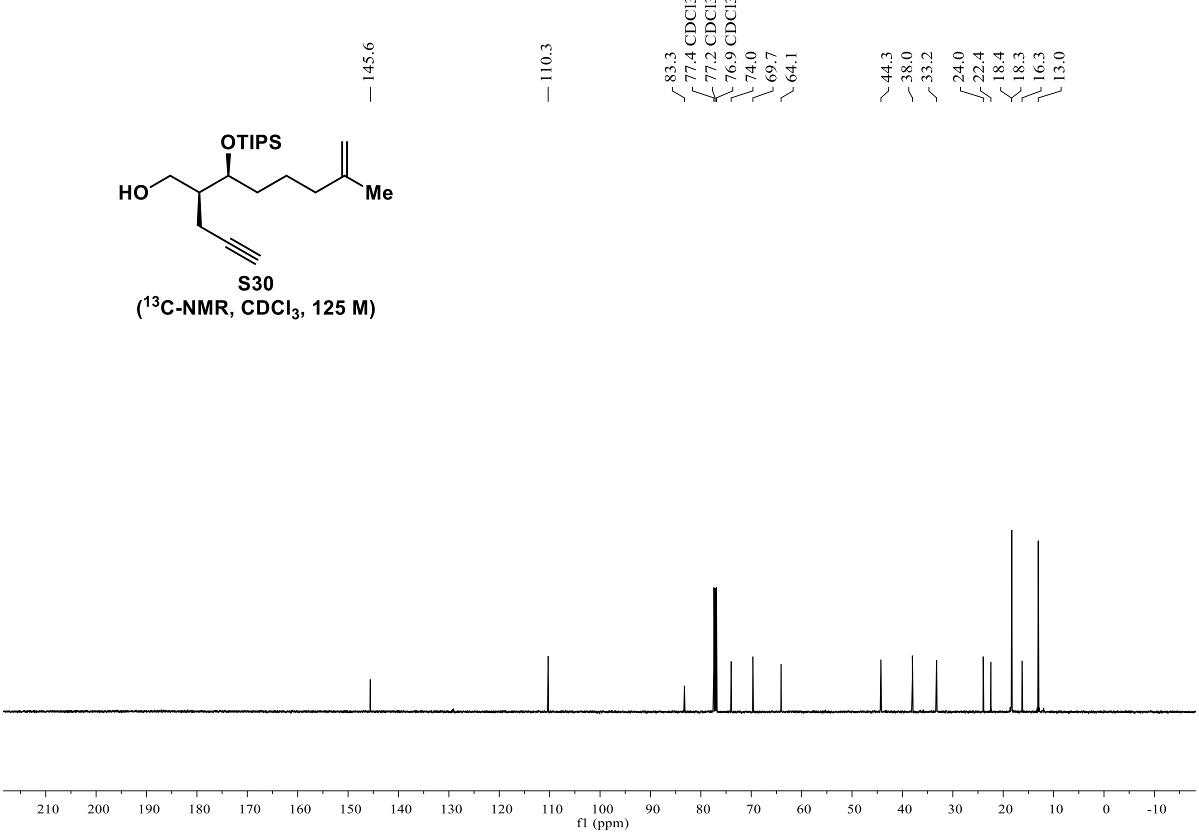
145.6
110.3
83.3 77.4 CDCl3 77.2 CDCl3 76.9 CDCl3 74.0 69.7 64.1
44.3 38.0 33.2 24.0 22.4 18.4 18.3 16.3 13.0
OTIPS
HO
Me
S30
(13C-NMR, CDCl3, 125 M)
210 200 190 180 170 160 150 140 130 120 110 100 90 80 70 60 50 40 30 20 10 0 -10
f1 (ppm)

7
(^{1}H-NMR, $CDCl_3$, 500 M)

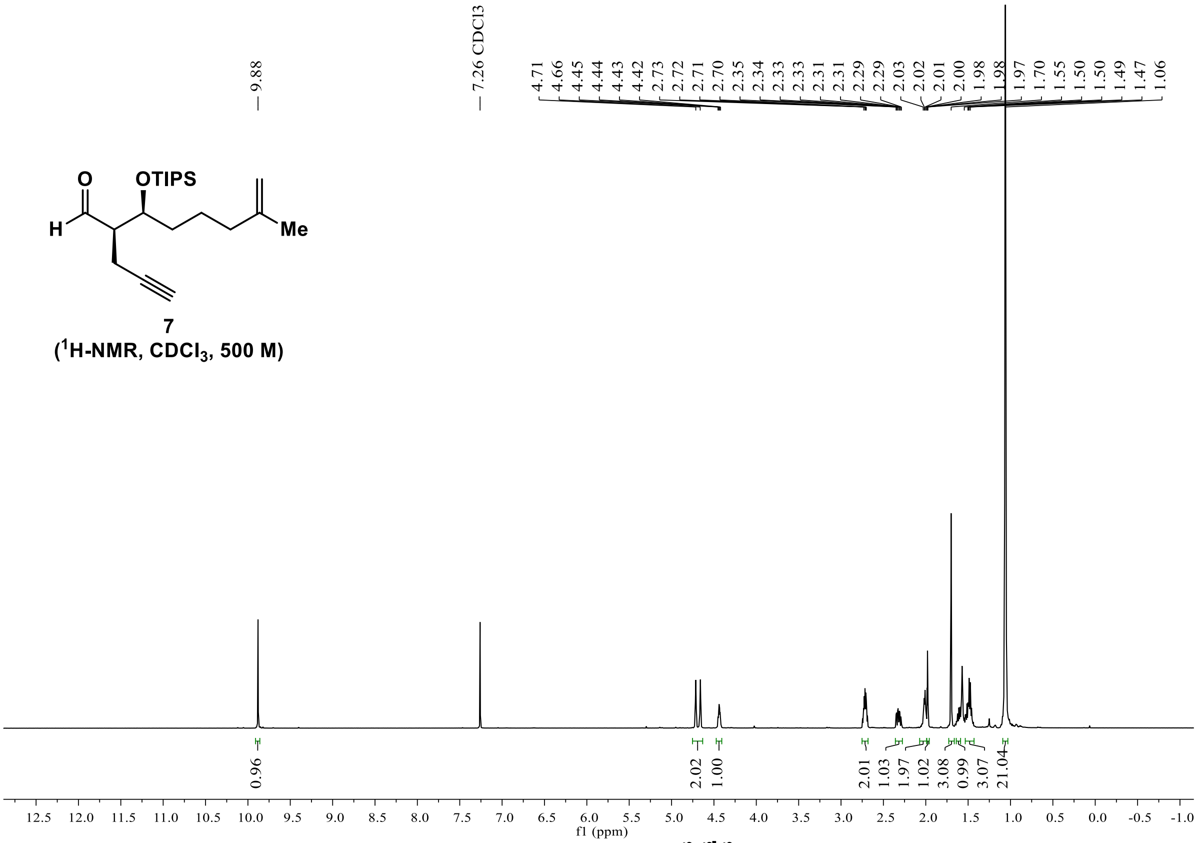

7
(^{13}C-NMR, $CDCl_3$, 125 M)

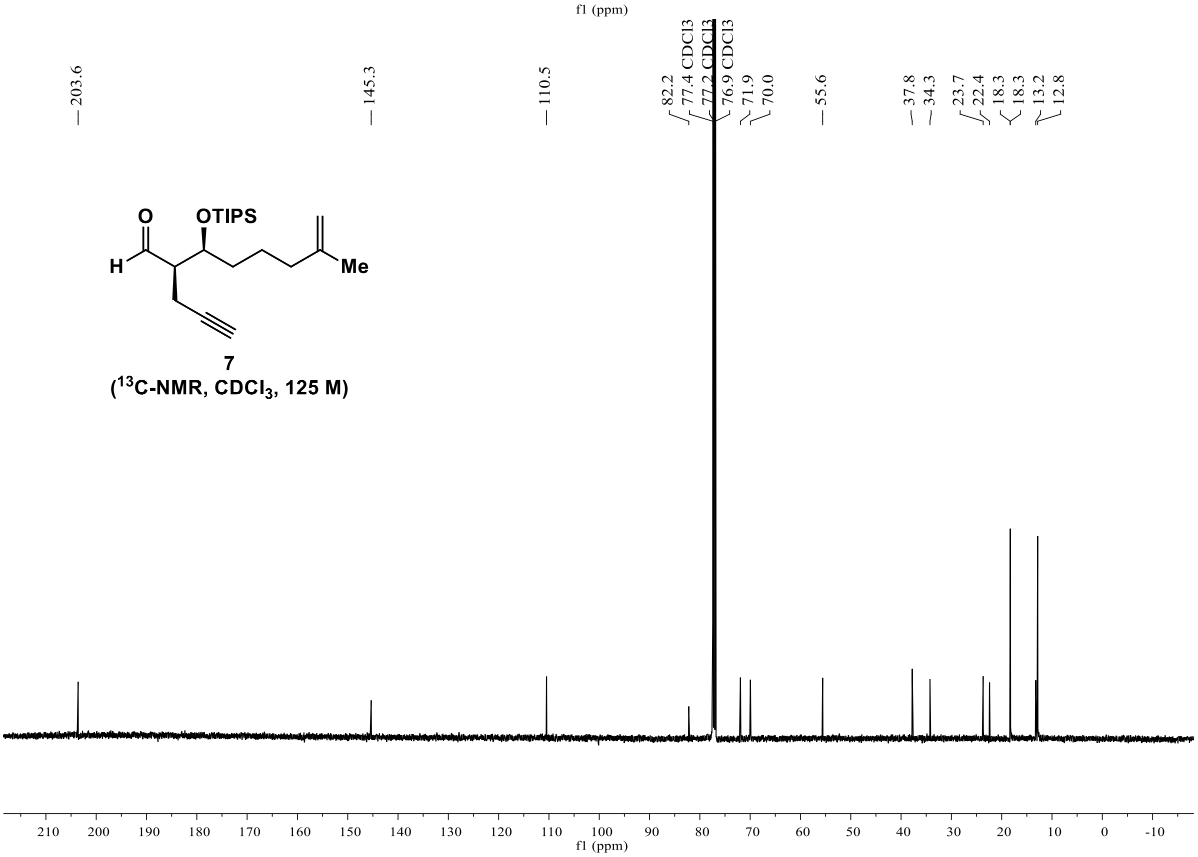

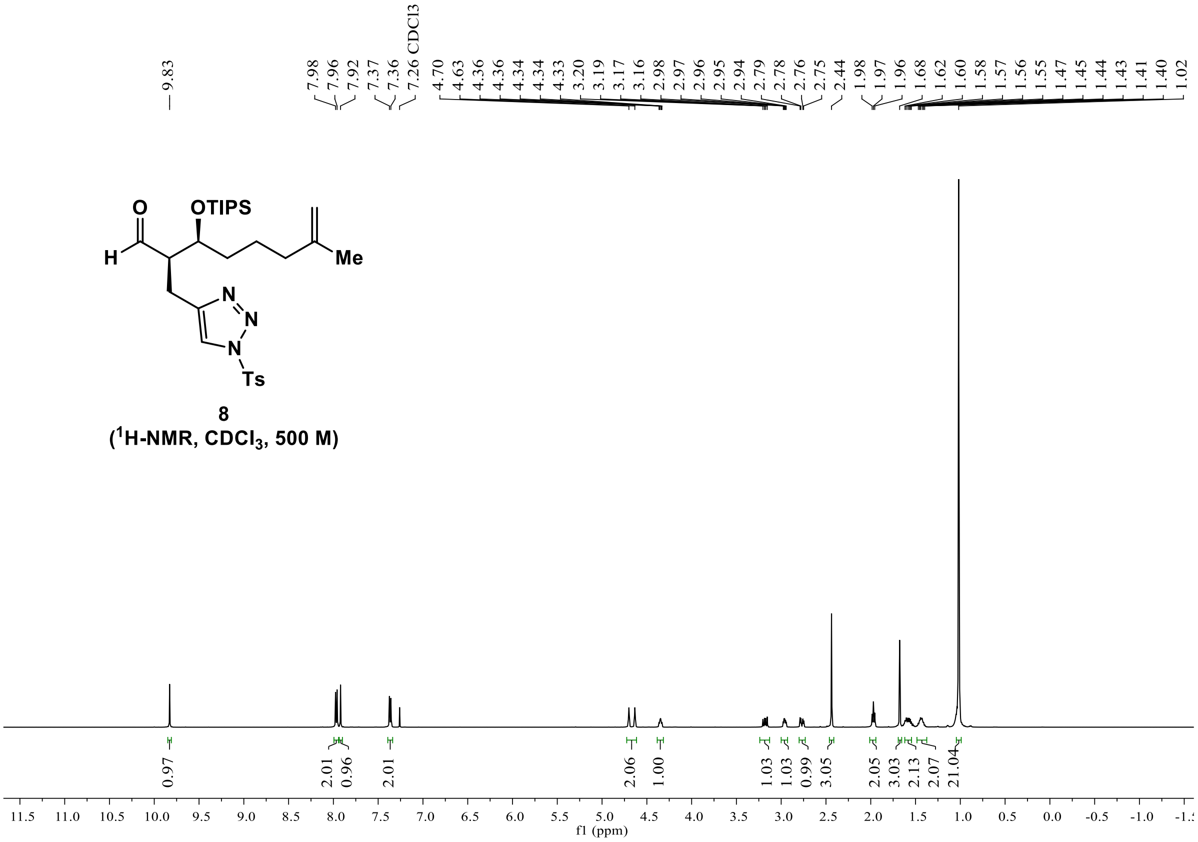

8
(^{1}H-NMR, $CDCl_3$, 500 M)

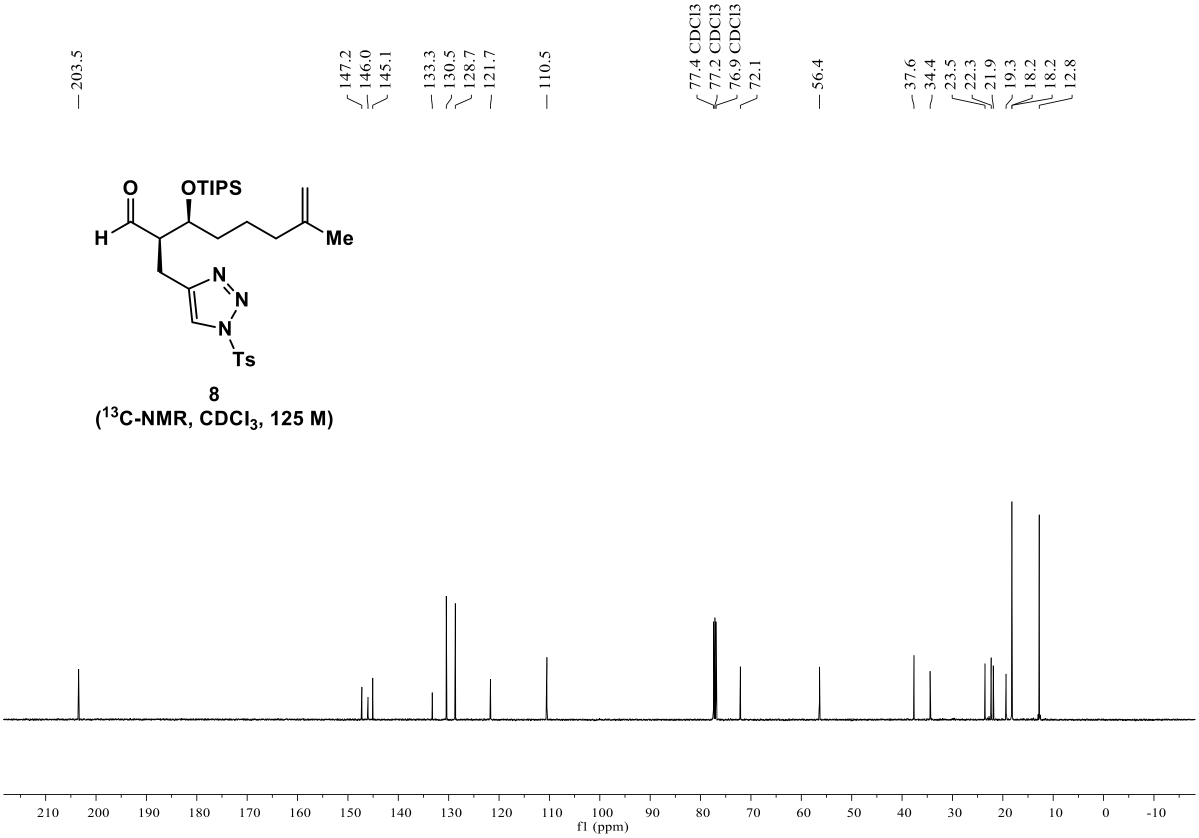

8
(^{13}C-NMR, $CDCl_3$, 125 M)

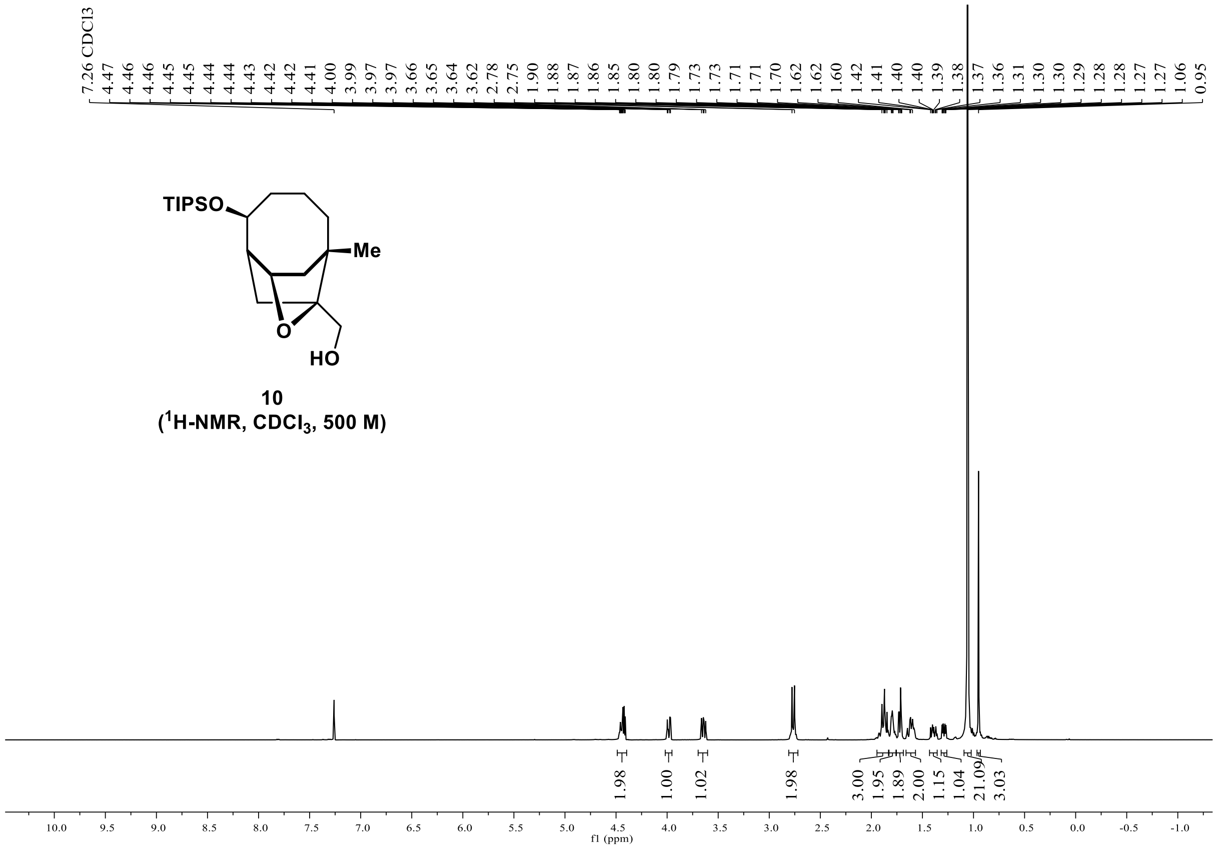

10
(^{1}H-NMR, $CDCl_3$, 500 M)

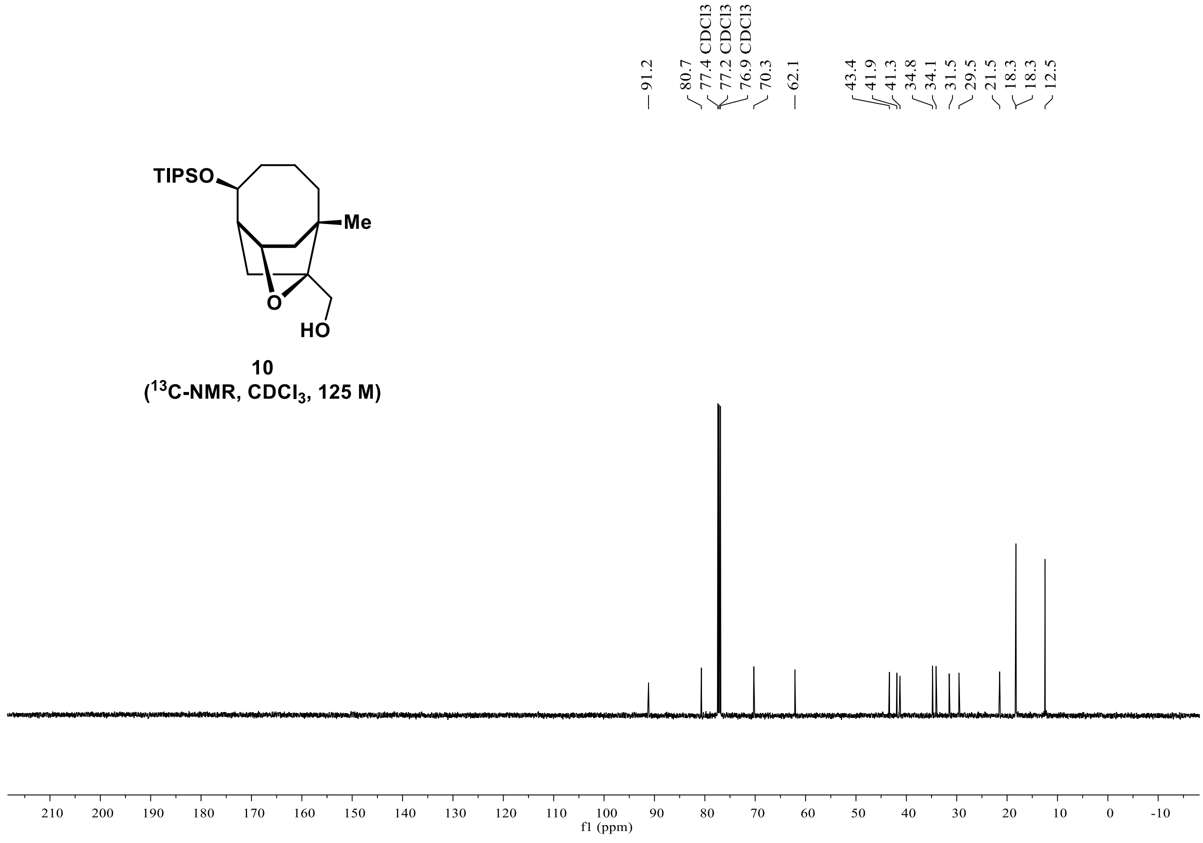

10
(^{13}C-NMR, $CDCl_3$, 125 M)

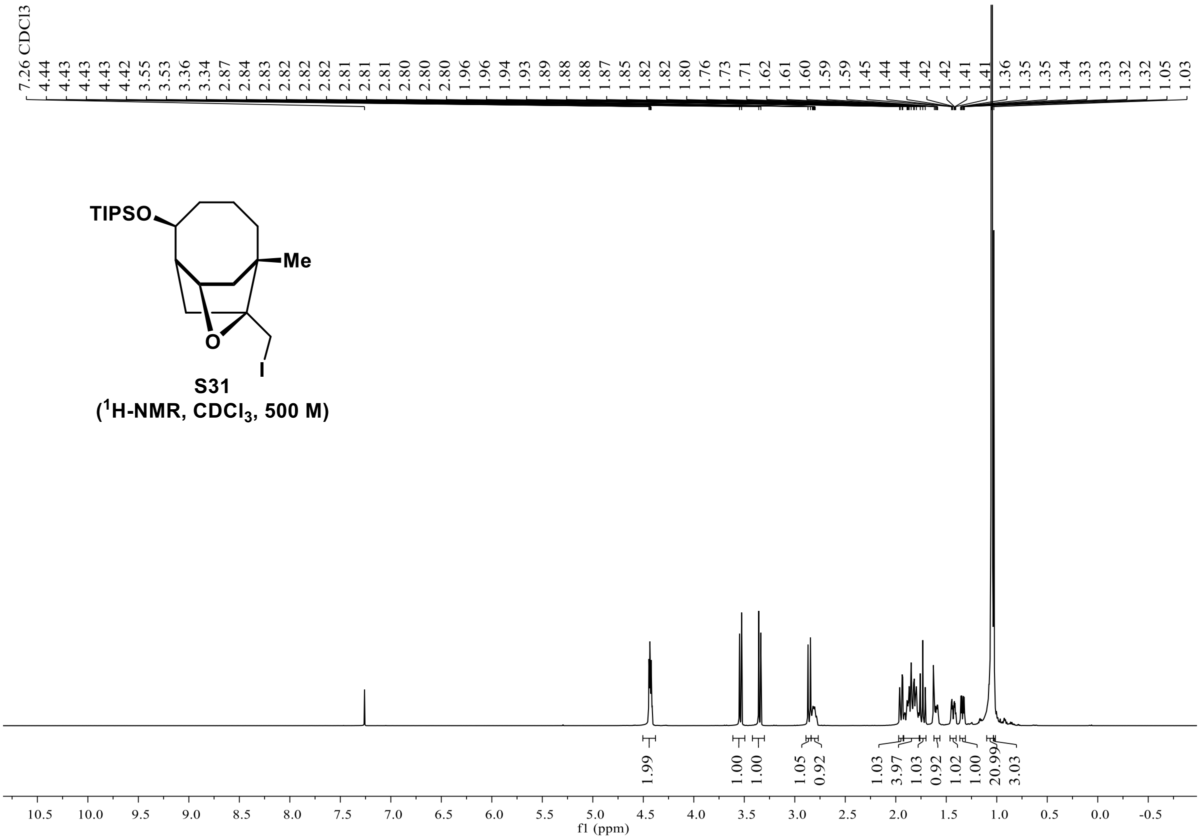
7.26 CDCl3
4.44
4.43
4.43
4.43
4.42
3.55
3.53
3.36
3.34
2.87
2.84
2.83
2.82
2.82
2.82
2.81
2.81
2.81
2.80
2.80
2.80
1.96
1.96
1.94
1.93
1.89
1.88
1.88
1.87
1.85
1.82
1.82
1.80
1.76
1.73
1.71
1.62
1.61
1.60
1.59
1.59
1.45
1.44
1.44
1.42
1.42
1.41
1.41
1.36
1.35
1.35
1.34
1.33
1.33
1.32
1.32
1.05
1.03
TIPSO
Me
O
I
S31
(^{1}H-NMR, CDCl$_3$, 500 M)
1.99
1.00
1.00
1.05
0.92
1.03
3.97
1.03
0.92
1.02
1.00
20.99
3.03
10.5 10.0 9.5 9.0 8.5 8.0 7.5 7.0 6.5 6.0 5.5 5.0 4.5 4.0 3.5 3.0 2.5 2.0 1.5 1.0 0.5 0.0 -0.5
f1 (ppm)

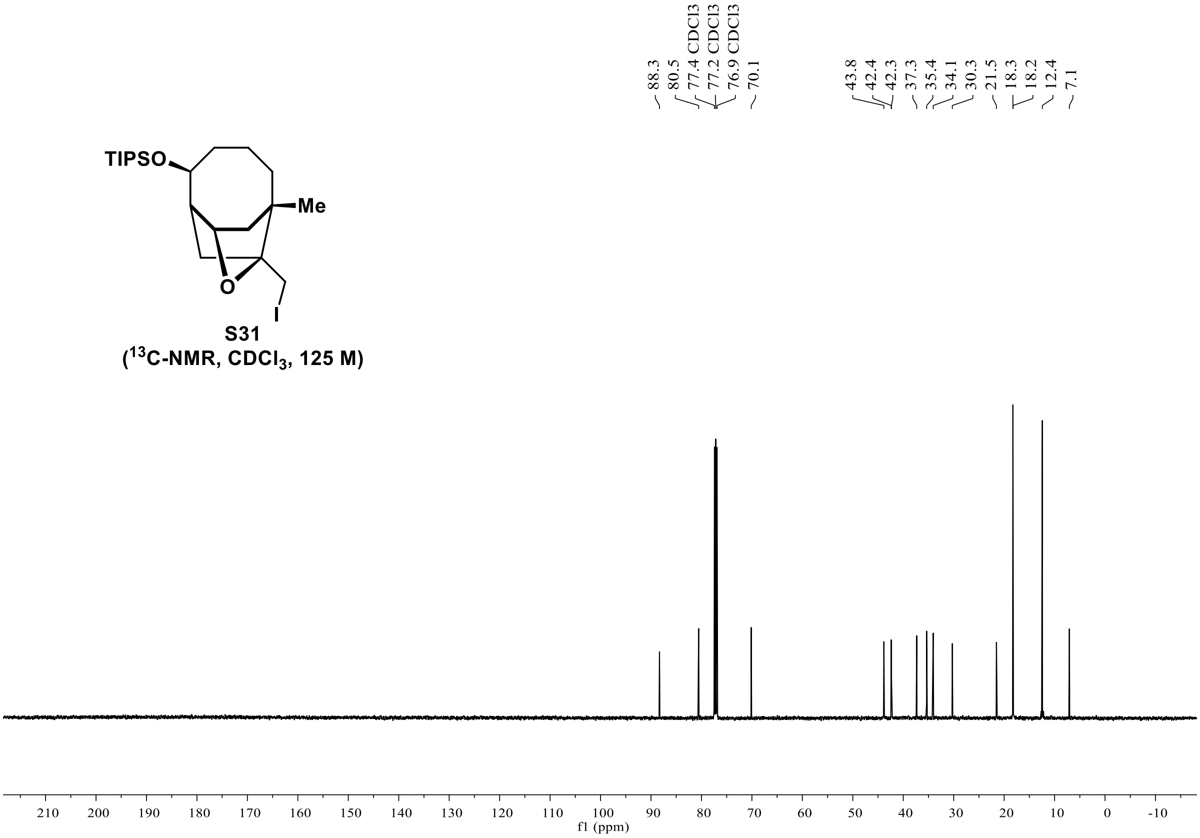
88.3
80.5
77.4 CDCl3
77.2 CDCl3
76.9 CDCl3
70.1
43.8
42.4
42.3
37.3
35.4
34.1
30.3
21.5
18.3
18.2
12.4
7.1
TIPSO
Me
O
I
S31
(^{13}C-NMR, CDCl$_3$, 125 M)
210 200 190 180 170 160 150 140 130 120 110 100 90 80 70 60 50 40 30 20 10 0 -10
f1 (ppm)

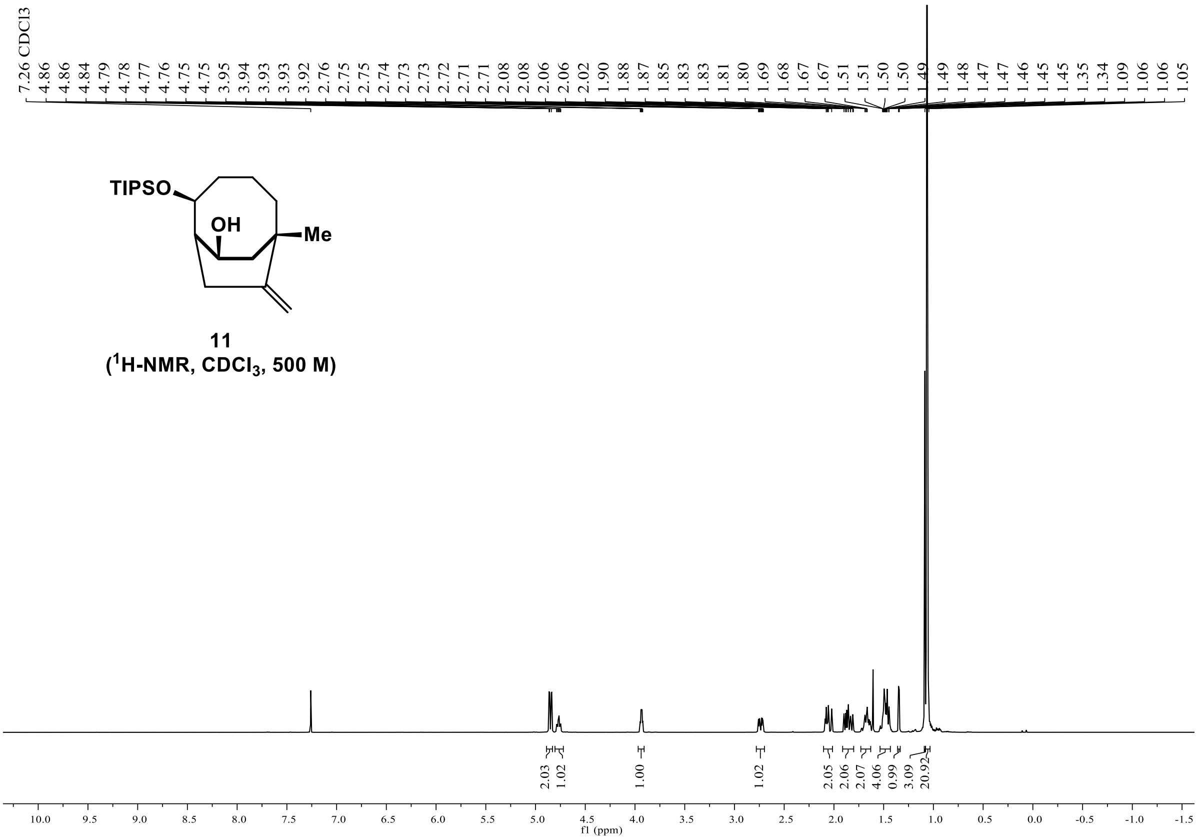

11
(^{1}H-NMR, $CDCl_3$, 500 M)

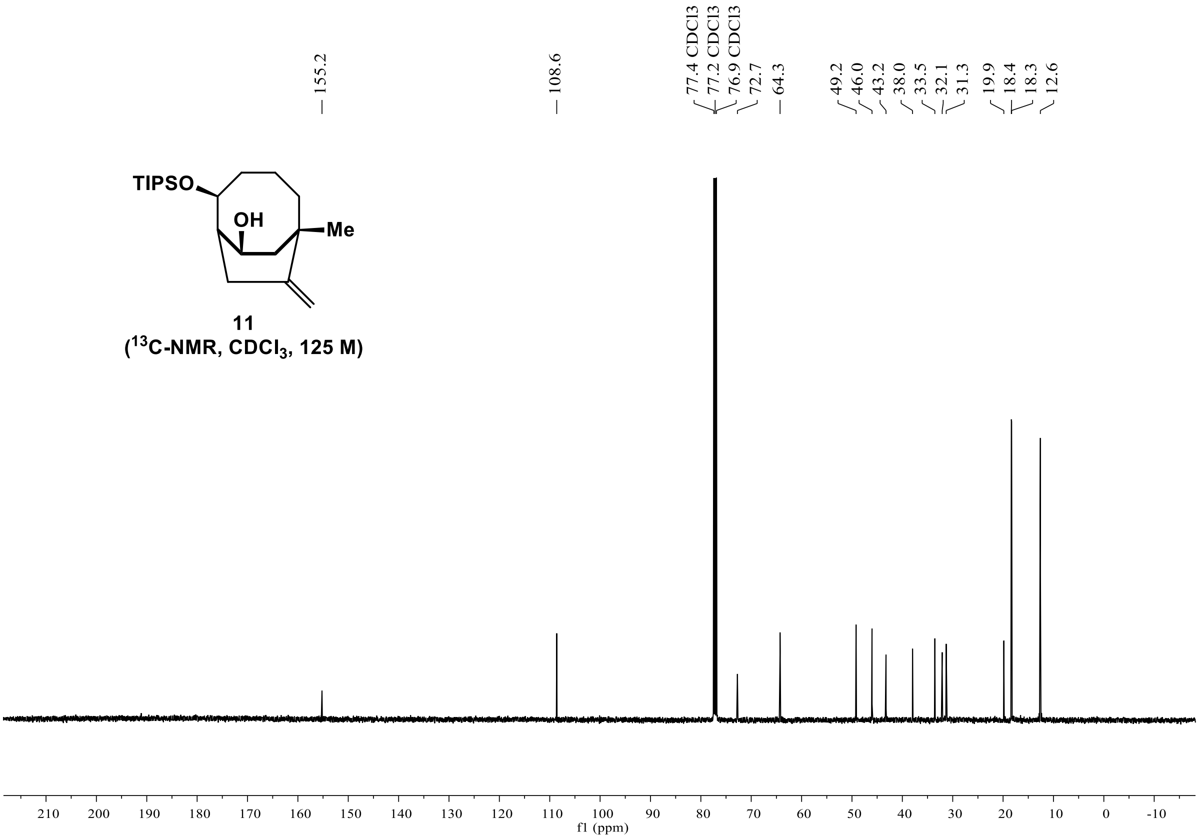

11
(^{13}C-NMR, $CDCl_3$, 125 M)

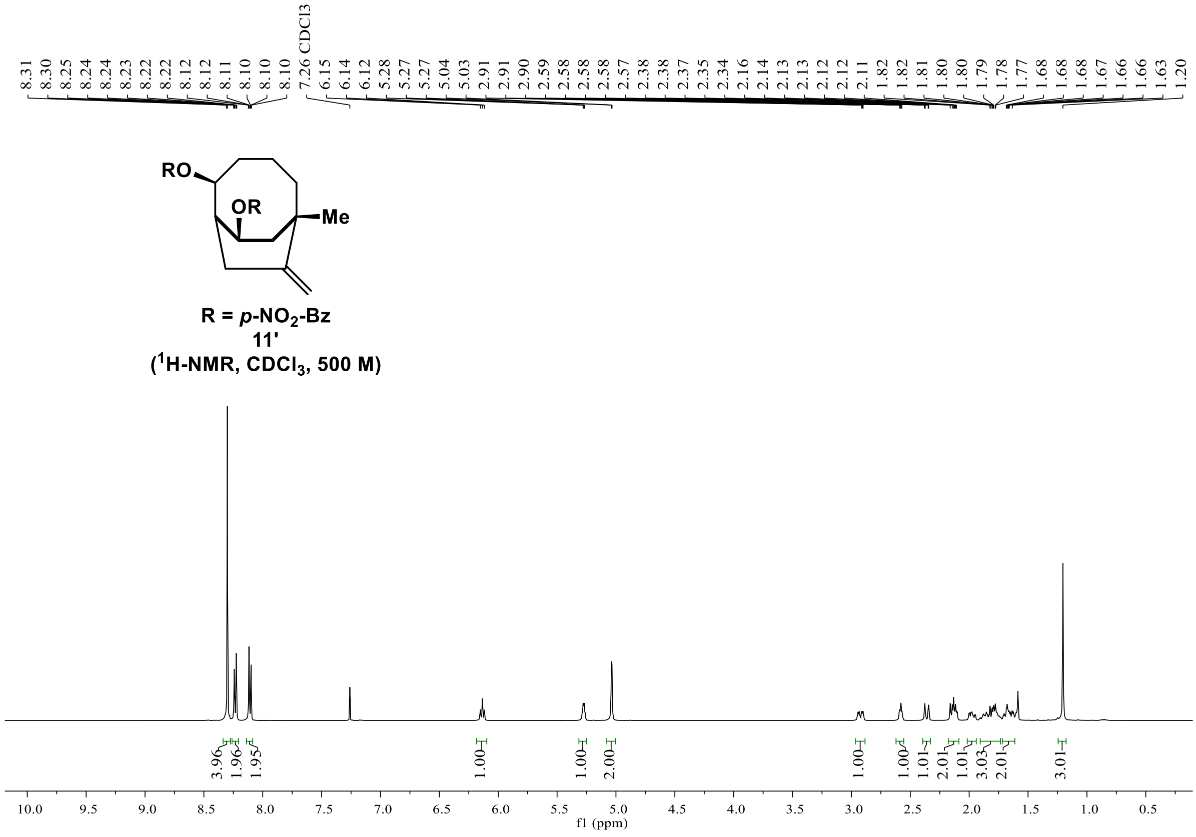
8.31 8.30 8.25 8.24 8.24 8.23 8.22 8.22 8.12 8.12 8.11 8.10 8.10 8.10 7.26 CDCl3 6.15 6.14 6.12 5.28 5.27 5.27 5.04 5.03 2.91 2.91 2.90 2.59 2.58 2.58 2.58 2.57 2.38 2.38 2.37 2.35 2.34 2.16 2.14 2.13 2.13 2.12 2.12 2.11 1.82 1.82 1.81 1.80 1.80 1.79 1.78 1.77 1.68 1.68 1.68 1.67 1.66 1.63 1.20
RO
OR
Me
R = p-NO2-Bz
11'
(1H-NMR, CDCl3, 500 M)
3.96 1.96 1.95 1.00 1.00 2.00 1.00 1.00 1.01 2.01 1.01 3.03 2.01 3.01
10.0 9.5 9.0 8.5 8.0 7.5 7.0 6.5 6.0 5.5 5.0 4.5 4.0 3.5 3.0 2.5 2.0 1.5 1.0 0.5
f1 (ppm)

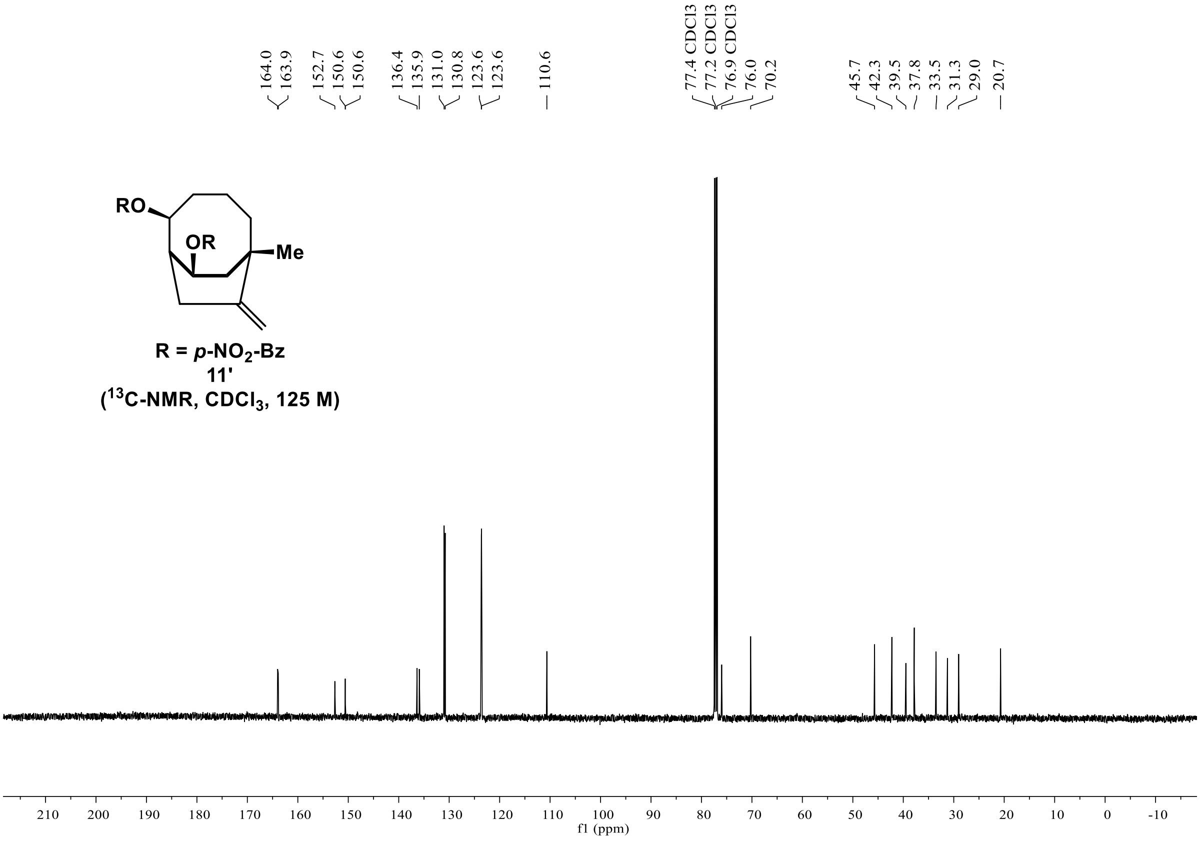
164.0 163.9 152.7 150.6 150.6 136.4 135.9 131.0 130.8 123.6 123.6 110.6 77.4 CDCl3 77.2 CDCl3 76.9 CDCl3 76.0 70.2 45.7 42.3 39.5 37.8 33.5 31.3 29.0 20.7
RO
OR
Me
R = p-NO2-Bz
11'
(13C-NMR, CDCl3, 125 M)
210 200 190 180 170 160 150 140 130 120 110 100 90 80 70 60 50 40 30 20 10 0 -10
f1 (ppm)

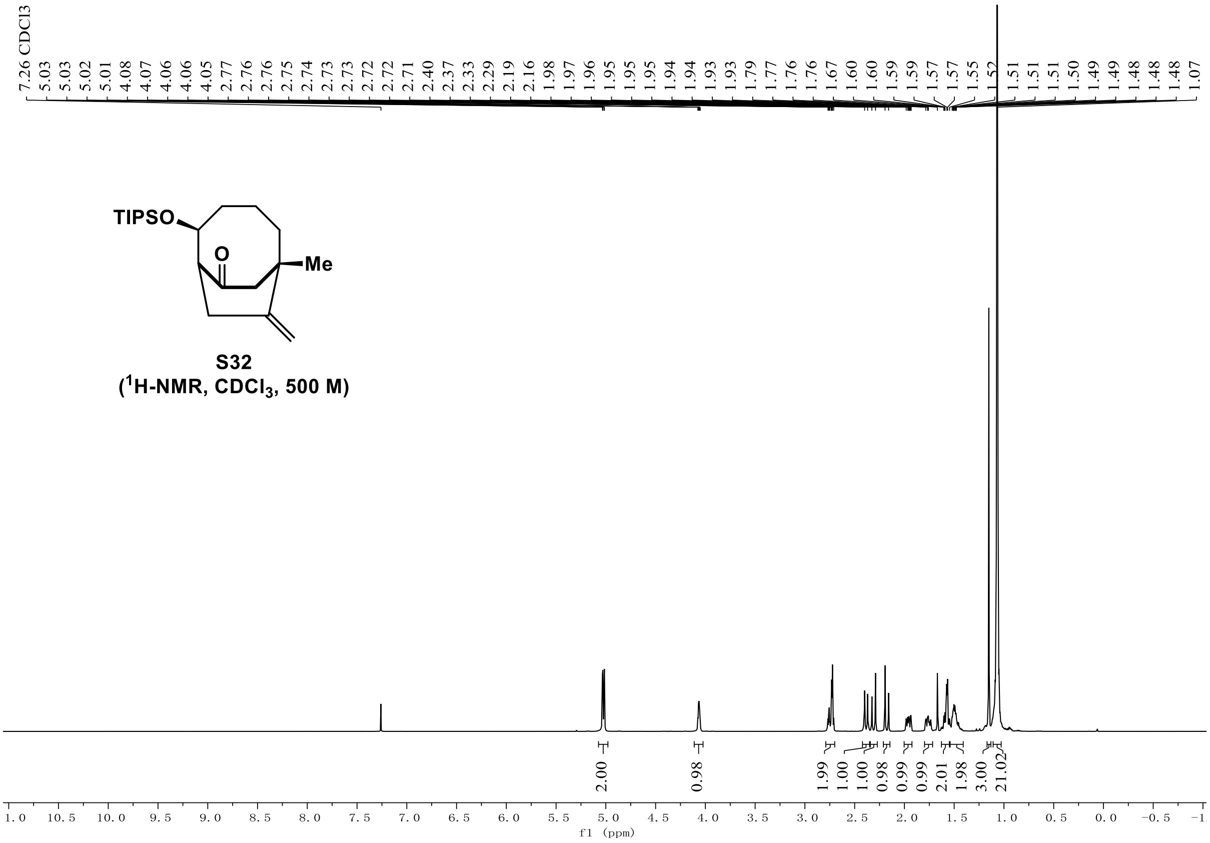
7.26 CDCl3
5.03
5.03
5.02
5.01
4.08
4.07
4.06
4.06
4.05
2.77
2.76
2.76
2.75
2.74
2.73
2.73
2.72
2.72
2.71
2.40
2.37
2.33
2.29
2.19
2.16
1.98
1.97
1.96
1.95
1.95
1.95
1.94
1.94
1.93
1.93
1.79
1.77
1.76
1.76
1.67
1.60
1.60
1.59
1.59
1.57
1.57
1.55
1.52
1.51
1.51
1.51
1.50
1.49
1.49
1.48
1.48
1.48
1.07
TIPSO
O
Me
S32
(1H-NMR, CDCl3, 500 M)
2.00
0.98
1.99
1.00
1.00
0.98
0.99
0.99
2.01
1.98
3.00
21.02
f1 (ppm)

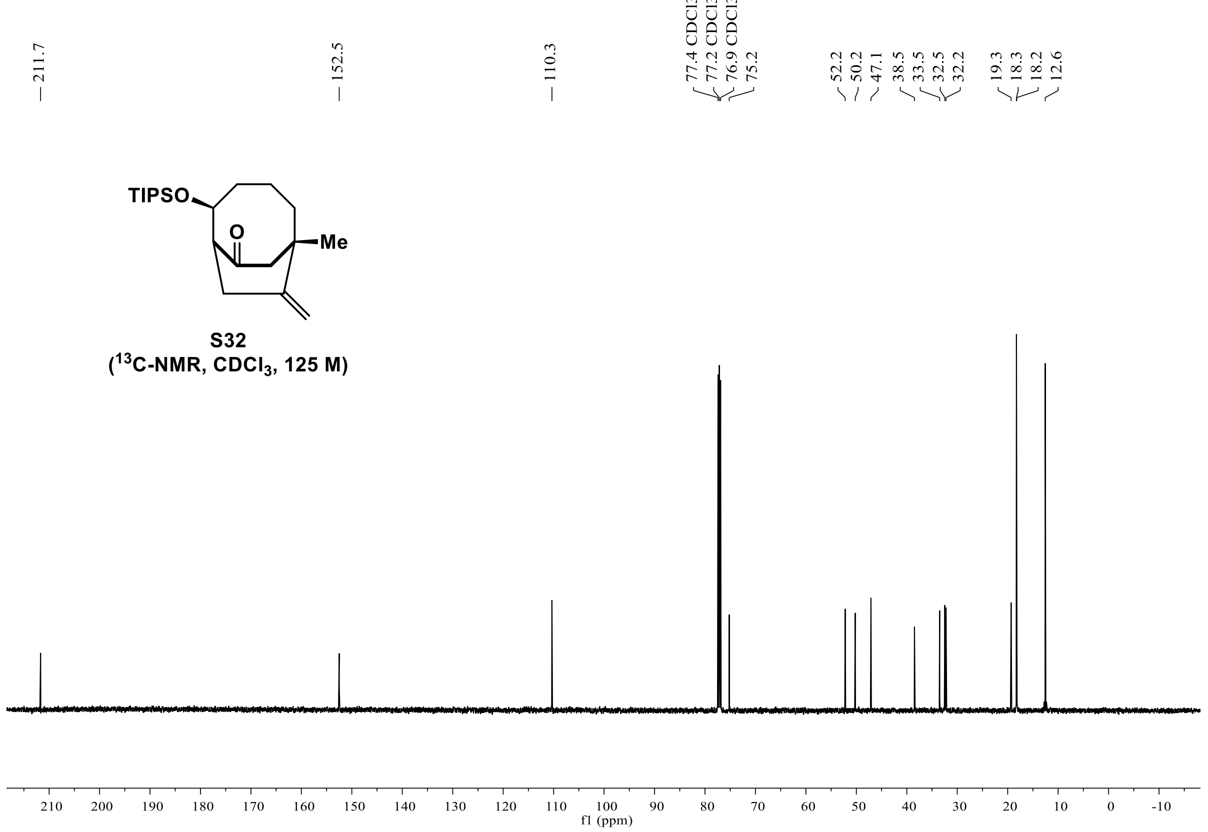
211.7
152.5
110.3
77.4 CDCl3
77.2 CDCl3
76.9 CDCl3
75.2
52.2
50.2
47.1
38.5
33.5
32.5
32.2
19.3
18.3
18.2
12.6
TIPSO
O
Me
S32
(13C-NMR, CDCl3, 125 M)
f1 (ppm)

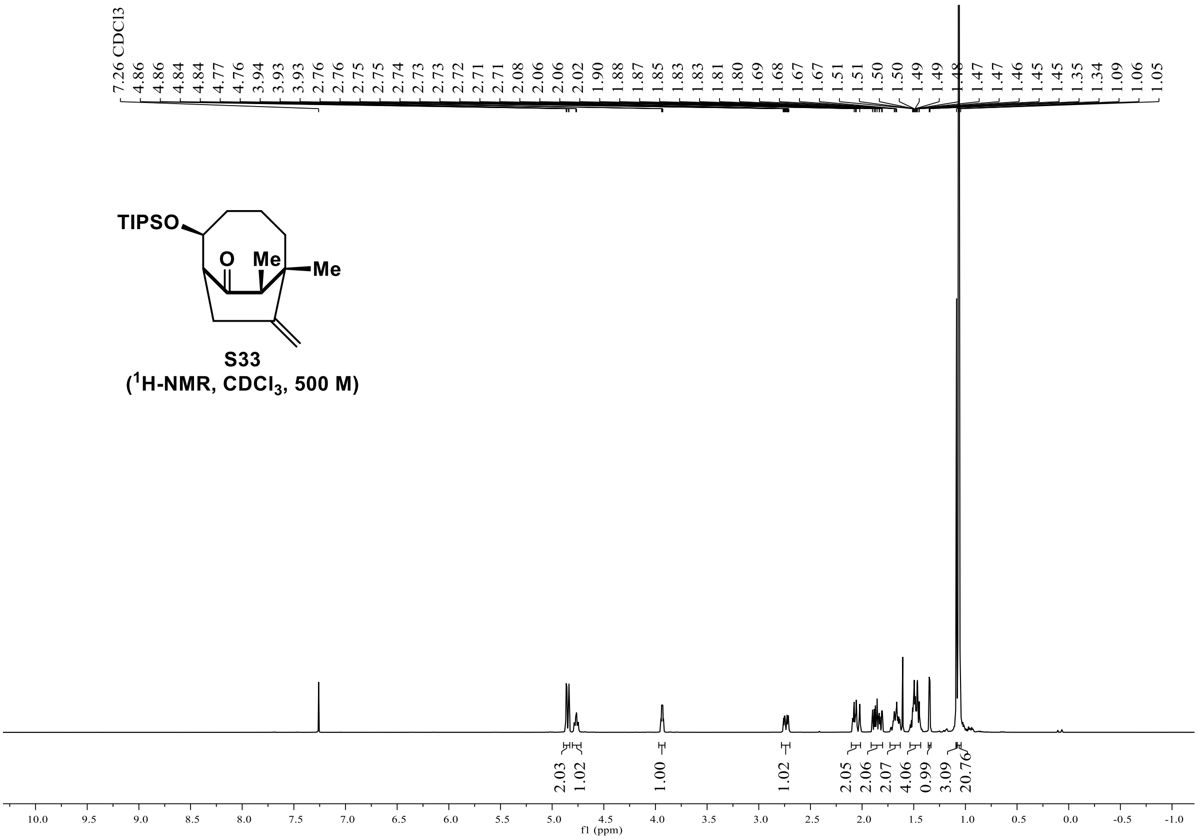
7.26 CDCl3
4.86
4.86
4.84
4.84
4.77
4.76
3.94
3.93
3.93
2.76
2.76
2.75
2.75
2.74
2.73
2.73
2.72
2.71
2.71
2.08
2.06
2.06
2.02
1.90
1.88
1.87
1.85
1.83
1.83
1.81
1.80
1.69
1.68
1.67
1.67
1.51
1.51
1.50
1.50
1.49
1.49
1.48
1.47
1.47
1.46
1.45
1.45
1.35
1.34
1.09
1.06
1.05
TIPSO
O
Me
Me
S33
(1H-NMR, CDCl3, 500 M)
2.03
1.02
1.00
1.02
2.05
2.06
2.07
4.06
0.99
3.09
20.76
10.0 9.5 9.0 8.5 8.0 7.5 7.0 6.5 6.0 5.5 5.0 4.5 4.0 3.5 3.0 2.5 2.0 1.5 1.0 0.5 0.0 -0.5 -1.0
f1 (ppm)

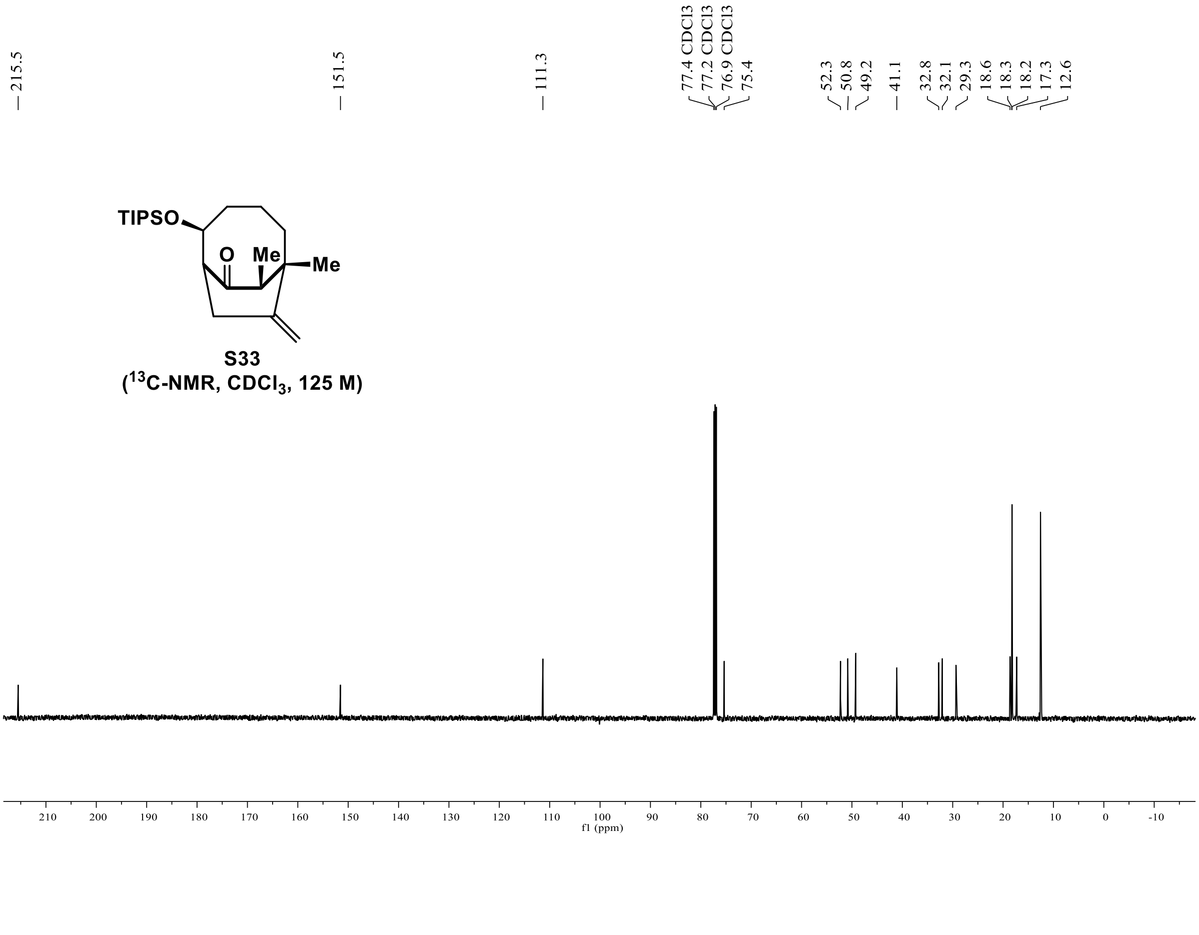
215.5
151.5
111.3
77.4 CDCl3
77.2 CDCl3
76.9 CDCl3
75.4
52.3
50.8
49.2
41.1
32.8
32.1
29.3
18.6
18.3
18.2
17.3
12.6
TIPSO
O
Me
Me
S33
(13C-NMR, CDCl3, 125 M)
210 200 190 180 170 160 150 140 130 120 110 100 90 80 70 60 50 40 30 20 10 0 -10
f1 (ppm)

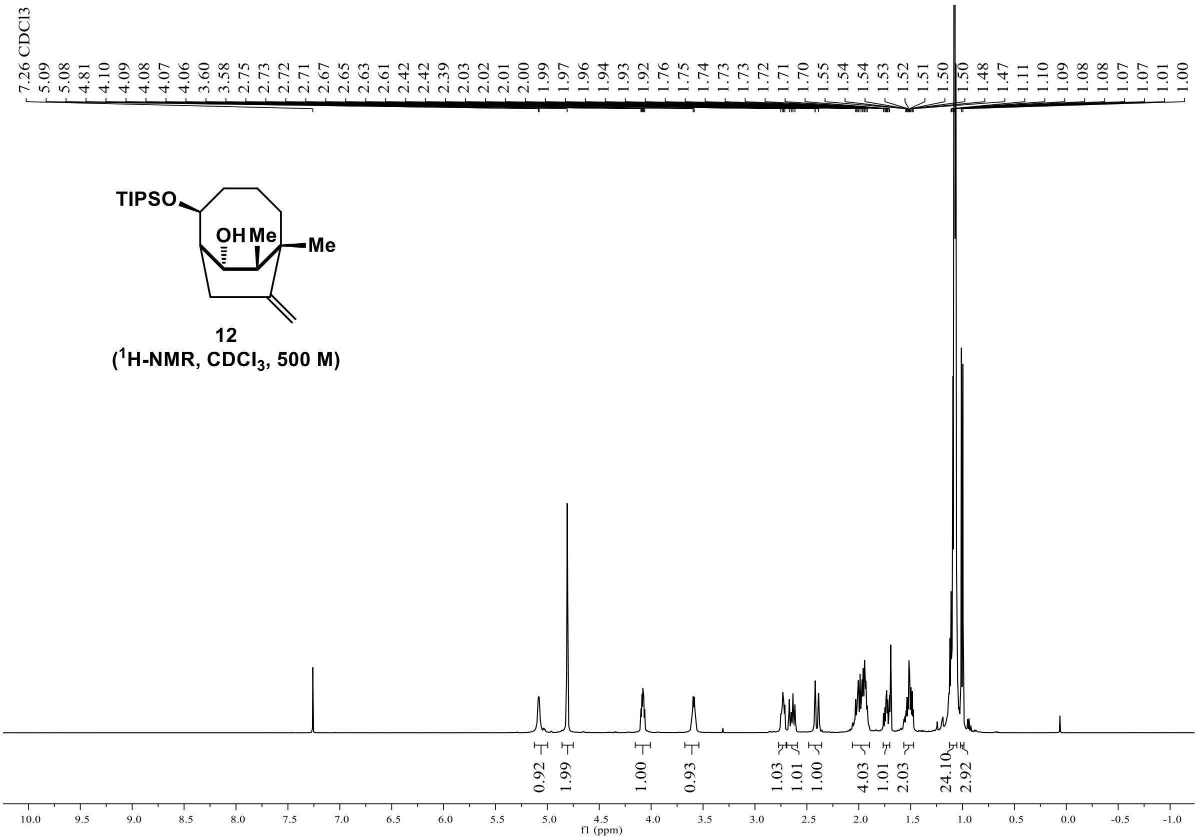
7.26 CDCl3
5.09
5.08
4.81
4.10
4.09
4.08
4.07
4.06
3.60
3.58
2.75
2.73
2.72
2.71
2.67
2.65
2.63
2.61
2.42
2.42
2.39
2.03
2.02
2.01
2.00
1.99
1.97
1.96
1.94
1.93
1.92
1.76
1.75
1.74
1.73
1.73
1.72
1.71
1.70
1.55
1.54
1.54
1.53
1.52
1.51
1.50
1.50
1.48
1.47
1.11
1.10
1.09
1.08
1.08
1.07
1.07
1.01
1.00
TIPSO
OH Me
Me
12
(1H-NMR, CDCl3, 500 M)
0.92
1.99
1.00
0.93
1.03
1.01
1.00
4.03
1.01
2.03
24.10
2.92
f1 (ppm)

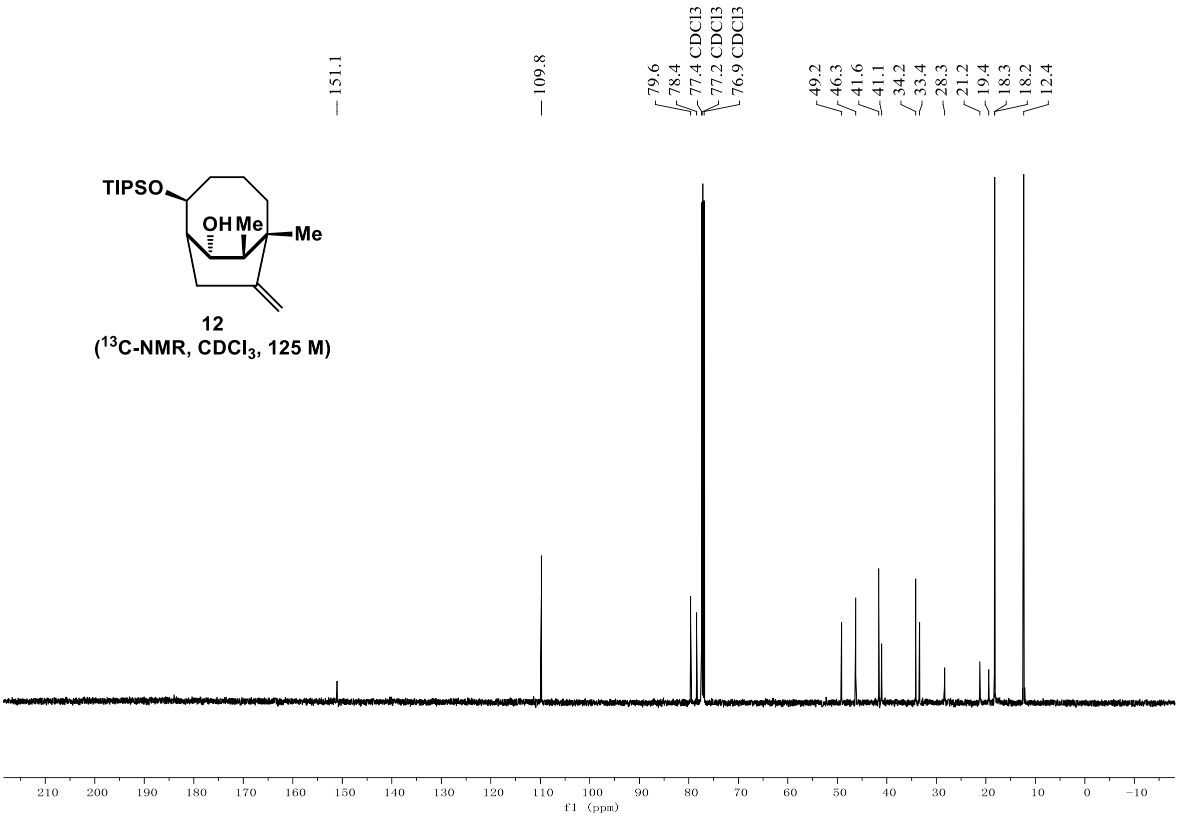
151.1
109.8
79.6
78.4
77.4 CDCl3
77.2 CDCl3
76.9 CDCl3
49.2
46.3
41.6
41.1
34.2
33.4
28.3
21.2
19.4
18.3
18.2
12.4
TIPSO
OH Me
Me
12
(13C-NMR, CDCl3, 125 M)
f1 (ppm)

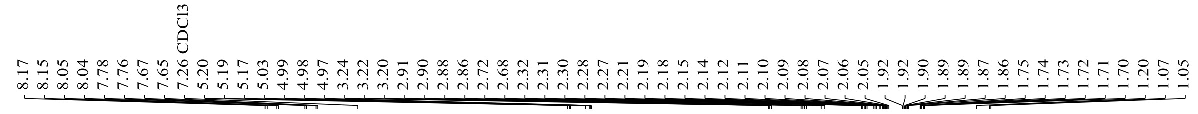

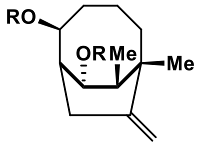

R = *p*-NO$_2$-Bz
12'
(^{1}H-NMR, CDCl$_3$, 500 M)

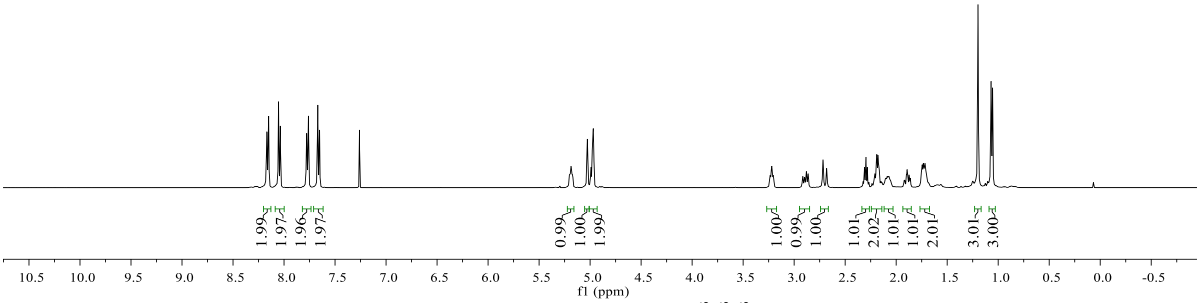

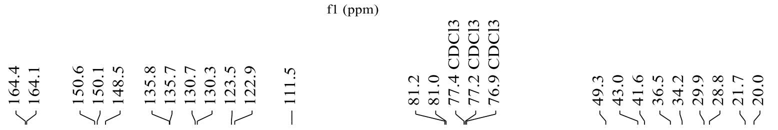

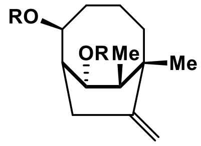

R = *p*-NO$_2$-Bz
12'
(^{13}C-NMR, CDCl$_3$, 125 M)

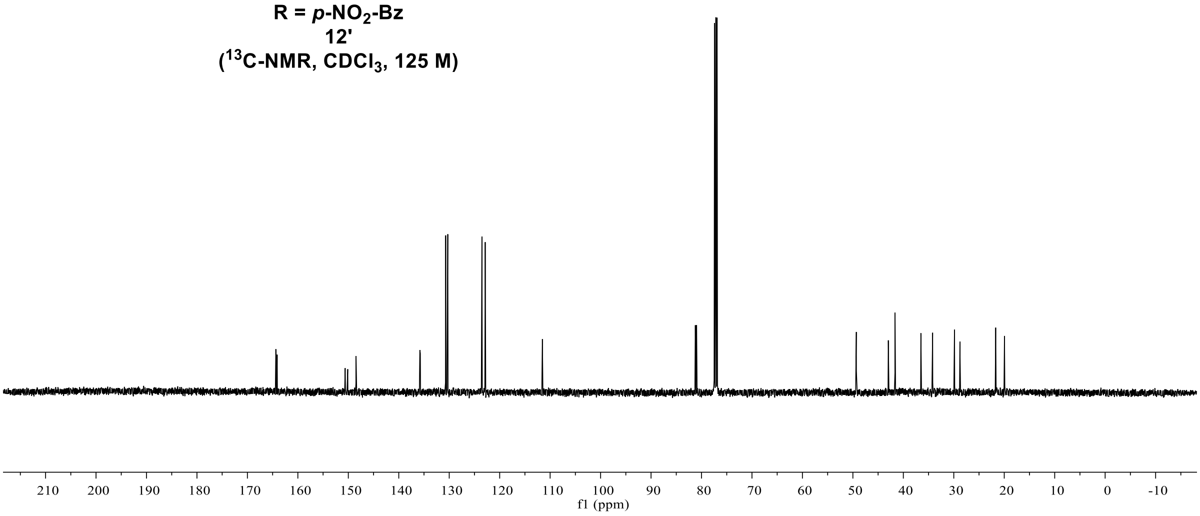

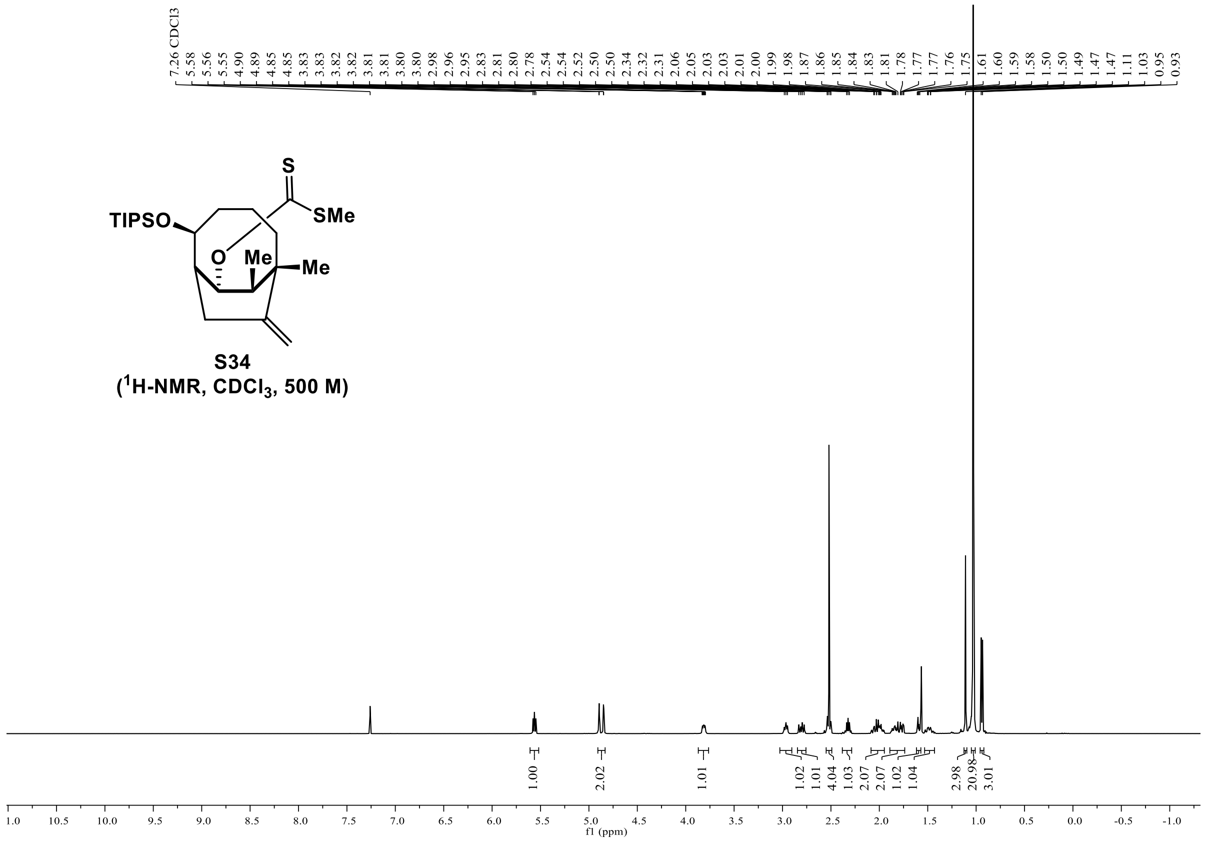
7.26 CDCl3
5.58
5.56
5.55
4.90
4.89
4.85
4.85
3.83
3.83
3.82
3.81
3.81
3.80
3.80
2.98
2.96
2.95
2.83
2.81
2.80
2.78
2.54
2.54
2.52
2.50
2.50
2.34
2.32
2.31
2.06
2.05
2.03
2.03
2.01
2.00
1.99
1.98
1.87
1.86
1.85
1.84
1.83
1.81
1.78
1.77
1.77
1.76
1.75
1.61
1.60
1.59
1.58
1.50
1.49
1.47
1.47
1.11
1.03
0.95
0.93
TIPSO
S
SMe
O
Me
Me
S34
(^{1}H-NMR, $CDCl_3$, 500 M)
1.00
2.02
1.01
1.02
1.01
4.04
1.03
2.07
2.07
1.02
1.04
2.98
20.98
3.01
f1 (ppm)

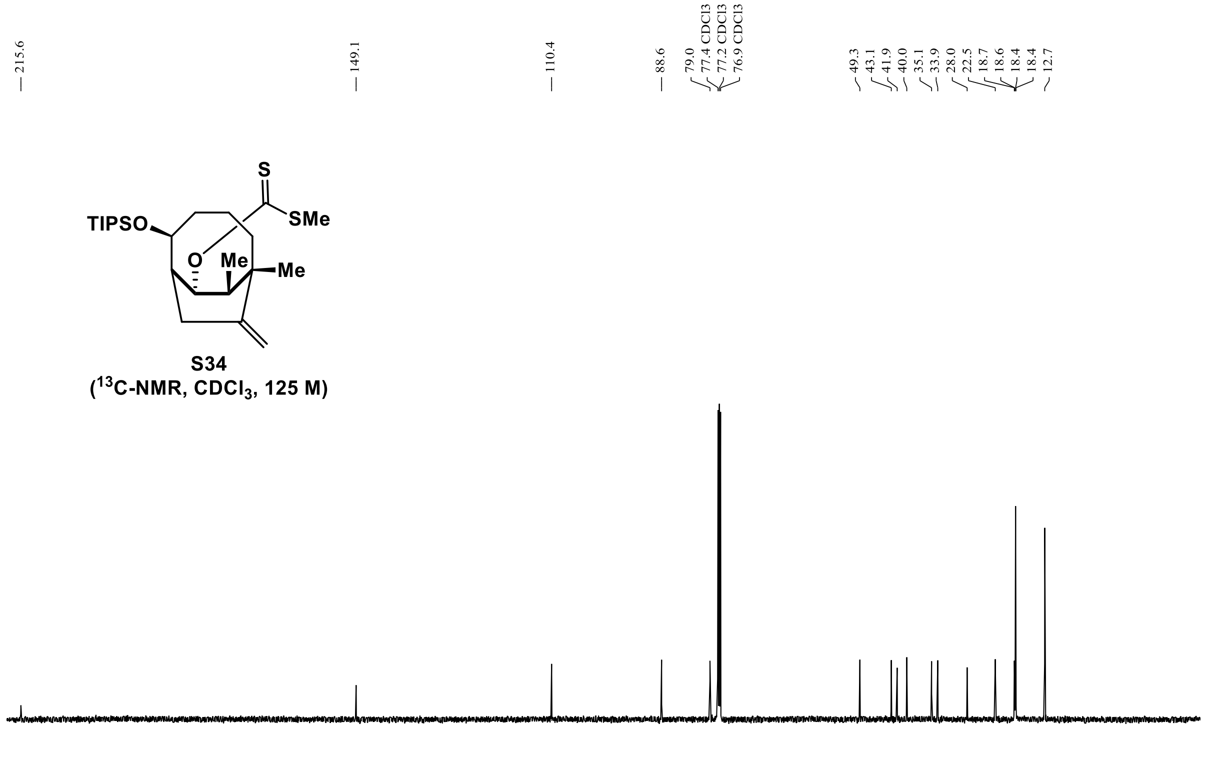
215.6
149.1
110.4
88.6
79.0
77.4 CDCl3
77.2 CDCl3
76.9 CDCl3
49.3
43.1
41.9
40.0
35.1
33.9
28.0
22.5
18.7
18.6
18.4
18.4
12.7
TIPSO
S
SMe
O
Me
Me
S34
(^{13}C-NMR, $CDCl_3$, 125 M)
f1 (ppm)

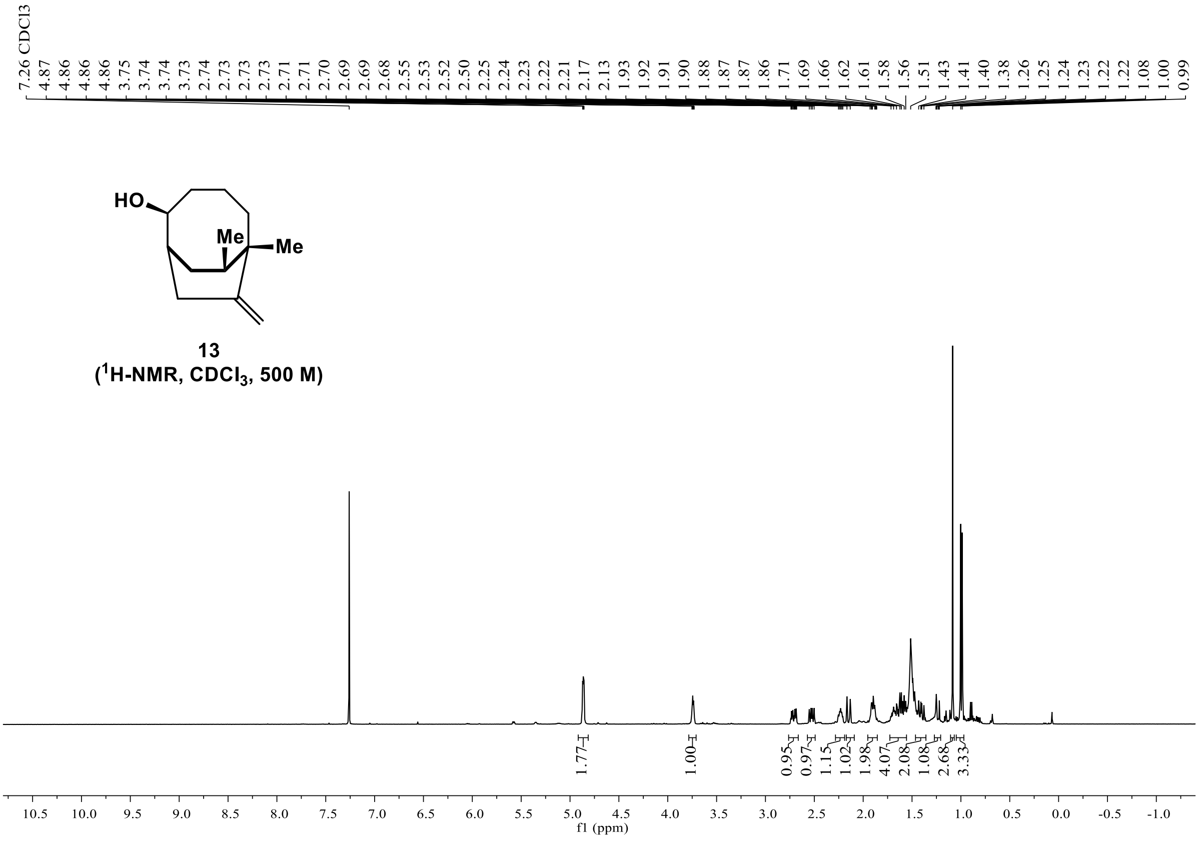
7.26 CDCl3
4.87 4.86 4.86 4.86 3.75 3.74 3.74 3.73 2.74 2.73 2.73 2.73 2.71 2.71 2.70 2.69 2.69 2.68 2.55 2.53 2.52 2.50 2.25 2.24 2.23 2.22 2.21 2.17 2.13 1.93 1.92 1.91 1.90 1.88 1.87 1.87 1.86 1.71 1.69 1.66 1.62 1.61 1.58 1.56 1.51 1.43 1.41 1.40 1.38 1.26 1.25 1.24 1.23 1.22 1.22 1.08 1.00 0.99
HO
Me
Me
13
(^{1}H-NMR, CDCl$_3$, 500 M)
1.77 1.00 0.95 0.97 1.15 1.02 1.98 4.07 2.08 1.08 2.68 3.33
f1 (ppm)

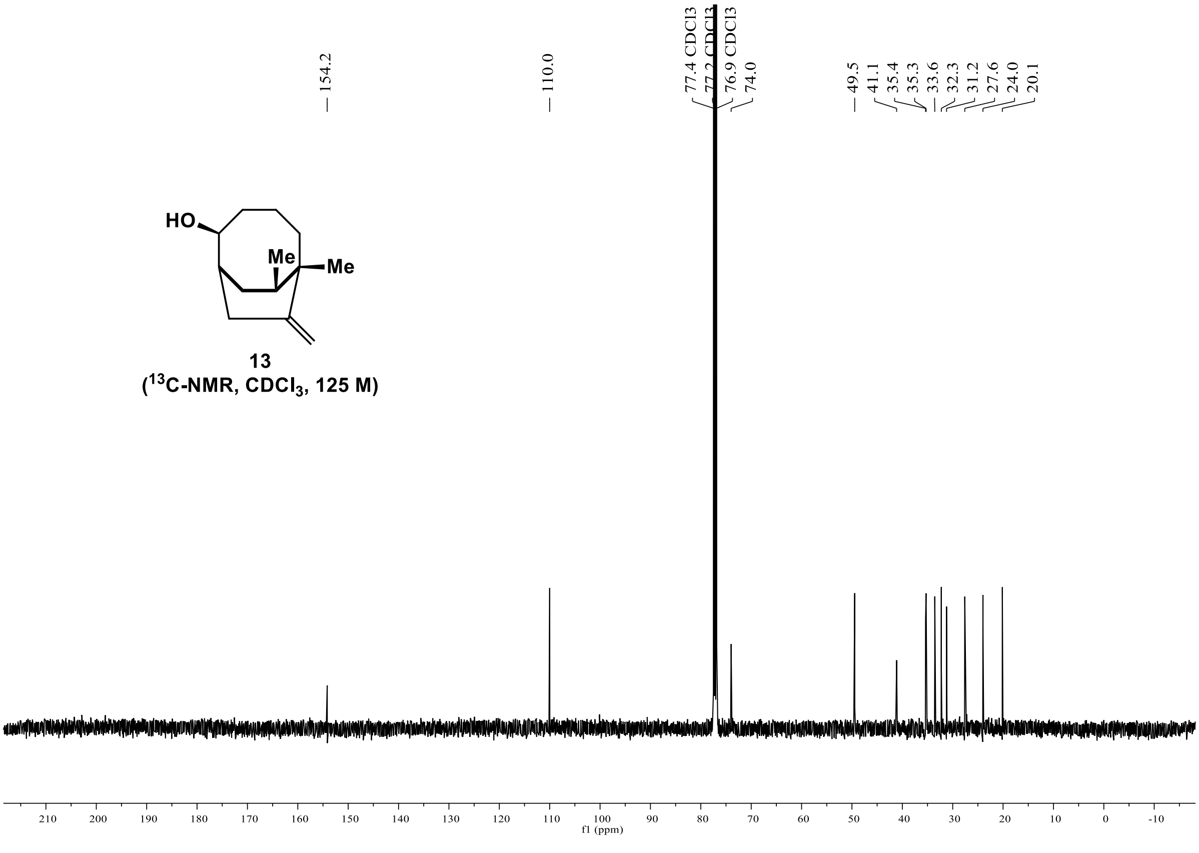
154.2
110.0
77.4 CDCl3
77.2 CDCl3
76.9 CDCl3
74.0
49.5 41.1 35.4 35.3 33.6 32.3 31.2 27.6 24.0 20.1
HO
Me
Me
13
(^{13}C-NMR, CDCl$_3$, 125 M)
f1 (ppm)

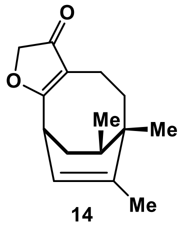

14
(^{1}H-NMR, $CDCl_3$, 500 M)

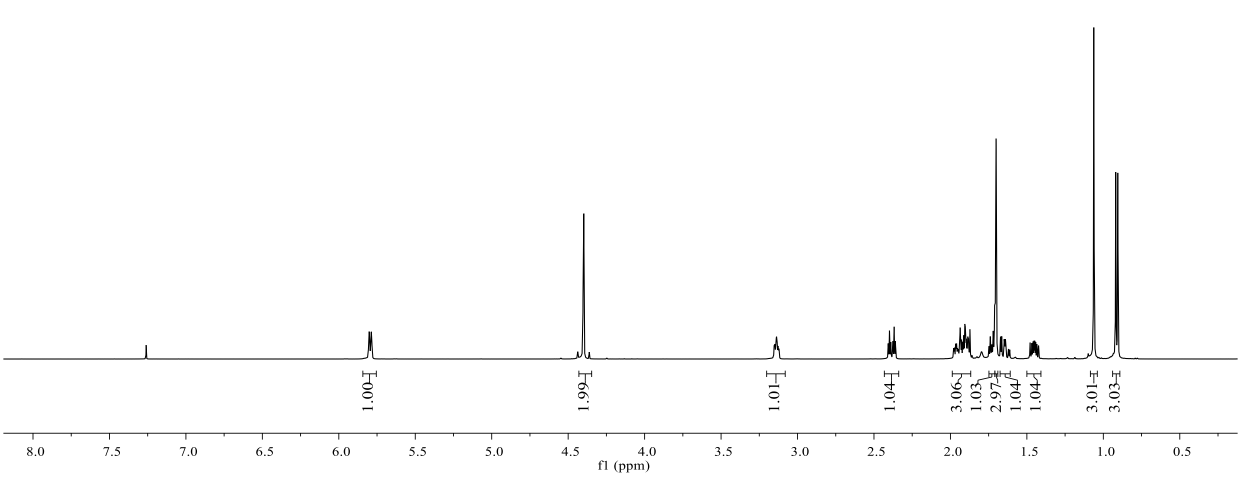

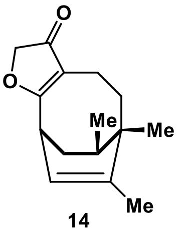

14
(^{13}C-NMR, $CDCl_3$, 125 M)

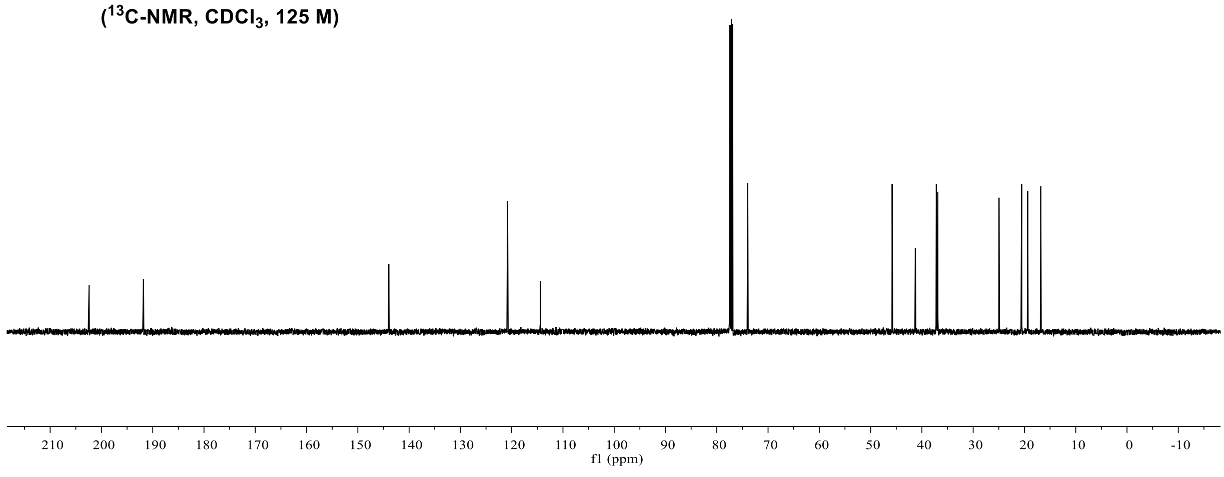

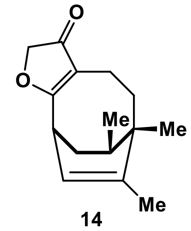

14
(dept 135, $CDCl_3$, 125 M)

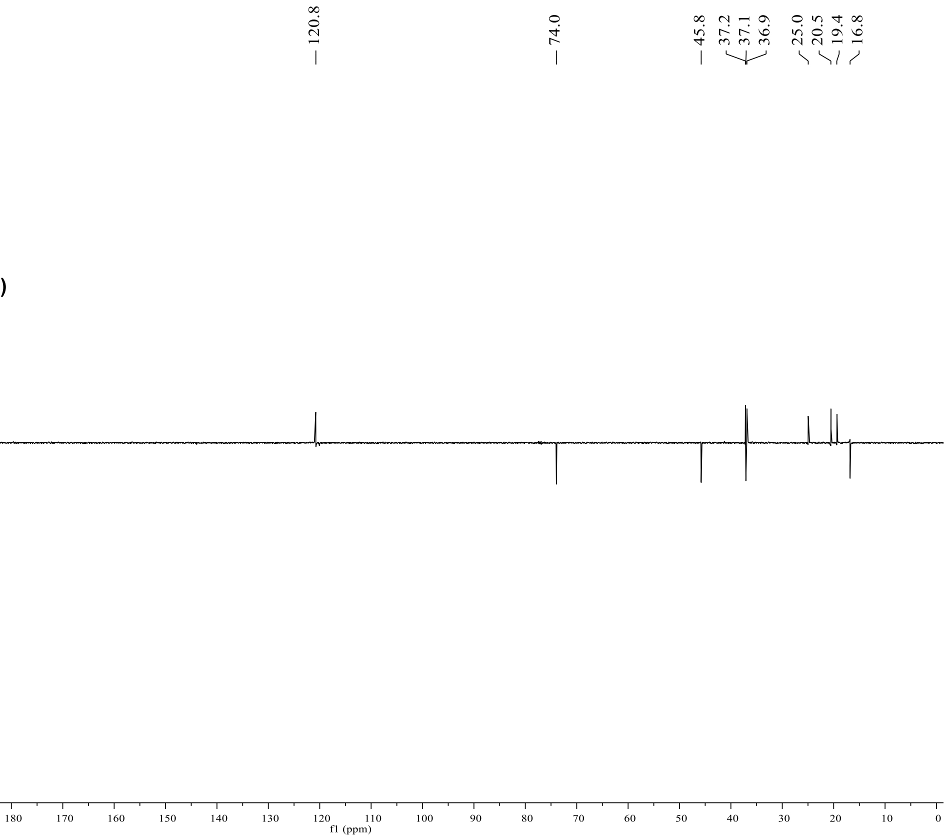

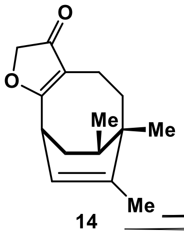

14
(H-H cosy, $CDCl_3$, 500 M)

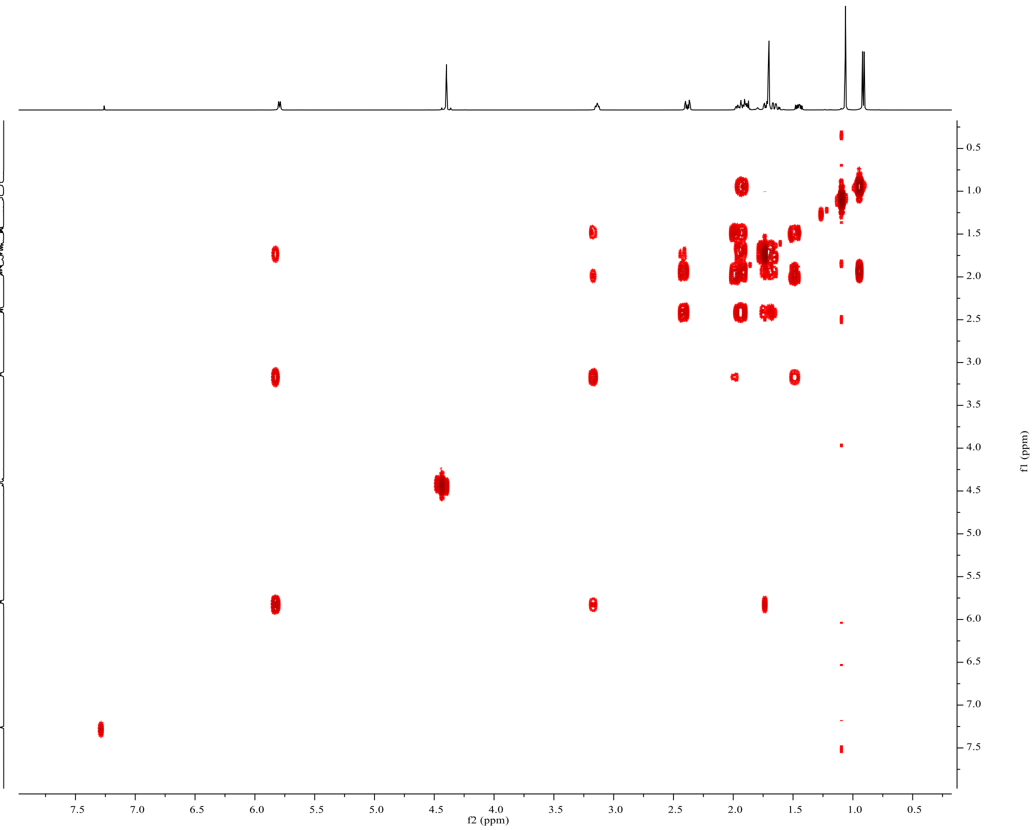

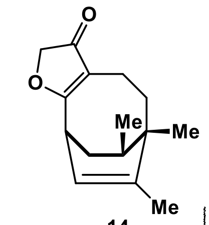

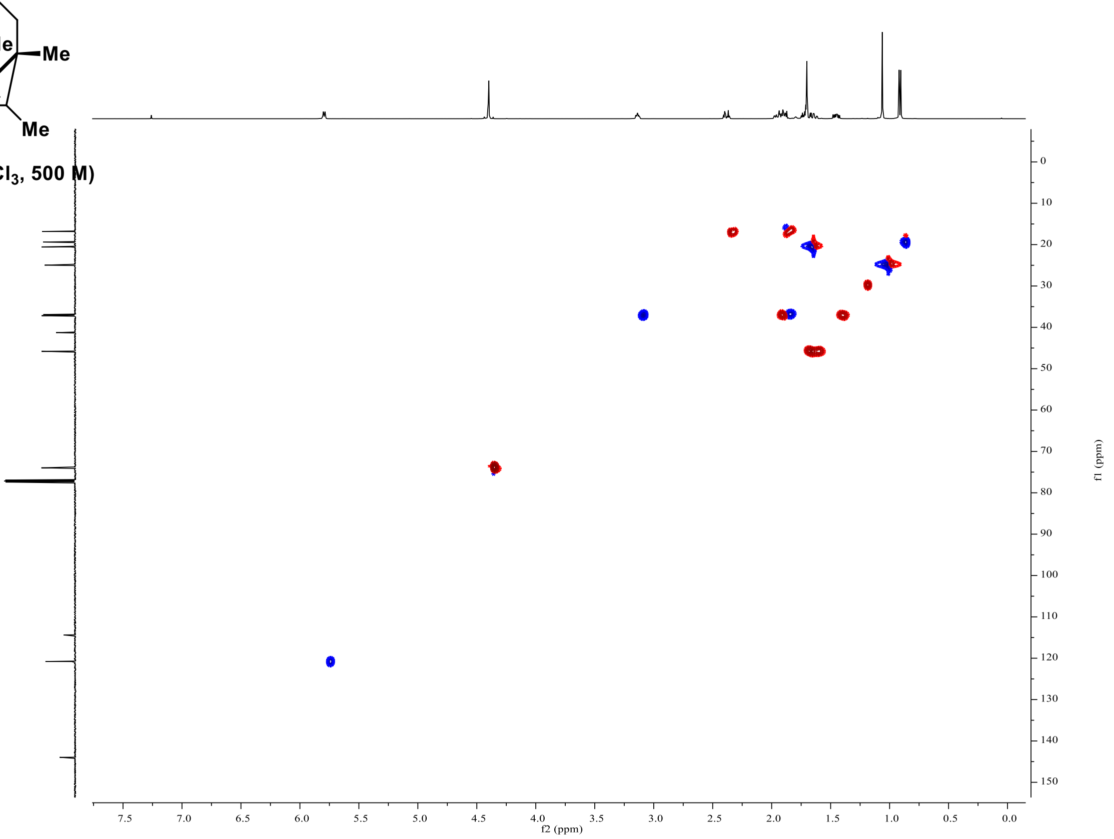

14
(HSQC, $CDCl_3$, 500 M)

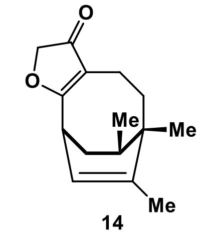

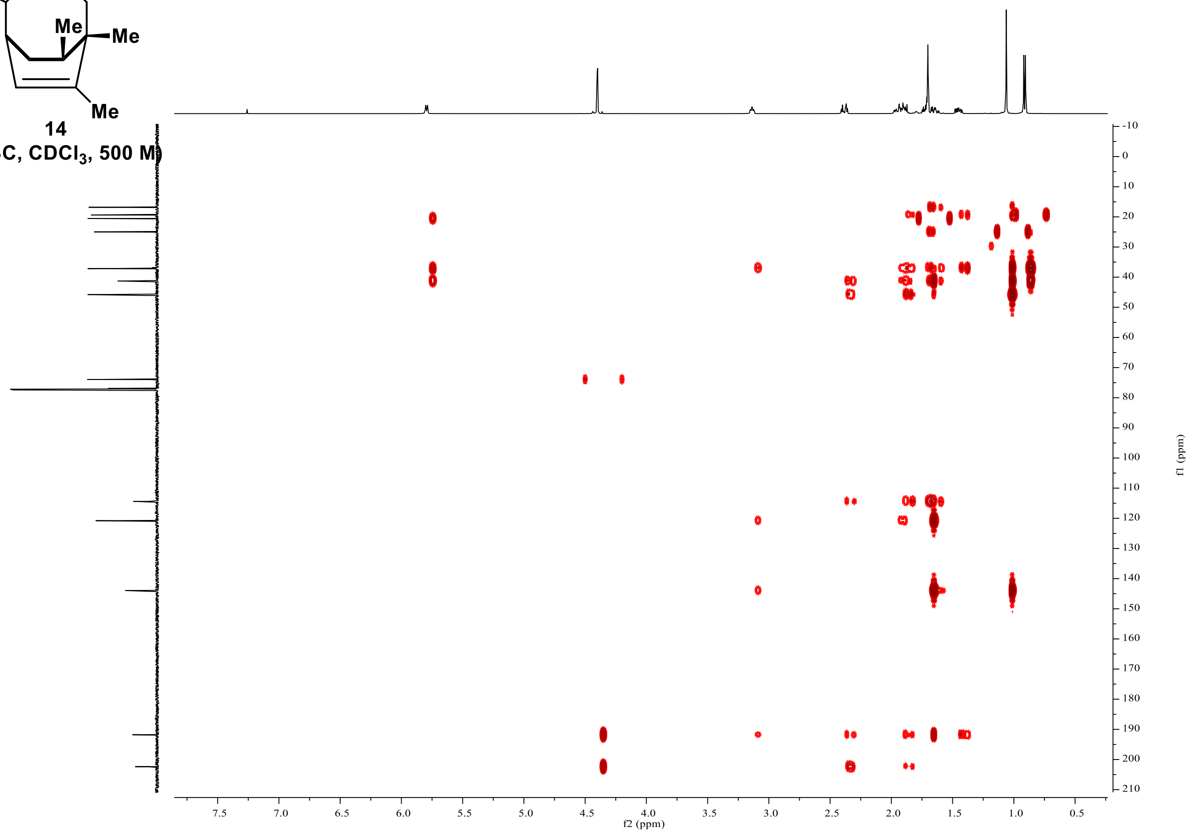

14
(HMBC, $CDCl_3$, 500 M)

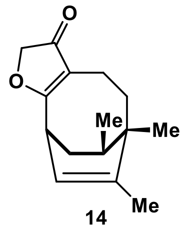

14
(NOE, $CDCl_3$, 500 M)

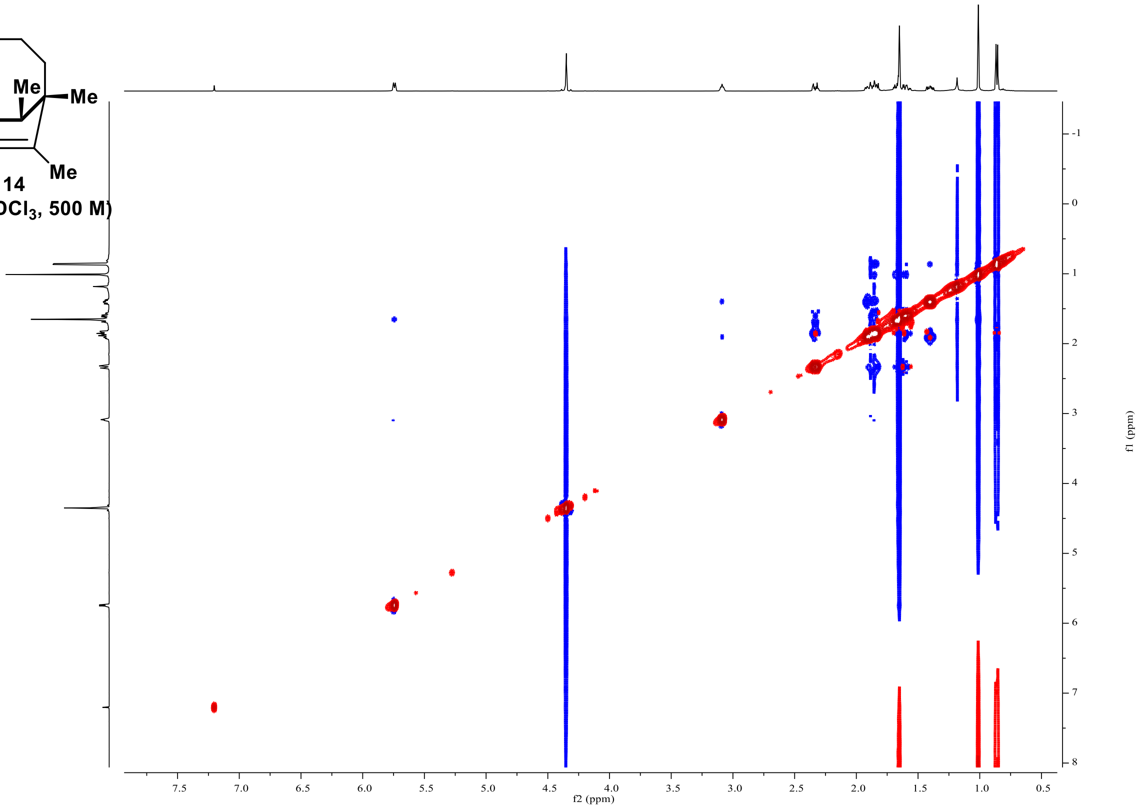

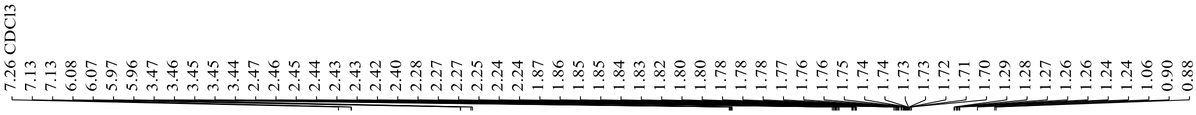

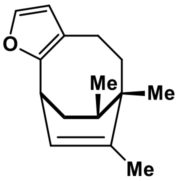

Nakafuran-8
(^{1}H-NMR, $CDCl_3$, 500 M)

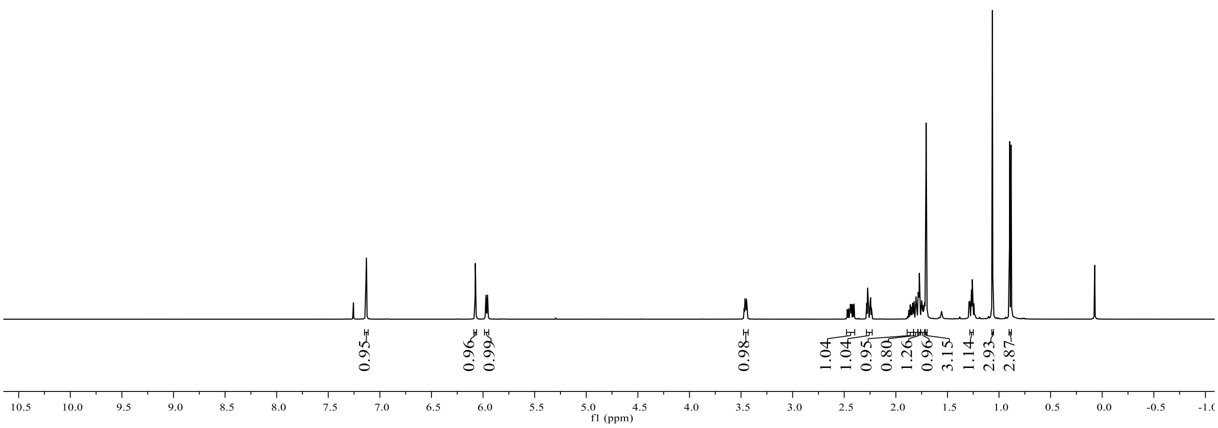

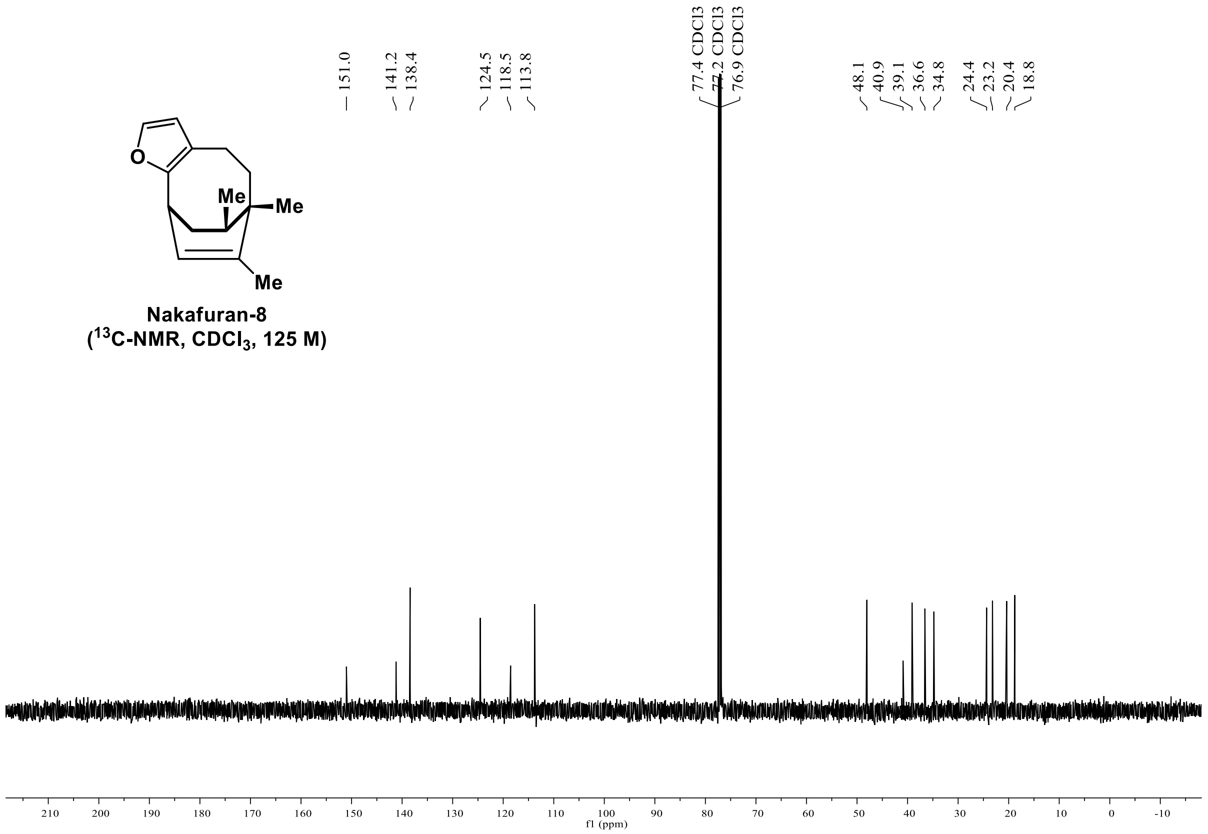

Nakafuran-8
(^{13}C-NMR, $CDCl_3$, 125 M)

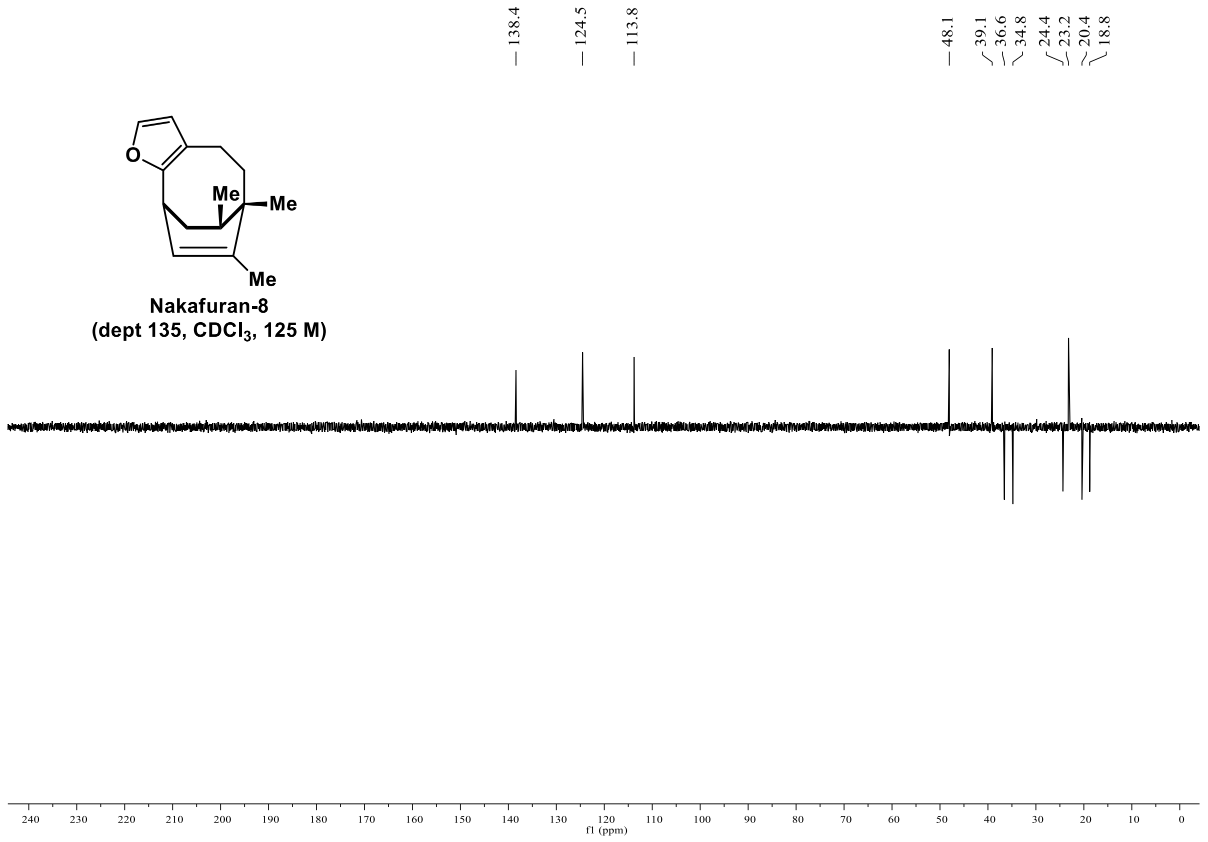

Nakafuran-8
(dept 135, $CDCl_3$, 125 M)

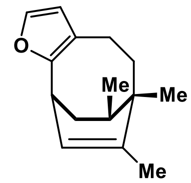

Nakafuran-8
(H-H cosy, $CDCl_3$, 500 M)

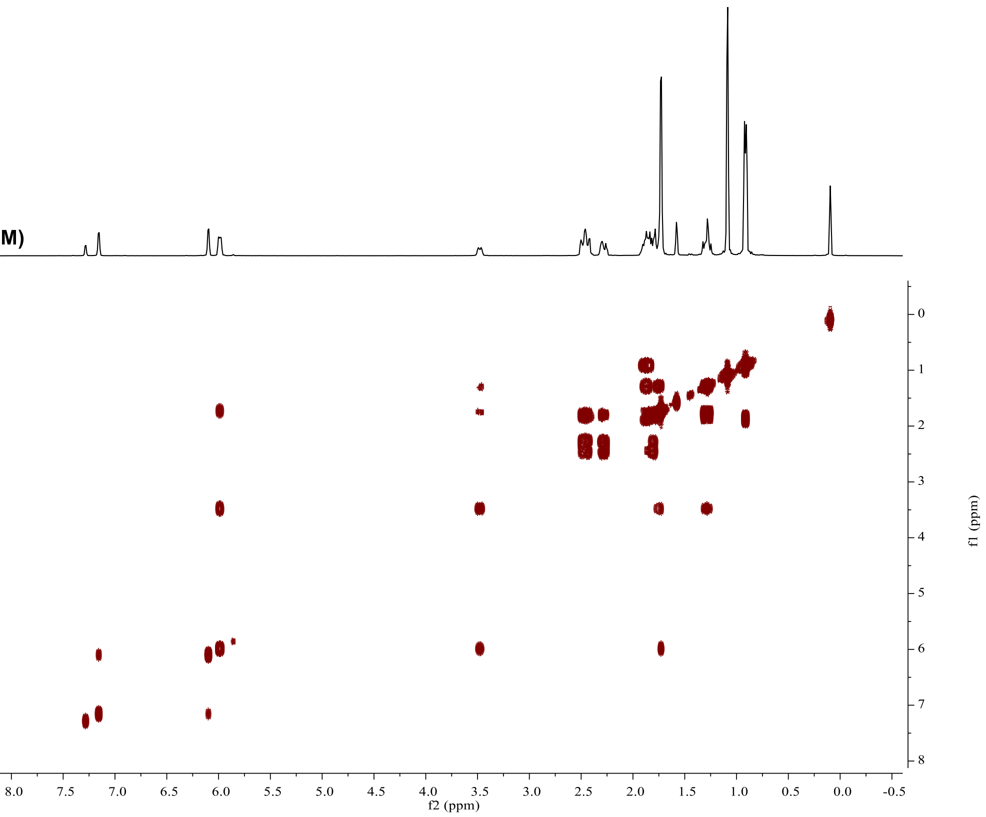

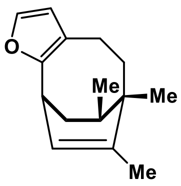

Nakafuran-8
(HSQC, $CDCl_3$, 500 M)

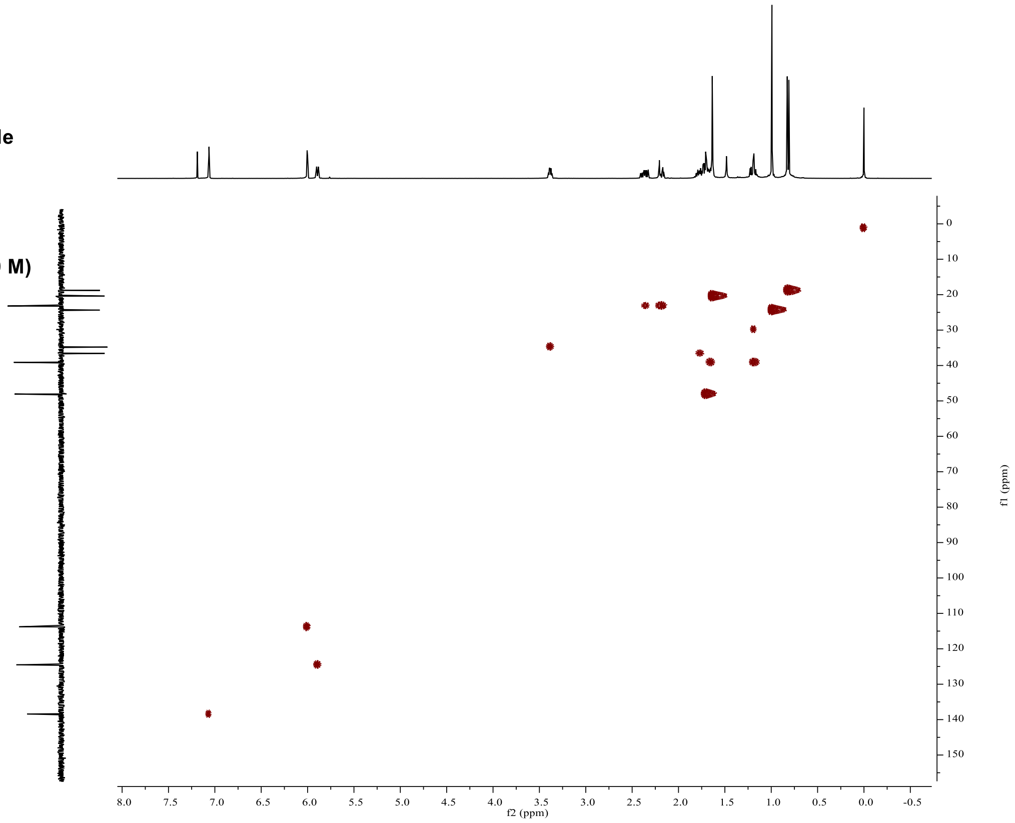

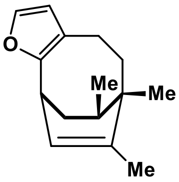

Nakafuran-8
(HMBC, $CDCl_3$, 500 M)

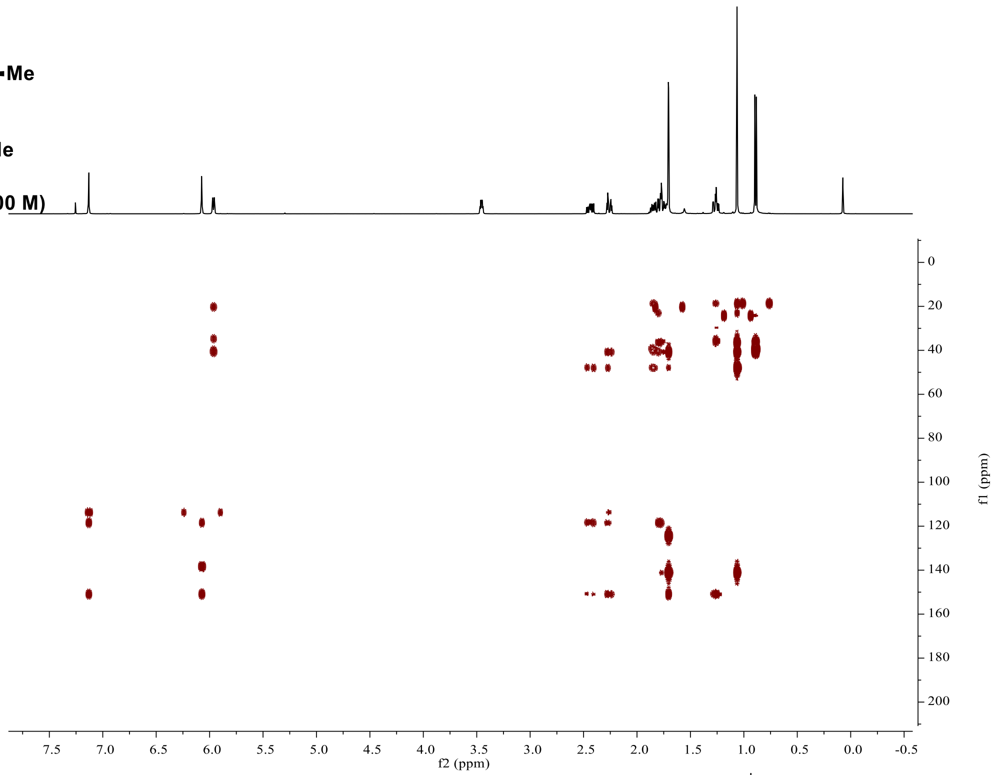

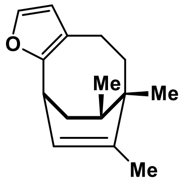

Nakafuran-8
(NOE, $CDCl_3$, 500 M)

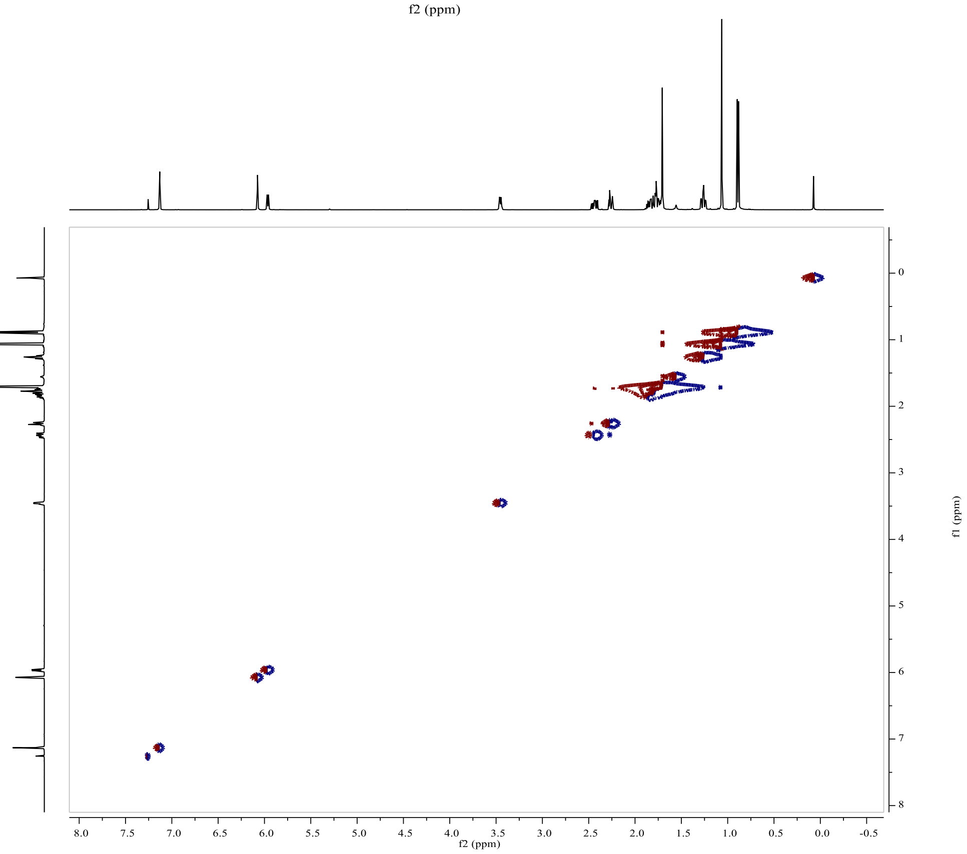

8. References

1. Bailey, W. F. & Khanolkar, A. D. Construction of a sterically congested carbon framework via 5-hexenyllithium cyclization. Synthesis of (±)-cuparene. *Tetrahedron* **47**, 7727-7738 (1991).
2. Bailey, W. F. et al. Stereoselectivity of cyclization of substituted 5-hexen-1-yllithiums: regiospecific and highly stereoselective insertion of an unactivated alkene into a carbon-lithium bond. *J. Am. Chem. Soc.* **113**, 5720-5727 (1991).
3. Gwaltney, S. L., Sakata, S. T. & Shea, K. J. Bridged to fused ring interchange. Methodology for the construction of fused cycloheptanes and cyclooctanes. Total syntheses of ledol, ledene, and compressanolide. *J. Org. Chem.* **61**, 7438-7451 (1996).
4. Hu, X. S. et al. Regioselective markovnikov hydrodifluoroalkylation of alkenes using difluoroenoxysilanes. *Nature Commun.* **11**, 5500-5508 (2020).
5. Li, J., Preinfalk, A. & Maulide, N. Enantioselective redox-neutral coupling of aldehydes and alkenes by an iron-catalyzed "catch–release" tethering approach. *J. Am. Chem. Soc.* **141**, 143-147 (2019).
6. Ojima, D. et al. Total synthesis of miuraenamides A and D. *J. Org. Chem.* **81**, 9886-9894 (2016).
7. Kita, Y., Yata, T., Nishimoto, Y. & Yasuda, M. Indium catalyzed hydrofunctionalization of styrene derivatives bearing a hydroxy group with organosilicon nucleophiles. *J. Org. Chem.* **83**, 740-753 (2018).
8. Selmani, A. & Darses, S. Enantioenriched 1-tetralones via rhodium-catalyzed arylative cascade desymmetrization/acylation of alkynylmalonates. *Org. Lett.* **21**, 8122-8126 (2019).
9. Larock, R. C., Yang, H., Weinreb, S. M. & Herr, R. J. Synthesis of pyrrolidines and piperidines via palladium-catalyzed coupling of vinylic halides and olefinic sulfonamides. *J. Org. Chem.* **59**, 4172-4178 (1994).
10. Teixeira, L. H. P., Barreiro, E. J. & Fraga, C. A. M. Reduction of 2-alkyl-2-carbomethoxy-cyclopentanone derivatives with sodium borohydride. II. The elucidation of the diastereoselective control. *Synth. Commun.* **27**, 3241-3257 (1997).
11. Lyu, M. Y., Zhong Z., Lo V. K. Y., Wong H. N. C. & Peng, X. S. Total synthesis of cryptotrione. *Angew. Chem. Int. Ed. Engl.* **59**, 19929-19933 (2020).
12. Schulte, G., Scheuer, P. J. & McConnell, O. J. Two furanosesquiterpene marine metabolites with antifeedant properties. *Helv. Chim. Acta.* **63**, 2159-2167 (1980).
13. Uyehara, T., Sugimoto, M., Suzuki, I. & Yamamoto, Y. Rearrangement approaches to cyclic skeletons. part 8. total synthesis of (±)-nakafuran-8, a marine metabolite with antifeedant properties, on the basis of bridgehead substitution of a bicyclo[2.2.2]oct-5-en-2-one system. *J.*

Chem. Soc., Perkin Trans. 1 1785-1788 (1992).

14. Vintonyak, V. V. & Maier, M. E. Synthesis of the core structure of cruentaren A. *Org. Lett.* **9**, 655-658 (2007).
15. Parsons, D. E. & Frontier, A. J. Noncanonical cation-π cyclizations of alkylidene β-ketoesters: synthesis of spiro-fused and bridged bicyclic ring systems. *Org. Lett.* **21**, 2008-2012 (2019).
16. Crossley, S. W. M., Barabé, F. & Shenvi, R. A. Simple, chemoselective, catalytic olefin isomerization. *J. Am. Chem. Soc.* **136**, 16788-16791 (2014).
17. Gaussian 16, Revision C.01. Frisch, M. J., Trucks, G. W., Schlegel, H. B., Scuseria, G. E., Robb, M. A., Cheeseman, J. R., Scalmani, G., Barone, V., Petersson, G. A., Nakatsuji, H., Li, X., Caricato, M., Marenich, A. V., Bloino, J., Janesko, B. G., Gomperts, R., Mennucci, B., Hratchian, H. P., Ortiz, J. V., Izmaylov, A. F., Sonnenberg, J. L., Williams-Young, D., Ding, F., Lipparini, F., Egidi, F., Goings, J., Peng, B., Petrone, A., Henderson, T., Ranasinghe, D., Zakrzewski, V. G., Gao, J., Rega, N., Zheng, G., Liang, W., Hada, M., Ehara, M., Toyota, K., Fukuda, R., Hasegawa, J., Ishida, M., Nakajima, T., Honda, Y., Kitao, O., Nakai, H., Vreven, T., Throssell, K., Montgomery, J. A., Jr., Peralta, J. E., Ogliaro, F., Bearpark, M. J., Heyd, J. J., Brothers, E. N., Kudin, K. N., Staroverov, V. N., Keith, T. A., Kobayashi, R., Normand, J., Raghavachari, K., Rendell, A. P., Burant, J. C., Iyengar, S. S., Tomasi, J., Cossi, M., Millam, J. M., Klene, M., Adamo, C., Cammi, R., Ochterski, J. W., Martin, R. L., Morokuma, K., Farkas, O., Foresman, J. B. & Fox, D. J. Gaussian, Inc., Wallingford CT (2016).
18. Chai, J.-D. & Head-Gordon, M. Long-range corrected hybrid density functionals with damped atom-atom dispersion corrections. *Phys. Chem. Chem. Phys.* **10**, 6615-6620 (2008).
19. Barone, V. & Cossi, M. Quantum calculation of molecular energies and energy gradients in solution by a conductor solvent model. *J. Phys. Chem. A.* **102**, 1995-2001 (1998).
20. Cossi, M., Rega, N., Scalmani, G. & Barone, V. Energies, structures, and electronic properties of molecules in solution with the C-PCM solvation model. *J. Comput. Chem.* **24**, 669-681 (2003).
21. Takano, Y. & Houk, K. N. Benchmarking the conductor-like polarizable continuum model (CPCM) for aqueous solvation free energies of neutral and ionic organic molecules. *J. Chem. Theory Comput.* **1**, 70-77 (2005).
22. Grimme, S. et al. Fully automated quantum-chemistry-based computation of spin-spin- coupled nuclear magnetic resonance spectra. *Angew. Chem. Int. Ed.* **56**, 14763-14769 (2017).
23. Grimme, S. Exploration of chemical compound, conformer, and reaction space with meta-dynamics simulations based on tight-binding quantum mechanical calculations. *J. Chem. Theory Comput.* **155**, 2847-2862 (2019).
24. Grimme, S., Bannwarth, C. & Shushkov, P. A robust and accurate tight-binding quantum

chemical method for structures, vibrational frequencies, and noncovalent interactions of large molecular systems parameterized for all spd-block elements (Z = 1-86). *J. Chem. Theory Comput.* **13**, 1989-2009 (2017).

25. Bannwarth, C., Ehlert, S. & Grimme, S. GFN2-xTB–an accurate and broadly parameterized self-consistent tight-binding quantum chemical method with multipole electrostatics and density-dependent dispersion contributions. *J. Chem. Theory Comput.* **15**, 1652-1671 (2019).
26. Pracht, P., Caldeweyher, E., Ehlert, S. & Grimme, S. A robust non-self-consistent tight-binding quantum chemistry method for large molecules. *ChemRxiv*, (2019)**.** preprint. DOI: 10.26434/chemrxiv.8326202.v1
27. Legault, C. Y. CYLview, 1.0b; Université de Sherbrooke (2009). http://www.cylview.org.
